# Iridium-Catalyzed Stereoselective α‑Alkylation of α‑Hydroxy Ketones with Minimally Polarized Alkenes

**DOI:** 10.1021/jacs.5c19724

**Published:** 2026-01-20

**Authors:** Yihong Wang, Fenglin Hong, Craig M. Robertson, Richard J. Mudd, John F. Bower

**Affiliations:** Department of Chemistry, 4591 University of Liverpool, Crown Street, Liverpool L69 7ZD, United Kingdom

## Abstract

Cationic Ir­(I)-complexes modified with homochiral diphosphines promote the α-C-H addition of α-hydroxy ketones to styrenes or alkyl olefins. These processes are predicated on the hydroxyl-directed formation of an Ir-enolate. Inter- and intramolecular processes are feasible, with the latter offering stereocontrolled access to carbocycles bearing two new stereocenters. The intramolecular processes constitute rare examples of alkene-based Conia-ene reactions that are enantio- and diastereoselective.

## Graphical abstract

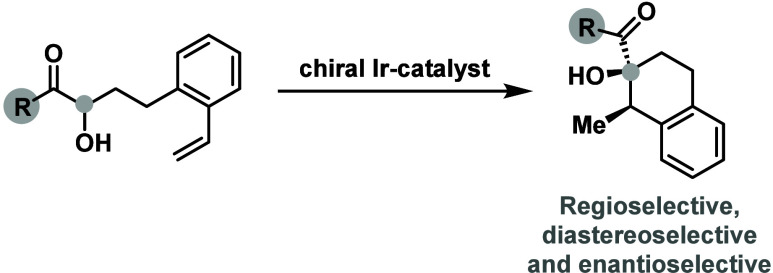

We are engaged in a program that aims to develop stereocontrolled α-alkylations of monocarbonyl compounds with low polarity and minimally activated alkenes (e.g., styrenes and α-olefins) (Scheme 1A).^1–3^ This approach offers an attractive framework for designing new types of C­(sp^3^)-C­(sp^3^) cross-coupling because (a) it avoids prefunctionalization, (b) it is atom economical and (c) it uses feedstock or readily available alkenes as the electrophilic component.^4–6^ Unfortunately, this area has proven to be exceptionally challenging, and this reflects wider difficulties in realizing “direct” (i.e., avoiding stoichiometric enolate formation) enantioselective α-alkylations of monocarbonyl compounds.^7,8^ Within this context, a significant milestone is MacMillan and co-workers’ tricatalytic system that allows the *linear selective* and enantioselective direct α-alkylation of aldehydes with alkenes via a radical addition pathway (Scheme 1B, eq 1).^9^ More recently, we have developed unusual methods that are based on the directing group-mediated generation of Ir-enolates (eq 2).^2,10^ Significantly, this framework allows minimally polarized alkenes to engage in enantioselective, diastereoselective and *branched selective* α-alkylation processes. The methods we have developed so far use an α- or β-aryl-amino directing group, and, in so doing, offer direct entries to complex amino acid derivatives. Expansion of this approach requires the identification of other types of “native” directing group that might mediate Ir-enolate formation. This led us to question whether α-hydroxy ketones could be effective, as these could potentially promote C–C bond formation via an Ir-enediolate (eq 3). Outlined below are our proof-of-concept studies in this area, which have resulted in interesting intermolecular cross-couplings and powerful intramolecular processes. The latter constitute rare examples of alkene-based Conia-ene reactions that are enantio- and diastereoselective, providing a stereocontrolled framework for the assembly of complex carbocyclic systems.^11,12^

**Scheme 1:**
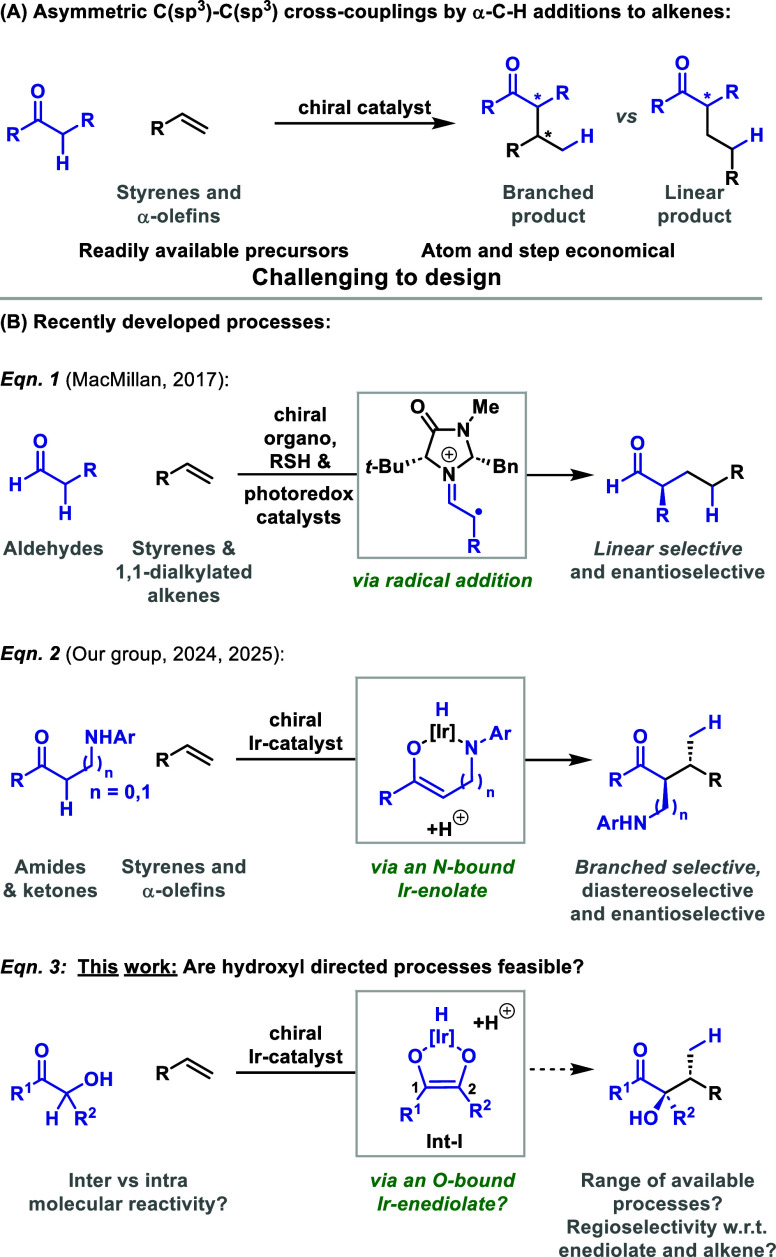
Introduction

Preliminary investigations focused on intermolecular processes (Scheme 2). Reaction of ketone **1a** with styrene **2** using Ir­(cod)_2_BARF/BINAP at 100 °C in dioxane gave tautomeric products **3a** and *iso* **-3a** in a 4:3 ratio and 50% yield (eq 4). In both cases, exclusive branched selectivity was observed. Despite extensive efforts we were unable to establish conditions that offered high selectivity for one or other tautomer. Accordingly, we evaluated methyl-substituted system **1b** because the expected products of this process cannot tautomerize (eq 5). In the event, this provided branched products **3b** and *iso-* **3b** in 43% yield. This outcome is consistent with alkylation of the putative Ir-enediolate **Int-I** at either C1 or C2 (see Scheme 1B, eq 3). Interestingly, we also observed the formation of linear adduct **4b′**, which, although speculative, may arise via α-ketol rearrangement of linear alkylation product **4b**;^13^ evidence supporting this is provided later. To address the issue of Ir-enediolate alkylation regioselectivity, we next examined the reaction of **1c** with styrene **2**, because this process would proceed via a symmetrical Ir-enediolate (eq 6). This provided branched product **3c** in 20% yield along with an 8% yield of α-ketol rearrangement product **3c**′. Interestingly, the major product was linear alkylation adduct **4c** (45% yield) and this was accompanied by rearrangement product **4c′** (18% yield).

**Scheme 2:**
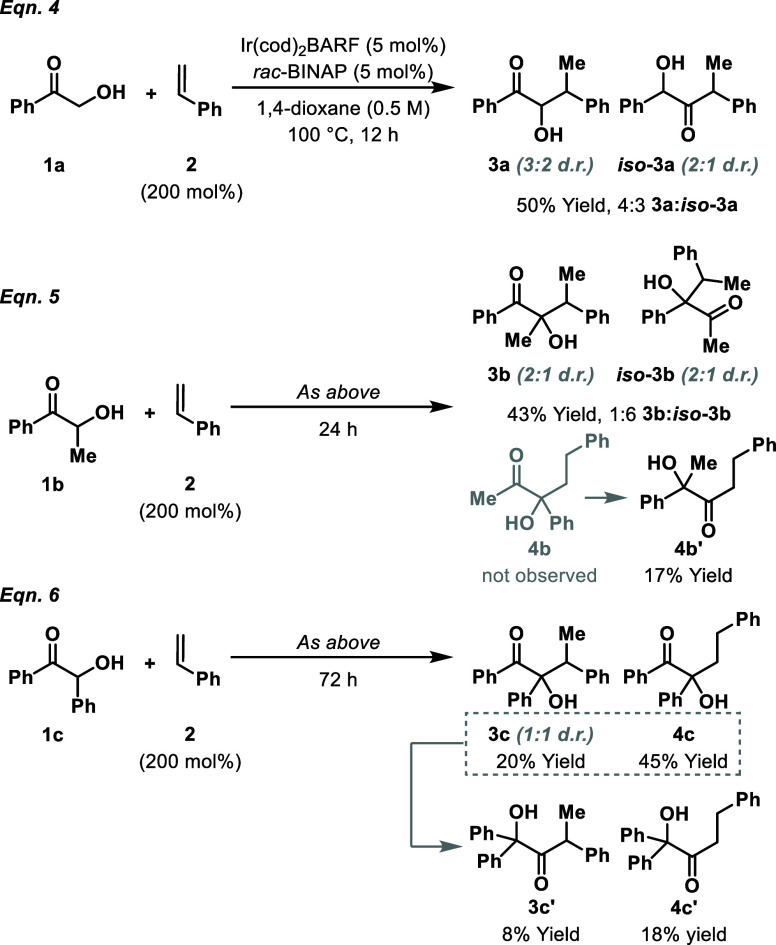
Preliminary Investigations into an Intermolecular Process

The results in Scheme 2 demonstrated the feasibility of the proposed C–C coupling process. However, these intermolecular variants were hampered by three significant issues: (1) variable branched:linear regioselectivity, (2) variable Ir-enediolate alkylation regioselectivity, and (3) competing α-ketol rearrangement of the initial products. To weave this unusual reactivity into a synthetically useful process, we considered whether intramolecularization might circumvent some of these issues. To this end, we designed substrate **5a** on the basis that geometric constraints would lead to a regioselective cyclization (Table 1). Under the conditions outlined in Scheme 2, 6-*exo* cyclization product **6a** and its rearranged isomer *iso* **-6a** were generated in a 1:10 ratio and 51% yield, with the latter formed in 11:1 d.r. (Entry 1). Notably, 5-*exo* and 6-*endo* products **6a′** and **6a′′** were not observed. Ir-systems with less dissociating counterions were unsuccessful (Entries 2 and 3); however, a wide range chiral diphosphines could be employed (Entries 4–10). Of these, (*R*)-SEGPHOS (**L2**) offered the most promise (Entry 4) and so this was advanced through further optimization studies that focused on temperature and reaction solvent. This led to the conditions in Entry 17 that deliver a mixture of **6a** and *iso* **-6a** (>20:1 d.r.) in a 1:10 ratio and 85% yield. These products were not observed in the absence of catalyst (Entry 19) and their ratio was strongly dependent on the reaction temperature, *with the former favored at 60 °C* (Entry 14). Control experiments showed that the rearrangement of **6a** to *iso* **-6a** is most efficient at elevated temperatures and, interestingly, requires the Ir-catalyst (see the SI).

**Table 1:**
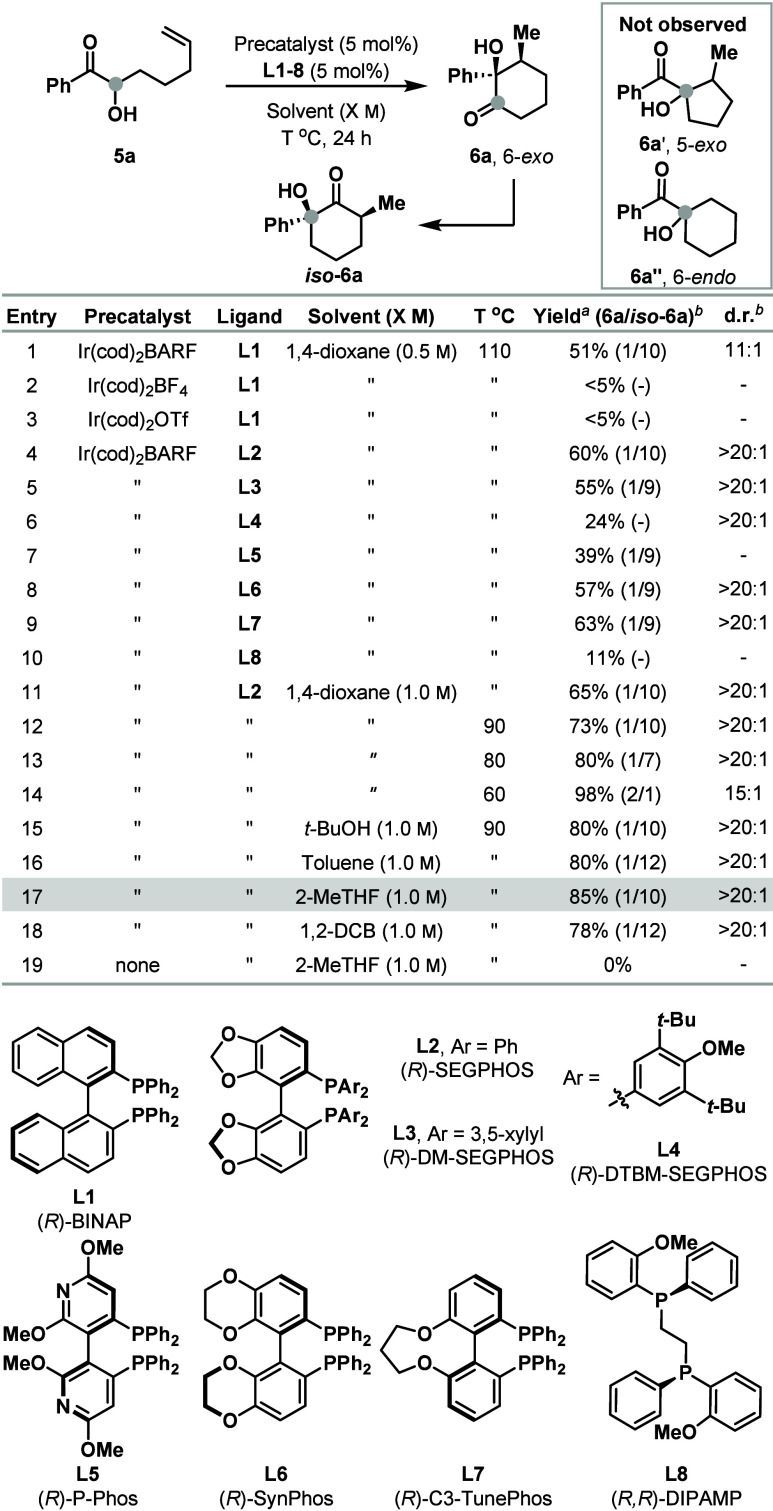
Optimization of an Intramolecular Process^a^

a Isolated yield.

b Determined by ^1^H NMR analysis of the crude mixture.

c 1,2-DCB = 1,2-dichlorobenzene.

The scope of the cyclization process is delineated in Table 2. For systems **5**, where n = 1, exclusive 6-*exo* cyclization was observed in almost all cases to provide **6** or *iso* **-6** with very high diastereoselectivity (generally >20:1 d.r.). To achieve optimal efficiencies and selectivities, fine-tuning of the reaction temperature was required in some cases, primarily to mitigate decomposition pathways associated with elevated temperatures and/or prolonged reaction times. In turn, in some instances procedural modifications were required. For example, the cyclization to form **6b**/*iso* **-6b** was most chemically efficient at 60 °C, but formed a 1:1 mixture of products. These were resubmitted to the reaction conditions to achieve a 1:10 ratio. Conversely, the cyclization of **5e** provided *iso* **-6e** with good selectivity using the standard procedure. In most cases, it was easier to achieve good selectivity for *iso* **-6** over **6** than vice versa. However, for systems with electron poor aryl ketones, the latter product could be formed selectively, with, for example, **5f** cyclizing to provide **6f** with >20:1 selectivity over *iso* **-6f**. This presumably reflects the lower migratory aptitude of the *meta*-CF_3_ aryl unit of **6f** versus e.g. the phenyl unit of **6a**. Interestingly, heteroaryl ketones **5h** and **5i** gave selectively **6h** and **6i**, and minimal rearrangement was observed. System **5k**, which possesses an ether tethered alkene provided unusual results, giving a 1.4:1 ratio of *iso* **-6k:6k′**. The latter arises via competing 5-*exo* cyclization, and this is the only case where we encountered such a process. Precursors **7**, where n = 2, behaved in a more predictable manner and underwent 6-*exo* cyclization to provide **8** in generally moderate to excellent yields, and with very high diastereoselectivities. Certain limitations were identified; for example, methyl ketones **5l** and **7l** were not suitable, and increased or decreased tether lengths were not tolerated (**9** and **10**).

**Table 2:**
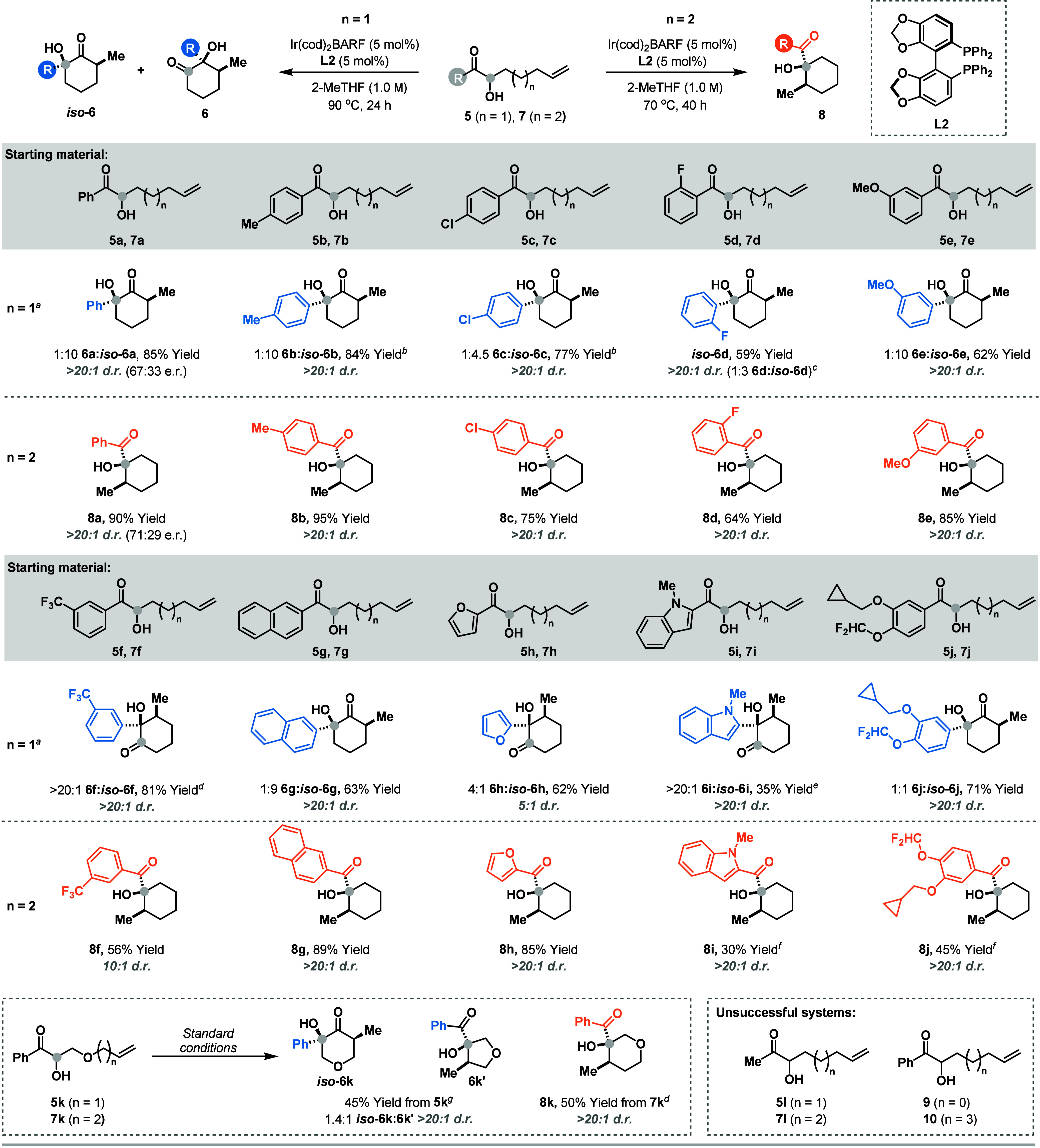
Scope of the Intramolecular Protocol

a Yields are for the mixture of **6:** *iso* **-6** unless stated otherwise; d.r. values refer to the major (depicted) product.

b The reaction was run at 60 °C for 72 h, the products were isolated as 1:1 mixture, and then resubmitted to the standard conditions.

c The yield refers to pure *iso* **-6d**. The selectivity for **6d:** *iso* **-6d** was determined by ^1^H NMR analysis of the crude reaction mixture and is given in parentheses.

d 60 °C for 72 h; the products formed in a 2:1 ratio when the reaction was run at 90 °C for 24 h.

e 70 °C for 72 h; substrate decomposition occurred when the reaction was run at 90 °C.

f 90 °C for 40 h.

g 60 °C for 72 h; substrate decomposition occurred when the reaction was run at 90 °C.

So far, we have been unable to develop highly enantioselective variants of the processes is Table 2, with, for example, *iso* **-6a** and **8a** being formed in 67:33 and 71:29 e.r., respectively, under optimized conditions using **L2**. By contrast, we have found that processes involving styrenic alkenes are more amenable to enantioinduction (Table 3A). For example, using **L6** as the ligand, cyclization of **7m** provided **8m** in 86% yield, > 20:1 d.r. and 97:3 e.r. This result shows that the method can offer high facial selectivity with respect to both the putative Ir-enediolate and the styrenic component, allowing the controlled introduction of challenging contiguous stereocenters. Similarly, other (heteroaryl)­ketones **7n**–**t** provided **8n**–**t** in high yields and with very good diastereo- and enantioselectivities, via subtle variations to temperature and ligand. The structures of **8t** and the 3,5-dinitrobenzoyl ester of **8p** were confirmed by single crystal X-ray diffraction, with the latter allowing the assignment of absolute stereochemistry.^14^ So far, the presence of a styrenic alkene is critical for achieving high levels of enantioselectivity. As outlined in Table 3B, isomeric system **11**, where the alkene is not conjugated to the arene, cyclized efficiently, but provided **12** in only 51:49 e.r. Nevertheless, this result is significant because it validates a distinct class of cyclization, suggesting that many variants may be achievable using the strategy described here. Indeed, **13**, where the alkene is linked via the aryl ketone, rather than the carbonyl α-position, also cyclized to provide **14** with promising levels of efficiency. Compound **15**, which is the desmethylene analogue of **7**, underwent an intriguing cyclization process to provide 2-naphthol **17** and isomeric system *iso* **-17** in 63% and 21% yield, respectively. The former presumably arises via dehydration-enolization of initial cyclization product **16**, whereas the latter requires prior intervention of an α-ketol rearrangement. Clearly, there is potential to develop these types of process further.

**Table 3:**
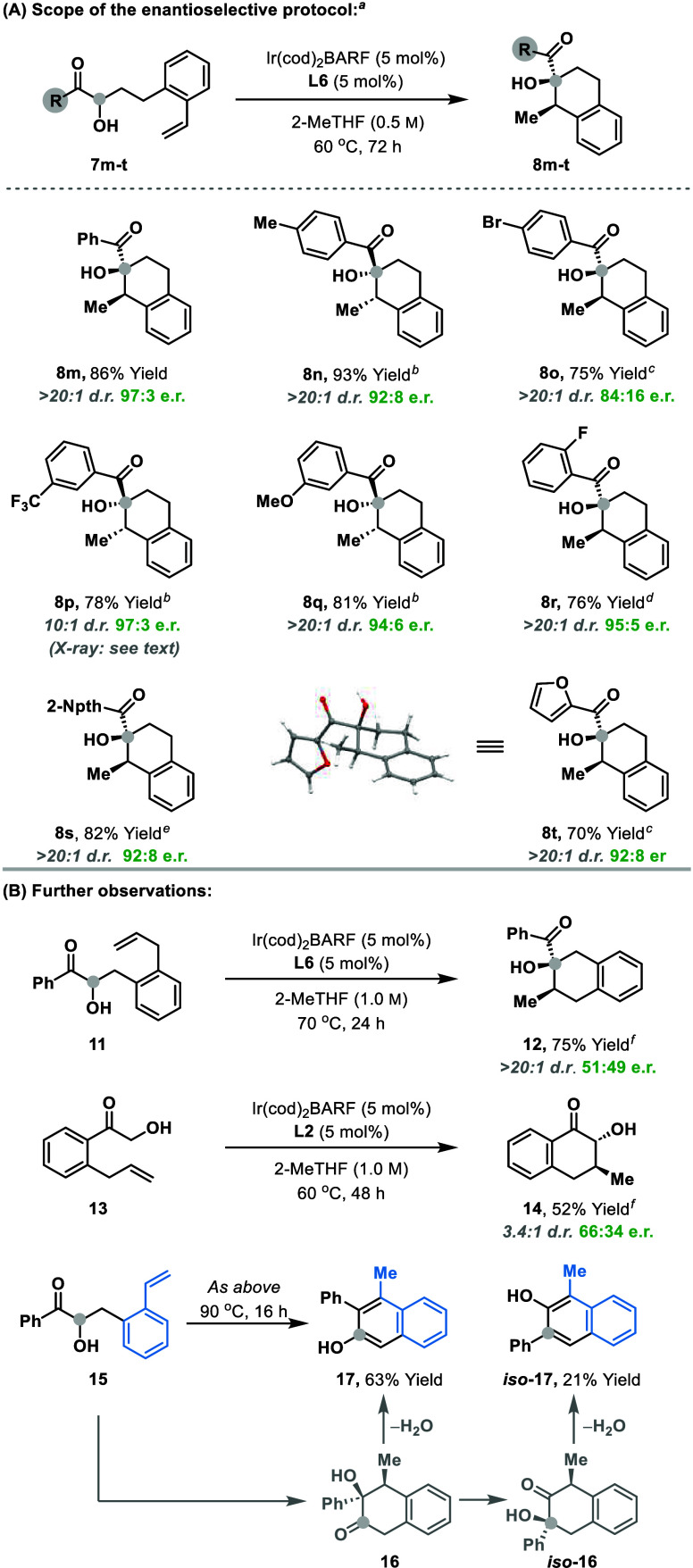
Enantioselective Processes^a^ ^,^ ^b^ ^,^ ^c^ ^,^ ^d^ ^,^ ^e^ ^,^ ^f^

a Full optimization details are given in the SI.

b (*S*)-SynPhos was used as ligand.

c 70 °C for 72 h (0.5 M) using (*R*)-DM-SynPhos.

d 70 °C for 48 h (0.5 M) using (*R*)-Tol-SynPhos.

e Using (*R*)-Tol-SynPhos.

f The absolute stereochemistry of the major enantiomer has not been assigned.

Control experiments have shown that the presence of the ketone and α-hydroxy unit are both critical for C–C bond formation, *and that the position of these units can be switched* (Scheme 3C, eq 7). Such a situation is consistent with the proposed directed enolization process to generate **Int-I** (Scheme 3A). From here, addition of the Ir-enediolate across the alkene is *exo*-selective, as might be expected based on geometric factors. For systems **5**, where n = 1, the process favors 6-*exo* rather 5-*exo* cyclization to deliver **Int-IIa**, whereas for **7**, where n = 2, the expected 6-*exo* cyclization mode predominates to give **Int-IIb**. The feasibility of the addition an Ir-enolate to a carbon-based π-unsaturate is supported by stoichiometric studies on the exposure of Ir­(acac) complexes to alkynes.^15^ For both **Int-IIa** and **Int-IIb**, the formation of 5,6-iridabicycle should strongly favor the formation of the *cis*-fused product, and this accounts for the very high diastereoselectivities observed in Tables 2 and 3.^16^ From **Int-IIa/b**, C–H or O–H reductive elimination and protodemetalation provides the products **6** and **8**, with the former being susceptible to α-ketol rearrangement to provide *iso* **-6**. As alluded to earlier, this process requires the Ir-catalyst, and so rearrangement may occur via the O–H oxidative addition derived Ir-alkoxide of **6** (not depicted). Because α-ketol rearrangements are stereospecific, the relative stereochemistry of **6** is transferred to the rearranged product *iso* **-6**, which is consistent with the experimental observations.^13^ The SI outlines further mechanistic experiments including deuterium labeling, deuterium exchange and resubjection experiments. It should be noted that these experiments do not discount an alternate pathway involving transfer hydrogenative oxidation of the α-hydroxy ketone to a 1,2-dicarbonyl, in advance of an oxidative cyclocoupling-transfer hydrogenation sequence (**Int-1′** to **Int-IIb′** to **8**, Scheme 3B). In the first cycle, the oxidation of **7** to **Int-I′** requires an oxidant, which could be an alkene or carbonyl, to turnover the “IrH_2_” species. We do not have any experimental observations that directly support the mechanism in Scheme 3B, although it is important to note that we cannot fully account for the mass balance of the processes described here. Oxidative cyclocouplings with minimally activated alkenes have been reported under Ru-catalyzed carbonylative conditions by Murai, Chatani and co-workers,^17^ and under Ru- and Os-catalyzed transfer hydrogenation conditions by Krische and co-workers.^18^ To the best of our knowledge, transfer hydrogenative oxidative cyclocouplings using cationic Ir-systems have not been reported with alkyl alkenes or styrenes, whereas alkylations of 1,3-dicarbonyls have been described by Takeuchi and co-workers.^19^ In these mechanistically uncertain processes, high branched selectivity is often observed, but minor variations in reaction conditions or substrate structure can lead to linear products, mirroring the results in Scheme 2.^20^ We note that **Int-I** and **Int-I′** are similar, differing in the oxidation state of the Ir-center and the presence/absence of a hydride ligand, which highlights the subtlety in distinguishing the two mechanistic options. Circumstantial support for the mechanism in Scheme 3A is provided by eqs 8 and 9 in Scheme 3C. The former shows that nonoxidizable O-directing groups can be used, albeit with competitive *ortho*-alkylation of the phenyl ketone.^21^ The latter shows that 1,2-diketones are not viable substrates in the presence of an exogenous alcohol reductant (*i*-PrOH), which disfavors the pathway in Scheme 3B.

**Scheme 3:**
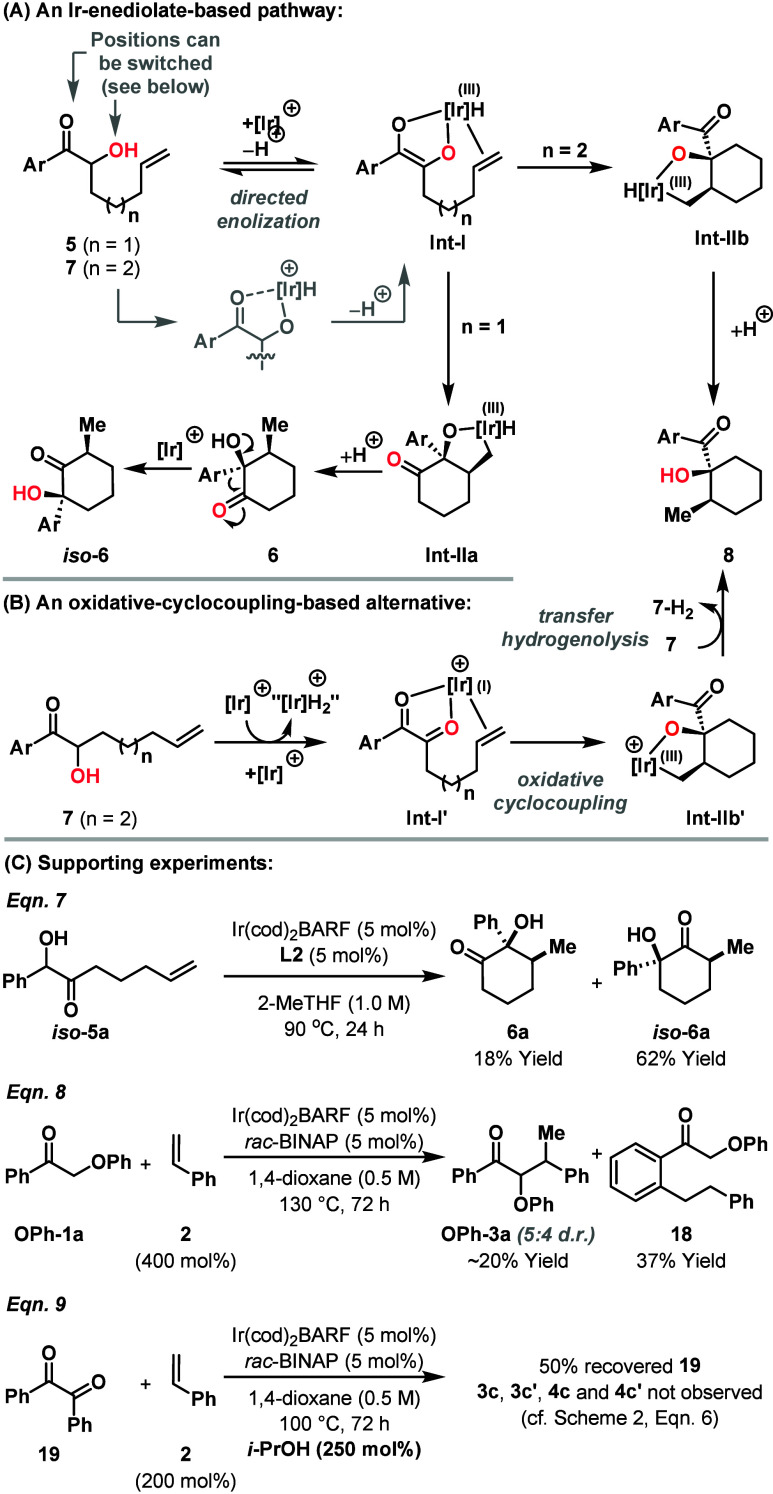
Mechanistic Considerations

In summary, we outline Ir-catalyzed inter- and intramolecular hydroalkylative C­(sp^3^)-C­(sp^3^) cross-couplings that involve the α-C-H addition of α-hydroxy ketones to styrenes or alkyl olefins. The intramolecular processes are generally highly regioselective and completely diastereoselective, with certain systems allowing high enantioselectivities. The processes are predicated on the directed generation of an Ir-enediolate in advance of branched selective addition of this to an alkene. The use of hydroxyl groups to direct Ir-enediolate formation represents a significant advance over our previous work, which required aniline-based directing groups (Scheme 1B, eq 2).^2^ In broader synthetic terms, the current method offers rare examples of alkene-based Conia-ene reactions that are both enantio- and diastereoselective,^11,12^ and, in so doing, highlights how Ir-enolates can be leveraged for the stereocontrolled assembly of complex carbocyclic systems.

## Supplementary Material



## Notes

Experimental details, optimization results, limitations, characterization data and crystallographic data. (PDF)
