# Supplementary material for: Iridium-Catalyzed Stereoselective α‑Alkylation of α‑Hydroxy Ketones with Minimally Polarized Alkenes
Source: J Am Chem Soc. 2026 Jan 20;148(4):3911–7. doi: 10.1021/jacs.5c19724 (PMC12879937; doi:10.1021/jacs.5c19724)
Supplement: Supplementary file 1 [file ja5c19724_si_001.pdf]

Iridium-Catalyzed Stereoselective α-Alkylation of α-Hydroxy Ketones with Minimally Polarized Alkenes

Yihong Wang, Fenglin Hong, Craig M. Robertson, Richard J. Mudd and John F. Bower*

Department of Chemistry, University of Liverpool, Crown Street, Liverpool, L69 7ZD, United Kingdom

john.bower@liverpool.ac.uk

Contents

Contents 2
General Information 3
Experimental procedures and data 4
General procedures 4
Optimization of an intermolecular alkene hydroalkylation (Table S1) 9
Intermolecular hydroalkylation products 10
Characterization of substrates 15
Characterization of products 45
Development of an enantioselective protocol (Table S2) 61
Limitations 78
Derivatizations 78
Mechanistic studies 86
Control experiments 86
C-Methylation of **5a** with sodium hydride and methyl iodide 88
Deuterium labelling and exchange experiments 90
References 93
NMR spectra 94
Intermolecular hydroalkylation products 95
Substrates 102
Products of Catalysis 165
Derivatizations 211
Mechanistic studies 218

General Information

Reagents, Solvents and Reactions. Starting materials were purchased from commercial sources (Acros, Aldrich, Alfa Aesar, Fluorochem, TCI, Apollo Scientific, BLD Pharm) and used without further purification unless otherwise stated. Anhydrous solvents were obtained by passage through drying columns supplied by Anhydrous Engineering Ltd. The removal of solvents in vacuo was achieved using both a Büchi rotary evaporator (bath temperatures up to 40 °C) at a pressure of either 15 mmHg (diaphragm pump) or 0.1 mmHg (oil pump), as appropriate, and a high vacuum line at room temperature. Reactions requiring anhydrous conditions were run under a dry atmosphere of nitrogen or argon; glassware was either flame dried immediately prior to use or placed in an oven (200 °C) for at least 2 h and allowed to cool either in a desiccator or under an atmosphere of nitrogen or argon; liquid reagents, solutions or solvents were added via syringe through rubber septa.

Flash column chromatography (FCC) was performed using Sigma-Aldrich silica gel (60 Å, 230-400 mesh, 40-63 μm). Thin-layer chromatography was performed using aluminium backed 60F254 silica plates. Visualisation was achieved by UV fluorescence or a basic $KMnO_4$ solution and heat.

Characterization data. NMR spectra were recorded on Bruker Nano 400 and Bruker Avance III HD 500 Cryo spectrometers. Chemical shifts (δ) are given in parts per million (ppm) and referenced to the appropriate residual solvent peak. Peaks are described as singlets (s), doublets (d), triplets (t), quartets (q), pentets (pent), sextets (sext), heptets (hept), multiplets (m) and broad (br). Coupling constants (J) are quoted to the nearest 0.5 Hz. Assignments of ^{1}H NMR and ^{13}C NMR signals were made, where possible, using COSY, HSQC, HMBC, and NOE experiments. Numbering systems for NMR signal assignments are specified on the structure and are not related to those used for the compound names. Infra-red (IR) spectra were recorded on a Perkin Elmer Spectrum Two FTIR spectrometer as either neat films or solids compressed on a diamond plate. Only selected absorption maxima (νmax) are reported in wavenumbers (cm^{-1}). High resolution mass spectra (HRMS) were recorded on a VG Analytical Autospec spectrometer by Chemical Ionisation (CI) and a Bruker micrOTOF instrument or a 6200 series TOF/6500 series QTOF instrument by Electrospray Ionisation (ESI). Melting points were determined using a Stuart SMP30 melting point apparatus and temperature controller and are uncorrected. Optical rotations ([α]D T) were measured using an ADP440+ polarimeter at the concentration and temperature stated. Enantiomeric excesses were determined using an Agilent 1290 Infinity chiral SFC as stated for each compound.

Naming of Compounds. Compound names were generated by ChemDraw 16.0 software (PerkinElmer), following IUPAC nomenclature.

Experimental procedures and data

General procedures

General procedure A: Preparation of alkenyl ketones[1]

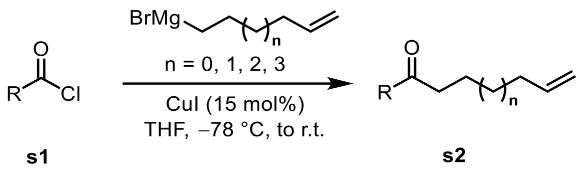

To a dry flask charged with CuI (0.15 equiv.) was added acid chloride **s1** (1.0 equiv.) in THF (4 mL/mmol). The reaction mixture was stirred at r.t. for 10 minutes. and the corresponding freshly prepared Grignard reagent in THF (1 M, 1.5 equiv.) was added dropwise at −78 °C. [Grignard preparation: a dry Schlenk tube equipped with a stirring bar was charged with Mg turnings (1.1 equiv.) and then the flask was evacuated and refilled with N_2 (× 3). The minimum amount of THF was added and then bromide (0.3 equiv.) was added neat. Once the Grignard formation started the remaining bromide (0.7 equiv.) was added as a solution in THF (0.7 M)]. The corresponding mixture was stirred under reflux for an additional 1 h].[2] The reaction mixture warmed to r.t. and was stirred overnight. The reaction mixture was cooled in an ice-bath and was slowly quenched with saturated aq. NH_4Cl (5 mL/mmol). The aqueous phase was extracted with Et_2O (3 × 5 mL/mmol) and the combined organic phases were dried ($MgSO_4$) and concentrated *in vacuo*. The product **s2** was eluted through a plug of silica (95:5 hexane/Et_2O) and used directly in the next step.

General procedure B: Preparation of α-hydroxyketones[3,4]

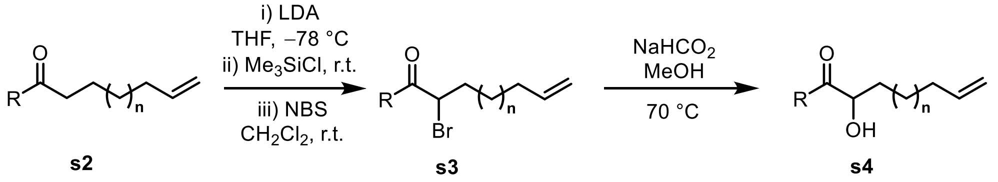

Ketone **s2** (1.0 equiv.) was added to a solution that contained LDA (1.5 equiv.) in THF (1 mL/mmol) at −78 °C. The reaction mixture was stirred at −78 °C for 15 min, and then Me_3SiCl (2.0 equiv.) was added. The mixture was allowed to warm to r.t., and then it was stirred for 2 h. Next, the volatiles were removed, and CH_2Cl_2 (2 mL/mmol) and then *N*-bromosuccinimide (1.2 equiv.; added portionwise) were added. The reaction mixture was stirred for 16 h, and then the reaction was quenched with H_2O (2 mL/mmol). The aqueous layer was extracted with CH_2Cl_2 (2 mL/mmol × 3). The organic layers were combined, dried ($MgSO_4$) and concentrated *in vacuo*. The product **s3** was eluted through a plug of silica (95:5 hexane/Et_2O), and due to its highly reactive nature, was used directly in the next step. To a solution of 2-bromoketone **s3** (1.0 equiv.) in MeOH (1.2 mL/mmol) was added sodium formate (4.00 equiv.). The mixture was stirred at 70 °C for 12 h. The reaction was cooled to room temperature and concentrated *in vacuo*. The residue was diluted with EtOAc (1 mL/mmol) and washed with H_2O (1 mL/mmol). The organic phase was extracted with EtOAc (3 × 1 mL/mmol) and the combined were washed with brine (0.6 mL/mmol), dried ($MgSO_4$) and concentrated *in vacuo*. The residue was purified by FCC (5:1 hexane/EtOAc) to afford the product **s4**.

Caution: *Hydroxyketones need to be freshly prepared and submitted to the cyclization conditions as soon as possible to ensure high efficiencies; long term storage (>1 week) will lead to reduced yields for the hydroalkylation product.*

General procedure C: Preparation of ketones via Weinreb amides[1]

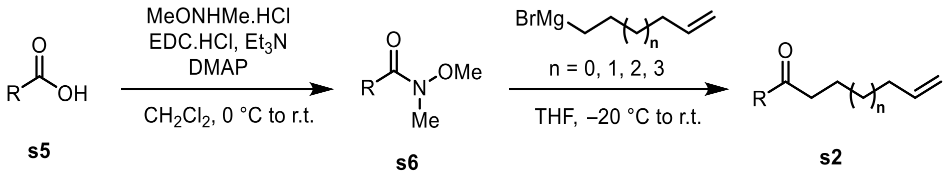

Step 1: To a mixture of carboxylic acid **s5** (1.0 equiv.), DMAP (0.1 equiv.) and *N*,*O*-dimethylhydroxylamine hydrochloride (1.3 equiv.) in CH_2Cl_2 (10 mL/mmol) at 0 °C were added EDC·HCl (1.3 equiv.) and Et_3N (1.33 equiv.). The reaction mixture was stirred at 0 °C for 1 h, and at r.t. overnight. The reaction mixture was diluted with CH_2Cl_2 (10 mL/mmol) and the organic layer was washed with 1 M aq. HCl (3 × 1 mL/mmol), saturated aq. $NaHCO_3$ and brine (10 mL/mmol). The combined organic phases were dried ($MgSO_4$) and concentrated *in vacuo*. The crude product was eluted through a plug of silica (eluent 5/2 hexane/EtOAc) to afford **s6**. *Step 2:* Under an argon atmosphere, the appropriate Grignard reagent (1.2 equiv., freshly prepared in THF)[2] was added dropwise at −20 °C to a solution of **s6** (1.0 equiv.) in THF (10 mL/mmol). The reaction mixture was gradually warmed to r.t. and was stirred for 12 h. The mixture was quenched with saturated aq. NH_4Cl (20 mL) at 0 °C. The aqueous phase was extracted with Et_2O (3 × 20 mL/mmol), and the combined organic phases were dried ($MgSO_4$) and concentrated *in vacuo*. The crude product was eluted through a plug of silica (95:5 hexane/Et_2O) to afford **s2**.

General procedure D: α-Hydroxylation of ketones with iodine[5]

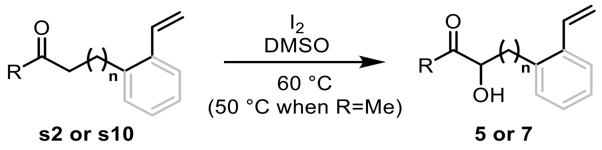

Following the literature procedure, **s2** or **s10** (1.0 equiv.) was added to iodine (20 mol%) and DMSO (1 mL/mmol) under air. The mixture was stirred at 60 °C for 24 h. After cooling to r.t., the solution was diluted with EtOAc (20 mL/mmol) and washed with 0.1 M aq. $Na_2S_2O_3$ (10 mL/mmol). The aqueous extract was back extracted with EtOAc (3× 10 mL/mmol), and the combined organic extracts were concentrated *in vacuo*. The crude reaction mixture was purified by FCC (5:1 hexane/EtOAc) to afford hydroxy ketones **5** or **7**.

Caution: *Hydroxyketones need to be freshly prepared and submitted to the cyclizations conditions as soon as possible to ensure high efficiencies and enantioselectivities; long term storage (>1 week) will lead to reduced yields and enantioselectivities for the hydroalkylation product.*

General procedure E: Preparation of styrenyl ketones

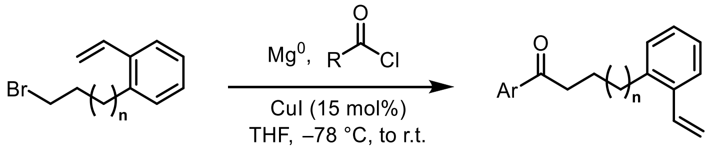

To a dry flask charged with CuI (0.15 equiv.) was added benzoyl chloride (1.0 equiv.) in THF (4 mL/mmol). The reaction mixture was stirred at r.t. for 10 minutes and the corresponding freshly prepared Grignard reagent in THF (1 M, 1.2 equiv.) was added dropwise at −78 °C. [Grignard preparation: a dry Schlenk tube equipped with a stirring bar was charged with Mg turnings (1.1 equiv.) and then the flask was evacuated and refilled with N_2 (× 3). The minimum amount of THF was added and then bromide (0.3 equiv.) was added neat. Once the Grignard formation started the remaining bromide (0.7 equiv.) was added as a solution in THF (0.7 M). The corresponding mixture was stirred under reflux for an additional 1 h]. The reaction mixture was warmed to r.t. and was stirred overnight. The reaction mixture was cooled in an ice-bath and was slowly quenched with saturated aq. NH_4Cl (5 mL/mmol). The aqueous phase was extracted with Et_2O (3 × 5 mL/mmol), and the combined organic phases were dried ($MgSO_4$) and concentrated *in vacuo*. The product **s10** was eluted through a plug of silica (20:1 petrol/Et_2O) and used directly in the next step.

General procedure F: Preparation of β-ether ketones[6,7]

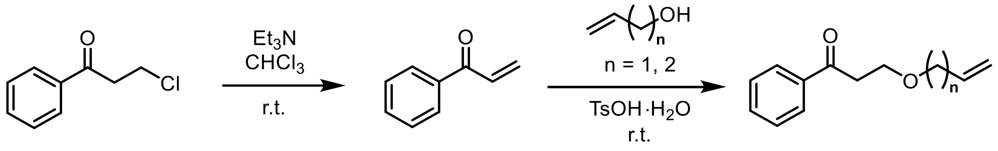

To a stirred solution of 3-chloropropiophenone **s22** (1.0 equiv) in chloroform (2 mL/mmol) was added dropwise Et_3N (2.4 equiv) over 5 minutes under an atmosphere of argon. The reaction mixture was stirred for 18 h and was then washed with 0.1 M aq. HCl (2 × 40 mL), H_2O (2 × 40 mL), saturated aq. $NaHCO_3$ (2 × 2 mL/mmol), and brine (2 mL/mmol). The organic layer was dried (Na_2SO_4) and concentrated *in vacuo*. The residue was purified by FCC (50:1 petrol/Et_2O) and **s23** was used directly in the next step. 1-Phenylprop-2-en-1-one **s23** (1.0 equiv) was dissolved in CH_2Cl_2 (5 mL/mmol) and *p*-TsOH monohydrate (0.05 equiv) was added at r.t., followed by the dropwise addition of the corresponding alkenyl alcohol (1.1 equiv). The reaction was stirred at r.t. for 18 h. Saturated aq. $NaHCO_3$ (5 mL/mmol) was added and the biphasic mixture was extracted with CH_2Cl_2 (2 × 5 mL/mmol). The combined organics were dried ($MgSO_4$) and concentrated *in vacuo*. The residue was purified by FCC (20:1 to 5:1 petrol/Et_2O) to give corresponding product **s24**.

General procedure G: Preparation of hydroxy ketones from Weinreb amides[8,9]

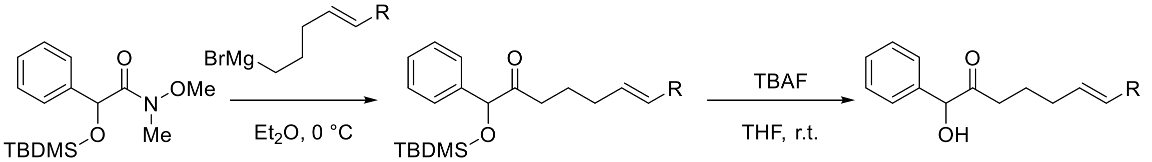

s27 s28 s29 or s30

To a 100 mL, three-necked, round-bottomed flask was added crude α-*tert*-butyldimethylsiloxy Weinreb type amide **s27** (1 equiv.) in anhydrous Et_2O (10 mL/mmol). Freshly prepared Grignard reagent (5 equiv.) in Et_2O (approx. 1.0 M) was added dropwise at 0 °C under a N_2 atmosphere. [Grignard preparation: a dry Schlenk tube equipped with a stirring bar was charged with Mg turnings (1.1 equiv.) and then the flask was evacuated and refilled with N_2 (× 3). The minimum amount of Et_2O was added and then bromide (0.3 equiv.) was added neat. Once the Grignard formation started the remaining bromide (0.7 equiv.) was added as a solution in Et_2O (0.7 M)]. The resulting reaction mixture was stirred for 5 h and then quenched with saturated aq. NH_4Cl (10 mL/mmol). The resulting mixture was extracted with EtOAc (3 × 10 mL/mmol). The combined organic extracts were dried ($MgSO_4$) and concentrated *in vacuo* to give the crude ketone **s28**, which was used directly to the next step. To a solution of ketone **s28** (1 equiv.) in anhydrous THF (12 mL/mmol) was added tetrabutylammonium fluoride (1.0 M in THF, 3 equiv.) at 0 °C under an argon atmosphere. After stirring for 30 minutes at r.t. the reaction was quenched with saturated aq. NH_4Cl (10 mL/mmol) and extracted with Et_2O (2 × 10 mL/mmol). The organic extracts were washed with brine (10 mL/mmol), dried (Na_2SO_4) and concentrated *in vacuo*. The residue was purified by FCC (20:1 to 4:1 petrol/EtOAc) to give ***iso*-5** or **s30**.

General procedure H: Intermolecular iridium-catalyzed alkene hydroalkylation

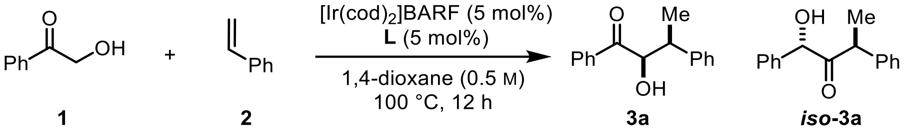

1 2 3a *iso*-3a

A Schlenk tube was charged with α-hydroxyketone (0.20 mmol, 100 mol%), $[Ir(cod)_2]BARF$ (0.01 mmol, 5 mol%) and *rac*-BINAP (0.01 mmol, 5 mol%). The tube was evacuated/backfilled with N_2 (three cycles), then styrene (200 mol%) was added followed by 1,4-dioxane (0.5 M). The tube was sealed and heated at 100 °C for 12 h. After cooling to r.t., the mixture was concentrated *in vacuo*, analyzed by ^1H NMR spectroscopy and the crude reaction mixture was purified by FCC (100% hexane to 4:1 hexane/Et_2O).

General procedure I: Intramolecular iridium-catalyzed alkene hydroalkylation

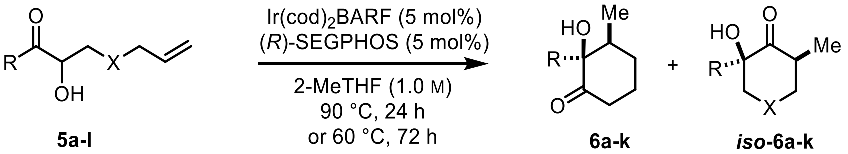

5a-l 6a-k *iso*-6a-k

A Schlenk tube was charged with $[Ir(cod)_2]BARF$ (6.36 mg, 0.005 mmol, 5 mol%) and (*R*)-SEGPHOS (3.05 mg, 0.005 mmol, 5 mol%). The tube was evacuated/backfilled with N_2 (three cycles), then α-ketol substrate (0.1 mmol, 1.0 equiv.) was added, followed by 2-MeTHF (0.1 mL, 1.0 M). The tube was sealed and heated at 90 °C for 24 h,

or 60 °C for 72 h (unless otherwise stated). After cooling to r.t., the mixture was concentrated *in vacuo* and the residue was analyzed by ^{1}H NMR spectrscopy before purification by FCC (4:1 hexane/Et_2O).

General procedure J: Enantioselective iridium-catalyzed alkene hydroalkylation

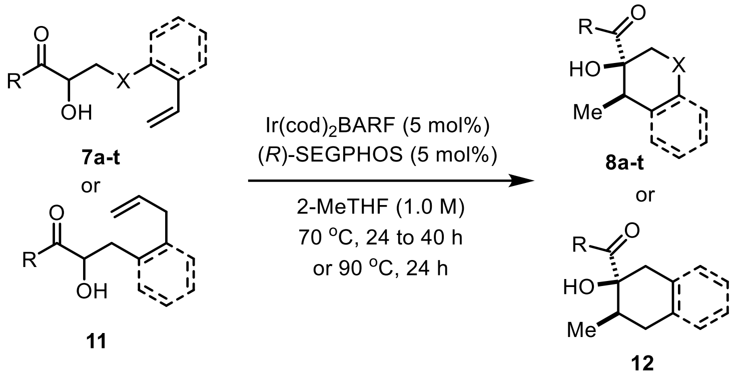

A Schlenk tube was charged with [Ir(cod)$_2$]BARF (6.36 mg, 0.005 mmol, 5 mol%) and (*R*)-SEGPHOS (3.05 mg, 0.005 mmol, 5 mol%). The tube was evacuated/backfilled with N_2 (three cycles), then α-ketol substrate (0.1 mmol, 1.0 equiv.) was added, followed by 2-MeTHF (0.1 mL, 1.0 M or 0.2 mL, 0.5 M for **8m-t**). The tube was sealed and heated at the stated temperature for the stated time. After cooling to r.t., the mixture was concentrated *in vacuo* and the residue was analyzed by ^{1}H NMR spectroscopy before purification by FCC (5:1 hexane/Et_2O).

General procedure K: Synthesis of 1,2-diols[10]

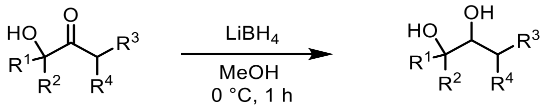

$LiBH_4$ (7.2 mg, 0.33 mmol, 1.1 equiv) was added to an oven-dried 4 mL vial equipped with a stirrer bar. The vial was purged with argon and a solution of ketone (0.30 mmol, 1.0 equiv.) in MeOH (1.5 mL) was added dropwise at 0°C. The solution was stirred at 0 °C for 1 h. The mixture was quenched by the addition of H_2O (3 mL), and then extracted with CH_2Cl_2 (3 × 5 mL). The combined organic extracts were dried ($MgSO_4$) and concentrated *in vacuo*. The residue was purified by FCC (2:1 hexane/Et_2O) to obtain the 1,2-diol.

Optimization of an intermolecular alkene hydroalkylation (Table S1)

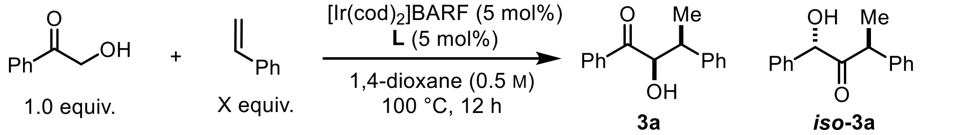

entry	ligand	X	yield[a]	d.r. (3a)[b]	d.r. (*iso*-3a)[b]	3a:*iso*-3a[b]	e.r. (3a major/3a minor/ *iso*-3a major)[c]
1	**L1**	4	50%	9:7	2:1	4:3	/
2[d]	**L1**	4	25%	8:7	2:1	5:3	/
3[e]	**L1**	4	/	/	/	/	/
4	**L1**	1.5	37%	12:11	2:1	3:2	47:53/50:50/47:53
5	**L2**	1.5	55%	6:5	2:1	3:2	67:33/66:34/65:35
6	**L3**	1.5	27%	6:5	2:1	3:2	65:35/66:34/65:35
7[f]	**L4**	1.5	50%	5:3	5:2	8:7	51:49/57:43/50:50
8	**L9**	1.5	41%	6:5	2:1	3:2	59:41/61:39/59:41
9[f]	**L10**	1.5	40%	3:2	2:1	5:3.6	51:49/51:49/51:49
10	**L11**	1.5	40%	5:4	2:1	3:2	54:46/54:46/54:46
11[f]	**L12**	1.5	8%	7:6	/	/	/
**12**[g]	**L2**	1.5	43%	7:6	2.2:1	1.6:1	/
**13**[h]	**L2**	1.5	45%	7:6	2:1	1.6:1	/
**14**[i]	**L2**	1.5	/	3:2	2:1	3:2	/
**15**[j]	**L2**	1.5	/	/	/	/	/

[a] NMR yield. [b] Determined by ^{1}H NMR analysis of the crude reaction mixture. [c] Determined by chiral SFC analysis. [d] 80 °C, 24 h. [e] 50 °C, 24 h; [f] 20 h. [g] $[Ir(cod)_2]OTf$ as precatalyst; [h] $[Ir(cod)_2]BF_4$ as precatalyst; [i] *t*-BuOH as solvent. [j] *o*-DCB as solvent.

Unsuccessful systems:

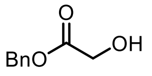

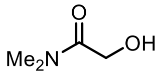

Ligand structures:

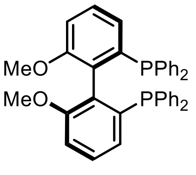

L9: (*R*)-MeO-BIPHEP

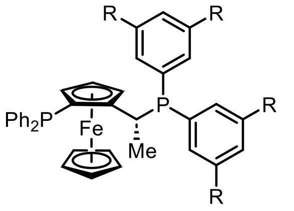

R = Me, L10: (*R*)-JOSIPHOS
R = CF$_3$, L11: (*R*)-WALPHOS

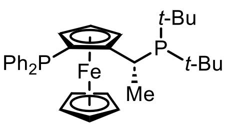

L12: (*R*)-JOSIPHOS-2

L1-8 are as reported in the manuscript.

Intermolecular hydroalkylation products

Eqn. 4

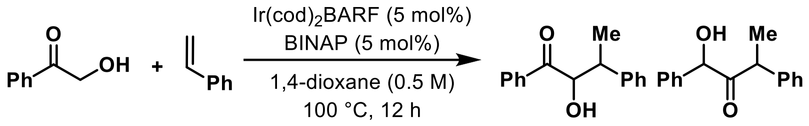

1a **2** (200 mol%) **3a** ***iso*-3a**

Conducted according to *General Procedure H.* **1a** (27.3 mg, 0.20 mmol, 100 mol%), [Ir(cod)$_2$]BARF (12.72 mg, 0.01 mmol, 5 mol%), *rac*-BINAP (6.23 mg, 0.01 mmol, 5 mol%), styrene (41.66 mg, 0.40 mmol, 200 mol%) and 1,4-dioxane (0.40 mL, 0.5 M) were used. The tube was sealed and heated at 100 °C for 12 h. The crude was analyzed by ^{1}H NMR spectroscopy and purified by FCC (100% hexane to 4:1 hexane/Et$_2$O). The major products were a 3:2 mixture of **3a**-*syn* and ***iso*-3a**-*anti* (15 mg, 30%), and the minor product was **3a**-*anti* (8 mg, 16%); these were isolated as colorless oils and data are given below.

syn-2-Hydroxy-1,3-diphenylbutan-1-one 3a (major) and *anti*-1-Hydroxy-1,3-diphenylbutan-2-one *iso*-3a

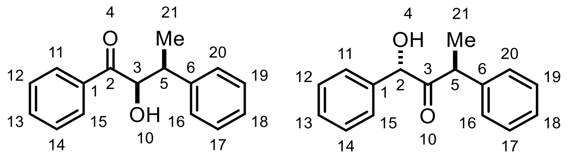

3a-*syn* ***iso*-3a**-*anti*

IR (film) ν_{max}/cm^{-1}: 3472, 3027, 1675, 1257, 1117, 973.

^{1}H NMR (500 MHz, CDCl$_3$) δ 7.99 – 7.97 (m, 2H, **3a**C(11, 15)*H*), 7.67 (t, *J* = 7.4 Hz, **3a**C13*H*), 7.55 (t, *J* = 7.8 Hz, **3a**C(12, 14)*H*), 7.44 – 7.18 (m, 12H, Ar*H*), 5.30 (dd, *J* = 5.8 Hz, 2.6 Hz, 1H, **3a**C3*H*), 5.10 (d, *J* = 3.1 Hz, 0.7H, ***iso*-3a**C2*H*), 4.28 (d, *J* = 4.7 Hz, 0.7H, ***iso*-3a**O*H*), 3.82 (d, *J* = 5.7 Hz, 1H, **3a**O*H*), 3.79 (q, *J* = 6.9 Hz, 0.7H, ***iso*-3a**C5*H*), 3.27 (qd, *J* = 7.1 Hz, 2.3 Hz, 1H, **3a**C5*H*), 1.30 (d, *J* = 7.0 Hz, 2.2H, ***iso*-3a**C21*H$_3$*), 1.30 (d, *J* = 7.1 Hz, 3H, **3a**C21*H$_3$*).

^{13}C NMR (126 MHz, CDCl$_3$) δ 209.7 (***iso*-3a**C3), 201.5 (**3a**C2), 143.9 (**3a**C6), 139.7 (***iso*-3a**C6), 138.2 (***iso*-3a**C1), 134.0 (**3a**C13), 133.9 (**3a**C1), 129.3 (***iso*-3a**ArC), 129.1 (***iso*-3a**ArC), 129.0 (**3a**ArC), 128.8 (***iso*-3a**ArC), 128.6 (**3a**ArC), 128.6 (**3a**ArC), 128.0 (***iso*-3a**ArC), 127.8 (**3a**ArC), 127.8 (***iso*-3a**ArC), 127.6 (***iso*-3a**ArC), 126.8 (**3a**ArC), 77.8 (***iso*-3a**C2), 77.3 (**3a**C3), 47.7 (***iso*-3a**C5), 43.4 (**3a**C5), 17.9 (***iso*-3a**C21), 13.4 (**3a**C21).

HRMS (ESI): calculated for $C_{16}H_{16}O_2Na$ [M+Na]$^+$ requires m/z 263.1048, found m/z 263.1043.

The spectroscopic properties were consistent with the data available in the literature.[11]

anti-2-Hydroxy-1,3-diphenylbutan-1-one 3a (minor)

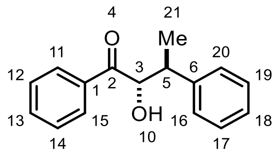

3a-*anti*

IR (film) ν_{max}/cm^{-1}: 3471, 3026, 1677, 1271, 1117, 976.

^{1}H NMR (500 MHz, $CDCl_3$) δ 7.84 – 7.83 (m, 2H, C(11, 15)*H*), 7.66 (t, *J* = 7.4 Hz, C13*H*), 7.53 (t, *J* = 7.8 Hz, C(12, 14)*H*), 7.20 – 7.17 (m, 3H, Ar*H*), 6.95 – 6.92 (m, 2H, Ar*H*), 5.26 (dd, *J* = 7.0 Hz, 2.9 Hz, 1H, C3*H*), 3.55 (d, *J* = 7.0 Hz, 1H, O*H*), 3.25 (qd, *J* = 7.2 Hz, 2.9 Hz, 1H, C5*H*), 1.58 (d, *J* = 7.2 Hz, 3H, C21*H_3*).

^{13}C NMR (126 MHz, $CDCl_3$) δ 200.9 (C2), 139.7 (C6), 134.6 (C1), 133.8 (C13), 128.9 (ArC), 128.5 (ArC), 128.2 (ArC), 128.0 (ArC), 127.0 (C18), 77.3 (C3), 44.3 (C5), 18.3 (C21).

HRMS (ESI): calculated for $C_{16}H_{16}O_2Na$ $[M+Na]^+$ requires m/z 263.1048, found m/z 263.1049.

Eqn. 5

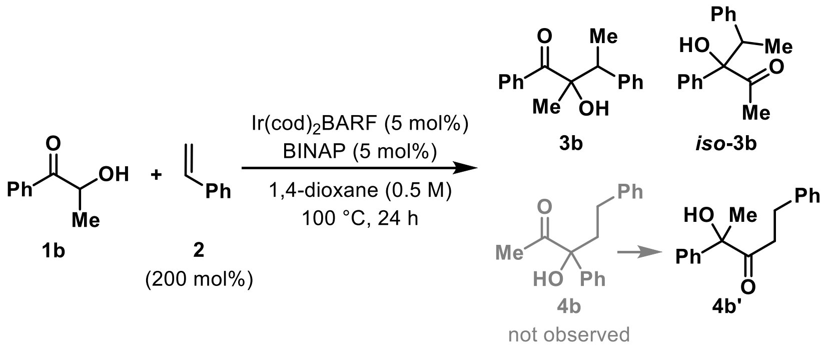

Conducted according to *General Procedure H.* **1b** (30.0 mg, 0.20 mmol, 100 mol%), $[Ir(cod)_2]$BARF (12.72 mg, 0.01 mmol, 5 mol%), *rac*-BINAP (6.23 mg, 0.01 mmol, 5 mol%, styrene (41.66 mg, 0.40 mmol, 200 mol%) and 1,4-dioxane (0.40 mL, 0.5 M) were used. The tube was sealed and heated at 100 °C for 12 h. The crude was analyzed by ^{1}H NMR spectroscopy and purified by FCC (100% hexane to 4:1 hexane/Et_2O). The major isolated products were a 1:6 mixture of **3b** (2:1 d.r.) and ***iso*-3b** (2:1 d.r.) (22 mg, 43%), and the minor isolated product was **4b'** (9 mg, 17%). These were isolated as colorless oils and data are given below.

2-Hydroxy-2-methyl-1,3-diphenylbutan-1-one 3b and 3-Hydroxy-3,4-diphenylpentan-2-one *iso*-3b

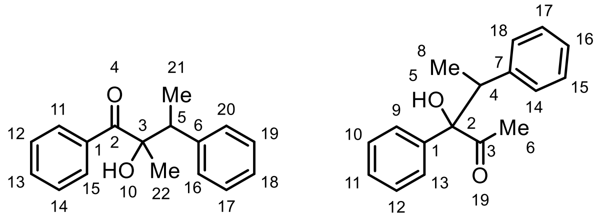

3b ***iso*-3b**

The data for the mixture of four compounds [**3b** (2:1 dr):***iso*-3b** (2:1 dr) = 1:6]:

IR (film) ν_{max}/cm^{-1}: 3460, 3063, 1706, 1492, 1007, 912.

^{1}H NMR (500 MHz, $CDCl_3$) δ 7.92 – 7.90 (m, 0.2H, Ar*H*), 7.58 (t, *J* = 7.4 Hz, 0.1H, Ar*H*), 7.53 (d, *J* = 7.4 Hz, 0.2H, Ar*H*), 7.03 – 7.01 (m, 0.2H, Ar*H*), 6.85 – 6.83 (m, 2H, Ar*H*), 4.70 (s, 0.1H, **3b minor**O*H*), 4.64 (s, 0.5H, ***iso*-3b minor**O*H*), 4.40 (s, 0.2H, **3b major**O*H*), 4.12 (s, 1H, ***iso*-3b major**O*H*), 4.10 (q, *J* = 7.0 Hz, 1H, ***iso*-3b major**C4*H*), 4.01 (q, *J* = 7.0 Hz, 0.7H, ***iso*-3b minor**C4*H*), 3.94 (q, *J* = 7.0 Hz, 0.2H, **3b major**C5*H*), 3.52 (q, *J* = 7.0 Hz, 0.1H, **3b minor**C5*H*), 2.31 (s, 0.5H, **3b major**C22*H_3*), 2.13 (s, 0.2H, **3b minor**C22*H_3*), 1.84 (s, 3H, ***iso*-3b major**C6*H_3*), 1.60 (s, 1.6H, ***iso*-3b major** C6*H_3*), 1.52 (d, *J* = 7.0 Hz, 0.4H, **3b minor**C21*H_3*), 1.41 (d, *J* = 7.0 Hz, 3H, ***iso*-3b major**C8*H_3*), 1.32 (d, *J* = 7.0 Hz, 0.6H, **3b major**C21*H_3*), 1.17 (d, *J* = 7.0 Hz, 3H, ***iso*-3b minor**C8*H_3*).

^{13}C NMR (126 MHz, $CDCl_3$) δ 212.7, 212.4, 209.4, 209.2, 141.7, 141.6, 141.2, 140.5, 140.3, 140.2, 140.1, 140.0, 132.9, 129.7, 129.3, 128.8, 128.8, 128.7, 128.6, 128.5, 128.5, 128.4, 128.3, 128.2, 128.2, 128.0, 128.0, 127.9, 127.7, 127.5, 127.4, 127.3, 126.8, 126.8, 126.6, 126.6, 126.4, 126.3, 126.3, 126.2, 85.8, 81.0, 80.5, 80.5, 47.5, 46.0, 45.7, 44.8, 38.7, 29.7, 25.4, 24.6, 24.1, 23.9, 23.5, 21.0, 20.3, 17.1, 15.4.

HRMS (ESI): calculated for $C_{17}H_{18}O_2Na$ $[M+Na]^+$ requires m/z 277.1204, found m/z 277.1199.

4-Hydroxy-1,4-diphenylpentan-3-one 4b'

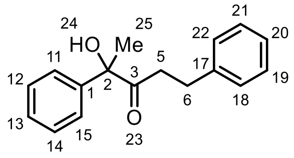

4b'

IR (film) ν_{max}/cm^{-1}: 3461, 3063, 1706, 1495, 1118, 1068.

^{1}H NMR (500 MHz, $CDCl_3$) δ 7.41 – 7.30 (m, 5H, Ar*H*), 7.25 – 7.22 (m, 2H, Ar*H*), 7.19 – 7.17 (m, 1H, Ar*H*), 7.04 – 7.03 (m, 2H, Ar*H*), 4.52 (s, 1H, O*H*), 2.90 – 2.64 (m, 4H, C(5, 6)H_2), 1.73 (s, 3H, C25H_3).

^{13}C NMR (126 MHz, $CDCl_3$) δ 211.0 (C3), 141.2 (C17), 140.4 (C1), 128.7 (ArC), 128.5 (ArC), 128.2 (ArC), 128.1 (ArC), 126.2 (ArC), 126.0 (ArC), 79.8 (C2), 37.5 (C5), 30.1 (C6), 23.9 (C25).

HRMS (ESI): calculated for $C_{17}H_{18}O_2Na$ $[M+Na]^+$ requires m/z 277.1204, found m/z 277.1199.

Eqn. 6

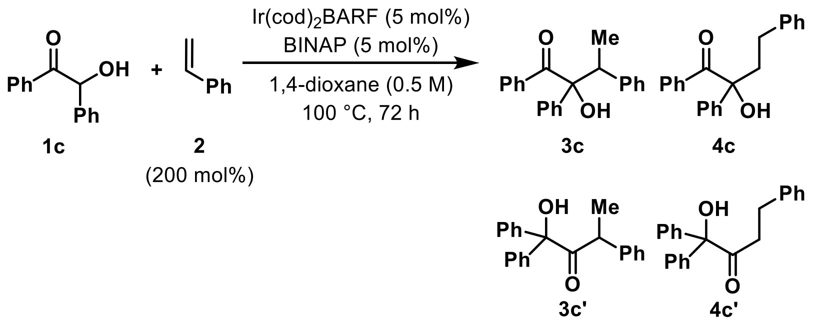

Conducted according to *General Procedure H.* **1c** (42.5 mg, 0.20 mmol, 100 mol%), $[Ir(cod)_2]BARF$ (12.72 mg, 0.01 mmol, 5 mol%), *rac*-BINAP (6.23 mg, 0.01 mmol, 5 mol%), styrene (41.66 mg, 0.40 mmol, 200 mol%) and 1,4-dioxane (0.40 mL, 0.5 M) were used. The tube was sealed and heated at 100 °C for 12 h. The crude was analyzed by ^{1}H NMR spectroscopy and purified by FCC (100% hexane to 4:1 hexane/Et_2O). The major isolated products were a 2:1 mixture of **4c** and **4c'** (34 mg, 54%), and the minor isolated products were a 3:1 mixture of **3c** and **3c'** (12 mg, 21%), and **4c** (6 mg, 9%). These were isolated as colorless oils and data are given below.

2-Hydroxy-1,2,4-triphenylbutan-1-one 4c

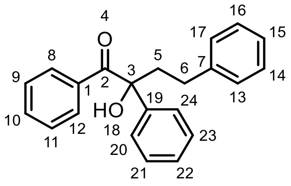

4c

IR (film) ν_{max}/cm^{-1}: 3447, 3063, 1670, 1495, 1115, 1070.

^{1}H NMR (500 MHz, $CDCl_3$) δ 7.71 – 7.69 (m, 2H, C(8, 12)*H*), 7.52 – 7.50 (m, 3H, Ar*H*), 7.43 – 7.40 (m, 2H, Ar*H*), 7.37 – 7.33 (m, 3H, Ar*H*), 7.26 – 7.23 (m, 2H, Ar*H*), 7.19 – 7.16 (m, 1H, Ar*H*), 7.06 – 7.04 (m, 2H, Ar*H*), 4.87 (s, 1H, O*H*), 2.91 – 2.79 (m, 1H, C6H_A), 2.75 – 2.64 (m, 2H, C5H_2), 2.34 (m, 1H, C6H_B).

^{13}C NMR (126 MHz, $CDCl_3$) δ 202.0 (C2), 141.9 (ArC), 141.6 (ArC), 134.1 (ArC), 133.1 (ArC), 129.8 (ArC), 129.0 (ArC), 128.4 (ArC), 128.4 (ArC), 128.4 (ArC), 128.3 (ArC), 126.2 (ArC), 126.0 (ArC), 81.5 (C3), 39.9 (C5), 29.7 (C6).

HRMS (ESI): calculated for $C_{22}H_{20}O_2Na$ $[M+Na]^+$ requires m/z 339.1361, found m/z 339.1357.

2-Hydroxy-1,2,4-triphenylbutan-1-one 4c and 1-Hydroxy-1,1,4-triphenylbutan-2-one 4c'

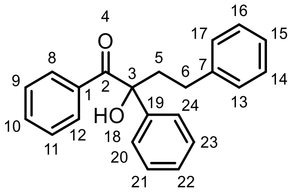

4c

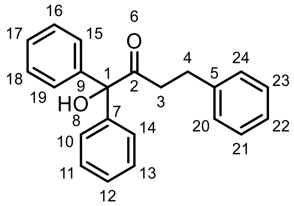

4c'

IR (film) ν_{max}/cm^{-1}: 3447, 3026, 1669, 1447, 1178, 1069.

^{1}H NMR (500 MHz, $CDCl_3$) δ 7.71 – 7.70 (m, 2H, **4c**C(8, 12)*H*), 7.52 – 7.49 (m, 3H, Ar*H*), 7.37 – 7.30 (m, 7H, Ar*H*), 7.27 – 7.16 (m, 4H, Ar*H*), 7.10 – 7.08 (m, 1H, Ar*H*), 7.06 – 7.05 (m, 2H, Ar*H*), 4.88 (s, 1H, **4c**O*H*), 4.84 (s, 1H, **4c'**O*H*), 2.92 – 2.79 (m, 3H, **4c'**C(3, 4)H_2, **4c**C6H_A), 2.75 – 2.64 (m, 2H, **4c**C5H_2), 2.35 (m, 1H **4c**C6H_B).

^{13}C NMR (126 MHz, $CDCl_3$) δ 210.2 (**4c'**C2), 202.0 (**4c**C2), 141.9 (ArC), 141.6 (ArC), 141.4 (ArC), 140.4 (ArC), 134.1 (ArC), 133.1 (ArC), 129.8 (ArC), 129.0 (ArC), 128.5 (ArC), 128.5 (ArC), 128.4 (ArC), 128.4 (ArC), 128.3 (ArC), 128.2 (ArC), 128.1 (ArC), 126.3 (ArC), 126.2 (ArC), 126.0 (ArC), 85.6 (**4c'**C3), 81.5 (**4c**C3), 40.5 (**4c'**C3), 39.9 (**4c**C5), 30.5 (**4c'**C4), 29.7 (**4c**C6).

HRMS (ESI): calculated for $C_{22}H_{20}O_2Na$ $[M+Na]^+$ requires m/z 339.1361, found m/z 339.1359.

2-Hydroxy-1,2,3-triphenylbutan-1-one 3c and 1-Hydroxy-1,1,3-triphenylbutan-2-one 3c'

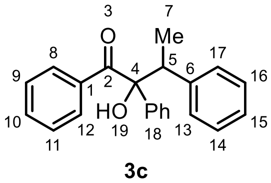

3c

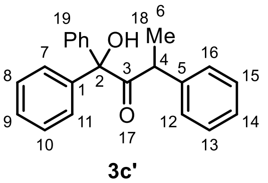

3c'

IR (film) ν_{max}/cm^{-1}: 3441, 3063, 1670, 1494, 1178, 967.

^{1}H NMR (500 MHz, $CDCl_3$) δ 7.76 – 7.74 (m, 0.7H, **3c**C(8, 12)*H*), 7.49 – 7.46 (m, 0.4H, **3c**C10*H*), 7.41 – 7.34 (m, 6H, Ar*H*), 7.25 – 7.07 (m, 12H, Ar*H*), 4.34 (q, J = 7.0 Hz, 1H, **3c'**C4*H*), 4.26 (s, 1H, **3c'**O*H*), 4.01 (q, J = 7.0 Hz, 0.3H, **3c**C5*H*), 3.53 (s, 1H, **3c**O*H*), 1.42 (d, J = 7.0 Hz, 1H, **3c**C7H_3), 1.29 (d, J = 7.0 Hz, 3H, **3c'**C6H_3).

^{13}C NMR (126 MHz, $CDCl_3$) δ 212.0 (**3c'**C3), 202.5 (**3c**C2), 142.0 (ArC), 141.5 (ArC), 140.6 (ArC), 140.4 (ArC), 140.2 (ArC), 136.3 (ArC), 132.3 (ArC), 129.6 (ArC), 129.2 (ArC), 128.4 (ArC), 128.4 (ArC), 128.4 (ArC), 128.2 (ArC), 128.1 (ArC), 128.1 (ArC), 128.1 (ArC), 127.9 (ArC), 127.8 (ArC), 127.5 (ArC), 126.8 (ArC), 126.5 (ArC), 125.9 (ArC), 86.4 (**3c'**C2), 86.0 (**3c**C4), 48.0 (**3c'**C4), 47.3 (**3c**C5), 21.2 (**3c'**C6), 16.8 (**3c**C7).

HRMS (ESI): calculated for $C_{22}H_{20}O_2Na$ $[M+Na]^+$ requires m/z 339.1361, found m/z 339.1355.

Characterization of substrates

2-Hydroxy-1-phenylhept-6-en-1-one 5a

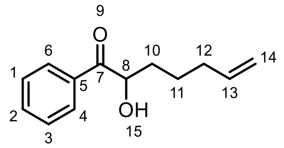

5a

Prepared according to *General Procedure A* and *General Procedure B* via a 3 step sequence. **5a-s1** (704 mg, 5.0 mmol, 1.0 equiv.), CuI (143 mg, 0.75 mmol, 0.15 equiv.), THF (20 mL) and Grignard reagent in THF (7.5 mmol, 7.5 mL, 1.5 equiv.) were used in step 1. **5a-s2** (584 mg, 3.1 mmol, 1.0 equiv.), LDA (4.6 mmol in 4.6 mL THF, 1.5 equiv.), CH_2Cl_2 (6 mL) and NBS (662 mg, 3.72 mmol, 1.2 equiv.) were used in step 2. **5a-s3** (695 mg, 2.6 mmol, 1.0 equiv.), MeOH (3.2 mL) and sodium formate (708 mg, 10.4 mmol, 4.0 equiv.) were used in step 3. This yielded **5a** (399 mg, 39% over 3 steps) as a pale yellow oil.

^{1}H NMR (500 MHz, $CDCl_3$) δ 7.95 – 7.92 (m, 2H, C(4, 6)*H*), 7.66 – 7.63 (m, 1H, C2*H*), 7.54 – 7.51 (m, 2H, C(1, 3)*H*), 5.81 – 5.72 (m, 1H, C13*H*), 5.10 (dd, *J* = 7.7, 3.4 Hz, 1H, C8*H*), 5.02 – 4.98 (dt, *J* = 17.1, 1.8 Hz, 1H, C14H_B), 4.99 – 4.94 (m, 1H, C14H_A), 3.72 (s, 1H, O*H*), 2.15 – 2.03 (m, 2H, C12H_2), 1.94 – 1.87 (m, 1H, C10H_B), 1.70 – 1.49 (m, 3H, C11H_2, C10H_A). The spectroscopic properties were consistent with the data available in the literature.[4]

2-Hydroxy-1-(*p*-tolyl)hept-6-en-1-one 5b

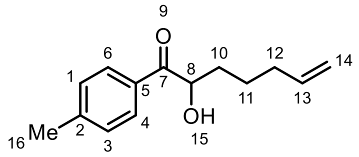

5b

Prepared according to *General Procedure A* and *General Procedure D* via a 2 step sequence. **5b-s1** (619 mg, 4.0 mmol, 1.0 equiv.), CuI (114 mg, 0.6 mmol, 0.15 equiv.), THF (16 mL) and Grignard reagent in THF (6.0 mmol, 6.0 mL, 1.5 equiv) were used in step 1. **5b-s2** (607 mg, 3.0 mmol, 1.0 equiv.), iodine (152 mg, 0.6 mmol, 20 mol%) and DMSO (3.0 mL) were used in step 2. This yielded **5b** (381 mg, 44% over 2 steps) as a pale yellow oil.

IR (film) ν_{max}/cm^{-1}: 3467, 2922, 1674, 1267, 1087, 912.

^{1}H NMR (500 MHz, $CDCl_3$) δ 7.83 (d, *J* = 8.0 Hz, 2H, C(4, 6)*H*), 7.32 (d, *J* = 8.0 Hz, 2H, C(1, 3)*H*), 5.81 – 5.73 (m, 1H, C13*H*), 5.07 (dd, *J* = 7.7, 3.2 Hz, 1H, C8*H*), 5.02 – 4.94 (m, 2H, C14H_2), 3.74 (s, 1H, O*H*), 2.45 (s, 3H, C16*H*), 2.14 – 2.02 (m, 2H, C12H_2), 1.93 – 1.86 (m, 1H, C10H_A), 1.70 – 1.48 (m, 3H, C11H_2, C10H_B).

^{13}C NMR (126 MHz, $CDCl_3$) δ 201.6 (C7), 145.0 (C2), 138.3 (C13), 131.0 (C5), 129.6 (C(1, 3)), 128.7 (C(4, 6)), 114.9 (C14), 72.7 (C8), 35.4 (C10), 33.3 (C12), 24.1 (C11), 21.8 (C16).

HRMS (ESI): calculated for $C_{14}H_{19}O_2$ $[M+H]^+$ requires m/z 219.1385, found m/z 219.1372.

1-(4-Chlorophenyl)-2-hydroxyhept-6-en-1-one 5c

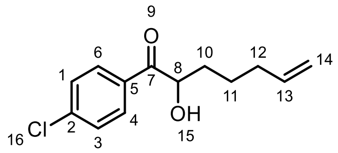

5c

Prepared according to *General Procedure A* and *General Procedure D* via a 2 step sequence. **5c-s1** (700 mg, 4.0 mmol, 1.0 equiv.), CuI (114 mg, 0.6 mmol, 0.15 equiv.), THF (16 mL) and Grignard reagent in THF (6.0 mmol, 6.0 mL, 1.5 equiv.) were used in step 1. **5c-s2** (557 mg, 2.5 mmol, 1.0 equiv.), iodine (127 mg, 0.5 mmol, 20 mol%) and DMSO (2.5 mL) were used in step 2. This yielded **5c** (240 mg, 25% over 2 steps) as a yellow oil.

IR (film) ν_{max}/cm^{-1}: 3474, 2925, 1682, 1589, 1091, 911.

^{1}H NMR (500 MHz, $CDCl_3$) δ 7.88 (d, *J* = 8.5 Hz, 2H, C(4, 6)*H*), 7.32 (d, *J* = 8.5 Hz, 2H, C(1, 3)*H*), 5.80 – 5.72 (m, 1H, C13*H*), 5.07 – 4.95 (m, 3H, C8*H*, C14H_2), 3.74 (d, *J* = 6.4 Hz, 1H, O*H*), 2.15 – 2.03 (m, 2H, C12H_2), 1.91 – 1.84 (m, 1H, C10H_A), 1.70 – 1.48 (m, 3H, C11H_2, C10H_B).

^{13}C NMR (126 MHz, $CDCl_3$) δ 200.9 (C7), 140.5 (C2), 138.1 (C13), 131.9 (C5), 129.6 (C(4, 6)), 129.3 (C(1, 3)), 115.1 (C14), 72.9 (C8), 35.2 (C10), 33.2 (C12), 24.0 (C11).

HRMS (ESI): calculated for $C_{13}H_{16}Cl^{35}O_2$ $[M+H]^+$ requires m/z 239.0839, found m/z 239.0849.

1-(2-Fluorophenyl)-2-hydroxyhept-6-en-1-one 5d

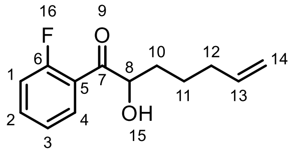

5d

Prepared according to *General Procedure A* and *General Procedure D* via a 2 step sequence. **5d-s1** (625 mg, 4.0 mmol, 1.0 equiv.), CuI (114 mg, 0.6 mmol, 0.15 equiv.), THF (16 mL) and Grignard reagent in THF (6.0 mmol, 6.0 mL, 1.5 equiv.) were used in step 1. **5d-s2** (598 mg, 2.9 mmol, 1.0 equiv.), iodine (152 mg, 0.6 mmol, 20 mol%) and DMSO (2.9 mL) were used in step 2. This yielded **5d** (417 mg, 46% over 2 steps) as a yellow oil.

IR (film) ν_{max}/cm^{-1}: 3485, 2924, 1679, 1454, 1089, 912.

^{1}H NMR (500 MHz, $CDCl_3$) δ 7.96 – 7.93 (m, 1H, C4*H*), 7.64 – 7.59 (m, 1H, C2*H*), 7.33 – 7.31 (m, 1H, C3*H*), 7.21 – 7.17 (m, 1H, C1*H*), 5.81 – 5.73 (m, 1H, C14*H*), 5.01 – 4.93 (m, 3H, C8*H*, C14H_2), 3.70 (s, 1H, O*H*), 2.15 – 2.01 (m, 2H, C12H_2), 1.90 – 1.84 (m, 1H, C10H_A), 1.70 – 1.44 (m, 3H, C10H_B, C11H_2).

^{19}F NMR (470 MHz, $CDCl_3$) δ –108.4.

^{13}C NMR (126 MHz, $CDCl_3$) δ 200.5 (d, *J* = 4.4 Hz, C7), 161.6 (d, *J* = 255.6 Hz, C6), 138.2 (C13), 135.6 (d, J = 9.2 Hz, C2), 131.1 (d, *J* = 2.7 Hz, C4), 124.9 (d, *J* = 3.3 Hz, C3), 122.4 (d, *J* = 13.2 Hz, C5), 116.8 (d, *J* = 23.5 Hz, C1), 114.8 (C14), 76.4 (d, *J* = 9.1 Hz, C8), 34.1 (C10), 33.3 (C12), 24.5 (C11).

HRMS (ESI): calculated for $C_{13}H_{16}FO_2$ $[M+H]^+$ requires m/z 223.1134, found m/z 223.1127.

2-Hydroxy-1-(3-methoxyphenyl)hept-6-en-1-one 5e

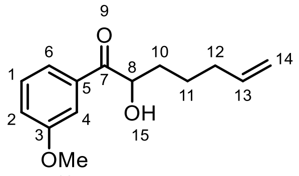

Prepared according to *General Procedure A* and *General Procedure D* via a 2 step sequence. **5e-s1** (853 mg, 5.0 mmol, 1.0 equiv.), CuI (143 mg, 0.75 mmol, 0.15 equiv.), THF (20 mL) and Grignard reagent in THF (7.5 mmol, 7.5 mL, 1.5 equiv.) were used in step 1. **5e-s2** (589 mg, 2.7 mmol, 1.0 equiv.), iodine (137 mg, 0.54 mmol, 20 mol%) and DMSO (2.7 mL) were used in step 2. This yielded **5e** (266 mg, 23% over 2 steps) as a pale yellow oil.

IR (film) ν_{max}/cm^{-1}: 3408, 2942, 1681, 1488, 1045, 912.

^{1}H NMR (500 MHz, $CDCl_3$) δ 7.49 – 7.47 (m, 2H, C(1, 6)*H*), 7.44 – 7.41 (m, 1H, C4*H*), 7.19 – 7.17 (m, 1H, C2*H*), 5.81 – 5.73 (m, 1H, C13*H*), 5.08 – 5.06 (m, 1H, C8*H*), 5.00 (dt, *J* = 17.2, 1.5 Hz, 1H, C14H_A), 4.96 (dt, *J* = 10.2, 1.5 Hz, 1H, C14H_B), 3.89 (s, 3H, C16*H*), 3.69 (d, *J* = 6.5 Hz, 1H, O*H*), 2.15 – 2.03 (m, 2H, C12H_2), 1.94 – 1.87 (m, 1H, C10H_B), 1.70 – 1.49 (m, 3H, C11H_2, C10H_A).

^{13}C NMR (126 MHz, $CDCl_3$) δ 202.0 (C7), 160.0 (C3), 138.2 (C13), 135.0 (C5), 129.9 (C4), 121.0 (C1), 120.3 (C6), 114.9 (C14), 113.0 (C2), 73.0 (C8), 55.5 (C16), 35.3 (C10), 33.3 (C12), 24.1 (C11).

HRMS (ESI): calculated for $C_{14}H_{18}O_3Na$ $[M+Na]^+$ requires m/z 257.1154, found m/z 257.1148.

2-Hydroxy-1-(3-(trifluoromethyl)phenyl)hept-6-en-1-one 5f

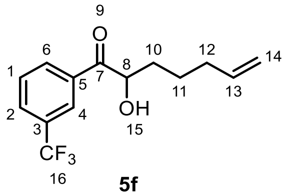

Prepared according to *General Procedure A* and *General Procedure D* via a 2 step sequence. **5f-s1** (835 mg, 4.0 mmol, 1.0 equiv.), CuI (114 mg, 0.6 mmol, 0.15 equiv.), THF (16 mL) and Grignard reagent in THF (6.0 mmol, 6.0 mL, 1.5 equiv.) were used in step 1. **5f-s2** (743 mg, 2.9 mmol, 1.0 equiv.), iodine (152 mg, 0.6 mmol, 20 mol%) and DMSO (2.9 mL) were used in step 2. This yielded **5f** (300 mg, 28% over 2 steps) as a yellow oil.

IR (film) ν_{max}/cm^{-1}: 3458, 2937, 1688, 1331, 1123, 913.

^{1}H NMR (500 MHz, $CDCl_3$) δ 8.20 (s, 1H, C4*H*), 8.10 (d, *J* = 7.8 Hz, 1H, C6*H*), 7.90 (d, *J* = 7.8 Hz, 1H, C2*H*), 7.68 (t, *J* = 7.8 Hz, 1H, C1*H*), 5.80 – 5.72 (m, 1H, C13*H*), 5.13 – 5.10 (m, 1H, C8*H*), 5.03 – 4.96 (m, 2H, C14H_2), 3.60 (d, *J* = 5.4 Hz, 1H, O*H*), 2.16 – 2.05 (m, 2H, C12H_2), 1.93 – 1.86 (m, 1H, C10H_A), 1.73 – 1.49 (m, 3H, C10H_B, C11H_2).

^{19}F NMR (470 MHz, $CDCl_3$) δ –62.9.

^{13}C NMR (126 MHz, $CDCl_3$) δ 201.0 (C7), 138.0 (C13), 134.3 (C5), 131.7 (q, *J* = 33.3 Hz, C3), 131.6 (C6), 130.4 (q, *J* = 3.7 Hz, C2), 129.6 (C1), 125.4 (q, *J* = 4.0 Hz, C4), 123.5 (q, *J* = 272.4 Hz, C16), 115.2 (C14), 73.0 (C8), 34.9 (C10), 33.1 (C12), 23.9 (C11).

HRMS (ESI): calculated for $C_{14}H_{15}F_3O_2Na$ $[M+Na]^+$ requires m/z 295.0922, found m/z 295.0913.

2-Hydroxy-1-(naphthalen-2-yl)hept-6-en-1-one 5g

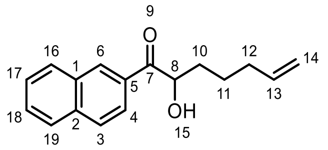

5g

Prepared according to *General Procedure A* and *General Procedure D* via a 2 step sequence. **5g-s1** (953 mg, 5.0 mmol, 1.0 equiv.), CuI (143 mg, 0.75 mmol, 0.15 equiv.), THF (20 mL) and Grignard reagent in THF (7.5 mmol, 7.5 mL, 1.5 equiv.) were used in step 1. **5g-s2** (882 mg, 3.7 mmol, 1.0 equiv.), iodine (188 mg, 0.74 mmol, 20 mol%) and DMSO (3.7 mL) were used in step 2. This yielded **5g** (435 mg, 34% over 2 steps) as a colorless solid.

m.p. = 37–39 °C.

IR (film) ν_{max}/cm^{-1}: 3468, 2939, 1676, 1282, 1087, 912.

^{1}H NMR (500 MHz, $CDCl_3$) δ 8.45 (s, 1H, C6*H*), 8.01 – 7.92 (m, 4H, C(3, 4, 16, 19)*H*), 7.68 – 7.60 (m, 2H, C(17, 18)*H*), 5.82 – 5.74 (m, 1H, C13*H*), 5.28 – 5.25 (m, 1H, C8*H*), 5.04 – 5.00 (dt, *J* = 17.0, 1.6 Hz, 1H, C14H_B), 4.98 – 4.95 (m, 1H, C14H_A), 3.80 (d, *J* = 6.5 Hz, 1H, O*H*), 2.16 – 2.05 (m, 2H, C12H_2), 2.02 – 1.95 (m, 1H, C10H_A), 1.76 – 1.52 (m, 3H, C11H_2, C10H_B).

^{13}C NMR (126 MHz, $CDCl_3$) δ 202.0 (C7), 138.3 (C13), 136.0 (C5), 132.4 (C1), 130.9 (C2), 130.4 (C6), 129.7 (C16), 129.0 (C18), 128.9 (C3), 127.9 (C19), 127.1 (C17), 124.0 (C4), 115.0 (C14), 72.9 (C8), 35.4 (C10), 33.3 (C12), 24.1 (C11).

HRMS (ESI): calculated for $C_{17}H_{18}O_2Na$ $[M+Na]^+$ requires m/z 277.1204, found m/z 277.1203.

1-(Furan-2-yl)-2-hydroxyhept-6-en-1-one 5h

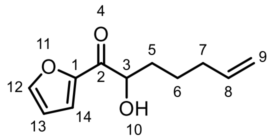

5h

Prepared according to *General Procedure A* and *General Procedure D* via a 2 step sequence. **5h-s1** (783 mg, 6.0 mmol, 1.0 equiv.), CuI (172 mg, 0.9 mmol, 0.15 equiv.), THF (24 mL) and Grignard reagent in THF (9.0 mmol, 9.0 mL, 1.5 equiv.) were used in step 1. **5h-s2** (385 mg, 2.2 mmol, 1.0 equiv.), iodine (112 mg, 0.44 mmol, 20 mol%) and DMSO (2.2 mL) were used in step 2. This yielded **5h** (138 mg, 12% over 2 steps) as a colorless solid.

m.p. = 34–36 °C.

IR (film) ν_{max}/cm^{-1}: 3422, 2940, 1672, 1464, 1032, 909.

^{1}H NMR (500 MHz, $CDCl_3$) δ 7.66 (d, *J* = 1.5 Hz, 1H, C14*H*), 7.33 (d, *J* = 3.6 Hz, 1H, C12*H*), 6.62 (dd, *J* = 3.7, 1.7 Hz, 1H, C13*H*), 5.84 – 5.76(m, 1H, C8*H*), 5.05 – 4.96 (m, 2H, C9H_2), 4.86 (m, 1H, C3H_2), 3.47 (d, *J* = 6.4 Hz, 1H, O*H*), 2.18 – 2.06 (m, 2H, C7H_2), 1.99 – 1.91 (m, 1H, C5H_A), 1.70 – 1.53 (m, 3H, C5H_B, C6H_2).

^{13}C NMR (126 MHz, $CDCl_3$) δ 190.6 (C2), 150.3 (C1), 147.1 (C14), 138.3 (C8), 118.9 (C12), 114.9 (C9), 112.6 (C13), 73.3 (C3), 35.0 (C5), 33.3 (C7), 24.3 (C6).

HRMS (ESI): calculated for $C_{11}H_{15}O_3$ $[M+H]^+$ requires m/z 195.1021, found m/z 195.1012.

2-Hydroxy-1-(1-methyl-1*H*-indol-2-yl)hept-6-en-1-one 5i

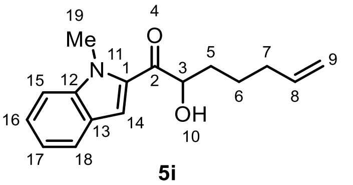

5i

Prepared according to *General Procedure C* and *General Procedure D* via a 3 step sequence. **5i-s5** (876 mg, 5.0 mmol, 1.0 equiv.), DMAP (61 mg, 0.5 mmol, 0.1 equiv.), MeONHMe·HCl (634 mg, 6.5 mmol, 1.3 equiv.), CH_2Cl_2 (50 mL), EDC·HCl (1246 mg, 6.5 mmol, 1.3 equiv.) and Et_3N (0.93 mL, 6.65 mmol, 1.33 equiv.) were used in step 1. **5i-s6** (720 mg, 3.3 mmol, 1.0 equiv.), THF (33 mL) and Grignard reagent in THF (4.0 mmol, 4.0 mL, 1.2 equiv.) were used in step 2. **5i-s2** (483 mg, 2.0 mmol, 1.0 equiv.), iodine (102 mg, 0.4 mmol, 20 mol%) and DMSO (2.0 mL) were used in step 3. This yielded **5i** (200 mg, 16% over 3 steps) as a yellow oil.

IR (film) ν_{max}/cm^{-1}: 3463, 2941, 1652, 1376, 1083, 912.

^{1}H NMR (500 MHz, $CDCl_3$) δ 7.74 (d, *J* = 8.0 Hz, 1H, C15*H*), 7.45 – 7.44 (m, 2H, C(16, 18)*H*), 7.30 (s, 1H, C14*H*), 7.24 – 7.19 (m, 1H, C17*H*), 5.84 – 5.76 (m, 1H, C8*H*), 5.05 – 4.96 (m, 3H, C3*H*, C9H_2), 4.12 (s, 3H, C19H_3), 3.64 (d, *J* = 6.8 Hz, 1H, O*H*), 2.18 – 2.06 (m, 2H, C7H_2), 2.03 – 1.95 (m, 1H, C5H_A), 1.74 – 1.54 (m, 3H, C5H_B, C6H_2).

^{13}C NMR (126 MHz, $CDCl_3$) δ 195.1 (C2), 140.4 (C1), 138.3 (C8), 131.5 (C12), 126.6 (C16), 125.9 (C13), 123.2 (C15), 121.1 (C17), 114.9 (C9), 112.2 (C14), 110.5 (C18), 73.5 (C3), 37.1 (C5), 33.4 (C7), 32.2 (C19), 24.5 (C6).

HRMS (ESI): calculated for $C_{16}H_{20}NO_2$ $[M+H]^+$ requires m/z 258.1494, found m/z 258.1495.

1-(3-(Cyclopropylmethoxy)-4-(difluoromethoxy)phenyl)-2-hydroxyhept-6-en-1-one 5j

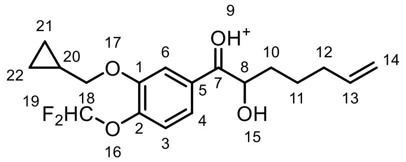

5j

Prepared according to *General Procedure C* and *General Procedure D* via a 3 step sequence. **5j-s5** (258 mg, 1.0 mmol, 1.0 equiv.), DMAP (12.3 mg, 0.1 mmol, 0.1 equiv.), MeONHMe·HCl (127 mg, 1.3 mmol, 1.3 equiv.), CH_2Cl_2 (10 mL), EDC·HCl (249 mg, 1.3 mmol, 1.3 equiv.) and Et_3N (0.19 mL, 1.33 mmol, 1.33 equiv.) were used in step 1. **5j-s6** (302 mg, 1.0 mmol, 1.0 equiv.), THF (10 mL) and Grignard reagent in THF (1.2 mmol, 1.2 mL, 1.2 equiv.) were used in step 2. **5j-s2** (227 mg, 0.7 mmol, 1.0 equiv.), iodine (36 mg, 0.14 mmol, 20 mol%) and DMSO (0.7 mL) were used in step 3. This yielded **5j** (105 mg, 31% over 3 steps) as a pale yellow oil.

IR (film) ν_{max}/cm^{-1}: 3474, 2926, 1679, 1508, 1108, 912.

^{1}H NMR (500 MHz, $CDCl_3$) δ 7.57 (d, *J* = 2.0 Hz, 1H, C6*H*), 7.46 (dd, *J* = 8.2, 2.0 Hz, 1H, C4*H*), 7.27 (d, *J* = 8.2 Hz, 1H, C3*H*), 6.77 (t, *J* = 74.7 Hz, 1H, C19*H*), 5.81 – 5.73 (m, 1H, C13*H*), 5.06 – 4.96 (m, 3H, C8*H*, C14H_2), 4.00 – 3.94 (m, 2H, C18H_2), 3.62 (s, 1H, O*H*), 2.15 – 2.04 (m, 2H, C12H_2), 1.93 – 1.86 (m, 1H, C10H_A), 1.70 – 1.48 (m, 3H, C10H_B, C11H_2), 1.37 – 1.29 (m, 1H, C20*H*), 0.70 (q, *J* = 6.0 Hz, 2H, C21$H_{a'}$ C22H_a), 0.40 (q, *J* = 5.0 Hz, 2H, C21$H_{b'}$ C22H_b).

^{19}F NMR (470 MHz, $CDCl_3$) δ –82.0, –82.1.

^{13}C NMR (126 MHz, $CDCl_3$) δ 200.8 (C7), 150.8 (C1), 144.8 (t, J = 3.1 Hz, C2), 138.2 (C13), 131.6 (C5), 122.0 (C4), 121.9 (C3), 115.6 (t, J = 262.0 Hz, C19), 115.0 (C14), 113.8 (C6), 74.2 (C18), 72.8 (C8), 35.4 (C10), 33.2 (C12), 24.1 (C11), 10.0 (C20), 3.3 (C(21, 22)).

HRMS (ESI): calculated for $C_{18}H_{23}F_2O_4$ $[M+H]^+$ requires m/z 341.1559, found m/z 341.1568.

2-Hydroxy-1-phenyloct-7-en-1-one 7a

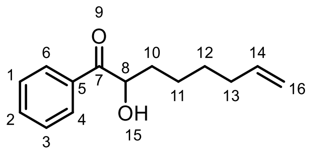

7a

Prepared according to *General Procedure A* and *General Procedure D* via a 2 step sequence. **7a-s1** (562 mg, 4.0 mmol, 1.0 equiv.), CuI (114 mg, 0.6 mmol, 0.15 equiv.), THF (16 mL) and Grignard reagent in THF (6.0 mmol, 6.0 mL, 1.5 equiv.) were used in step 1. **7a-s2** (668 mg, 3.3 mmol, 1.0 equiv.), iodine (168 mg, 0.66 mmol, 20 mol%) and DMSO (3.3 mL) were used in step 2. This yielded **7a** (484 mg, 55% over 2 steps) as a pale yellow oil.

IR (film) ν_{max}/cm^{-1}: 3475, 2927, 1679, 1266, 910, 696.

^{1}H NMR (500 MHz, $CDCl_3$) δ 7.94 – 7.92 (m, 2H, C(4, 6)*H*), 7.66 – 7.63 (m, 1H, C2*H*), 7.53 – 7.51 (m, 2H, C(1, 3)*H*), 5.82 – 5.74 (m, 1H, C14*H*), 5.11– 5.09 (m, 1H, C8*H*), 4.99 – 4.92 (m, 2H, C16H_2), 3.71 (s, 1H, O*H*), 2.06 – 2.02 (m, 2H, C13H_2), 1.93 – 1.85 (m, 1H, C10H_A), 1.61 – 1.52 (m, 2H, C11H_A, C10H_B), 1.47 – 1.34 (m, 2H, C11H_B, C12H_2).

^{13}C NMR (126 MHz, $CDCl_3$) δ 202.1 (C7), 138.6 (C14), 133.9 (C2), 133.7 (C5), 128.9 (C4, C6), 128.5 (C1, 3), 114.5 (C16), 73.1 (C8), 35.7 (C10), 33.5 (C13), 28.6 (C12), 24.4 (C11).

HRMS (ESI): calculated for $C_{14}H_{19}O_2$ $[M+H]^+$ requires m/z 219.1385, found m/z 219.1386.

2-Hydroxy-1-(*p*-tolyl)oct-7-en-1-one 7b

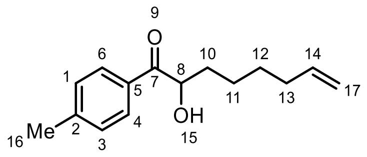

7b

Prepared according to *General Procedure A* and *General Procedure D* via a 2 step sequence. **7b-s1** (618 mg, 4.0 mmol, 1.0 equiv.), CuI (114 mg, 0.6 mmol, 0.15 equiv.), THF (16 mL) and Grignard reagent in THF (6.0 mmol, 6.0 mL, 1.5 equiv.) were used in step 1. **7b-s2** (757 mg, 3.5 mmol, 1.0 equiv.), iodine (178 mg, 0.7 mmol, 20 mol%) and DMSO (3.5 mL) were used in step 2. This yielded **7b** (230 mg, 25% over 2 steps) as a pale yellow oil.

IR (film) ν_{max}/cm^{-1}: 3334, 2973, 1677, 1380, 1047, 880.

^{1}H NMR (500 MHz, $CDCl_3$) δ 7.93 – 7.91 (d, J = 8.0 Hz, 2H, C(4, 6)*H*), 7.32 (d, J = 8.0 Hz, 2H, C(1, 3)*H*), 5.82 – 5.74 (m, 1H, C14*H*), 5.07– 5.05 (m, 1H, C8*H*), 4.99 – 4.92 (m, 2H, C17H_2), 3.74 (s, 1H, O*H*), 2.45 (s, 3H, C16*H*), 2.06 – 2.02 (m, 2H, C13H_2), 1.91 – 1.85 (m, 1H, C10H_A), 1.59 – 1.52 (m, 2H, C11H_A, C10H_B), 1.46 – 1.34 (m, 3H, C11H_B, C12H_2).

^{13}C NMR (126 MHz, $CDCl_3$) δ 201.6 (C7), 145.0 (C2), 138.6 (C14), 131.1 (C5), 129.6 (C(1, 3)), 128.7 (C(4, 6)), 128.5 (C1, 3), 114.5 (C17), 72.9 (C8), 35.9 (C10), 33.6 (C13), 28.6 (C12), 24.5 (C11), 21.8 (C16).

HRMS (ESI): calculated for $C_{15}H_{21}O_2$ $[M+H]^+$ requires m/z 233.1542, found m/z 233.1534.

1-(4-Chlorophenyl)-2-hydroxyoct-7-en-1-one 7c

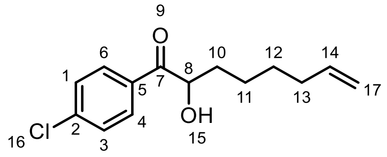

7c

Prepared according to *General Procedure A* and *General Procedure D* via a 2 step sequence. **7c-s1** (700 mg, 4.0 mmol, 1.0 equiv.), CuI (114.3 mg, 0.6 mmol, 0.15 equiv.), THF (16 mL) and Grignard reagent in THF (6.0 mmol, 6.0 mL, 1.5 equiv.) were used in step 1. **7c-s2** (687 mg, 2.9 mmol, 1.0 equiv.), iodine (152 mg, 0.6 mmol, 20 mol%) and DMSO (2.9 mL) were used in step 2. This yielded **7c** (171 mg, 17% over 2 steps) as a colorless solid.

m.p. = 34–36 °C.

IR (film) ν_{max}/cm^{-1}: 3311, 2919, 1683, 1401, 1096, 914.

^{1}H NMR (500 MHz, $CDCl_3$) δ 7.88 – 7.86 (m, 2H, C(4, 6)*H*), 7.51 – 7.50 (m 2H, C(1, 3)*H*), 5.82 – 5.74 (m, 1H, C14*H*), 5.05– 5.03 (m, 1H, C8*H*), 5.00 – 4.93 (m, 2H, C17*H*$_2$), 3.64 (d, *J* = 5.5 Hz, 1H, O*H*), 2.06 – 2.02 (m, 2H, C13*H*$_2$), 1.90 – 1.83 (m, 1H, C10H_A), 1.59 – 1.50 (m, 2H, C11H_A, C10H_B), 1.47 – 1.34 (m, 3H, C11H_B, C12*H*$_2$).

^{13}C NMR (126 MHz, $CDCl_3$) δ 201.0 (C7), 140.5 (C2), 138.5 (C14), 132.0 (C5), 129.9 (C(4, 6)), 129.3 (C(1, 3)), 114.6 (C17), 73.1 (C8), 35.7 (C10), 33.5 (C13), 28.6 (C12), 24.4 (C11).

HRMS (ESI): calculated for $C_{14}H_{18}Cl^{35}O_2$ $[M+H]^+$ requires m/z 253.0995, found m/z 253.0990.

1-(2-Fluorophenyl)-2-hydroxyoct-7-en-1-one 7d

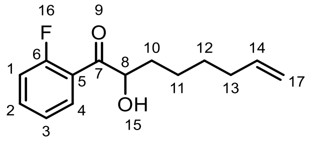

7d

Prepared according to *General Procedure A* and *General Procedure D* via a 2 step sequence. **7d-s1** (553 mg, 4.0 mmol, 1.0 equiv.), CuI (114 mg, 0.6 mmol, 0.15 equiv.), THF (16 mL) and Grignard reagent in THF (6.0 mmol, 6.0 mL, 1.5 equiv.) were used in step 1. **7d-s2** (639 mg, 2.9 mmol, 1.0 equiv.), iodine (152 mg, 0.6 mmol, 20 mol%) and DMSO (2.9 mL) were used in step 2. This yielded **7d** (160 mg, 24% over 2 steps) as a pale yellow oil.

IR (film) ν_{max}/cm^{-1}: 3325, 2974, 1691, 1416, 1047, 880.

^{1}H NMR (500 MHz, $CDCl_3$) δ 7.96 – 7.92 (m, 1H, C4*H*), 7.63 – 7.59 (m, 1H, C2*H*), 7.32 – 7.29 (m, 1H, C3*H*), 7.21 – 7.17 (m, 1H, C1*H*), 5.82 – 5.74 (m, 1H, C14*H*), 5.00– 4.91 (m, 3H, C8*H*, C17*H*$_2$), 3.72 (s, 1H, O*H*), 2.06 – 2.02 (m, 2H, C13*H*$_2$), 1.88 – 1.81 (m, 1H, C10H_A), 1.61 – 1.33 (m, 5H, C10H_B, C11*H*$_2$, C12*H*$_2$).

^{19}F NMR (470 MHz, $CDCl_3$) δ –108.4.

^{13}C NMR (126 MHz, $CDCl_3$) δ 200.6 (d, *J* = 4.5 Hz, C7), 161.6 (d, *J* = 255.5 Hz, C6), 138.7 (C14), 135.5 (d, *J* = 9.3 Hz, C2), 131.1 (d, *J* = 2.7 Hz, C4), 124.9 (d, *J* = 3.5 Hz, C3), 122.4 (d, *J* = 13.2 Hz, C5), 116.8 (d, *J* = 24.0 Hz, C1), 114.5 (C17), 76.5 (d, *J* = 9.0 Hz, C8), 34.5 (C10), 33.5 (C13), 28.5 (C12), 24.7 (C11).

HRMS (ESI): calculated for $C_{14}H_{18}FO_2$ $[M+H]^+$ requires m/z 237.1291, found m/z 237.1282.

2-Hydroxy-1-(3-methoxyphenyl)oct-7-en-1-one 7e

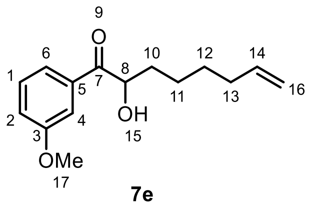

7e

Prepared according to *General Procedure A* and *General Procedure D* via a 2 step sequence. **7e-s1** (858 mg, 5.0 mmol, 1.0 equiv.), CuI (143 mg, 0.75 mmol, 0.15 equiv.), THF (20 mL) and Grignard reagent in THF (7.5 mmol, 7.5 mL, 1.5 equiv.) were used in step 1. **7e-s2** (813 mg, 3.5 mmol, 1.0 equiv.), iodine (178 mg, 0.7 mmol, 20 mol%) and DMSO (3.5 mL) were used in step 2. This yielded **7e** (437 mg, 35% over 2 steps) as a pale yellow oil.

IR (film) ν_{max}/cm^{-1}: 3344, 2973, 1684, 1269, 1046, 879.

^{1}H NMR (500 MHz, $CDCl_3$) δ 7.48 – 7.47 (m, 2H, C(1, 6)*H*), 7.44 – 7.41 (m, 1H, C4*H*), 7.19 – 7.17 (m, 1H, C2*H*), 5.82 – 5.74 (m, 1H, C14*H*), 5.08 – 5.05 (m, 1H, C8*H*), 5.00 – 4.92 (m, 2H, C16H_2), 3.89 (s, 3H, C17*H*), 3.68 (d, *J* = 6.4 Hz, 1H, O*H*), 2.06 – 2.02 (m, 2H, C13H_2), 1.92 – 1.85 (m, 1H, C10H_A), 1.58 – 1.52 (m, 2H, C10H_B, C11H_A), 1.47 – 1.34 (m, 3H, C11H_B, C12H_2).

^{13}C NMR (126 MHz, $CDCl_3$) δ 202.0 (C7), 160.0 (C3), 138.6 (C14), 135.0 (C5), 129.9 (C4), 121.0 (C1), 120.3 (C6), 114.5 (C16), 113.0 (C2), 73.2 (C8), 55.5 (C17), 35.8 (C10), 33.6 (C13), 28.6 (C11), 24.1 (C12).

HRMS (ESI): calculated for $C_{15}H_{21}O_3$ $[M+H]^+$ requires m/z 249.1491, found m/z 249.1491.

2-Hydroxy-1-(3-(trifluoromethyl)phenyl)oct-7-en-1-one 7f

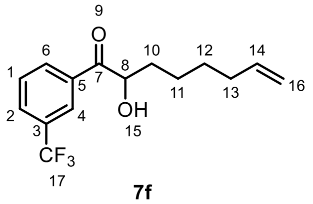

7f

Prepared according to *General Procedure A* and *General Procedure D* via a 2 step sequence. **7f-s1** (1042 mg, 5.0 mmol, 1.0 equiv.), CuI (143 mg, 0.75 mmol, 0.15 equiv.), THF (20 mL) and Grignard reagent in THF (7.5 mmol, 7.5 mL, 1.5 equiv.) were used in step 1. **7f-s2** (892 mg, 3.3 mmol, 1.0 equiv.), iodine (168 mg, 0.66 mmol, 20 mol%) and DMSO (3.3 mL) were used in step 2. This yielded **7f** (437 mg, 30% over 2 steps) as a pale yellow oil.

IR (film) ν_{max}/cm^{-1}: 3477, 2931, 1688, 1331, 1126, 911.

^{1}H NMR (500 MHz, $CDCl_3$) δ 8.20 (s, 1H, C4*H*), 8.10 (d, *J* = 7.8 Hz, 1H, C6*H*), 7.90 (d, *J* = 7.7 Hz, 1H, C2*H*), 7.69 (t, *J* = 7.7 Hz, 1H, C1*H*), 5.82 – 5.73 (m, 1H, C14*H*), 5.12 – 5.10 (m, 1H, C8*H*), 4.99 – 4.92 (m, 2H, C16H_2), 3.60 (s, 1H, O*H*), 2.07 – 2.03 (m, 2H, C13H_2), 1.92 – 1.84 (m, 1H, C10H_A), 1.61 – 1.52 (m, 2H, C10H_B, C11H_A), 1.49 – 1.35 (m, 3H, C11H_B, C12H_2).

^{19}F NMR (470 MHz, $CDCl_3$) δ –62.9.

^{13}C NMR (126 MHz, $CDCl_3$) δ 201.0 (C7), 138.4 (C14), 134.3 (C5), 131.7 (q, J = 33.1 Hz, C3), 131.6 (C6), 130.3 (q, J = 3.7 Hz, C2), 129.6 (C1), 125.4 (q, J = 3.8 Hz, C4), 123.5 (q, J = 273.2 Hz, C17), 114.6 (C16), 73.3 (C8), 35.5 (C10), 33.5 (C13), 28.5 (C11), 24.3 (C12).

HRMS (ESI): calculated for $C_{15}H_{18}F_3O_2$ $[M+H]^+$ requires m/z 287.1259, found m/z 287.1245.

2-Hydroxy-1-(naphthalen-2-yl)oct-7-en-1-one 7g

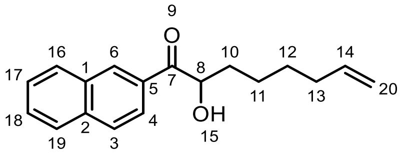

7g

Prepared according to *General Procedure A* and *General Procedure D* via a 2 step sequence. **7g-s1** (953 mg, 5.0 mmol, 1.0 equiv.), CuI (143 mg, 0.75 mmol, 0.15 equiv.), THF (20 mL) and Grignard reagent in THF (7.5 mmol, 7.5 mL, 1.5 equiv.) were used in step 1. **7g-s2** (892 mg, 4.2 mmol, 1.0 equiv.), iodine (213 mg, 0.85 mmol, 20 mol%) and DMSO (4.2 mL) were used in step 2. This yielded **7g** (551 mg, 41% over 2 steps) as a pale brown solid.

m.p. = 44–46 °C.

IR (film) ν_{max}/cm^{-1}: 3329, 2973, 1679, 1380, 1046, 880.

^{1}H NMR (500 MHz, $CDCl_3$) δ 8.45 (s, 1H, C6*H*), 8.02 – 7.92 (m, 4H, C(3, 4, 16, 19)*H*), 7.68 – 7.65 (m, 1H, Ar*H*), 7.63 – 7.59 (m, 1H, Ar*H*), 5.82 – 5.74 (m, 1H, C14*H*), 5.27 – 5.24 (m, 1H, C8*H*), 4.99 – 4.91 (m, 2H, C20H_2), 3.80 (d, J = 5.6 Hz, 1H, O*H*), 2.05 (d, J = 7.0 Hz, 2H, C13H_2), 2.00 – 1.93 (m, 1H, C10H_A), 1.68 – 1.57 (m, 2H, C10H_A, C11H_A), 1.52 – 1.35 (m, 3H, C11H_B, C12H_2).

^{13}C NMR (126 MHz, $CDCl_3$) δ 202.1 (C7), 138.6 (C14), 136.0 (C5), 132.4 (C1), 131.0 (C2), 130.4 (C6), 129.7 (C16), 129.0 (C18), 128.9 (C3), 127.9 (C19), 127.1 (C17), 124.0 (C4), 114.6 (C20), 73.2 (C8), 36.0 (C10), 33.6 (C13), 28.6 (C11), 24.4 (C12).

HRMS (ESI): calculated for $C_{18}H_{20}O_2Na$ $[M+Na]^+$ requires m/z 291.1361, found m/z 291.1364.

1-(Furan-2-yl)-2-hydroxyoct-7-en-1-one 7h

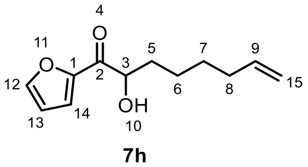

7h

Prepared according to *General Procedure A* and *General Procedure D* via a 2 step sequence. **7h-s1** (522 mg, 4.0 mmol, 1.0 equiv.), CuI (114 mg, 0.6 mmol, 0.15 equiv.), THF (16 mL) and Grignard reagent in THF (6.0 mmol, 6.0 mL, 1.5 equiv.) were used in step 1. **7h-s2** (269 mg, 1.4 mmol, 1.0 equiv.), iodine (70 mg, 0.28 mmol, 20 mol%) and DMSO (1.4 mL) were used in step 2. This yielded **7h** (100 mg, 12% over 2 steps) as a colorless solid.

m.p. = 40–42 °C

IR (film) ν_{max}/cm^{-1}: 3430, 2934, 1671, 1464, 1036, 909.

^{1}H NMR (500 MHz, $CDCl_3$) δ 7.66 (d, *J* = 1.6 Hz, 1H, C14*H*), 7.33 (d, *J* = 3.6 Hz, 1H, C12*H*), 6.62 (dd, *J* = 3.6, 1.6 Hz, 1H, C13*H*), 5.84 – 5.76 (m, 1H, C9*H*), 5.02 – 4.93 (m, 2H, C15*H₂*), 4.85 (dd, *J* = 8.0, 3.5 Hz, 1H, C3*H*), 3.47 (s, 1H, O*H*), 2.07 (q, *J* = 6.6 Hz, 2H, C8H_2), 1.96 – 1.91 (m, 1H, C5H_A), 1.68 – 1.39 (m, 5H, C5H_B, C6H_2, C7H_2).

^{13}C NMR (126 MHz, $CDCl_3$) δ 190.6 (C2), 150.3 (C1), 147.1 (C14), 138.7 (C9), 118.9 (C12), 114.5 (C15), 112.6 (C13), 73.4 (C3), 35.4 (C5), 33.6 (C8), 28.6 (C7), 24.4 (C6).

HRMS (ESI): calculated for $C_{12}H_{17}O_3$ $[M+H]^+$ requires m/z 209.1178, found m/z 209.1179.

2-Hydroxy-1-(1-methyl-1*H*-indol-2-yl)oct-7-en-1-one 7i

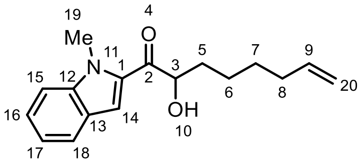

7i

Prepared according to *General Procedure C* and *General Procedure D* via a 3 step sequence. **7i-s5** (526 mg, 3.0 mmol, 1.0 equiv.), DMAP (37 mg, 0.3 mmol, 0.1 equiv.), MeONHMe·HCl (380 mg, 3.9 mmol, 1.3 equiv.), CH_2Cl_2 (30 mL), EDC·HCl (748 mg, 3.9 mmol, 1.3 equiv.) and Et_3N (0.56 mL, 4.0 mmol, 1.33 equiv.) were used in step 1. **7i-s6** (459 mg, 2.1 mmol, 1.0 equiv.), THF (21 mL) and Grignard reagent in THF (2.5 mmol, 2.5 mL, 1.2 equiv.) were used in step 2. **7i-s2** (306 mg, 1.2 mmol, 1.0 equiv.), iodine (61 mg, 0.24 mmol, 20 mol%) and DMSO (1.2 mL) were used in step 3. This yielded **7i** (105 mg, 13% over 3 steps) as a yellow oil.

IR (film) ν_{max}/cm^{-1}: 3473, 2925, 1652, 1512, 1085, 910.

^{1}H NMR (500 MHz, $CDCl_3$) δ 7.74 (dt, *J* = 8.0 Hz, 1.0 Hz, 1H, C15*H*), 7.45 – 7.44 (m, 2H, C(16, 18)*H*), 7.29 (s, 1H, C14*H*), 7.24 – 7.18 (m, 1H, C17*H*), 5.84 – 5.76 (m, 1H, C9*H*), 5.02 – 4.93 (m, 3H, C3*H*, C20H_2), 4.12 (s, 3H, C19H_3), 3.63 (s, 1H, O*H*), 2.09 – 2.05 (m, 2H, C8H_2), 2.02 – 1.96 (m, 1H, C5H_A), 1.73 – 1.36 (m, 5H, C5H_B, C6H_2, C7H_2).

^{13}C NMR (126 MHz, $CDCl_3$) δ 195.2 (C2), 140.3 (C1), 138.7 (C9), 131.5 (C12), 126.6 (C16), 125.9 (C13), 123.1 (C15), 121.1 (C17), 114.5 (C20), 112.1 (C14), 110.5 (C18), 73.7 (C3), 37.5 (C5), 33.6 (C8), 32.2 (C19), 28.7 (C7), 24.5 (C6).

HRMS (ESI): calculated for $C_{17}H_{22}NO_2$ $[M+H]^+$ requires m/z 272.1651, found m/z 272.1654.

1-(3-(Cyclopropylmethoxy)-4-(difluoromethoxy)phenyl)-2-hydroxyoct-7-en-1-one 7j

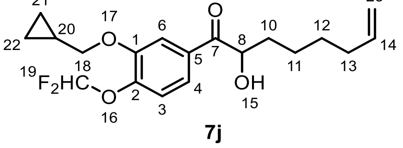

7j

Prepared according to *General Procedure C* and *General Procedure D* via a 3 step sequence. **7j-s5** (516 mg, 2.0 mmol, 1.0 equiv.), DMAP (25 mg, 0.2 mmol, 0.1 equiv.), MeONHMe·HCl (254 mg, 2.6 mmol, 1.3 equiv.), CH_2Cl_2 (20 mL), EDC·HCl (498 mg, 2.6 mmol, 1.3 equiv.) and Et_3N (0.38 mL, 2.66 mmol, 1.33 equiv.) were used in step 1. **7j-s6** (603 mg, 2.0 mmol, 1.0 equiv.), THF (20 mL) and Grignard reagent in THF (2.4 mmol, 2.4

mL, 1.2 equiv.) were used in step 2. **7j-s2** (237 mg, 0.7 mmol, 1.0 equiv.), iodine (36 mg, 0.14 mmol, 20 mol%) and DMSO (0.7 mL) were used in step 3. This yielded **7j** 233 mg, 33% over 3 steps) as a pale yellow oil.

IR (film) ν_{max}/cm^{-1}: 3474, 2927, 1679, 1268, 1111, 913.

^{1}H NMR (500 MHz, $CDCl_3$) δ 7.57 (d, *J* = 1.9 Hz, 1H, C6*H*), 7.46 (dd, *J* = 8.3 Hz, 1.9 Hz, 1H, C4*H*), 7.27 (d, *J* = 8.3 Hz, 1H, C3*H*), 6.77 (t, *J* = 74.8 Hz, 1H, C19*H*), 5.82 – 5.74 (m, 1H, C14*H*), 5.04 – 4.93 (m, 3H, C8*H*, C23H_2), 4.00 – 3.94 (m, 2H, C18H_2), 3.61 (s, 1H, O*H*), 2.07 – 2.03 (m, 2H, C13H_2), 1.91 – 1.84 (m, 1H, C10H_A), 1.61 – 1.28 (m, 6H, C10H_B, C11H_2, C12H_2, C20*H*), 0.72 – 0.68 (m, 2H, C21H_2), 0.40 (q, *J* = 5.0 Hz, 2H, C22H_2).

^{19}F NMR (470 MHz, $CDCl_3$) δ –82.0, –82.1.

^{13}C NMR (126 MHz, $CDCl_3$) δ 200.8 (C7), 150.8 (C1), 144.8 (t, *J* = 3.1 Hz, C2), 138.5 (C14), 131.7 (C5), 122.0 (C4), 121.9 (C3), 115.6 (t, *J* = 262.0 Hz, C19), 114.6 (C23), 113.8 (C6), 74.2 (C18), 73.0 (C8), 35.9 (C10), 33.5 (C13), 28.6 (C12), 24.1 (C11), 10.0 (C20), 3.3 (C(21, 22)).

HRMS (ESI): calculated for $C_{19}H_{25}F_2O_4$ $[M+H]^+$ requires m/z 355.1721, found m/z 355.1726.

3-Hydroxyoct-7-en-2-one 5l

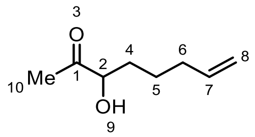

5l

Prepared according to *General Procedure A* and *General Procedure D* via a 2 step sequence. **5l-s1** (314 mg, 4.0 mmol, 1.0 equiv.), CuI (114 mg, 0.6 mmol, 0.15 equiv.), THF (16 mL) and Grignard reagent in THF (6.0 mmol, 6.0 mL, 1.5 equiv.) were used in step 1. **5l-s2** (328 mg, 2.6 mmol, 1.0 equiv.), iodine (130 mg, 0.52 mmol, 20 mol%) and DMSO (2.6 mL) were used in step 2. This yielded **5l** (89 mg, 16% over 2 steps) as a colorless oil.

^{1}H NMR (500 MHz, $CDCl_3$) δ 5.85 – 5.77 (m, 1H, C7*H*), 5.07 – 4.99 (m, 2H, C8H_2), 4.22 – 4.01 (m, 1H, C2*H*), 3.48 (s, 1H, O*H*), 2.22 (s, 3H, C10*H*), 2.17 – 2.07 (m, 2H, C4H_2), 1.62 – 1.44 (m, 4H, C5H_2, C6H_2). The spectroscopic properties were consistent with the data available in the literature.[12]

3-Hydroxynon-8-en-2-one 7l

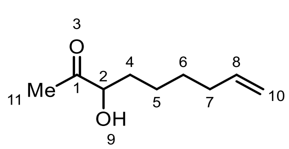

7l

Prepared according to *General Procedure A* and *General Procedure D* via a 2 step sequence. **7l-s1** (785 mg, 10.0 mmol, 1.0 equiv.), CuI (286 mg, 1.5 mmol, 0.15 equiv.), THF (40 mL) and Grignard reagent in THF (15.0 mmol, 15.0 mL, 1.5 equiv.) were used in step 1. **7l-s2** (1122 mg, 8.0 mmol, 1.0 equiv.), iodine (406 mg, 1.6 mmol, 20 mol%) and DMSO (8.0 mL) were used in step 2. This yielded **7l** (291 mg, 18% over 2 steps) as a pale yellow oil.

IR (film) ν_{max}/cm^{-1}: 3458, 2928, 1713, 1358, 1096, 910.

^{1}H NMR (500 MHz, $CDCl_3$) δ 5.85 – 5.77 (m, 1H, C8*H*), 5.02 (m, 1H, C10H_A), 4.96 (m, 1H, C10H_B), 4.20 (m, 1H, C2*H*), 3.48 (s, 1H, O*H*), 2.22 (s, 3H, C11*H*), 2.08 (q, *J* = 6.9 Hz, 2H, C7H_2), 1.89 – 1.83 (m, 1H, C4H_A), 1.61 – 1.33 (m, 5H, C4H_B, C5H_2, C6H_2).

^{13}C NMR (126 MHz, $CDCl_3$) δ 209.9 (C1), 138.6 (C8), 114.6 (C10), 76.8 (C2), 33.5 (C7), 33.4 (C4), 28.7 (C6), 25.2 (C11), 24.2 (C5).

HRMS (ESI): calculated for $C_9H_{16}O_2Na$ $[M+Na]^+$ requires m/z 179.1048, found m/z 179.1040.

2-Hydroxy-1-phenylhex-5-en-1-one 9

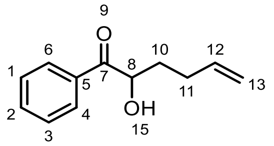

9

Prepared according to *General Procedure A* and *General Procedure B* via a 3 step sequence. **9-s1** (704 mg, 5.0 mmol, 1.0 equiv.), CuI (143 mg, 0.75 mmol, 0.15 equiv.), THF (20 mL) and Grignard reagent in THF (7.5 mmol, 7.5 mL, 1.5 equiv.) were used in step 1. **9-s2** (680 mg, 3.9 mmol, 1.0 equiv.), LDA (5.9 mmol in 5.9 mL THF, 1.5 equiv.), CH_2Cl_2 (8 mL) and NBS (837 mg, 4.7 mmol, 1.2 equiv.) were used in step 2. **9-s3** (709 mg, 2.8 mmol, 1.0 equiv.), MeOH (3.4 mL) and sodium formate (762 mg, 11.2 mmol, 4.0 equiv.) were used in step 3. This yielded **9** (249 mg, 26% over 3 steps) as a pale yellow oil.

^{1}H NMR (500 MHz, $CDCl_3$) δ 7.94 – 7.92 (m, 2H, C(4, 6)*H*), 7.67 – 7.63 (m, 1H, C2*H*), 7.55 – 7.52 (m, 2H, C(1, 3)*H*), 5.87 – 5.79 (m, 1H, C12*H*), 5.13 – 5.12 (m, 1H, C17*H*$_2$, C8*H*), 3.71 (d, *J* = 6.4 Hz, 1H, O*H*), 2.37 – 2.29 (m, 1H, C10*H*$_A$), 2.26 – 2.18 (m, 1H, C10*H*$_B$), 2.01 – 1.94 (m, 1H, C11*H*$_A$), 1.67 – 1.60 (m, 1H, C11*H*$_B$). The spectroscopic properties were consistent with the data available in the literature.[13]

2-Hydroxy-1-phenylnon-8-en-1-one 10

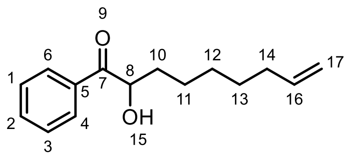

10

Prepared according to *General Procedure A* and *General Procedure B* via a 3 step sequence. **10-s1** (704 mg, 5.0 mmol, 1.0 equiv.), CuI (143 mg, 0.75 mmol, 0.15 equiv.), THF (20 mL) and Grignard reagent in THF (7.5 mmol, 7.5 mL, 1.5 equiv.) were used in step 1. **10-s2** (909 mg, 4.2 mmol, 1.0 equiv.), LDA (6.3 mmol in 6.3 mL THF, 1.5 equiv.), CH_2Cl_2 (8.4 mL) and NBS (897 mg, 5.0 mmol, 1.2 equiv.) were used in step 2. **10-s3** (650 mg, 2.2 mmol, 1.0 equiv.), MeOH (2.6 mL) and sodium formate (598 mg, 8.8 mmol, 4.0 equiv.) were used in step 3. This yielded **10** (207 mg, 19% over 3 steps) as a pale yellow oil.

IR (film) ν_{max}/cm^{-1}: 3350, 2928, 1682, 1450, 1047, 698.

^{1}H NMR (500 MHz, $CDCl_3$) δ 7.94 – 7.92 (m, 2H, C(4, 6)*H*), 7.66 – 7.63 (m, 1H, C2*H*), 7.54 – 7.51 (m, 2H, C(1, 3)*H*), 5.83 – 5.75 (m, 1H, C16*H*), 5.11 – 5.08 (m, 1H, C8*H*), 5.00 – 4.92 (m, 2H, C17*H*$_2$), 3.71 (d, *J* = 6.2 Hz, 1H, O*H*), 2.05 – 2.01 (m, 2H, C14*H*$_2$), 1.92 – 1.84 (m, 1H, C10*H*$_A$), 1.60 – 1.25 (m, 7H, C(11, 12, 13)*H*$_2$, C10*H*$_B$).

^{13}C NMR (126 MHz, $CDCl_3$) δ 202.2 (C7), 138.9 (C16), 133.9 (C2), 133.7 (C5), 128.9 (C4, C6), 128.5 (C1, 3), 114.3 (C17), 73.1 (C8), 35.9 (C10), 33.6 (C14), 28.8 (C13), 28.7 (C11), 24.8 (C12).

HRMS (ESI): calculated for $C_{15}H_{20}O_2Na$ $[M+Na]^+$ requires m/z 255.1361, found m/z 255.1365.

Preparation of 1-(3-bromopropyl)-2-vinylbenzene s9[14]

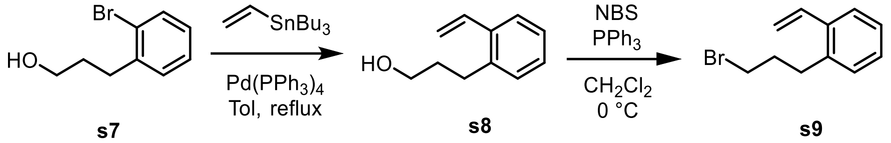

s7 **s8** **s9**

Under an argon atmosphere, to a solution of $Pd(PPh_3)_4$ (693 mg, 0.6 mmol, 0.05 equiv.) in PhMe (120 mL) was added commercially available alcohol **s7** (12 mmol, 1 equiv.) and tributylvinylstannane (7.61 g, 24 mmol, 2 equiv.). The resulting solution was heated at reflux for 24 h. The mixture was cooled to r.t. and H_2O (150 mL) was added. The mixture was then extracted with EtOAc (2 × 150 mL). The combined organic extracts were washed with brine (150 mL), dried (Na_2SO_4) and concentrated *in vacuo*. The residue was purified by FCC (10:1 to 4:1 petrol/EtOAc) to afford alcohol **s8**. Under an argon atmosphere, a solution of alcohol **s8** (12 mmol, 1.0 equiv.) in CH_2Cl_2 (40 mL) was cooled to 0 °C in an ice bath. Triphenylphosphine (3.94 g, 15 mmol, 1.2 equiv.) was added in one portion and the resulting solution was maintained at 0 °C for 10 minutes with stirring. Following this period, *N*-bromosuccinimide (2.67 g, 15 mmol, 1.2 equiv.) was added portion-wise over 5 minutes. The reaction was then maintained at 0 °C for 1 h until alcohol **s8** was consumed (as indicated by TLC analysis). The reaction mixture was concentrated *in vacuo* and purified by FCC (10:1 to 4:1 petrol/EtOAc) to afford bromide **s9**. Spectroscopic data were in accordance with the literature.[15]

1-Phenyl-4-(2-vinylphenyl)butan-1-one 7m-s10

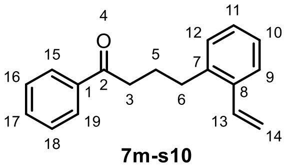

7m-s10

Prepared according to *General Procedure E.* **s9** (540.4 mg, 2.4 mmol, 1.2 equiv.) was used to prepare the Grignard reagent (2.4 mmol in 2.4 mL THF), which was used in THF (8 mL) with **7m-s1** (282 mg, 2.0 mmol, 1.0 equiv.). This yielded **7m-s10** (452 mg, 90%) as a colorless oil.

IR (film) ν_{max}/cm^{-1}: 2917, 1684, 1450, 1048, 690.

^{1}H NMR (500 MHz, $CDCl_3$) δ 7.96 – 7.94 (m, 2H, C(15, 19)*H*), 7.59 – 7.46 (m, 4H, Ar*H*), 7.24 – 7.18 (m, 3H, Ar*H*), 7.05 (dd, *J* = 17.4 Hz, 10.9 Hz, 1H, C13*H*), 5.67 (d, *J* = 17.4 Hz, 1H, C14H_A), 5.31 (d, *J* = 10.9 Hz, 1H, C14H_B), 3.02 (m, 2H, C3H_2), 2.81 (t, *J* = 7.9 Hz, 2H, C6H_2), 2.09 – 2.03 (m, 2H, C5H_2).

^{13}C NMR (126 MHz, $CDCl_3$) δ 200.0 (C2), 139.1 (C7), 137.0 (C8), 136.6 (C1), 134.6 (C13), 133.0 (ArC), 129.7 (ArC), 128.6 (C15, 19), 128.0 (C16, 18), 127.8 (ArC), 126.5 (ArC), 125.8 (ArC), 115.6 (C14), 37.8 (C3), 32.6 (C6), 25.3 (C5).

HRMS (ESI): calculated for $C_{18}H_{19}O$ $[M+H]^+$ requires m/z 251.1436, found m/z 251.1428.

2-Hydroxy-1-phenyl-4-(2-vinylphenyl)butan-1-one 7m

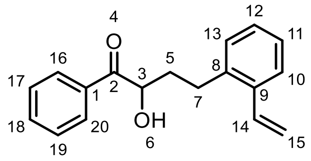

7m

Prepared according to *General Procedure D.* **7m-s10** (452 mg, 1.8 mmol, 1.0 equiv.), iodine (91.4 mg, 0.36 mmol, 20 mol%) and DMSO (1.8 mL) were used. This yielded **7m** (215 mg. 45%) as a pale yellow oil.

IR (film) ν_{max}/cm^{-1}: 3348, 2973, 1680, 1449, 1047, 880.

^{1}H NMR (500 MHz, $CDCl_3$) δ 7.80 – 7.79 (m, 2H, C(16, 20)*H*), 7.63 – 7.60 (m, 1H, C18*H*), 7.50 – 7.46 (m, 3H, C(17, 19, 12)*H*), 7.24 – 7.16 (m, 3H, C(10, 11, 13)*H*), 6.86 (dd, *J* = 17.4 Hz, 10.9 Hz, 1H, C14*H*), 5.60 (d, *J* = 17.4 Hz, 1H, C15H_A), 5.20 (d, *J* = 10.9 Hz, 1H, C15H_B), 5.07 – 5.03 (m, 1H, C3*H*), 3.84 (d, *J* = 5.9 Hz, 1H, O*H*), 2.96 – 2.83 (m, 2H, C7H_2), 2.19 – 2.12 (m, 1H, C5H_A), 1.85 – 1.78 (m, 1H, C5H_B).

^{13}C NMR (126 MHz, $CDCl_3$) δ 201.9 (C2), 138.3 (C8), 136.7 (C9), 134.3 (C14), 134.0 (C18), 133.4 (C1), 130.0 (C13), 128.9 (C17, 19), 128.6 (C16, 20), 127.9 (C12), 126.7 (C10), 125.9 (C11), 115.7 (C15), 72.4 (C3), 37.0 (C5), 28.7 (C7).

HRMS (ESI): calculated for $C_{18}H_{19}O_2$ $[M+H]^+$ requires m/z 267.1385, found m/z 267.1386.

1-(*p*-Tolyl)-4-(2-vinylphenyl)butan-1-one 7n-s10

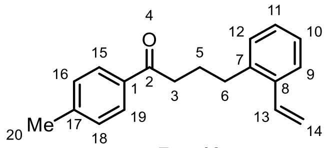

7n-s10

Prepared according to *General Procedure E.* **s9** (811 mg, 3.6 mmol, 1.2 equiv.) was used to prepare Grignard reagent (3.6 mmol in 3.6 mL THF), which was used in THF (12 mL) with **7n-s1** (464 mg, 3.0 mmol, 1.0 equiv.). This yielded **7n-s10** (611 mg, 77%) as a colorless oil.

IR (film) ν_{max}/cm^{-1}: 2925, 1680, 1606, 1179, 910.

^{1}H NMR (500 MHz, $CDCl_3$) δ 7.85 (d, *J* = 7.9 Hz, 2H, C(15, 19)*H*), 7.54 – 7.51 (m, 1H, Ar*H*), 7.28 – 7.18 (m, 5H, Ar*H*), 7.06 (dd, *J* = 17.4 Hz, 10.9 Hz, 1H, C13*H*), 5.67 (d, *J* = 17.4 Hz, 1H, C14H_A), 5.32 (d, *J* = 10.9 Hz, 1H, C14H_B), 3.00 (t, *J* = 7.2 Hz, 2H, C3H_2), 2.81 (t, *J* = 7.7 Hz, 2H, C6H_2), 2.43 (s, 3H, C20H_3), 2.08 – 2.02 (m, 2H, C5H_2).

^{13}C NMR (126 MHz, $CDCl_3$) δ 199.7 (C2), 143.7 (C17), 139.2 (C7), 136.6 (C8), 134.6 (C13), 134.6 (C1), 129.7 (ArC), 129.3 (C(16, 18)), 128.2 (C(15, 19)), 127.8 (ArC), 126.4 (ArC), 125.8 (ArC), 115.6 (C14), 37.7 (C3), 32.6 (C6), 25.4 (C5), 21.6 (C20).

HRMS (ESI): calculated for $C_{19}H_{20}ONa$ $[M+Na]^+$ requires m/z 287.1412, found m/z 287.1411.

2-Hydroxy-1-(*p*-tolyl)-4-(2-vinylphenyl)butan-1-one 7n

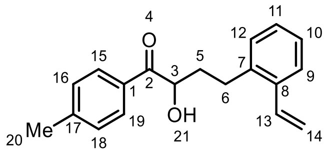

7n

Prepared according to *General Procedure D.* **7n-s10** (611 mg, 2.3 mmol, 1.0 equiv.), iodine (117 mg, 0.46 mmol, 20 mol%) and DMSO (2.3 mL) were used. This yielded **7n** (362 mg, 56%) as a colorless oil.

IR (film) ν_{max}/cm^{-1}: 3467, 2923, 1674, 1607, 1269, 911.

^{1}H NMR (500 MHz, $CDCl_3$) δ 7.70 (d, *J* = 8.1 Hz, 2H, C(15, 19)*H*), 7.51 – 7.48 (m, 1H, Ar*H*), 7.27 (d, *J* = 8.1 Hz, 2H, C(16, 18)*H*), 7.23 – 7.19 (m, 2H, Ar*H*), 7.18 – 7.16 (m, 1H, Ar*H*), 6.87 (dd, *J* = 17.4 Hz, 10.9 Hz, 1H, C13*H*), 5.61 (dd, *J* = 17.4 Hz, 1.4 Hz, 1H, C14H_A), 5.21 (dd, *J* = 10.9 Hz, 1.4 Hz, 1H, C14H_B), 5.03 – 5.00 (m, 1H, C3*H*), 3.86 (d, *J* = 6.2 Hz, 1H, O*H*), 2.95 – 2.82 (m, 2H, C6H_2), 2.44 (s, 3H, C20H_3), 2.17 – 2.10 (m, 1H, C5H_A), 1.83 – 1.76 (m, 1H, C5H_B).

^{13}C NMR (126 MHz, $CDCl_3$) δ 201.4 (C2), 145.0 (C17), 138.4 (C7), 136.6 (C8), 134.3 (C13), 130.8 (C1), 129.9 (ArC), 129.5 (C(16, 18)), 128.7 (C(15, 19)), 127.9 (ArC), 126.7 (ArC), 125.9 (ArC), 115.7 (C14), 72.3 (C3), 37.2 (C5), 28.7 (C6), 21.8 (C20).

HRMS (ESI): calculated for $C_{19}H_{20}O_2Na$ $[M+Na]^+$ requires m/z 303.1361, found m/z 303.1366.

1-(4-Bromophenyl)-4-(2-vinylphenyl)butan-1-one 7o-s10

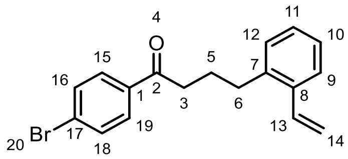

7o-s10

Prepared according to *General Procedure E.* **s9** (1351 mg, 6.0 mmol, 1.2 equiv.) was used to prepare Grignard reagent (6.0 mmol in 6.0 mL THF), which was used in THF (20 mL) with **7o-s1** (1094 mg, 5.0 mmol, 1.0 equiv.). This yielded **7o-s10** (1.1 g, 67%, with ~10% impurities) as a pale yellow oil.

IR (film) ν_{max}/cm^{-1}: 2934, 1684, 1269, 1070, 757.

^{1}H NMR (500 MHz, $CDCl_3$) δ 7.81 – 7.79 (m, 2H, C(15, 19)*H*), 7.62 – 7.60 (m, 2H, C(16, 18)*H*), 7.54 – 7.51 (m, 1H, Ar*H*), 7.25 – 7.16 (m, 3H, Ar*H*), 7.04 (dd, *J* = 17.4 Hz, 10.9 Hz, 1H, C13*H*), 5.67 (dd, *J* = 17.4 Hz, 1.1 Hz, 1H, C14H_A), 5.31 (dd, *J* = 10.9 Hz, 1.1 Hz, 1H, C14H_B), 2.97 (t, *J* = 7.0 Hz, 2H, C3H_2), 2.81 (t, *J* = 7.3 Hz, 2H, C6H_2), 2.08 – 2.02 (m, 2H, C5H_2).

^{13}C NMR (126 MHz, $CDCl_3$) δ 198.9 (C2), 139.0 (C7), 136.6 (C8), 135.7 (ArC), 134.5 (C13), 131.9 (C(16, 18)), 129.7 (ArC), 129.6 (C(15, 19)), 128.1 (ArC), 127.8 (ArC), 126.5 (ArC), 125.9 (ArC), 115.7 (C14), 37.7 (C3), 32.4 (C6), 25.2 (C5).

HRMS (ESI): calculated for $C_{18}H_{18}Br^{79}O$ $[M+H]^+$ requires m/z 329.0541, found m/z 329.0523.

1-(4-Bromophenyl)-2-hydroxy-4-(2-vinylphenyl)butan-1-one 7o

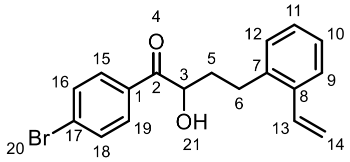

7o

Prepared according to *General Procedure D.* **7o-s10** (988 mg, 3.0 mmol, 1.0 equiv.), iodine (152 mg, 0.6 mmol, 20 mol%) and DMSO (3.0 mL) were used. This yielded **7o** (403 mg, 38%) as a yellow oil.

IR (film) ν_{max}/cm^{-1}: 3482, 2948, 1682, 1268, 1070, 974.

^{1}H NMR (500 MHz, $CDCl_3$) δ 7.63 – 7.59 (m, 4H, Ar*H*), 7.51 – 7.50 (m, 1H, Ar*H*), 7.26 – 7.21 (m, 2H, Ar*H*), 7.18 – 7.16 (m, 1H, Ar*H*), 6.87 (dd, *J* = 17.3 Hz, 10.9 Hz, 1H, C13*H*), 5.62 (dd, *J* = 17.3 Hz, 1.4 Hz, 1H, C14H_A), 5.24 (dd, *J* = 10.9 Hz, 1.4 Hz, 1H, C14H_B), 4.98 – 4.94 (m, 1H, C3*H*), 3.74 (d, *J* = 6.2 Hz, 1H, O*H*), 2.96 – 2.84 (m, 2H, C6H_2), 2.14 – 2.08 (m, 1H, C5H_A), 1.83 – 1.76 (m, 1H, C5H_B).

^{13}C NMR (126 MHz, $CDCl_3$) δ 200.9 (C2), 138.1 (C7), 136.7 (C8), 134.2 (C13), 132.2 (ArC), 132.1 (ArC), 130.1 (ArC), 130.0 (ArC), 129.2 (ArC), 128.0 (ArC), 126.8 (ArC), 126.0 (ArC), 115.9 (C14), 72.3 (C3), 36.9 (C5), 28.7 (C6).

HRMS (ESI): calculated for $C_{18}H_{18}Br^{79}O_2$ $[M+H]^+$ requires m/z 345.0490, found m/z 345.0495.

1-(3-(Trifluoromethyl)phenyl)-4-(2-vinylphenyl)butan-1-one 7p-s10

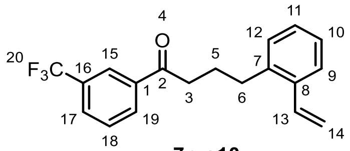

7p-s10

Prepared according to *General Procedure E.* **s9** (811 mg, 3.6 mmol, 1.2 equiv.) was used to prepare Grignard reagent (3.6 mmol in 3.6 mL THF), which was used in THF (12 mL) with **7p-s1** (626 mg, 3.0 mmol, 1.0 equiv.). This yielded **7p-s10** (740 mg, 77%, with ~10% impurities) as a colorless oil.

IR (film) ν_{max}/cm^{-1}: 2928, 1693, 1328, 1125, 912.

^{1}H NMR (500 MHz, $CDCl_3$) δ 8.19 (s, 1H, C(15)*H*), 8.12 (d, *J* = 8.0 Hz, 1H, C(19)*H*), 7.83 (d, *J* = 8.0 Hz, 1H, C(17)*H*), 7.62 (t, *J* = 8.0 Hz, 1H, C(18)*H*), 7.55 – 7.51 (m, 1H, Ar*H*), 7.25 – 7.18 (m, 3H, Ar*H*), 7.05 (dd, *J* = 17.4 Hz, 10.9 Hz, 1H, C13*H*), 5.67 (d, *J* = 17.4 Hz, 1H, C14H_A), 5.32 (d, *J* = 10.9 Hz, 1H, C14H_B), 3.04 (t, *J* = 7.2 Hz, 2H, C3H_2), 2.83 (t, *J* = 7.7 Hz, 2H, C6H_2), 2.11 – 2.05 (m, 2H, C5H_2).

^{19}F NMR (470 MHz, $CDCl_3$) δ –62.8.

^{13}C NMR (126 MHz, $CDCl_3$) δ 198.5 (C2), 138.9 (C7), 137.5 (C1), 136.7 (C8), 134.5 (C13), 131.2 (q, *J* = 32.9 Hz, C16), 131.1 (ArC), 129.7 (ArC), 129.4 (q, *J* = 3.6 Hz, ArC), 129.3 (ArC), 127.9 (ArC), 126.6 (ArC), 125.9 (ArC), 124.9 (q, *J* = 3.8 Hz, ArC), 123.7 (q, *J* = 273.1 Hz, C20), 115.7 (C14), 37.9 (C3), 32.4 (C6), 25.1 (C5).

HRMS (ESI): calculated for $C_{19}H_{18}F_3O$ $[M+H]^+$ requires m/z 319.1310, found m/z 319.1292.

2-Hydroxy-1-(3-(trifluoromethyl)phenyl)-4-(2-vinylphenyl)butan-1-one 7p

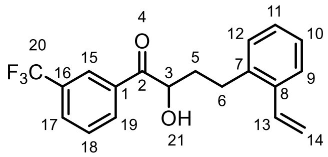

7p

Prepared according to *General Procedure D.* **7p-s10** (700 mg, 2.2 mmol, 1.0 equiv.), iodine (112 mg, 0.44 mmol, 20 mol%) and DMSO (2.2 mL) were used.This yielded **7p** (360 mg, 49%) as a pale yellow oil.

IR (film) ν_{max}/cm^{-1}: 3481, 2924, 1687, 1331, 1127, 913.

^{1}H NMR (500 MHz, $CDCl_3$) δ 8.02 (s, 1H, C(15)*H*), 7.88 (m, 2H, C(17, 19)*H*), 7.60 (t, *J* = 7.9 Hz, 1H, C(18)*H*), 7.50 – 7.48 (m, 1H, Ar*H*), 7.25 – 7.21 (m, 2H, Ar*H*), 7.19 – 7.17 (m, 1H, Ar*H*), 6.87 (dd, *J* = 17.4 Hz, 10.9 Hz, 1H, C13*H*), 5.59 (dd, *J* = 17.4 Hz, 1.1 Hz, 1H, C14H_A), 5.21 (dd, *J* = 10.9 Hz, 1.1 Hz, 1H, C14H_B), 5.04 – 5.01 (m, 1H, C3*H*), 3.70 (d, *J* = 6.3 Hz, 1H, O*H*), 2.99 – 2.88 (m, 2H, C6H_2), 2.17 – 2.10 (m, 1H, C5H_A), 1.85 – 1.78 (m, 1H, C5H_B).

^{19}F NMR (470 MHz, $CDCl_3$) δ –62.9.

^{13}C NMR (126 MHz, $CDCl_3$) δ 200.7 (C2), 137.9 (C7), 137.7 (C8), 134.1 (C13), 134.0 (C1), 131.6 (q, *J* = 33.2 Hz, C16), 131.6 (ArC), 130.3 (q, *J* = 3.7 Hz, ArC), 130.1 (ArC), 129.5 (ArC), 128.0 (ArC), 126.9 (ArC), 126.1 (ArC), 125.3 (q, *J* = 3.8 Hz, ArC), 123.4 (q, *J* = 273.0 Hz, C20), 115.9 (C14), 72.4 (C3), 36.7 (C5), 28.6 (C6).

HRMS (ESI): calculated for $C_{19}H_{17}F_3O_2Na$ $[M+Na]^+$ requires m/z 357.1078, found m/z 357.1065.

1-(3-Methoxyphenyl)-4-(2-vinylphenyl)butan-1-one 7q-s10

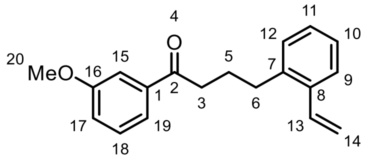

7q-s10

Prepared according to *General Procedure E.* **s9** (811 mg, 3.6 mmol, 1.2 equiv.) was used to prepare Grignard reagent (3.6 mmol in 3.6 mL THF), which was used in THF (12 mL) with **7q-s1** (512 mg, 3.0 mmol, 1.0 equiv.). This yielded **7q-s10** (660 mg) as a colorless oil.

IR (film) ν_{max}/cm^{-1}: 2918, 1683, 1463, 1258, 919.

^{1}H NMR (500 MHz, $CDCl_3$) δ 7.54 – 7.49 (m, 3H, Ar*H*), 7.38 (t, *J* = 7.9 Hz, 1H, C18*H*), 7.24 – 7.18 (m, 3H, Ar*H*), 7.12 (m, 1H, Ar*H*), 7.06 (dd, *J* = 17.4 Hz, 10.9 Hz, 1H, C13*H*), 5.67 (d, *J* = 17.4 Hz, 1H, C14H_A), 5.32 (d, *J* = 10.9 Hz, 1H, C14H_B), 3.88 (s, 3H, C20H_3), 3.01 (t, *J* = 7.2 Hz, 2H, C3H_2), 2.81 (t, *J* = 7.9 Hz, 2H, C6H_2), 2.08 – 2.02 (m, 2H, C5H_2).

^{13}C NMR (126 MHz, $CDCl_3$) δ 199.9 (C2), 159.8 (C16), 139.1 (C7), 138.4 (C1), 136.6 (C8), 134.6 (C13), 129.7 (ArC), 129.6 (ArC), 127.8 (ArC), 126.5 (ArC), 125.8 (ArC), 120.7 (ArC), 119.5 (ArC), 115.6 (C14), 112.2 (ArC), 55.5 (C20), 38.0 (C3), 32.6 (C6), 25.4 (C5).

HRMS (ESI): calculated for $C_{19}H_{20}O_2Na$ $[M+Na]^+$ requires m/z 303.1361, found m/z 303.1350.

2-Hydroxy-1-(3-methoxyphenyl)-4-(2-vinylphenyl)butan-1-one 7q

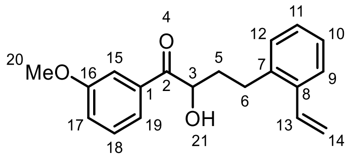

7q

Prepared according to *General Procedure D.* **7q-s10** (617 mg, 2.2 mmol, 1.0 equiv.), iodine (112 mg, 0.44 mmol, 20 mol%) and DMSO (2.2 mL) were used. This yielded **7q** (377 mg, 58%) as a pale yellow oil.

IR (film) ν_{max}/cm^{-1}: 3474, 2925, 1679, 1453, 1270, 910.

^{1}H NMR (500 MHz, $CDCl_3$) δ 7.51 – 7.47 (m, 1H, Ar*H*), 7.38 – 7.31 (m, 3H, Ar*H*), 7.23 – 7.21 (m, 2H, Ar*H*), 7.18 – 7.15 (m, 2H, Ar*H*), 6.87 (dd, *J* = 17.3 Hz, 10.9 Hz, 1H, C13*H*), 5.61 (dd, *J* = 17.3 Hz, 1.3 Hz, 1H, C14H_A), 5.22 (dd, *J* = 10.9 Hz, 1.3 Hz, 1H, C14H_B), 5.04 – 5.00 (m, 1H, C3*H*), 3.84 (s, 3H, C20H_3), 3.80 (d, *J* = 6.2 Hz, 1H, O*H*), 2.96 – 2.83 (m, 2H, C6H_2), 2.18 – 2.11 (m, 1H, C5H_A), 1.85 – 1.77 (m, 1H, C5H_B).

^{13}C NMR (126 MHz, $CDCl_3$) δ 201.8 (C2), 159.9 (C16), 138.3 (C7), 136.6 (C8), 134.7 (C1), 134.3 (C13), 130.0 (ArC), 129.8 (ArC), 127.9 (ArC), 126.7 (ArC), 125.9 (ArC), 121.0 (ArC), 120.6 (ArC), 115.7 (C14), 112.7 (ArC), 72.5 (C3), 55.5 (C20), 37.1 (C5), 28.7 (C6).

HRMS (ESI): calculated for $C_{19}H_{20}O_3Na$ $[M+Na]^+$ requires m/z 319.1310, found m/z 319.1304.

1-(2-Fluorophenyl)-4-(2-vinylphenyl)butan-1-one 7r-s10

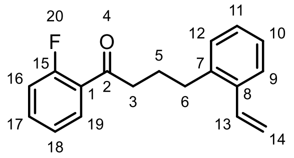

7r-s10

Prepared according to *General Procedure E.* **s9** (811 mg, 3.6 mmol, 1.2 equiv.) was used to prepare Grignard reagent (3.6 mmol in 3.6 mL THF), which was used in THF (12 mL) with **7r-s1** (476 mg, 3.0 mmol, 1.0 equiv.). This yielded **7r-s10** (630 mg, 78%) as a colorless oil.

IR (film) ν_{max}/cm^{-1}: 2929, 1684, 1451, 1211, 910.

^{1}H NMR (500 MHz, $CDCl_3$) δ 7.88 (m, 1H, C(19)*H*), 7.55 – 7.51 (m, 2H, Ar*H*), 7.26 – 7.13 (m, 5H, Ar*H*), 7.05 (dd, *J* = 17.4 Hz, 10.9 Hz, 1H, C13*H*), 5.67 (d, *J* = 17.4 Hz, 1H, C14H_A), 5.31 (d, *J* = 10.9 Hz, 1H, C14H_B), 3.05 (td, *J* = 7.0 Hz, 3.0 Hz, 2H, C3H_2), 2.80 (t, *J* = 7.9 Hz, 2H, C6H_2), 2.06 – 2.00 (m, 2H, C5H_2).

^{19}F NMR (470 MHz, $CDCl_3$) δ –109.5.

^{13}C NMR (126 MHz, $CDCl_3$) δ 198.4 (d, *J* = 4.1 Hz, C2), 161.9 (d, *J* = 254.1 Hz, C15), 139.2 (C7), 136.6 (C8), 134.5 (C13), 134.4 (d, *J* = 9.1 Hz, ArC), 130.6 (d, *J* = 2.7 Hz, C19), 129.6 (ArC), 127.8 (ArC), 126.4 (ArC), 125.8 (d, *J* = 13.0 Hz, C1), 125.8 (ArC), 124.4 (d, *J* = 3.3 Hz, ArC), 116.7 (d, *J* = 24.1 Hz, C16), 115.6 (C14), 43.0 (d, *J* = 7.1 Hz, C3), 32.6 (C6), 25.2 (d, *J* = 1.9 Hz, C5).

HRMS (ESI): calculated for $C_{18}H_{17}FONa$ $[M+Na]^+$ requires m/z 291.1161, found m/z 291.1150.

1-(2-Fluorophenyl)-2-hydroxy-4-(2-vinylphenyl)butan-1-one 7r

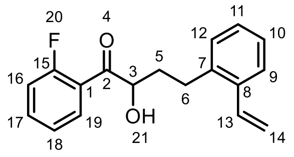

7r

Prepared according to *General Procedure D.* **7r-s10** (590 mg, 2.2 mmol, 1.0 equiv.), iodine (112 mg, 0.44 mmol, 20 mol%) and DMSO (2.2 mL) were used. This yielded **7r** (257 mg, 41% yield) as a pale yellow oil.

IR (film) ν_{max}/cm^{-1}: 3488, 2919, 1679, 1453, 1084, 910.

^{1}H NMR (500 MHz, $CDCl_3$) δ 7.89 (m, 1H, Ar*H*), 7.63 – 7.58 (m, 1H, Ar*H*), 7.47 – 7.45 (m, 1H, Ar*H*), 7.29 (m, 1H, Ar*H*), 7.20 – 7.12 (m, 4H, Ar*H*), 6.85 (dd, *J* = 17.4 Hz, 10.9 Hz, 1H, C13*H*), 5.57 (dd, *J* = 17.4 Hz, 1.2 Hz, 1H, C14H_A), 5.19 (dd, *J* = 10.9 Hz, 1.2 Hz, 1H, C14H_B), 5.04 – 5.00 (m, 1H, C(3)*H*), 3.87 (d, *J* = 5.7 Hz, O*H*), 2.93 – 2.84 (m, 2H, C6H_2), 2.15 – 2.08 (m, 1H, C5H_A), 1.77 – 1.69 (m, 1H, C5H_B).

^{19}F NMR (470 MHz, $CDCl_3$) δ –108.3.

^{13}C NMR (126 MHz, $CDCl_3$) δ 200.2 (d, *J* = 4.5 Hz, C2), 161.6 (d, *J* = 255.5 Hz, C15), 138.6 (C7), 136.6 (C8), 135.6 (d, *J* = 9.3 Hz, ArC), 134.2 (ArC13), 131.1 (d, *J* = 2.8 Hz, C19), 129.7 (ArC), 127.8 (ArC), 126.6 (ArC), 125.8 (ArC), 124.9 (d, *J* = 3.4 Hz, ArC), 122.3 (d, *J* = 13.3 Hz, C1), 116.8 (d, *J* = 23.6 Hz, C16), 115.6 (C14), 76.3 (d, *J* = 8.9 Hz, C3), 35.9 (C5), 29.1 (C6).

HRMS (ESI): calculated for $C_{18}H_{17}FO_2Na$ $[M+Na]^+$ requires m/z 307.1110, found m/z 307.1104.

1-(Naphthalen-2-yl)-4-(2-vinylphenyl)butan-1-one 7s-s10

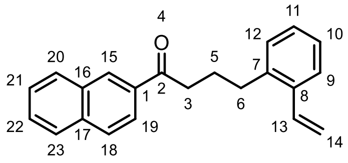

7s-s10

Prepared according to *General Procedure E.* **s9** (811 mg, 3.6 mmol, 1.2 equiv.) was used to prepare Grignard reagent (3.6 mmol in 3.6 mL THF), which was used in THF (12 mL) with **7s-s1** (572 mg, 3.0 mmol, 1.0 equiv.). This yielded **7s-s10** (707 mg, 78%) as a pale yellow oil.

IR (film) ν_{max}/cm^{-1}: 2938, 1678, 1467, 1123, 910.

^{1}H NMR (500 MHz, $CDCl_3$) δ 8.44 (s, 1H, C15*H*), 8.04 (m, 1H, C19*H*), 7.97 (m, 1H, C18*H*), 7.92 – 7.89 (m, 2H, Ar*H*), 7.64 – 7.53 (m, 3H, Ar*H*), 7.26 – 7.21 (m, 3H, Ar*H*), 7.08 (dd, *J* = 17.4 Hz, 10.9 Hz, 1H, C13*H*), 5.68 (d, *J* = 17.4 Hz, 1H, C14H_A), 5.32 (d, *J* = 10.9 Hz, 1H, C14H_B), 3.16 (t, *J* = 7.2 Hz, 2H, C3H_2), 2.86 (t, *J* = 7.7 Hz, 2H, C6H_2), 2.16 – 2.10 (m, 2H, C5H_2).

^{13}C NMR (126 MHz, $CDCl_3$) δ 200.0 (C2), 139.2 (C7), 136.7 (C8), 135.6 (C1), 134.6 (C13), 134.3 (ArC), 132.6 (ArC), 129.7 (C15), 129.7 (ArC), 129.6 (ArC), 128.4 (ArC), 128.4 (ArC), 127.8 (ArC), 127.8 (ArC), 126.8 (ArC), 126.5 (ArC), 126.5 (ArC), 123.9 (C19), 115.6 (C14), 37.9 (C3), 32.6 (C6), 25.4 (C5).

HRMS (ESI): calculated for $C_{22}H_{20}ONa$ $[M+Na]^+$ requires m/z 323.1412, found m/z 323.1407.

2-Hydroxy-1-(naphthalen-2-yl)-4-(2-vinylphenyl)butan-1-one 7s

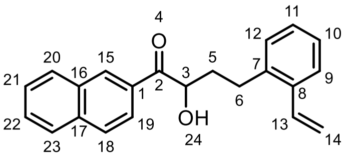

7s

Prepared according to *General Procedure D.* **7s-s10** (661 mg, 2.2 mmol, 1.0 equiv.), iodine (112 mg, 0.44 mmol, 20 mol%) and DMSO (2.2 mL) were used. This yielded **7s** (320 mg, 46%) as a yellow oil.

IR (film) ν_{max}/cm^{-1}: 3472, 2928, 1673, 1280, 1106, 910.

^{1}H NMR (500 MHz, $CDCl_3$) δ 8.19 (s, 1H, C15*H*), 7.92 – 7.87 (m, 4H, Ar*H*), 7.67 – 7.64 (m, 1H, Ar*H*), 7.61 – 7.58 (m, 1H, Ar*H*), 7.53 – 7.52 (m, 1H, Ar*H*), 7.28 – 7.20 (m, 3H, Ar*H*), 6.88 (dd, *J* = 17.4 Hz, 10.9 Hz, 1H, C13*H*), 5.58 (dd, *J* = 17.4 Hz, 1.2 Hz, 1H, C14H_A), 5.19 (dd, *J* = 8.3 Hz, 2.6 Hz, 1H, C3*H*), 5.12 (dd, *J* = 10.9 Hz, 1.2 Hz, 1H, C14H_B), 3.90 (s, 1H, O*H*), 3.04 – 2.98 (m, 1H, C6H_A), 2.94 – 2.89 (m, 1H, C6H_B), 2.27 – 2.21 (m, 1H, C5H_A), 1.93 – 1.85 (m, 1H, C5H_B).

^{13}C NMR (126 MHz, $CDCl_3$) δ 201.8 (C2), 138.4 (C7), 136.7 (C8), 135.9 (C1), 134.2 (C13), 132.4 (ArC), 130.6 (ArC), 130.5 (C15), 130.1 (ArC), 129.6 (ArC), 129.0 (ArC), 128.8 (ArC), 127.9 (ArC), 127.9 (ArC), 127.1 (ArC), 126.8 (ArC), 126.0 (ArC), 123.9 (C19), 115.8 (C14), 72.3 (C3), 37.3 (C6), 28.8 (C5).

HRMS (ESI): calculated for $C_{22}H_{20}O_2Na$ $[M+Na]^+$ requires m/z 339.1361, found m/z 339.1361.

1-(Furan-2-yl)-4-(2-vinylphenyl)butan-1-one 7t-s10

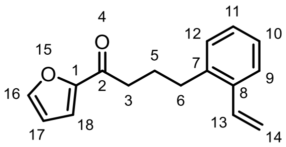

7t-s10

Prepared according to *General Procedure E.* **s9** (811 mg, 3.6 mmol, 1.2 equiv.) was used to prepare Grignard reagent (3.6 mmol in 3.6 mL THF), which was used in THF (12 mL) with **7t-s1** (392 mg, 3.0 mmol, 1.0 equiv.). This yielded **7t-s10** (506 mg, 70%) as a yellow oil.

IR (film) ν_{max}/cm^{-1}: 2936, 1672, 1467, 1012, 908, 757.

^{1}H NMR (500 MHz, $CDCl_3$) δ 7.59 (d, *J* = 1.4 Hz, 1H, C18*H*), 7.54 – 7.50 (m, 1H, Ar*H*), 7.24 – 7.21 (m, 2H, Ar*H*), 7.19 – 7.16 (m, 2H, Ar*H*), 7.04 (dd, *J* = 17.4 Hz, 10.8 Hz, 1H, C13*H*), 6.54 (dd, *J* = 3.5 Hz, 1.7 Hz, 1H, C17*H*), 5.67 (dd, *J* = 17.4 Hz, 1.1 Hz, 1H, C14H_A), 5.32 (d, *J* = 10.8 Hz, 1.1 Hz, 1H, C14H_B), 2.88 (t, *J* = 7.2 Hz, 2H, C3H_2), 2.79 (t, *J* = 7.9 Hz, 2H, C6H_2), 2.06 – 2.00 (m, 2H, C5H_2).

^{13}C NMR (126 MHz, $CDCl_3$) δ 189.2 (C2), 152.8 (C1), 146.2 (C18), 139.0 (C7), 136.6 (C8), 134.5 (C13), 129.6 (ArC), 127.8 (ArC), 126.5 (ArC), 125.8 (ArC), 116.8 (ArC), 115.6 (C14), 112.2 (C17), 37.8 (C3), 32.6 (C6), 25.2 (C5).

HRMS (ESI): calculated for $C_{16}H_{17}O_2$ $[M+H]^+$ requires m/z 241.1229, found m/z 241.1220.

1-(Furan-2-yl)-2-hydroxy-4-(2-vinylphenyl)butan-1-one 7t

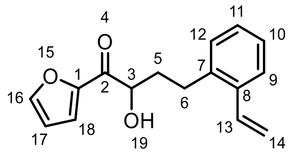

7t

Prepared according to *General Procedure D.* **7t-s10** (529 mg, 2.2 mmol, 1.0 equiv.), iodine (112 mg, 0.44 mmol, 20 mol%) and DMSO (2.2 mL) were used. This yielded **7t** (259 mg, 46%) as an orange oil.

IR (film) ν_{max}/cm^{-1}: 3459, 2934, 1668, 1465, 1280, 1016, 912.

^{1}**H NMR** (500 MHz, $CDCl_3$) δ 7.63 (d, *J* = 1.6 Hz, 1H, C18*H*), 7.52 – 7.49 (m, 1H, Ar*H*), 7.22 – 7.18 (m, 4H, Ar*H*), 6.95 (dd, *J* = 17.4 Hz, 11.0 Hz, 1H, C13*H*), 6.59 (dd, *J* = 3.6 Hz, 1.7 Hz, 1H, C17*H*), 5.64 (dd, *J* = 17.4 Hz, 1.3 Hz, 1H, C14H_A), 5.27 (dd, *J* = 11.0 Hz, 1.3 Hz, 1H, C14H_B), 4.85 – 4.82 (m, 1H, C3*H*), 3.61 (d, *J* = 6.3 Hz, 1H, O*H*), 2.95 – 2.86 (m, 2H, C6H_2), 2.25 – 2.18 (m, 1H, C5H_A), 1.95 – 1.87 (m, 1H, C5H_B).

^{13}**C NMR** (126 MHz, $CDCl_3$) δ 190.2 (C2), 150.1 (C1), 147.2(C18), 138.6 (C7), 136.6(C8), 134.4 (C13), 129.8 (ArC), 127.9 (ArC), 126.6 (ArC), 125.9 (ArC), 119.0 (ArC), 115.7 (C14), 112.6 (C17), 73.0 (C3), 36.8 (C5), 28.7 (C6).

HRMS (ESI): calculated for $C_{16}H_{17}O_3$ $[M+H]^+$ requires m/z 257.1178, found m/z 257.1171.

3-Hydroxy-5-(2-vinylphenyl)pentan-2-one 7u

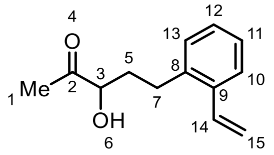

7u

Prepared according to *General Procedure E* and *General Procedure D* via a 2 step sequence. **s9** (811 mg, 3.6 mmol, 1.2 equiv.) was used to prepare Grignard reagent (3.6 mmol in 3.6 mL THF), which was used in THF (12 mL) with **7u-s1** (235.5 mg, 3.0 mmol, 1.0 equiv.) in step 1. **7t-s10** (414 mg, 2.2 mmol, 1.0 equiv.), iodine (112 mg, 0.44 mmol, 20 mol%) and DMSO (2.2 mL) were used in step 2. This yielded **7u** (188 mg, 25% over 2 steps) as a pale yellow oil.

IR (film) ν_{max}/cm^{-1}: 3456, 2919, 1712, 1451, 1086, 915.

^{1}**H NMR** (500 MHz, $CDCl_3$) δ 7.54 – 7.51 (m, 1H, C10*H*), 7.28 – 7.18 (m, 3H, Ar*H*), 7.03 (dd, 1H, *J* = 17.4 Hz, 11.0 Hz, C14*H*), 5.69 (d, 1H, *J* = 17.4 Hz, C15H_A), 5.35 (d, *J* = 11.0 Hz, 1H, C15H_B), 4.20 – 4.17 (m, 1H, C3*H*), 3.59 (d, *J* = 4.6 Hz, 1H, O*H*), 2.91 – 2.81 (m, 2H, C7H_2), 2.17 (s, 3H, C1H_3), 2.14 – 2.09 (m, 1H, C5H_A), 1.83 – 1.76 (m, 1H, C5H_B).

^{13}**C NMR** (126 MHz, $CDCl_3$) δ 209.6 (C2), 138.4 (C9), 136.6 (C8), 134.4 (C14), 129.8 (ArC), 128.0 (ArC), 126.7 (ArC), 126.0 (ArC), 115.9 (C15), 76.3 (C3), 34.7 (C5), 28.6 (C7), 25.1 (C1).

HRMS (ESI): calculated for $C_{13}H_{16}O_2Na$ $[M+Na]^+$ requires m/z 227.1048, found m/z 227.1042.

Synthesis of 2-hydroxy-1-phenyl-4-(2-(prop-1-en-2-yl)phenyl)butan-1-one 7v[16,14]

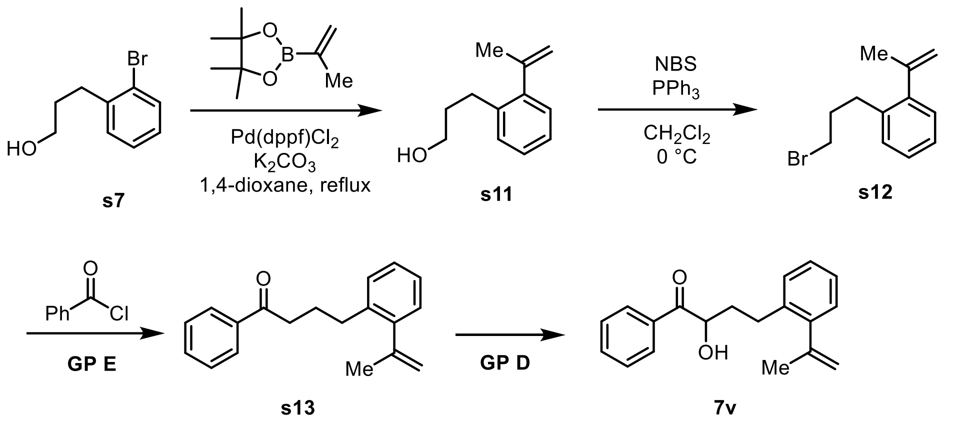

7v was synthesized from commercially sourced bromide **s7** according to reported procedures via a 4 step sequence. Alcohol **s7** (2151 mg, 10.00 mmol, 1.00 equiv), pinacol vinylboronate (2.29 mL, 12.00 mmol, 1.20 equiv) and Pd(dppf)Cl_2 (732 mg, 1.00 mmol, 0.10 equiv) were dissolved in dioxane (16 mL) under an N_2 atmosphere at 20 °C. Then, K_2CO_3 (1797 mg, 13.00 mmol, 1.30 equiv) in H_2O (4 mL) was added and the mixture was heated at reflux for 6 h. The mixture was cooled to r.t., brine (30 mL) was added, and the mixture was extracted with EtOAc (3 × 30 mL). The combined organic extracts were dried (Na_2SO_4) and concentrated *in vacuo*. The residue was purified by FCC (10:1 to 5:1 petrol/EtOAc) to give the desired alcohol **s11** as pale yellow oil, which was used directly to the next step. Under an argon atmosphere, a solution of alcohol **s11** (564 mg, 3.2 mmol, 1 equiv.) in CH_2Cl_2 (12 mL) was cooled to 0 °C in an ice bath. Triphenylphosphine (997 mg, 3.8 mmol, 1.2 equiv.) was added one portion and the resulting solution was maintained at 0 °C for 10 minutes. Following this, *N*-bromosuccinimide (676 mg, 3.8 mmol, 1.2 equiv.) was added portion-wise over 5 minutes. The reaction was then maintained at 0 °C for 1 h until alcohol **s11** was consumed (as indicated by TLC analysis). The mixture was concentrated *in vacuo* and the residue was purified by FCC (100:1 petrol/ Et_2O) to afford bromide **s12** which was used directly in the next step. Following *General Procedure E*, bromide **s12** was used to make the corresponding Grignard reagent (2.4 mmol, 1.2 equiv.), which was used with benzoyl chloride (2.4 mmol, 1 equiv.) and copper iodide (69 mg, 0.36 mmol, 15 mol%). The residue was purified by FCC (20:1 petrol/Et_2O) to afford ketone **s13**, which was used directly in the next step. Following *General Procedure D*, ketone **s13** (449 mg, 1.7 mmol, 1 equiv.), iodine (86 mg, 0.34 mmol, 20 mol%) and DMSO (3 mL) were used, and the residue was purified by FCC (10:1 to 4:1 petrol/Et_2O) to afford **7v** (220 mg, 18% over 4 steps) as a pale yellow oil.

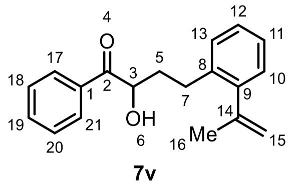

7v

IR (film) ν_{max}/cm^{-1}: 3466, 2933, 1709, 1493 1094, 990.

^{1}H NMR (500 MHz, $CDCl_3$) δ 7.85 – 7.84 (m, 2H, C(17, 21)*H*), 7.65 – 7.61 (m, 1H, C19*H*), 7.51 – 7.48 (m, 2H, C(18, 20)*H*), 7.21 – 7.15 (m, 3H, C(10, 12, 13)*H*), 7.10 – 7.07 (m, 1H, C11*H*), 5.12 – 5.09 (m, 1H, C3*H*), 5.04 – 5.03 (m, 1H, C15*H$_A$*), 4.71 (s, 1H, C15*H$_B$*), 3.81 (d, *J* = 5.9 Hz, 1H, O*H*), 2.89 – 2.75 (m, 2H, C7*H$_2$*), 2.19 – 2.12 (m, 1H, C5*H$_A$*), 1.95 (s, 3H, C16*H*). 1.86 – 1.78 (m, 1H, C5*H$_B$*).

^{13}**C NMR** (126 MHz, $CDCl_3$) δ 201.9 (C2), 138.3 (C8), 136.7 (C9), 134.3 (C14), 134.0 (C18), 133.4 (C1), 130.0 (C13), 128.9 (C17, 19), 128.6 (C16, 20), 127.9 (C12), 126.7 (C10), 125.9 (C11), 115.7 (C15), 72.4 (C3), 37.0 (C5), 28.7 (C7).

HRMS (ESI): calculated for $C_{19}H_{20}O_2Na$ $[M+Na]^+$ requires m/z 303.1361, found m/z 303.1360.

1-Phenyl-3-(2-vinylphenyl)propan-1-one 15-s10

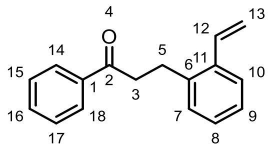

15-s10

Prepared according to *General Procedure E* **s9b** (760 mg, 3.6 mmol, 1.2 equiv.) was used to prepare Grignard reagent (3.6 mmol in 3.6 mL THF), which was used in THF (12 mL) with **15-s1** (422 mg, 3.0 mmol, 1.0 equiv.). This yielded **15-s10** (606 mg, 86% with ~5% impurities) as a pale yellow oil.

IR (film) ν_{max}/cm^{-1}: 2972, 1683, 1449, 1047, 880.

^{1}**H NMR** (500 MHz, $CDCl_3$) δ 7.98 (d, *J* = 7.9 Hz, 2H, C(14, 18)*H*), 7.60 – 7.54 (m, 2H, Ar*H*), 7.48 (t, *J* = 7.6 Hz, 2H, C(15, 17)*H*), 7.26 – 7.25 (m, 3H, Ar*H*), 7.05 (dd, *J* = 17.4 Hz, 11.0 Hz, 1H, C12*H*), 5.72 (d, *J* = 17.4 Hz, 1H, C13H_A), 5.36 (d, *J* = 11.0 Hz, 1H, C13H_B), 3.27 (t, *J* = 8.1 Hz, 2H, C3H_2), 3.18 (t, *J* = 8.1 Hz, 2H, C5H_2).

^{13}**C NMR** (126 MHz, $CDCl_3$) δ 199.2 (C2), 138.6 (C1), 136.8 (C6), 136.6 (C11), 134.4 (C12), 133.1 (ArC), 129.6 (ArC), 128.6 (C15, 17), 128.1 (C14, 18), 128.0 (ArC), 126.7 (ArC), 126.0 (ArC), 116.1 (C13), 39.9 (C3), 27.6 (C5).

HRMS (ESI): calculated for $C_{17}H_{17}O$ $[M+H]^+$ requires m/z 237.1279, found m/z 237.1280.

2-Hydroxy-1-phenyl-3-(2-vinylphenyl)propan-1-one 15

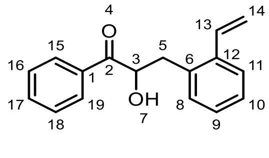

Prepared according to *General Procedure D.* **15-s10** (520 mg, 2.2 mmol, 1.0 equiv.), iodine (112 mg, 0.44 mmol, 20 mol%) and DMSO (2.2 mL) were used. This yielded **15** (156 mg, 28%) as an orange oil.

IR (film) ν_{max}/cm^{-1}: 3468, 3062, 1678, 1449, 1073, 915.

^{1}**H NMR** (500 MHz, $CDCl_3$) δ 7.92 – 7.90 (m, 2H, C(15, 19)*H*), 7.65 – 7.62 (m, 1H, C17*H*), 7.51 – 7.47 (m, 3H, C(16, 18, 9)*H*), 7.25 – 7.11 (m, 3H, C(8, 10, 11)*H*), 7.02 (dd, *J* = 17.4 Hz, 11.0 Hz, 1H, C13*H*), 5.65 (d, *J* = 17.4 Hz, 1H, C14H_A), 5.35 – 5.30 (m, 2H, C15H_B, C3*H*), 3.68 (d, *J* = 6.9 Hz, 1H, O*H*), 3.24 (dd, *J* = 14.4 Hz, 4.7 Hz, 1H, C5H_A), 2.95 (dd, *J* = 14.4 Hz, 7.7 Hz, 1H, C5H_B).

^{13}**C NMR** (126 MHz, $CDCl_3$) δ 201.6 (C2), 137.4 (C6), 135.0 (C13), 134.2 (C12), 134.0 (C17), 134.0 (C1), 130.7 (C8), 128.8 (C16, 18), 128.7 (C15, 19), 127.7 (C11), 127.3 (C10), 126.2 (C9), 116.4 (C14), 73.3 (C3), 39.3 (C5).

HRMS (ESI): calculated for $C_{17}H_{16}O_2Na$ $[M+Na]^+$ requires m/z 275.1048, found m/z 275.1046.

3-(2-Allylphenyl)-2-hydroxy-1-phenylpropan-1-one 11[17,14]

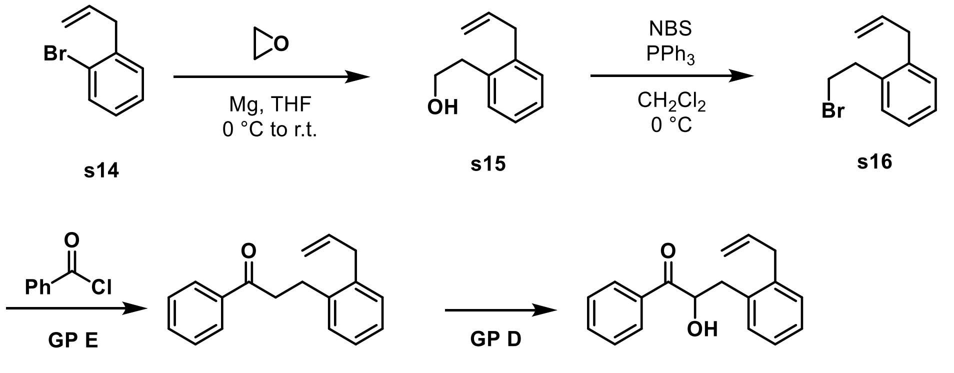

s17 11

11 was synthesized from commercially sourced bromide **s14** according to reported procedures via a 4 step sequence. A dry round bottom flask was charged with a stir bar and Mg (146 mg, 6 mmol, 1.2 equiv.) under a N_2 atmosphere. A quarter of the solution of bromide **s14** (1000 mg, 5 mmol, 1 equiv.) in THF (4 mL) was added to the flask and stirred. After the color of the mixture faded, the rest solution was added via dropping funnel. Then the mixture was stirred at reflux for 1 h. After that, oxirane (0.5 mL, 10 mmol, 2 equiv.) in THF (4 mL) was added dropwise at 0 °C. The reaction was stirred at r.t. for 1 h and then quenched with saturated aq. NH_4Cl. The mixture was extracted with EtOAc, dried (Na_2SO_4) and concentrated *in vacuo*. The residue was purified by FCC (10:1 to 4:1 petrol/Et_2O) to afford the product **s15** (780 mg, 95% yield). Data were in accordance with the literature.[17] Under an argon atmosphere, a solution of alcohol **s15** (779 mg, 4.8 mmol, 1 equiv.) in CH_2Cl_2 (20 mL) was cooled to 0 °C in an ice bath. Triphenylphosphine (1.36 g, 5.2 mmol, 1.2 equiv.) was added in one portion and the resulting solution was maintained at 0 °C for 10 minutes with stirring. Following this, *N*-bromosuccinimide (925 mg, 5.2 mmol, 1.2 equiv.) was added portion-wise as a solid over 5 minutes. The reaction was then maintained at 0 °C for 1 h until alcohol **s15** was consumed (as indicated by TLC analysis). The reaction was concentrated *in vacuo* and the residue was purified by FCC (50:1 petrol/Et_2O) to afford bromide **s16** which was used directly in the subsequent step. Data were in accordance with the literature.[17] Following *General Procedure E*, bromide **s16** (3.4 mmol, 1.2 equiv.), benzoyl chloride (2.8 mmol, 1 equiv.) and CuI (0.5 mmol, 0.15 equiv.) were used. Purification by FCC (20:1 petrol/Et_2O) afforded ketone **s17** which was used directly in the next step. Following *General Procedure D*, ketone **s17** (400 mg, 1.6 mmol, 1 equiv.), iodine (81 mg, 0.32 mmol, 20 mol%) and DMSO (3 mL) were used. Purification by FCC (10:1 to 4:1 petrol/Et_2O) afforded **11** (107 mg, 10% over 4 steps) as a yellow oil.

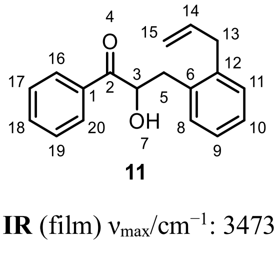

11

IR (film) ν_{max}/cm^{-1}: 3473, 3061, 1678, 1449, 1260, 971.

^{1}H NMR (500 MHz, $CDCl_3$) δ 7.95 – 7.93 (m, 2H, C(16, 20)*H*), 7.67 – 7.63 (m, 1H, C18*H*), 7.54 – 7.51 (m, 2H, C(17, 19)*H*), 7.23 – 7.15 (m, 4H, C(8, 9, 10, 11)*H*), 5.98 – 5.90 (m, 1H, C14*H*), 5.38 – 5.34 (m, 1H, C3*H*), 5.06 – 5.03 (m, 1H, C15H_A), 4.94 – 4.89 (m, 1H, C15H_B), 3.71 (d, J = 6.2 Hz, 1H, O*H*), 3.46 – 3.37 (m, 2H, C13H_2), 3.21 (dd, J = 14.7 Hz, 4.5 Hz, 1H, C5H_A), 2.88 (dd, J = 14.7 Hz, 8.2 Hz, 1H, C5H_B).

^{13}C NMR (126 MHz, $CDCl_3$) δ 201.6 (C2), 138.4 (C6), 137.1 (C14), 135.2 (C12), 134.1 (C18), 133.9 (C1), 130.3 (C11), 129.8 (C8), 128.9 (C16, 20), 128.7 (C17, C19), 127.1 (C10), 126.4 (C9), 115.9 (C15), 73.7 (C3), 38.5 (C5), 37.3 (C13).

HRMS (ESI): calculated for $C_{18}H_{18}O_2Na$ $[M+Na]^+$ requires m/z 289.1204, found m/z 289.1206.

2-Hydroxy-1-(2-vinylphenyl)ethan-1-one s20[18]

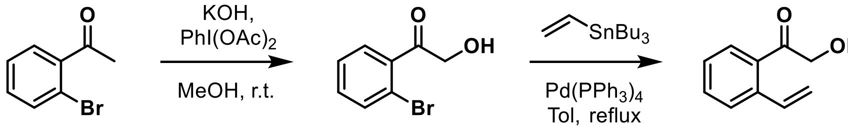

s18 **s19** **s20**

S20 was synthesized from commercially sourced bromide **s18** according to reported procedures via a 2 step sequence. Under an argon atmosphere, *o*-bromoacetophenone **s18** (740 mg, 3.72 mmol, 1.0 equiv.) was dissolved in anhydrous MeOH (20 mL), and then KOH (1150 mg, 20.5 mmol, 5.5 equiv.) and $PhI(OAc)_2$ (1433 mg, 4.45 mmol, 1.2 equiv.) were added. The mixture was stirred at r.t. for 5 h, and then concentrated *in vacuo*. The product was extracted with EtOAc, and the organic layer was washed with brine, dried ($MgSO_4$) and concentrated *in vacuo*. The residue was dissolved in MeOH (10 mL), and 2 M aq. HCl (10 mL) was added. The mixture was stirred at r.t. for 12 h, and MeOH was removed under vacuum. The product was extracted with EtOAc, and the organic layer was washed with saturated aq. Na_2CO_3 and brine, dried ($MgSO_4$) and concentrated *in vacuo*. Purification by FCC (10:1 to 4:1 petrol/EtOAc) afforded the product s**19** which was used directly in the next step. Data were in accordance with the literature.[18] Under an argon atmosphere, to a solution of $Pd(PPh_3)_4$ (75 mg, 0.065 mmol, 0.05 equiv.) in PhMe (15 mL) was added ketone **s19** (280 mg, 1.3 mmol, 1 equiv.) and tributylvinylstannane (824 mg, 2.6 mmol, 2 equiv.). The resulting solution was heated at reflux for 24 h. The mixture was cooled to r.t. and H_2O (20 mL) was added. The aqueous layer was extracted with EtOAc (2 × 25 mL), and the combined organic extracts were washed with brine (20 mL), dried (Na_2SO_4) and concentrated *in vacuo*. The residue was purified by FCC (10:1 to 4:1 petrol/EtOAc) to afford **s20** (107 mg, 45% over 2 steps) as a colorless oil.

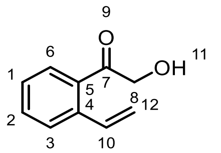

s20

IR (film) ν_{max}/cm^{-1}: 3334, 2974, 2890, 1697, 1045, 879.

^{1}H NMR (500 MHz, $CDCl_3$) δ 7.66 – 7.55 (m, 3H, C(1, 2, 6)*H*), 7.42 – 7.39 (m, 1H, C3*H*), 7.30 (dd, *J* = 17.3 Hz, 11.0 Hz, 1H, C10*H*), 5.70 (d, *J* = 17.3 Hz, 1H, C12H_A), 5.43 (d, *J* = 11.0 Hz, 1H, C12H_B), 4.78 (d, *J* = 4.7 Hz, 2H, C8H_2), 3.51 (t, *J* = 4.7 Hz, 1H, O*H*).

^{13}C NMR (126 MHz, $CDCl_3$) δ 201.6 (C7), 138.9 (C4), 135.4 (C10), 132.9 (C2), 132.5 (C5), 128.1 (C3), 128.0 (C1), 127.7 (C6), 117.7 (C12), 67.1 (C8).

HRMS (ESI): calculated for $C_{10}H_{10}O_2K$ $[M+K]^+$ requires m/z 201.0318, found m/z 201.0315.

Synthesis of 1-(2-allylphenyl)-2-hydroxyethan-1-one 13[19]

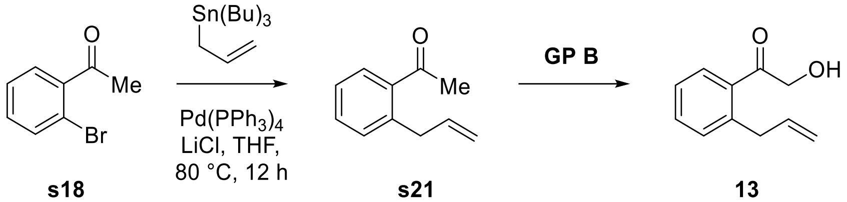

s18 **s21** **13**

13 was synthesized from commercially sourced bromide **s18** according to reported procedures via a 3 step sequence. To a round bottom flask fitted with magnetic stir bar was added 1-(2-bromophenyl)ethanone **s18** (597 mg, 3.0 mmol, 1.0 equiv.), LiCl (636 mg, 15.0 mmol, 5.0 equiv.), allyltributyltin (1.09 g, 3.3 mmol, 1.1 equiv.), and anhydrous THF (30 mL, 0.10 M). The reaction vessel was rigorously deoxygenated with Ar for 10 minutes. $Pd(PPh_3)_4$ (347 mg, 3.0 mmol, 0.10 equiv.) was added and the reaction was heated at reflux for 12 h. The reaction was cooled to r.t. and diluted with H_2O (60 mL). The mixture was extracted EtOAc (3 × 100 mL), and the organic extracts were dried (Na_2SO_4) and concentrated *in vacuo*. The crude material was purified by FCC (10% KF/SiO_2, 100% hexane to 1:9 EtOAc/hexane) to obtain **s21** which was used directly in the next step. Data were in accordance with the literature.[20] According to *General Procedure B*; Ketone **s21** (465 mg, 2.9 mmol, 1.0 equiv.), LDA (4.0 mmol, 1.4 equiv.), chlorotrimethylsilane (0.74 mL, 5.8 mmol, 2.0 equiv.) and *N*-bromosuccinimide (543 mg, 3.05 mmol, 1.05 equiv.) were used. Purification by FCC (50:1 to 20:1 petrol/EtOAc) afforded the bromoketone which was used directly in the next step. Bromoketone (350 mg, 1.46 mmol, 1.0 equiv.), sodium formate (408 mg, 6.0 mmol, 4.0 equiv.) and MeOH (2 mL) were used. Purification by FCC (20:1 to 10:1 petrol/EtOAc) afforded **13** (176 mg, 24% over 3 steps) as a colorless oil.

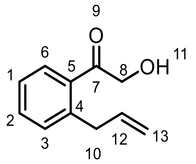

13

IR (film) ν_{max}/cm^{-1}: 3438, 2915, 1686, 1445, 1087, 972.

^{1}H NMR (500 MHz, $CDCl_3$) δ 7.62 – 7.60 (m, 1H, C6*H*), 7.52 (m, 1H, C1*H*), 7.38 – 7.34 (m, 2H, C(2, 3)*H*), 6.04 – 5.96 (m, 1H, C12*H*), 5.07 (dd, *J* = 10.2 Hz, 1.7 Hz, 1H, C13H_A), 5.01 (dd, *J* = 17.2 Hz, 1.7 Hz, 1H, C13H_B), 4.75 (d, *J* = 3.9 Hz, 2H, C8H_2), 3.73 (dt, *J* = 6.4 Hz, 1.6 Hz, 2H, C10H_2), 3.54 (t, *J* = 3.9 Hz, 1H, O*H*).

^{13}C NMR (126 MHz, $CDCl_3$) δ 201.5 (C7), 141.0 (C4), 136.9 (C12), 133.5 (C5), 132.9 (C1), 131.7 (C3), 128.3 (C1), 126.5 (C2), 116.0 (C13), 66.8 (C8), 38.0 (C10).

HRMS (ESI): calculated for $C_{11}H_{13}O_2$ $[M+H]^+$ requires m/z 177.0916, found m/z 177.0911.

3-(Allyloxy)-2-hydroxy-1-phenylpropan-1-one 5k

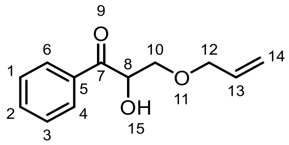

5k

5k was synthesized from commercially sourced chloride **s22** according to *General Procedure F* and *General Procedure D* via a 3 step sequence. *General Procedure F:* 3-Chloropropiophenone **s22** (3.71 g, 22 mmol, 1.0 equiv), chloroform (45 mL), Et_3N (7.24 mL, 52 mmol, 2.4 equiv) were used in the first step. 1-Phenylprop-2-en-1-one **s23** (500 mg, 3.78 mmol, 1.0 equiv), CH_2Cl_2 (15 mL), *p*-TsOH monohydrate (36 mg, 0.19 mmol, 0.05 equiv) and allyl alcohol (285 µL, 4.13 mmol, 1.1 equiv) were used in the second step to give **5k-s24** which was used directly in the next step. *General Procedure D*: Ketone **5k-s24** (380 mg, 2.0 mmol, 1 equiv.), iodine (102 mg, 0.4 mmol, 20 mol%) and DMSO (2 mL) were used. Purification by FCC (10:1 to 3:1 petrol/EtOAc) yielded **5k** (100 mg, 15% over 3 steps) as a yellow oil.

^{1}H NMR (500 MHz, $CDCl_3$): δ 7.96 (d, *J* = 8.0 Hz, 2H, C(4, 6)*H*), 7.65 (t, *J* = 8.0 Hz, 1H, C2*H*), 7.53 (t, *J* = 8.0 Hz, 2H, C(1, 3)*H*), 5.79 – 5.72 (m, 1H, C13*H*), 5.25 – 5.22 (m, 1H, C8*H*), 5.18 – 5.10 (m, 2H, C14H_2), 4.00 – 3.92 (m, 3H, O*H*, C12H_2), 3.83 (dd, *J* = 10.4, 3.2 Hz, 1H, C10H_A), 3.75 (dd, *J* = 10.4, 4.6 Hz, 1H, C10H_B).

^{13}C NMR (126 MHz, $CDCl_3$): δ 199.6 (C7), 134.1 (C13), 134.0 (C5), 134.0 (C2), 128.8 (C(1, 3)), 128.6 (C(4, 6)), 117.3 (C14), 73.7 (C8), 72.6 (C12), 72.5 (C10). Data are consistent with literature.[21]

3-(But-3-en-1-yloxy)-1-phenylpropan-1-one 7k-s24

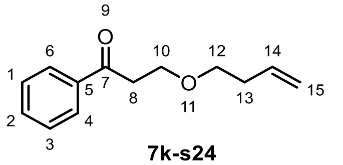

7k-s24

7k-24 was synthesized from commercially sourced chloride **s22** according to *General Procedure F* via 2 step sequence. 3-Chloropropiophenone **s22** (3.71 g, 22 mmol, 1.0 equiv), chloroform (45 mL) and Et_3N (7.24 mL, 52 mmol, 2.4 equiv) were used to give **s23** in the first step. 1-Phenylprop-2-en-1-one **s23** (500 mg, 3.78 mmol, 1.0 equiv), CH_2Cl_2 (15 mL), *p*-TsOH monohydrate (36 mg, 0.19 mmol, 0.05 equiv), and 3-buten-1-ol (354 µL, 4.13 mmol, 1.1 equiv) were used in the second step to give **7k-s24** (758 mg, 98% over 2 steps) as a pale yellow oil.

IR (film) ν_{max}/cm^{-1}: 2866, 1683, 1449, 1110, 1000, 914.

^{1}H NMR (500 MHz, $CDCl_3$) δ 7.99 (d, *J* = 7.7 Hz, 2H, C(4, 6)*H*), 7.58 (t, *J* = 7.7 Hz, 1H, C2*H*), 7.48 (t, *J* = 7.7 Hz, 2H, C(1, 3)*H*), 5.86 – 5.78 (m, 1H, C14*H*), 5.10 (dq, *J* = 17.2 Hz, 1.9 Hz, 1H, C15H_A), 5.04 (dq, *J* = 10.4 Hz, 1.9 Hz, 1H, C15H_B), 3.90 (t, *J* = 6.7 Hz, 2H, C10H_2), 3.55 (t, *J* = 6.8 Hz, 2H, C12H_2), 3.28 (t, *J* = 6.7 Hz, 2H, C8H_2), 2.35 (m, 2H, C13H_2).

^{13}C NMR (126 MHz, $CDCl_3$) δ 198.5 (C7), 137.0 (C5), 135.2 (C14), 133.2 (C2), 128.6 (C1, 3), 128.2 (C4, 6), 116.4 (C15), 70.6 (C12), 66.1 (C10), 38.9 (C8), 34.1 (C13).

HRMS (ESI): calculated for $C_{13}H_{16}O_2Na$ $[M+Na]^+$ requires m/z 227.1048, found m/z 227.1046.

3-(But-3-en-1-yloxy)-2-hydroxy-1-phenylpropan-1-one 7k

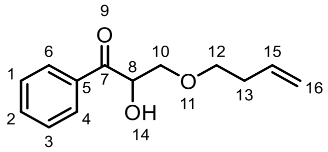

7k

7k was synthesized from **7k-s24** according to *General Procedure D*. Ketone **7k-s24** (612 mg, 3.0 mmol, 1 equiv.), iodine (153 mg, 0.6 mmol, 20 mol%) and DMSO (3 mL) were used. Purification by FCC (10:1 to 3:1 petrol/EtOAc) yielded **7k** (150 mg, 23%) as a yellow oil.

IR (film) ν_{max}/cm^{-1}: 3465, 2863, 1683, 1449, 1112, 1000.

^{1}H NMR (500 MHz, $CDCl_3$) δ 7.96 (d, *J* = 7.7 Hz, 2H, C(4, 6)*H*), 7.64 (t, *J* = 7.7 Hz, 1H, C2*H*), 7.52 (t, *J* = 7.7 Hz, 2H, C(1, 3)*H*), 5.74 – 5.66 (m, 1H, C15*H*), 5.23 – 5.20 (m, 1H, C8*H*), 5.02 – 4.97 (m, 2H, C16H_2), 3.91 (d, *J* = 6.8 Hz, 1H, O*H*), 3.82 (dd, *J* = 10.4 Hz, 3.6 Hz, 1H, C10H_A), 3.75 (dd, *J* = 10.4 Hz, 4.8 Hz, 1H, C10H_B), 3.51 – 3.41 (m, 2H, C12H_2), 2.24 (q, *J* = 6.8 Hz, 2H, C13H_2).

^{13}C NMR (126 MHz, $CDCl_3$) δ 199.7 (C7), 134.9 (C15), 134.1 (C5), 133.9 (C2), 128.8 (C1, 3), 128.6 (C4, 6), 116.4 (C16), 73.7 (C8), 73.4 (C10), 71.1 (C12), 33.9 (C13).

HRMS (ESI): calculated for $C_{13}H_{16}O_3Na$ $[M+Na]^+$ requires m/z 243.0997, found m/z 243.0993.

2-((*tert*-Butyldimethylsilyl)oxy)-*N*-methoxy-*N*-methyl-2-phenylacetamide S27[8]

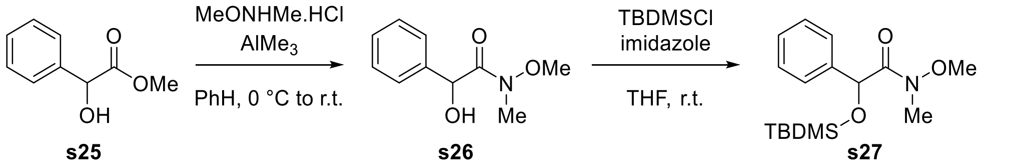

To a 100-mL, three-necked, round-bottomed flask was added *N*,*O*-dimethyl-hydroxylamine hydrochloride (1.95 g, 20 mmol, 2 equiv.) in anhydrous benzene (20 mL). $AlMe_3$ in heptane (1.0 M, 20 mL, 20 mmol, 2 equiv.) was added dropwise at 0 °C under a N_2 atmosphere. The reaction mixture was warmed to r.t. and stirred for 2 h. During this period, methane gas evolution was observed. A solution of the methyl mandelate **s25** (1.66 g, 10 mmol, 1 equiv.) in anhydrous benzene (10 mL) was added to the reaction mixture at 0 °C under a N_2 atmosphere. The reaction mixture was warmed r.t. and stirred for 8 h. The reaction was then quenched with 1 M aq. HCl (20 mL) at 0 °C under a N_2 atmosphere. The resulting mixture was extracted with EtOAc (3 × 20 mL). The combined organic extracts were dried ($MgSO_4$) and concentrated *in vacuo* to give the crude Weinreb amide which was used directly in the next step. To a 50-mL, two-necked, round-bottomed flask was added the crude Weinreb amide **s26** (1.62 g, 8.3 mmol, 1 equiv.) and imidazole (1.13 g, 16.6 mmol, 2 equiv.) in anhydrous THF (20 mL). *tert*-Butyldimethylsilyl (TBS) chloride (1.25 g, 8.3 mmol, 1 equiv) was added at 0 °C. The reaction mixture was warmed to r.t. and stirred for 8 h. The resulting mixture was quenched with H_2O (20 mL) and extracted with EtOAc (3 × 20 mL). The combined organic extracts were dried ($MgSO_4$) and concentrated *in vacuo* to give the crude amide **s27** which was divided and used directly in the next step. Data were in accordance with the literature.[22]

1-Hydroxy-1-phenylhept-6-en-2-one *iso*-5

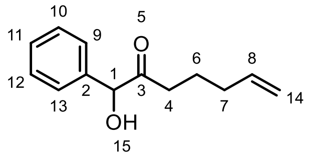

***iso*-5**

Following *General Procedure G* using a 2 step sequence. *Step 1*: Weinreb amide **s27** (309 mg, 1 mmol, 1 equiv.). Et_2O (10 mL) and Grignard reagent (5 mmol, 5 equiv.) were used to give crude ketone **s28a** which was used directly in the next step. Data were in accordance with the literature.[23] *Step 2:* ketone **s28a** (191 mg, 0.6 mmol, 1 equiv.), THF (8.0 mL) and tetrabutylammonium fluoride (1.0 M in THF, 1.8 mL, 3 equiv.) were used to give ***iso*-5** (65 mg, 53% over 2 steps) as a colorless oil.

IR (film) ν_{max}/cm^{-1}: 3456, 2934, 1712, 1454, 1062, 914.

^{1}H NMR (500 MHz, $CDCl_3$) δ 7.42 – 7.32 (m, 5H, Ph*H*), 5.71 – 5.62 (m, 1H, C8*H*), 5.10 (d, *J* = 4.4 Hz, 1H, C1*H*), 4.95 – 4.90 (m, 2H, C14*H*$_2$), 4.36 (d, *J* = 4.4 Hz, 1H, O*H*), 2.44 – 2.30 (m, 2H, C4*H*$_2$), 2.02 – 1.89 (m, 2H, C7*H*$_2$), 1.72 – 1.56 (m, 2H, C6*H*$_2$).

^{13}C NMR (126 MHz, $CDCl_3$) δ 209.4 (C3), 138.1 (C2), 137.4 (C8), 129.0 (C(9, 13), 128.7 (C11), 127.4 (C10, C12), 115.5 (C14), 79.8 (C1), 37.0 (C4), 33.3 (C7), 24.1 (C6).

HRMS (ESI): calculated for $C_{13}H_{16}O_2Na$ $[M+Na]^+$ requires m/z 227.1048, found m/z 227.1043.

(*E*)-1-Hydroxy-1-phenyloct-6-en-2-one s30

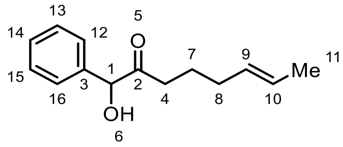

s30

Following *General Procedure G* using a 2 step sequence. *Step 1*: Weinreb amide **s27** (309 mg, 1 mmol, 1 equiv.). Et_2O (10 mL), and Grignard reagent (~1.0 M, 5 mL, 5 mmol, 5 equiv.) were used to give ketone **s28b**, which was used directly in the next step. *Step 2:* ketone **s28b** (333 mg, 1 mmol, 1 equiv.), THF (8 mL), and tetrabutylammonium fluoride (1.0 M in THF, 3.0 mL, 3 equiv.) were used to give **s30** (145 mg, 66% over 2 steps, 96:4 *trans*:*cis*) as a colorless oil.

IR (film) ν_{max}/cm^{-1}: 3457, 2931, 1712, 1454, 1061, 966.

^{1}H NMR (500 MHz, $CDCl_3$) δ 7.42 – 7.32 (m, 5H, Ph*H*), 5.35 – 5.22 (m, 2H, C(9, 10)*H*), 5.09 (d, *J* = 3.9 Hz, 1H, C1*H*), 4.37 (d, *J* = 4.3 Hz, 1H, O*H*), 2.42 – 2.29 (m, 2H, C4*H*$_2$), 1.93 – 1.81 (m, 2H, C8*H*$_2$), 1.67 – 1.53 (m, 2H, C7*H*$_2$), 1.61 (d, *J* = 5.9 Hz, 3H, C11*H*$_3$).

^{13}C NMR (126 MHz, $CDCl_3$) δ 209.6 (C2), 138.2 (C3), 129.9 (C9), 129.0 (C(12, 16)), 128.7 (C10), 127.4 (C(13, 15)), 126.1 (C14), 79.7 (C1), 37.0 (C4), 31.6 (C8), 23.4 (C7), 17.9 (C11).

HRMS (ESI): calculated for $C_{14}H_{18}O_2Na$ $[M+Na]^+$ requires m/z 241.1204, found m/z 241.1197.

1-Oxo-1-phenyloct-7-en-2-yl acetate Ac-7a

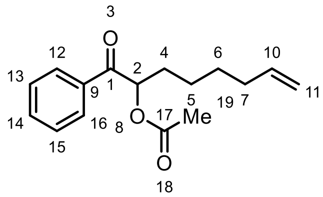

Ac-7a

Following the literature procedures,[24] to a solution of **7a** (109 mg, 0.5 mmol) and pyridine (2.0 mL, 2.5 mmol, 5.0 equiv.) in CH_2Cl_2 (0.5 mL) was added acetyl chloride (59 mg, 0.75 mmol) and DMAP (61 mg, 0.5 mmol) at 0 ºC. The solution was waremed to r.t. and stirred for 1.5 h. Saturated aq. $NaHCO_3$ was added to the mixture, which was then extracted with EtOAc. The organic extracts were washed with H_2O and brine, dried ($MgSO_4$) and concentrated *in vacuo*. The residue was purified by FCC (20:1 to 5:1 petrol/Et_2O) to give **Ac-7a** (125 mg, 96%) as colorless oil.

IR (film) ν_{max}/cm^{-1}: 2931, 2861, 1740, 1697, 1229, 912.

^{1}H NMR (500 MHz, $CDCl_3$) δ 7.96 – 7.95 (m, 2H, C(12, 16)*H*), 7.62 – 7.59 (m, 1H, C14*H*), 7.51 – 7.48 (m, 2H, C(13, 15)*H*), 5.88 (dd, *J* = 8.5 Hz, 4.2 Hz, 1H, C2*H*), 5.83 – 5.74 (m, 1H, C10*H*), 5.02 – 4.94 (m, 2H, C11H_2), 2.18 (s, 3H, C19H_3), 2.02 (m, 2H, C7H_2), 1.94 – 1.81 (m, 2H, C4H_2), 1.53 – 1.39 (m, 4H, C5H_2, C6H_2).

^{13}C NMR (126 MHz, $CDCl_3$) δ 196.6 (C1), 170.7 (C17), 138.4 (C10), 134.8 (C9), 133.5 (C14), 128.8 (C(13, 15)), 128.4 (C(12, 16)), 114.7 (C11), 75.3 (C2), 33.4 (C7), 31.2 (C4), 28.4 (C6), 25.0 (C5), 20.7 (C19).

HRMS (ESI): calculated for $C_{16}H_{20}O_3Na$ $[M+Na]^+$ requires m/z 283.1310, found m/z 283.1311.

Characterization of products

Regiochemical assignments

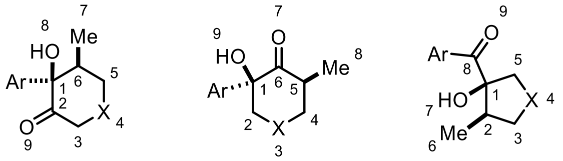

6 ***iso*-6** **6'**

Assignment of regioisomers **6** versus ***iso*-6** was made by using key HMBC correlations between the methyl group and either the alcohol bearing carbon (C1) in **6** or the ketone (C6) in ***iso*-6**. For the latter, key correlations were also observed between C2H_2 and the *ipso*-carbon of the Ar group. For these systems, no HMBC correlation was observed between the *ortho*-C-*H* of the arene and the ketone carbonyl, whereas a correlation between the *ortho*-C-H of the arene and C1 was observed in both cases. These observations allowed us to differentiate **6** and ***iso*-6** from 5-*exo* product **6'**. An additional distinguishing feature is that the ^{13}C NMR resonance of the ketone carbon is distinct for endocyclic (~215 ppm; as in **6** and ***iso*-6**) vs exocyclic (~205 ppm; as in **6'** and **8**) products.

(*trans*)-2-Hydroxy-6-methyl-2-phenylcyclohexan-1-one *iso*-6a

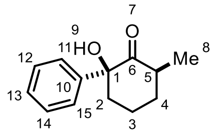

Synthesized from **5a** (20.4 mg) according to *General Procedure I* (90 °C, 24 h). Analysis of the ^{1}H NMR spectrum of the crude mixture gave a >20:1 d.r. and 1:10 ratio of **6a:*iso*-6a**. **6a:*iso*-6a** (1:10 ratio, >20:1 d.r., 17.4 mg, 85%) were isolated as a colorless oil.

IR (film) ν_{max}/cm^{-1}: 3469, 2935, 1706, 1449, 1120, 988.

^{1}H NMR (500 MHz, $CDCl_3$) δ 7.42 – 7.38 (m, 2H, C(12, 14)*H*), 7.32 – 7.29 (m, 3H, C(11,13, 15)*H*), 4.68 (s, 1H, O*H*), 3.09 – 3.04 (m, 1H, C2H_A), 2.62 – 2.54 (m, 1H, C5*H*), 2.10 – 2.05 (m, 1H, C3H_A), 1.90 – 1.81 (m, 3H, C2H_B, C4H_2), 1.52 – 1.44 (m, 1H, C3H_B), 1.10 (d, *J* = 6.5 Hz, 3H, C8*H*).

^{13}C NMR (126 MHz, $CDCl_3$) δ 214.4 (C6), 140.3 (C10), 129.2 (C(12, 14)), 128.2 (C13), 126.4 (C(11, 15)), 80.0 (C1), 42.1 (C5), 39.2 (C2), 37.3 (C4), 22.6 (C3), 14.3 (C8).

HRMS (ESI): calculated for $C_{13}H_{16}O_2Na$ $[M+Na]^+$ requires m/z 227.1048, found m/z 227.1046.

$[\alpha]_D^{25}$ = +22.2 (c 1.0, CH_2Cl_2).

Chiral SFC: YMC Chiral ART Cellulose-SC column (25 cm), CO_2:*i*-PrOH 95:5, 2.0 mL/min, 168 bar, 40 °C. Retention times: 8.4 mins (minor), 9.0 mins (major), e.r. = 67:33. The racemate was prepared using *rac*-BINAP.

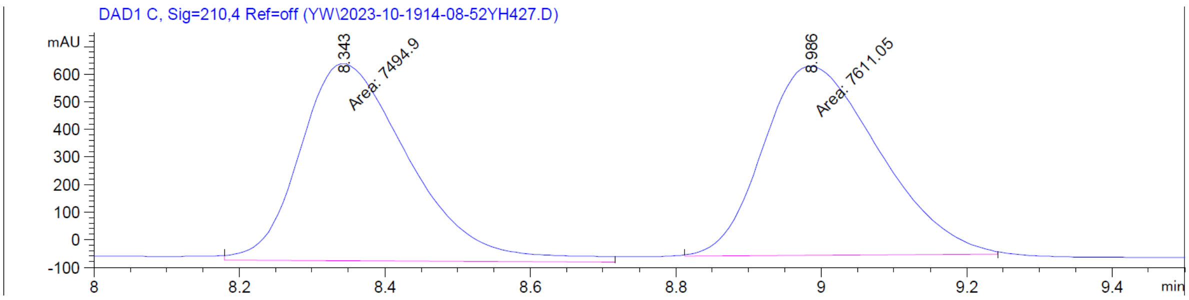

Signal 3: DAD1 C, Sig=210,4 Ref=off

Peak #	RetTime [min]	Type	Width [min]	Area [mAU*s]	Height [mAU]	Area %
1	8.343	MM	0.1746	7494.89893	715.41449	49.6156
2	8.986	MM	0.1847	7611.04736	686.93781	50.3844
Totals :				1.51059e4	1402.35229	

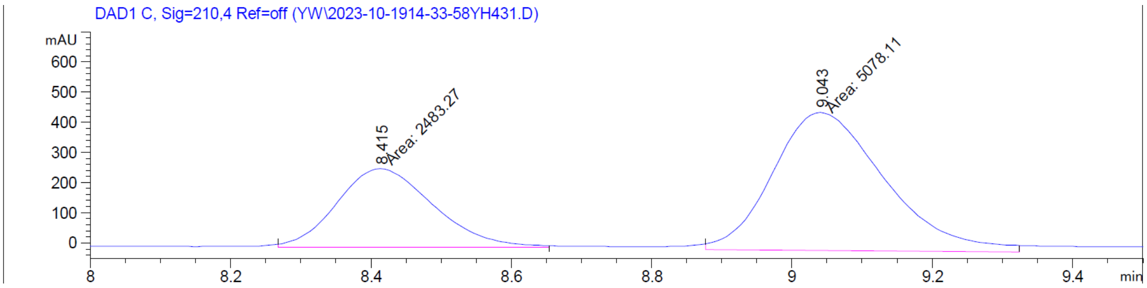

Signal 3: DAD1 C, Sig=210,4 Ref=off

Peak #	RetTime [min]	Type	Width [min]	Area [mAU*s]	Height [mAU]	Area %
1	8.415	MM	0.1593	2483.27100	259.77948	32.8415
2	9.043	MM	0.1852	5078.11475	457.01071	67.1585
Totals :				7561.38574	716.79019	

(*trans*)-2-Hydroxy-6-methyl-2-(*p*-tolyl)cyclohexan-1-one *iso*-6b

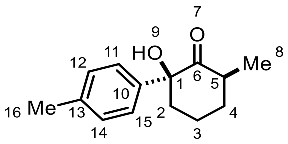

***iso*-6b**

Synthesized from **5b** (21.8 mg) according to *General Procedure I* (60 °C, 72 h). **6b:*iso*-6b** were formed as 1:1 mixture, and then resubmitted to the standard conditions (90 °C, 24 h). Analysis of the ^{1}H NMR spectrum of the crude mixture gave a >20:1 d.r. and 1:10 ratio of **6b:*iso*-6b**. **6b:*iso*-6b** (1:10 ratio, >20:1 d.r.,18.0 mg, 84%) were isolated as a colorless oil.

IR (film) ν_{max}/cm^{-1}: 3419, 2934, 1705, 1452, 1079, 988.

^{1}H NMR (400 MHz, CDCl$_3$) δ 7.23 – 7.17 (m, 4H, Ar*H*), 4.62 (s, 1H, O*H*), 3.05 – 3.01 (m, 1H, C2H_A), 2.65 – 2.55 (m, 1H, C5*H*), 2.36 (s, 3H, C16H_3), 2.09 – 2.01 (m, 3H, C3H_A), 1.89 – 1.80 (m, 3H, C2H_B, C4H_2), 1.52 – 1.42 (m, 1H, C3H_B), 1.10 (d, *J* = 6.6 Hz, 3H, C8H_3).

^{13}C NMR (101 MHz, $CDCl_3$) δ 214.5 (C6), 138.0 (C10), 137.5 (C13), 129.8 (C(12, 14)), 126.3 (C(11, 15)), 79.8 (C1), 42.1 (C5), 39.2 (C2), 37.3 (C4), 22.6 (C3), 21.1 (C16), 14.3 (C8).

HRMS (ESI): calculated for $C_{14}H_{18}O_2Na$ $[M+Na]^+$ requires m/z 241.1204, found m/z 241.1200.

The relative stereochemistry of ***iso-6b*** *was assigned by NOE analysis; the relative stereochemical assignments of related products is based upon this.*

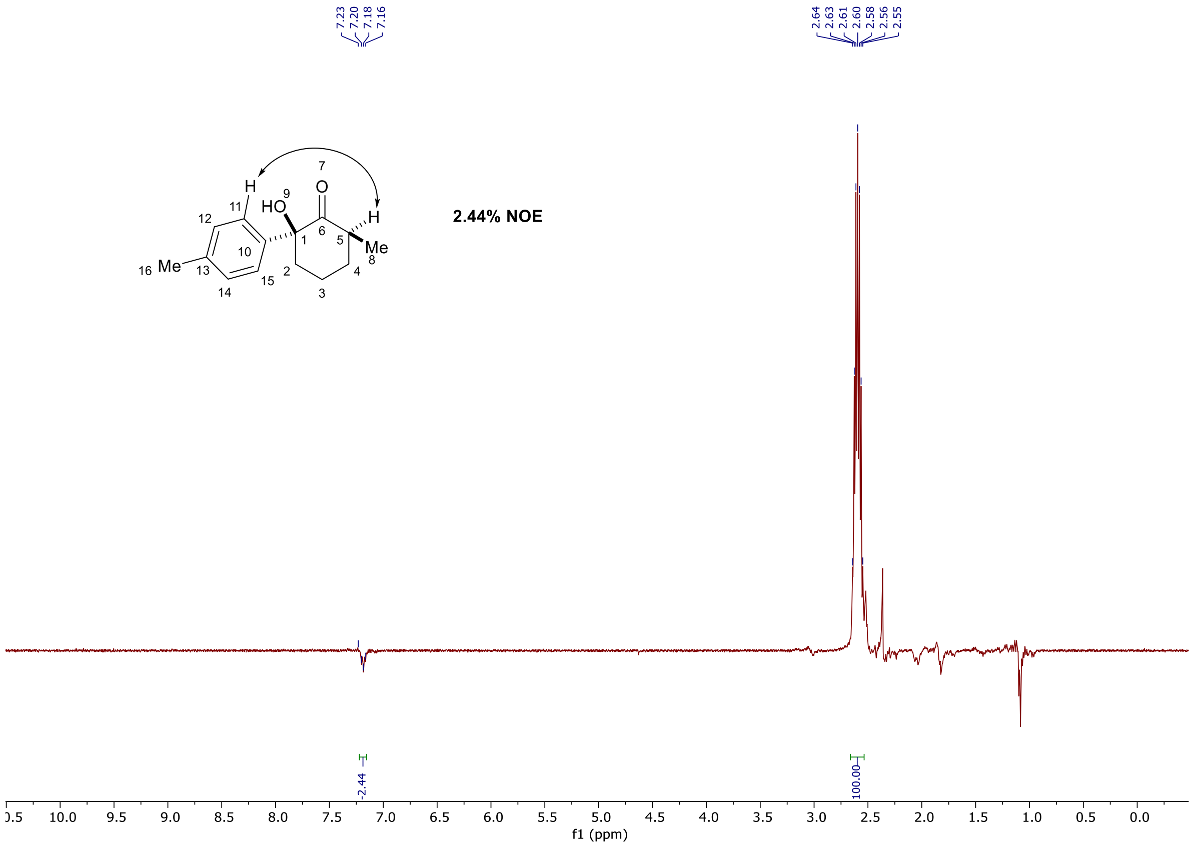

1D NOE spectrum: irradiation of C5*H* proton (2.55 – 2.64 ppm, m) showed a correlation to C(11, 15)*H* (7.16 – 7.23 ppm, m).

(*trans*)-2-(4-Chlorophenyl)-2-hydroxy-6-methylcyclohexan-1-one *iso*-6c and (*trans)*-2-(4-Chlorophenyl)-2-hydroxy-3-methylcyclohexan-1-one 6c

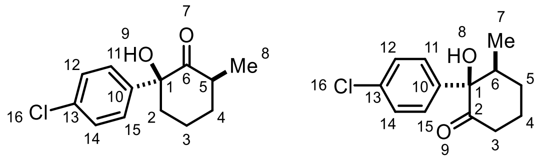

***iso*-6c** **6c**

Synthesized from **5c** (23.9 mg) according to *General Procedure I* (60 °C for 72 h), the products were isolated as 1:1 mixture and then resubmitted to the standard conditions (90 °C, 24 h). Analysis of the ^{1}H NMR spectrum of the crude mixture gave a >20:1 d.r. and 1:4.5 ratio of **6c:*iso*-6c**. **6c:*iso*-6c** (1:4.5 ratio, >20:1 d.r., 18.0 mg, 77%) were isolated as a colorless oil.

IR (film) ν_{max}/cm^{-1}: 3466, 2933, 1709, 1493, 1094, 990.

^{1}H NMR (400 MHz, $CDCl_3$) δ 7.40 – 7.36 (m, 2H, *iso*-6cC(12, 14)*H*), 7.25 – 7.22 (m, 2H, *iso*-6cC(11, 15)*H*), 4.69 (s, 1H, *iso*-6cO*H*), 4.16 (s, 6cO*H*), 3.05 – 3.03 (m, 6cC6*H*), 3.02 – 2.96 (m, 1H, *iso*-6cC2H_A), 2.60 – 2.41 (m, 1H, *iso*-6cC5*H*), 2.12 – 2.05 (m, 1H, *iso*-6cC3H_A), 2.00 – 1.97 (m, 2H, 6cC5H_A, 6cC4H_A), 1.91 – 1.70 (m, 3H, *iso*-6cC2H_B, *iso*-6cC4H_2), 1.54 – 1.43 (qd, J = 12.8 Hz, 4.2 Hz, 1H, *iso*-6cC3H_B), 1.10 (d, J = 6.6 Hz, 3H, *iso*-6cC8H_3), 1.02 (d, J = 7.0 Hz, 6cC7H_3).

^{13}C NMR (101 MHz, $CDCl_3$) δ 213.9 (*iso*-6cC6), 212.6 (6cC2), 139.6 (6cC10), 138.9 (*iso*-6cC10), 134.2 (*iso*-6cC13), 134.0 (6cC13), 129.3 (*iso*-6cC(12, 14)), 129.1 (6cC(12, 14)), 128.0 (6cC(11, 15)), 127.9 (*iso*-6cC(11, 15)), 82.3 (6cC1), 79.4 (*iso*-6cC1), 42.2 (*iso*-6cC5), 39.4 (6cC6), 39.3 (*iso*-6cC2), 38.3 (6cC3), 37.3 (*iso*-6cC4), 28.8 (6cC5), 22.9 (6cC4), 22.6 (*iso*-6cC3), 14.2 (*iso*-6cC7), 13.5 (6cC8).

HRMS (ESI): calculated for $C_{13}H_{15}Cl^{35}O_2Na$ $[M+Na]^+$ requires m/z 261.0658, found m/z 261.0638.

***(trans)*-2-(2-Fluorophenyl)-2-hydroxy-6-methylcyclohexan-1-one *iso*-6d and *(trans)*-2-(2-Fluorophenyl)-2-hydroxy-6-methylcyclohexan-1-one *iso*-6d**

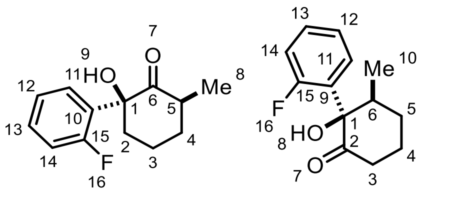

Synthesized from **5d** (22.3 mg) according to *General Procedure I* (90 °C, 24 h). Analysis of the ^{1}H NMR spectrum of the crude mixture gave a >20:1 d.r. and 1:3 ratio of **6d:*iso*-6d**. ***iso*-6d** (>20:1 d.r., 13.0 mg, 59%) was isolated as a colorless oil. Continued elution provided **6d** (>20:1 d.r., 14.0 mg, 18%) as a colorless solid.

Data for ***iso*-6d**:

IR (film) ν_{max}/cm^{-1}: 3457, 2936, 1708, 1451, 1117, 981.

^{1}H NMR (500 MHz, $CDCl_3$) δ 7.62 – 7.58 (m, 1H, C13*H*), 7.39 – 7.34 (m, 1H, C14*H*), 7.25 – 7.23 (m, 1H, C12*H*), 7.10 – 7.06 (m, 1H, C11*H*), 4.80 (s, 1H, O*H*), 3.10 – 3.04 (m, 1H, C2H_A), 2.68 – 2.61 (m, 1H, C5*H*), 2.09 – 2.05 (m, 1H, C3H_A), 1.82 – 1.67 (m, 3H, C2H_B, C4H_2), 1.49 – 1.40 (m, 1H, C3H_B), 1.15 (d, J = 6.4 Hz, 3H, C8*H*).

^{19}F NMR (470 MHz, $CDCl_3$) δ –112.0.

^{13}C NMR (126 MHz, $CDCl_3$) δ 213.4 (C6), 160.8 (d, J = 248.3 Hz, C15), 130.4 (d, J = 8.8 Hz, C14), 128.0 (d, J = 4.2 Hz, C13), 127.7 (d, J = 13.0 Hz, C10), 124.6 (d, J = 3.3 Hz, C12), 116.6 (d, J = 22.7 Hz, C11), 77.9 (C1), 41.8 (d, J = 1.9 Hz, C5), 40.6 (C2), 38.1 (C3), 22.4 (C4), 14.4 (C8).

HRMS (ESI): calculated for $C_{13}H_{15}FO_2Na$ $[M+Na]^+$ requires m/z 245.0954, found m/z 245.0946.

Data for **6d**:

m.p. = 116–118 °C.

IR (film) ν_{max}/cm^{-1}: 3370, 2974, 1701, 1489, 1047, 986.

^{1}H NMR (500 MHz, $CDCl_3$) δ 7.64 – 7.51 (m, 1H, C13*H*), 7.37 – 7.33 (m, 1H, C14*H*), 7.24 – 7.21 (m, 1H, C12*H*), 7.10 – 7.06 (m, 1H, C11*H*), 4.05 (s, 1H, O*H*), 3.10 – 3.05 (m, 1H, C6*H*), 2.65 – 2.51 (m, 2H, C3H_2), 2.02 – 1.87 (m, 3H, C5H_A, C4H_2), 1.76 – 1.67 (m, 1H, C5H_B), 0.97 (d, J = 7.1 Hz, 3H, C10*H*).

^{19}F NMR (470 MHz, $CDCl_3$) δ –110.5.

^{13}C NMR (126 MHz, $CDCl_3$) δ 210.8 (C2), 160.4 (d, J = 247.2 Hz, C15), 130.0 (d, J = 9.0 Hz, C14), 128.7 (d, J = 12.7 Hz, C9), 127.7 (d, J = 4.4 Hz, C13), 124.4 (d, J = 3.2 Hz, C12), 116.4 (d, J = 23.0 Hz, C11), 80.2 (d, J = 2.1 Hz, C1), 39.7 (C6), 37.8 (df, J = 2.1 Hz, C3), 28.6ff (C5), 23.4 (C4), 12.8 (C10).

HRMS (ESI): calculated for $C_{13}H_{15}FO_2Na$ $[M+Na]^+$ requires m/z 245.0954, found m/z 245.0948.

***(trans)*-2-Hydroxy-2-(3-methoxyphenyl)-6-methylcyclohexan-1-one *iso*-6e**

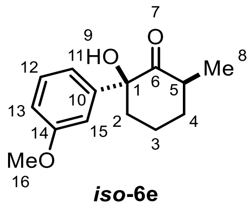

***iso*-6e**

Synthesized from **5e** (23.5 mg) according to *General Procedure I* (90 °C, 24 h). Analysis of the ^{1}H NMR spectrum of the crude mixture gave a >20:1 d.r. and 1:10 ratio of **6e:*iso*-6e**. **6e:*iso*-6e** (1:10 ratio, >20:1 d.r., 14.5 mg, 62%) were isolated as a colorless oil.

IR (film) ν_{max}/cm^{-1}: 3458, 2936, 1708, 1452, 1048, 991.

^{1}H NMR (500 MHz, $CDCl_3$) δ 7.34 – 7.31 (m, 1H, C12*H*), 6.88 – 6.84 (m, 3H, C(11, 13, 15)*H*), 4.66 (s, 1H, O*H*), 3.82 (C16*H*), 3.05 – 2.98 (m, 1H, C2H_A), 2.64 – 2.56 (m, 1H, C5*H*), 2.09 – 2.04 (m, 1H, C3H_A), 1.89 – 1.81 (m, 3H, C2H_B, C4H_2), 1.51 – 1.43 (m, 1H, C3H_B), 1.10 (d, J = 6.5 Hz, 3H, C8*H*).

^{13}C NMR (126 MHz, $CDCl_3$) δ 214.2 (C6), 160.2 (C14), 141.9 (C10), 130.2 (C12), 118.7 (C11), 113.3 (C15), 112.6 (C13), 79.9 (C1), 55.3 (C16), 42.1 (C5), 39.2 (C2), 37.2 (C4), 22.6 (C3), 14.3 (C8).

HRMS (ESI): calculated for $C_{14}H_{18}O_3Na$ $[M+Na]^+$ requires m/z 257.1154, found m/z 257.1161.

***(trans)*-2-Hydroxy-3-methyl-2-(3-(trifluoromethyl)phenyl)cyclohexan-1-one 6f**

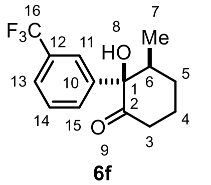

6f

Synthesized from **5f** (27.3 mg) according to *General Procedure I* (60 °C, 72 h). Analysis of the ^{1}H NMR spectrum of the crude mixture gave a >20:1 d.r. and >20:1 ratio of **6f:*iso*-6f**. **6f** (>20:1 d.r., 22.0 mg, 81%) was isolated as a colorless solid. **6f:*iso*-6f** formed in a 2:1 ratio when the reaction was run at 90 °C for 24 h.

m.p. = 46–48 °C.

IR (film) ν_{max}/cm^{-1}: 3457, 2938, 1713, 1327, 1120, 978.

^{1}H NMR (500 MHz, $CDCl_3$) δ 7.61 – 7.59 (m, 2H, C(11, 13)*H*), 7.54 – 7.47 (m, 2H, C(14, 15)*H*), 4.17 (s, 1H, O*H*), 3.09 – 3.03 (m, 1H, C6*H*), 2.66 – 2.61 (m, 1H, C3H_A), 2.48 – 2.42 (m, 1H, C3H_B), 2.07 – 1.89 (m, 3H, C5H_A, C4H_2), 1.79 – 1.74 (m, 1H, C5H_B), 1.03 (d, J = 7.0 Hz, 3H, C7H_3).

^{19}**F NMR** (470 MHz, $CDCl_3$) δ –62.6.

^{13}**C NMR** (126 MHz, $CDCl_3$) δ 212.1 (C2), 142.0 (C10), 131.2 (q, J = 32.4 Hz, C12), 130.1 (C14), 129.4 (C15), 125.0 (q, J = 3.7 Hz, C11), 124.0 (q, J = 271.3 Hz, C16), 123.3 (q, J = 3.8 Hz, C13), 82.4 (C1), 39.6 (C6), 38.3 (C3), 28.8 (C5), 23.1 (C4), 13.5 (C7).

HRMS (ESI): calculated for $C_{14}H_{15}F_3O_2Na$ $[M+Na]^+$ requires m/z 295.0922, found m/z 295.0911.

***(trans)*-2-Hydroxy-6-methyl-2-(naphthalen-2-yl)cyclohexan-1-one *iso*-6g**

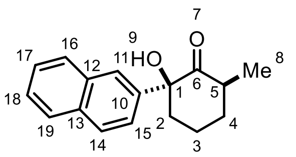

***iso*-6g**

Synthesized from **5g** (25.5 mg) according to *General Procedure I* (90 °C, 24 h). Analysis of the ^{1}H NMR spectrum of the crude mixture gave a >20:1 d.r. and 1:9 ratio of **6g:*iso*-6g**. **6g:*iso*-6g** (1:9 ratio, >20:1 d.r., 16.0 mg, 63%) were isolated as a colorless solid.

IR (film) ν_{max}/cm^{-1}: 3349, 2973, 1713, 1452, 1046, 879.

^{1}**H NMR** (500 MHz, $CDCl_3$) δ 7.89 – 7.84 (m, 4H, Ar*H*), 7.55 – 7.51 (m, 2H, Ar*H*), 7.31 (dd, J = 8.6, 1.9 Hzm C15*H*), 4.78 (s, 1H, O*H*), 3.82 (C16*H*), 3.24 – 3.17 (m, 1H, C2H_A), 2.63 – 2.58 (m, 1H, C5*H*), 2.10 – 2.07 (m, 1H, C3H_A), 1.97 – 1.91 (m, 3H, C2H_B, C4H_2), 1.56 – 1.48 (m, 1H, C3H_B), 1.12 (d, J = 6.5 Hz, 3H, C8*H*).

^{13}**C NMR** (126 MHz, $CDCl_3$) δ 214.4 (C6), 137.6 (C10), 133.4 (C12), 132.9 (C13), 129.2 (ArC), 128.3 (ArC), 127.6 (ArC), 126.6 (ArC), 126.5 (ArC), 125.5 (ArC), 124.2 (C15), 80.1 (C1), 42.3 (C5), 39.4 (C2), 37.4 (C4), 22.8 (C3), 14.3 (C8).

HRMS (ESI): calculated for $C_{17}H_{18}O_2Na$ $[M+Na]^+$ requires m/z 277.1204, found m/z 277.1198.

***(trans)*-2-(Furan-2-yl)-2-hydroxy-3-methylcyclohexan-1-one 6h and *(trans)*-2-(Furan-2-yl)-2-hydroxy-6-methylcyclohexan-1-one *iso*-6h**

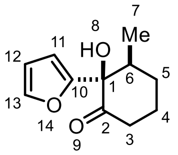

6h

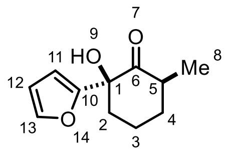

***iso*-6h**

Synthesized from **5h** (19.5 mg) according to *General Procedure I* (90 °C, 24 h). Analysis of the ^{1}H NMR spectrum of the crude mixture gave a 5:1 d.r. and 4:1 ratio of **6h:*iso*-6h**. **6h:*iso*-6h** (4:1 ratio, 5:1 d.r., 12.0 mg, 62%) were isolated a colorless oil.

IR (film) ν_{max}/cm^{-1}: 3448, 2950, 1715, 1462, 1058, 946.

^{1}**H NMR** (500 MHz, $CDCl_3$) δ 7.62 – 7.62 (m, 0.2H, 6h minorC13*H*), 7.40 – 7.39 (m, 1.5H, 6h majorC13*H*, 6h minorC11*H*, *iso*-6hC13*H*), 6.59 (dd, J = 3.6 Hz, 1.7 Hz, 0.2H, 6h minorC12*H*), 6.41 – 6.37 (m, 2.6H, 6h majorC(11, 12)*H*, *iso*-6hC(11, 12)*H*), 4.59 (s, 0.3H, *iso*-6hO*H*), 4.32 (s, 1H, 6h majorO*H*), 3.93 (s, 0.2H, 6h minorO*H*), 3.04 – 2.98 (m, 1H, 6h majorC6*H*), 2.87 – 2.83 (m, 0.3H, *iso*-6hC2H_A), 2.66 – 2.56 (m, 0.6H, 6h minorC6*H*, *iso*-6hC5*H*), 2.56 – 2.52 (m, 2H, 6h majorC3H_2), 2.17 – 2.06 (m, 1.5H, 6h majorC5H_A), 2.03 – 1.80

(m, 4H), 1.71 – 1.68 (m, 1H, 6h majorC5H_B), 1.45 (qd, J = 13.0 Hz, 3.7 Hz, 0.4H, *iso*-6hC3H_B), 1.12 (d, J = 6.5 Hz, 1.1H, *iso*-6hC8H_3), 1.01 (d, J = 7.1 Hz, 3H, 6h majorC7H_3), 0.87 (d, J = 6.7 Hz, 3H, 6h minorC7H_3).

^{13}C NMR (126 MHz, $CDCl_3$) δ 211.1 (*iso*-6hC6), 210.0 (6h majorC2), 192.8 (6h minorC2), 154.7 (6h majorC10), 154.3 (*iso*-6h C10), 150.6 (6h minorC10), 146.4 (6h minorC13), 142.7 (*iso*-6hC13), 142.4 (6h majorC13), 119.9 (6h minorC12), 112.3 (6h minorC11), 110.7 (*iso*-6hC12), 110.5 (6h majorC12), 107.7 (*iso*-6hC11), 107.5 (6h majorC11), 86.8 (6h minorC1), 79.0 (6h majorC1), 76.4 (*iso*-6hC1), 44.7 (6h minorC6), 42.0 (*iso*-6hC5), 40.1 (6h majorC6), 39.9 (*iso*-6hC2), 39.5 (6h minorC3), 38.2 (6h majorC3), 36.8 (*iso*-6hC4), 33.2 (6h minorC5), 29.0 (6h majorC5), 23.5 (6h minorC4), 22.4 (*iso*-6hC3), 22.3 (6h majorC4), 14.4 (*iso*-6hC8), 13.1 (6h majorC7), 12.1 (6h minorC7).

HRMS (ESI): calculated for $C_{11}H_{14}O_3Na$ $[M+Na]^+$ requires m/z 217.41 found m/z 217.0828.

***(trans)*-2-Hydroxy-3-methyl-2-(1-methyl-1*H*-indol-2-yl)cyclohexan-1-one 6i**

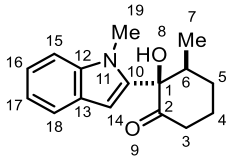

6i

Synthesized from **5i** (25.8 mg) according to *General Procedure I* (70 °C, 72 h; substrate decomposition occurred when the reaction was run at 90 °C). Analysis of the ^{1}H NMR spectrum of the crude mixture gave a >20:1 d.r. and >20:1 ratio of **6i:*iso*-6i**. **6i** (>20:1 d.r., 9.0 mg, 35%) was isolated as a pale yellow oil.

IR (film) ν_{max}/cm^{-1}: 3454, 2953, 1633, 1460, 1144, 994.

^{1}H NMR (500 MHz, $CDCl_3$) δ 7.73 (d, J = 8.1 Hz, 1H, C15H), 7.45 – 7.42 (m, 2H, C(16, 18)H), 7.41 (s, 1H, C14H), 7.21 – 7.18 (m, 1H, C17H), 4.17 (s, 1H, OH), 4.10 (s, 3H, C19H_3), 2.77 – 2.71 (m, 1H, C3H_A), 2.70 – 2.62 (m, 1H, C6H), 2.15 – 1.95 (m, 4H, C3H_B, C5H_A, C4H_2), 1.73 – 1.64 (m, 1H, C5H_B), 0.91 (d, J = 6.5 Hz, 3H, C7H_3).

^{13}C NMR (126 MHz, $CDCl_3$) δ 198.1 (C2), 139.7 (C10), 130.7 (C12), 126.3 (C16), 125.7 (C13), 123.1 (C15), 121.0 (C17), 112.2 (C14), 110.5 (C18), 87.8 (C1), 47.4 (C6), 41.6 (C3), 33.4 (C5), 32.9 (C19), 23.7 (C4), 12.2 (C7).

HRMS (ESI): calculated for $C_{16}H_{20}NO_2$ $[M+H]^+$ requires m/z 258.1494, found m/z 258.1496.

(*trans*)-2-(3-(Cyclopropylmethoxy)-4-(difluoromethoxy)phenyl)-2-hydroxy-6-methylcyclohexan-1-one *iso*-6j and (*trans*)-2-(3-(Cyclopropylmethoxy)-4-(difluoromethoxy)phenyl)-2-hydroxy-3-methylcyclohexan-1-one 6j

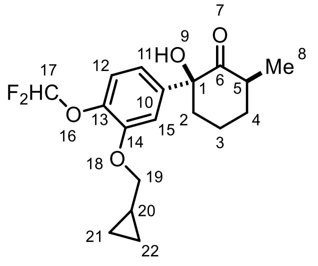

***iso*-6j**

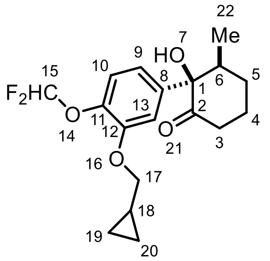

6j

Synthesized from **5j** (34.1 mg) according to *General Procedure I* (90 °C, 24 h). Analysis of the ^{1}H NMR spectrum of the crude mixture gave a >20:1 d.r. and 1:1 ratio of **6j:*iso*-6j**. **6j:*iso*-6j** (1:1 ratio, >20:1 d.r., 24.0 mg, 71%) were isolated as a colorless oil.

IR (film) ν_{max}/cm^{-1}: 3462, 2937, 1709, 1509, 1112, 1008.

^{1}H NMR (500 MHz, $CDCl_3$) δ 7.18 (m, 2H, *iso*-6jC12*H* and 6jC10*H*), 6.90 (m, 2H, *iso*-6jC11*H* and 6jC9*H*), 6.84 (m, 2H, *iso*-6jC15*H* and 6jC13*H*), 6.64 (m, 2H, *iso*-6jC17*H* and 6jC15*H*), 4.68 (s, 1H, *iso*-6jO*H*), 4.19 (s, 1H, 6jO*H*), 3.89 – 3.83 (m, 4H, *iso*-6j C19*H* and 6jC17*H*), 3.06 – 3.01 (m, 1H, 6jC6*H*), 2.99 – 2.95 (m, 1H, *iso*-6jC2H_A), 2.61 – 2.47 (m, 3H, *iso*-6jC5*H*, 6jC3H_2), 2.11 – 1.94 (m, 3H, *iso*-6jC3H_A, 6jC5H_A, 6jC4H_A), 1.92 – 1.81 (m, 4H, 6jC4H_B, *iso*-6jC2H_B, *iso*-6jC4H_2), 1.75 – 1.70 (m, 1H, 6jC5H_B), 1.52 – 1.44 (m, 1H, *iso*-6jC3H_B), 1.32 – 1.24 (m, 2H, *iso*-6jC20*H*, 6jC18*H*), 1.10 (d, *J* = 6.4 Hz, 3H, *iso*-6jC8H_3), 1.02 (d, *J* = 7.0 Hz, 3H, 6jC22H_3), 0.68 – 0.64 (m, 4H, *iso*-6jC21H_2, 6jC19H_2), 0.38 – 0.35 (m, 4H, *iso*-6jC22H_2, 6jC20H_2).

^{19}F NMR (470 MHz, $CDCl_3$) δ –81.5, –81.6, –81.7, –81.7.

^{13}C NMR (126 MHz, $CDCl_3$) δ 214.0 (*iso*-6jC6), 212.8 (6jC2), 151.0 (*iso*-6jC14), 150.8 (6jC12), 140.3 (t, *J* = 3.1 Hz, *iso*-6jC13), 140.2 (t, *J* = 3.1 Hz, 6jC11), 139.6 (*iso*-6jC10), 138.9 (6jC8), 123.0 (*iso*-6jC11), 122.8 (6jC9), 119.2 (6jC10), 119.1 (*iso*-6jC12), 116.12 (t, *J* = 259.8 Hz, *iso*-6jC17 or 6jC15f), 116.08 (t, *J* = 260.0 Hz, *iso*-6jC17 or 6jC15) 113.0 (6jC13), 112.9 (*iso*-6jC15), 82.4 (6jC1), 79.6 (*iso*-6jC1), 74.1 (*iso*-6jC19, 6jC17), 42.1 (*iso*-6jC5), 39.4 (*iso*-6jC2), 39.4 (6jC6), 38.4 (6jC3), 37.2 (*iso*-6jC4), 28.8 (6jC5), 22.8 (6jC4), 22.6 (*iso*-6jC3), 14.3 (6jC8), 13.5 (*iso*-6jC22), 10.1 (*iso*-6jC20), 10.1 (6jC18), 3.2 (*iso*-6jC(21, 22)), 3.2 (6jC(19, 20)).

HRMS (ESI): calculated for $C_{18}H_{22}F_2O_4Na$ $[M+Na]^+$ requires m/z 363.1384, found m/z 363.1378.

(*trans*)-3-Hydroxy-5-methyl-3-phenyltetrahydro-4*H*-pyran-4-one *iso*-6k and (*trans*)-3-Hydroxy-4-methyltetrahydrofuran-3-yl)(phenyl)methanone 6k’

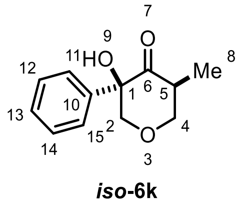

***iso*-6k**

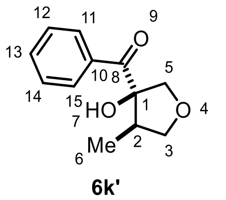

6k'

Synthesized from **5k** (20.7 mg) according to *General Procedure I* (60 °C, 72 h); substrate decomposition occurred when the reaction was run at 90 °C. Analysis of the ^{1}H NMR spectrum of the crude mixture gave a >20:1 d.r. and 1:1.4 ratio of **6k’:*iso*-6k**. **6k’:*iso*-6k** (1:1.4 ratio, >20:1 d.r., 16.0 mg, 45%) were isolated as a colorless oil.

IR (film) ν_{max}/cm^{-1}: 3460, 2931, 2851, 1715, 1095, 936.

^{1}H NMR (500 MHz, $CDCl_3$) δ 8.21 (d, *J* = 7.6 Hz, 6k'C(11, 15)*H*), 7.65 (d, *J* = 7.2 Hz, 2H, *iso*-6kC(11, 15)*H*), 7.63 (t, *J* = 7.6 Hz, 6k'C13*H*), 7.51 (t, *J* = 7.6 Hz, 6k'C(12, 14)*H*), 7.41 (t, *J* = 7.2 Hz, 2H, *iso*-6kC(12, 14)*H*), 7.34 (t, *J* = 7.2 Hz, 1H, *iso*-6kC13*H*), 4.80 (dd, *J* = 11.7 Hz, 1.9 Hz, 1H, *iso*-6kC4H_A), 4.74 (d, *J* = 11.0 Hz, 6k'C5H_A), 4.73 (s, 1H, *iso*-6kO*H*), 4.42 (s, 6k'O*H*), 4.36 (dd, *J* = 7.8, 7.8 Hz, 6k'C3H_A), 4.27 (ddd, *J* = 11.0 Hz, 6.7 Hz, 1.9 Hz, 1H, *iso*-6kC4H_A), 3.93 (d, *J* = 11.0 Hz, 6k'C5fH_B), 3.62 (d, *J* = 11.7 Hz, 1H, *iso*-6kC2H_B), 3.62 (dd, *J* = 11.7 Hz, 7.8 Hz, 6k'C3H_B), 3.41 (t, *J* = 11.0 Hz, 1H, *iso*-6kC4H_B), 2.99 – 2.91 (m, 1H , *iso*-6kC5*H*), 2.82 – 2.75 (m, 6k'C2*H*), 0.98 (d, *J* = 6.6 Hz, 3H, *iso*-6kC8H_3), 0.93 (d, *J* = 6.7 Hz, 6k'C6H_3).

^{13}C NMR (126 MHz, $CDCl_3$) δ 209.9 (*iso*-6kC6), 200.9 (6k'C8), 140.1 (*iso*-6kC10), 133.8 (6k'C13), 131.6 (6k'C10), 130.1 (6k'C(11, 15)), 128.8 (*iso*-6kC(12, 14)), 128.7 (6k'C(12, 14)), 128.3 (*iso*-6kC13), 126.6 (*iso*-6kC(11, 15)), 85.9 (6k'C1), 79.6 (6k'C5), 78.3 (*iso*-6kC1), 77.1 (*iso*-6kC4), 75.4 (*iso*-6kC2), 75.0 (6k'C3), 45.6 (6k'C2), 42.2 (*iso*-6kC5), 8.7 (*iso*-6kC8), 8.5 (6k'C6).

HRMS (ESI): calculated for $C_{12}H_{14}O_3Na$ $[M+Na]^+$ requires m/z 229.0841, found m/z 229.0831.

(*trans)*-(1-Hydroxy-2-methylcyclohexyl)(phenyl)methanone 8a

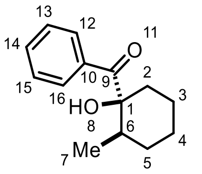

8a

Synthesized from **7a** (21.9 mg) according to *General Procedure J* (70 °C, 40 h). Analysis of the ^{1}H NMR spectrum of the crude mixture gave a >20:1 d.r. of **8a**. **8a** (20.0 mg, 90%) was isolated as a colorless oil.

IR (film) ν_{max}/cm^{-1}: 3585, 2918, 1662, 1464, 1235, 996.

^{1}H NMR (500 MHz, $CDCl_3$) δ 8.00 – 7.98 (m, 2H, C(12, 16)*H*), 7.59 – 7.56 (m, 1H, C14*H*), 7.50 – 7.47 (m, 2H, C(13, 15)*H*), 3.99 (s, 1H, O*H*), 2.31 – 2.24 (m, 1H, C6*H*), 2.08 – 2.02 (m, 1H, C2H_A), 1.85 – 1.66 (m, 4H, C2H_B, C3H_2, C4H_A,), 1.60 – 1.40 (m, 3H, C4H_B, C5H_2), 0.74 (d, *J* = 6.7 Hz, 3H, C7*H*).

^{13}C NMR (126 MHz, $CDCl_3$) δ 206.3 (C9), 135.2 (C10), 132.6 (C14), 129.1 (C(12, 16)), 128.4 (C(13, 15)), 81.2 (C1), 37.8 (C6), 36.8 (C2), 30.1 (C5), 25.9 (C4), 21.1 (C3), 15.9 (C7).

The structural assignment was supported by HMBC correlation between C7H_3 and C1, and between C12/16H and C9. Additionally, and as noted earlier, the ^{13}C resonance of endocyclic (~215 ppm) vs exocyclic (~205 ppm) hydroxy-ketones is distinctive.[**Error! Bookmark not defined.**] *The relative stereochemistry was assigned by NOE analysis of 8 a; related stereochemical assignments are based upon this. These observations were used to support other structural assignments.*

HRMS (ESI): calculated for $C_{14}H_{18}O_2Na$ $[M+Na]^+$ requires m/z 241.1204, found m/z 241.1205.

$[\alpha]_D^{25}$ = +2.6 (c 1.0, CH_2Cl_2).

Chiral SFC: YMC Chiral ART Cellulose-SC column (25 cm), CO_2:*i*-PrOH 95:5, 2.0 mL/min, 171 bar, 40 °C. Retention times: 4.6 mins (major), 4.9 mins (minor), e.r. = 71:29. The racemate was prepared using *rac*-BINAP.

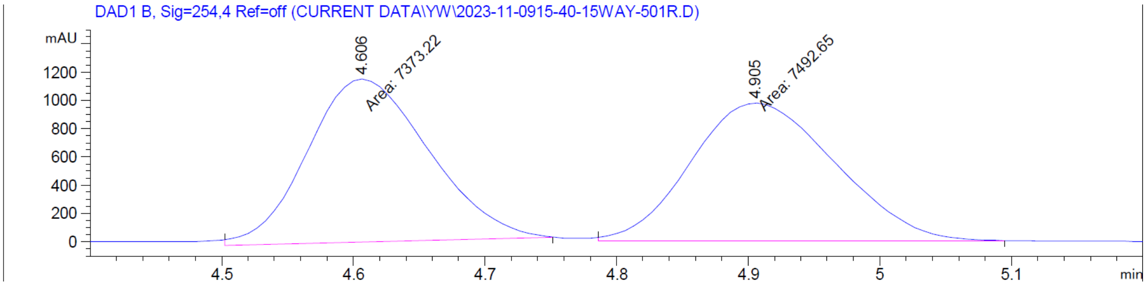

Signal 2: DAD1 B, Sig=254,4 Ref=off

Peak #	RetTime [min]	Type	Width [min]	Area [mAU*s]	Height [mAU]	Area %
1	4.606	MM	0.1064	7373.21826	1154.58557	49.5983
2	4.905	MM	0.1276	7492.65479	978.62250	50.4017

Totals : 1.48659e4 2133.20807

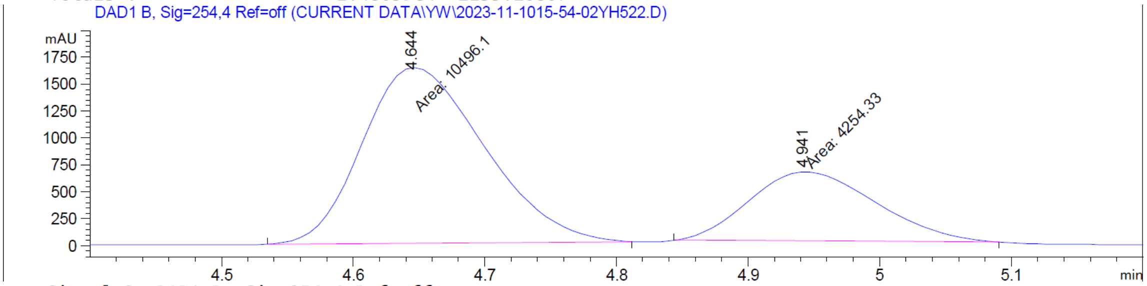

Signal 2: DAD1 B, Sig=254,4 Ref=off

Peak #	RetTime [min]	Type	Width [min]	Area [mAU*s]	Height [mAU]	Area %
1	4.644	MM	0.1070	1.04961e4	1634.74524	71.1580
2	4.941	MM	0.1109	4254.33105	639.37537	28.8420

Totals : 1.47505e4 2274.12061

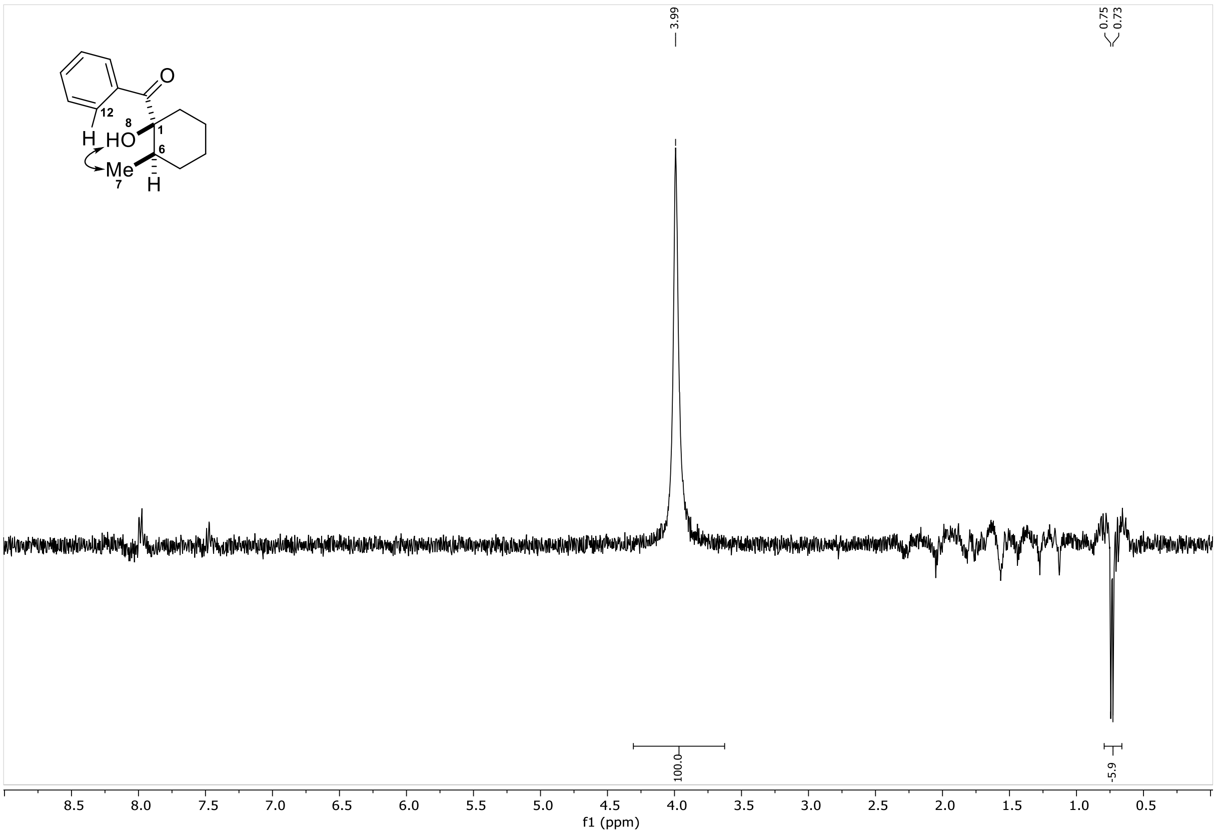

1D NOE spectrum: irradiation of the OH (3.99 ppm) signal showed a correlation to $C7H_3$ (0.75 ppm), which supports a *syn*-relationship.

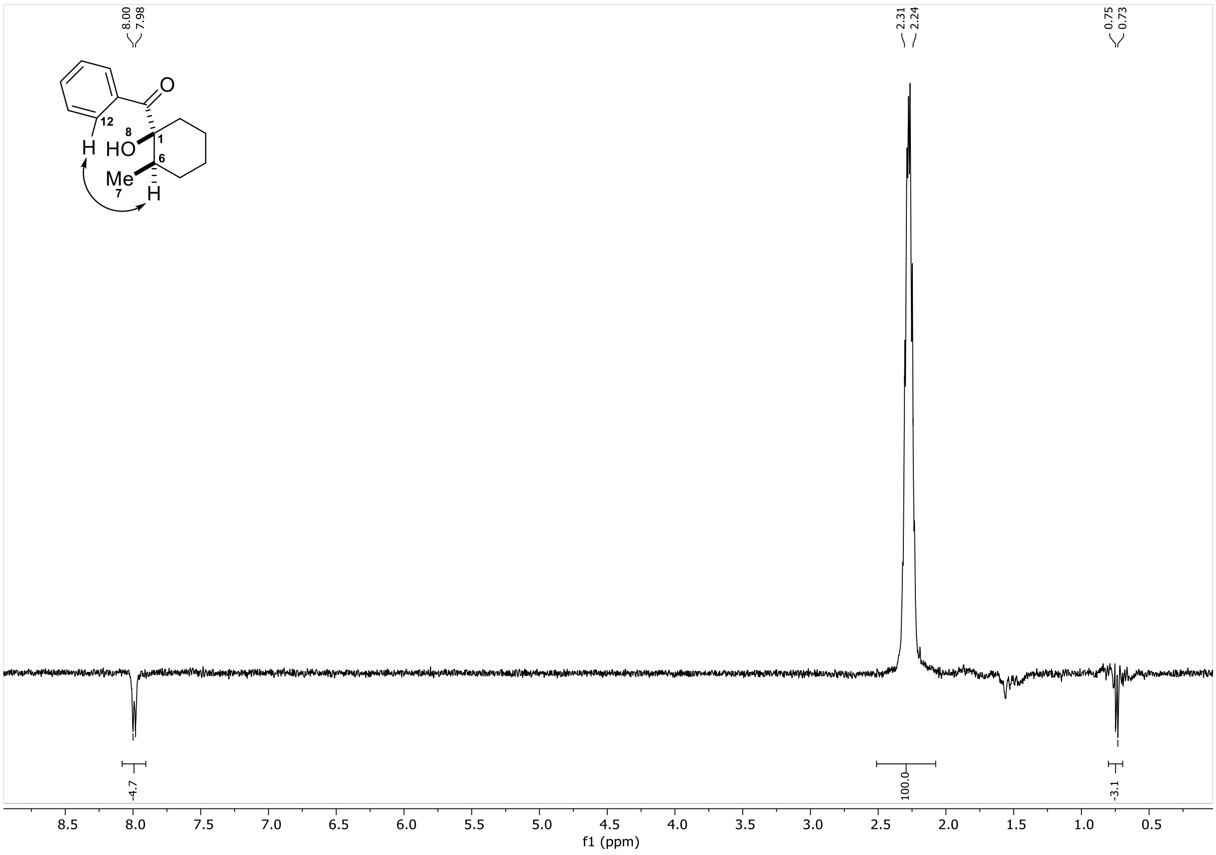

1D NOE spectrum: irradiation of C6H (2.27 ppm) showed a correlation to C12H (8.00 ppm).

(*trans)*-(1-Hydroxy-2-methylcyclohexyl)(*p*-tolyl)methanone 8b

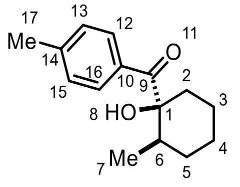

8b

Synthesized from **7b** (23.3 mg) according to *General Procedure J* (70 °C, 40 h). Analysis of the ^{1}H NMR spectrum of the crude mixture gave a >20:1 d.r. of **8b**. **8b** (22.0 mg, 95%) was isolated as a colorless solid.

m.p. = 44–46 °C.

IR (film) ν_{max}/cm^{-1}: 3438, 2930, 1649, 1443, 1047, 997.

^{1}**H NMR** (500 MHz, $CDCl_3$) δ 7.96 – 7.94 (m, 2H, C(12, 16)*H*), 7.28 (m, 2H, C(13, 15)*H*), 4.12 (s, 1H, O*H*), 2.44 (s, 3H, C17*H*), 2.32 – 2.25 (m, 1H, C6*H*), 2.11 – 2.04 (m, 1H, C2H_A), 1.86 – 1.44 (m, 7H, C2H_B, C5H_2, C3H_2, C4H_2), 0.71 (d, *J* = 6.6 Hz, 3H, C7*H*).

^{13}**C NMR** (126 MHz, $CDCl_3$) δ 205.4 (C9), 143.6 (C14), 132.1 (C10), 129.5 (C(12, 16)), 129.1 (C(13, 15)), 81.0 (C1), 38.1 (C6), 36.9 (C2), 30.1 (C5), 26.0 (C4), 21.6 (C17), 21.2 (C3), 15.8 (C7).

HRMS (ESI): calculated for $C_{15}H_{21}O_2$ [M+H]$^+$ requires m/z 233.1542, found m/z 233.1531.

(4-Chlorophenyl)-(*trans)*-(1-hydroxy-2-methylcyclohexyl)methanone 8c

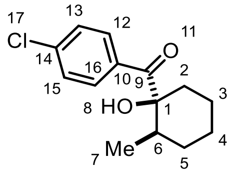

8c

Synthesized from **7c** (25.3 mg) according to *General Procedure J* (70 °C, 40 h). Analysis of the ^{1}H NMR spectrum of the crude mixture gave a >20:1 d.r. of **8c**. **8c** (19.0 mg, 75%) was isolated as a colorless solid

m.p. = 52–54 °C.

IR (film) ν_{max}/cm^{-1}: 3481, 2935, 1663, 1443, 1248, 996.

^{1}**H NMR** (500 MHz, $CDCl_3$) δ 7.98 – 7.96 (m, 2H, C(12, 16)*H*), 7.47 7.44 (m, 2H, C(13, 15)*H*), 3.78 (s, 1H, O*H*), 2.26 – 2.19 (m, 1H, C6*H*), 2.01 – 1.095 (m, 1H, C2H_A), 1.85 – 1.39 (m, 7H, C2H_B, C5H_2, C3H_2, C4H_2), 0.73 (d, *J* = 6.6 Hz, 3H, C7*H*).

^{13}**C NMR** (126 MHz, $CDCl_3$) δ 205.1 (C9), 139.1 (C14), 133.4 (C10), 130.8 (C(12, 16)), 128.7 (C(13, 15)), 81.4 (C1), 37.9 (C6), 36.8 (C2), 30.0 (C5), 25.8 (C4), 21.0 (C3), 15.9 (C7).

HRMS (ESI): calculated for $C_{14}H_{18}Cl^{35}O_2$ [M+H]$^+$ requires m/z 253.0995, found m/z 253.0965.

(2-Fluorophenyl)-(*trans)*-(1-hydroxy-2-methylcyclohexyl)methanone 8d

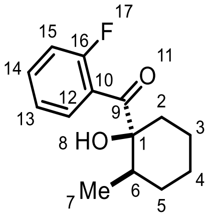

8d

Synthesized from **7d** (23.7 mg) according to *General Procedure J* (70 °C, 40 h). Analysis of the ^{1}H NMR spectrum of the crude mixture gave a >20:1 d.r. of **8d**. **8d** (15.0 mg, 64%) was isolated as a colorless oil.

IR (film) ν_{max}/cm^{-1}: 3496, 2932, 1688, 1448, 1211, 995.

^{1}**H NMR** (500 MHz, $CDCl_3$) δ 7.49 – 7.45 (m, 1H, C14*H*), 7.40 – 7.36 (m, 1H, C12*H*), 7.22 (m, 1H, C13*H*), 7.15 (m, 1H, C15*H*), 3.24 (s, 1H, O*H*), 2.12 – 2.04 (m, 1H, C6*H*), 1.89 – 1.85 (m, 1H, C2H_A), 1.75 – 1.54 (m, 5H, C2H_B, C5H_2, C3H_A, C4H_A), 1.45 (m, 1H, C3H_B), 1.36 – 1.27 (m, 1H, C4H_B), 0.87 (d, *J* = 6.7 Hz, 3H, C7*H*).

^{19}**F NMR** (470 MHz, $CDCl_3$) δ –110.7.

^{13}**C NMR** (126 MHz, $CDCl_3$) δ 208.5 (d, *J* = 1.7 Hz, C9), 158.8 (d, *J* = 249.2 Hz, C16), 132.1 (d, *J* = 8.2 Hz, C14), 128.3 (d, *J* = 3.7 Hz, C12), 127.1 (d, *J* = 17.3 Hz, C10), 124.1 (d, *J* = 3.5 Hz, C13), 116.2 (d, *J* = 22.6 Hz, C15), 83.3 (C1), 36.3 (C6), 34.9 (d, *J* = 2.7 Hz, C2), 29.8 (C5), 25.5 (C4), 20.6 (C3), 16.2 (C7).

HRMS (ESI): calculated for $C_{14}H_{17}FO_2Na$ [M+Na]$^+$ requires m/z 259.1110, found m/z 259.1102.

(*trans)*-(1-Hydroxy-2-methylcyclohexyl)(3-methoxyphenyl)methanone 8e

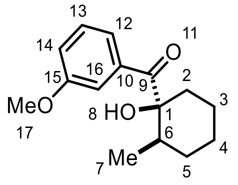

8e

Synthesized from **7e** (24.9 mg) according to *General Procedure J* (70 °C, 40 h). Analysis of the ^{1}H NMR spectrum of the crude mixture gave a >20:1 d.r. of **8e**. **8e** (21.0 mg, 85%) was isolated as a colorless oil.

IR (film) ν_{max}/cm^{-1}: 3353, 2972, 1663, 1448, 1256, 1047.

^{1}**H NMR** (500 MHz, $CDCl_3$) δ 8.59 (dt, *J* = 7.9, 1.2 Hz, 1H, C12*H*), 7.49 (t, *J* = 2.0 Hz, 1H, C16*H*), 7.39 (t, *J* = 8.1 Hz, 1H, C13*H*), 7.12 (dd, *J* = 8.1, 2.6 Hz, 1H, C14*H*), 3.98 (s, 1H, O*H*), 3.88 (s, 3H, C17*H*), 2.31 – 2.24 (m, 1H, C6*H*), 2.08 – 2.01 (m, 1H, C2H_A), 1.85 – 1.66 (m, 4H, C2H_B, C3H_2, C4H_A,), 1.59 – 1.39 (m, 3H, C4H_B, C5H_2), 0.74 (d, *J* = 6.7 Hz, 3H, C7*H*).

^{13}**C NMR** (126 MHz, $CDCl_3$) δ 206.2 (C9), 159.5 (C15), 136.4 (C10), 129.4 (C13), 121.4 (C12), 118.5 (C14), 114.3 (C16), 81.3 (C1), 55.5 (C17), 37.9 (C6), 36.8 (C2), 30.1 (C5), 25.9 (C4), 21.1 (C3), 15.9 (C7).

HRMS (ESI): calculated for $C_{15}H_{20}O_3Na$ [M+Na]$^+$ requires m/z 271.1310, found m/z 271.1307.

(*trans*)-(1-Hydroxy-2-methylcyclohexyl)(3-(trifluoromethyl)phenyl)methanone 8f

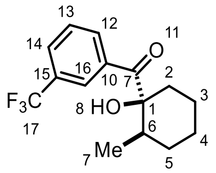

8f

Synthesized from **7f** (28.7 mg) according to *General Procedure J* (70 °C, 40 h). Analysis of the ^{1}H NMR spectrum of the crude mixture gave a 10:1 d.r. of **8f**. **8f** (10:1 d.r., 16.0 mg, 56%) was isolated as a colorless solid.

m.p. = 86–89 °C.

IR (film) ν_{max}/cm^{-1}: 3503, 2941, 1668, 1329, 1121, 988.

^{1}H NMR (500 MHz, $CDCl_3$) δ 8.25 (s, 1H, C16*H*), 8.19 (d, *J* = 7.9 Hz, 1H, C12*H*), 7.83 (d, *J* = 7.9 Hz, 1H, C14*H*), 7.62 (t, *J* = 7.8 Hz, 1H, C13*H*), 3.54 (s, 1H, O*H*), 2.26 – 2.19 (m, 1H, C6*H*), 1.98 – 1.92 (m, 1H, C2H_A), 1.85 – 1.67 (m, 4H, C2H_B, C3H_2, C4H_A,), 1.63 – 1.39 (m, 3H, C4H_B, C5H_2), 0.78 (d, *J* = 6.8 Hz, 3H, C7*H*).

^{19}F NMR (470 MHz, $CDCl_3$) δ –62.8.

^{13}C NMR (126 MHz, $CDCl_3$) δ 205.4 (C9), 136.2 (C10), 132.3 (C13), 131.0 (q, *J* = 32.9 Hz, C15), 129.0 (C12), 128.9 (q, *J* = 3.4 Hz, C14), 126.1 (q, *J* = 3.8 Hz, C16), 123.6 (q, *J* = 271.5 Hz, C17), 81.8 (C1), 37.8 (C6), 36.8 (C2), 29.9 (C5), 25.7 (C4), 20.9 (C3), 16.0 (C7).

HRMS (ESI): calculated for $C_{15}H_{17}F_3O_2Na$ $[M+Na]^+$ requires m/z 309.1078, found m/z 309.1068.

(*trans*)-(1-Hydroxy-2-methylcyclohexyl)(naphthalen-2-yl)methanone 8g

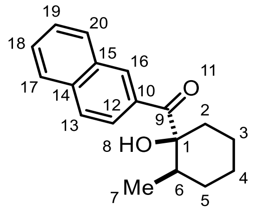

8g

Synthesized from **7g** (26.8 mg) according to *General Procedure J* (70 °C, 40 h). Analysis of the ^{1}H NMR spectrum of the crude mixture gave a >20:1 d.r. of **8g**. **8g** (24.0 mg, 89%) was isolated as a colorless solid.

m.p. = 54–56 °C.

IR (film) ν_{max}/cm^{-1}: 3505, 2934, 1654, 1446, 1190, 1001.

^{1}H NMR (500 MHz, $CDCl_3$) δ 8.59 (s, 1H, C16*H*), 8.05 (d, *J* = 8.5 Hz, 1H, C12*H*), 8.00 (d, *J* = 8.2 Hz, 1H, C13*H*), 7.91 (t, *J* = 8.5 Hz, 2H, Ar*H*), 7.65 – 7.58 (m, 2H, Ar*H*), 4.05 (s, 1H, O*H*), 2.43 – 2.36 (m, 1H, C6*H*), 2.20 – 2.14 (m, 1H, C2H_A), 1.89 – 1.71 (m, 4H, C2H_B, C3H_2, C4H_A), 1.61 – 1.47 (m, 3H, C4H_B, C5H_2), 0.78 (d, *J* = 6.7 Hz, 3H, C7*H*).

^{13}C NMR (126 MHz, $CDCl_3$) δ 206.1 (C9), 135.1 (C10), 132.3 (C14, C15), 130.6 (C16), 129.6 (C13), 128.6 (ArC), 128.2 (ArC), 127.7 (ArC), 126.9 (ArC), 125.2 (C12), 81.4 (C1), 38.1 (C6), 37.1 (C2), 30.1 (C5), 26.0 (C4), 21.2 (C3), 16.0 (C7).

HRMS (ESI): calculated for $C_{18}H_{20}O_2Na$ $[M+Na]^+$ requires m/z 291.1361, found m/z 291.1370.

(Furan-2-yl)-(*trans)*-(1-hydroxy-2-methylcyclohexyl)methanone 8h

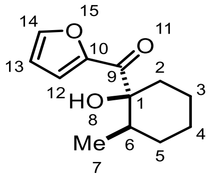

8h

Synthesized from **7h** (20.8 mg) according to *General Procedure J* (70 °C, 40 h). Analysis of the ^{1}H NMR spectrum of the crude mixture gave a >20:1 d.r. of **8h**. **8h** (18.0 mg, 85%) was isolated as a colorless oil.

IR (film) ν_{max}/cm^{-1}: 3462, 2929, 1650, 1463, 1287, 1001.

^{1}H NMR (500 MHz, $CDCl_3$) δ 7.65 (d, *J* = 1.6 Hz, 1H, C12*H*), 7.43 (d, *J* = 3.5 Hz, 1H, C14*H*), 6.59 (dd, *J* = 3.6, 1.7 Hz, 1H, C13*H*), 3.94 (s, 1H, O*H*), 2.39 – 2.32 (m, 1H, C6*H*), 2.15 (td, *J* = 13.2, 4.8 Hz, 1H, C2H_A), 1.84 – 1.44 (m, 7H, C2H_B, C(3, 4, 5)H_2), 0.69 (d, *J* = 6.7 Hz, 3H, C7*H*).

^{13}C NMR (126 MHz, $CDCl_3$) δ 193.3 (C9), 150.6 (C10), 146.5 (C12), 120.4 (C14), 112.3 (C13), 79.9 (C1), 37.2 (C6), 35.8 (C2), 29.9 (C5), 25.9 (C4), 21.0 (C3), 15.7 (C7).

HRMS (ESI): calculated for $C_{12}H_{16}O_3Na$ $[M+Na]^+$ requires m/z 231.0997, found m/z 231.0990.

(*trans)*-(1-Hydroxy-2-methylcyclohexyl)(1-methyl-1*H*-indol-2-yl)methanone 8i

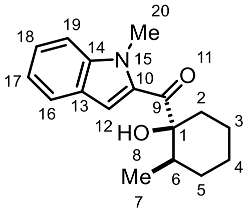

8i

Synthesized from **7i** (27.2 mg) according to *General Procedure J* (90 °C for 40 h). Analysis of the ^{1}H NMR spectrum of the crude mixture gave a >20:1 d.r. of **8i**. **8i** (8.0 mg, 30%) was isolated as a yellow solid.

m.p. = 61–63 °C.

IR (film) ν_{max}/cm^{-1}: 3401, 2925, 1628, 1369, 1123, 941.

^{1}H NMR (500 MHz, $CDCl_3$) δ 7.74 (dt, *J* = 8.0 Hz, 1.1 Hz, 1H, C19*H*), 7.57 (s, 1H, C12*H*), 7.44 – 7.43 (m, 2H, C(16, 18)*H*), 7.23 – 7.17 (m, 1H, C17*H*), 4.25 (s, 1H, O*H*), 4.07 (s, 3H, C20H_3), 2.44 – 2.37 (m, 1H, C6*H*), 2.23 – 2.17 (m, 1H, C2H_A), 1.89 – 1.70 (m, 4H, C2H_B, C5H_2, C4H_A), 1.61 – 1.51 (m, 3H, C3H_2, C4H_B), 0.73 (d, *J* = 6.6 Hz, 3H, C7*H*).

^{13}C NMR (126 MHz, $CDCl_3$) δ 198.7 (C9), 139.7 (C10), 131.2 (C14), 126.4 (C18), 125.7 (C13), 123.1 (C19), 121.0 (C17), 112.5 (C12), 110.5 (C16), 80.8 (C1), 39.5 (C6), 38.3 (C2), 33.0 (C20), 30.4 (C5), 26.1 (C4), 21.5 (C3), 15.7 (C7).

HRMS (ESI): calculated for $C_{17}H_{22}NO_2$ $[M+H]^+$ requires m/z 272.1651, found m/z 272.1646.

(3-(Cyclopropylmethoxy)-4-(difluoromethoxy)phenyl)-(*trans)*-(1-hydroxy-2-methylcyclohexyl)methanone 8j

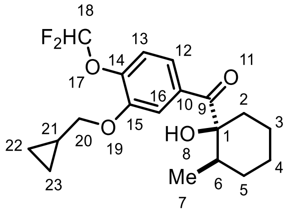

8j

Synthesized from **7j** (35.4 mg) according to *General Procedure J* (90 °C, 40 h). Analysis of the ^{1}H NMR spectrum of the crude mixture gave a >20:1 d.r. of **8j**. **8j** (16.0 mg, 45%) was isolated as a colorless oil.

IR (film) ν_{max}/cm^{-1}: 3472, 2931, 1661, 1265, 1111, 997.

^{1}H NMR (500 MHz, $CDCl_3$) δ 7.70 (dd, *J* = 8.5 Hz, 1.8 Hz, 1H, C12*H*), 7.62 (d, *J* = 1.8 Hz, 1H, C16*H*), 7.22 (d, *J* = 8.5 Hz, 1H, C13*H*), 6.76 (t, *J* = 74.8 Hz, 1H, C18*H*), 3.95 (d, *J* = 7.0 Hz, 2H, C20*H*), 3.83 (s, 1H, O*H*), 2.29 – 2.22 (m, 1H, C6*H*), 2.01 (td, *J* = 13.2 Hz, 4.2 Hz, 1H, C2H_A), 1.81 – 1.66 (m, 4H, C2H_B, C5H_2, C4H_A), 1.61 – 1.28 (m, 4H, C3H_2, C4H_B, C21*H*), 0.73 (d, *J* = 6.6 Hz, 3H, C7*H*), 0.71 – 0.67 (m, 2H, C22H_2), 0.41 – 0.38 (m, 2H, C23H_2).

^{19}F NMR (470 MHz, $CDCl_3$) δ –81.9, –82.0.

^{13}C NMR (126 MHz, $CDCl_3$) δ 204.6 (C9), 150.3 (C15), 143.9 (t, *J* = 3.2 Hz, C14), 132.9 (C10), 122.7 (C12), 121.4 (C13), 115.7 (t, *J* = 261.0 Hz, C18), 115.5 (C16), 81.4 (C1), 74.1 (C20), 38.2 (C6), 37.0 (C2), 30.0 (C5), 25.9 (C4), 21.1 (C3), 15.2 (C9), 10.1 (C21), 3.3 (C22, 23).

HRMS (ESI): calculated for $C_{19}H_{25}F_2O_4$ [M+H]$^+$ requires m/z 355.1721, found m/z 355.1725.

(*trans)*-(3-Hydroxy-4-methyltetrahydro-2*H*-pyran-3-yl)(phenyl)methanone 8k

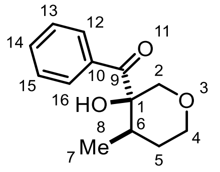

Synthesized from **7k** (22.0 mg) according to *General Procedure J* (90 °C, 40 h). Analysis of the ^{1}H NMR spectrum of the crude mixture gave a >20:1 d.r. of **8k**. **8k** (11.0 mg, 50%) was isolated as a colorless oil.

IR (film) ν_{max}/cm^{-1}: 3456, 2920, 1667, 1447, 1229, 1086.

^{1}H NMR (500 MHz, $CDCl_3$) δ 8.09 – 8.07 (m, 2H, C(12, 16)*H*), 7.59 – 7.56 (m, 1H, C14*H*), 7.48 – 7.43 (m, 2H, C(13, 15)*H*), 4.06 (dd, *J* = 11.4 Hz, 4.9 Hz, 1H, C4H_A) 3.87 (d, *J* = 11.5 Hz, 1H, C2H_A), 3.82 (d, *J* = 11.5 Hz, 1H, C2H_B), 3.58 (ddd, *J* = 12.8 Hz, 11.4 Hz, 2.6 Hz, 1H, C4H_B), 3.34 (s, 1H, O*H*), 2.48 – 2.41 (m, 1H, C6*H*), 1.75 (m, 1H, C5H_A), 1.58 – 1.54 (m, 1H, C5H_B), 0.91 (d, *J* = 6.7 Hz, 3H, C7*H*).

^{13}C NMR (126 MHz, $CDCl_3$) δ 203.9 (C9), 136.5 (C10), 132.8 (C14), 129.6 (C(12, 16)), 128.2 (C(13, 15)), 81.7 (C1), 74.6 (C4), 68.3 (C2), 36.2 (C6), 29.5 (C5), 15.9 (C7).

HRMS (ESI): calculated for $C_{13}H_{16}O_3Na$ [M+Na]$^+$ requires m/z 243.0997, found m/z 243.0994.

Development of an enantioselective protocol (Table S2)

***L1-8** are as shown in the manuscript and **L9-12** as shown in Table S1.*

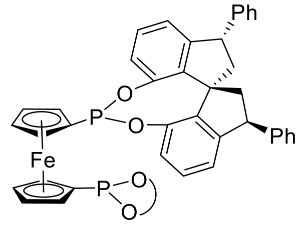

L13

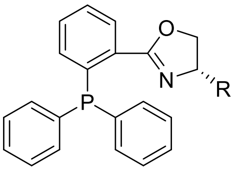

R = *t*Bu, **L14**
R = Ph, **L15**

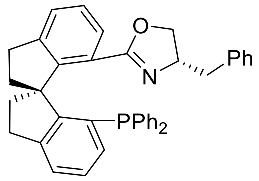

L16

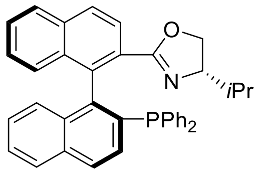

L17

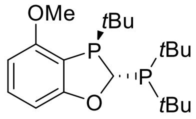

L18

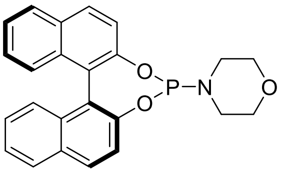

L19

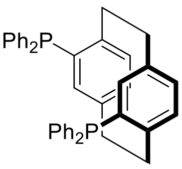

L20

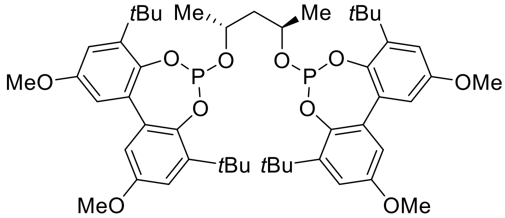

L21

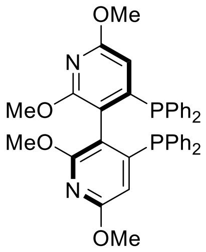

L22

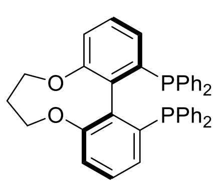

L23

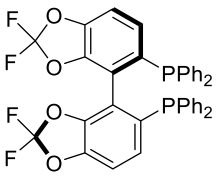

L24

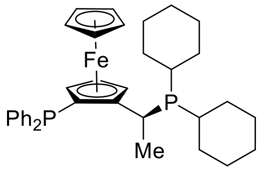

L25

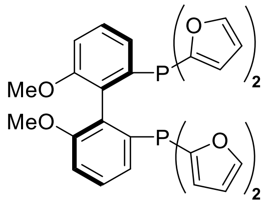

L26

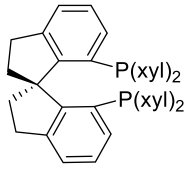

L27

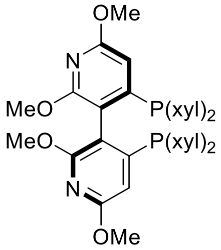

L28

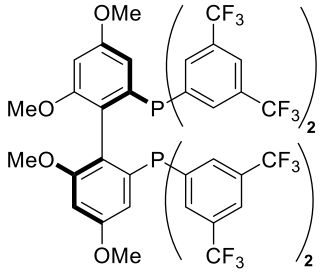

L29

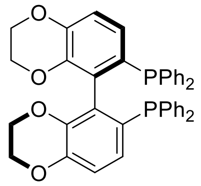

(*S*)-L6
(*S*)-SYNPHOS

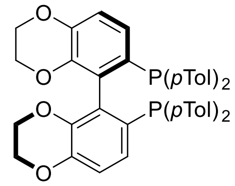

L30
(*R*)-Tol-SYNPHOS

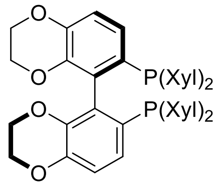

L31
(*R*)-DM-SYNPHOS

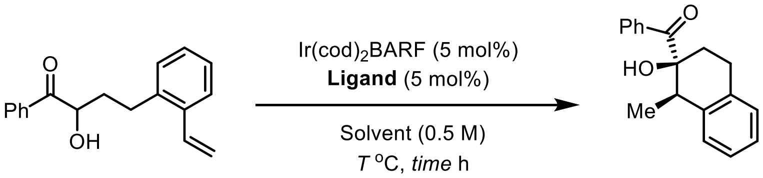

7m **8m**, >20:1 dr[a]

Entry	Ligand	Solvent	*T* °C/time h[b]	Yield[c]	e.r.[d]
1	**L2**	2-MeTHF	70/24	75%	76:24
2	**L2**	2-MeTHF	60/72	82%	78:22
3	**L3**	2-MeTHF	70/24	75%	88:12
4	**L4**	2-MeTHF	90/24	63%	61:39
5	**L13**	2-MeTHF	80/72	69%	55:45
6	**L14**	2-MeTHF	90/24	0	-
7	**L15**	2-MeTHF	90/24	0	-
8	**L16**	2-MeTHF	90/24	0	-
9	**L17**	2-MeTHF	90/24	0	-
10	**L18**	2-MeTHF	80/72	53%	56.5:43.5
11	**L19**	2-MeTHF	80/72	38%	60.5:39:5
12	**L20**	2-MeTHF	80/72	30%	63.5:36.5
13	**L21**	2-MeTHF	80/24	*decomposed*	-
14	**L22**	2-MeTHF	70/24	75%	90:10
15	**L23**	2-MeTHF	70/24	60%	55:45
16	**L6**	2-MeTHF	70/24	82%	92.5:7.5
17	**L24**	2-MeTHF	70/24	56%	73:27
18	**L25**	2-MeTHF	80/72	75%	83:17
19	**L26**	2-MeTHF	80/72	64%	63.5:36.5
20	**L27**	2-MeTHF	90/24	<10%	-
21	**L28**	2-MeTHF	80/72	90%	87:13
22	**L29**	2-MeTHF	70/24	86%	56:44
23	**L6**	Toluene	70/24	38%	75.5:24.5
24	**L6**	1,4-Dioxane	70/24	<10%	-
25	**L6**	*o*-DCB	70/24	53%	63.5:36.5
26	**L6**	PhCl	70/24	45%	66:34
27	**L6**	2-MeTHF	60/72	86%	97:3
28	**L6**	THF	60/72	90%	94:6
29	**L6**	CPME	60/72	94%	94:6
30	**L6**	TBME	60/72	90%	79:21

[a] Diastereoselectivities were determined by 1H NMR analysis of the crude mixture; [b] The lowest temperature that gave significant conversion was used, and the reaction time was adjusted to allow >80% conversion of starting material in most of entries; [c] Isolated yield; [d] Determined by chiral SFC analysis.

(1*R*,2*S*)-(2-Hydroxy-1-methyl-1,2,3,4-tetrahydronaphthalen-2-yl)(phenyl)methanone 8m

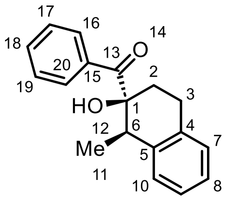

8m

Synthesized from **7m** (26.6 mg) according to *General Procedure J* (60 ˚C, 72 h) with **L6** (*R*)-SynPhos (3.19 mg). **8** Analysis of the ^{1}H NMR spectrum of the crude mixture gave a >20:1 d.r. of **8m**. **8m** (23.0 mg, 86%) was isolated as a colorless oil.

IR (film) ν_{max}/cm^{-1}: 3457, 2933, 1662, 1446, 1213, 959.

^{1}H NMR (500 MHz, $CDCl_3$) δ 8.06 (d, *J* = 8.0 Hz, 2H, C(16, 20)*H*), 7.62 – 7.59 (m, 1H, C18*H*), 7.51 – 7.48 (m, 2H, C(17, 19)*H*), 7.31 – 7.28 (m, 1H, C10*H*), 7.25 – 7.20 (m, 3H, C(7, 8, 9)*H*), 4.01 (s, 1H, O*H*), 3.69 (q, *J* = 6.9 Hz, 1H, C6*H*), 3.31 – 3.24 (m, 1H, C3H_A), 2.93 – 2.87 (m, 1H, C3H_B), 2.59 – 2.53 (m, 1H, C2H_A), 2.12 – 2.08 (m, 1H, C2H_B). 1.25 (d, *J* = 6.9 Hz, 3H, C11H_3).

^{13}C NMR (126 MHz, $CDCl_3$) δ 205.1 (C13), 138.4 (C5), 135.8 (C4), 134.6 (C15), 133.0 (C18), 129.4 (C(16, 20)), 128.6 (C7), 128.6 (C(17, 19)), 127.0 (C10), 126.4 (C8), 126.0 (C9), 80.7 (C1), 39.0 (C6), 33.8 (C2), 26.0 (C3), 14.2 (C11).

$[\alpha]_D^{25}$ = +33.8 (c 0.5, CH_2Cl_2).

HRMS (ESI): calculated for $C_{18}H_{19}O_2$ $[M+H]^+$ requires m/z 267.1385, found m/z 267.1384.

Chiral SFC: YMC Chiral ART Cellulose-SC column (25 cm), CO_2:*i*-PrOH 95:5, 2.0 mL/min, 168 bar, 40 °C. Retention times: 14.5 mins (minor), 16.1 mins (major), e.r. = 97:3. The racemate was prepared using *rac*-BINAP.

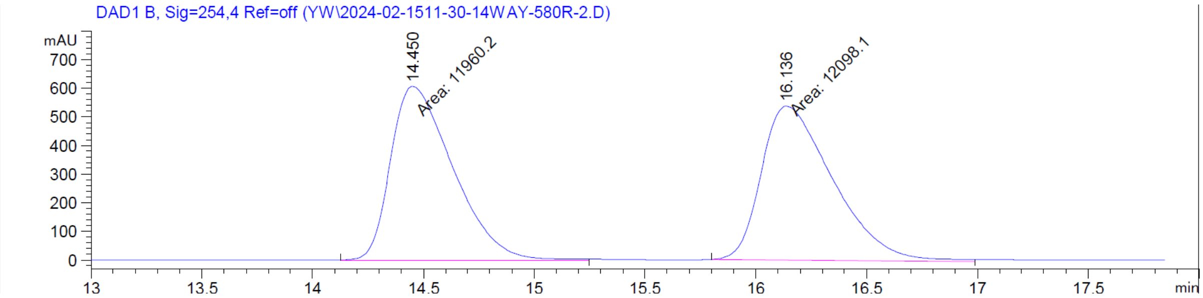

Signal 2: DAD1 B, Sig=254,4 Ref=off

Peak #	RetTime [min]	Type	Width [min]	Area [mAU*s]	Height [mAU]	Area %
1	14.450	MM	0.3270	1.19602e4	609.54321	49.7134
2	16.136	MM	0.3740	1.20981e4	539.16949	50.2866
Totals :				2.40583e4	1148.71271	

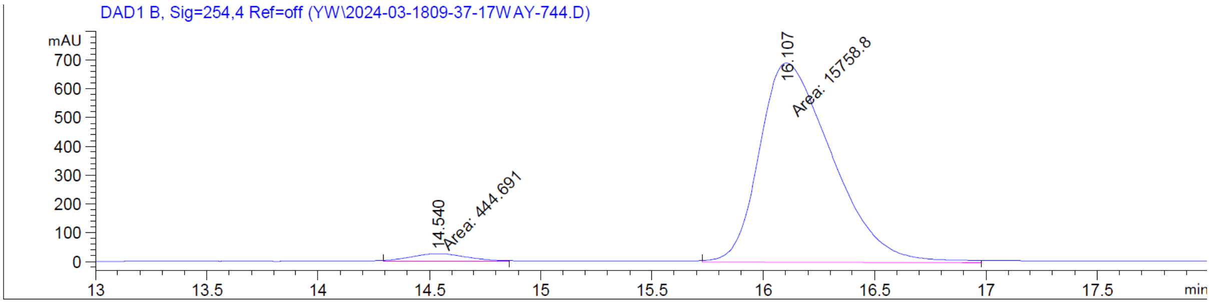


```
Signal 2: DAD1 B, Sig=254,4 Ref=off

Peak RetTime Type  Width     Area      Height     Area
  #   [min]        [min]   [mAU*s]     [mAU]        %
----|-------|----|-------|----------|----------|--------|
   1  14.540 MM    0.2893  444.69107   25.61879   2.7444
   2  16.107 MM    0.3793 1.57588e4   692.51532  97.2556

Totals :                  1.62035e4   718.13411
```

(1*R*,2*S*)-(2-Hydroxy-1-methyl-1,2,3,4-tetrahydronaphthalen-2-yl)(*p*-tolyl)methanone 8n

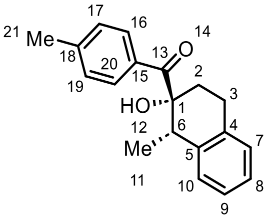

8n

Synthesized from **7n** (28.0 mg) according to *General Procedure J* (60 ˚C, 72 h) with **L6** (*S*)-SynPhos (3.19 mg). Analysis of the ^{1}H NMR spectrum of the crude mixture gave a >20:1 d.r. of **8n**

Yield for **8n** (26.0 mg, 93%) as a colorless oil.

IR (film) ν_{max}/cm^{-1}: 3333, 2974, 1667, 1380, 1046, 880.

^{1}H NMR (500 MHz, $CDCl_3$) δ 8.00 (d, *J* = 8.3 Hz, 2H, C(16, 20)*H*), 7.31 – 7.29 (m, 3H, Ar*H*), 7.25 – 7.19 (m, 3H, Ar*H*), 4.16 (s, 1H, O*H*), 3.69 (q, *J* = 6.8 Hz, 1H, C6*H*), 3.32 – 3.25 (m, 1H, C3H_A), 2.92 – 2.87 (m, 1H, C3H_B), 2.60 – 2.54 (m, 1H, C2H_A), 2.45 (s, 3H, C21*H*), 2.10 – 2.05 (m, 1H, C2H_B). 1.22 (d, *J* = 6.9 Hz, 3H, C11H_3).

^{13}C NMR (126 MHz, $CDCl_3$) δ 204.2 (C13), 144.1 (C18), 138.5 (C5), 135.9 (C4), 131.5 (C15), 129.8 (C(16, 20)), 129.3 (C(17, 19)), 128.6 (ArC), 127.0 (ArC), 126.3 (ArC), 126.0 (ArC), 80.4 (C1), 39.1 (C6), 33.9 (C2), 26.1 (C3), 21.7 (C21), 14.1 (C11).

$[\alpha]_D^{25}$ = –30.9 (c 1.0, CH_2Cl_2).

HRMS (ESI): calculated for $C_{18}H_{20}O_2Na$ $[M+Na]^+$ requires m/z 303.1361, found m/z 303.1353.

Chiral SFC: YMC Chiral ART Cellulose-SC column (25 cm), CO_2:*i*-PrOH 90:10, 2.0 mL/min, 170 bar, 40 °C. Retention times: 10.2 mins (major), 11.6 mins (minor), e.r. = 92:8. The racemate was prepared using *rac*-BINAP.

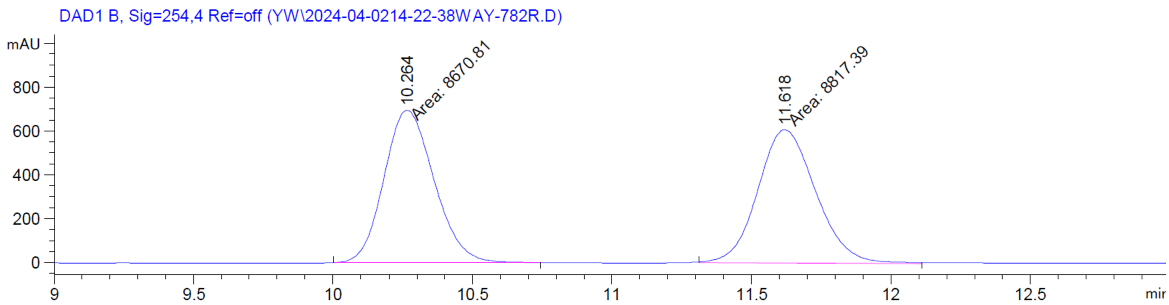

Signal 2: DAD1 B, Sig=254,4 Ref=off

Peak #	RetTime [min]	Type	Width [min]	Area [mAU*s]	Height [mAU]	Area %
1	10.264	MM	0.2079	8670.81055	695.11902	49.5809
2	11.618	MM	0.2416	8817.38672	608.38367	50.4191
Totals :				1.74882e4	1303.50269	

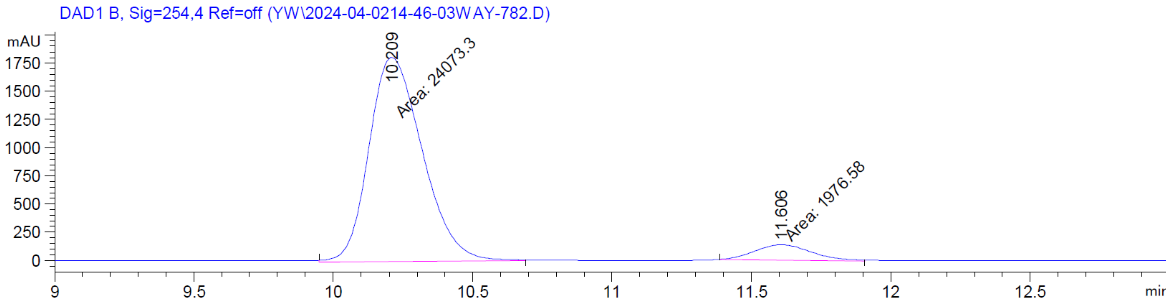

Signal 2: DAD1 B, Sig=254,4 Ref=off

Peak #	RetTime [min]	Type	Width [min]	Area [mAU*s]	Height [mAU]	Area %
1	10.209	MM	0.2215	2.40733e4	1811.00696	92.4123
2	11.606	MM	0.2390	1976.58240	137.84418	7.5877
Totals :				2.60499e4	1948.85114	

(4-Bromophenyl)-(1*R*,2*S*)-(2-hydroxy-1-methyl-1,2,3,4-tetrahydronaphthalen-2-yl)methanone 8o

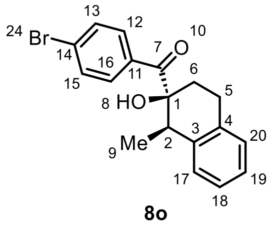

8o

Synthesized from **7o** (34.5 mg) according to *General Procedure J* (70 °C, 72 h) with **L31** (*R*)-DM-SynPhos (3.75 mg). Analysis of the ^{1}H NMR spectrum of the crude mixture gave a >20:1 d.r. of **8o**. **8o** (26.0 mg, 75%) was isolated as a colorless oil.

IR (film) ν_{max}/cm^{-1}: 3467, 2970, 1667, 1582, 1073, 960.

^{1}H NMR (500 MHz, $CDCl_3$) δ 7.98 – 7.95 (m, 2H, C(12, 16)*H*), 7.64 – 7.61 (m, 2H, C(13, 15)*H*), 7.29 (d, *J* = 7.9 Hz, 1H, C20*H*), 7.25 – 7.18 (m, 3H, Ar*H*), 3.68 (s, 1H, O*H*), 3.64 (q, *J* = 6.9 Hz, 1H, C2*H*), 3.28 – 3.21 (m, 1H, C5H_A), 2.91 – 2.86 (m, 1H, C5H_B), 2.49 – 2.43 (m, 1H, C6H_A), 2.12 – 2.08 (m, 1H, C6H_B). 1.25 (d, *J* = 6.9 Hz, 3H, C9H_3).

^{13}C NMR (126 MHz, $CDCl_3$) δ 203.9 (C7), 138.2 (ArC), 135.5 (ArC), 133.4 (ArC), 131.8 (C(13, 15)), 131.2 (C(12, 16)), 128.6 (ArC), 128.2 (ArC), 127.1 (ArC), 126.5 (ArC), 126.1 (ArC), 80.9 (C1), 39.1 (C2), 33.7 (C6), 25.9 (C5), 14.4 (C9).

$[\alpha]_D^{25}$ = +9.2, (c 1.0, CH_2Cl_2).

HRMS (ESI): calculated for $C_{18}H_{18}Br^{79}O_2$ $[M+H]^+$ requires m/z 345.0490, found m/z 345.0485.

Chiral SFC: YMC Chiral ART Cellulose-SC column (25 cm), CO_2:*i*-PrOH 90:10, 2.0 mL/min, 159 bar, 40 °C. Retention times: 7.5 mins (minor), 7.9 mins (major), e.r. = 84:16. The racemate was prepared using *rac*-BINAP.

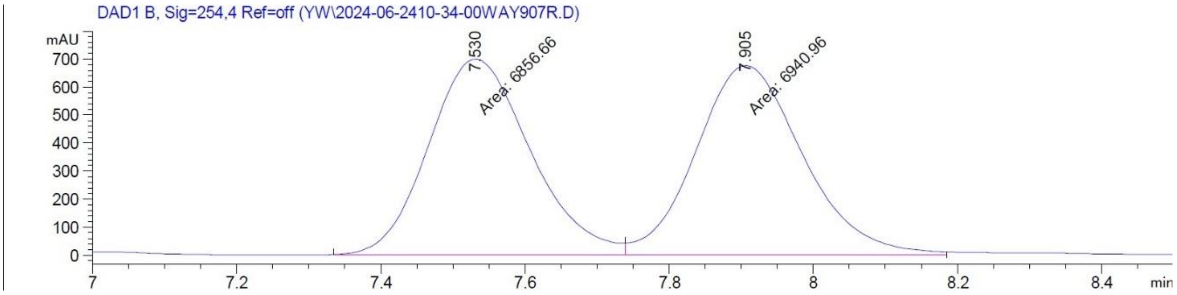

Signal 2: DAD1 B, Sig=254,4 Ref=off

Peak #	RetTime [min]	Type	Width [min]	Area [mAU*s]	Height [mAU]	Area %
1	7.530	MF	0.1633	6856.66357	699.76105	49.6945
2	7.905	FM	0.1712	6940.96484	675.85968	50.3055
Totals :				1.37976e4	1375.62073	

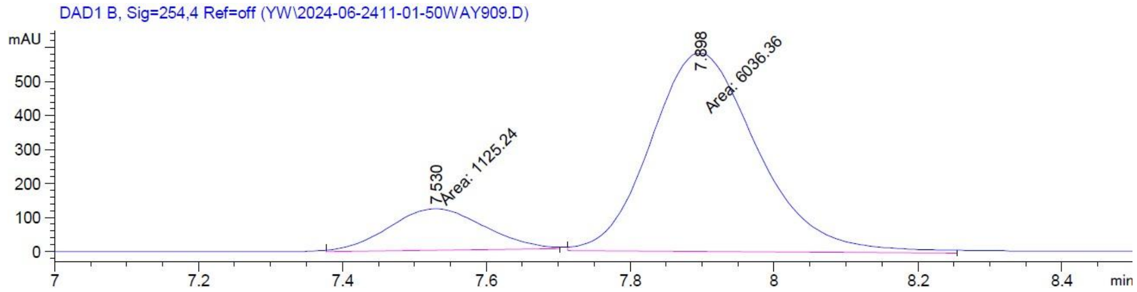

Signal 2: DAD1 B, Sig=254,4 Ref=off

Peak #	RetTime [min]	Type	Width [min]	Area [mAU*s]	Height [mAU]	Area %
1	7.530	MM	0.1538	1125.23767	121.93246	15.7121
2	7.898	MM	0.1721	6036.35986	584.69574	84.2879
Totals :				7161.59753	706.62820	

(1*R*,2*S*)-(2-Hydroxy-1-methyl-1,2,3,4-tetrahydronaphthalen-2-yl)(3-(trifluoromethyl)phenyl)methanone 8p

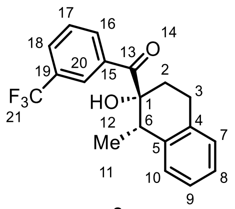

8p

Synthesized from **7p** (33.4 mg) according to *General Procedure J* (60 °C, 72 h) with **L6** (*S*)-SynPhos (3.19 mg). Analysis of the ^{1}H NMR spectrum of the crude mixture gave a 10:1 d.r. of **8p**. **8p** (10:1 d.r., 26.0 mg, 78%) was isolated as a colorless oil.

IR (film) ν_{max}/cm^{-1}: 3491, 2937, 18k, 1330, 1122, 960.

^{1}H NMR (500 MHz, $CDCl_3$) δ 8.35 (s, 1H, C20*H*), 8.27 (d, *J* = 7.9 Hz, 1H, C16*H*), 7.85 (d, *J* = 7.9 Hz, 1H, C18*H*), 7.63 (t, *J* = 7.9 Hz, 1H, C17*H*), 7.31 (d, *J* = 7.9 Hz, 1H, C10*H*), 7.26 – 7.18 (m, 3H, Ar*H*), 3.65 (q, *J* = 7.0 Hz, 1H, C6*H*), 3.40 (s, 1H, O*H*), 3.27 – 3.20 (m, 1H, C3H_A), 2.94 – 2.88 (m, 1H, C3H_B), 2.48 – 2.42 (m, 1H, C2H_A), 2.18 – 2.13 (m, 1H, C2H_B). 1.29 (d, *J* = 7.0 Hz, 3H, C11H_3).

^{19}F NMR (470 MHz, $CDCl_3$) δ –62.8.

^{13}C NMR (126 MHz, $CDCl_3$) δ 203.9 (C13), 138.0 (C5), 135.8 (C15), 135.3 (C4), 132.7 (C16), 131.1 (q, *J* = 32.9 Hz, C19), 129.2 (q, *J* = 3.7 Hz, C18), 129.0 (ArC), 128.6 (ArC), 127.2 (ArC), 126.5 (C10), 126.5 (q, *J* = 4.0 Hz, C20), 126.3 (ArC), 123.6 (q, *J* = 273.0 Hz, C21), 81.4 (C1), 39.1 (C6), 33.6 (C2), 25.7 (C3), 14.5 (C11).

$[\alpha]_D^{25}$ = –0.4 (c 1.0, CH_2Cl_2).

HRMS (ESI): calculated for $C_{19}H_{17}F_3O_2Na$ $[M+Na]^+$ requires m/z 357.1078, found m/z 357.1068.

Chiral SFC: DAICEL CHIRALPACK SB column (25 cm), CO_2:*i*-PrOH 98:2, 2.0 mL/min, 165 bar, 40 °C. Retention times: 18.8 mins (major), 20.5 mins (minor), e.r. = 97:3. The racemate was prepared using *rac*-BINAP.

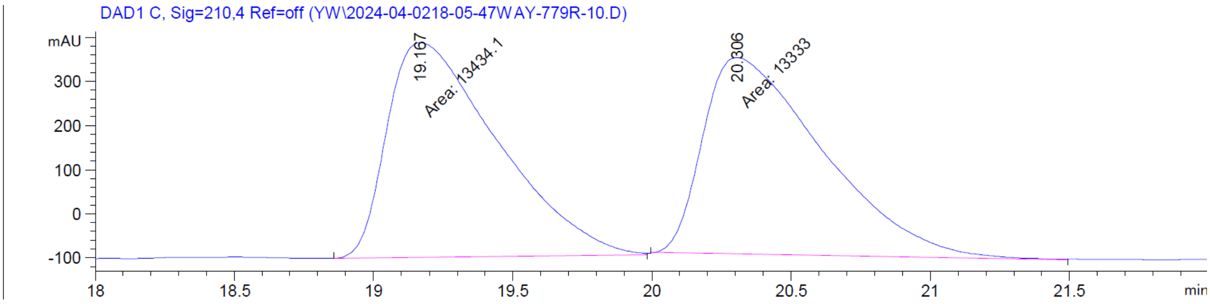

Signal 3: DAD1 C, Sig=210,4 Ref=off

Peak #	RetTime [min]	Type	Width [min]	Area [mAU*s]	Height [mAU]	Area %
1	19.167	MM	0.4602	1.34341e4	486.53769	50.1888
2	20.306	MM	0.4991	1.33330e4	445.27090	49.8112
Totals :				2.67671e4	931.80859	

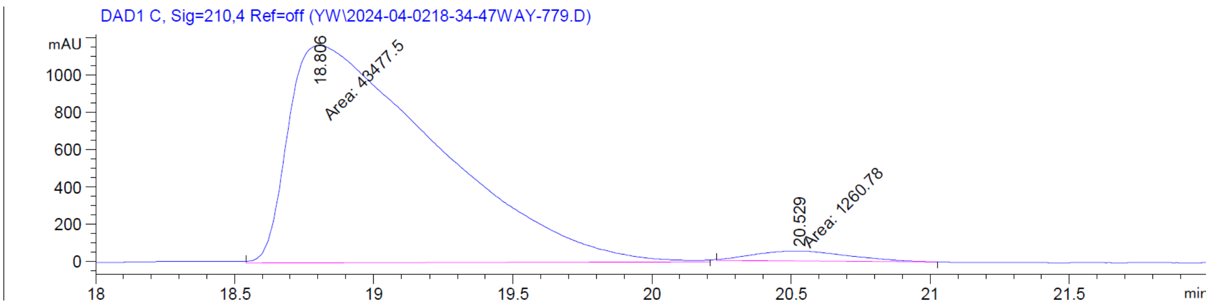

Signal 3: DAD1 C, Sig=210,4 Ref=off

Peak #	RetTime [min]	Type	Width [min]	Area [mAU*s]	Height [mAU]	Area %
1	18.806	MM	0.6220	4.34775e4	1165.00366	97.1819
2	20.529	MM	0.3981	1260.77576	52.78699	2.8181

Totals : 4.47383e4 1217.79065

(1S,2*R*)-1-Methyl-2-(3-(trifluoromethyl)benzoyl)-1,2,3,4-tetrahydronaphthalen-2-yl 3,5-dinitrobenzoate 8p-Bz(NO$_2$)$_2$

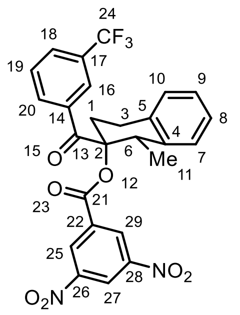

8p-Bz(NO$_2$)$_2$

Et_3N (50 µL, 0.36 mmol) and then 3,5-dinitrobenzoylchloride (24.2 mg, 0.105 mmol) were added to a solution of **8p** (16.7 mg, 0.05 mmol) and 4-(dimethylamino)pyridine (6.7 mg, 0.055 mmol) in CH_2Cl_2 (1 mL) at r.t.. The solution was stirred for 18 h, diluted with EtOAc and washed successively with 1 M aq. HCl, 10% aq. NaOH, saturated aq. $NaHCO_3$ and brine. The solution was dried ($MgSO_4$) and concentrated *in vacuo*. FCC (1:9 to 1:3 EtOAc in hexanes) provided **8p-Bz(NO$_2$)$_2$** (17 mg, 64 %) as colorless crystals.

m.p. = 170–172 °C.

IR (film) ν_{max}/cm^{-1}: 3101, 2974, 1733, 1539, 1159, 718.

^{1}H NMR (500 MHz, $CDCl_3$) δ 9.28 (t, *J* = 2.1 Hz, 1H, C27*H*), 9.12 (d, *J* = 2.1 Hz, 2H, C(25, 29)*H*), 8.31 (t, *J* = 2.1 Hz, 1H, C16*H*), 8.21 (dt, *J* = 8.0 Hz, 1.4 Hz, 1H, C20*H*), 7.76 (d, *J* = 7.8 Hz, 1H, C18*H*), 7.55 (t, *J* = 7.8 Hz, 1H, C19*H*), 7.28 – 7.26 (m, 2H, Ar*H*), 7.21 – 7.18 (m, 1H, Ar*H*), 7.10 – 7.09 (m, 1H, Ar*H*), 4.13 (q, *J* = 7.2 Hz, 1H, C6*H*), 3.06 – 2.98 (m, 1H, C3H_A), 2.83 – 2.76 (m, 3H, C1H_2, C3H_B), 1.42 (d, *J* = 7.2 Hz, 3H, C11H_3).

^{19}F NMR (470 MHz, $CDCl_3$) δ –63.0.

^{13}C NMR (126 MHz, $CDCl_3$) δ 194.9 (C13), 161.5 (C21), 148.8 (C(26, 28)), 139.3 (C5), 134.9 (C14), 132.8 (ArC), 132.7 (ArC), 131.7 (ArC), 131.1 (q, *J* = 32.2 Hz, C17), 129.5 (ArC), 129.4 (q, *J* = 3.7 Hz, C18), 129.4 (ArC), 128.4 (ArC), 128.3 (ArC), 126.8 (ArC), 126.4 (ArC), 125.3 (q, *J* = 3.9 Hz, C16), 123.4 (q, *J* = 272.0 Hz, C24), 123.1 (ArC), 90.5 (C2), 38.9 (C6), 27.8 (C1), 26.2 (C3), 19.2 (C11).

$[\alpha]_D^{25}$ = +142.5 (c 0.5, $CHCl_3$).

HRMS (ESI): a stable ion was not found in ESI and CI.

X-Ray crystallography data of **8p-Bz(NO_2)$_2$**

The structure and the absolute configuration of compound **8p-Bz(NO_2)$_2$** were determined by single crystal X-ray diffraction of crystals grown from CH_2Cl_2/hexane. See CCDC 2478526.

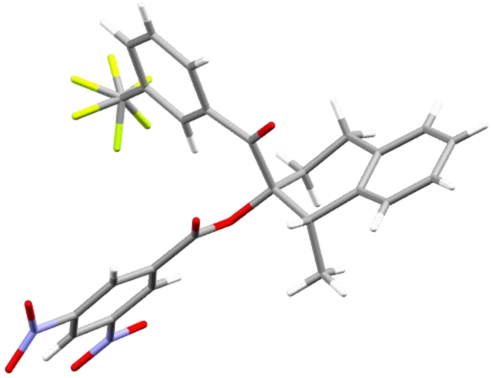

(1*R*,2*S*)-(2-Hydroxy-1-methyl-1,2,3,4-tetrahydronaphthalen-2-yl)(3-methoxyphenyl)methanone 8q

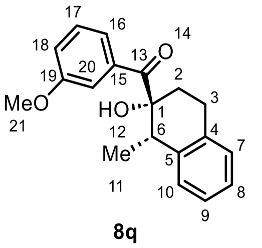

8q

Synthesized from **7q** (29.6 mg) according to *General Procedure J* (60 ˚C, 72 h) with **L6** (*S*)-SynPhos (3.19 mg). Analysis of the ^{1}H NMR spectrum of the crude mixture gave a >20:1 d.r. of **8q**. **8q** (24.0 mg, 81%) was isolated as a colorless oil.

IR (film) ν_{max}/cm^{-1}: 3458, 2936, 1665, 1259, 1035, 960.

^{1}H NMR (500 MHz, $CDCl_3$) δ 7.64 (dt, *J* = 7.8 Hz, 1.1 Hz, 1H, C16*H*), 7.57 (t, *J* = 2.2 Hz, 1H, C20*H*), 7.39 (t, *J* = 8.0 Hz, 1H, C17*H*), 7.29 (d, *J* = 7.2 Hz, 1H, Ar*H*), 7.24 – 7.19 (m, 3H, Ar*H*), 7.14 (dd, *J* = 8.2 Hz, 2.6 Hz, 1H, C18*H*), 4.00 (s, 1H, O*H*), 3.88 (s, 3H, C21H_3), 3.67 (q, *J* = 6.9 Hz, 1H, C6*H*), 3.30 – 3.23 (m, 1H, C3H_A), 2.92 – 2.87 (m, 1H, C3H_B), 2.58 – 2.52 (m, 1H, C2H_A), 2.11 – 2.06 (m, 1H, C2H_B). 1.24 (d, *J* = 6.9 Hz, 3H, C11H_3).

^{13}C NMR (126 MHz, $CDCl_3$) δ 204.9 (C13), 159.7 (C19), 138.4 (C5), 135.8 (C4), 135.8 (C15), 129.5 (C17), 128.6 (ArC), 127.0 (ArC), 126.4 (ArC), 126.0 (ArC), 121.7 (C16), 119.0 (C18), 114.5 (C20), 80.8 (C1), 55.5 (C21), 39.0 (C6), 33.8 (C2), 26.0 (C3), 14.2 (C11).

$[\alpha]_D^{25}$ = –22.9 (c 1.0, CH_2Cl_2).

HRMS (ESI): calculated for $C_{19}H_{20}O_3Na$ $[M+Na]^+$ requires m/z 319.1310, found m/z 319.1310.

Chiral SFC: DAICEL CHIRALCEL OD-H column (25 cm), CO_2:*i*-PrOH 90:10, 2.0 mL/min, 170 bar, 40 °C. Retention times: 9.3 mins (minor), 10.7 mins (major), e.r. = 94:6. The racemate was prepared using *rac*-BINAP.

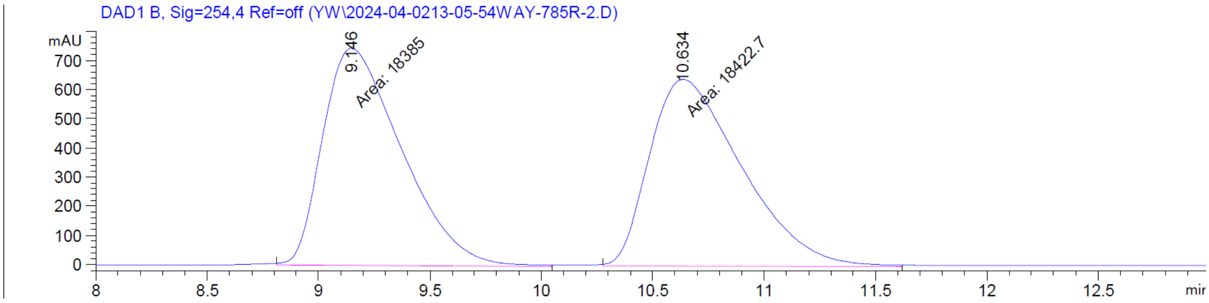

Signal 2: DAD1 B, Sig=254,4 Ref=off

Peak #	RetTime [min]	Type	Width [min]	Area [mAU*s]	Height [mAU]	Area %
1	9.146	MM	0.4111	1.83850e4	745.37457	49.9489
2	10.634	MM	0.4795	1.84227e4	640.30298	50.0511
Totals :				3.68077e4	1385.67755	

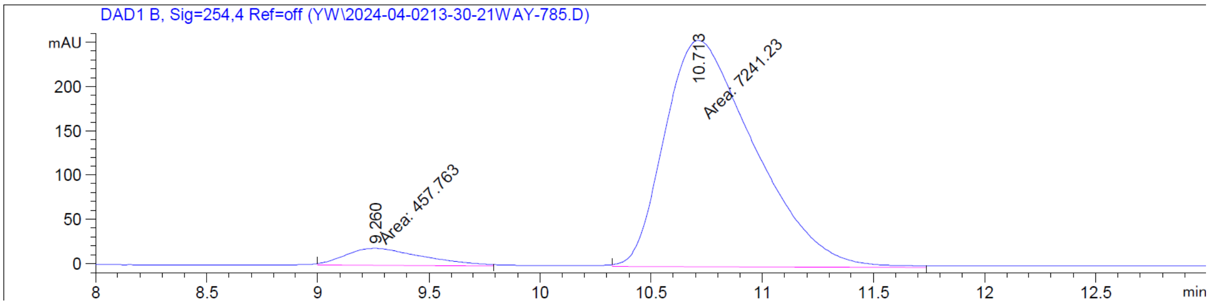

Signal 2: DAD1 B, Sig=254,4 Ref=off

Peak #	RetTime [min]	Type	Width [min]	Area [mAU*s]	Height [mAU]	Area %
1	9.260	MM	0.4012	457.76321	19.01779	5.9458
2	10.713	MM	0.4712	7241.22900	256.10483	94.0542
Totals :				7698.99222	275.12262	

(2-Fluorophenyl)-(1*R*,2*S*)-(2-hydroxy-1-methyl-1,2,3,4-tetrahydronaphthalen-2-yl)methanone 8r

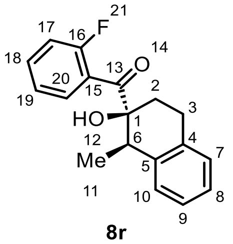

8r

Synthesized from **7r** (28.4 mg) according to *General Procedure J* (70 ˚C, 48 h) with **L30** (*R*)-Tol-SynPhos (3.47 mg). Analysis of the ^{1}H NMR spectrum of the crude mixture gave a >20:1 d.r. of **8r**. **8r** (22.0 mg, 76%) was isolated as a colorless oil.

IR (film) ν_{max}/cm^{-1}: 3493, 2937, 1691, 1448, 1207, 960.

^{1}H NMR (500 MHz, $CDCl_3$) δ 7.51 – 7.46 (m, 2H, Ar*H*), 7.30 (d, *J* = 7.9 Hz, 1H, Ar*H*), 7.24 (t, *J* = 7.6 Hz, 1H, C18*H*), 7.20 – 7.14 (m, 4H, Ar*H*), 3.55 (q, *J* = 7.0 Hz, 1H, C6*H*), 3.33 (d, *J* = 1.6 Hz, 1H, O*H*), 3.22 – 3.15 (m,

1H, C3H_A), 2.87 – 2.81 (m, 1H, C3H_B), 2.27 – 2.21 (m, 1H, C2H_A), 2.17 – 2.13 (m, 1H, C2H_B). 1.36 (d, J = 7.0 Hz, 3H, C11H_3).

^{19}F NMR (470 MHz, $CDCl_3$) δ –110.3.

^{13}C NMR (126 MHz, $CDCl_3$) δ 207.3 (d, J = 1.8 Hz, C13), 158.9 (d, J = 249.6 Hz, C16), 138.1 (C5), 135.2 (C4), 132.5 (d, J = 8.5 Hz, C17), 128.7 (d, J = 3.5 Hz, C18), 128.5 (ArC), 127.2 (ArC), 126.2 (d, J = 17.0 Hz, C15), 126.4 (ArC), 126.0 (ArC), 124.2 (d, J = 3.4 Hz, C20), 116.3 (d, J = 22.4 Hz, C16), 81.7 (C1), 37.7 (C6), 31.8 (d, J = 2.9 Hz, C2), 25.4 (C3), 14.6 (C11).

$[\alpha]_D^{25}$ = +4.2 (c 0.5, CH_2Cl_2).

HRMS (ESI): calculated for $C_{18}H_{17}FO_2Na$ $[M+Na]^+$ requires m/z 307.1110, found m/z 307.1107.

Chiral SFC: YMC Chiral ART Cellulose-SC column (25 cm), CO_2:*i*-PrOH 90:10, 2.0 mL/min, 168 bar, 40 °C. Retention times: 6.2 mins (minor), 6.7 mins (major), e.r. = 95:5. The racemate was prepared using *rac*-BINAP.

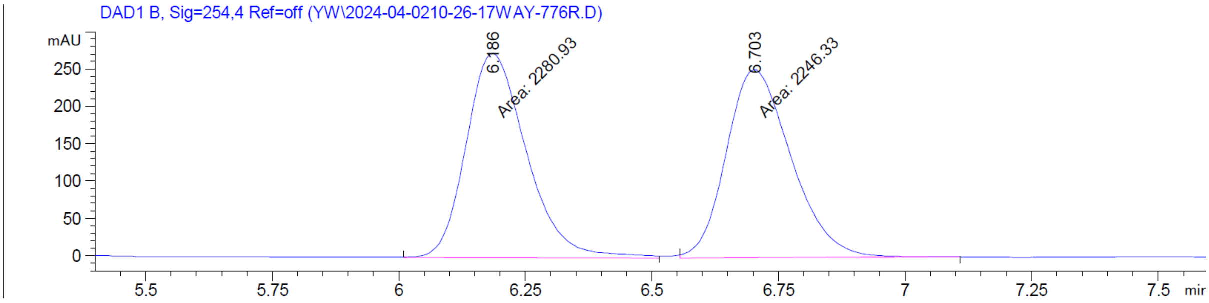

Signal 2: DAD1 B, Sig=254,4 Ref=off

Peak #	RetTime [min]	Type	Width [min]	Area [mAU*s]	Height [mAU]	Area %
1	6.186	MM	0.1387	2280.93262	274.13205	50.3822
2	6.703	MM	0.1484	2246.32983	252.22101	49.6178
Totals :				4527.26245	526.35306	

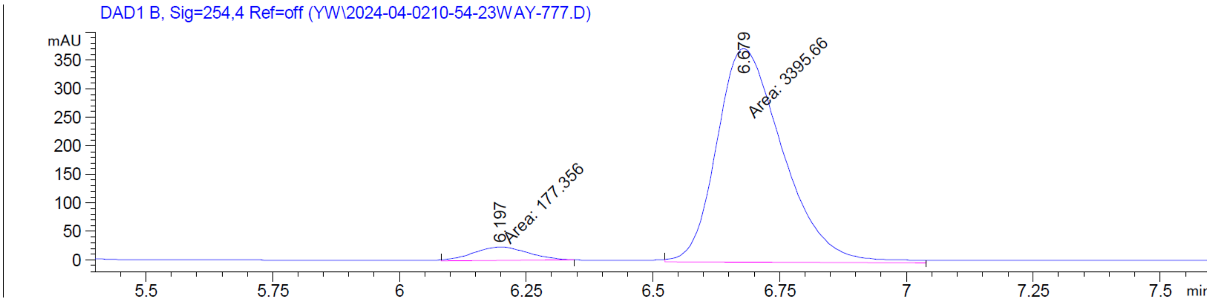

Signal 2: DAD1 B, Sig=254,4 Ref=off

Peak #	RetTime [min]	Type	Width [min]	Area [mAU*s]	Height [mAU]	Area %
1	6.197	MM	0.1251	177.35619	23.62380	4.9638
2	6.679	MM	0.1509	3395.66162	374.95236	95.0362
Totals :				3573.01781	398.57617	

(1*R*,2*S*)-(2-Hydroxy-1-methyl-1,2,3,4-tetrahydronaphthalen-2-yl)(naphthalen-2-yl)methanone 8s

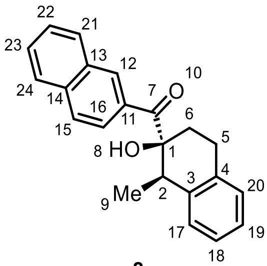

8s

Synthesized from **7s** (31.6 mg) according to *General Procedure J* run at 60 ˚C for 72 h with **L30** (*R*)-Tol-SynPhos (3.47 mg). Analysis of the ^{1}H NMR spectrum of the crude mixture gave a >20:1 d.r. of **8s**. **8s** (26.0 mg, 82%) was isolated as a colorless oil.

IR (film) ν_{max}/cm^{-1}: 3456, 2934, 1659, 1462, 1114, 909.

^{1}H NMR (500 MHz, $CDCl_3$) δ 8.67 (s, 1H, C12*H*), 8.13 (dd, *J* = 8.7 Hz, 1.8 Hz, 1H, C16H), 7.99 – 7.91 (m, 3H, Ar*H*), 7.67 – 7.58 (m, 2H, Ar*H*), 7.34 – 7.32 (m, 1H, Ar*H*), 7.28 – 7.22 (m, 3H, Ar*H*), 4.13 (s, 1H, O*H*), 3.82 (q, *J* = 6.9 Hz, 1H, C2*H*), 3.34 (ddd, *J* = 16.5 Hz, 10.8 Hz, 5.8 Hz, 1H, C5H_A), 2.96 (ddd, *J* = 16.5 Hz, 5.9 Hz, 3.9 Hz, 1H, C5H_B), 2.70 (ddd, *J* = 13.3 Hz, 10.8 Hz, 5.9 Hz, 1H, C6H_A), 2.19 (ddd, *J* = 13.3 Hz, 5.8 Hz, 3.9 Hz, 1H, C6H_B). 1.29 (d, *J* = 6.9 Hz, 3H, C9H_3).

^{13}C NMR (126 MHz, $CDCl_3$) δ 204.8 (C7), 138.5 (C3), 135.9 (C4), 135.4 (C11), 132.3 (ArC), 131.6 (ArC), 131.2 (C12), 129.7 (ArC), 128.8 (ArC), 128.7 (ArC), 128.4 (ArC), 127.7 (ArC), 127.1 (ArC), 127.0 (ArC), 126.4 (ArC), 126.1 (ArC), 125.3 (ArC), 80.8 (C1), 39.3 (C2), 34.0 (C6), 26.1 (C5), 14.3 (C9).

$[\alpha]_D^{25}$ = +16.6 (c 1.0, CH_2Cl_2).

HRMS (ESI): calculated for $C_{22}H_{20}O_2Na$ $[M+Na]^+$ requires m/z 339.1361, found m/z 339.1365.

Chiral SFC: YMC Chiral ART Cellulose-SC column (25 cm), CO_2:*i*-PrOH 90:10, 2.0 mL/min, 164 bar, 40 °C. Retention times: 16.1 mins (minor), 16.7 mins (major), e.r. = 92:8. The racemate was prepared using *rac*-BINAP.

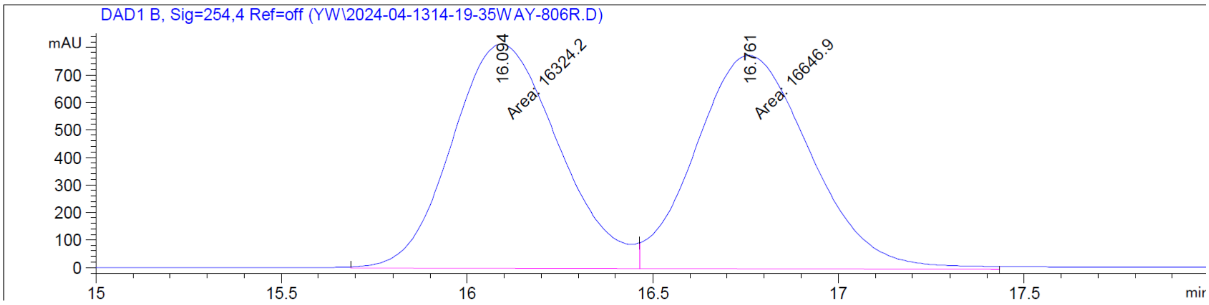

Signal 2: DAD1 B, Sig=254,4 Ref=off

Peak #	RetTime [min]	Type	Width [min]	Area [mAU*s]	Height [mAU]	Area %
1	16.094	MF	0.3342	1.63242e4	814.17413	49.5108
2	16.761	FM	0.3582	1.66469e4	774.58008	50.4892
Totals :				3.29711e4	1588.75421	

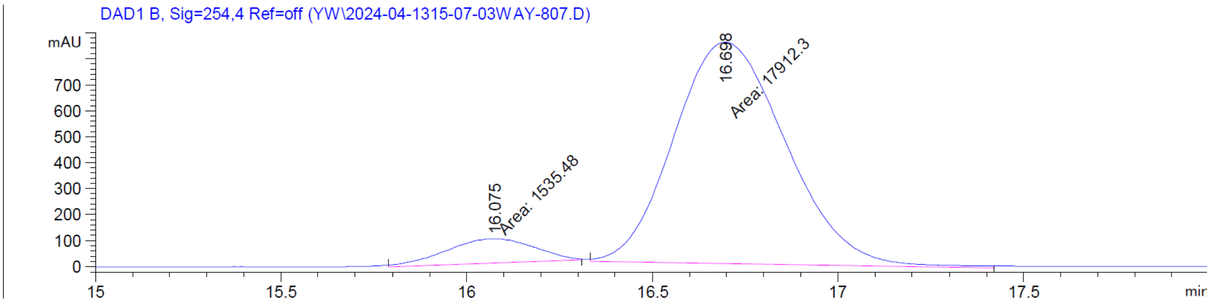

Signal 2: DAD1 B, Sig=254,4 Ref=off

Peak #	RetTime [min]	Type	Width [min]	Area [mAU*s]	Height [mAU]	Area %
1	16.075	MM	0.2745	1535.47668	93.23345	7.8954
2	16.698	MM	0.3501	1.79123e4	852.68054	92.1046
Totals :				1.94478e4	945.91399	

(Furan-2-yl)-(1*R*,2*S*)-(2-Hydroxy-1-methyl-1,2,3,4-tetrahydronaphthalen-2-yl)methanone 8t

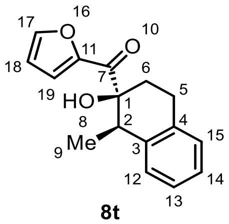

8t

8t was synthesized from **7t** (25.6 mg) according to *General Procedure J* (70 ˚C, 72 h) with **L31** (*R*)-DM-SynPhos (3.75 mg). Analysis of the ^{1}H NMR spectrum of the crude mixture gave a >20:1 d.r. of **8t**. **8t** (18.0 mg, 70%) was isolated as a colorless solid.

m.p. = 98–101 °C.

IR (film) ν_{max}/cm^{-1}: 3472, 2929, 1649, 1458, 1295, 1021, 963, 764.

^{1}H NMR (500 MHz, $CDCl_3$) δ 7.64 (t, *J* = 0.7 Hz, 1H, C19*H*), 7.51 (d, *J* = 3.6 Hz, 1H, C17*H*), 7.31 (d, *J* = 7.6 Hz, 1H, Ar*H*), 7.25 – 7.19 (m, 3H, Ar*H*), 6.63 (dd, *J* = 3.6 Hz, 1.5 Hz, 1H, C18*H*), 4.07 (s, 1H, O*H*), 3.77 (q, *J* = 7.0 Hz, 1H, C2*H*), 3.27 (ddd, *J* = 17.0 Hz, 11.6 Hz, 5.6 Hz, 1H, C5H_A), 2.90 (ddd, *J* = 17.0 Hz, 5.9 Hz, 3.0 Hz, 1H, C5H_B), 2.64 (ddd, *J* = 13.0 Hz, 11.6 Hz, 5.9 Hz, 1H, C6H_A), 1.95 (ddd, *J* = 13.0 Hz, 5.6 Hz, 3.0 Hz, 1H, C6H_B). 1.22 (d, *J* = 7.0 Hz, 3H, C9H_3).

^{13}C NMR (126 MHz, $CDCl_3$) δ 192.4 (C7), 150.4 (C11), 146.8 (C19), 138.4 (C3), 135.8 (C4), 128.6 (ArC), 126.8 (ArC), 126.3 (ArC), 125.9 (ArC), 120.9 (C17), 112.6 (C18), 79.4 (C1), 38.1 (C2), 32.7 (C6), 25.8 (C5), 13.8 (C9).

$[\alpha]_D^{25}$ = +39.1, (c 1.0, CH_2Cl_2).

HRMS (ESI): calculated for $C_{16}H_{17}O_3$ $[M+H]^+$ requires m/z 257.1178, found m/z 257.1174.

Chiral SFC: YMC Chiral ART Cellulose-SC column (25 cm), CO_2:*i*-PrOH 95:5, 2.0 mL/min, 161 bar, 40 °C. Retention times: 18.2 mins (minor), 18.8 mins (major), e.r. = 92:8. The racemate was prepared using *rac*-BINAP.

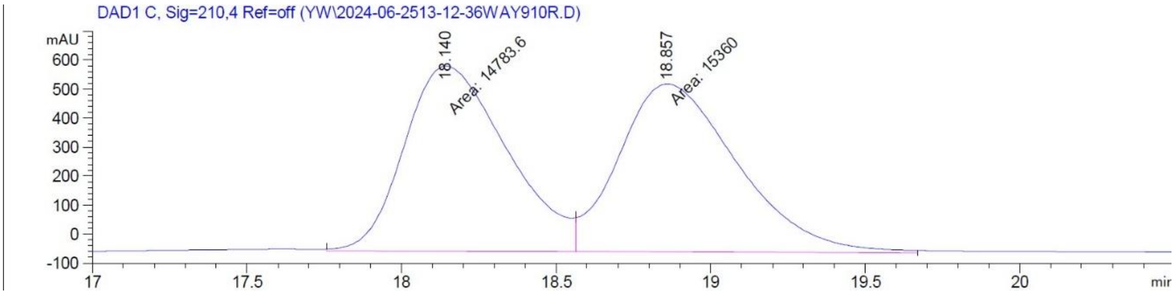

Signal 3: DAD1 C, Sig=210,4 Ref=off

Peak #	RetTime [min]	Type	Width [min]	Area [mAU*s]	Height [mAU]	Area %
1	18.140	MF	0.3862	1.47836e4	637.91748	49.0440
2	18.857	FM	0.4425	1.53600e4	578.57391	50.9560
Totals :				3.01436e4	1216.49139	

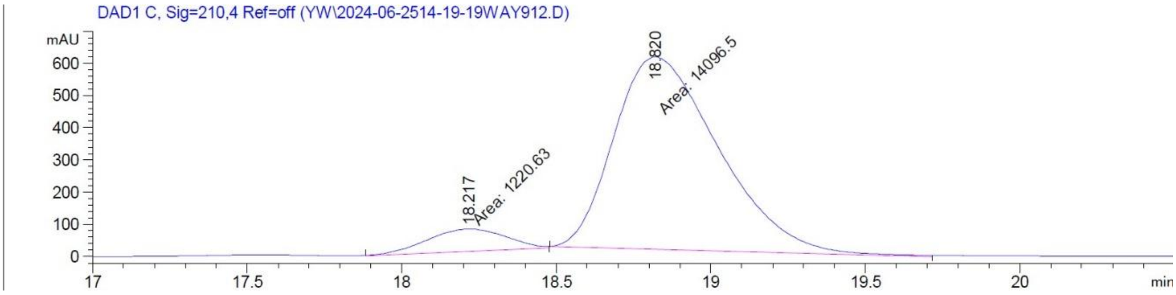

Signal 3: DAD1 C, Sig=210,4 Ref=off

Peak #	RetTime [min]	Type	Width [min]	Area [mAU*s]	Height [mAU]	Area %
1	18.217	MM	0.2949	1220.62512	68.97713	7.9690
2	18.820	MM	0.3933	1.40965e4	597.38104	92.0310
Totals :				1.53171e4	666.35817	

X-Ray crystallography data of 8t

The structure and the relative configuration of compound **8t** were determined by single crystal X-ray diffraction of crystals grown from MeOH/hexane. See CCDC 2478527.

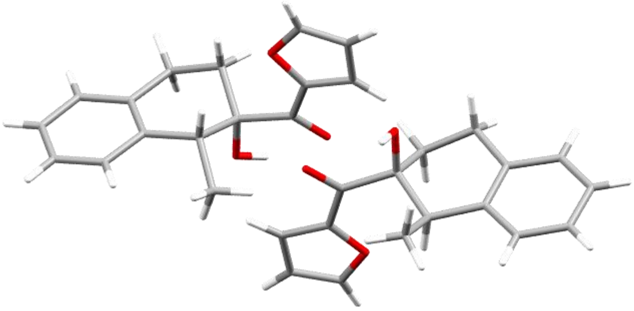

(*trans)*-(2-Hydroxy-1-methyl-1,2,3,4-tetrahydronaphthalen-2-yl)(3-(trifluoromethyl)phenyl)methanone 12

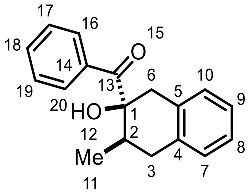

12

Synthesized from **11** (26.6 mg) according to *General Procedure J* (70 ˚C, 24 h). Analysis of the ^{1}H NMR spectrum of the crude mixture gave a >20:1 d.r. of **12**. **12** (20.0 mg, 75%) was isolated as a colorless oil.

IR (film) ν_{max}/cm^{-1}: 3348, 2974, 1664, 1380, 1046, 879.

^{1}H NMR (500 MHz, $CDCl_3$) δ 8.08 – 8.06 (m, 2H, C(16, 20)*H*), 7.63 – 7.60 (m, 1H, C18*H*), 7.49 (t, *J* = 7.8 Hz, 2H, C(17, 19)*H*), 7.20 – 7.16 (m, 3H, C(7, 8, 9)*H*), 7.10 – 7.08 (m, 1H, C10*H*), 4.36 (s, 1H, O*H*), 3.69 (d, *J* = 17.2 Hz, 1H, C6H_A), 3.01 – 2.94 (m, 2H, C6H_B, C3H_A), 2.83 – 2.72 (m, 2H, C3H_B, C2*H*), 0.93 (d, *J* = 6.4 Hz, 3H, C11H_3).

^{13}C NMR (126 MHz, $CDCl_3$) δ 204.5 (C13), 136.2 (C14), 134.0 (C4), 133.3 (C18), 132.6 (C5), 129.4 (C(16, 20)), 129.1 (C10), 128.7 (C7), 128.7 (C8), 126.1 (C9), 80.2 (C1), 41.2 (C6), 35.9 (C2), 34.2 (C3), 15.2 (C11).

HRMS (ESI): calculated for $C_{18}H_{18}O_2Na$ $[M+Na]^+$ requires m/z 289.1204, found m/z 289.1202.

$[\alpha]_D^{25}$ = +0.4 (c 1.0, CH_2Cl_2).

Chiral SFC: YMC Chiral ART Cellulose-SC column (25 cm), CO_2:*i*-PrOH 80:20, 2.0 mL/min, 168 bar, 40 °C. Retention times: 3.9 mins (minor), 6.1 mins (major), e.r. = 51:49. The racemate was prepared using *rac*-BINAP.

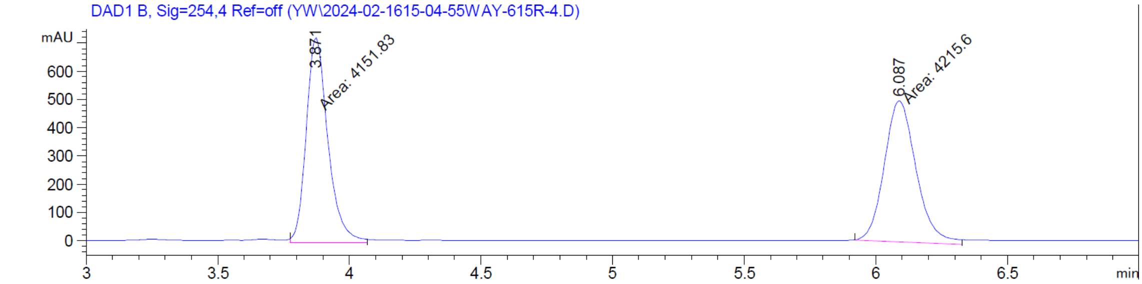

Signal 2: DAD1 B, Sig=254,4 Ref=off

Peak #	RetTime [min]	Type	Width [min]	Area [mAU*s]	Height [mAU]	Area %
1	3.871	MM	0.0952	4151.82568	726.63647	49.6189
2	6.087	MM	0.1404	4215.60156	500.57639	50.3811
Totals :				8367.42725	1227.21286	

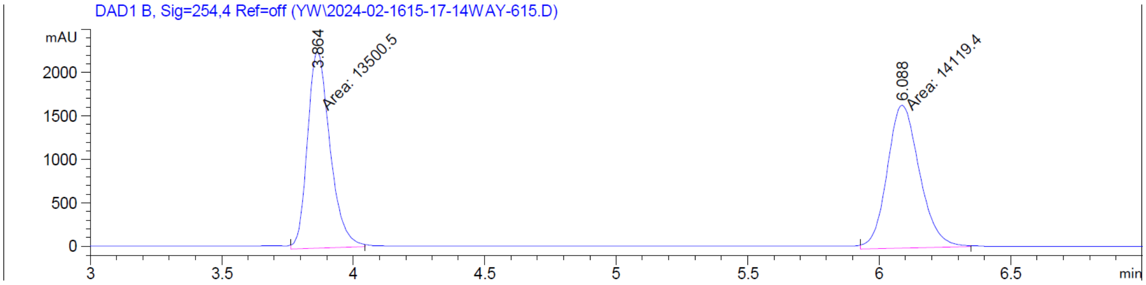

Signal 2: DAD1 B, Sig=254,4 Ref=off

Peak #	RetTime [min]	Type	Width [min]	Area [mAU*s]	Height [mAU]	Area %
1	3.864	MM	0.0993	1.35005e4	2266.90088	48.8795
2	6.088	MM	0.1430	1.41194e4	1645.91992	51.1205
Totals :				2.76199e4	3912.82080	

(*trans*)-2-Hydroxy-3-methyl-3,4-dihydronaphthalen-1(2*H*)-one 14

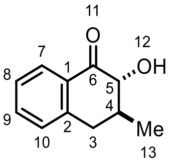

14

Synthesized from **13** (17.6 mg) according to *General Procedure J* (60 ˚C, 14 h). Analysis of the ^{1}H NMR spectrum of the crude mixture gave a 3.4:1 d.r. **14** (9.2 mg, 52%) was isolated as a yellow solid.

m.p. = 53–55 °C.

IR (film) ν_{max}/cm^{-1}: 3387, 2972, 1687, 1458, 1047, 879.

^{1}H NMR (500 MHz, $CDCl_3$) δ 8.05 (d, *J* = 7.9 Hz, 1H, C7*H*), 7.55 (td, *J* = 7.5 Hz, 1.2Hz, 1H, C9*H*), 7.37 (t, *J* = 7.6 Hz, 1H, C8*H*), 7.28 (d, *J* = 7.6 Hz, 1H, C10*H*), 4.03 (dd, *J* = 12.4 Hz, 2.0 Hz, 1H, C5*H*), 3.94 (d, *J* = 2.0 Hz, 1H, O*H*), 3.03 (dd, *J* = 16.9 Hz, 4.4 Hz, 1H, C3H_A), 2.86 (dd, *J* = 16.9 Hz, 11.9 Hz, 1H, C3H_B), 2.28 – 2.18 (m, 1H, C4*H*). 1.35 (d, *J* = 6.3 Hz, 3H, C13*H*).

^{13}C NMR (126 MHz, $CDCl_3$) δ 199.5 (C6), 143.7 (C2), 134.3 (C9), 130.2 (C1), 128.7 (C10), 131.7 (C3), 127.6 (C7), 126.9 (C8), 79.0 (C5), 38.6 (C4), 36.6 (C3), 18.5 (C13).

HRMS (ESI): calculated for $C_{11}H_{13}O_2$ $[M+H]^+$ requires m/z 177.0916, found m/z 177.0912.

$[\alpha]_D^{25}$ = –4.7 (c 0.5, CH_2Cl_2).

Chiral SFC: YMC Chiral ART Cellulose-SC column (25 cm), CO_2:*i*-PrOH 90:10, 2.0 mL/min, 168 bar, 40 °C. Retention times: 5.0 mins (major), 5.5 mins (minor), e.r. = 66:34. The racemate was prepared using *rac*-BINAP.

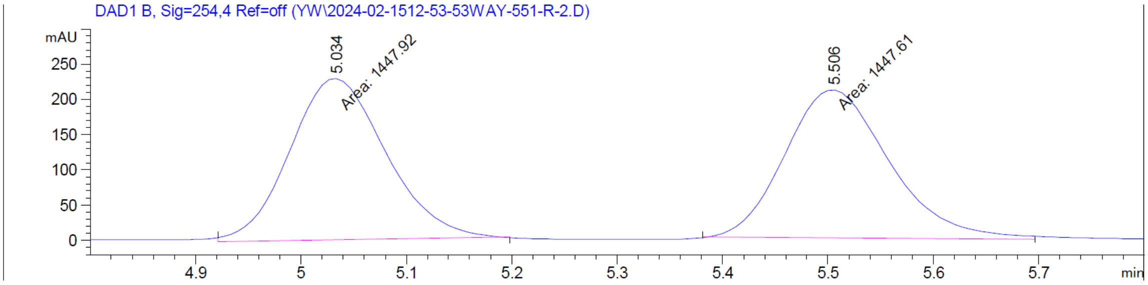

Signal 2: DAD1 B, Sig=254,4 Ref=off

Peak #	RetTime [min]	Type	Width [min]	Area [mAU*s]	Height [mAU]	Area %
1	5.034	MM	0.1052	1447.91919	229.44438	50.0054
2	5.506	MM	0.1146	1447.60559	210.50700	49.9946

Totals : 2895.52478 439.95139

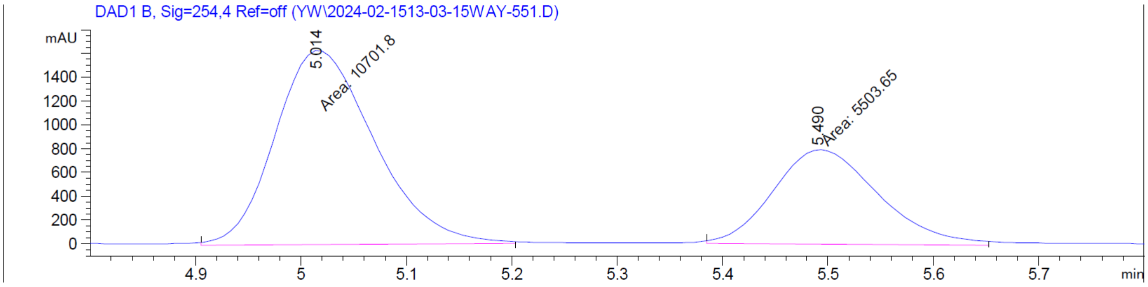

Signal 2: DAD1 B, Sig=254,4 Ref=off

Peak #	RetTime [min]	Type	Width [min]	Area [mAU*s]	Height [mAU]	Area %
1	5.014	MM	0.1091	1.07018e4	1635.58508	66.0383
2	5.490	MM	0.1155	5503.64795	794.45575	33.9617

Totals : 1.62054e4 2430.04083

4-Methyl-3-phenylnaphthalen-2-ol 17 and 1-Methyl-3-phenylnaphthalen-2-ol *iso*-17

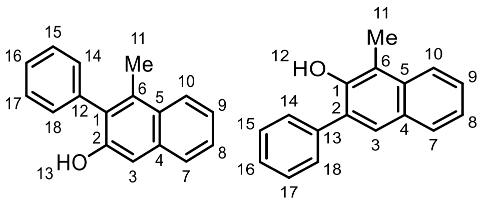

17 ***iso*-17**

Synthesized from **15** (25.2 mg) according to *General Procedure I* (90 °C, 16 h). Analysis of the ^{1}H NMR spectrum of the crude mixture gave a 3:1 mixture of **17**:***iso*-17**. **17** (15.0 mg, 63%) was isolated as an orange solid. Continued elution provided ***iso*-17** (4.9 mg, 21%) as a yellow solid.

Data for **17**:

m.p. = 85–87 °C.

IR (film) ν_{max}/cm^{-1}: 3495, 3065, 1629, 1426, 1230, 1010.

^{1}H NMR (500 MHz, $CDCl_3$) δ 8.00 (d, *J* = 8.4 Hz, 1H, C7*H*), 7.77 (dd, *J* = 8.2, 1.3 Hz, 1H, C10*H*), 7.60 – 7.47 (m, 4H, Ar*H*), 7.44 – 7.37 (m, 3H, Ar*H*), 7.28 (s, 1H, C3*H*), 4.83 (s, 1H, O*H*), 2.46 (s, 3H, C11*H₃*).

^{13}C NMR (126 MHz, $CDCl_3$) δ 150.9 (C2), 135.8 (C12), 134.3 (C4), 133.5 (C6), 130.6 (C15, C17), 130.0 (C1), 129.4 (C14, C18), 128.4 (C16), 128.1 (C5), 127.2 (C7), 126.3 (C8), 124.7 (C10), 123.7 (C9), 107.8 (C3), 16.6 (C11). *The regiochemistry was assigned using HMBC analysis; a correlation was observed from OH to C1, C2 and C3.*

HRMS (ESI): calculated for $C_{17}H_{15}O$ [M+H]$^+$ requires m/z 235.1123, found m/z 235.1118.

Data for ***iso*-17**:

^{1}H NMR (500 MHz, $CDCl_3$) δ 7.98 (d, *J* = 8.5 Hz, 1H, C7*H*), 7.81 (d, *J* = 7.9, 1H, C10*H*), 7.63 (s, 1H, C3*H*), 7.58 – 7.49 (m, 6H, Ar*H*), 7.38 (dd, *J* = 8.7, *J* = 6.9, 1H, Ar*H*), 5.35 (s, 1H, O*H*), 2.64 (s, 3H, C11*H₃*). Analytical data are consistent with the literature.[25]

Limitations

Further unsuccessful substrates.

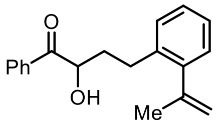

7v

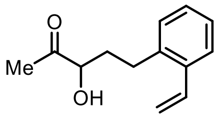

7u

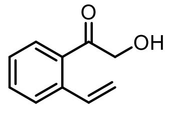

s20

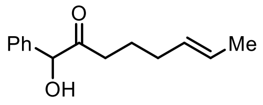

s30

Derivatizations

3-Methyl-1-phenylcyclohexane-1,2-diol s31

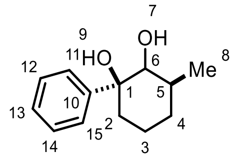

s31

Synthesized from ***iso*-6a** (61.3 mg) following *General Procedure K.* **s31** (>20:1 d.r., 54.5 mg, 89%) was isolated as a colorless solid. The relative configuration of the new stereocentre at C6 was not assigned.

m.p. = 94–98 °C.

IR (film) ν_{max}/cm^{-1}: 3602, 3296, 2927, 1447, 1050, 972.

^{1}H NMR (500 MHz, $CDCl_3$) δ 7.77 (d, J = 7.6 Hz, 2H, C(11, 15)H), 7.37 (t, J = 7.6, 2H, C(12, 14)H), 7.31 – 7.28 (m, 1H, C13H), 3.51 – 3.47 (m, 1H, C6H), 2.58 (s, 1H, OH), 2.38 (d, J = 13.1 Hz, 1H, C2H_A), 2.25 (s, 1H, OH), 2.08 – 2.00 (m, 1H, C5H), 1.79 – 1.61 (m, 3H, C2H_B, C3H_A, C4H_A), 1.32 – 1.15 (m, 2H, C3H_B, C4H_B), 1.12 (dd, J = 6.5 Hz, 1.7 Hz, 3H, C8H_3).

^{13}C NMR (126 MHz, $CDCl_3$) δ 143.2 (C10), 128.2 (C(12, 14)), 128.1 (C(11, 15)), 127.4 (C13), 84.0 (C6), 39.0 (C2), 35.8 (C5), 33.3 (C4), 21.8 (C3), 18.9 (C8).

HRMS (ESI): calculated for $C_{13}H_{18}O_2Na$ $[M+Na]^+$ requires m/z 229.1204, found m/z 229.1199.

(1*R*,2*S*)-2-(Hydroxy(phenyl)methyl)-1-methyl-1,2,3,4-tetrahydronaphthalen-2-ol s32-major and s32-minor

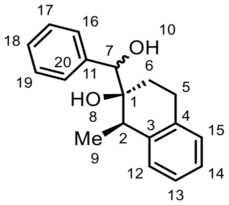

s32

Synthesized from **8m** (79.9 mg) following *General Procedure K.* Analysis of the ^{1}H NMR spectrum of the crude mixture gave an 8:1 d.r. of **s32-major**:**s32-minor**. The relative configuration of the new stereocenter was not assigned. **s32-major** (53.9 mg, 67%) was isolated as a colorless oil. Continued elution provided **s32-minor** (8.0 mg, 10%) as a colorless solid.

Data for **s32-major**:

IR (film) ν_{max}/cm^{-1}: 3380, 2956, 1403, 1043, 978, 700.

^{1}H NMR (500 MHz, $CDCl_3$) δ 7.36 – 7.32 (m, 3H, ArH), 7.18 – 7.15 (m, 5H, ArH), 6.98 – 6.96 (m, 1H, ArH), 4.63 (d, J = 5.3 Hz, 1H, C7H), 3.08 – 2.98 (m, 2H, C5H_2), 2.53 (q, J = 7.1 Hz, 1H, C2H), 2.46 (d, J = 5.3 Hz, 1H, O10H), 2.28 – 2.23 (m, 1H, C6H_A), 2.09 – 2.03 (m, 1H, C6H_B), 2.03 (s, 1H, O8H), 1.18 (d, J = 7.1 Hz, 3H, C9H_3).

^{13}C NMR (126 MHz, $CDCl_3$) δ 141.0 (C11), 140.3 (C3), 134.4 (C4), 129.4 (ArC), 128.9 (ArC), 128.1 (C(17, 19)), 128.0 (ArC), 127.8 (C(16, 20)), 126.1 (ArC), 126.1 (ArC), 75.1 (C(1, 7)), 39.9 (C2), 27.0 (C5), 26.4 (C6), 19.3 (C9).

$[\alpha]_D^{25}$ = −33.6 (c 1.0, CH_2Cl_2).

HRMS (ESI): calculated for $C_{18}H_{20}O_2Na$ $[M+Na]^+$ requires m/z 291.1361, found m/z 291.1355.

Chiral SFC: DAICEL CHIRALCEL IE column (25 cm), CO_2:*i*-PrOH 90:10, 2.0 mL/min, 168 bar, 40 °C. Retention times: 12.9 mins (major), 13.9 mins (minor), e.r. = 96:4. The racemate was prepared from ***rac*-8m**, which was synthesized using *rac*-BINAP.

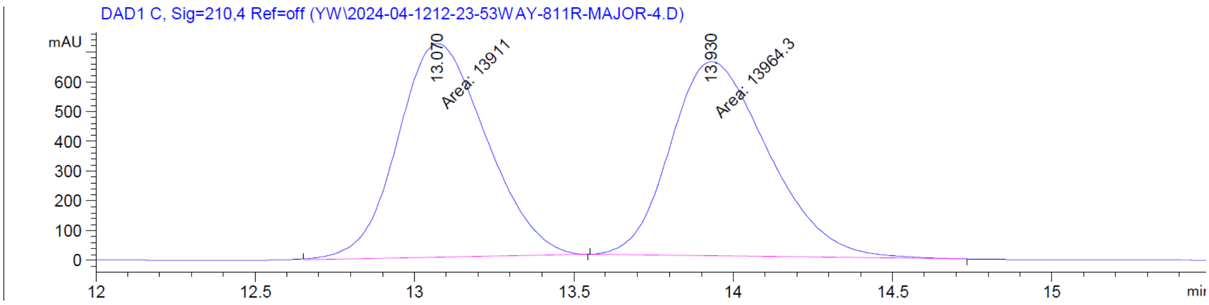

Signal 3: DAD1 C, Sig=210,4 Ref=off

Peak #	RetTime [min]	Type	Width [min]	Area [mAU*s]	Height [mAU]	Area %
1	13.070	MM	0.3234	1.39110e4	716.93018	49.9043
2	13.930	MM	0.3570	1.39643e4	651.93964	50.0957
Totals :				2.78753e4	1368.86981	

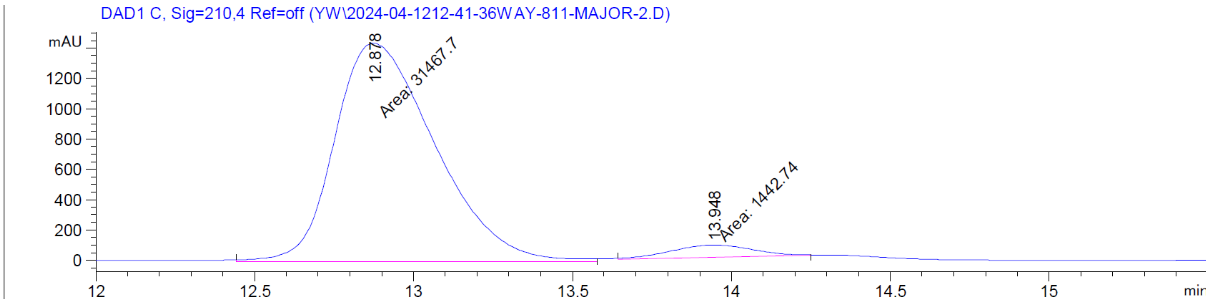

Signal 3: DAD1 C, Sig=210,4 Ref=off

Peak #	RetTime [min]	Type	Width [min]	Area [mAU*s]	Height [mAU]	Area %
1	12.878	MM	0.3643	3.14677e4	1439.58789	95.6162
2	13.948	MM	0.2985	1442.74048	80.55667	4.3838
Totals :				3.29104e4	1520.14456	

Data for **s32-minor**:

m.p. = 85–87 °C.

IR (film) ν_{max}/cm^{-1}: 3456, 3290, 2944, 1392, 1033, 756.

^{1}H NMR (500 MHz, $CDCl_3$) δ 7.49 – 7.47 (m, 2H, C(16, 20)*H*), 7.39 – 7.32 (m, 3H, Ar*H*), 7.24 – 7.11 (m, 4H, Ar*H*), 4.64 (d, *J* = 3.6 Hz, 1H, C7*H*), 3.35 (q, *J* = 7.2 Hz, 1H, C2*H*), 2.90 – 2.71 (m, 2H, C5H_2), 2.45 (s, 1H, O8*H*), 2.41 (d, *J* = 3.6 Hz, 1H, O10*H*), 2.08 – 2.02 (m, 1H, C6H_A), 1.40 (d, *J* = 7.2 Hz, 3H, C9H_3), 1.34 – 1.29 (m, 1H, C6H_B).

^{13}C NMR (126 MHz, $CDCl_3$) δ 141.0 (C11), 140.6 (C3), 134.4 (C4), 129.5 (ArC), 128.6 (ArC), 128.3 (C(17, 19)), 128.2 (ArC), 127.9 (C(16, 20)), 126.1 (ArC), 125.9 (ArC), 76.2 (C(1, 7)), 39.3 (C2), 26.1 (C5), 25.9 (C6), 18.5 (C9).

$[\alpha]_D^{25}$ = −2.8 (c 0.2, CH_2Cl_2).

HRMS (ESI): calculated for $C_{18}H_{20}O_2Na$ $[M+Na]^+$ requires m/z 291.1361, found m/z 291.1355.

Chiral SFC: DAICEL CHIRALCEL IE column (25 cm), CO_2:*i*-PrOH 90:10, 2.0 mL/min, 168 bar, 40 °C. Retention times: 14.0 mins (major), 19.2 mins (minor), e.r. = 95:5. The racemate was prepared from ***rac*-8m**, which was synthesized using *rac*-BINAP.

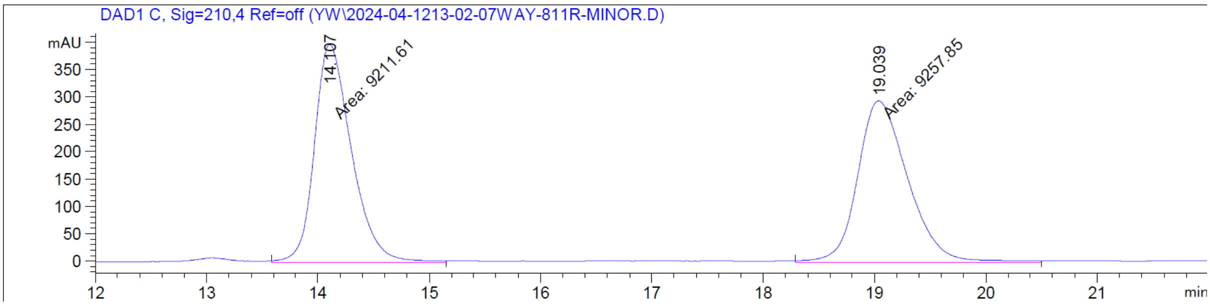

Signal 3: DAD1 C, Sig=210,4 Ref=off

Peak #	RetTime [min]	Type	Width [min]	Area [mAU*s]	Height [mAU]	Area %
1	14.107	MM	0.3858	9211.60742	397.95801	49.8748
2	19.039	MM	0.5228	9257.84668	295.13058	50.1252
Totals :				1.84695e4	693.08859	

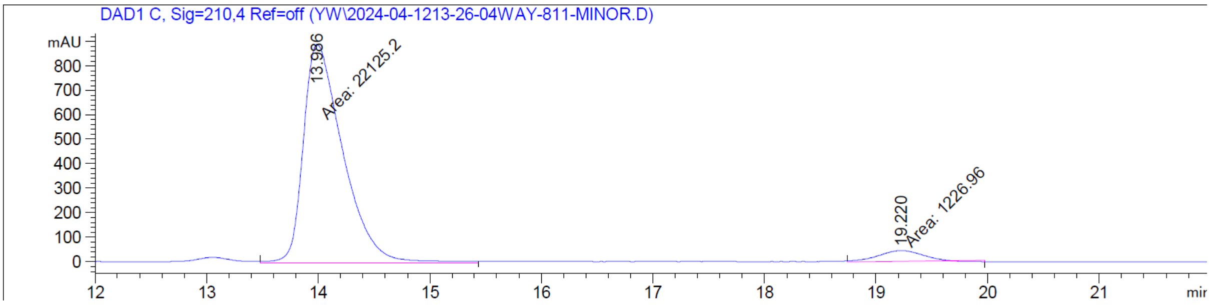

Signal 3: DAD1 C, Sig=210,4 Ref=off

Peak #	RetTime [min]	Type	Width [min]	Area [mAU*s]	Height [mAU]	Area %
1	13.986	MM	0.4123	2.21252e4	894.41376	94.7458
2	19.220	MM	0.4632	1226.96228	44.14548	5.2542
Totals :				2.33521e4	938.55923	

(*E*)-(*trans*)-2-Hydroxy-1-methyl-1,2,3,4-tetrahydronaphthalen-2-yl)(phenyl)methanone oxime s33

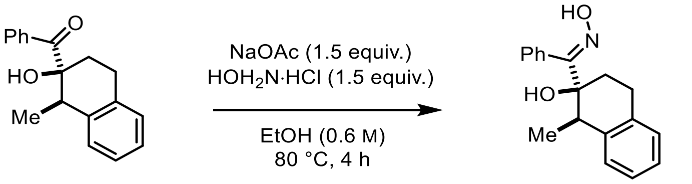

8m, >20:1 dr

s33, 62%, >20:1 dr
E/*Z* = 14/1

Following a modified literature procedure,[26] a solution of **8m** (53 mg, 0.2 mmol, 1.0 equiv.), sodium acetate (25 mg, 0.3 mmol, 1.5 equiv.), and hydroxylamine hydrochloride (21 mg, 0.3 mmol, 1.5 equiv.) in EtOH (0.3 mL) were heated at 80 °C for 4 h. The mixture was cooled to r.t. and filtered. The filtrate was diluted with CH_2Cl_2 (1

mL), washed with H_2O, dried ($MgSO_4$), and concentrated *in vacuo*. The crude mixture was purified by FCC (2:1 hexane/Et_2O) to obtain **s33** (35 mg, 62%, *E*/*Z* = 14/1, >20:1 dr) as a colorless solid.

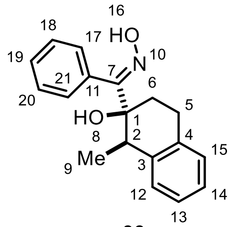

s33

m.p. = 140–144 °C.

IR (film) ν_{max}/cm^{-1}: 3341, 2932, 1345, 1011, 954, 707.

^{1}H NMR (500 MHz, $CDCl_3$) δ 7.47 – 7.41 (m, 3H, Ar*H*), 7.24 – 7.23 (m, 3H, Ar*H*), 7.17 – 7.10 (m, 3H, Ar*H*), 3.25 – 3.19 (m, 1H, C5*H$_A$*), 3.15 (q, *J* = 6.9 Hz, 1H, C2*H*), 2.78 (ddd, *J* = 16.8 Hz, 5.8 Hz, 3.7 Hz, 1H, C5*H$_B$*), 2.13 – 2.01 (m, 2H, C6*H$_2$*), 1.46 (d, *J* = 6.9 Hz, 3H, C9*H$_3$*).

^{13}C NMR (126 MHz, $CDCl_3$) δ 162.8 (C7), 138.9 (C3), 125.2 (C4), 131.7 (C11), 128.9 (ArC), 128.6 (ArC), 128.4 (C(18, 20)), 127.8 (ArC), 127.6 (C(17, 21)), 126.1 (ArC), 125.7 (ArC), 75.9 (C1), 37.7 (C2), 32.6 (C6), 25.7 (C5), 14.9 (C9).

HRMS (ESI): calculated for $C_{18}H_{19}NO_2Na$ $[M+Na]^+$ requires m/z 304.1313, found m/z 304.1313.

((1*R*,2*S*)-2-(Benzyloxy)-1-methyl-1,2,3,4-tetrahydronaphthalen-2-yl)(phenyl)methanone Bn-8m

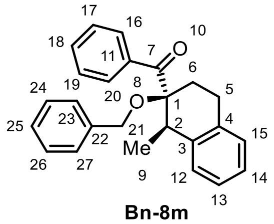

Bn-8m

Following a modified literature procedure,[27] tertiary alcohol **8m** (32.0 mg, 0.12 mmol) was dissolved in DMF (0.3 mL), and then NaH (8.7 mg, 60% in oil, 0.36 mmol, 3.0 equiv.) was added portion-wise at 0 °. After 10 minutes, BnBr (43 μL, 0.36 mmol, 3.0 equiv.) was added dropwise and the reaction was stirred at r.t. for 10 minutes before being quenched by ice. The mixture was diluted with hexane and washed with brine. The organic phase was dried ($MgSO_4$) and concentrated *in vacuo* to give the crude product. This was purified by FCC (50:1 to 20:1 hexane/Et_2O) to give the benzyl protected alcohol **Bn-8m** (39 mg, 91%, >20:1 d.r., 94:6 e.r.) as a colorless oil.

IR (film) ν_{max}/cm^{-1}: 2971, 1680, 1447, 1174, 1069, 698.

^{1}H NMR (500 MHz, $CDCl_3$) δ 8.28 (d, *J* = 7.6 Hz, 2H, C(16, 20)*H*), 7.57 (t, *J* = 7.3 Hz, 1H, C18*H*), 7.45 – 7.32 (m, 7H, Ar*H*), 7.19 – 7.10 (m, 3H, Ar*H*), 7.01 (d, *J* = 7.6 Hz, 1H, C15*H*), 4.65 (d, *J* = 10.5 Hz, 1H, C21*H$_A$*), 4.43 (d, *J* = 10.5 Hz, 1H, C21*H$_B$*), 3.89 (q, *J* = 7.1 Hz, 1H, C2*H*), 2.89 – 2.83 (m, 1H, C5*H$_A$*), 2.63 – 2.55 (m, 2H, C6*H$_2$*), 2.40 – 2.33 (m, 1H, C5*H$_B$*), 1.41 (d, *J* = 7.1 Hz, 3H, C9*H$_3$*).

^{13}C NMR (126 MHz, $CDCl_3$) δ 201.2 (C7), 141.2 (C3), 137.8 (C22), 135.4 (C11), 133.1 (C4), 132.8 (C18), 129.5 (ArC), 128.9 (ArC), 128.5 (C(17, 19)), 128.4 (C(16, 20)), 127.8 (ArC), 127.8 (ArC), 126.1 (ArC), 125.9 (ArC), 85.5 (C1), 65.9 (C21), 37.2 (C2), 27.0 (C6), 26.7 (C5), 19.2 (C9).

$[\alpha]_D^{25}$ = −72.0 (c 1.0, CH_2Cl_2).

HRMS (ESI): calculated for $C_{25}H_{24}O_2Na$ $[M+Na]^+$ requires m/z 379.1674, found m/z 379.1684.

Chiral SFC: DAICEL CHIRALCEL IE column (25 cm), CO_2:*i*-PrOH 95:5, 2.0 mL/min, 162 bar, 40 °C. Retention times: 13.3 mins (major), 17.2 mins (minor), e.r. = 94:6.

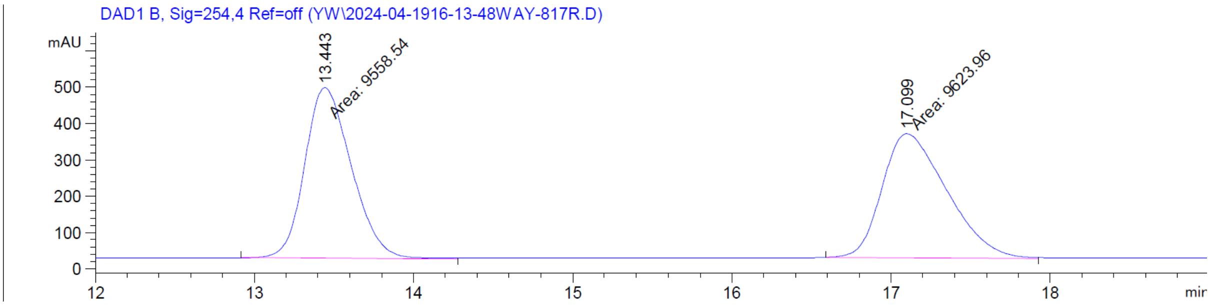

Signal 2: DAD1 B, Sig=254,4 Ref=off

Peak #	RetTime [min]	Type	Width [min]	Area [mAU*s]	Height [mAU]	Area %
1	13.443	MM	0.3392	9558.54492	469.72729	49.8295
2	17.099	MM	0.4694	9623.96289	341.68069	50.1705
Totals :				1.91825e4	811.40799	

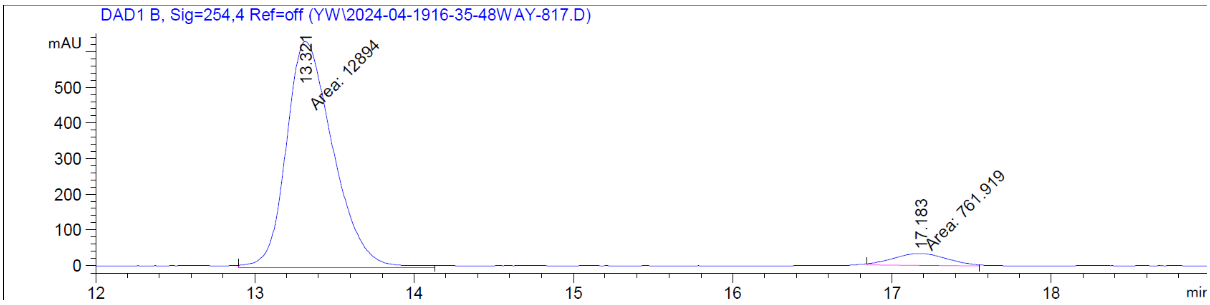

Signal 2: DAD1 B, Sig=254,4 Ref=off

Peak #	RetTime [min]	Type	Width [min]	Area [mAU*s]	Height [mAU]	Area %
1	13.321	MM	0.3391	1.28940e4	633.66016	94.4206
2	17.183	MM	0.3856	761.91901	32.93325	5.5794
Totals :				1.36559e4	666.59341	

(1*R*,2*S*)-2-(Benzyloxy)-1-methyl-2-(1-phenylvinyl)-1,2,3,4-tetrahydronaphthalene s34

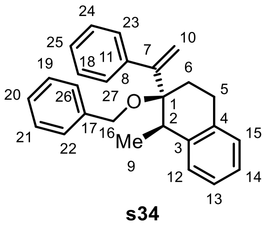

s34

Following a modified literature procedure,[27] a suspension of Ph_3PCH_3Br (64.3 mg, 0.18 mmol, 3.6 equiv.) in THF (0.3 mL) was cooled to −78 °C, and KHMDS in THF (1.0 M, 0.15 mL, 0.15 mmol, 3.0 equiv.) was added. The reaction was then stirred at r.t. for 30 minutes before being cooled back to −78 °C. A solution of **Bn-8m** (17.8 mg,

0.05 mmol) in THF (0.2 mL) was added. The reaction was warmed to r.t. and stirred for 1 h. The reaction was quenched with saturated aq. NH_4Cl and extracted with Et_2O. The organic phase was dried ($MgSO_4$) and concentrated *in vacuo* to give a crude product. The crude product was purified by FCC (100:0 to 20:1 hexane/Et_2O) to give **s34** (15 mg, 85%, >20:1 d.r., 94:6 e.r.) as a colorless oil.

IR (film) ν_{max}/cm^{-1}: 2966, 2870, 1491, 1061, 915, 696.

^{1}H NMR (500 MHz, $CDCl_3$) δ 7.50 – 7.47 (m, 4H, Ar*H*), 7.41 (t, *J* = 7.4 Hz, 2H, C(18, 24)*H*), 7.34 (t, *J* = 7.3 Hz, 1H, C25*H*), 7.29 – 7.28 (m, 3H, Ar*H*), 7.16 – 7.09 (m, 3H, Ar*H*), 7.03 (d, *J* = 7.0 Hz, 1H, C15*H*), 5.45 (s, 1H, C10H_A), 5.35 (s, 1H, C10H_B), 4.62 (q, *J* = 11.4 Hz, 2H, C16H_2), 3.63 (q, *J* = 7.0 Hz, 1H, C2*H*), 2.73 (dd, *J* = 17.4 Hz, 6.1 Hz, 1H, C5H_A), 2.63 – 2.55 (m, 1H, C5H_B), 2.22 – 2.15 (m, 1H, C6H_A), 2.04 (dd, *J* = 13.1 Hz, 6.3 Hz, 1H, C5H_B), 1.41 (d, *J* = 7.0 Hz, 3H, C9H_3).

^{13}C NMR (126 MHz, $CDCl_3$) δ 146.9 (C7), 141.7 (C3), 141.1 (C8), 138.8 (C17), 134.6 (C4), 128.8 (ArC), 128.6 (ArC), 128.5 (ArC), 128.5 (ArC), 127.9 (ArC), 127.7 (ArC), 127.4 (ArC), 127.3 (ArC), 125.8 (ArC), 125.8 (ArC), 120.1 (C10), 80.0 (C1), 65.6 (C16), 38.4 (C2), 26.7 (C6), 26.5 (C5), 19.9 (C9).

$[\alpha]_D^{25}$ = –60.7 (c 0.5, CH_2Cl_2).

Chiral SFC: YMC Chiral ART Cellulose-SC column (25 cm), CO_2:*i*-PrOH 98:2, 2.0 mL/min, 156 bar, 40 °C. Retention times: 9.0 mins (major), 9.6 mins (minor), e.r. = 94:6.

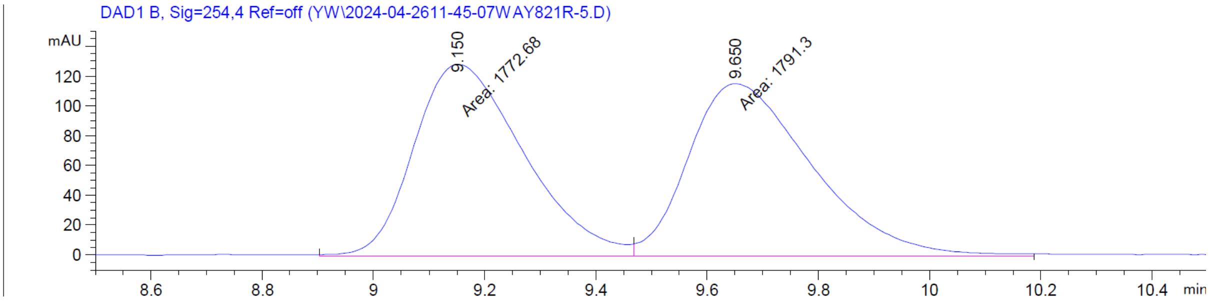

Signal 2: DAD1 B, Sig=254,4 Ref=off

Peak #	RetTime [min]	Type	Width [min]	Area [mAU*s]	Height [mAU]	Area %
1	9.150	MF	0.2294	1772.68005	128.77779	49.7388
2	9.650	FM	0.2581	1791.29504	115.66270	50.2612
Totals :				3563.97510	244.44049	

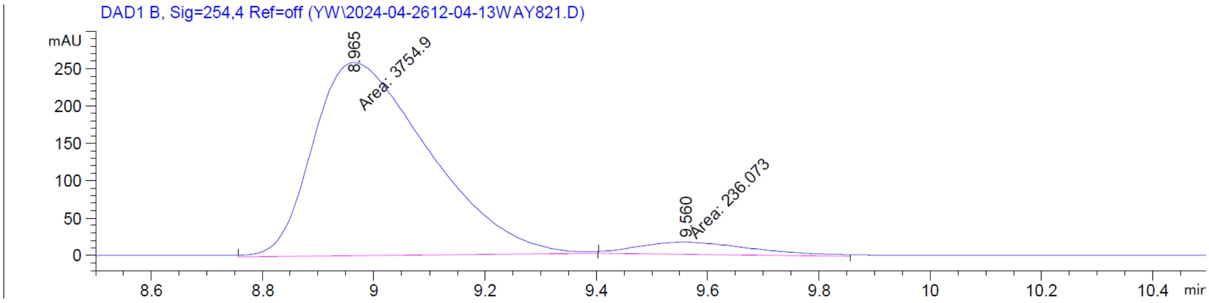

Signal 2: DAD1 B, Sig=254,4 Ref=off

Peak #	RetTime [min]	Type	Width [min]	Area [mAU*s]	Height [mAU]	Area %
1	8.965	MM	0.2417	3754.89819	258.97058	94.0848
2	9.560	MM	0.2428	236.07294	16.20604	5.9152

Totals : 3990.97113 275.17662

Mechanistic studies

Control experiments

Importance of the hydroxyl and carbonyl groups

The presence of both the ketone and α-hydroxy groups (Scheme s1a & b) was found to be important for effective catalysis, and protecting the alcohol was found to halt catalysis (Scheme 1c). Interestingly, switching the position of these groups is possible (Scheme 1d), supporting the proposed intermediacy of an enediolate (*manuscript* **Int-I**). Circumstantial support for the mechanism in Scheme 3A is provided by Scheme 1e and 1f. The former shows that non-oxidizable O-directing groups can be used, albeit with competitive *ortho*-alkylation of the phenyl ketone. The latter shows that 1,2-diketones are not viable substrates in the presence of an exogenous alcohol reductant (i-PrOH), which disfavors the pathway in Scheme 3B.

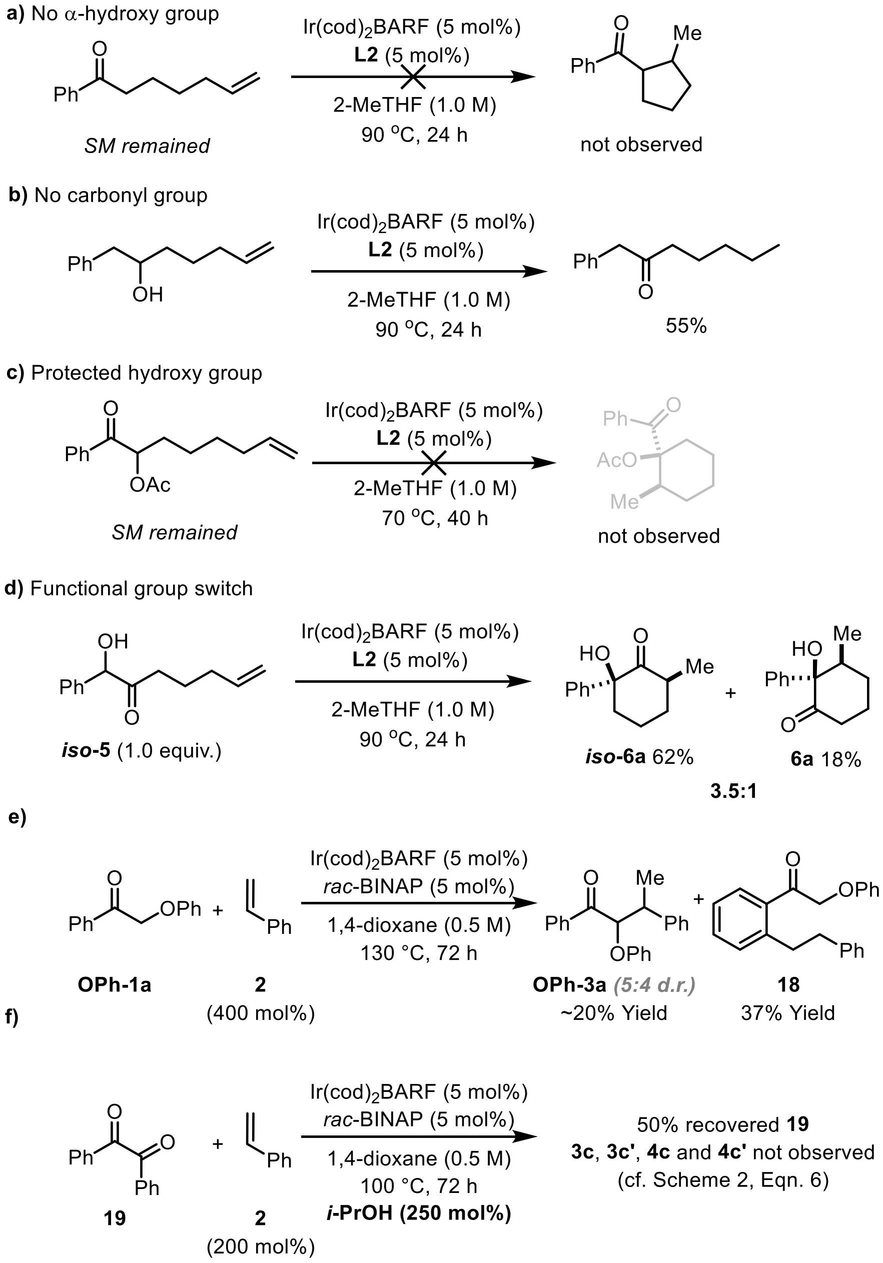

Scheme s1 Experiments demonstrating the necessity of the hydroxyl and carbonyl groups.

Procedure and data for Scheme 1e:

OPh-3a *and* 1-(2-phenethylphenyl)-2-phenoxyethan-1-one 18

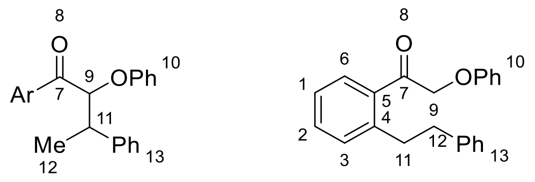

OPh-3a **18**

Conducted according to *General Procedure H.* **OPh-1a** (42.5 mg, 0.20 mmol, 100 mol%), [Ir(cod)$_2$]BARF (12.7 mg, 0.01 mmol, 5 mol%), *rac*-BINAP (6.2 mg, 0.01 mmol, 5 mol%), styrene (82.4 mg, 0.80 mmol, 400 mol%) and 1,4-dioxane (0.40 mL, 0.5 M) were used. The tube was sealed and heated at 130 °C for 72 h. The crude was analyzed by ^{1}H NMR spectroscopy and purified by FCC (20:1 to 10:1 hexane/EtOAc).

The first product to elute was **OPh-3a** (22 mg, ~5:4 d.r.), which was isolated as a colorless oil. This was contaminated with unidentified impurities/signals, which could not be separated. We estimated a yield for **OPh-3a** of approximately 20% and 2D NMR experiments were used to confirm the core structure. We tentatively suggest that Ar = Ph for **OPh-3a** based on the HRMS data, but we were unable to discount that *o*-alkylation of this unit may have also occurred (cf. **18**).

IR (film) v_{max}/cm^{-1}: 3061 (s), 1695 (s), 1598 (s), 1493 (s), 1222 (s), 1028 (s), 752 (s).

^{1}H NMR (500 MHz, CDCl$_3$) δ 7.81 (0.43H, d, *J* = 7.7 Hz, Ph*H*), 7.66 (0.57H, d, *J* = 7.7 Hz, Ph*H*), 7.45 – 7.38 (1.7H, m, Ph*H*), 7.33 – 7.17 (16H, m, Ph*H*), 6.97 – 6.78 (3.7H, m, Ph*H*), 5.47 (0.44H, d, *J* = 4.9 Hz, minor C9*H*), 5.41 (0.58H, d, *J* = 5.8 Hz, major C9*H*), 3.62 – 3.55 (0.7H, m, major C11*H*), 3.45 – 3.41 (0.44H, m, minor C11*H*), 1.51 (1.05H, d, *J* = 7.1 Hz, minor C12*H$_3$*), 1.47 (1.36H, d, *J* = 7.2 Hz, major C12*H$_3$*).

^{13}C NMR (126 MHz, CDCl$_3$) δ 201.4 (major, C7), 200.8 (minor, C7), 158.4 (major, CPh), 158.2 (minor, CPh), CPh: 143.3, 143.2, 142.6, 142.1, 142.0, 141.8, 135.6, 135.6, 133.5, 131.8, 131.7, 131.6, 129.5, 129.5, 128.6, 128.5, 128.4, 128.4, 128.3, 128.2, 127.9, 127.0, 125.9, 125.9, 125.8, 125.7, 86.7 (major, C9), 85.9 (minor, C9), 43.3 (major, C11), 42.6 (minor, C11), 18.8 (major, C12), 16.2 (minor, C12).

HRMS (ESI): calculated for $C_{22}H_{20}NaO_2$ [M+Na]$^+$ requires *m/z* 339.1356, found *m/z* 339.1353.

Continued elution provided *ortho*-alkylation product **18** (23.2 mg, 37% yield) as a colorless oil

IR (film) v_{max}/cm^{-1}: 3027 (s), 1703 (s), 1560 (s), 1495 (s), 1213 (s), 966 (s), 753 (s);

^{1}H NMR (500 MHz, CDCl$_3$) δ 7.69 (1H, d, *J* = 7.5 Hz, C6*H*), 7.47 (1H, t, *J* = 7.4 Hz, Ph*H*), 7.35 – 7.26 (8H, m, Ph*H*), 7.21 – 7.15 (4H, m, Ph*H*), 7.01 (1H, t, *J* = 7.4 Hz, Ph*H*), 6.95 (2H, d, *J* = 8.0 Hz, Ph*H*), 5.05 (2H, s, C9*H$_2$*), 3.17 (2H, t, *J* = 8.2 Hz, C11*H$_2$*), 2.92 (2H, t, *J* = 7.7 Hz, C12*H$_2$*).

^{13}C NMR (126 MHz, $CDCl_3$) δ 198.5 (C7), 157. 9 (CPh), 142.6 (CPh), 141.6 (CPh), 132.1 (CPh), 131.8 (CPh), 129.6 (CPh), 128.7 (CPh), 128.4 (CPh), 128.3 (CPh), 126.0 (CPh), 125.9 (CPh), 121.7 (CPh), 114.8 (CPh), 71.8 (C9), 38.2 (C12), 36.0 (C11).

HRMS (ESI): calculated for $C_{22}H_{20}NaO_2$ $[M+Na]^+$ requires *m/z* 339.1356, found *m/z* 339.1358.

Rearrangement of 6a to *iso*-6a

A mixture of **6a**/***iso*-6a** was resubjected to the reaction conditions (Scheme s2): (a) in the presence of D_2O, wherein no C-H deuterium incorporation was observed in the starting material or rearranged product; (b) when [Ir] was omitted from the reaction, the ratio of products did not change, implying that [Ir] plays a critical role in promoting the rearrangement. These reactions were carried out according to *General Procedure I.*

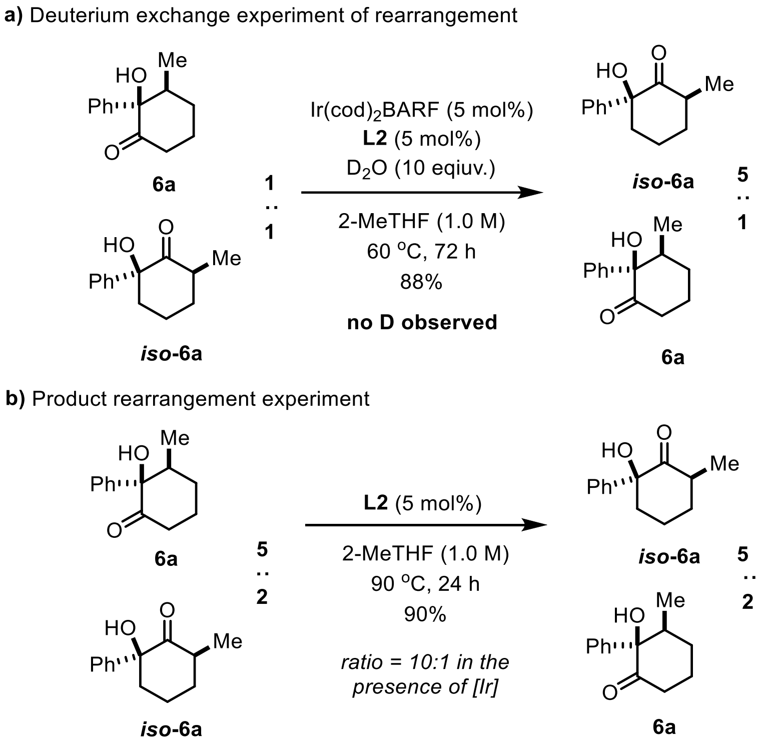

Scheme s2 Experiments highlighting the necessity of [Ir] for product rearrangement.

C-Methylation of 5a with sodium hydride and methyl iodide

When α-hydroxy ketone **5a** was subjected to base-mediated methylation conditions using 1.1 equivalents of NaH and 1.2 equivalents of MeI, only a mixture of C-methylated products (**s33** & **s34**) was observed in 49% overall yield. This implies that the initially generated sodium alkoxide acidifies the α-H to such an extent that it deprotonates in preference to another -OH unit. This then generates a *bis*-sodium enediolate which alkylates with methyl iodide at either carbon center. These observations provide circumstantial support for the soft enolization mechanism in the main paper, and show that **5a**-derived enediolates will alkylate through either carbon center.

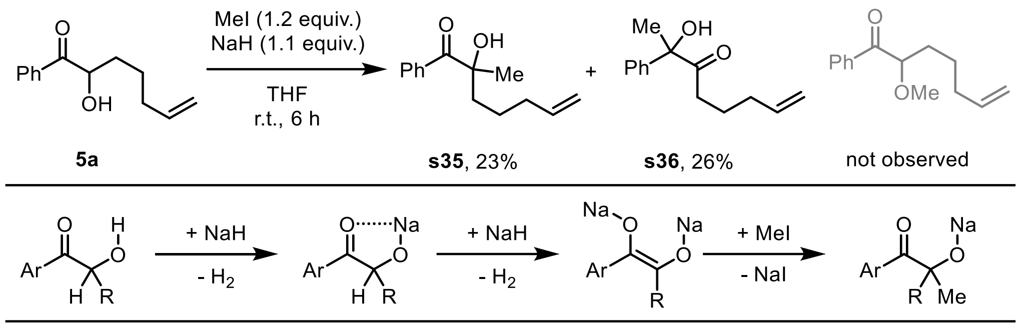

Procedure: Following a modified literature procedure,[28] sodium hydride (52 mg, 60% dispersion in mineral oil, 1.3 mmol, 1.1 equiv.) was added to a solution of **5a** (245 mg, 1.2 mmol) in anhydrous THF (10 mL) under nitrogen atmosphere. To the resulting solution, methyl iodide (87 µL, 1.4 mmol, 1.2 equiv.) was added and the mixture was stirred for 6 h. The final yellow suspension was concentrated *in vacuo*. H_2O (1 mL) was added to quench excess sodium hydride and the mixture was extracted with chloroform (3 × 15 mL). The organic extracts were dried ($MgSO_4$) and concentrated *in vacuo* to yield a colorless oil. This was purified by FCC (20:1 to 5:1 hexane/Et_2O) to give **s33** (60 mg, 23%) and **s34** (67 mg, 26%) as colorless oils. Data are given below.

2-Hydroxy-2-methyl-1-phenylhept-6-en-1-one s35

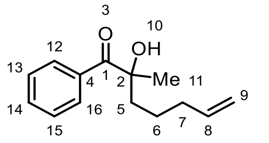

s35

IR (film) ν_{max}/cm^{-1}: 3457, 2934, 1706, 1447, 1068, 912.

^{1}H NMR (500 MHz, $CDCl_3$) δ 8.02 – 8.00 (m, 2H, C(12, 16)*H*), 7.61 (t, *J* = 7.4 Hz, 1H, C14*H*), 7.50 (t, *J* = 7.9 Hz, 2H, C(13, 15)*H*), 5.75 – 5.67 (m, 1H, C8*H*), 4.98 – 4.92 (m, 2H, C9H_2), 4.30 (s, 1H, O*H*), 2.10 – 1.92 (m, 4H, C5H_2, C7H_2), 1.63 (s, 3H, C11H_3), 1.62 – 1.60 (m, 1H, C6H_A), 1.22 – 1.15 (m, 1H, C6H_B).

^{13}C NMR (126 MHz, $CDCl_3$) δ 204.8 (C1), 138.2 (C8), 133.9 (C4), 133.1 (C14), 129.4 (C(12, 16)), 128.6 (C(13, 15)), 114.9 (C9), 78.8 (C2), 40.7 (C5), 33.7 (C7), 27.5 (C11), 22.7 (C6).

HRMS (ESI): calculated for $C_{14}H_{18}O_2Na$ $[M+Na]^+$ requires m/z 241.1204, found m/z 241.1198.

2-Hydroxy-2-phenyloct-7-en-3-one s36

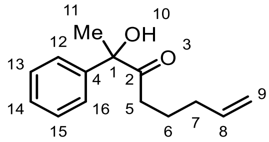

s36

IR (film) ν_{max}/cm^{-1}: 3464, 2930, 1668, 1447, 1163, 911.

^{1}H NMR (500 MHz, $CDCl_3$) δ 7.45 – 7.43 (m, 2H, C(12, 16)*H*), 7.41 – 7.38 (m, 2H, C(13, 15)*H*), 7.34 – 7.31 (m, 1H, C14*H*), 5.69 – 5.60 (m, 1H, C8*H*), 4.93 – 4.87 (m, 2H, C9H_2), 4.61 (s, 1H, O*H*), 2.50 – 2.44 (m, 1H, C5H_A), 2.38 – 2.32 (m, 1H, C5H_B), 1.97 – 1.85 (m, 2H, C7H_2), 1.79 (s, 3H, C11H_3), 1.68 – 1.51 (m, 2H, C6H_2).

^{13}C NMR (126 MHz, $CDCl_3$) δ 211.8 (C2), 141.5 (C4), 137.6 (C8), 128.7 (C(13, 15)), 128.0 (C14), 126.1 (C(12, 16)), 115.3 (C9), 79.8 (C2), 34.6 (C5), 32.7 (C7), 27.1 (C11), 23.0 (C6).

HRMS (ESI): calculated for $C_{14}H_{18}O_2Na$ $[M+Na]^+$ requires m/z 241.1204, found m/z 241.1198.

Deuterium labelling and exchange experiments

Preparation of deuterated starting material 5a-D

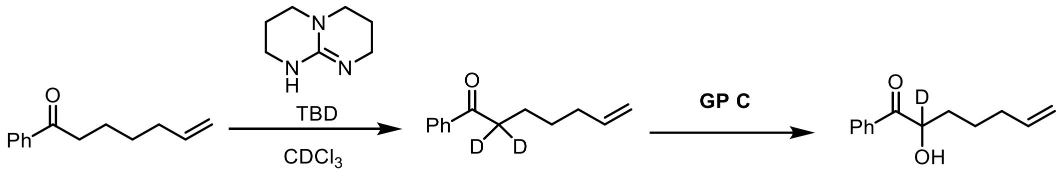

5a-s2 **5a-s2-D**, 89%, 1.87 D **5a-D**, 42%, 0.87 D

Preparation of **5a-D**: Following literature procedures,[29] to a solution of of TBD (40 mg, 0.29 mmol, 10 mol %) in $CDCl_3$ (10 mL), was added ketone **5a-s2** (546 mg, 2.90 mmol). The reaction mixture was stirred at r.t. for 16 h and quenched with 1 M aq. HCl (1 mL). The organic layer was washed with H_2O (5 × 2 mL), brine (5 mL), dried (Na_2SO_4) and concentrated *in vacuo* to afford **5a-s2-D** (480 mg, 89%, 1.87 D), which was used directly in *General Procedure D* to afford **5a-D** (170 mg, 42%, 0.87 D).

Procedure of deuterium labelling and exchange experiments detailed below: A Schlenk tube was charged with α-hydroxyketone **5a** or **5a-D** (0.10 mmol, 100 mol%), $[Ir(cod)_2]BARF$ (0.005 mmol, 5 mol%) and (*R*)-SEGPHOS (0.005 mmol, 5 mol%). The Schlenk tube was evacuated/backfilled with N_2 (three cycles), then D_2O (1000 mol%) (if using **5a** as starting material) was added, followed by 2-MeTHF (0.10 mL, 1.0 M). The tube was sealed and heated at the specified time and temperature (see Scheme s3). After cooling to r.t., the mixture was concentrated *in vacuo* and residue was purified by FCC (100% hexane to 4:1 hexane/Et_2O). The obtained products were analyzed by ^{1}H NMR and ^{2}H NMR spectroscopy using either $CDCl_3$ or $CHCl_3$ as solvent.

Summary of experiments

When the reaction was performed using deuterated starting material **5a-D**, deuterium was transferred to H3 and H1 of ***iso*-6a** (Scheme s3a). Transfer to the methyl group supports the proposed mechanism, wherein inner sphere addition of an [Ir]-D/H enediolate across the alkene occurs prior to C-D/H reductive elimination. The exchange at H1 possibly occurs via enolization of **6a** (prior to rearrangement to ***iso*-6a**). In support of this, a D_2O exchange experiment using **5a** (Scheme s3b) was run at 60 °C, leading to a 2.4:1 ratio of **6a**:***iso*-6a**. The former had 0.41D incorporation at H1.

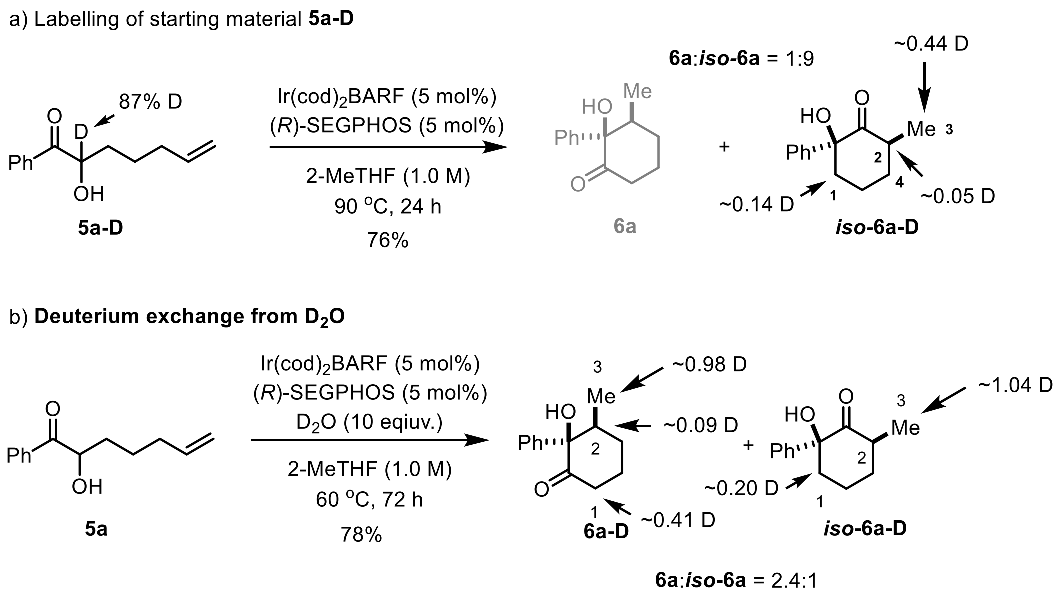

Scheme s3 Deuterium labelling and exchange experiments

Representative NMR spectra

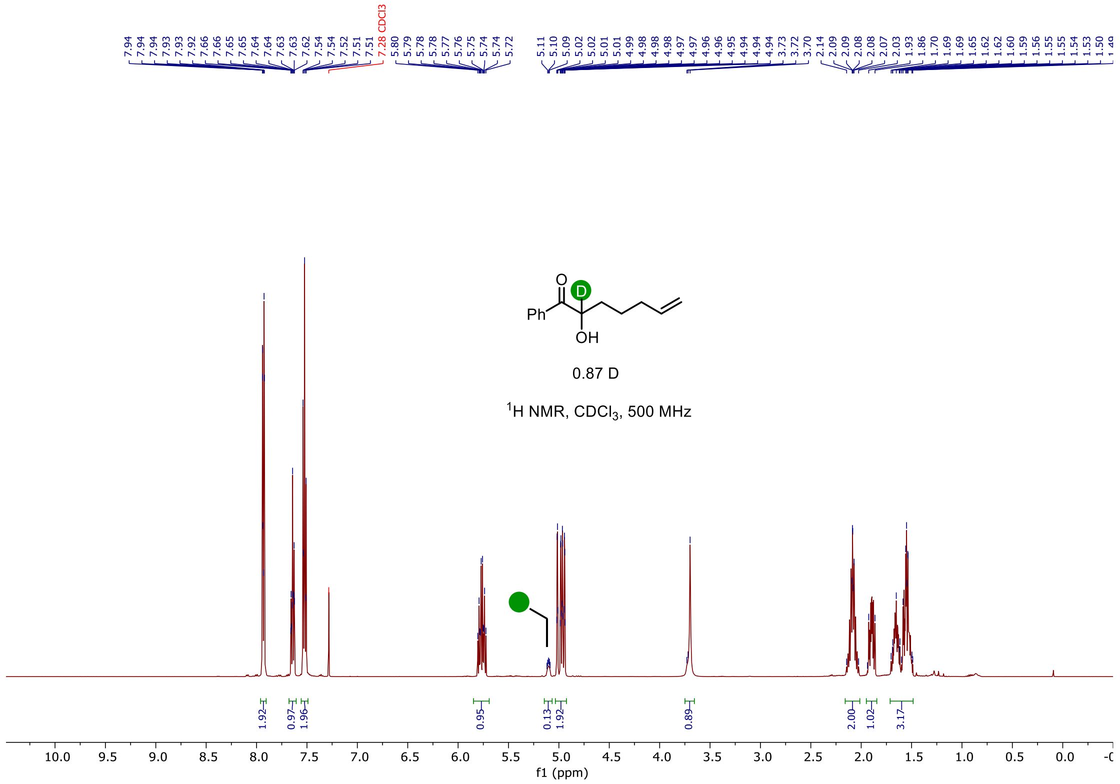

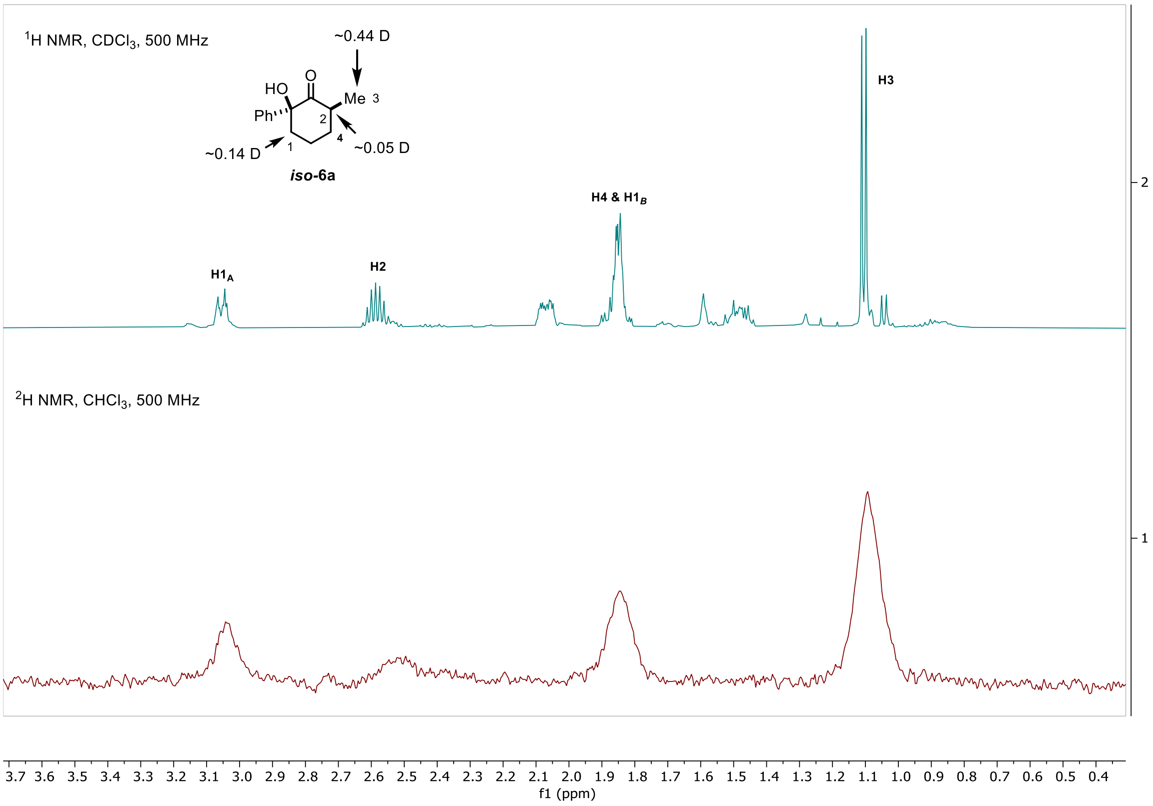

^{1}H NMR, CDCl$_3$, 500 MHz
~0.44 D
HO
O
Me 3
Ph
2
1
4
~0.14 D
~0.05 D
iso-6a
H3
H4 & H1$_B$
H1$_A$
H2
^{2}H NMR, CHCl$_3$, 500 MHz
3.7 3.6 3.5 3.4 3.3 3.2 3.1 3.0 2.9 2.8 2.7 2.6 2.5 2.4 2.3 2.2 2.1 2.0 1.9 1.8 1.7 1.6 1.5 1.4 1.3 1.2 1.1 1.0 0.9 0.8 0.7 0.6 0.5 0.4
f1 (ppm)

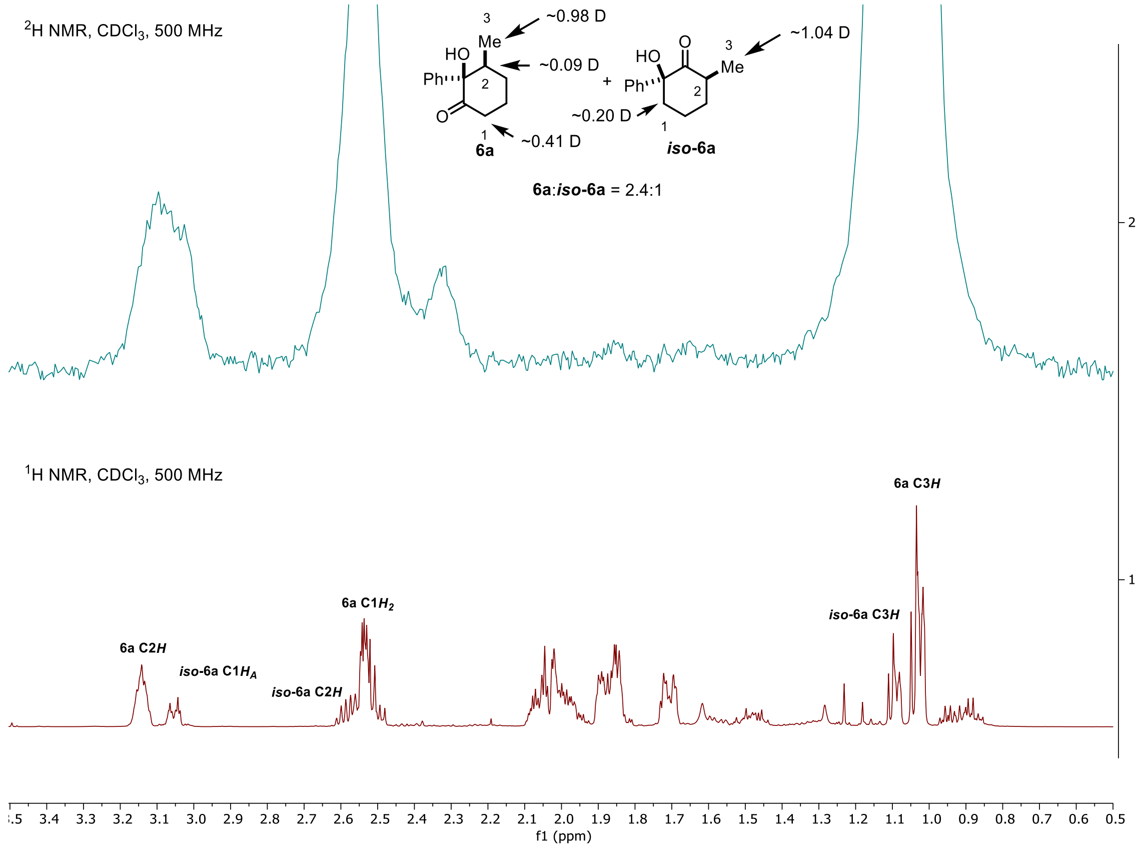

^{2}H NMR, CDCl$_3$, 500 MHz
~0.98 D
~0.09 D
~0.41 D
6a
~1.04 D
~0.20 D
iso-6a
6a:iso-6a = 2.4:1
^{1}H NMR, CDCl$_3$, 500 MHz
6a C3H
iso-6a C3H
6a C1H$_2$
6a C2H
iso-6a C1H$_A$
iso-6a C2H
3.4 3.3 3.2 3.1 3.0 2.9 2.8 2.7 2.6 2.5 2.4 2.3 2.2 2.1 2.0 1.9 1.8 1.7 1.6 1.5 1.4 1.3 1.2 1.1 1.0 0.9 0.8 0.7 0.6 0.5
f1 (ppm)

References

[1] Guven, S.; Kundu, G.; Wessels, A.; Ward, J. S.; Rissanen, K.; Schoenebeck, F. *J. Am. Chem. Soc* **2021**, *143*, 8375–8380.
[2] Davies, J.; Booth, S. G.; Essafi, S.; Dryfe, R. A. W.; Leonori, D. *Angew. Chem. Int. Ed.* **2015**, *54*, 14017–14021.
[3] Yin, H.; Fu, G. C. *J. Am. Chem. Soc.* **2019**, *141*, 15433–15440.
[4] Liu, Y.; Brown, M. K. *J. Am. Chem. Soc.* **2023**, *145*, 25061–25067.
[5] Liang, Y.-F.; Wu, K.; Song, S.; Li, X.; Huang, X.; Jiao, N. *Org. Lett.* **2015**, *17*, 876–879.
[6] Chanthamath, S.; Takaki, S.; Shibatomi, K.; Iwasa, S. *Angew. Chem., Int. Ed.* **2013**, *52*, 5818–5821.
[7] Barrios Antunez, D.-J.; Greenhalgh, M. D.; Fallan, C.; Slawin, A. M. Z.; Smith, A. D. *Org. Biomol. Chem.* **2016**, *14*, 7268–7274.
[8] Chen, C.-T.; Kao, J.-Q.; Salunke, S. B.; Lin, Y.-H. *Org. Lett.* **2011**, *13*, 26–29.
[9] Nakamura, T.; Shirokawa, S.; Hosokawa, S.; Nakazaki, A.; Kobayashi, S. *Org. Lett.* **2006**, *8*, 677–679.
[10] Shimosato, J.; Sawamura, M.; Masuda, Y. *Org. Lett.* **2024**, *26*, 2023–2028.
[11] a) Hünig, S.; Marschner, C. *Chem. Ber.* **1989**, *122*, 1329–1339; b) Chen, T.; Liu, W.; Gu, W.; Niu, S.; Lan, S.; Zhao, Z.; Gong, F.; Liu, J.; Yang, S.; Cotman, A. E.; Song, J.; Fang, X. *J. Am. Chem. Soc*. **2023**, *145*, 585–599.
[12] Sedrani, R.; Kallen, J.; Cabrejas, L. M. M.; Papageorgiou, C. D.; Senia, F.; Rohrbach, S.; Wagner, D.; Thai, B.; Eme, A. M. J.; France, J.; Oberer, L.; Rihs, G.; Zenke, G.; Wagner, J. *J. Am. Chem. Soc*. **2003**, *125*, 3849–3859.
[13] Wessig, P.; Mühling, O. *Helv. Chim. Acta* **2003**, *86*, 865–893.
[14] Xing, S.; Gu, N.; Wang, X.; Liu, J.; Xing, C.; Wang, K.; Zhu, B. *Org. Lett.* **2018**, *20*, 5680–5683.
[15] Genrich, F.; Schaumann, E. *Tetrahedron Lett.* **2009**, *50*, 6187-6190.
[16] Zheng, J.; Meng, S.; Wang, Q. *Beilstein. J. Org. Chem*. **2021**, *17*, 1481–1489.
[17] Lin, S.; Song, C.-X.; Cai, G.-X.; Wang, W.-H.; Shi, Z.-J. *J. Am. Chem. Soc.* **2008**, *130*, 12901–12903.
[18] Tang, M.; Kong, Y.-F.; Chu, B.-J.; Feng, D. *Adv. Synth. Catal.* **2016**, *358*, 926–939.
[19] Tarantino, K. T.; Liu, P.; Knowles, R. R. *J. Am. Chem. Soc.* **2013**, *135*, 10022–10025.
[20] Yabe, R.; Ebe, Y.; Nishimura, T. *Synthesis* **2021**, *53*, 3051–3056.
[21] Plietker, B. *Eur. J. Org. Chem.* **2005**, 1919–1929.
[22] Barrow, R.A.; Moore, R. E.; Li, L.; Tius, M. A. *Tetrahedron* **2000**, *56*, 3339–3351.
[23] Chciuk, T. V.; Anderson Jr., W. R.; Flowers II, R. A. *Organometallics* **2017**, *36*, 4579–4583.
[24] Shindo, M.; Yoshimura, Y.; Hayashi, M.; Soejima, H.; Yoshikawa, T.; Matsumoto, K.; Shishido, K. *Org. Lett.* **2007**, *9,* 1963–1966.
[25] Oguma, T.; Katsuki, T. *J. Am. Chem. Soc.* **2012**, *134*, 20017–20020.
[26] Barnard, K. R.; Shiers, D. W.; Kelly, N. J.; Lombardo, D. *Solvent Extr. Ion Exch.* **2015**, *33*, 166–182.
[27] Peng, R.; Van Nieuwenhze, M. S. *J. Org. Chem.* **2019**, *84*, 760–768.
[28] Bhattacharya, S.; Rahaman, R.; Chatterjee, S.; Paine, T. K. *Chem. Eur. J.* **2017**, *23*, 3815–3818.
[29] Sabot, C.; Kumar, K. A.; Antheaume, C.; Mioskowski, C. *J. Org. Chem.* **2007**, *72*, 5001–5004.

NMR spectra

Intermolecular hydroalkylation products

(*syn*)-2-Hydroxy-1,3-diphenylbutan-1-one 3a and (*anti*)-1-Hydroxy-1,3-diphenylbutan-2-one *iso*-3a

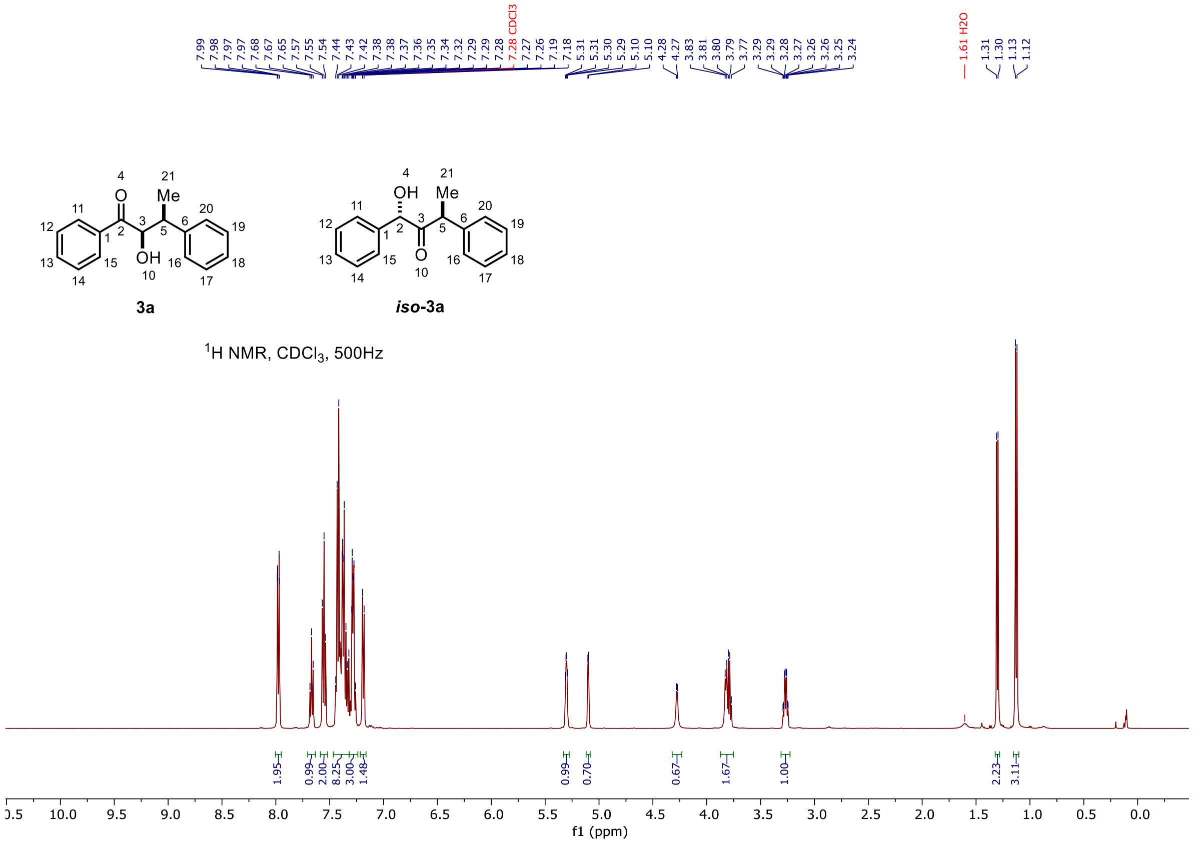

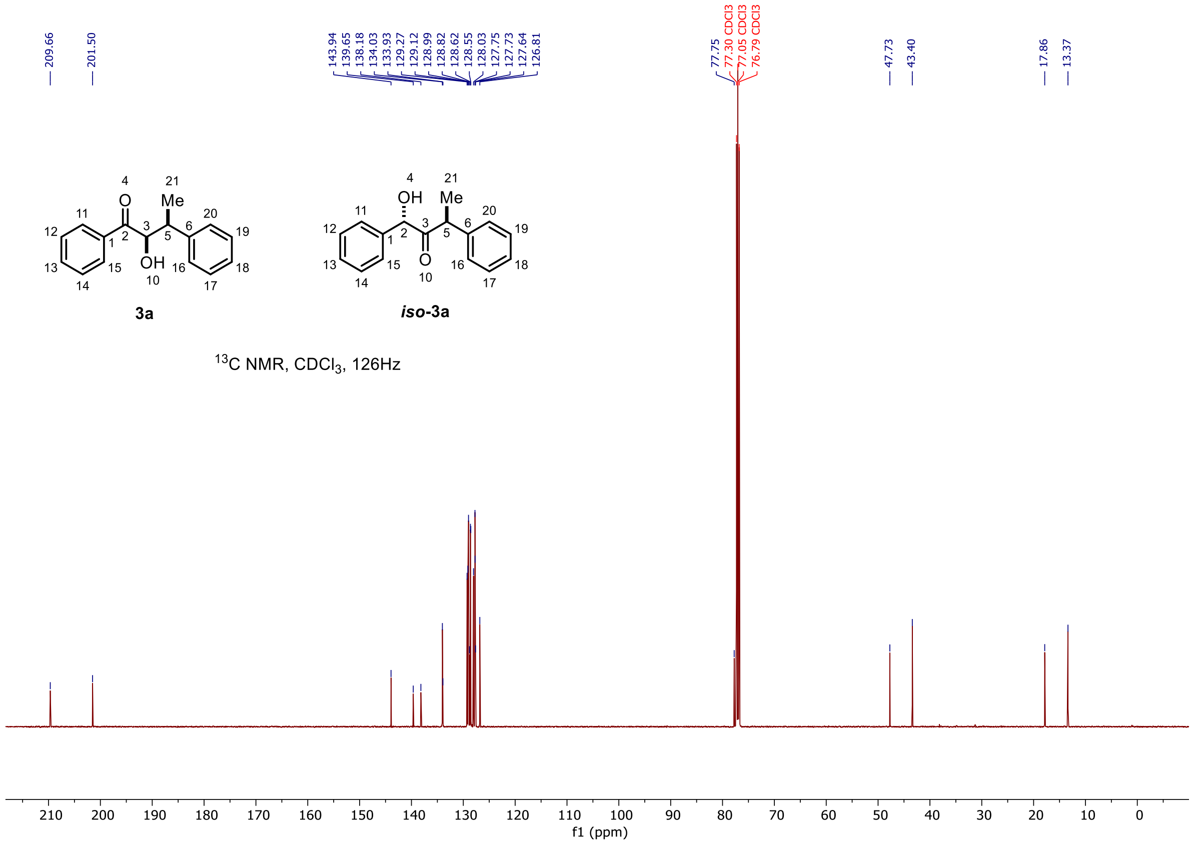

(*anti*)-2-Hydroxy-1,3-diphenylbutan-1-one 3a (minor)

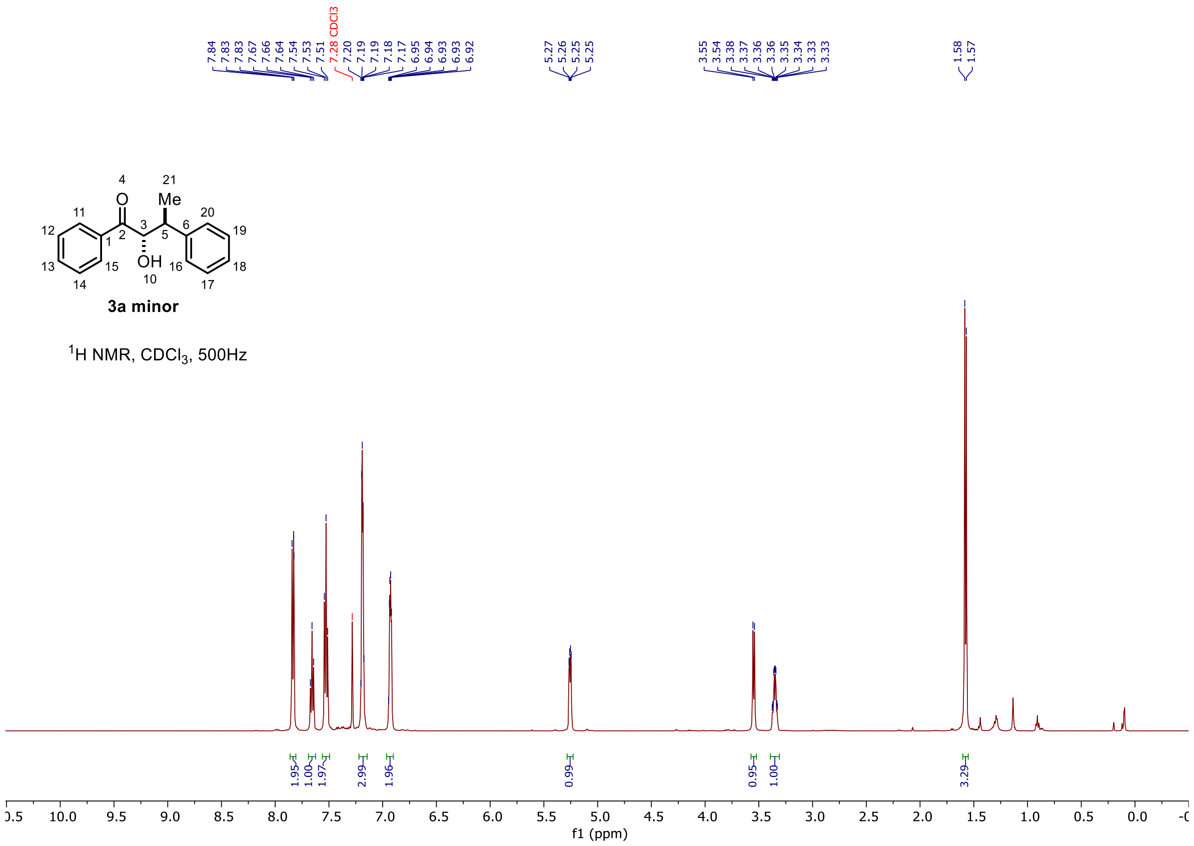

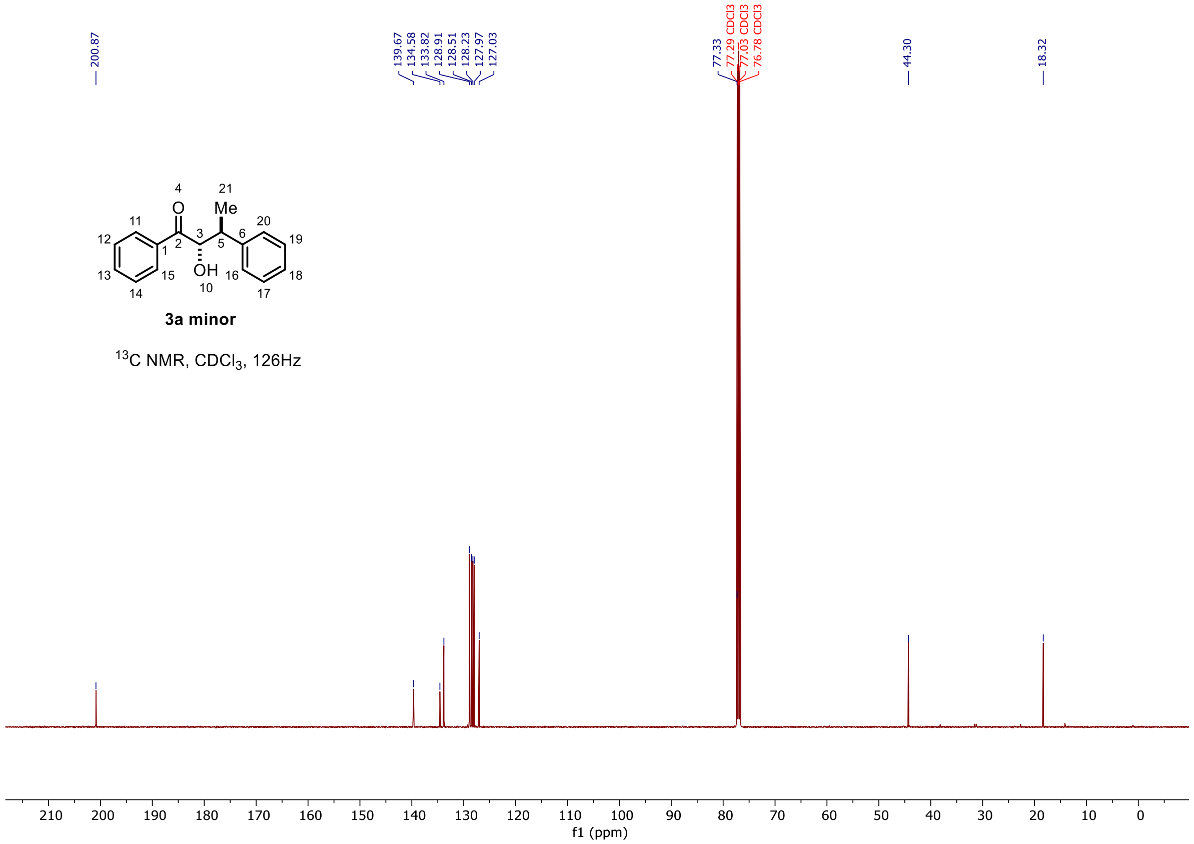

2-Hydroxy-2-methyl-1,3-diphenylbutan-1-one 3b and 3-Hydroxy-3,4-diphenylpentan-2-one *iso*-3b

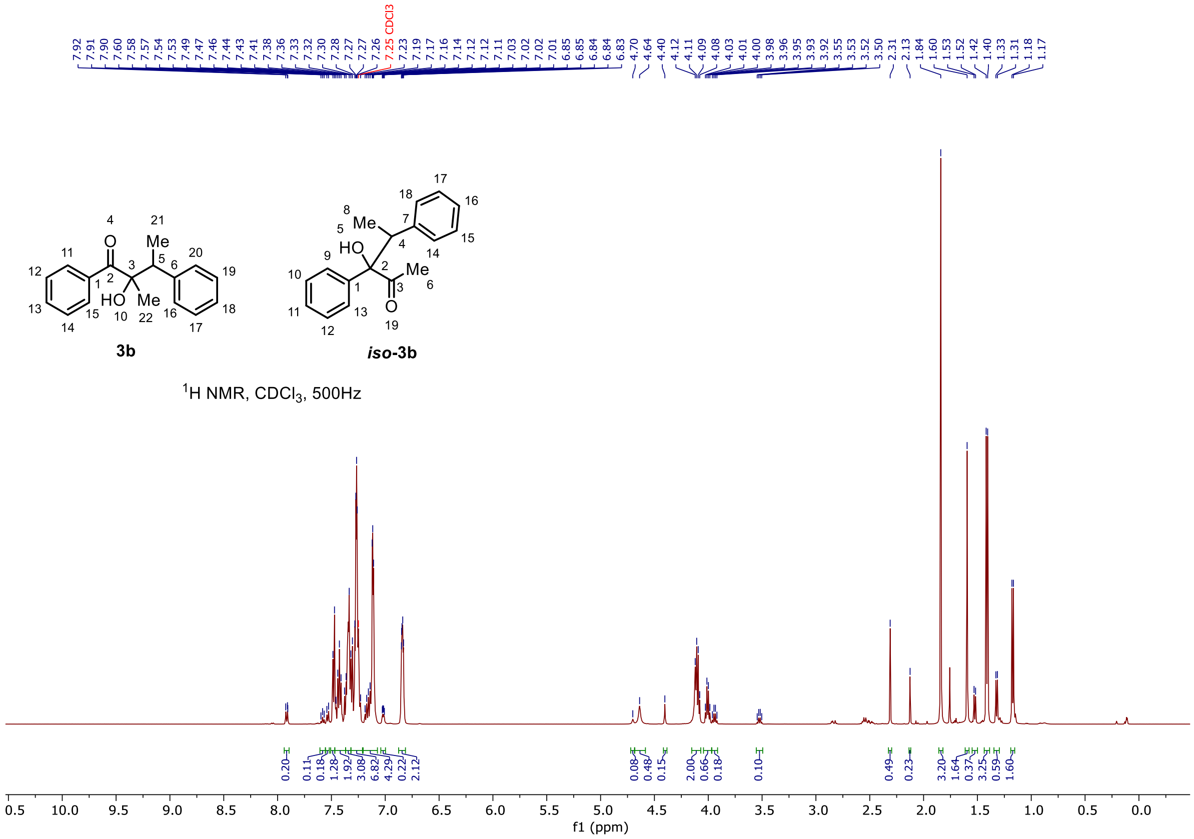

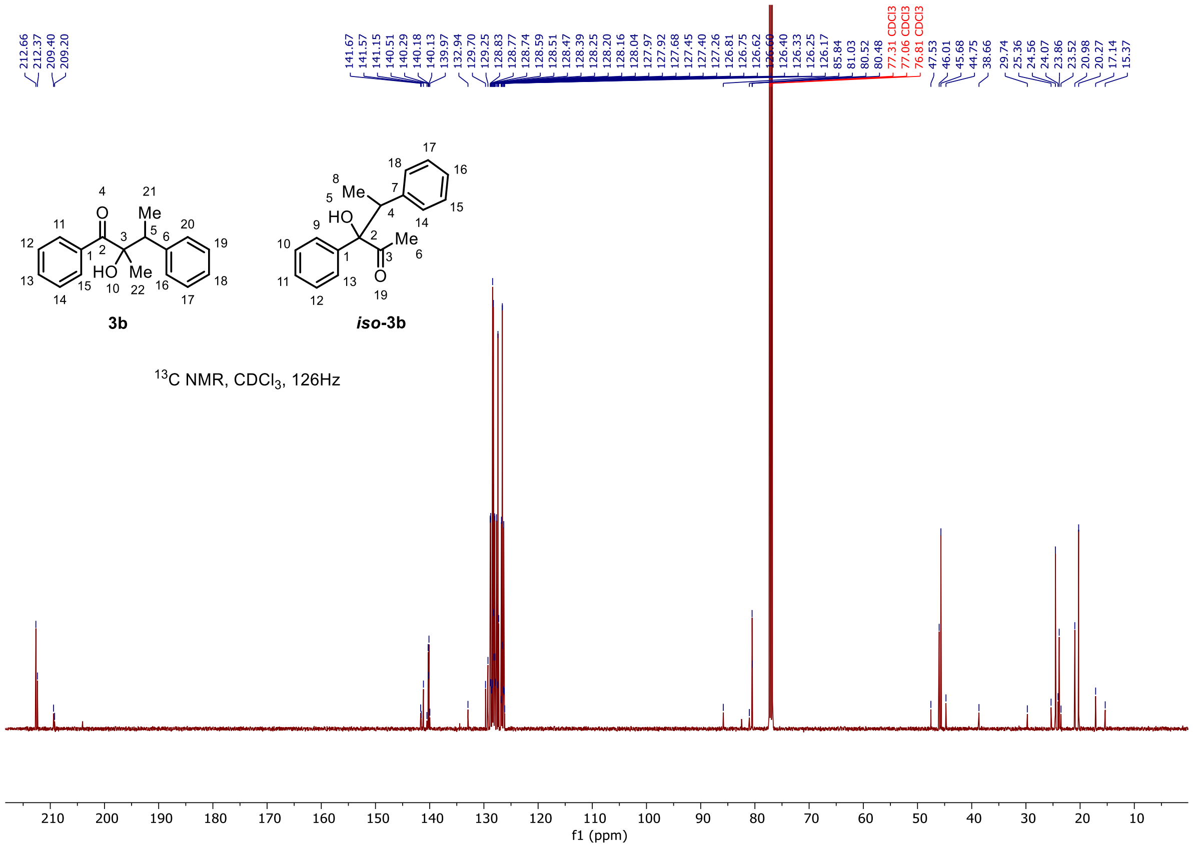

4-Hydroxy-1,4-diphenylpentan-3-one 4b’

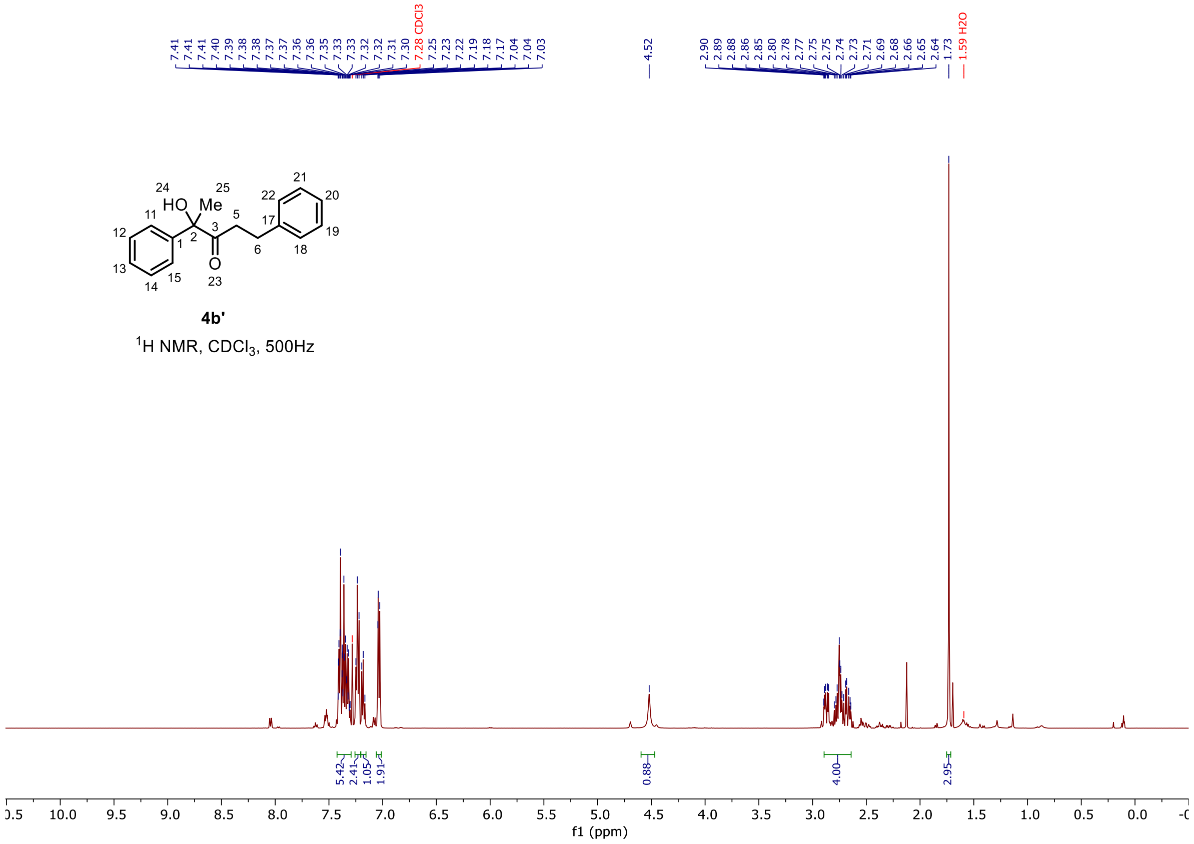

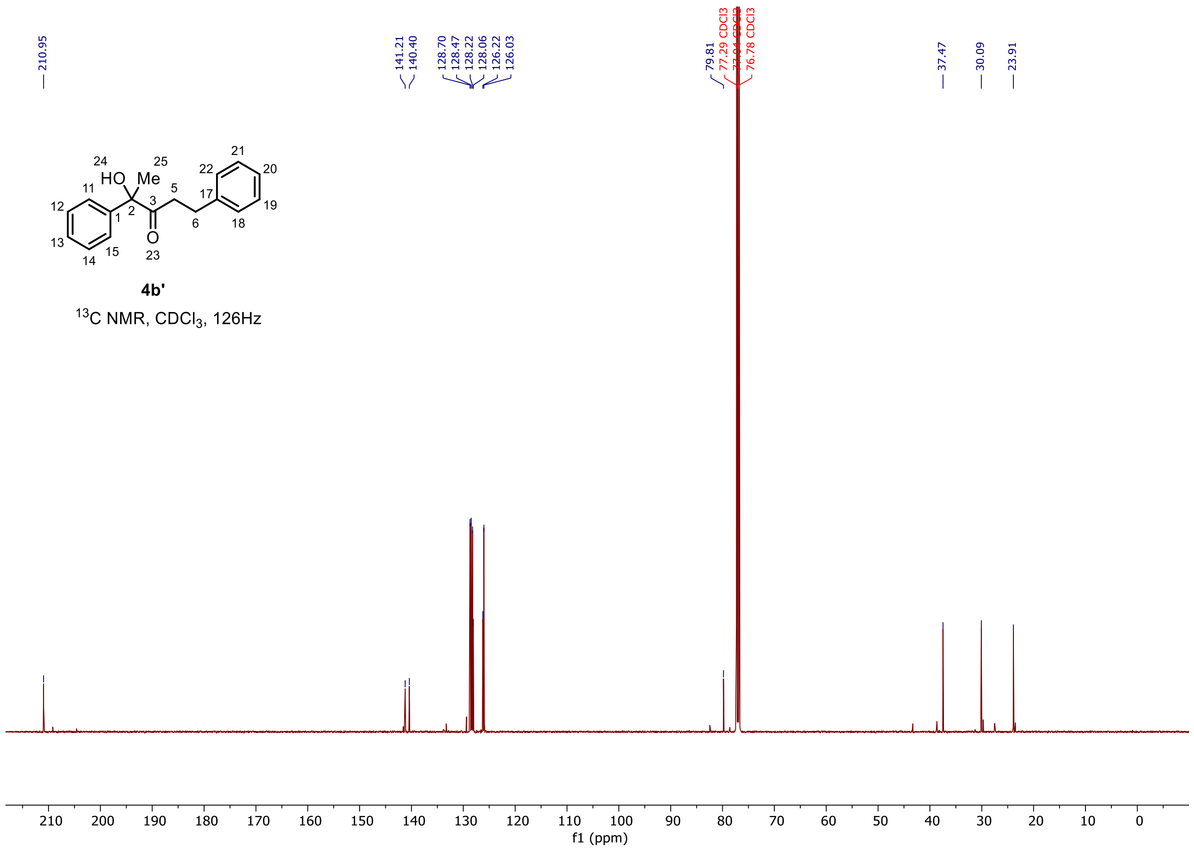

2-Hydroxy-1,2,4-triphenylbutan-1-one 4c

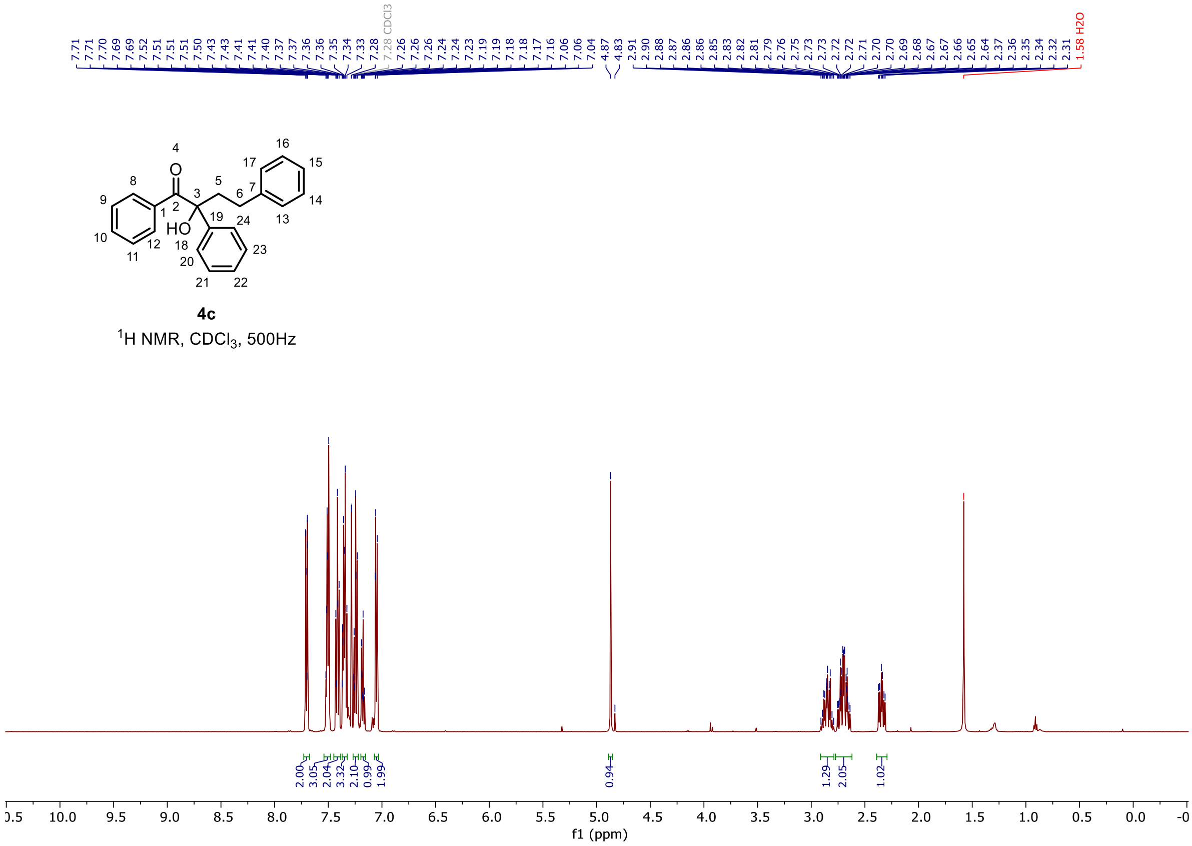

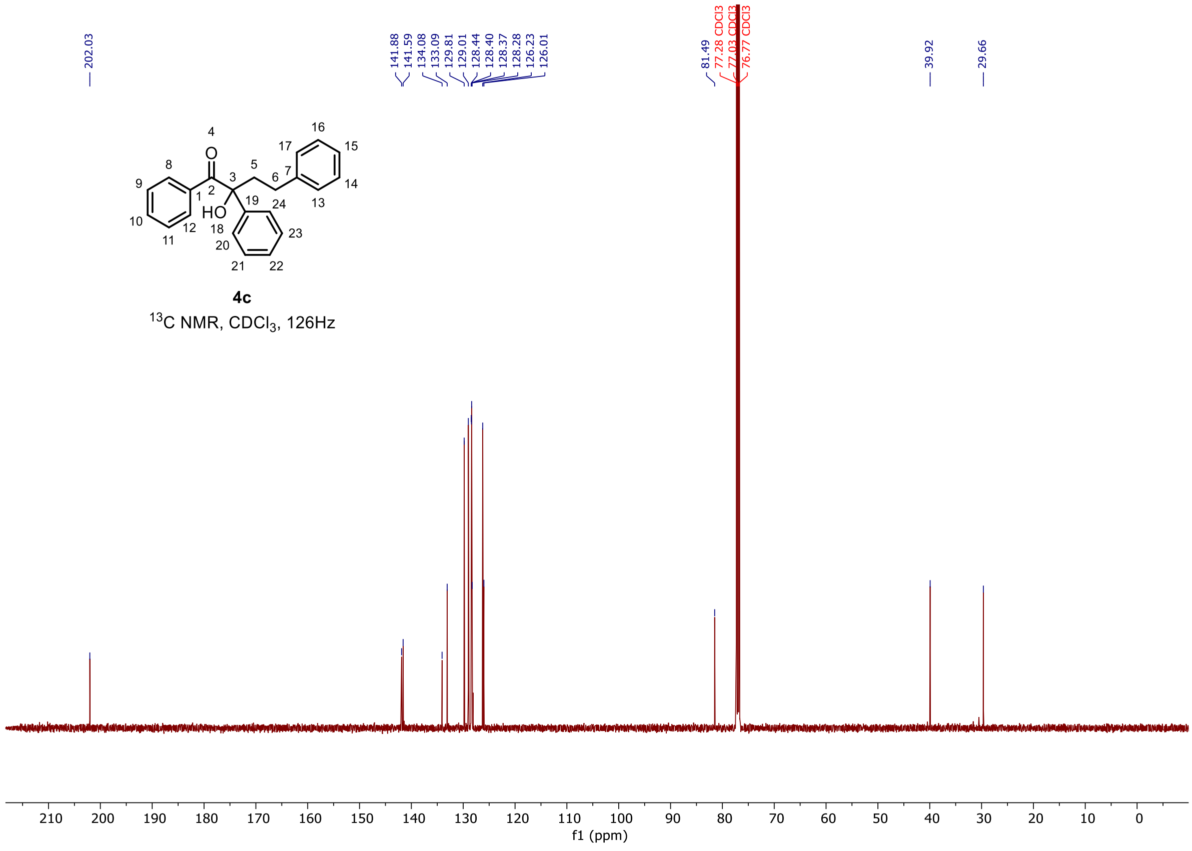

2-Hydroxy-1,2,4-triphenylbutan-1-one 4c and 1-Hydroxy-1,1,4-triphenylbutan-2-one 4c'

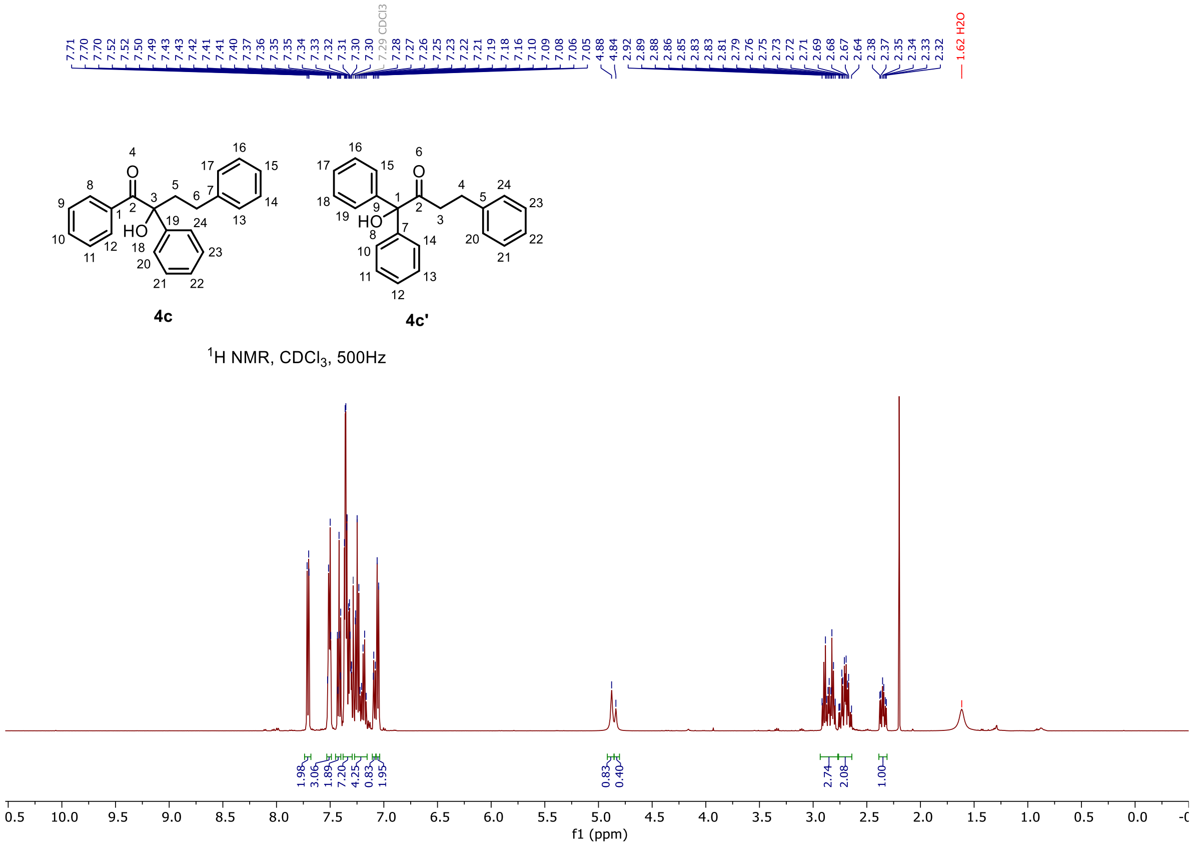

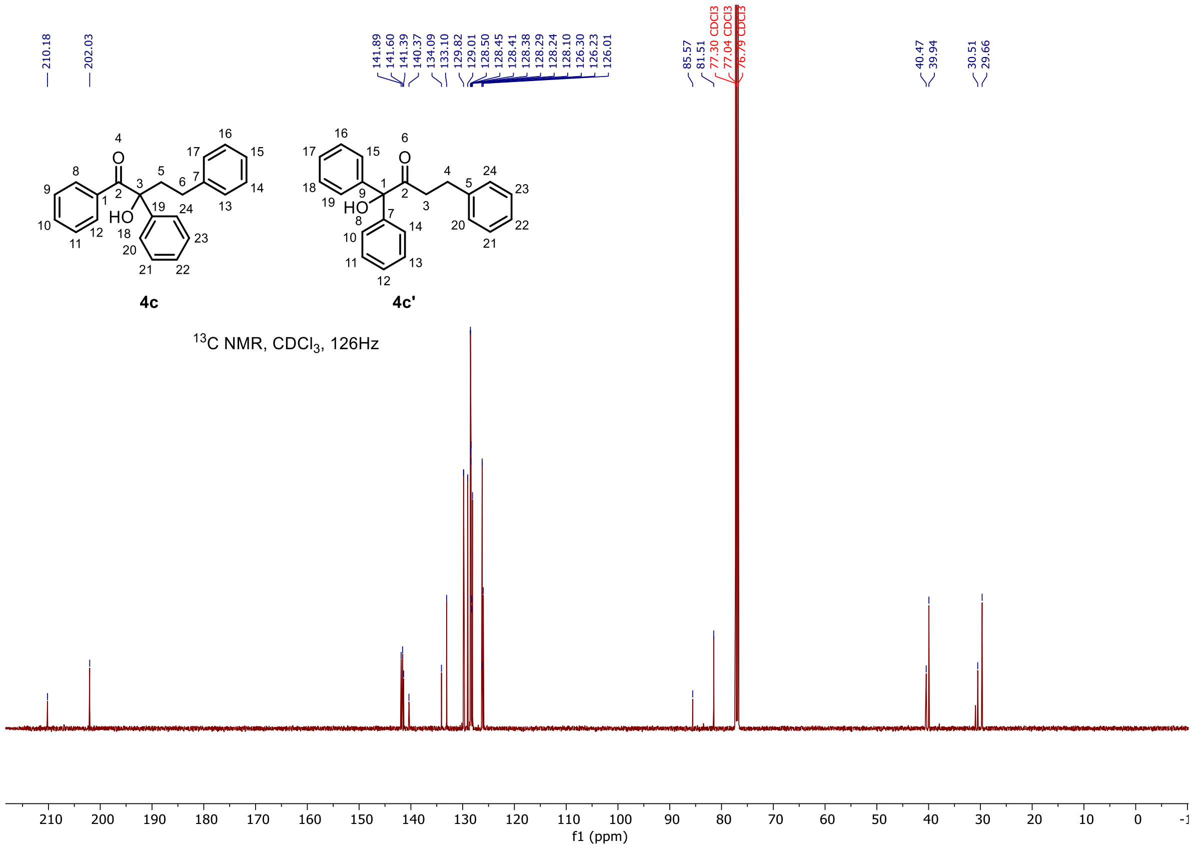

2-Hydroxy-1,2,3-triphenylbutan-1-one 3c and 1-Hydroxy-1,1,3-triphenylbutan-2-one 3c'

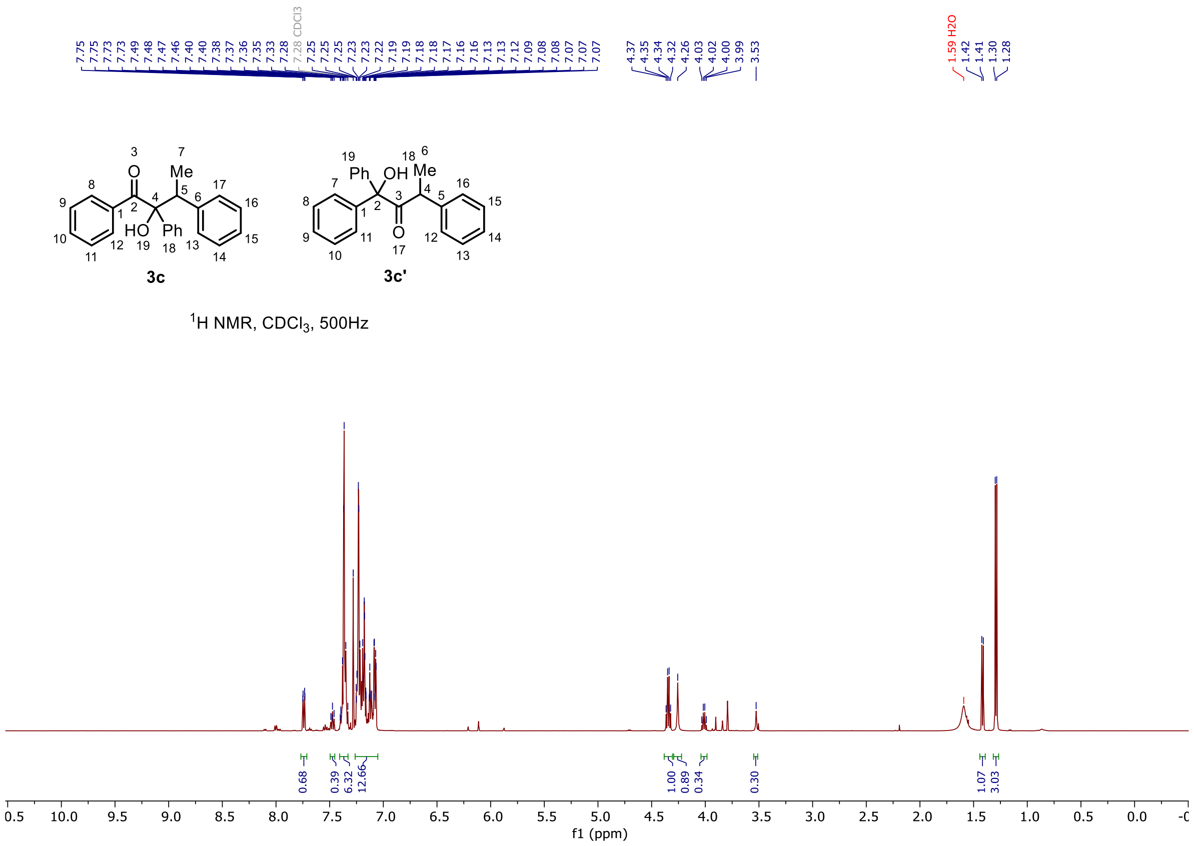

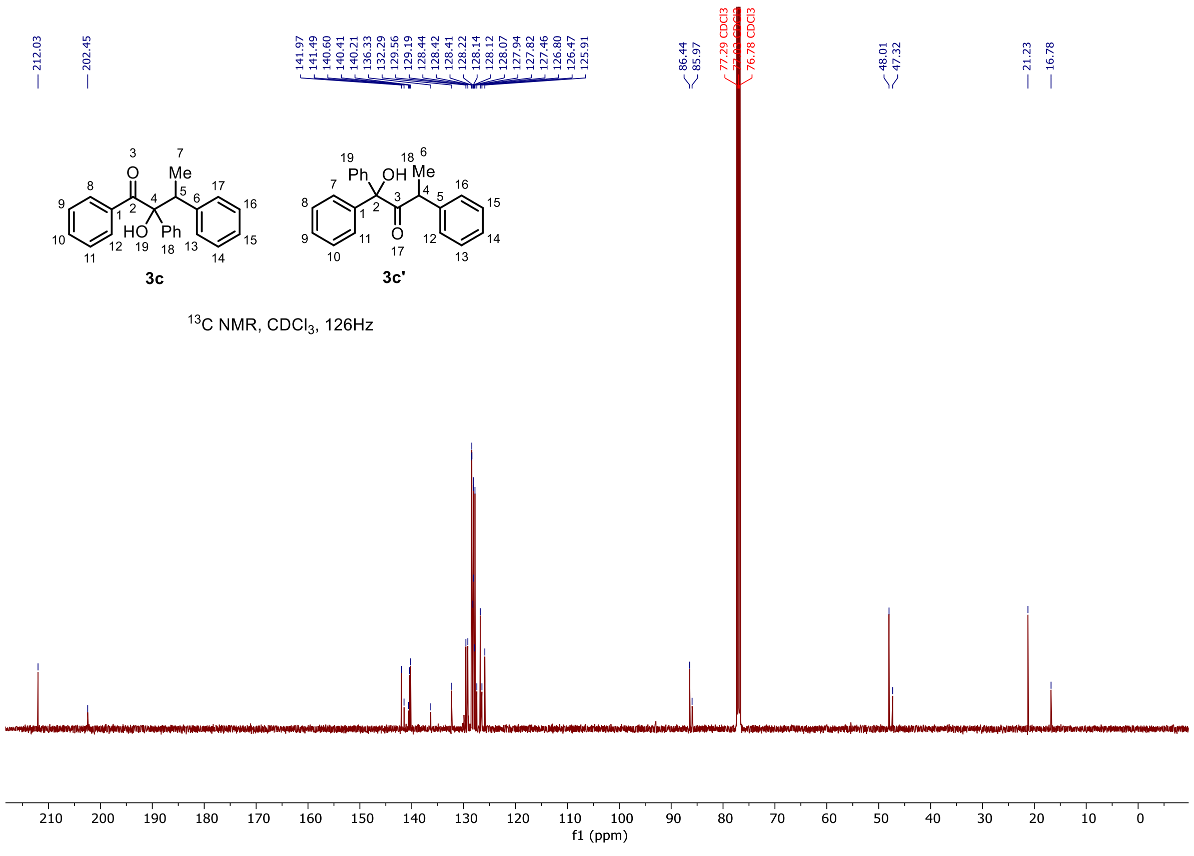

Substrates

2-Hydroxy-1-phenylhept-6-en-1-one 5a

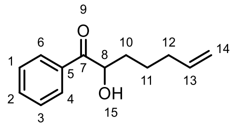

5a

^{1}H NMR, $CDCl_3$, 500 Hz

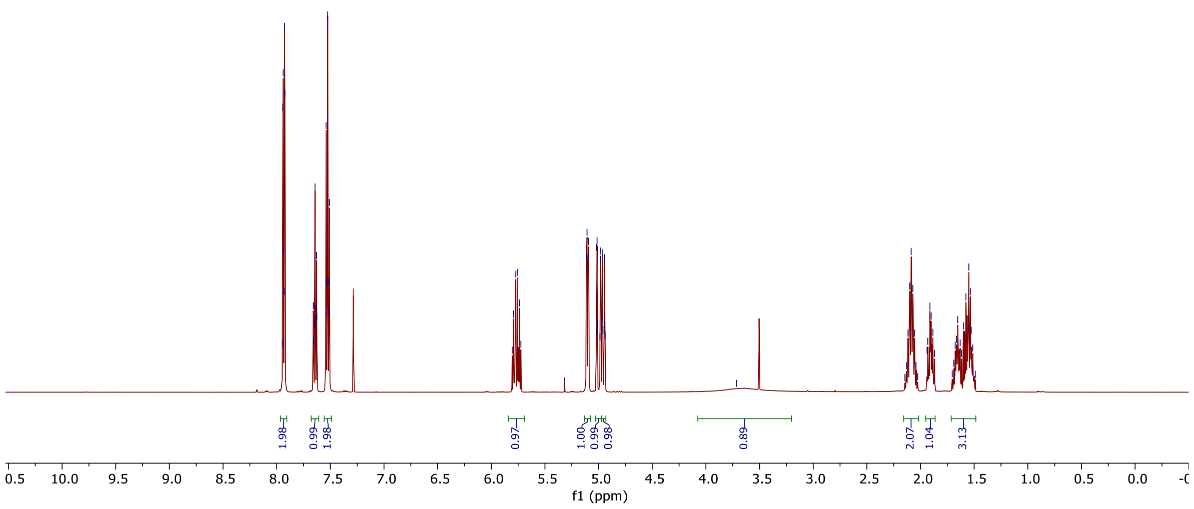

2-Hydroxy-1-(p-tolyl)hept-6-en-1-one 5b

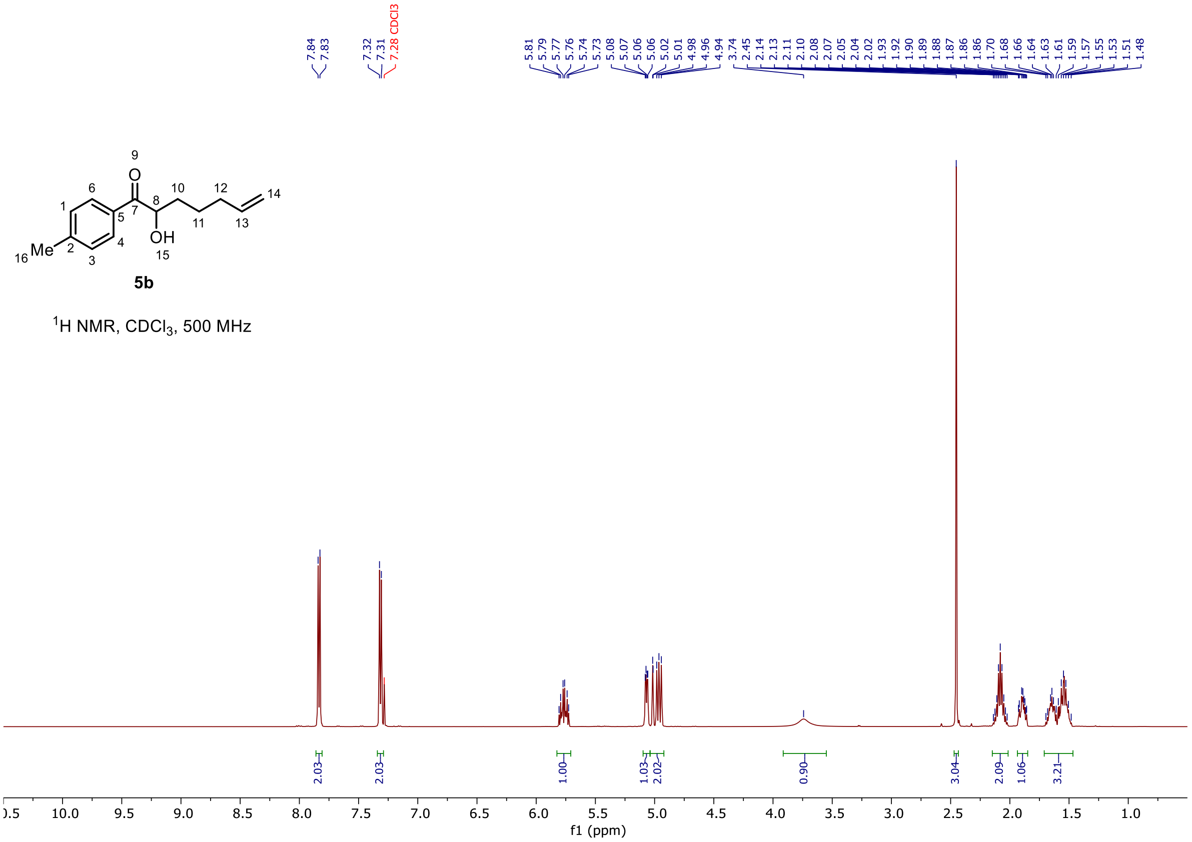

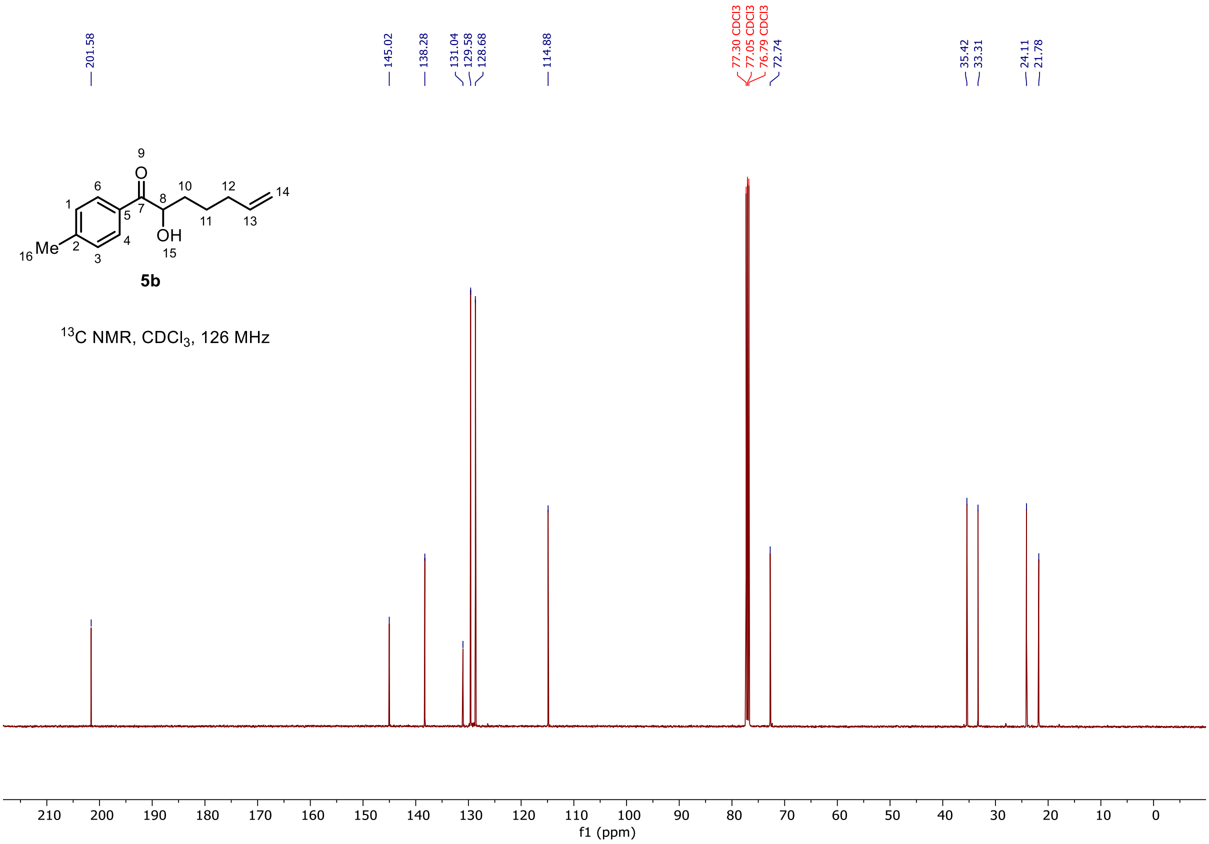

1-(4-Chlorophenyl)-2-hydroxyhept-6-en-1-one 5c

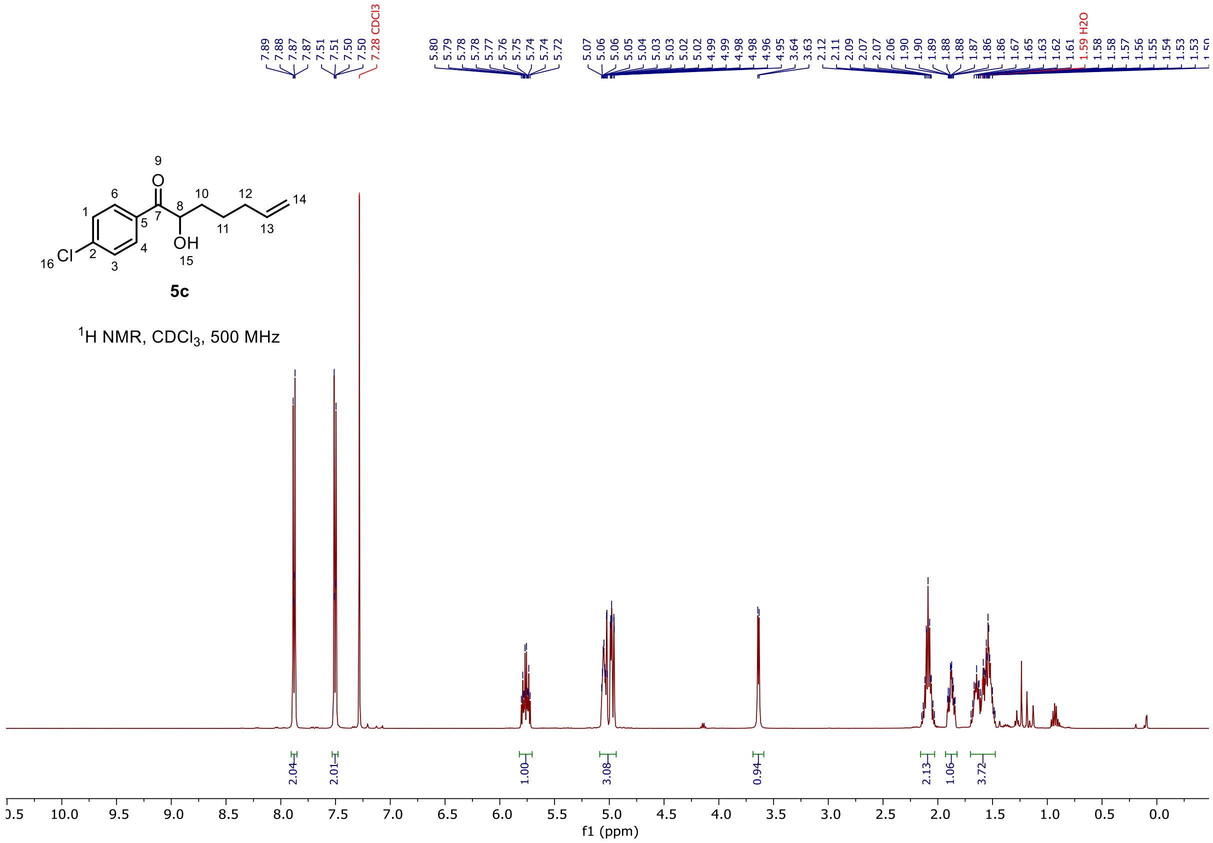

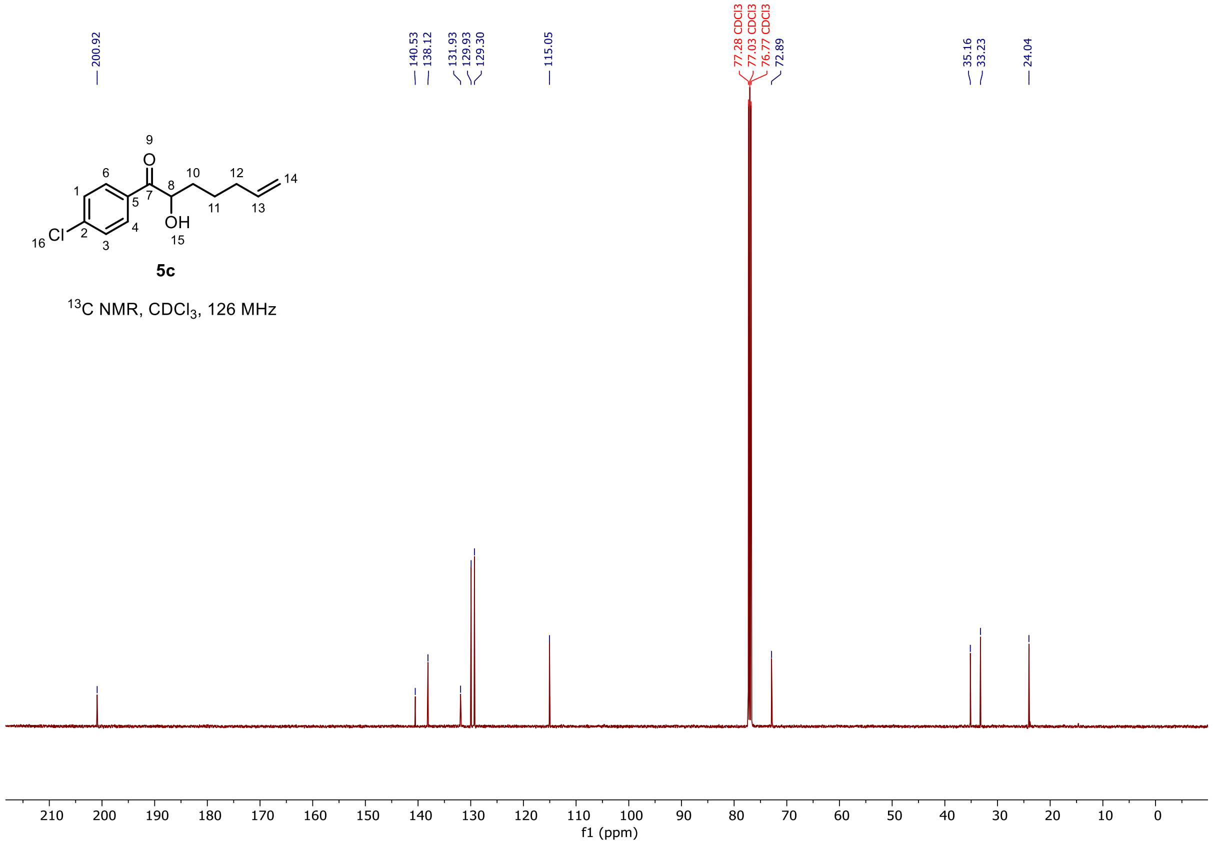

1-(2-Fluorophenyl)-2-hydroxyhept-6-en-1-one 5d

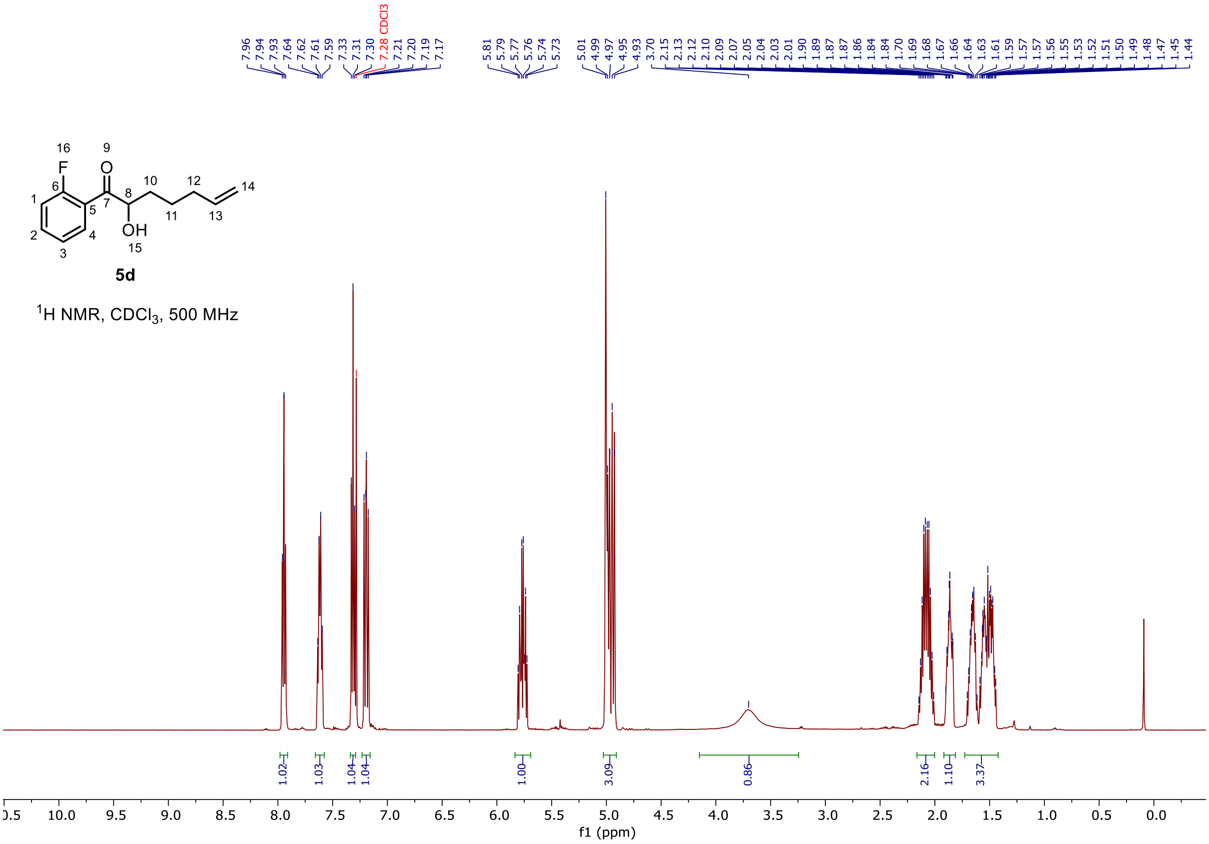

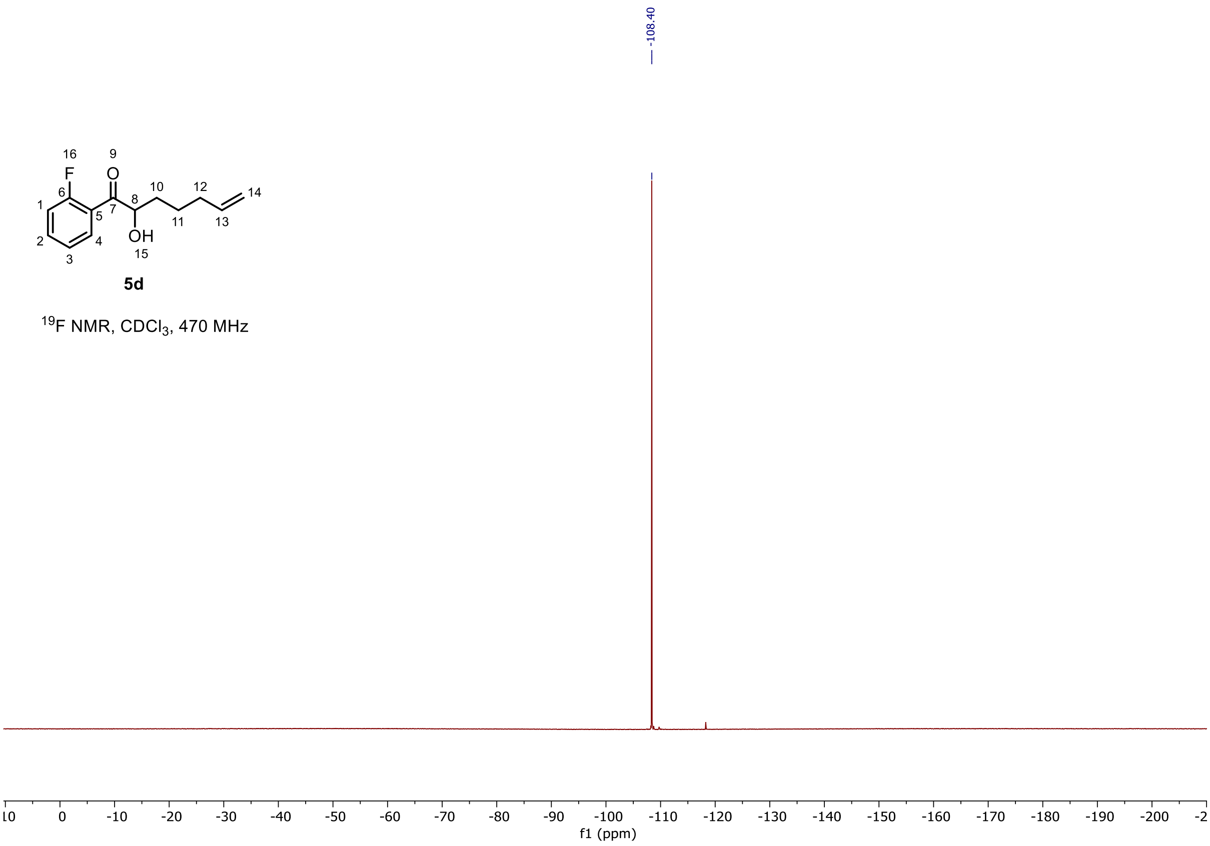

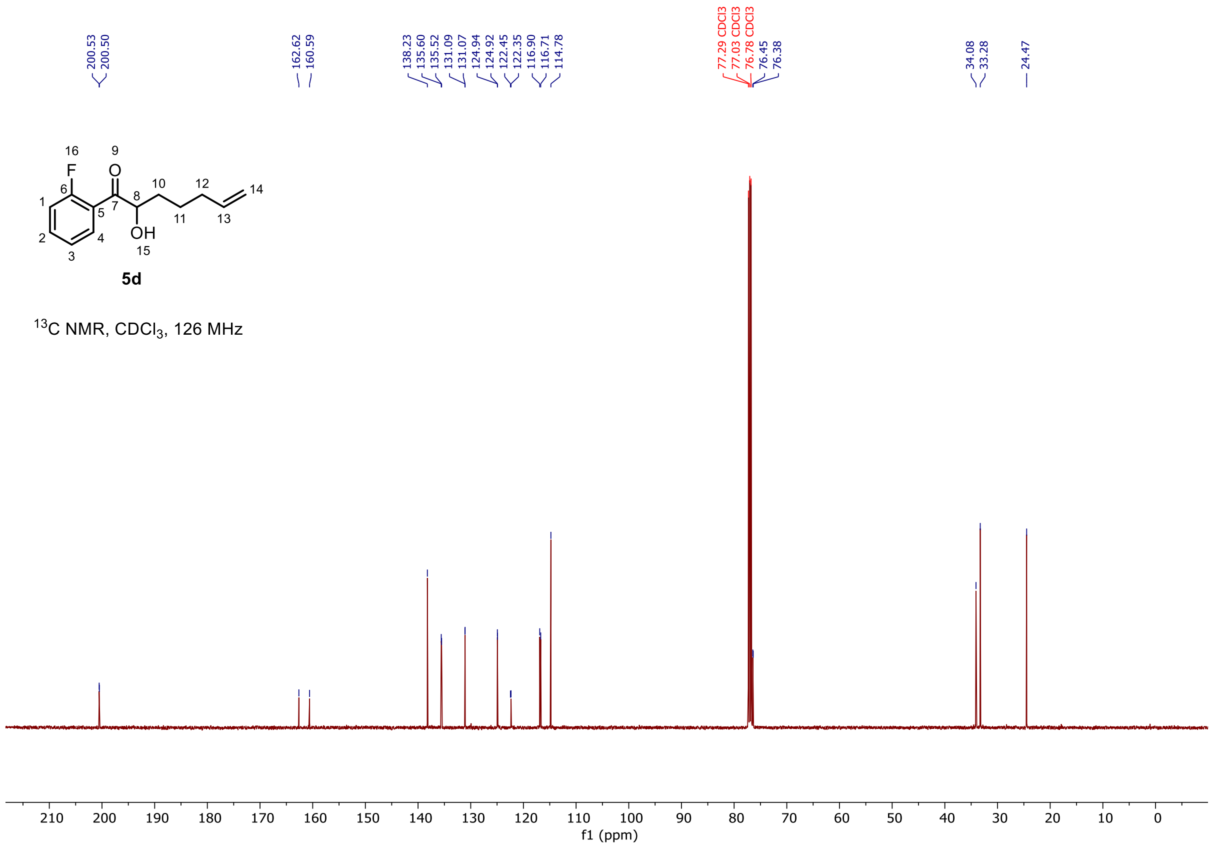
200.53
200.50
162.62
160.59
138.23
135.60
135.52
131.09
131.07
124.94
124.92
122.45
122.35
116.90
116.71
114.78
77.29 CDCl3
77.03 CDCl3
76.78 CDCl3
76.45
76.38
34.08
33.28
24.47
5d
^{13}C NMR, $CDCl_3$, 126 MHz
f1 (ppm)

2-Hydroxy-1-(3-methoxyphenyl)hept-6-en-1-one 5e

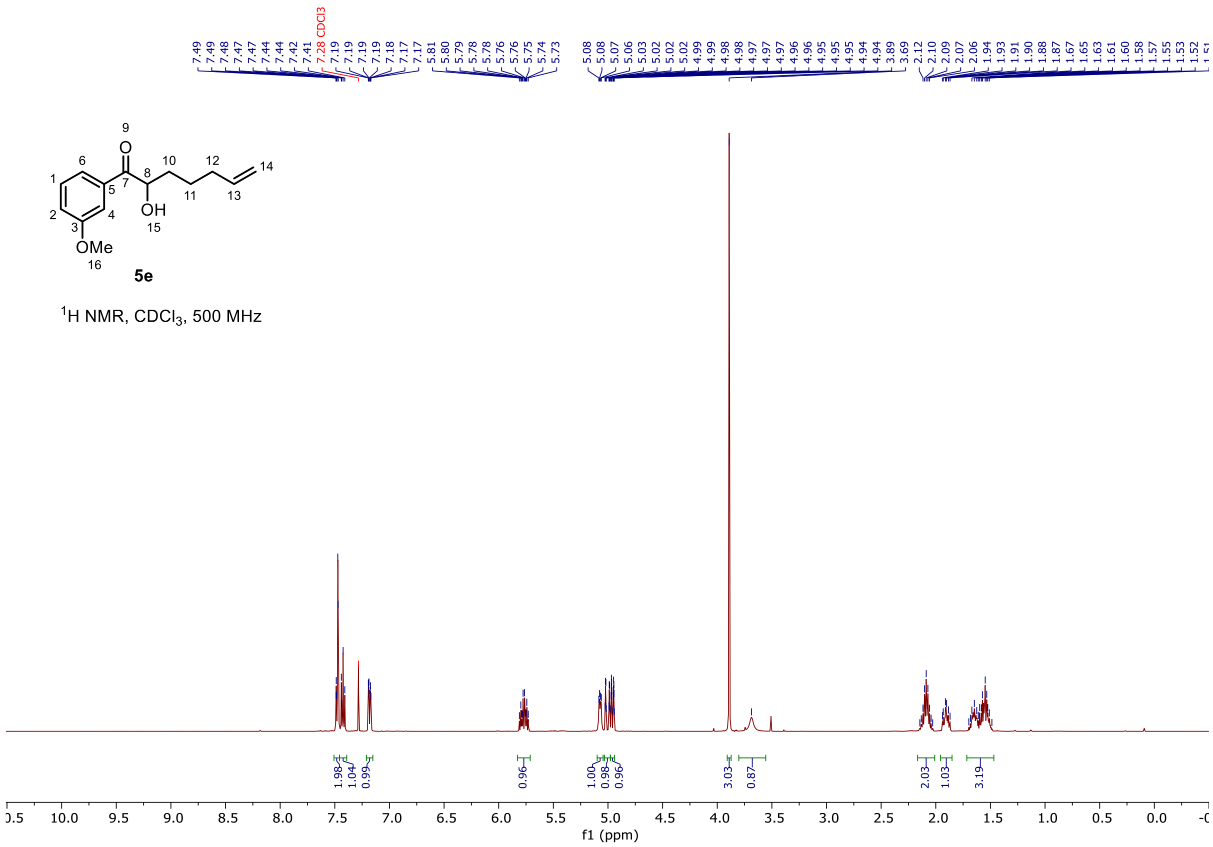

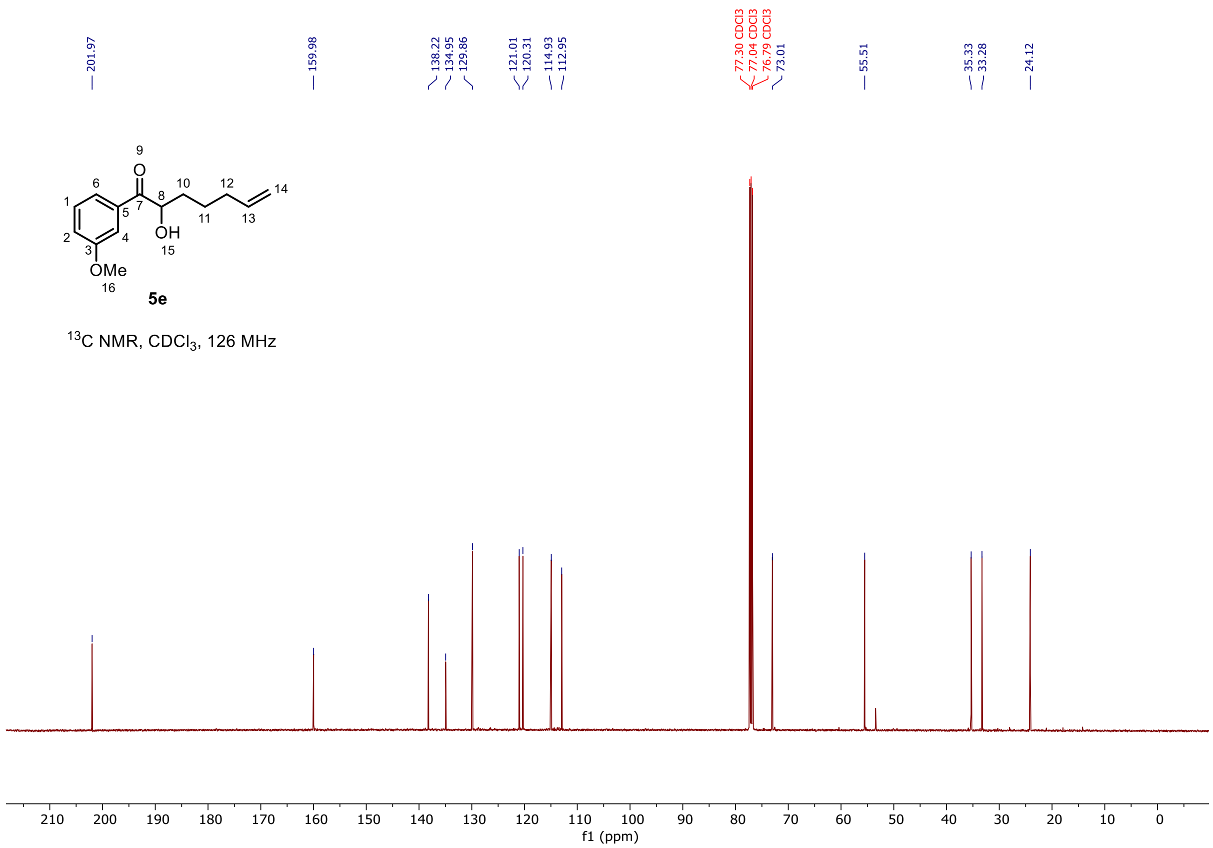

2-Hydroxy-1-(3-(trifluoromethyl)phenyl)hept-6-en-1-one 5f

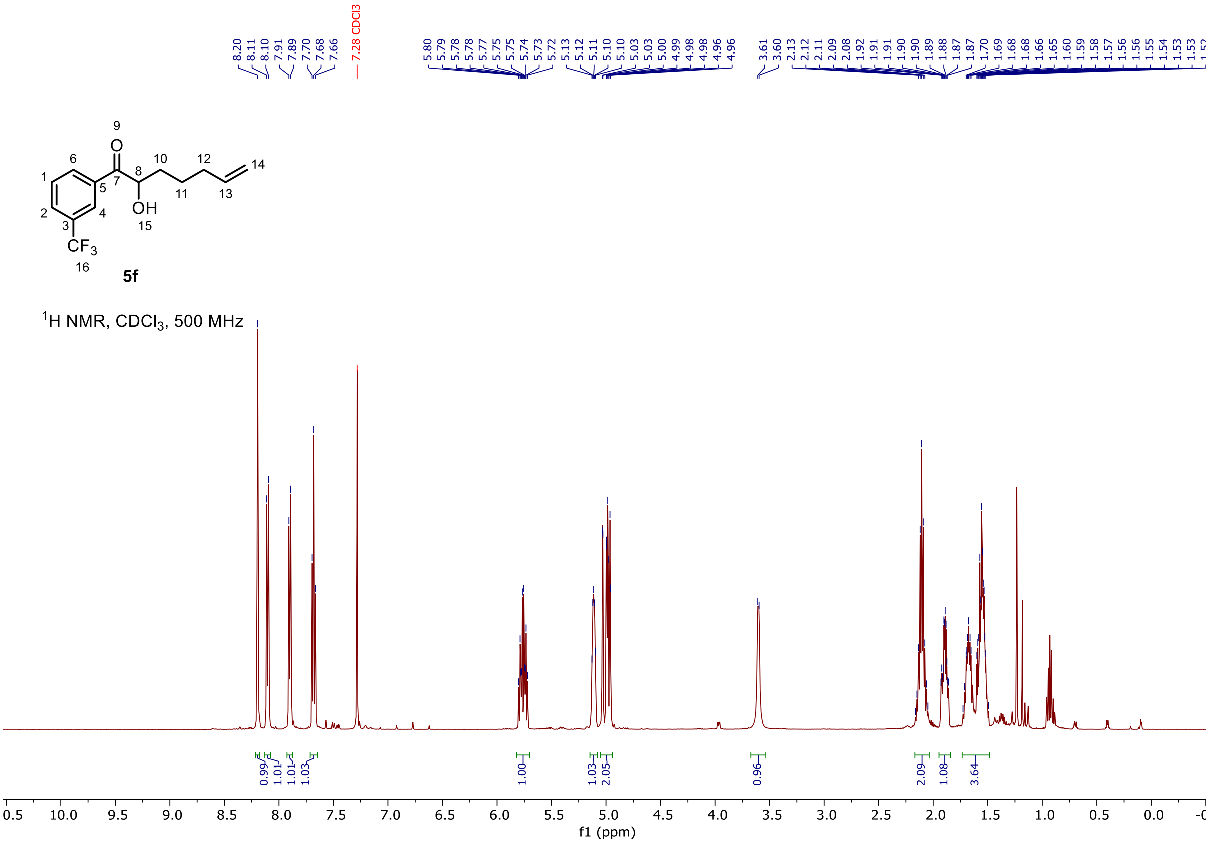

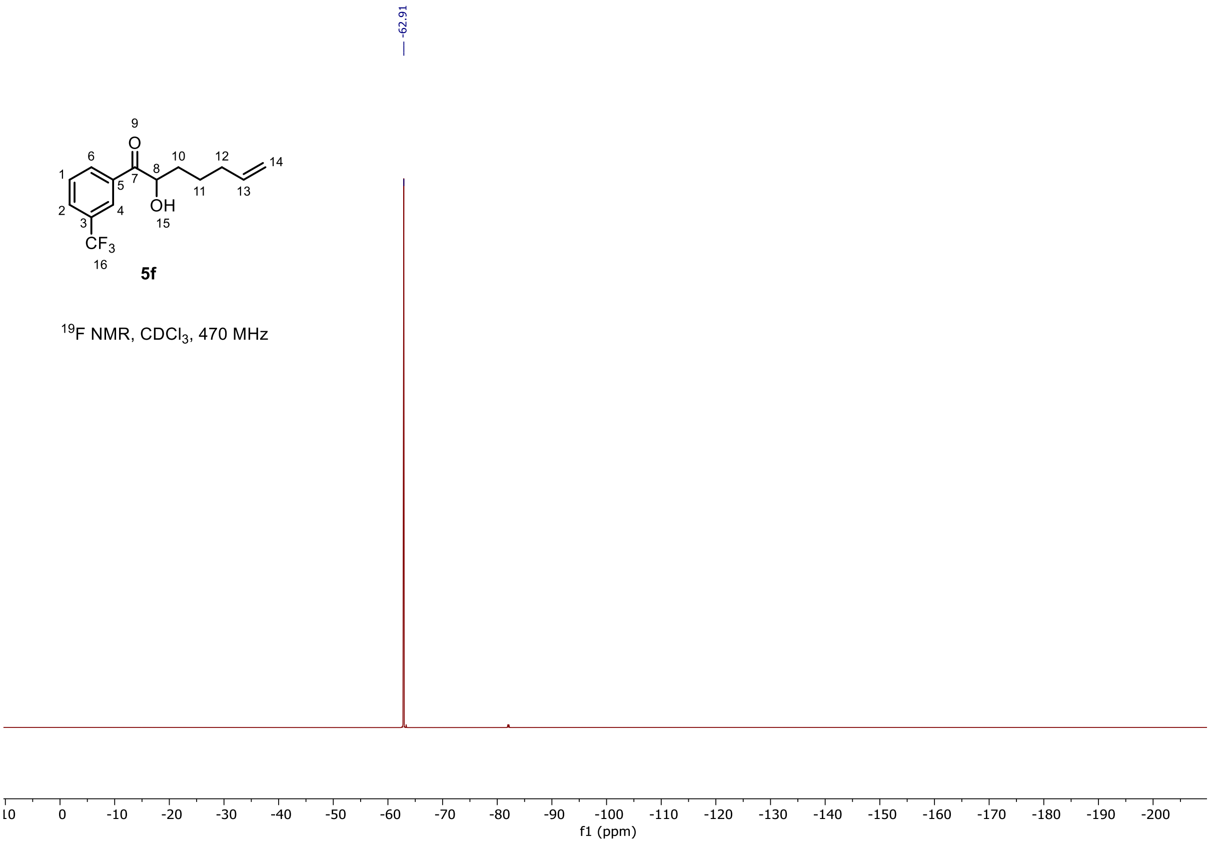

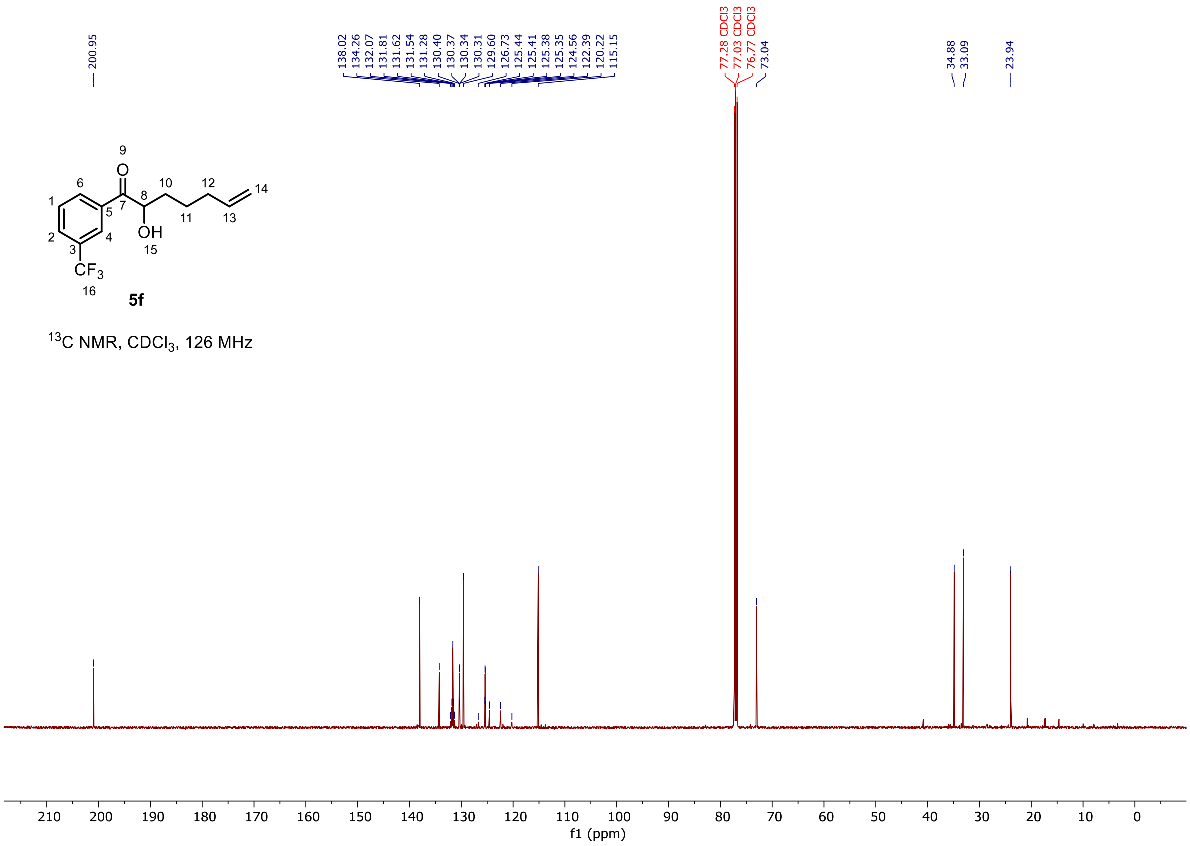
200.95
138.02
134.26
132.07
131.81
131.62
131.54
131.28
130.40
130.37
130.34
130.31
129.60
126.73
125.44
125.41
125.38
125.35
124.56
122.39
120.22
115.15
77.28 CDCl3
77.03 CDCl3
76.77 CDCl3
73.04
34.88
33.09
23.94
5f
^{13}C NMR, CDCl$_3$, 126 MHz
f1 (ppm)

2-Hydroxy-1-(naphthalen-2-yl)hept-6-en-1-one 5g

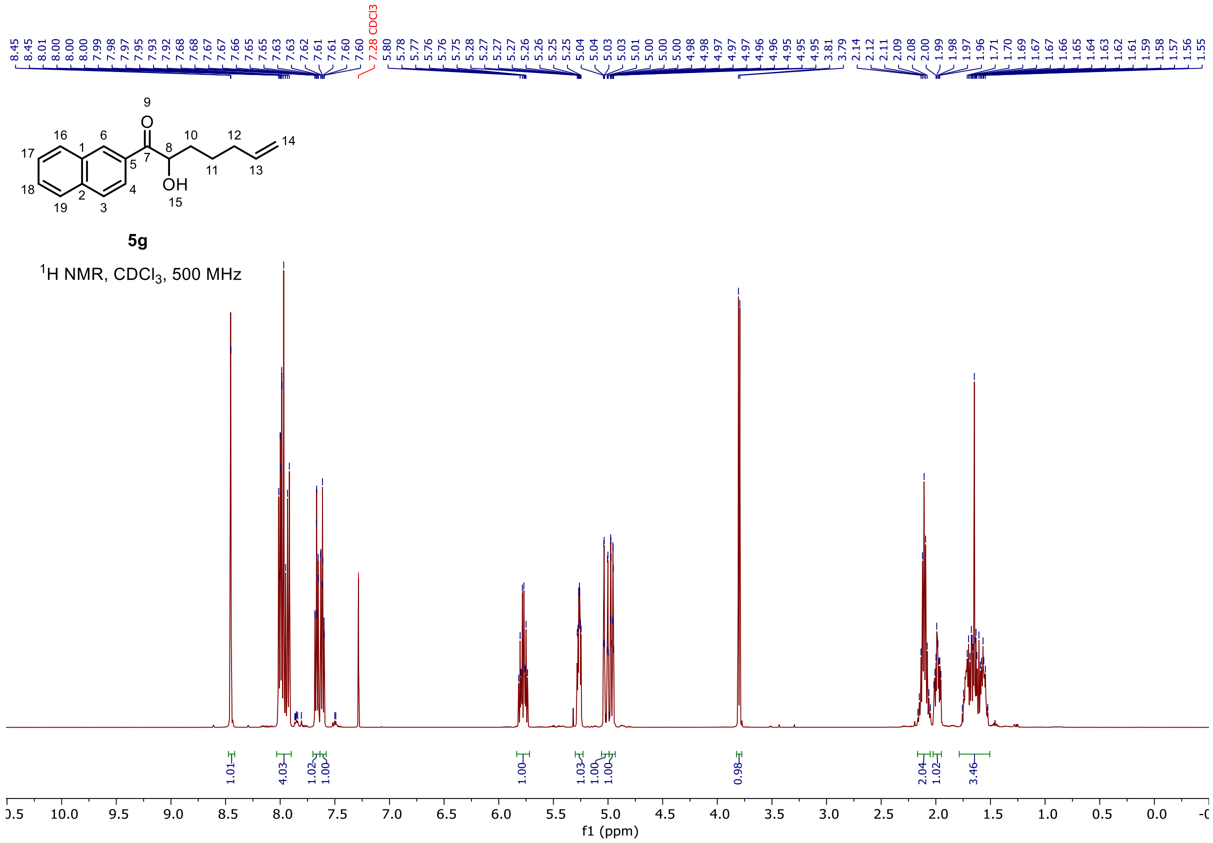

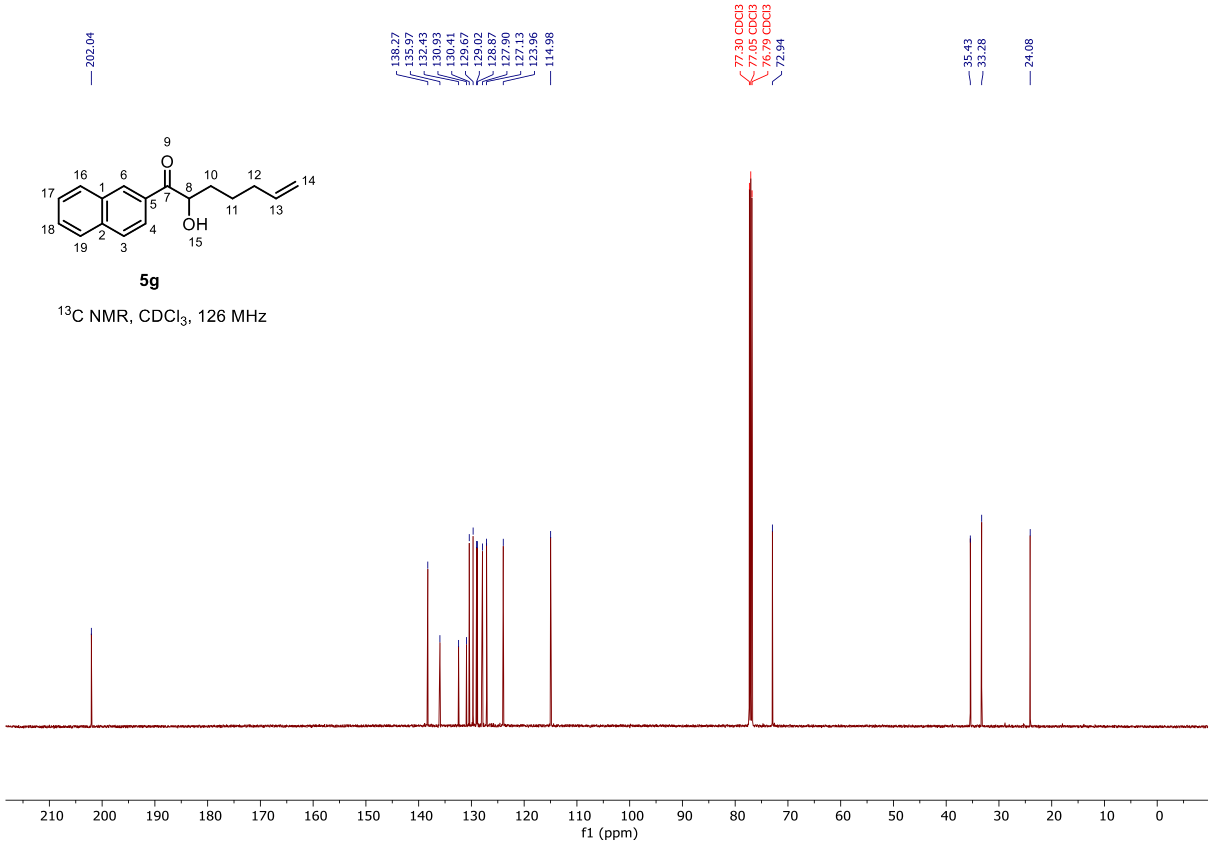

1-(Furan-2-yl)-2-hydroxyhept-6-en-1-one 5h

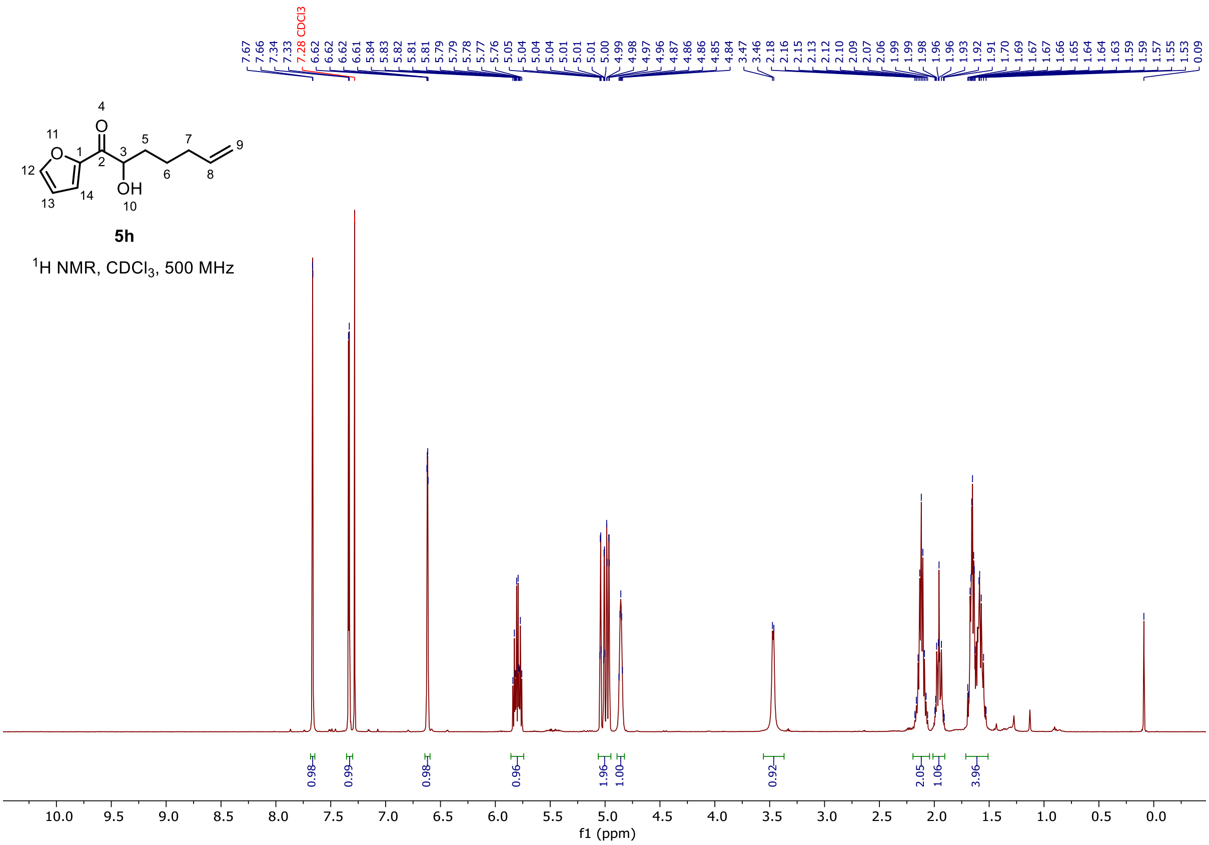

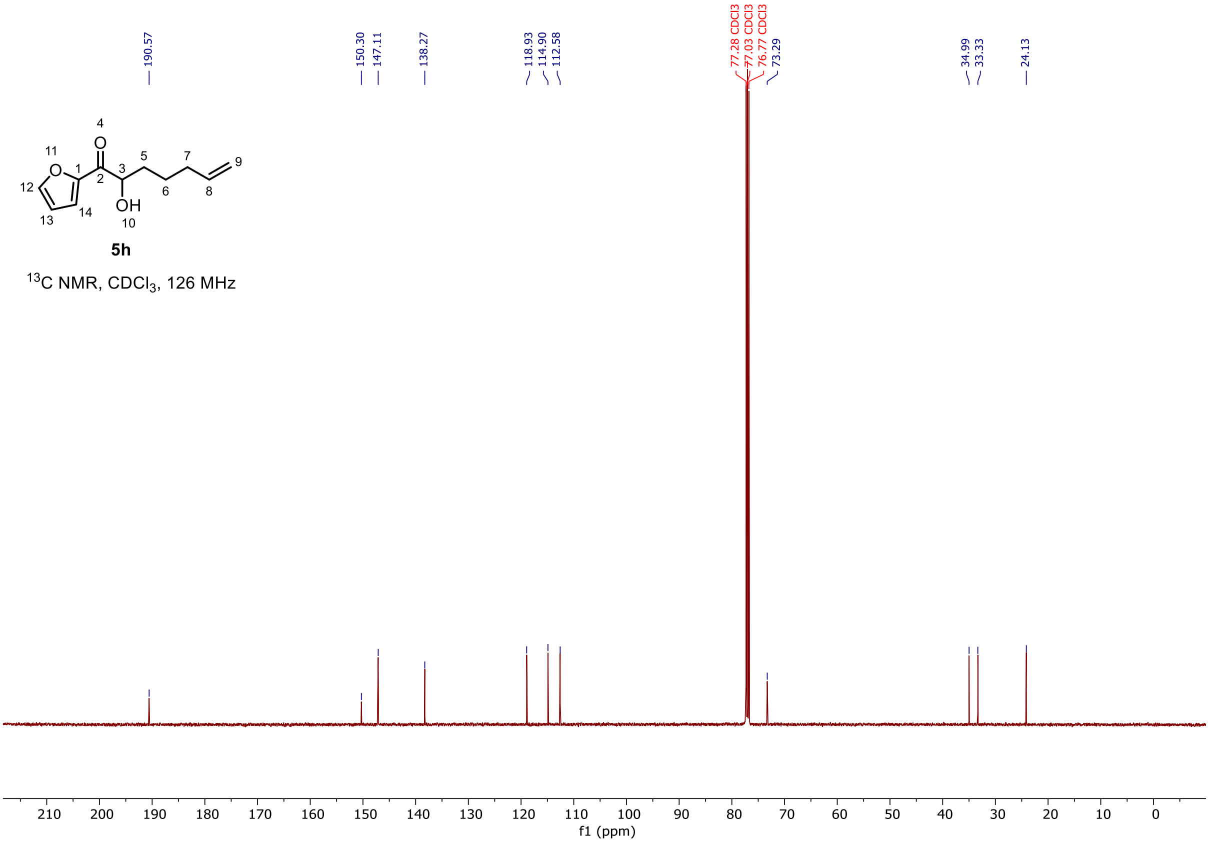

2-Hydroxy-1-(1-methyl-1*H*-indol-2-yl)hept-6-en-1-one 5i

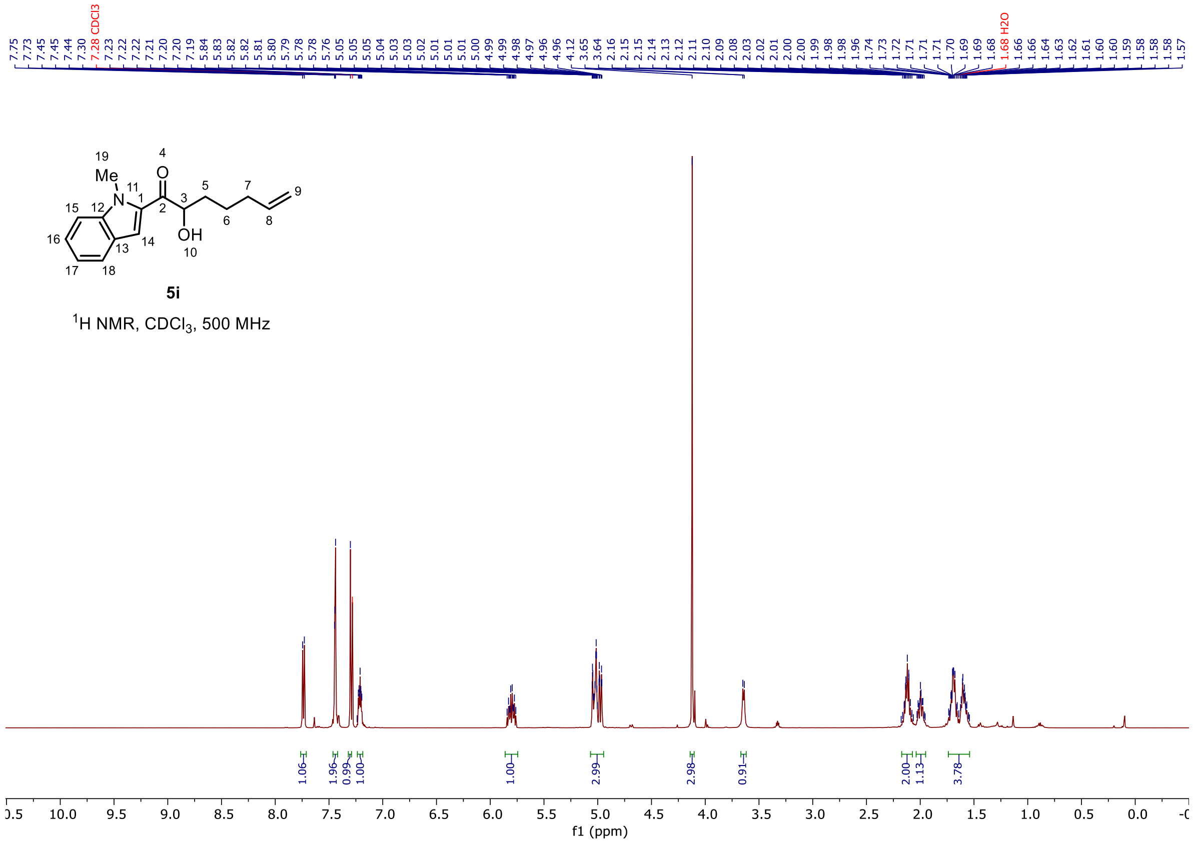

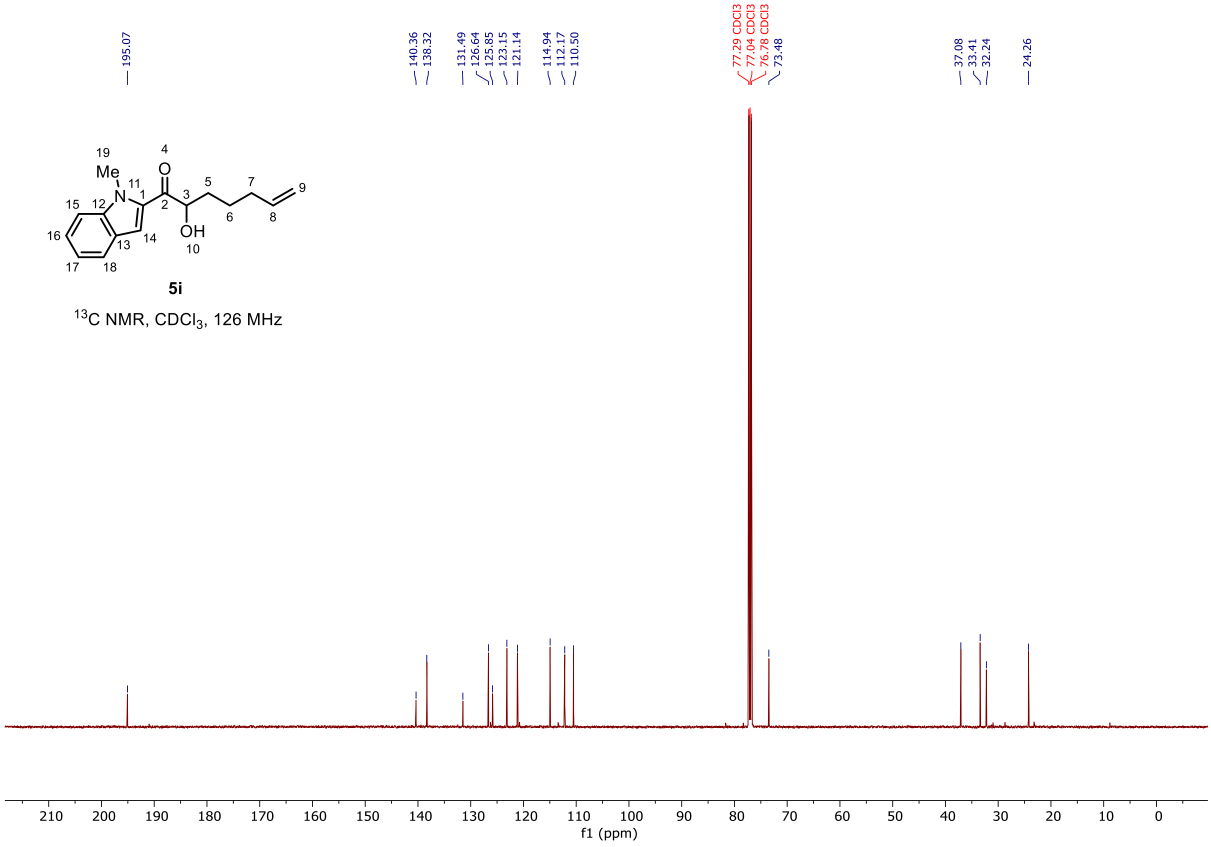

1-(3-(Cyclopropylmethoxy)-4-(difluoromethoxy)phenyl)-2-hydroxyhept-6-en-1-one 5j

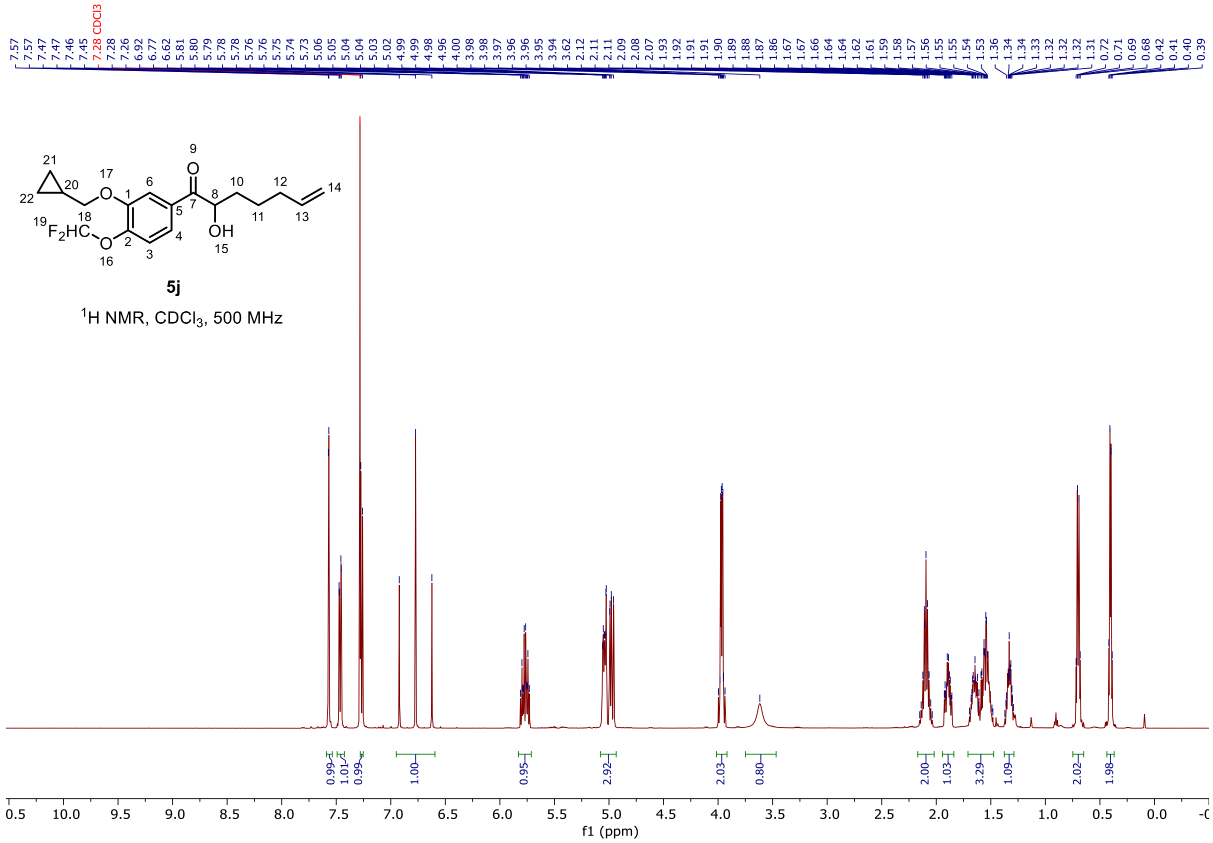

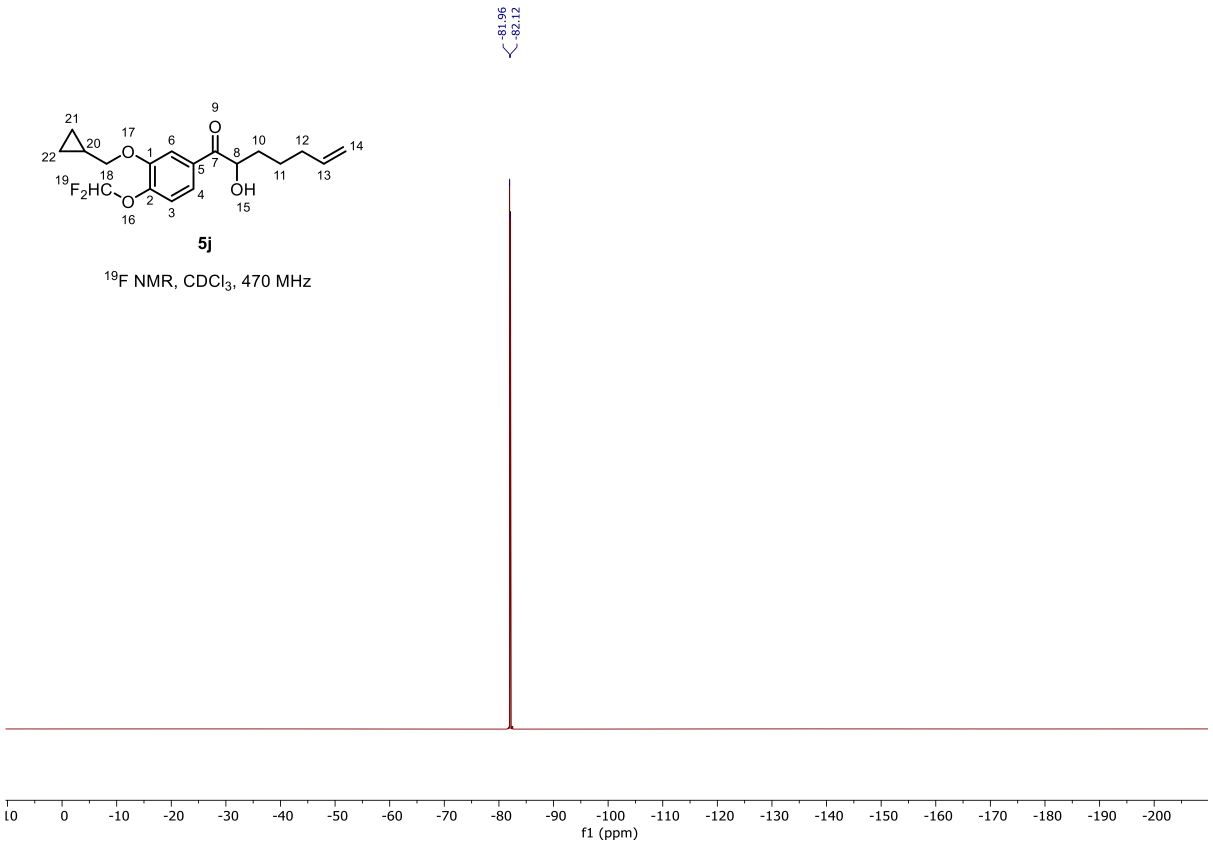

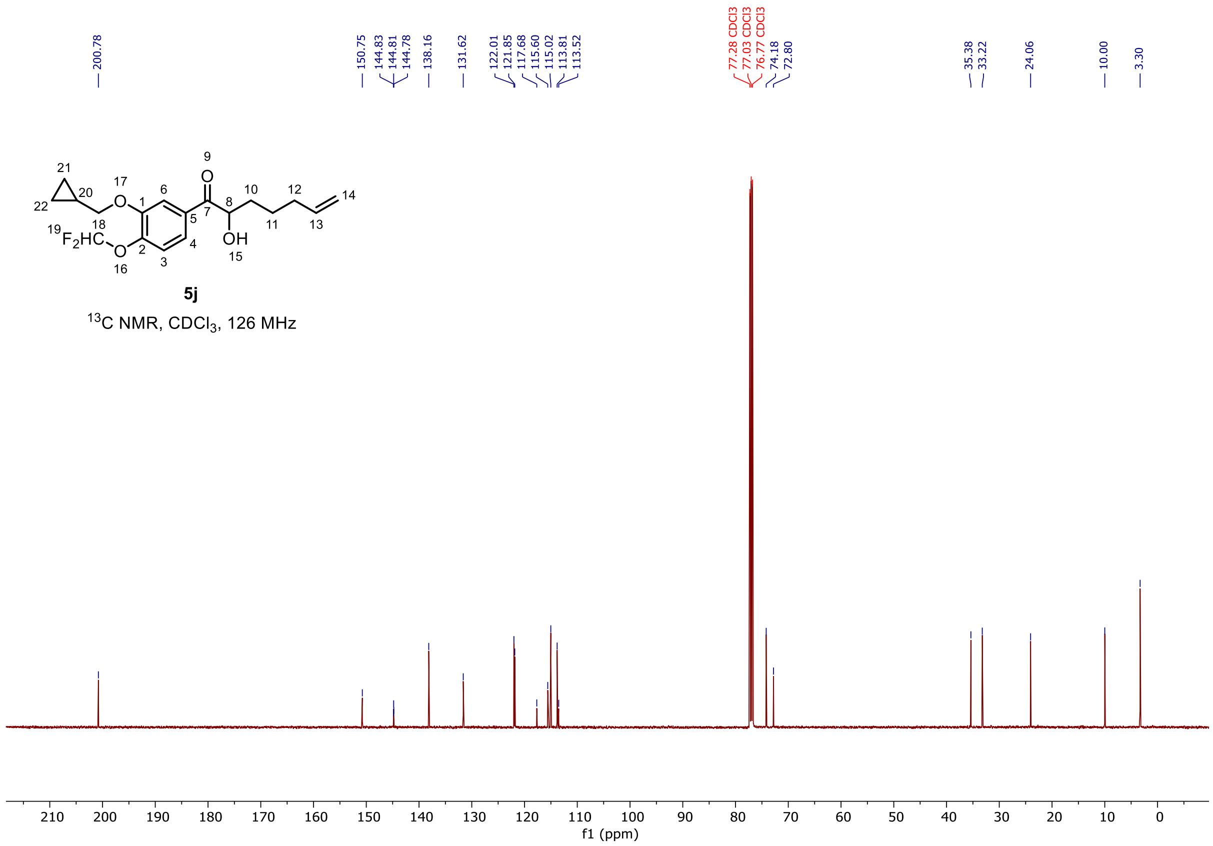
200.78
150.75
144.83
144.81
144.78
138.16
131.62
122.01
121.85
117.68
115.60
115.02
113.81
113.52
77.28 CDCl3
77.03 CDCl3
76.77 CDCl3
74.18
72.80
35.38
33.22
24.06
10.00
3.30
5j
^{13}C NMR, CDCl$_3$, 126 MHz
f1 (ppm)

2-Hydroxy-1-phenyloct-7-en-1-one 7a

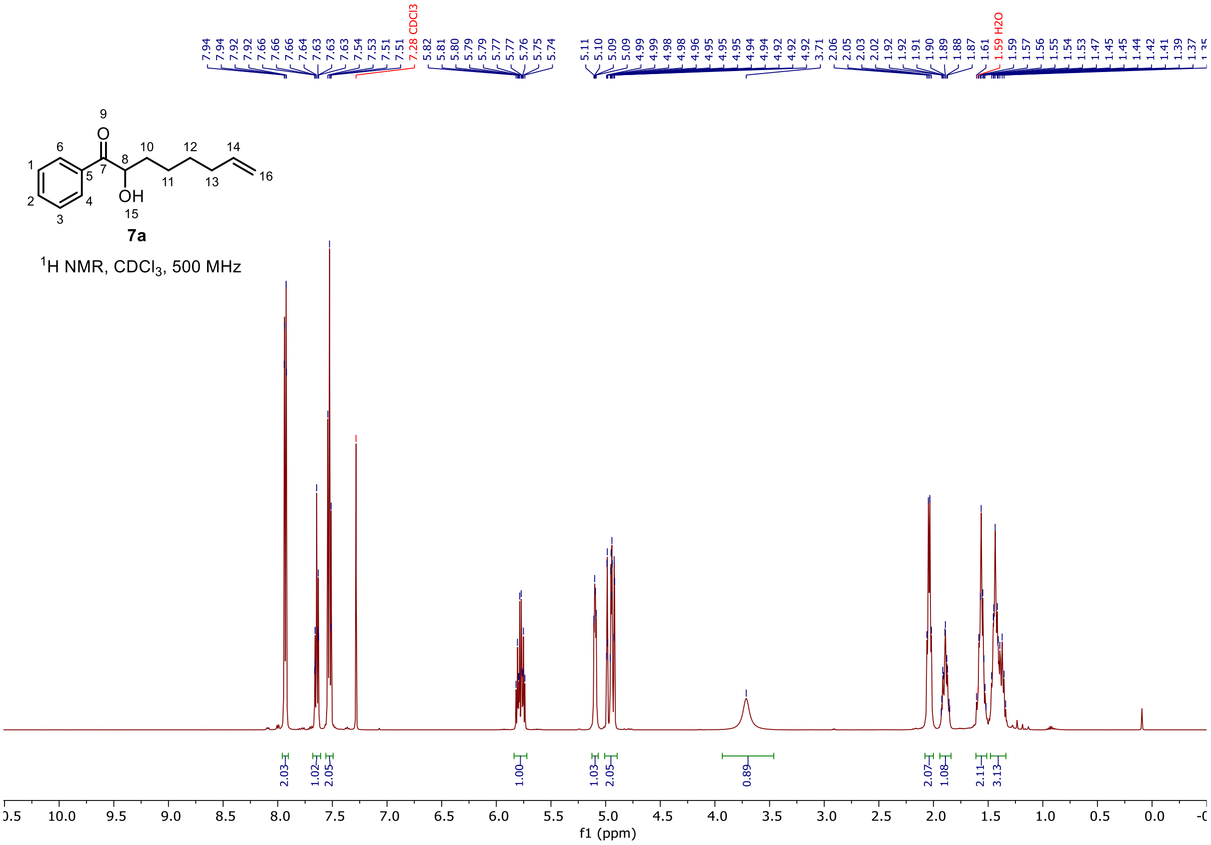

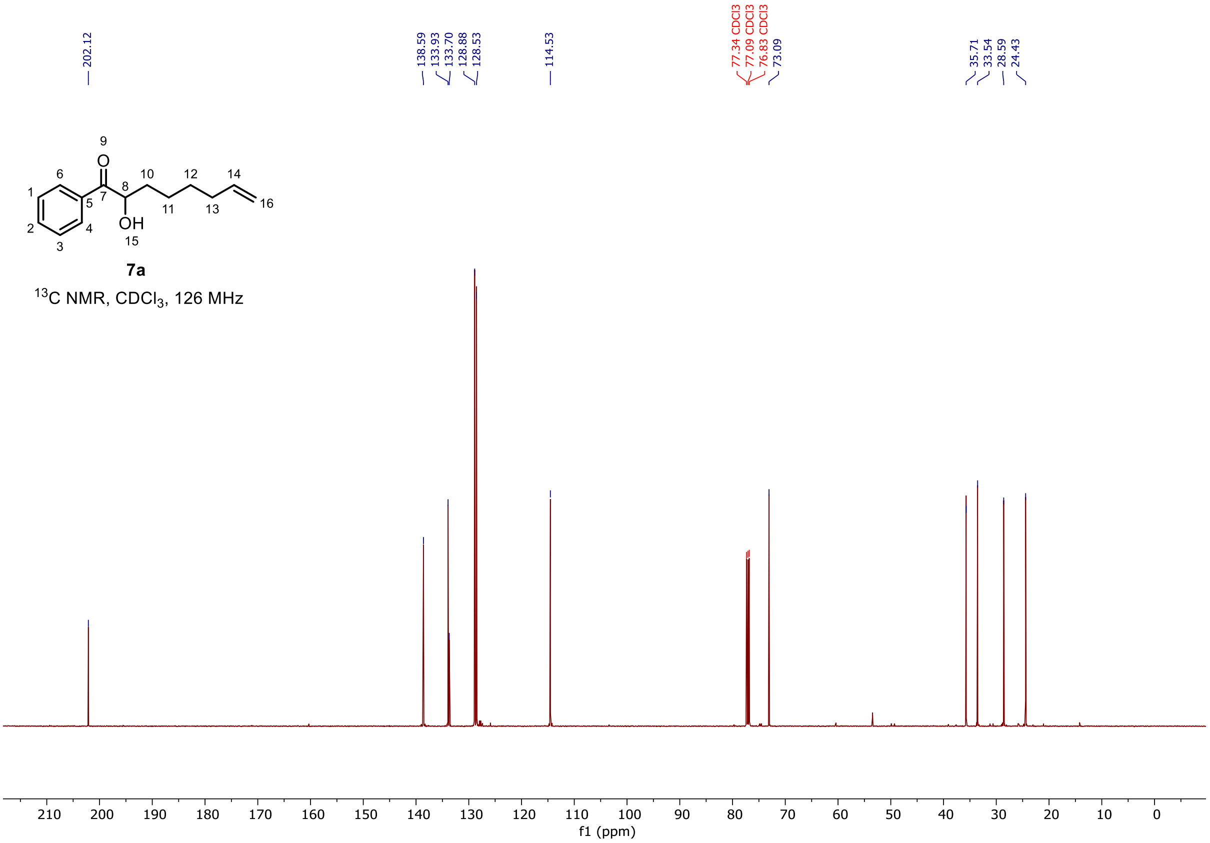

2-Hydroxy-1-(p-tolyl)oct-7-en-1-one 7b

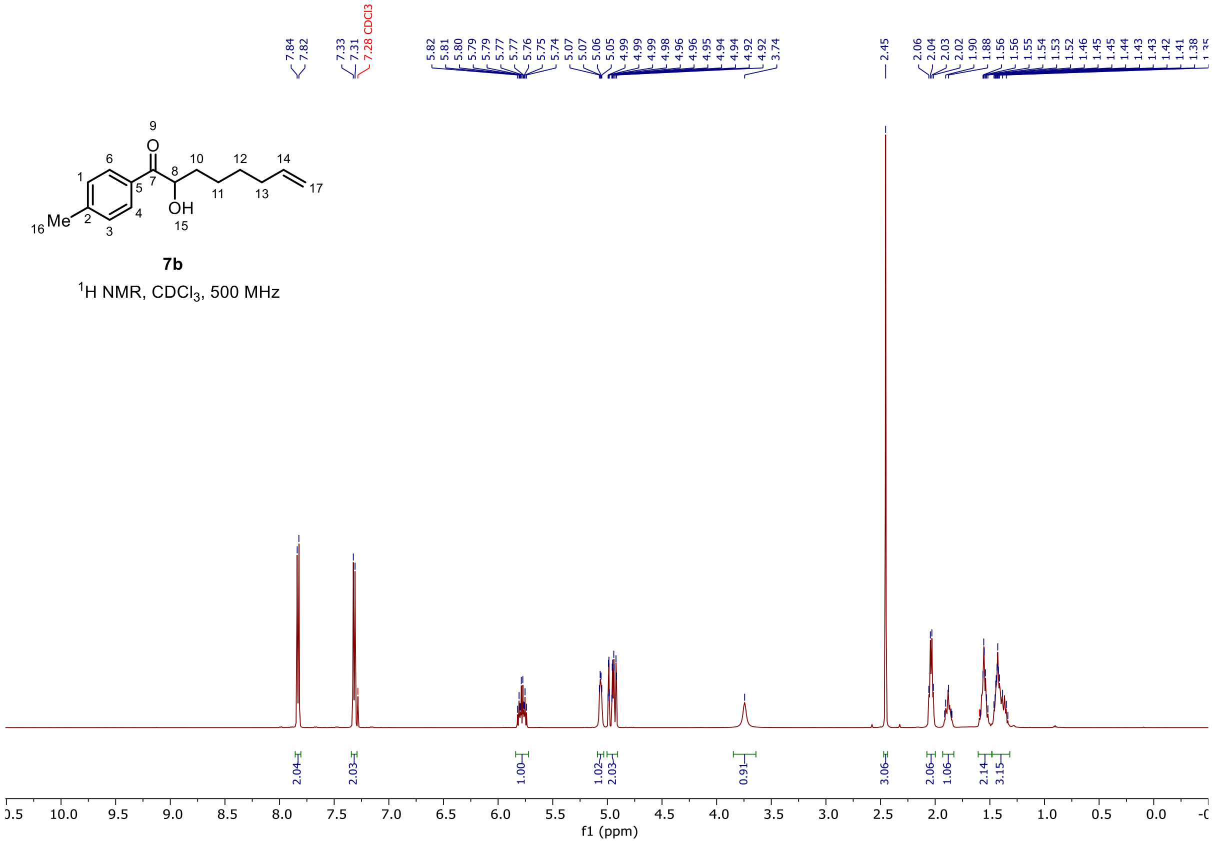

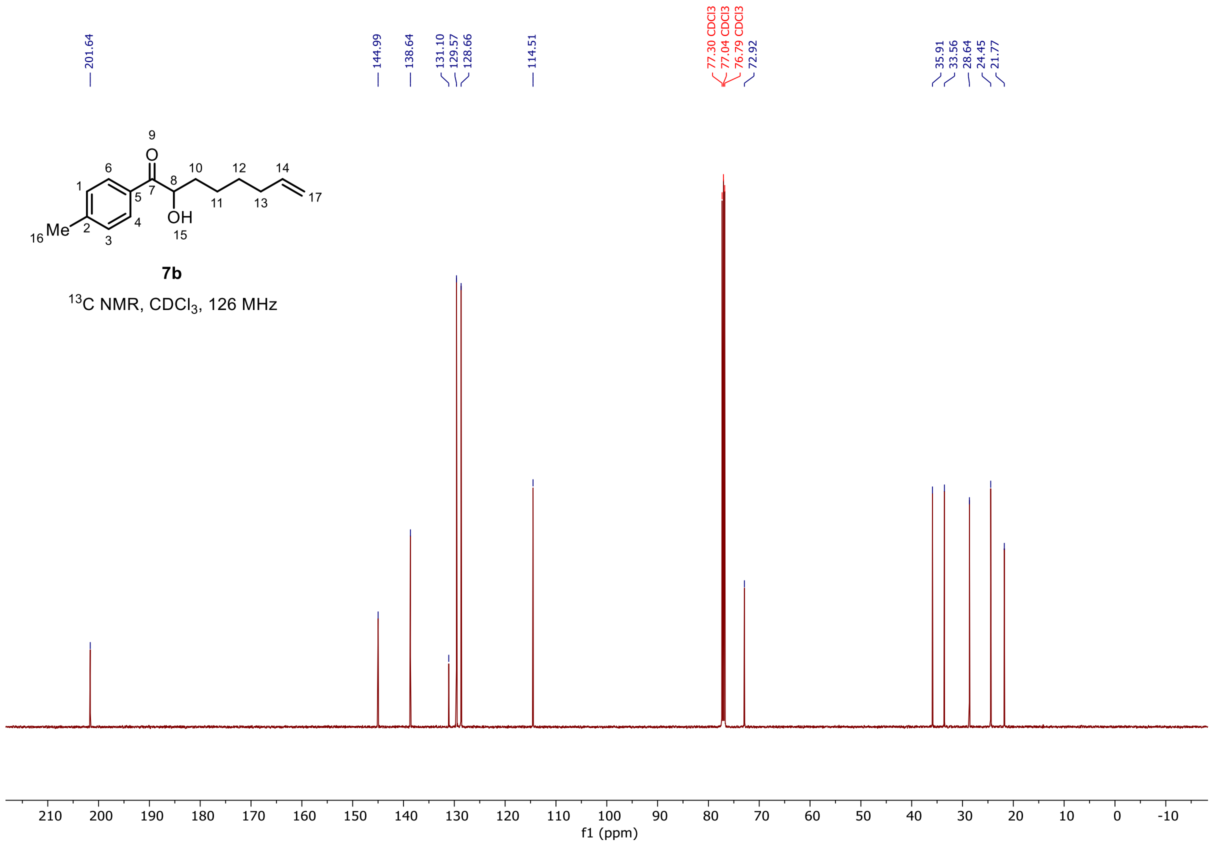

1-(4-Chlorophenyl)-2-hydroxyoct-7-en-1-one 7c

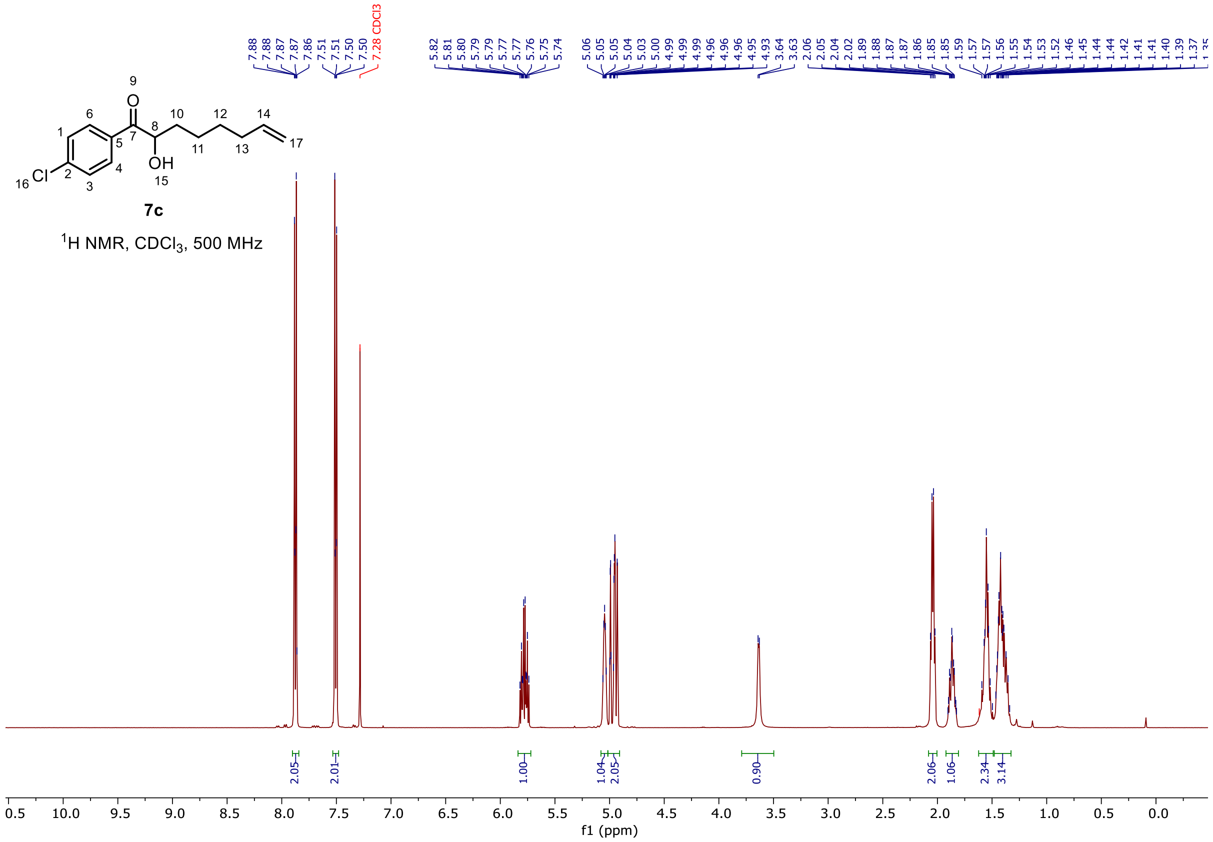

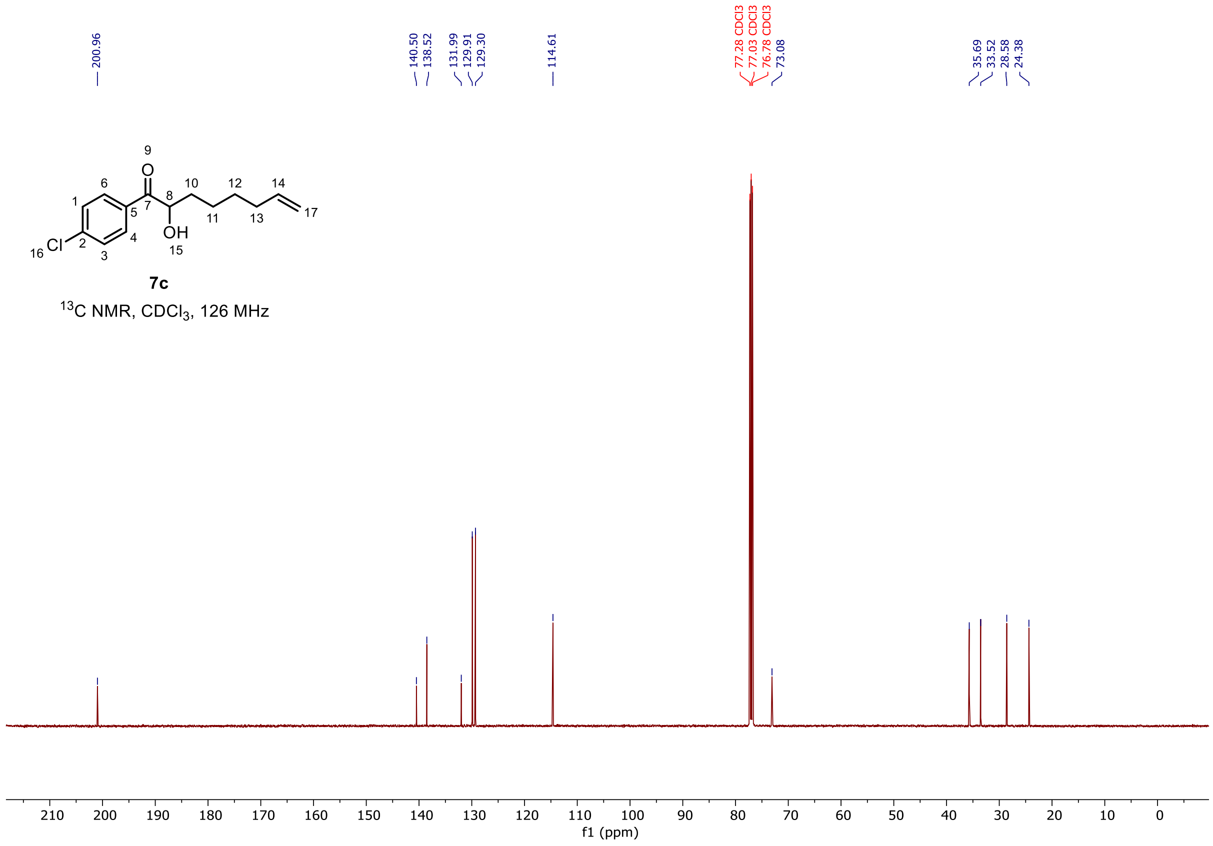

1-(2-Fluorophenyl)-2-hydroxyoct-7-en-1-one 7d

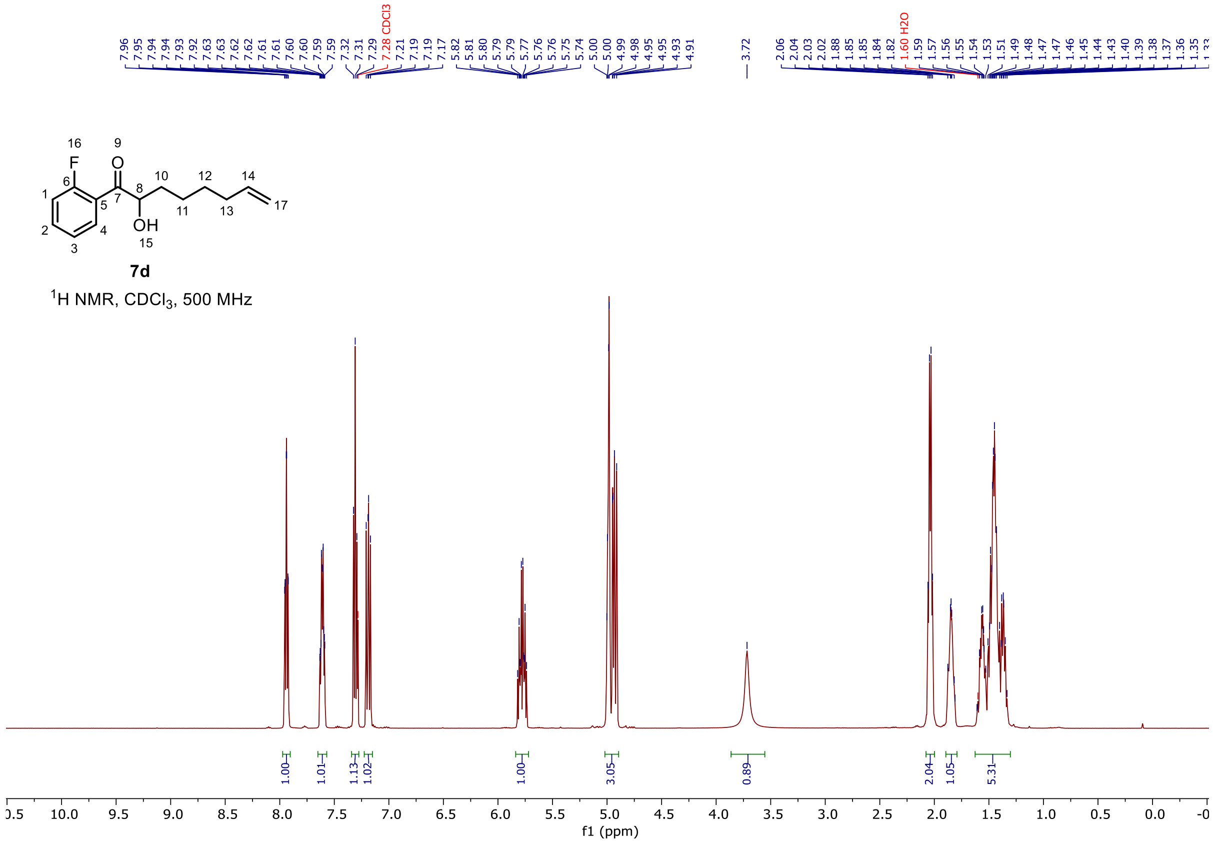

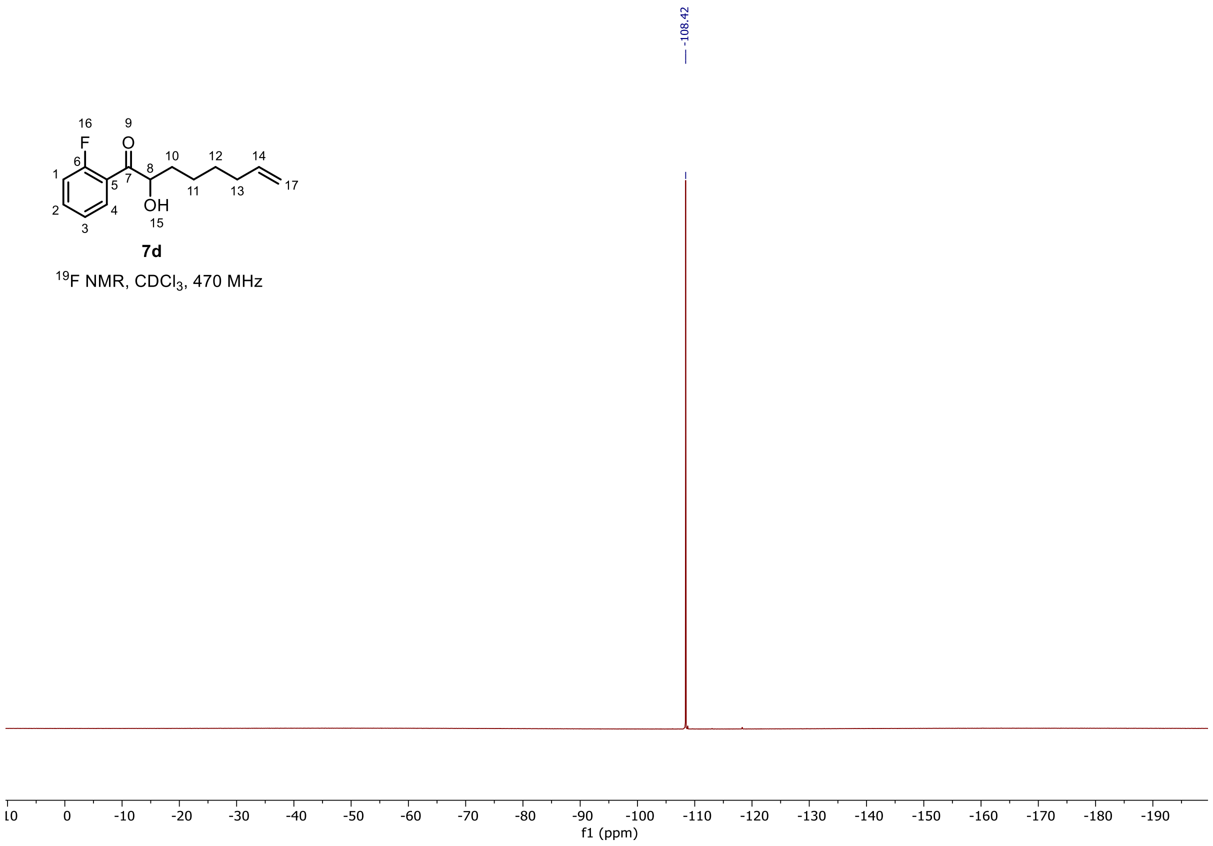

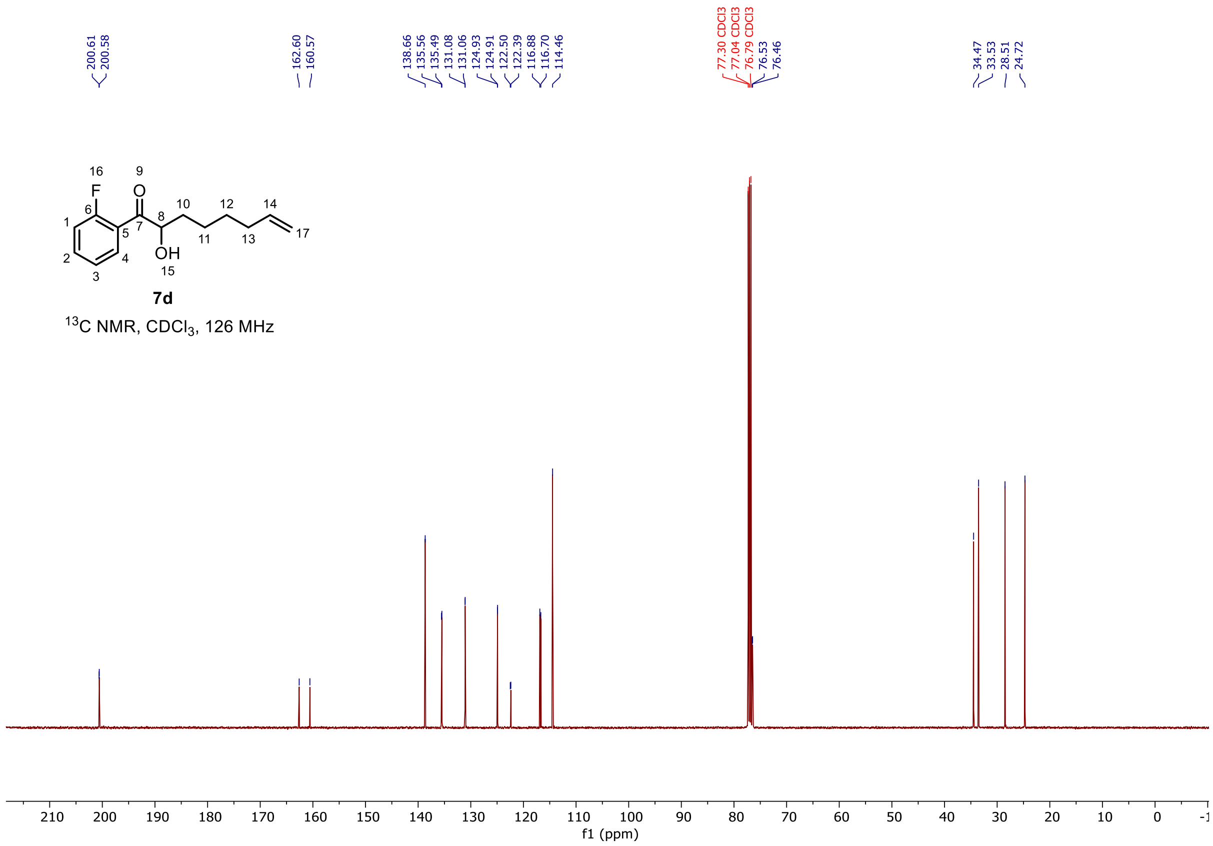
200.61
200.58
162.60
160.57
138.66
135.56
135.49
131.08
131.06
124.93
124.91
122.50
122.39
116.88
116.70
114.46
77.30 CDCl3
77.04 CDCl3
76.79 CDCl3
76.53
76.46
34.47
33.53
28.51
24.72
16
9
F
O
6
7
8
10
12
14
1
5
11
13
17
2
4
OH
15
3
7d
13C NMR, CDCl3, 126 MHz
f1 (ppm)

2-Hydroxy-1-(3-methoxyphenyl)oct-7-en-1-one 7e

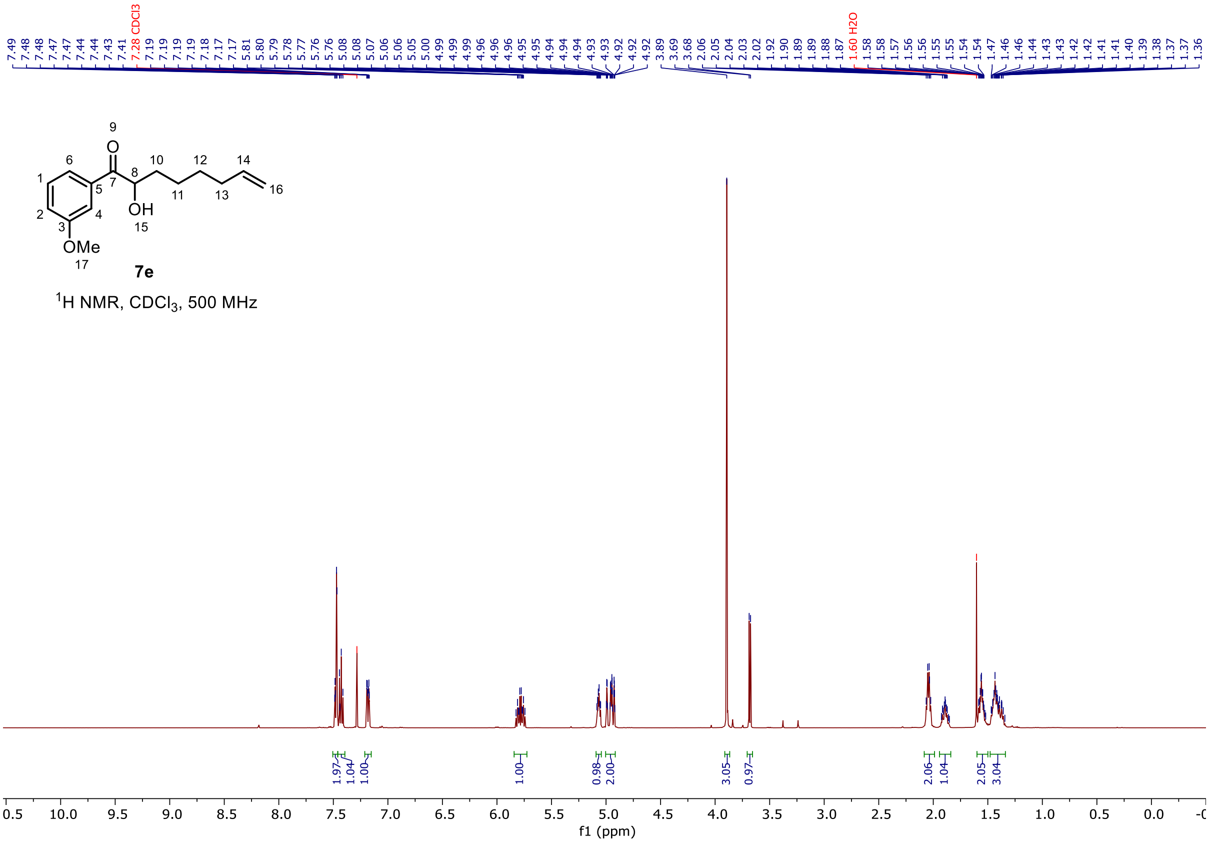

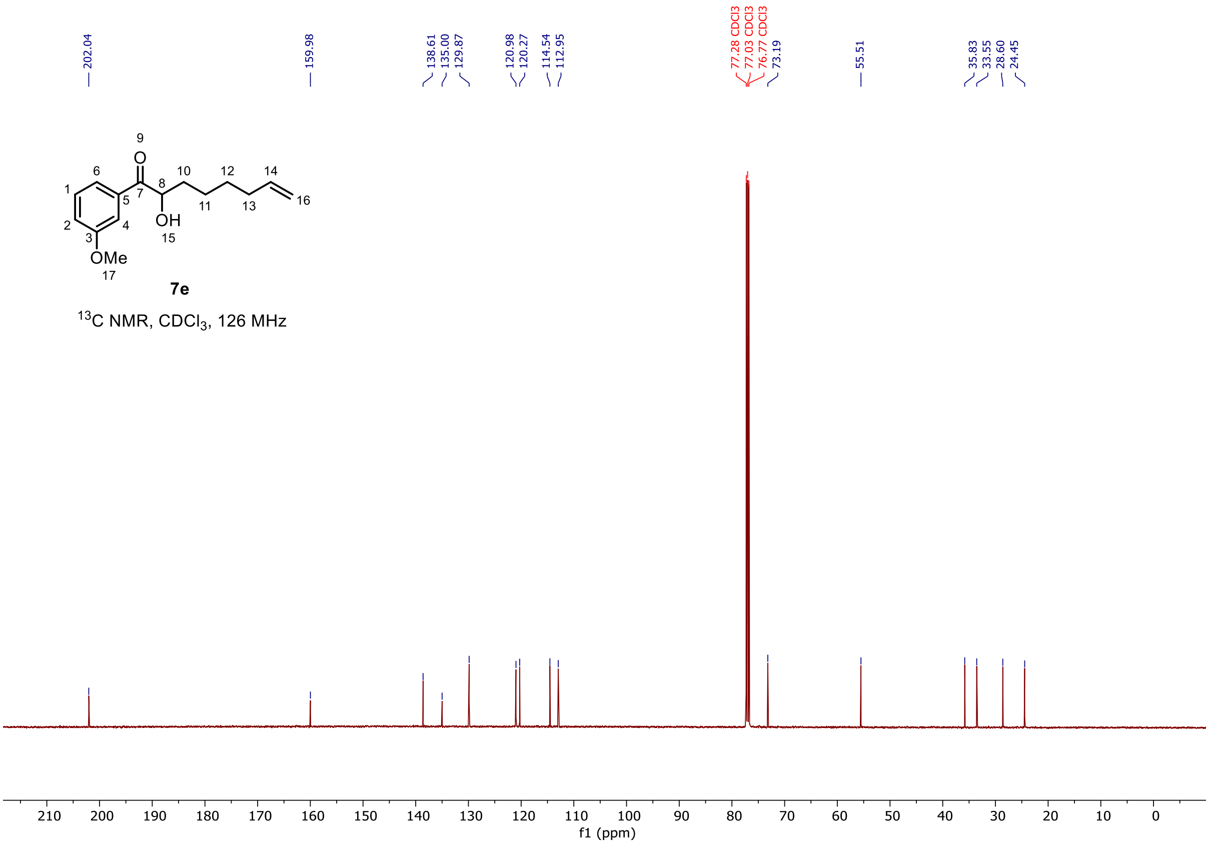

2-Hydroxy-1-(3-(trifluoromethyl)phenyl)oct-7-en-1-one 7f

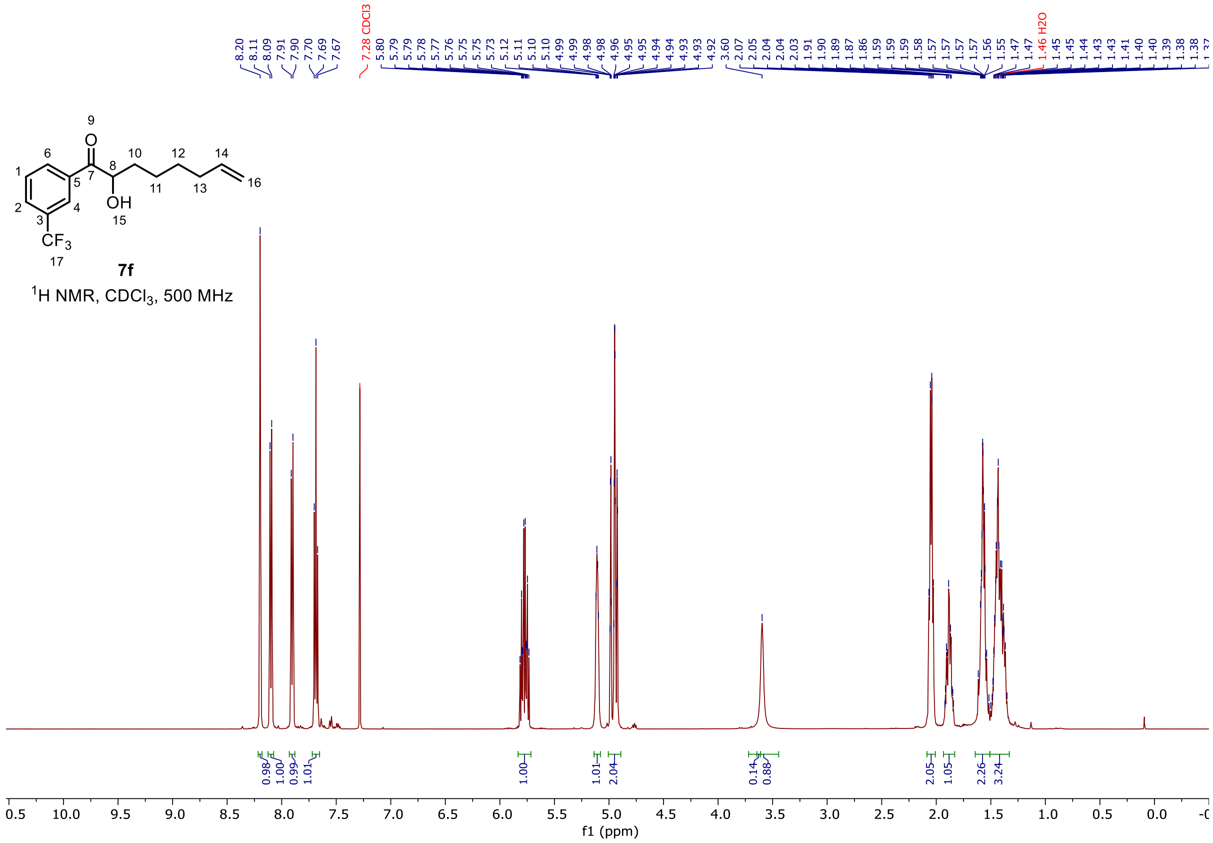

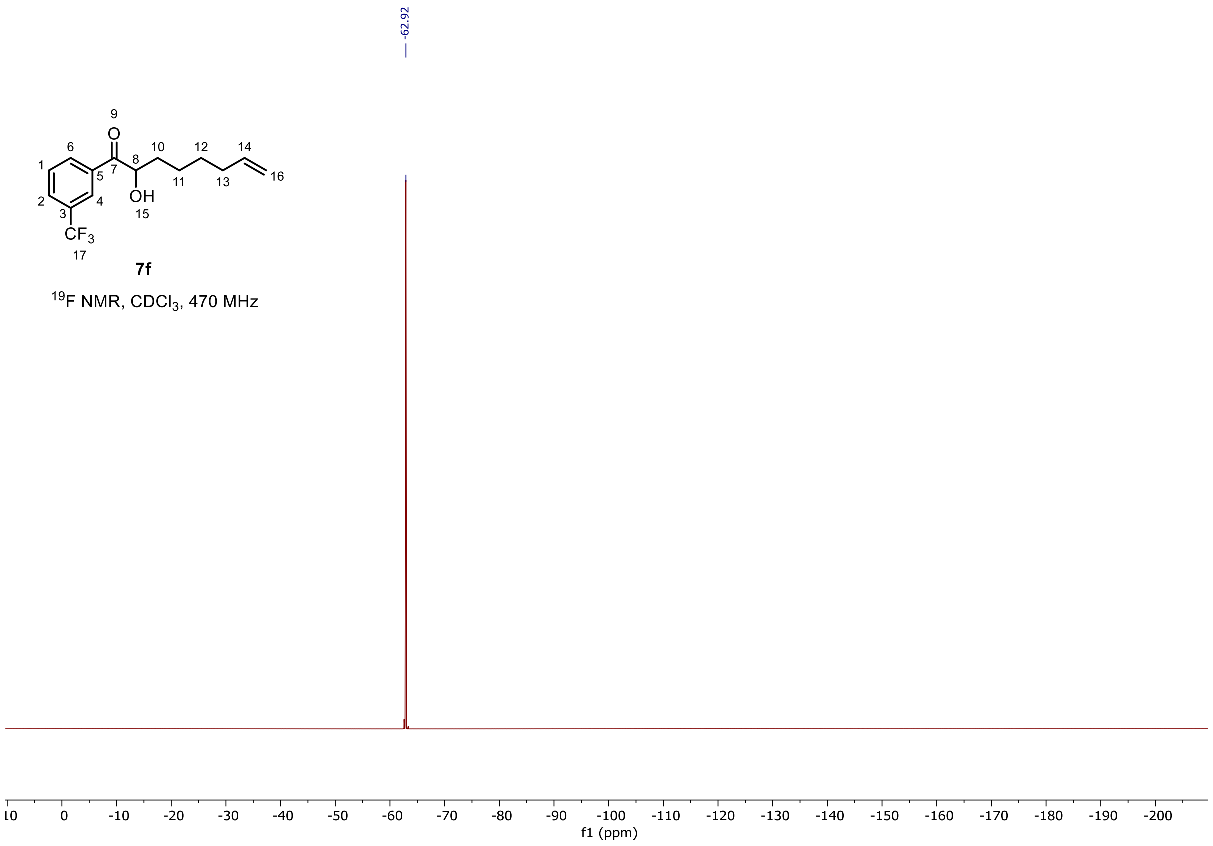

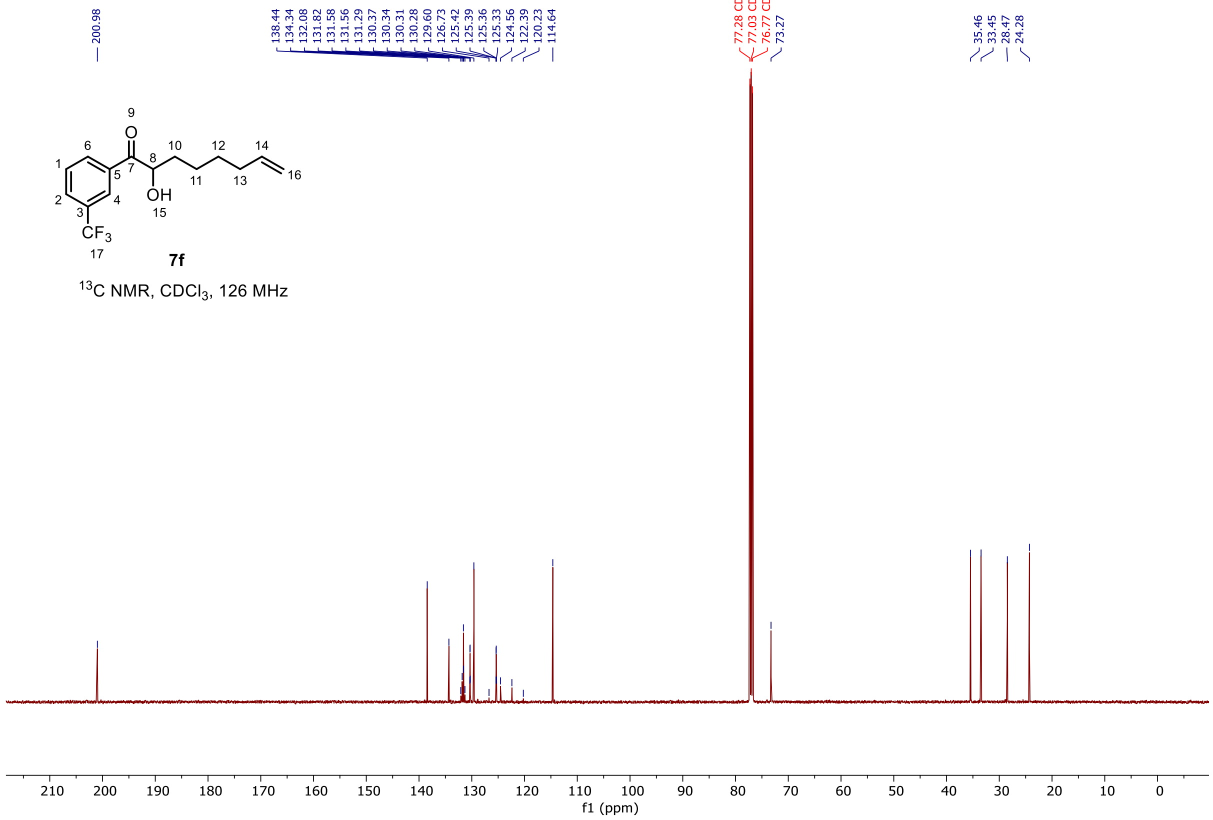
200.98
138.44
134.34
132.08
131.82
131.58
131.56
131.29
130.37
130.34
130.31
130.28
129.60
126.73
125.42
125.39
125.36
125.33
124.56
122.39
120.23
114.64
77.28 CDCl3
77.03 CDCl3
76.77 CDCl3
73.27
35.46
33.45
28.47
24.28
9
6
1
5
7
8
10
12
14
16
11
13
2
3
4
OH
15
CF3
17
7f
13C NMR, CDCl3, 126 MHz
f1 (ppm)

2-Hydroxy-1-(naphthalen-2-yl)oct-7-en-1-one 7g

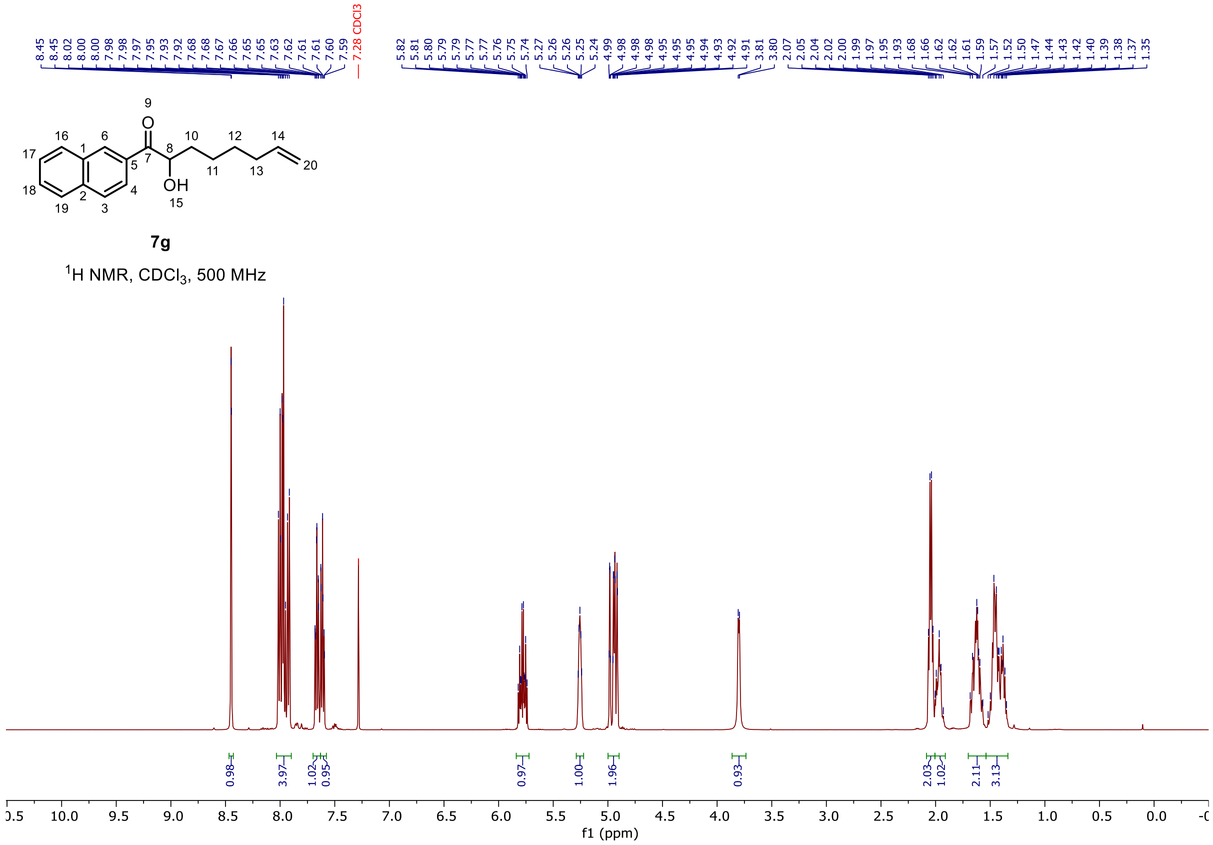

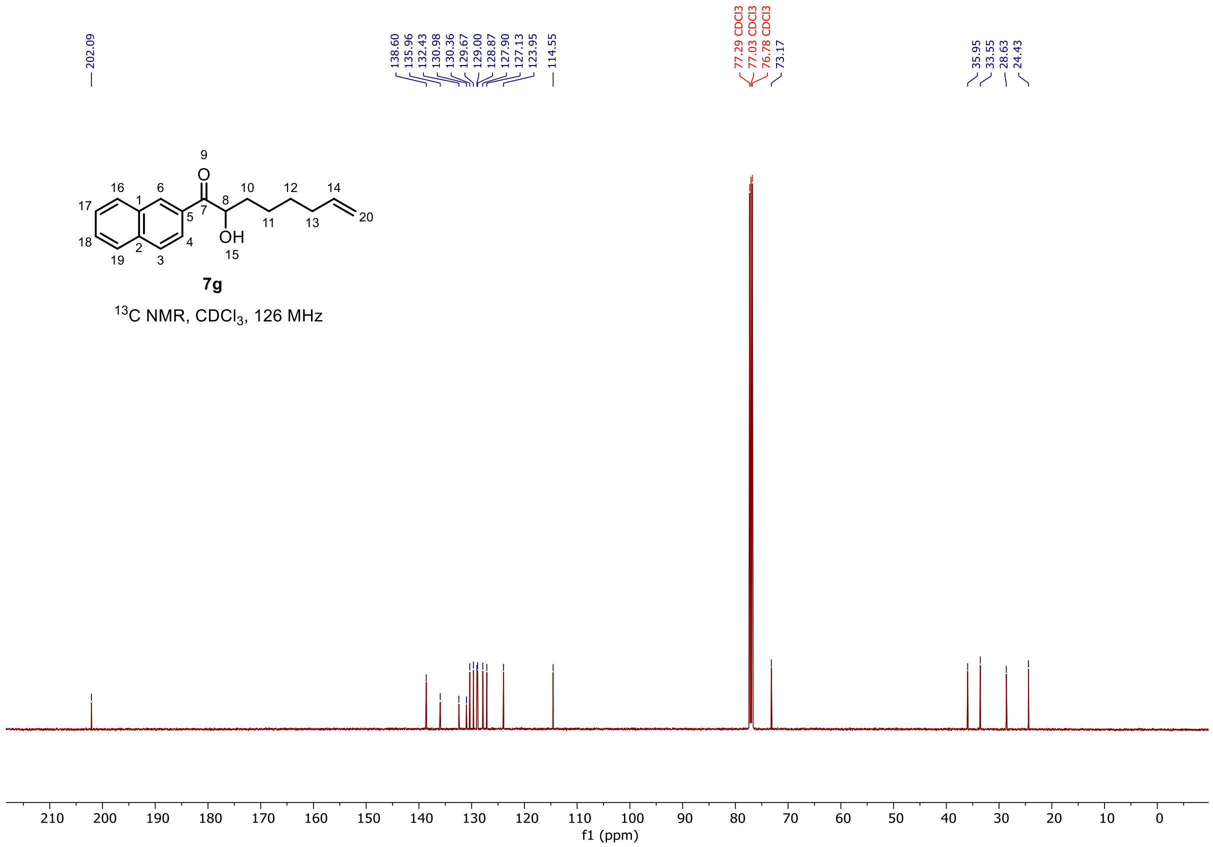

1-(Furan-2-yl)-2-hydroxyoct-7-en-1-one 7h

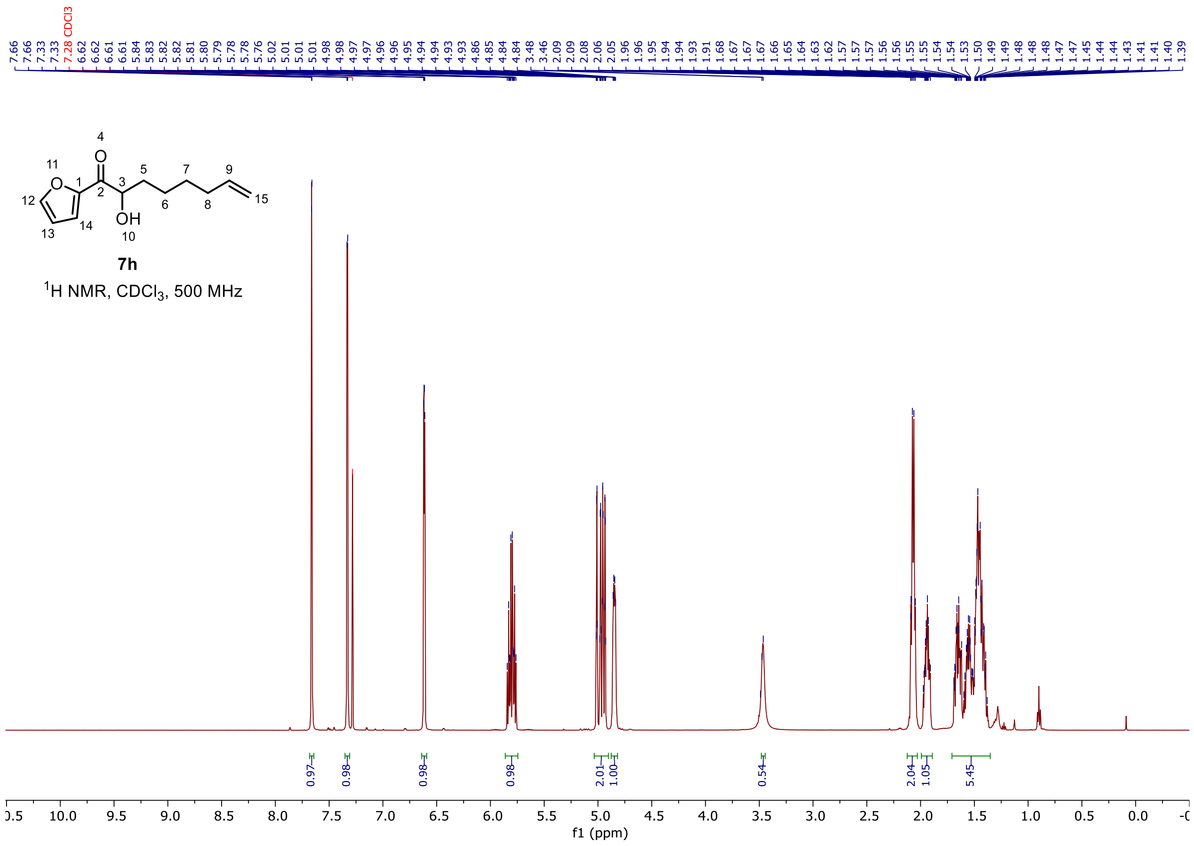

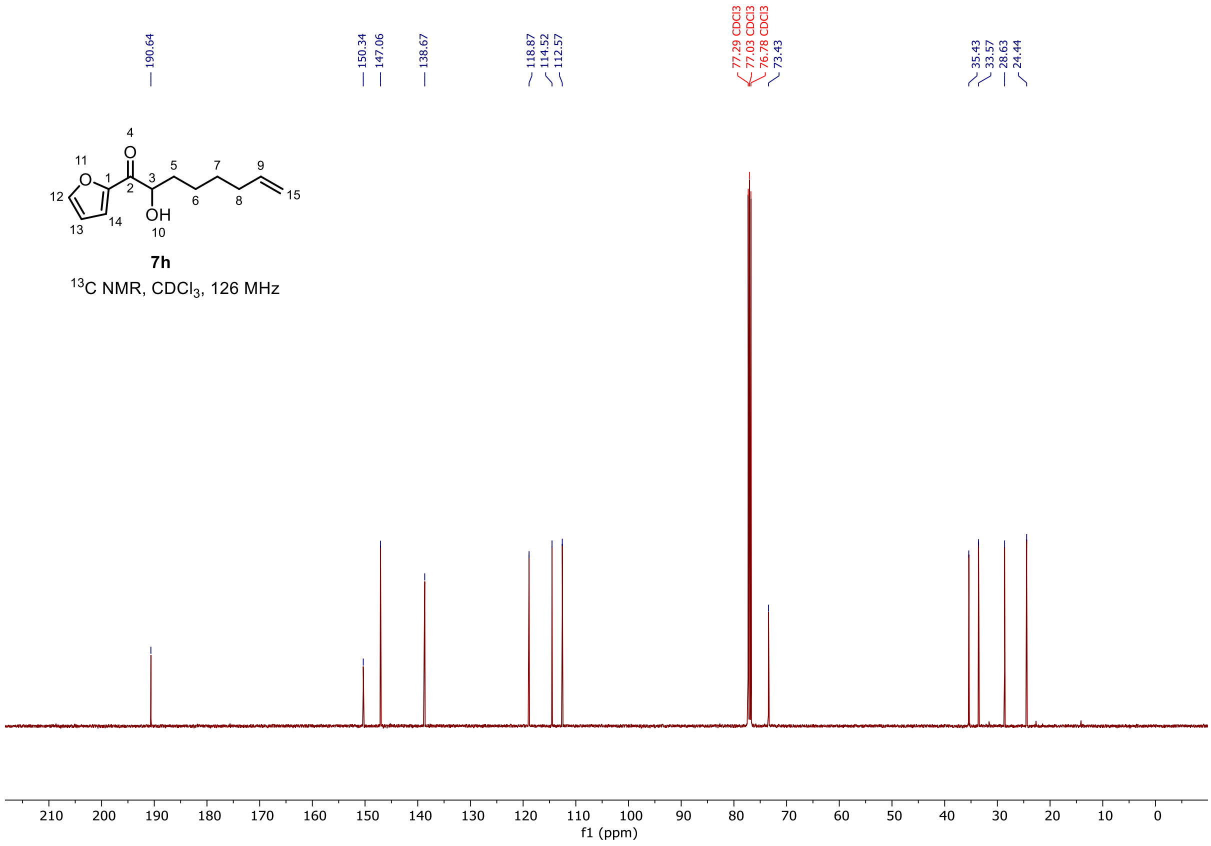

2-Hydroxy-1-(1-methyl-1*H*-indol-2-yl)oct-7-en-1-one 7i

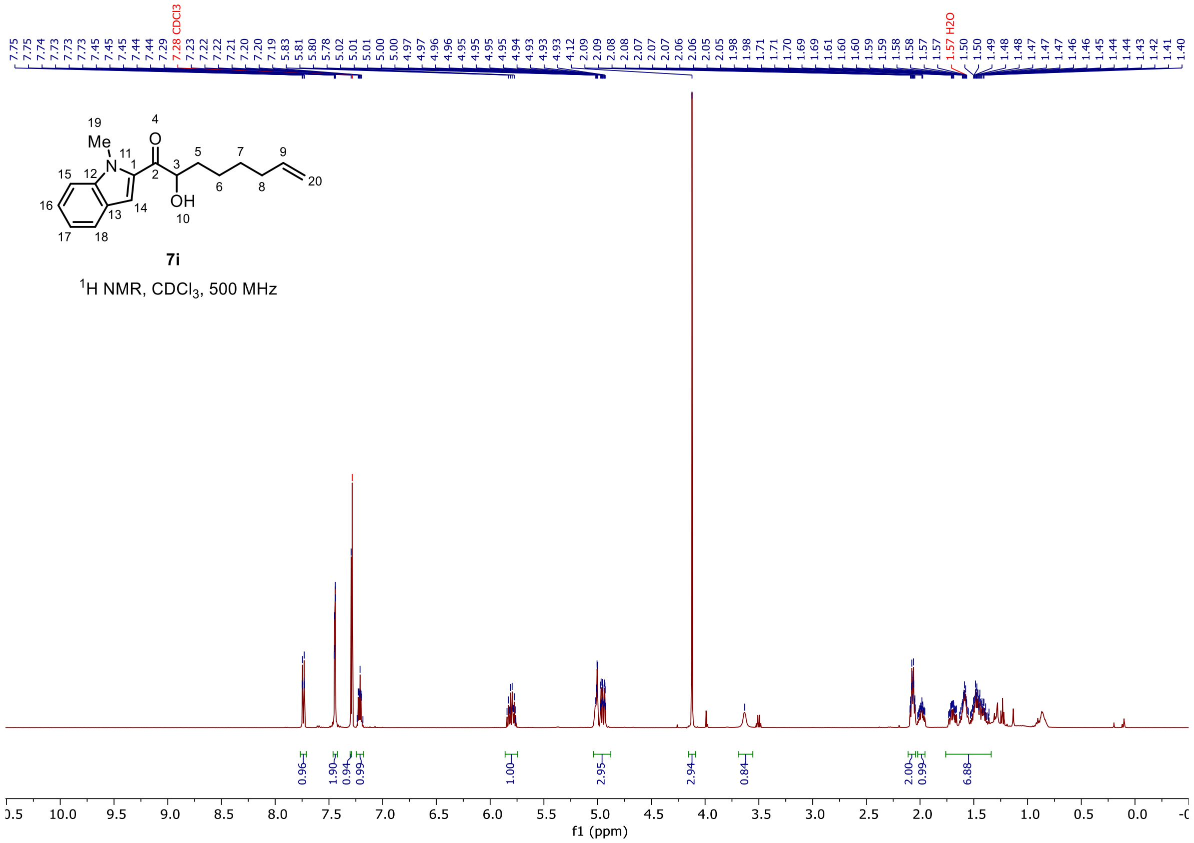

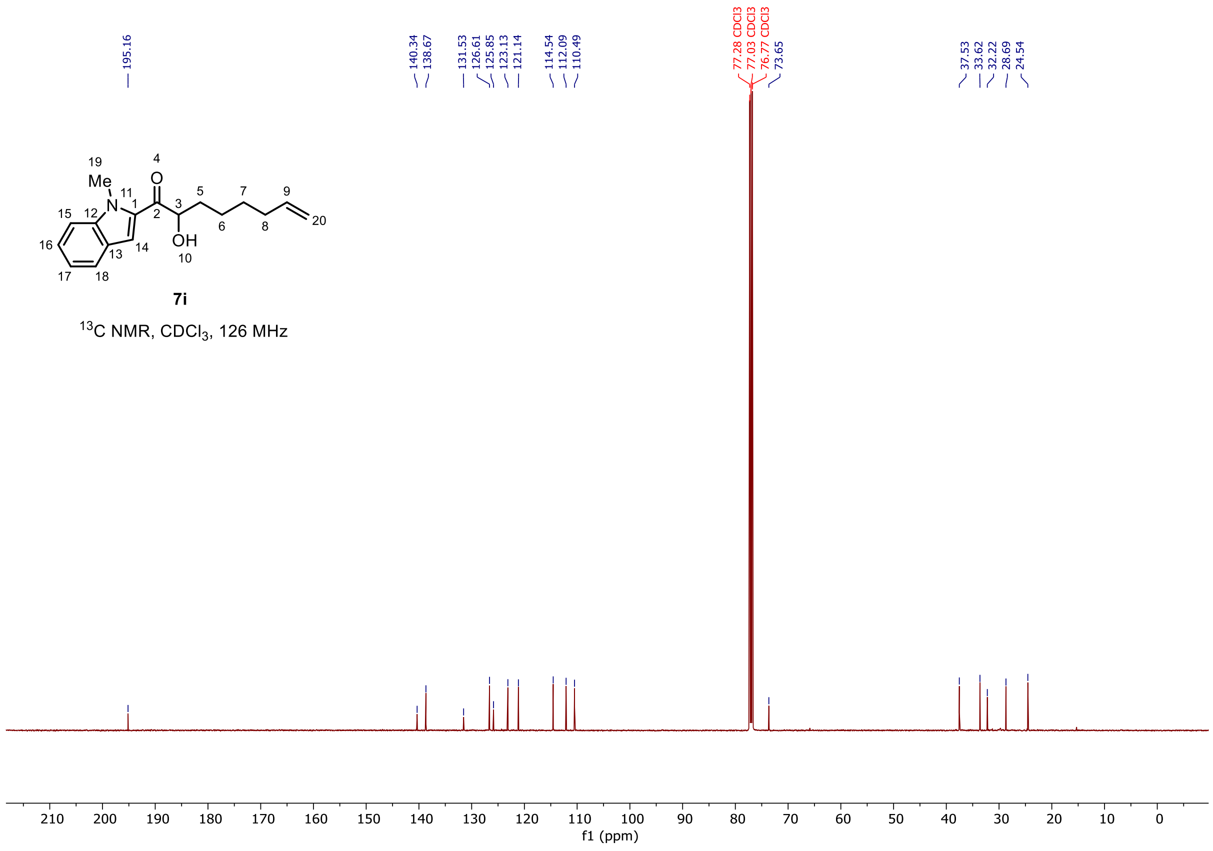

1-(3-(Cyclopropylmethoxy)-4-(difluoromethoxy)phenyl)-2-hydroxyoct-7-en-1-one 7j

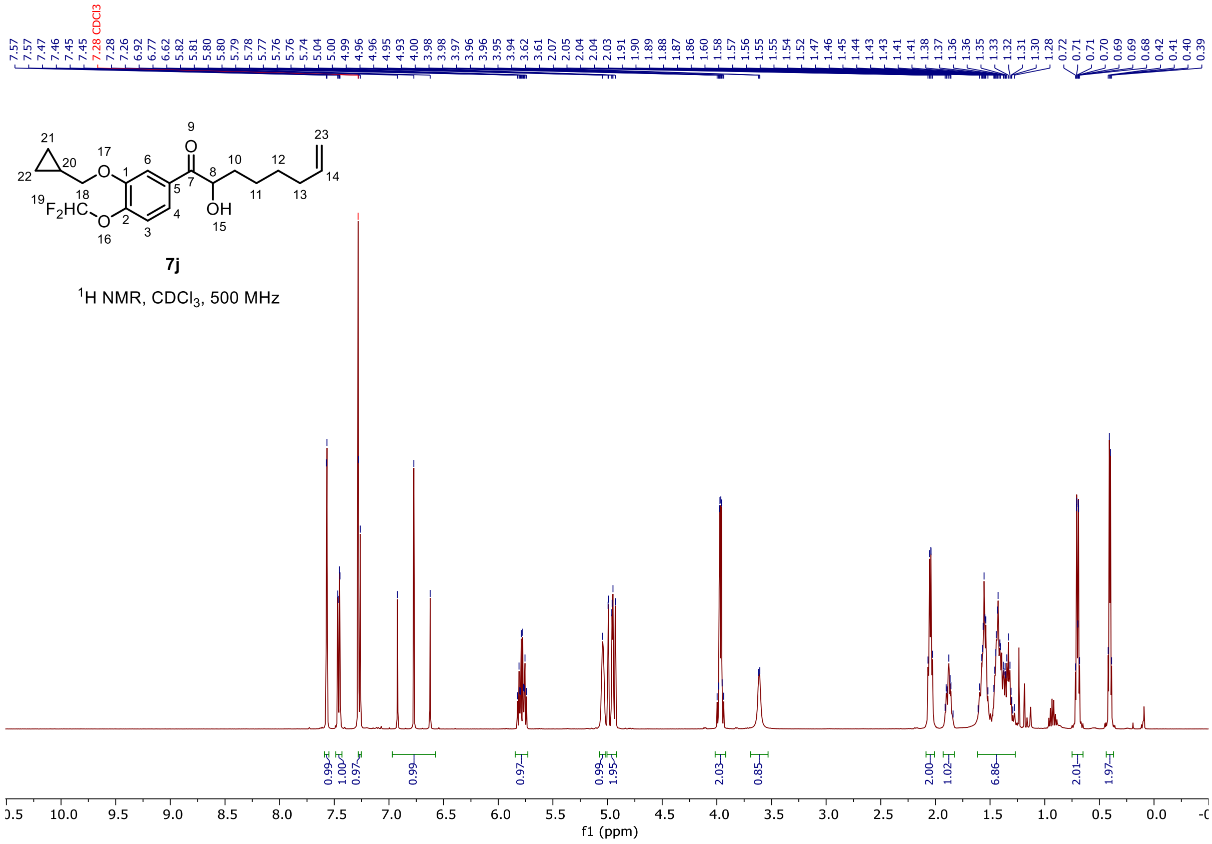

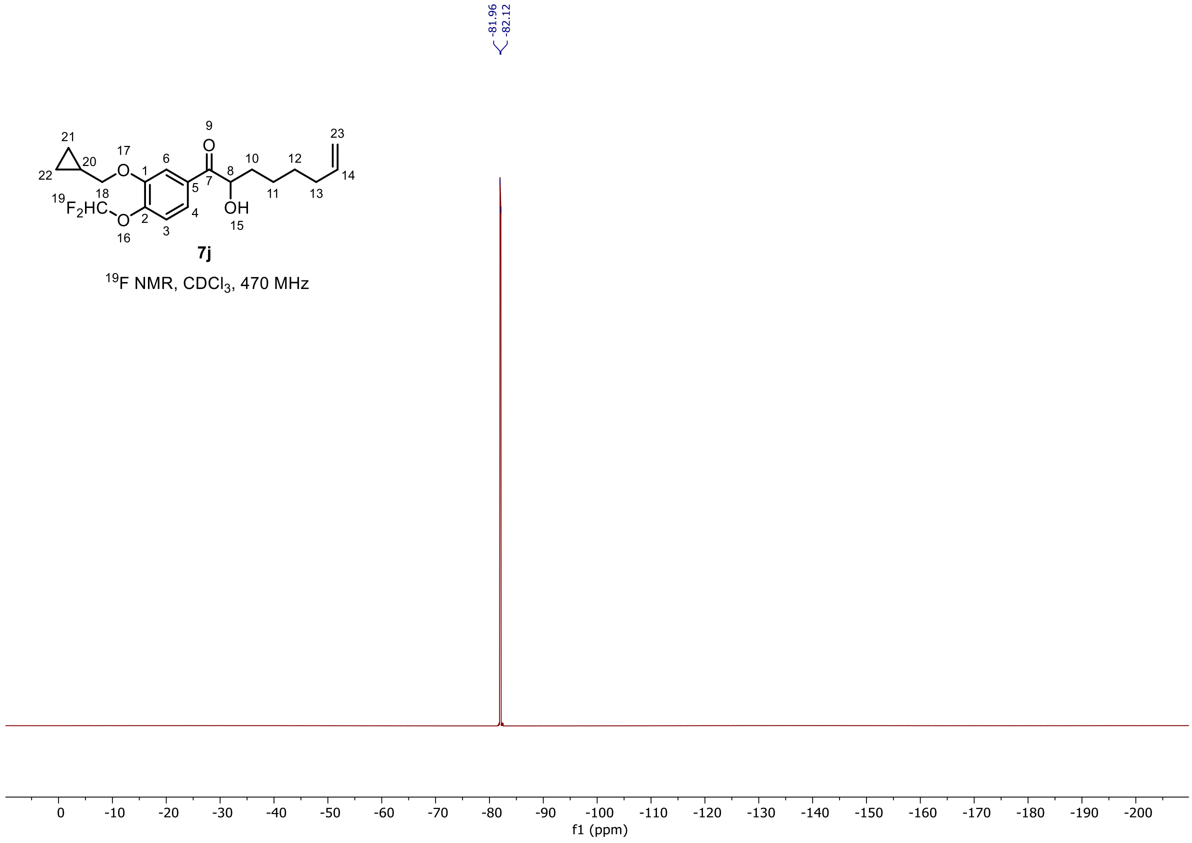

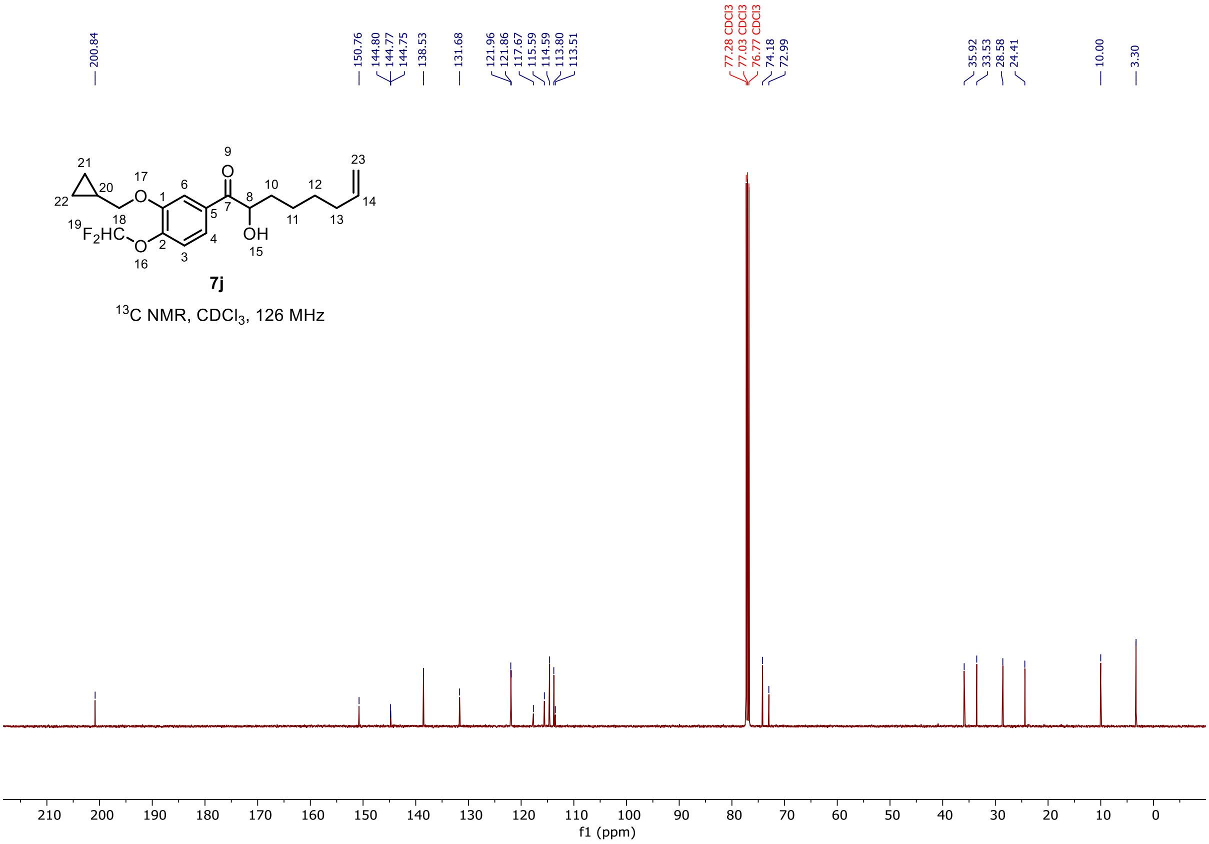
200.84
150.76
144.80
144.77
144.75
138.53
131.68
121.96
121.86
117.67
115.59
114.59
113.80
113.51
77.28 CDCl3
77.03 CDCl3
76.77 CDCl3
74.18
72.99
35.92
33.53
28.58
24.41
10.00
3.30
7j
13C NMR, CDCl3, 126 MHz
f1 (ppm)

3-Hydroxyoct-7-en-2-one 5l

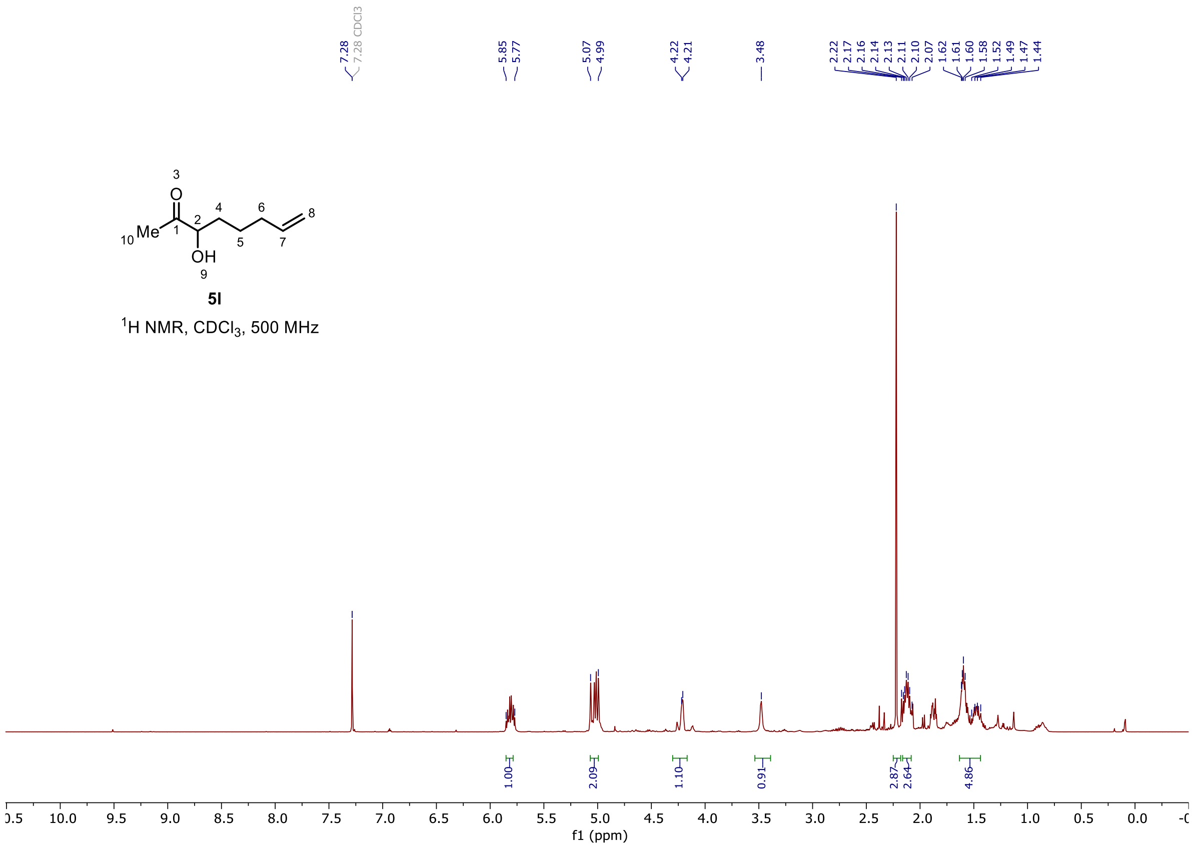

3-Hydroxynon-8-en-2-one 7l

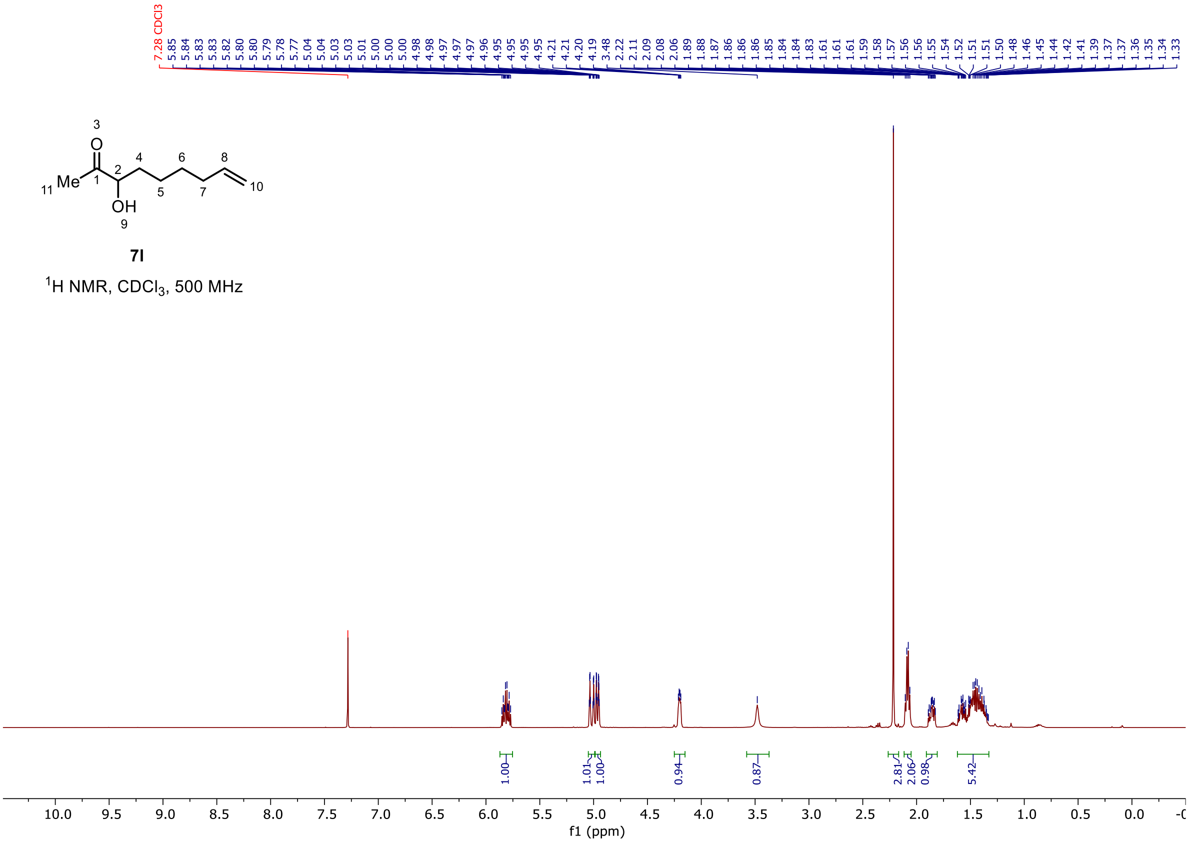

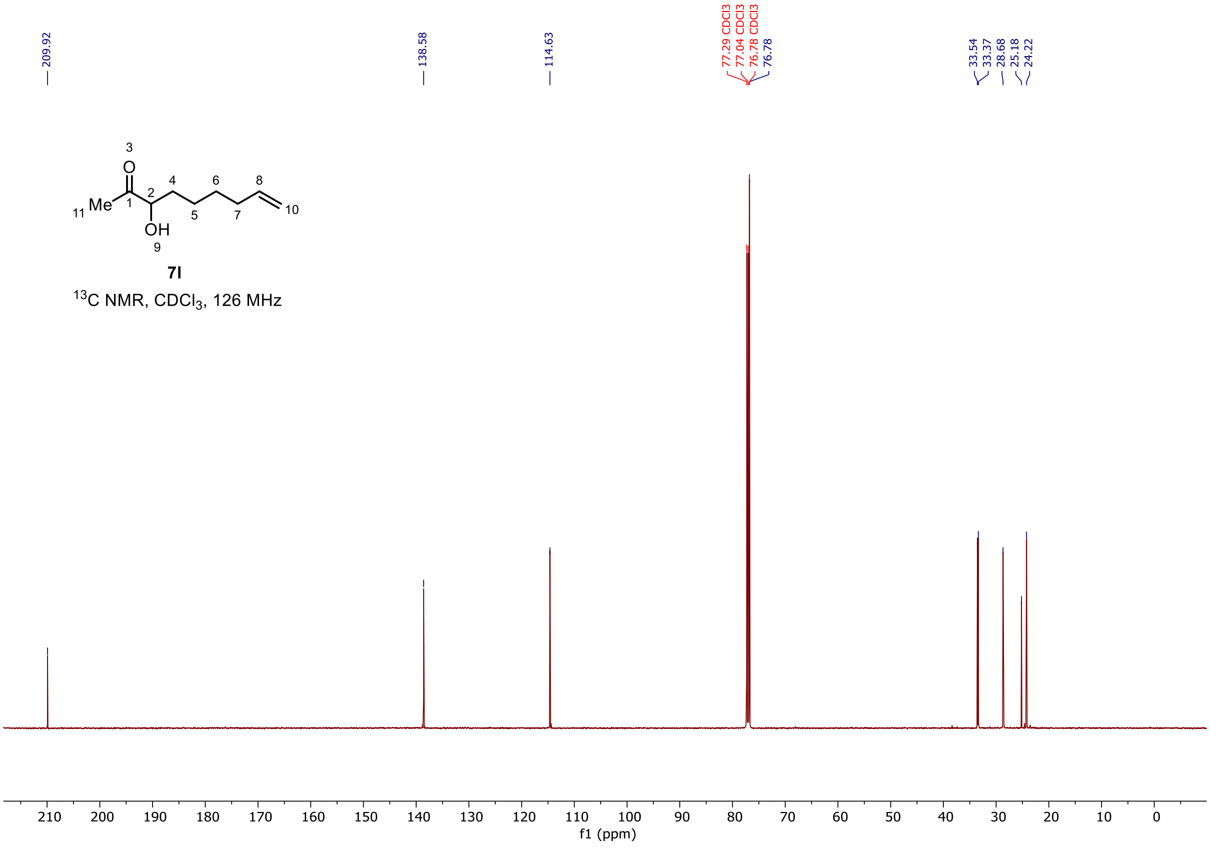

2-Hydroxy-1-phenylhex-5-en-1-one 9

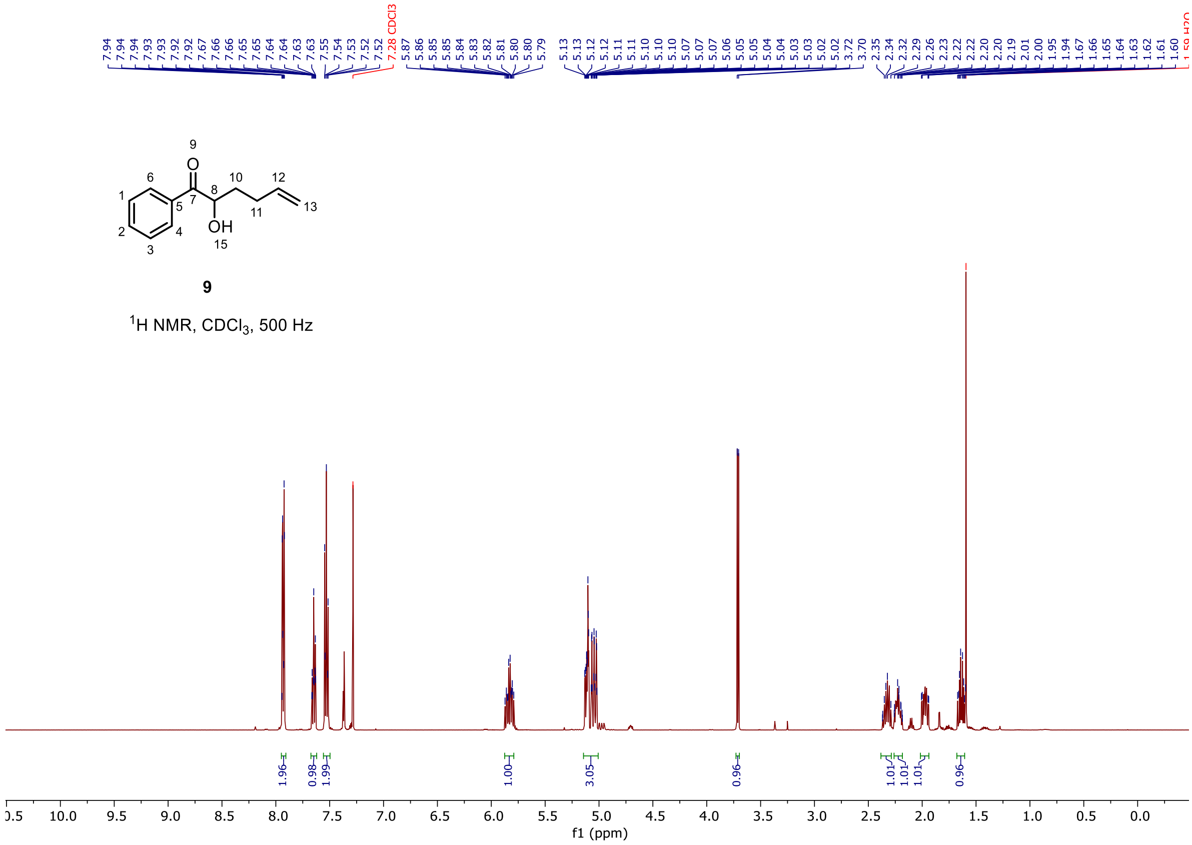

2-Hydroxy-1-phenylnon-8-en-1-one 10

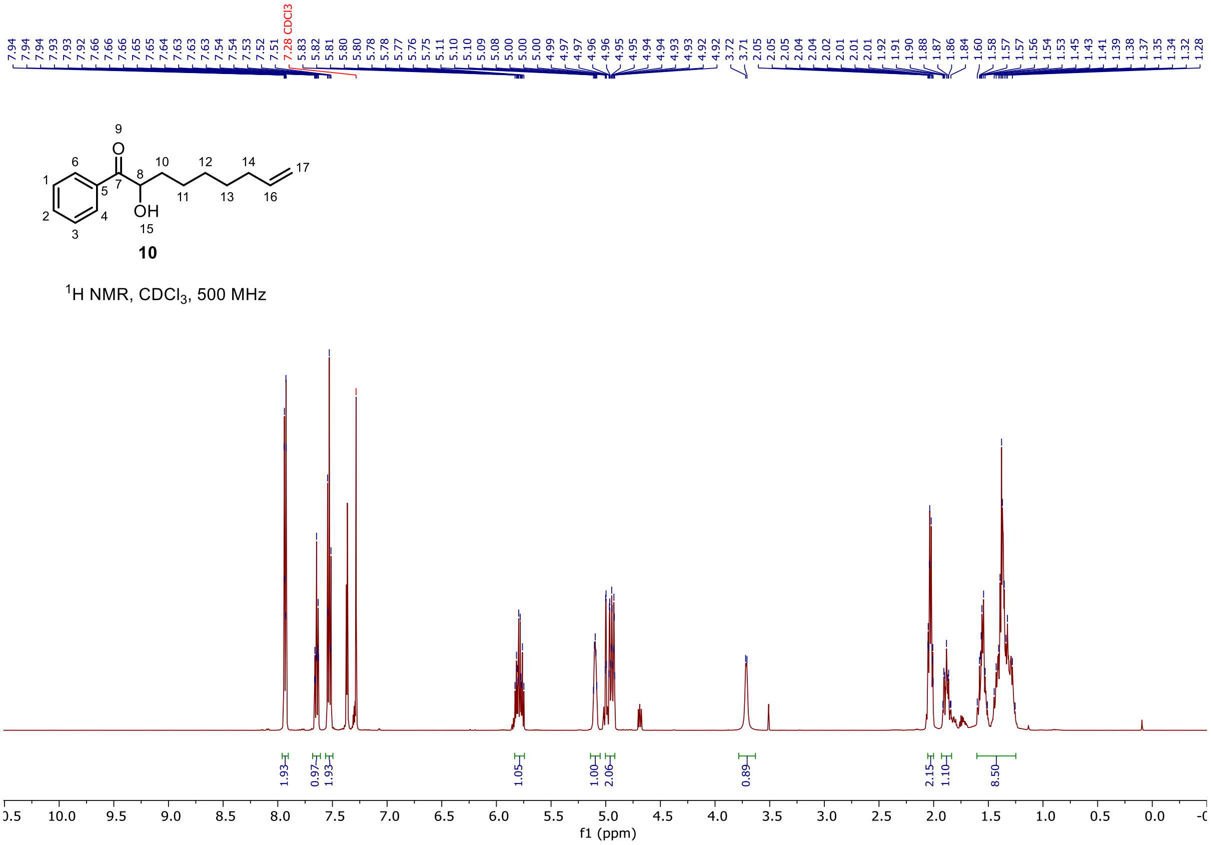

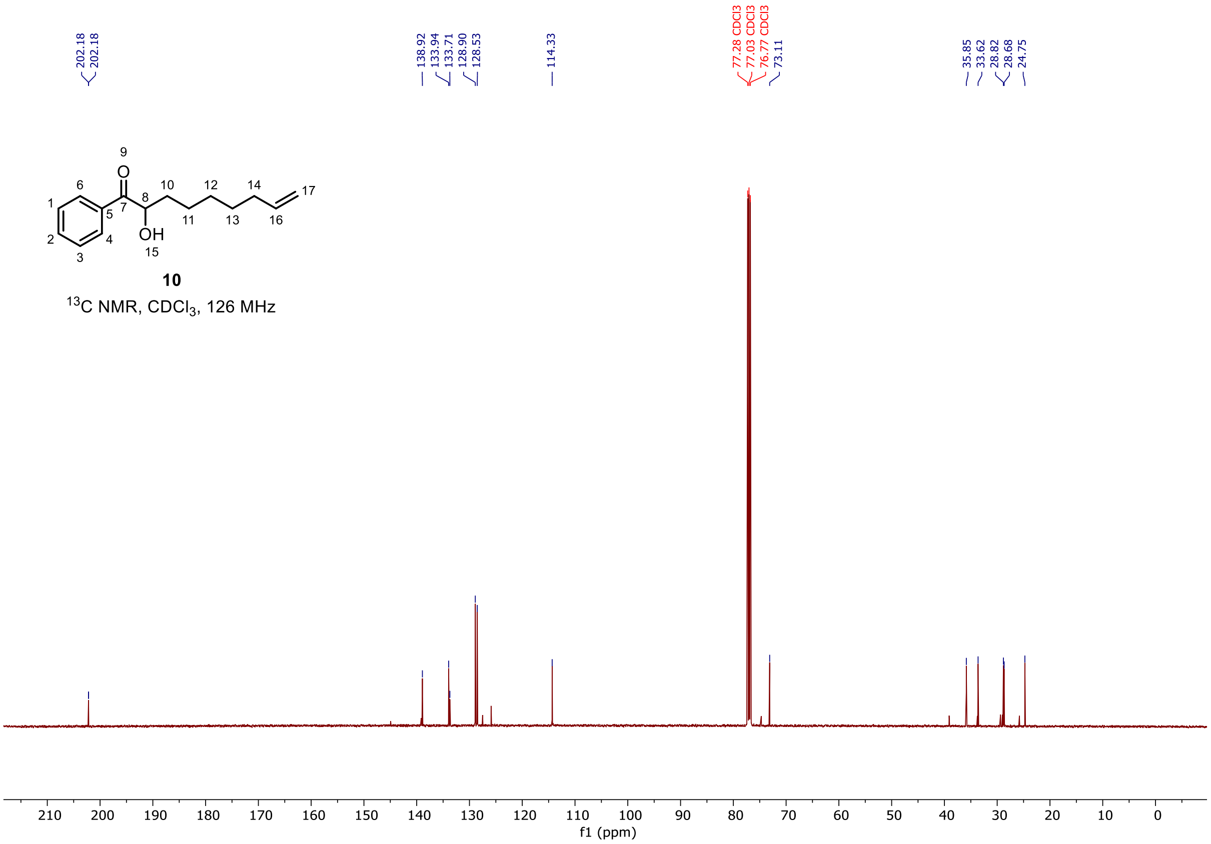

1-Phenyl-4-(2-vinylphenyl)butan-1-one 7m-s10

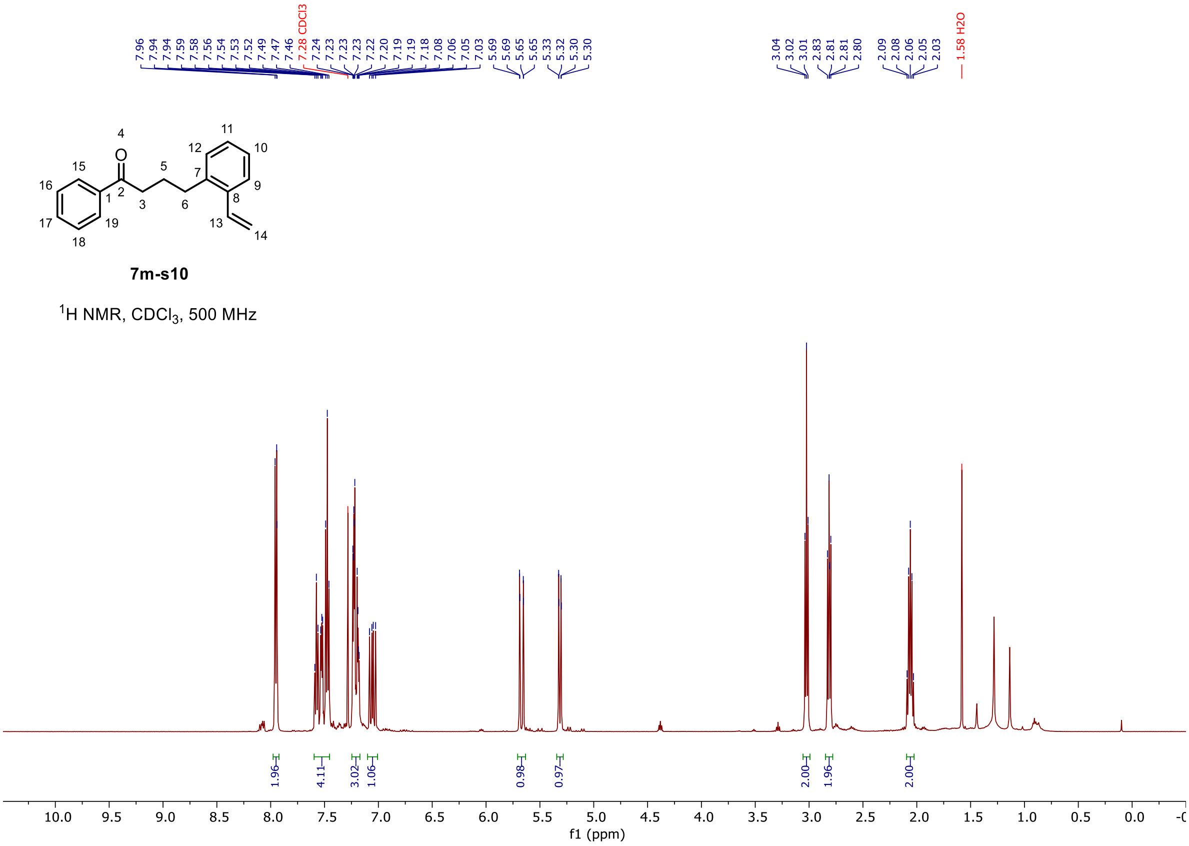

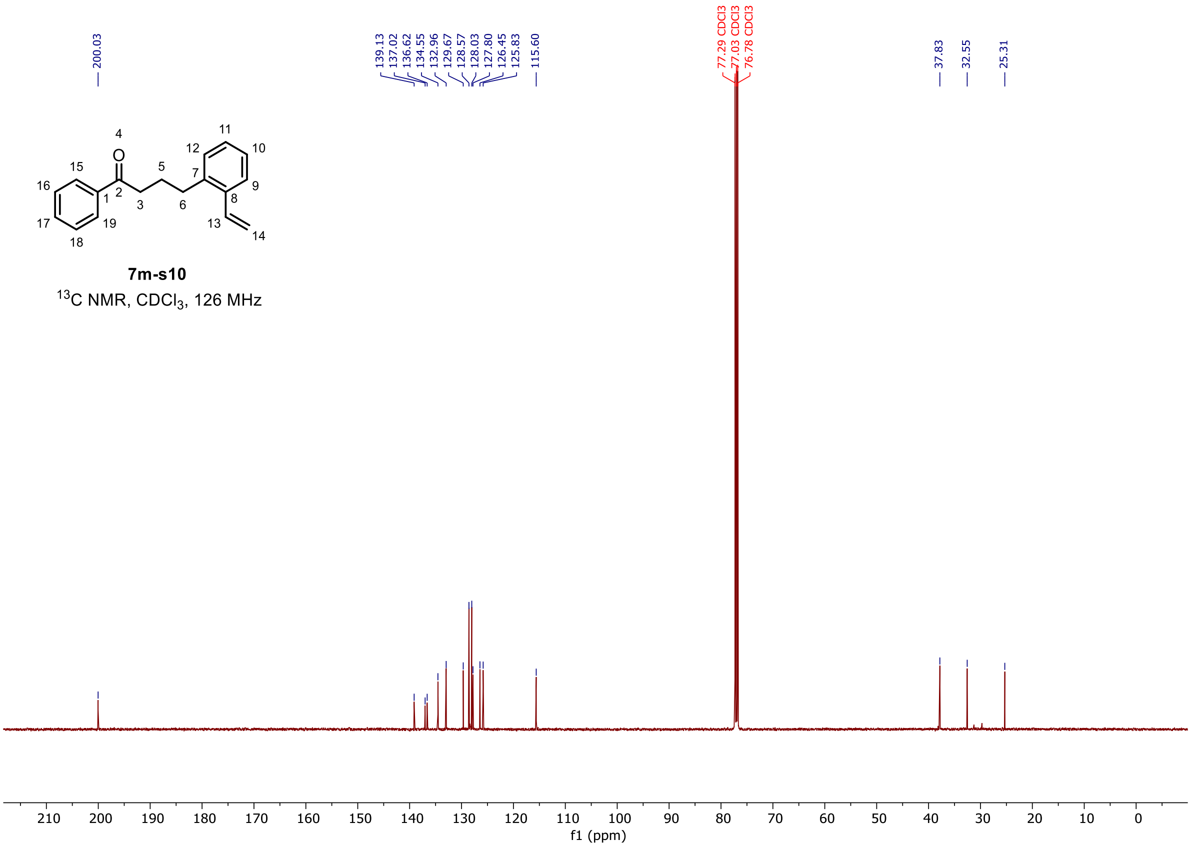

2-Hydroxy-1-phenyl-4-(2-vinylphenyl)butan-1-one 7m

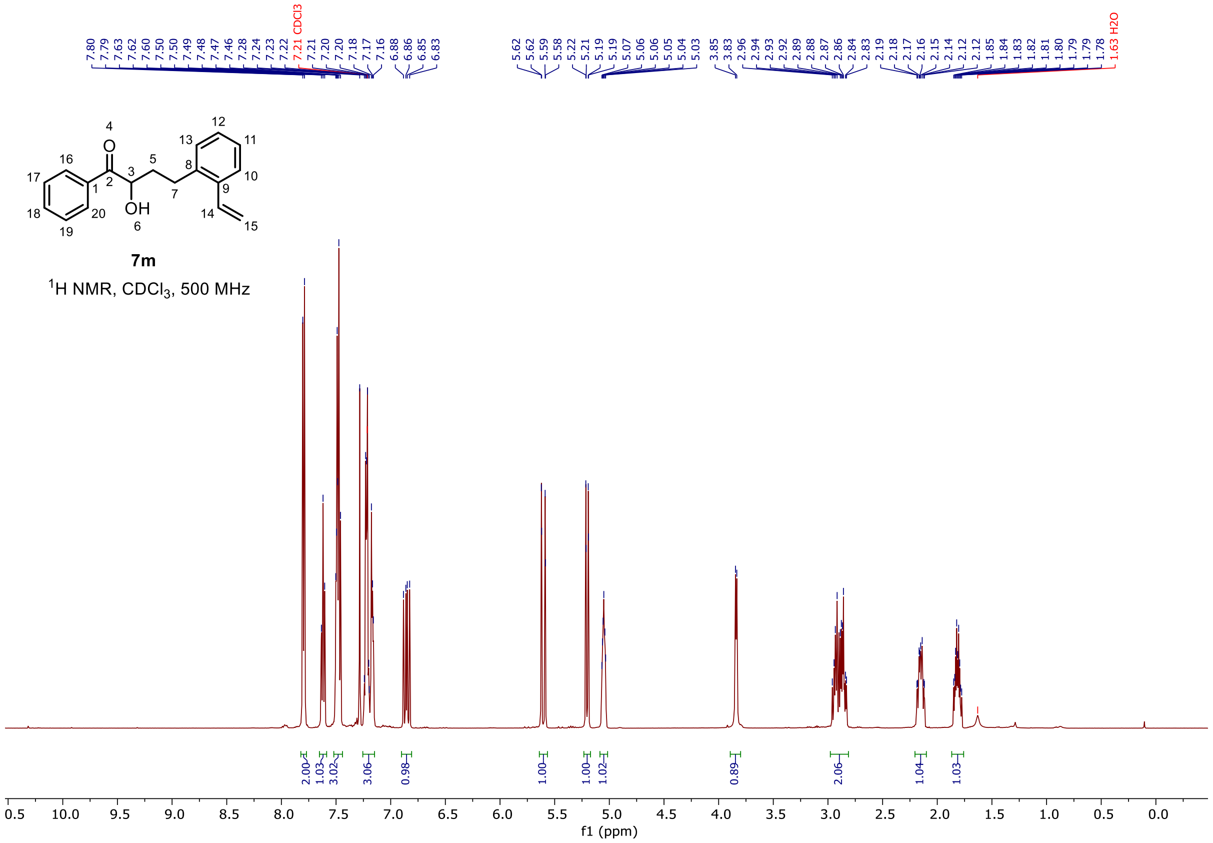

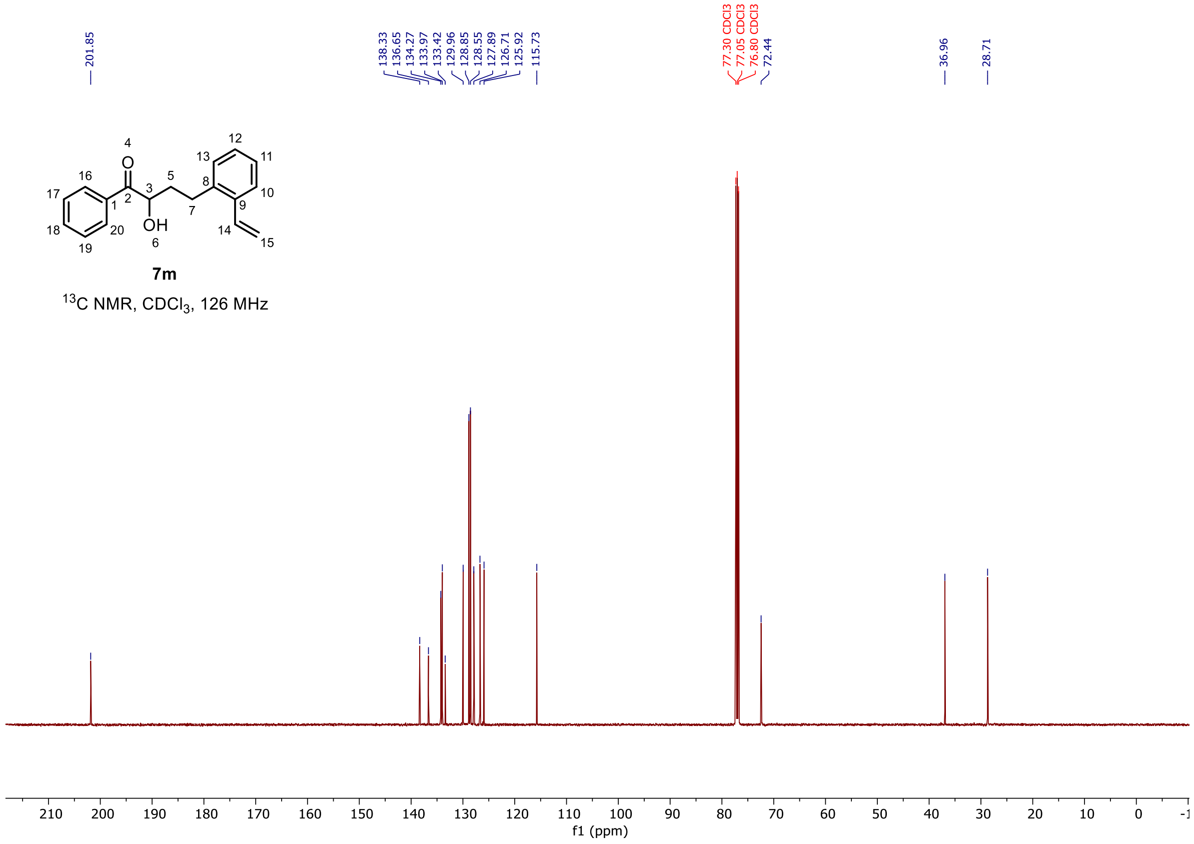

1-(*p*-Tolyl)-4-(2-vinylphenyl)butan-1-one 7n-s10

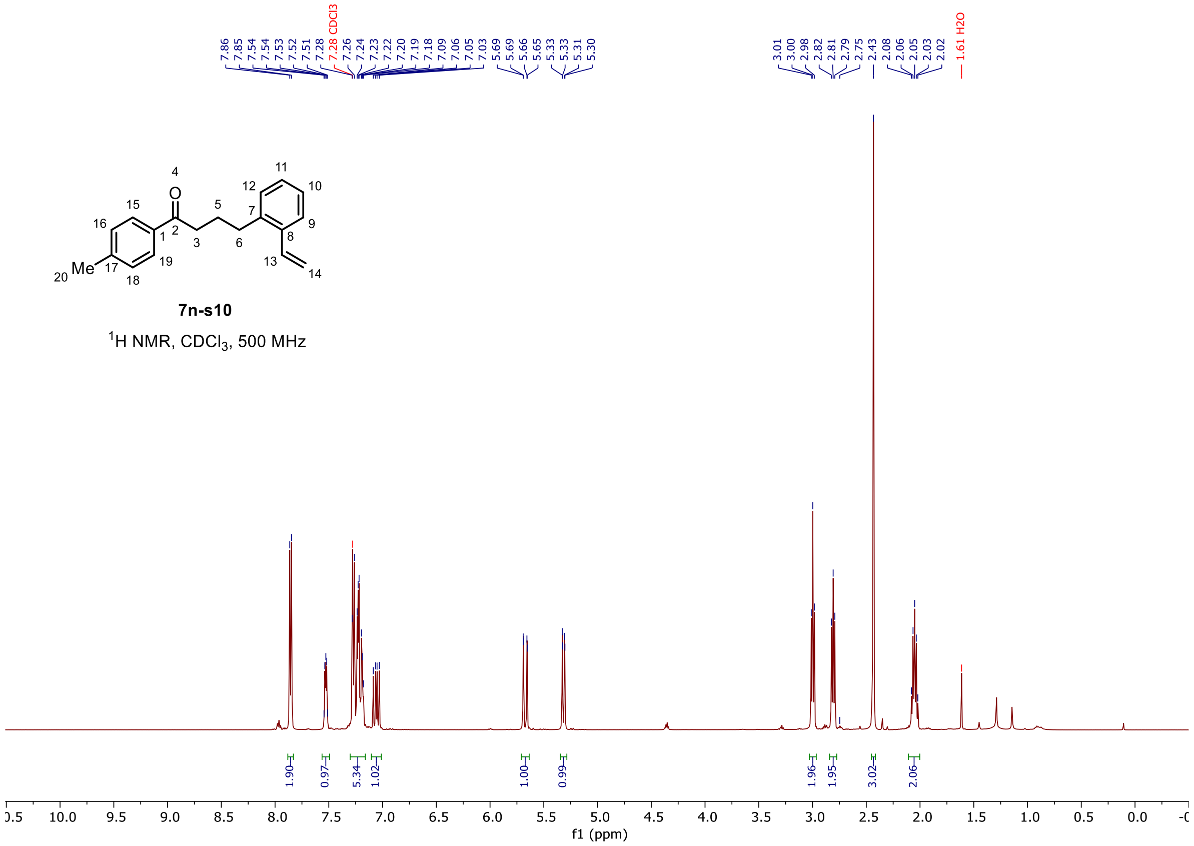

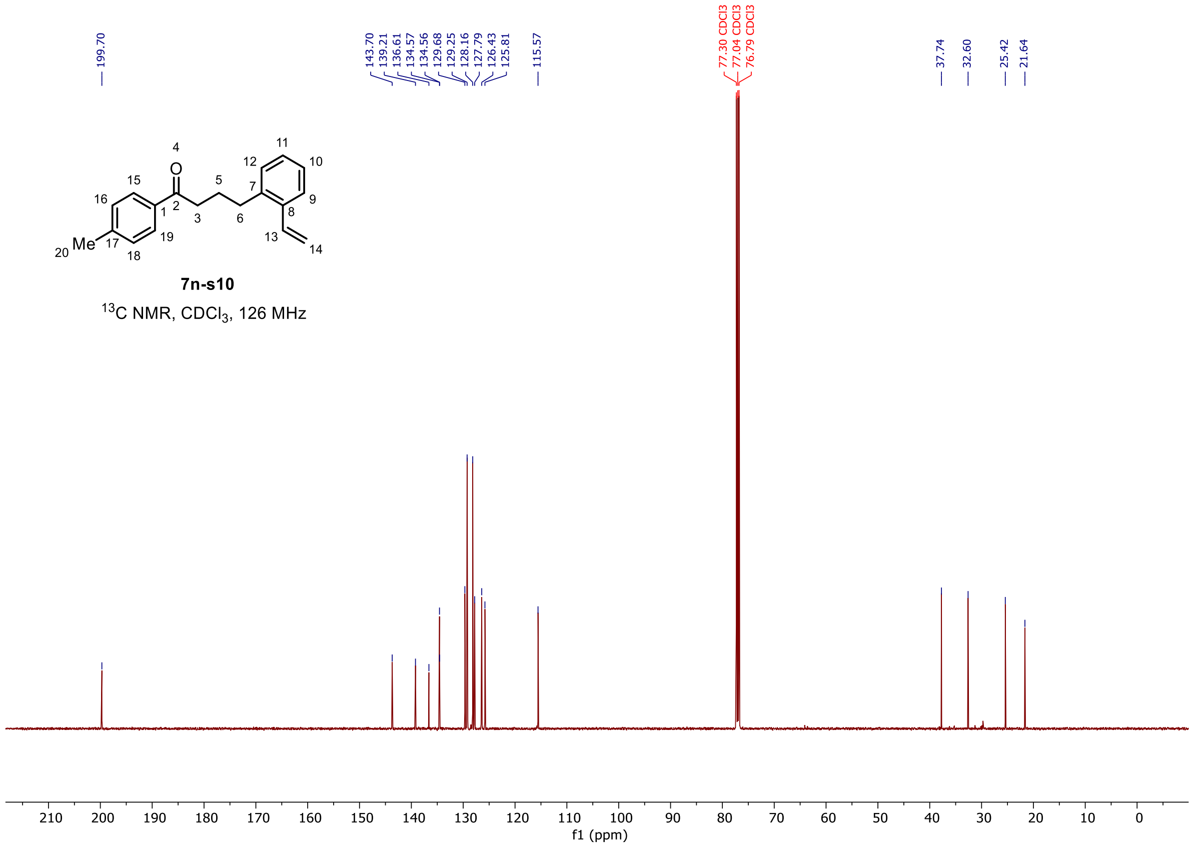

2-Hydroxy-1-(p-tolyl)-4-(2-vinylphenyl)butan-1-one 7n

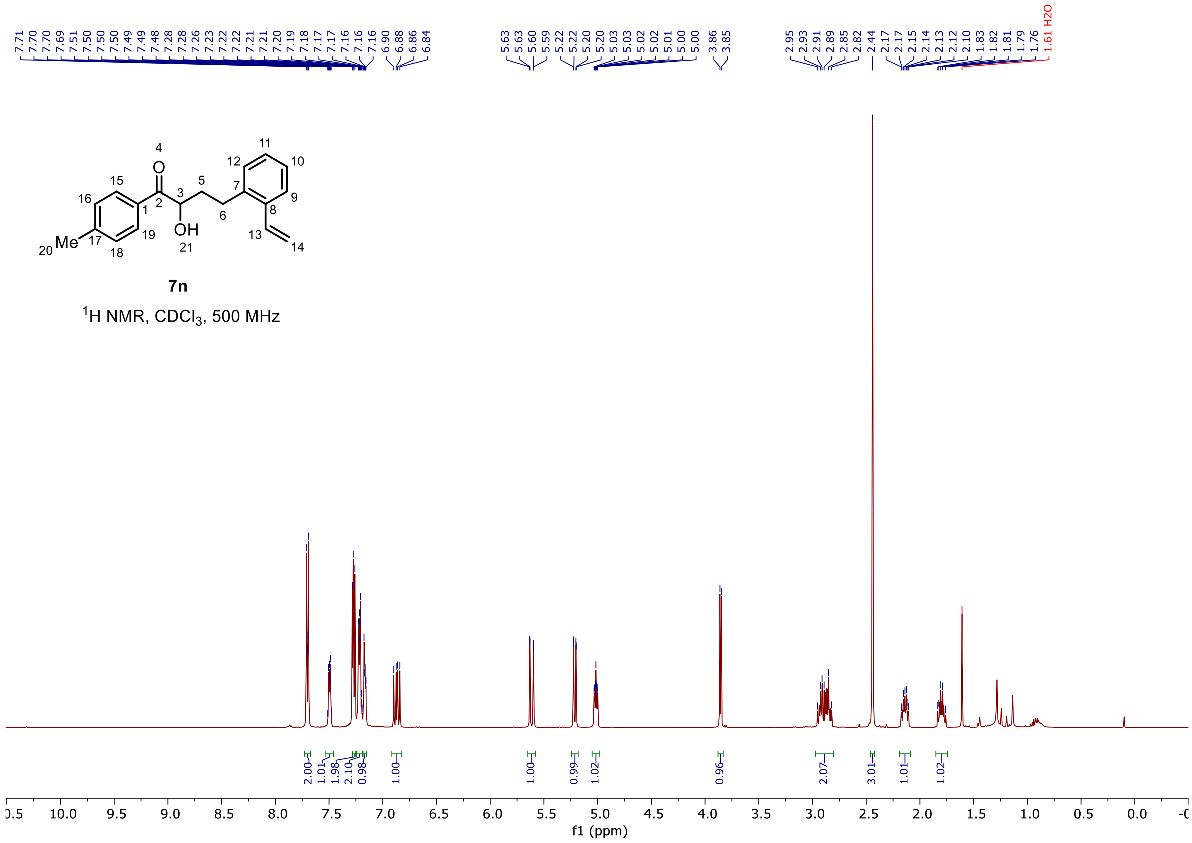

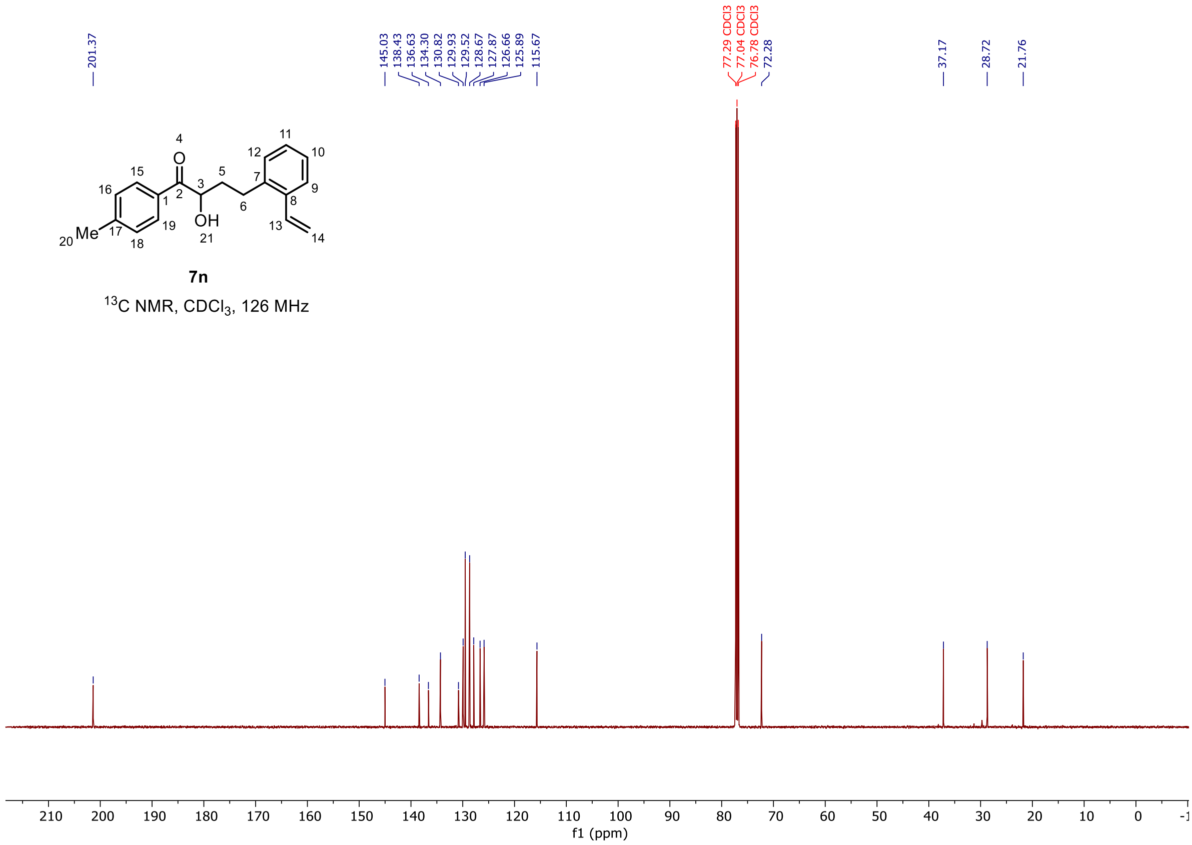

1-(4-Bromophenyl)-4-(2-vinylphenyl)butan-1-one 7o-s10

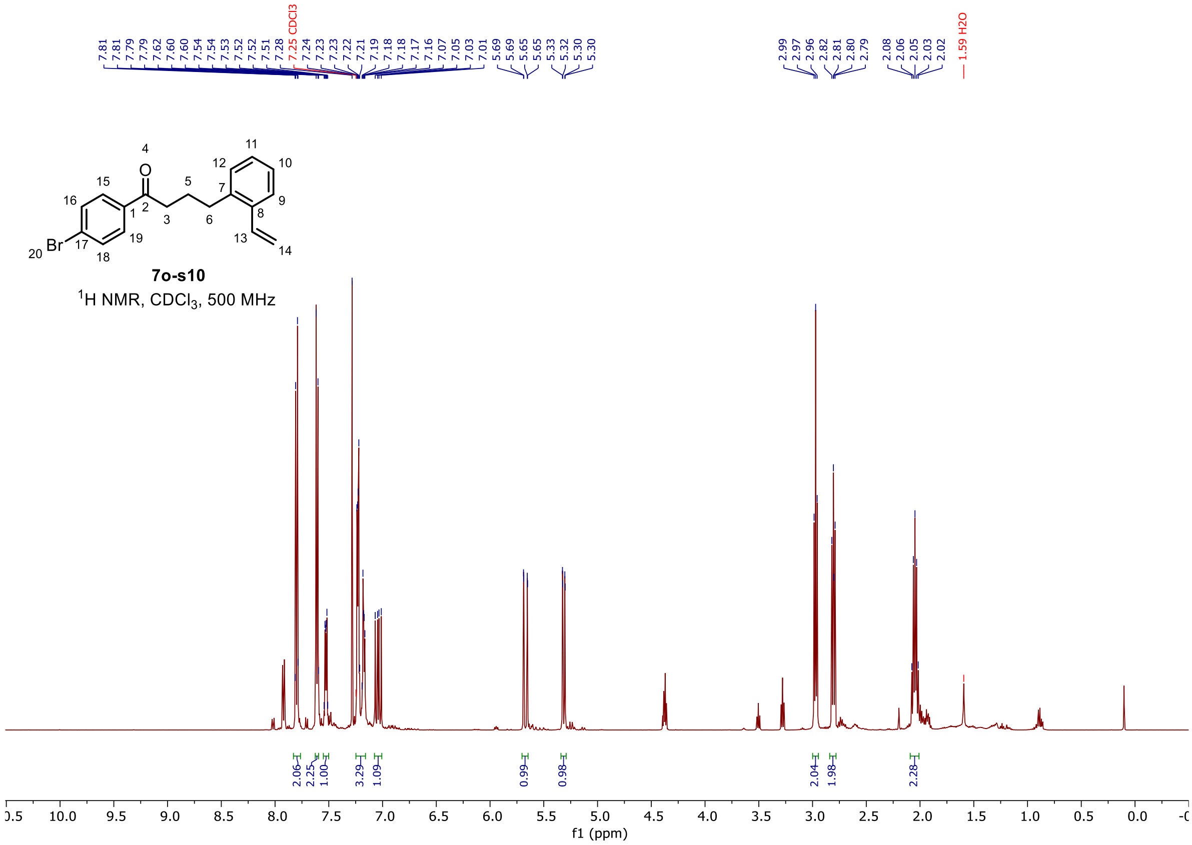

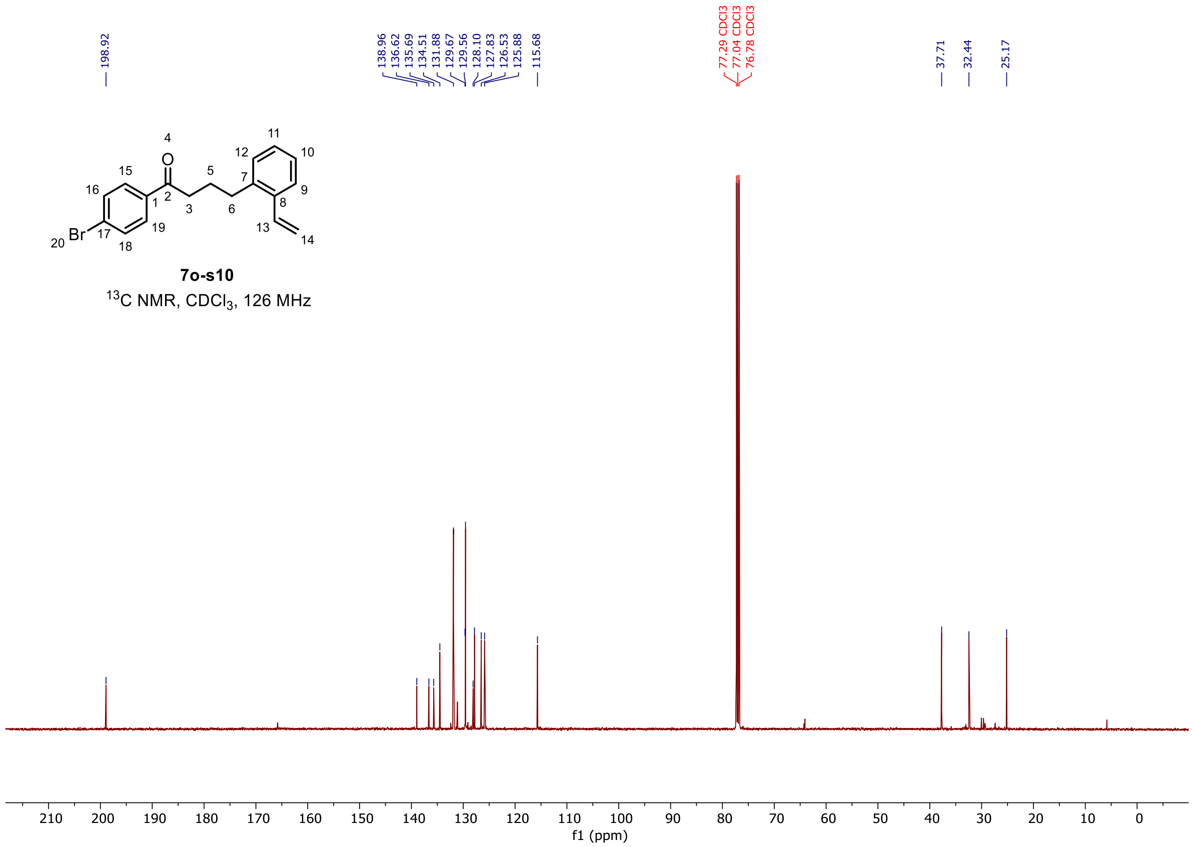

1-(4-Bromophenyl)-2-hydroxy-4-(2-vinylphenyl)butan-1-one 7o

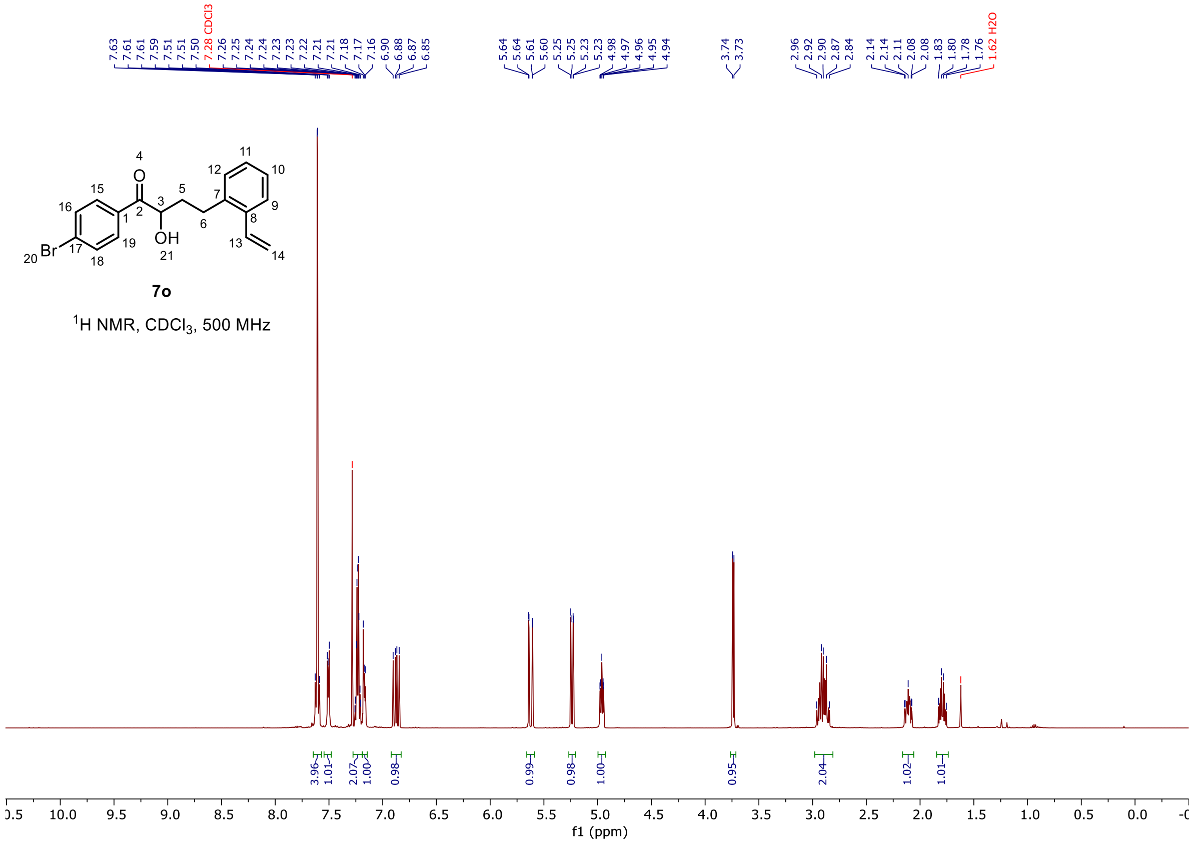

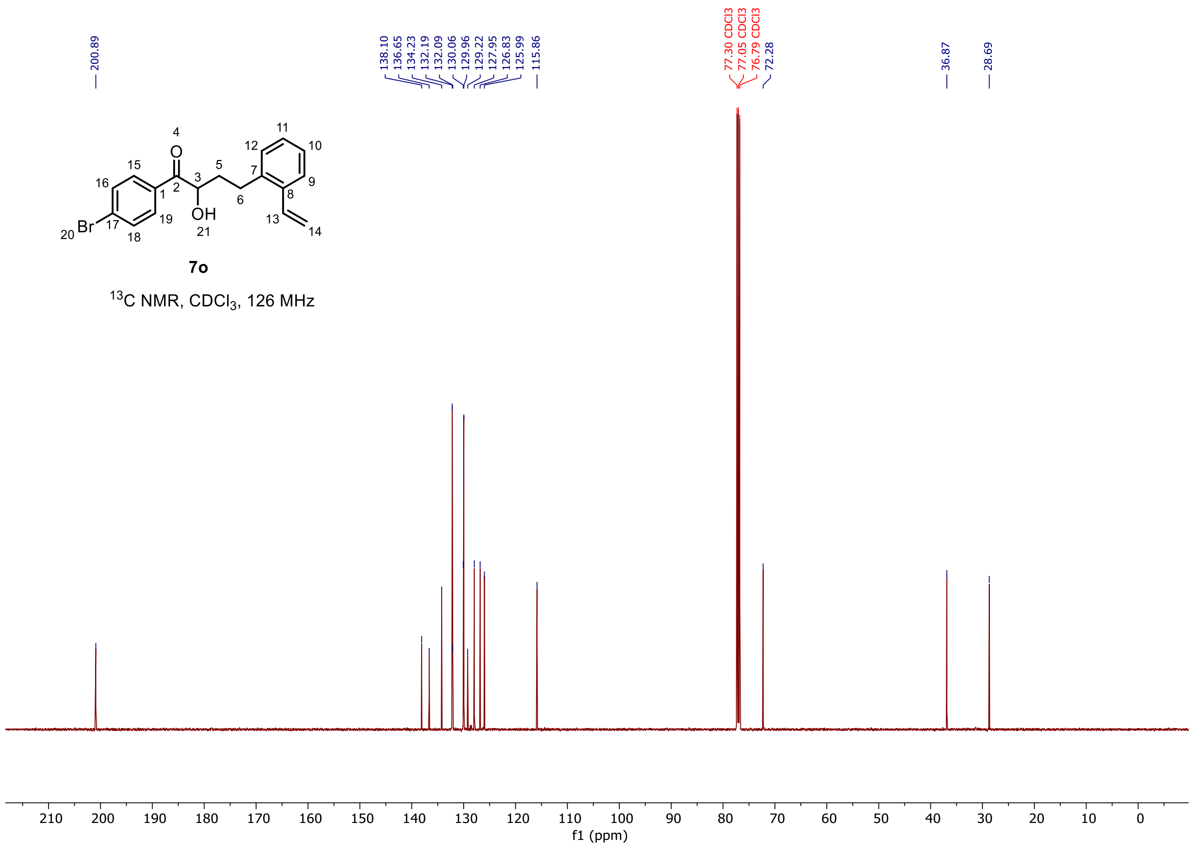

1-(3-(Trifluoromethyl)phenyl)-4-(2-vinylphenyl)butan-1-one 7p-s10

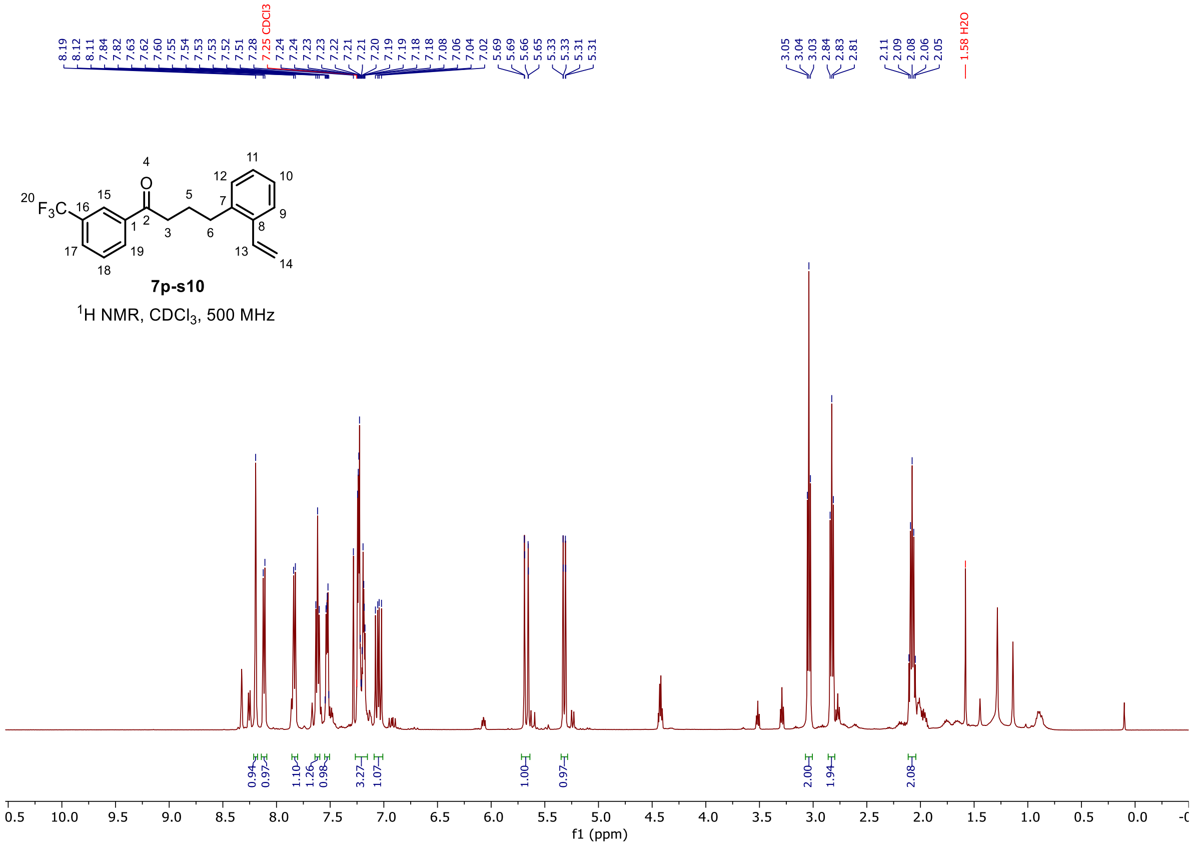

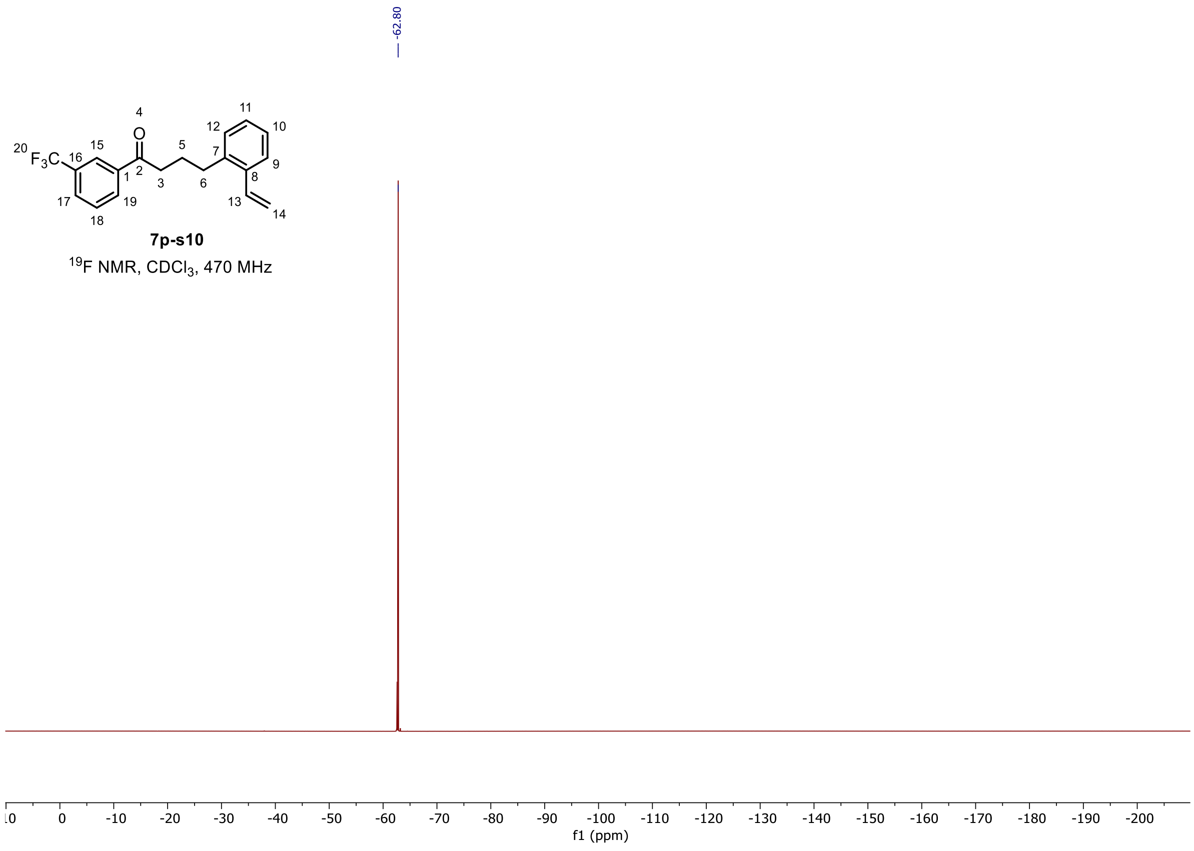

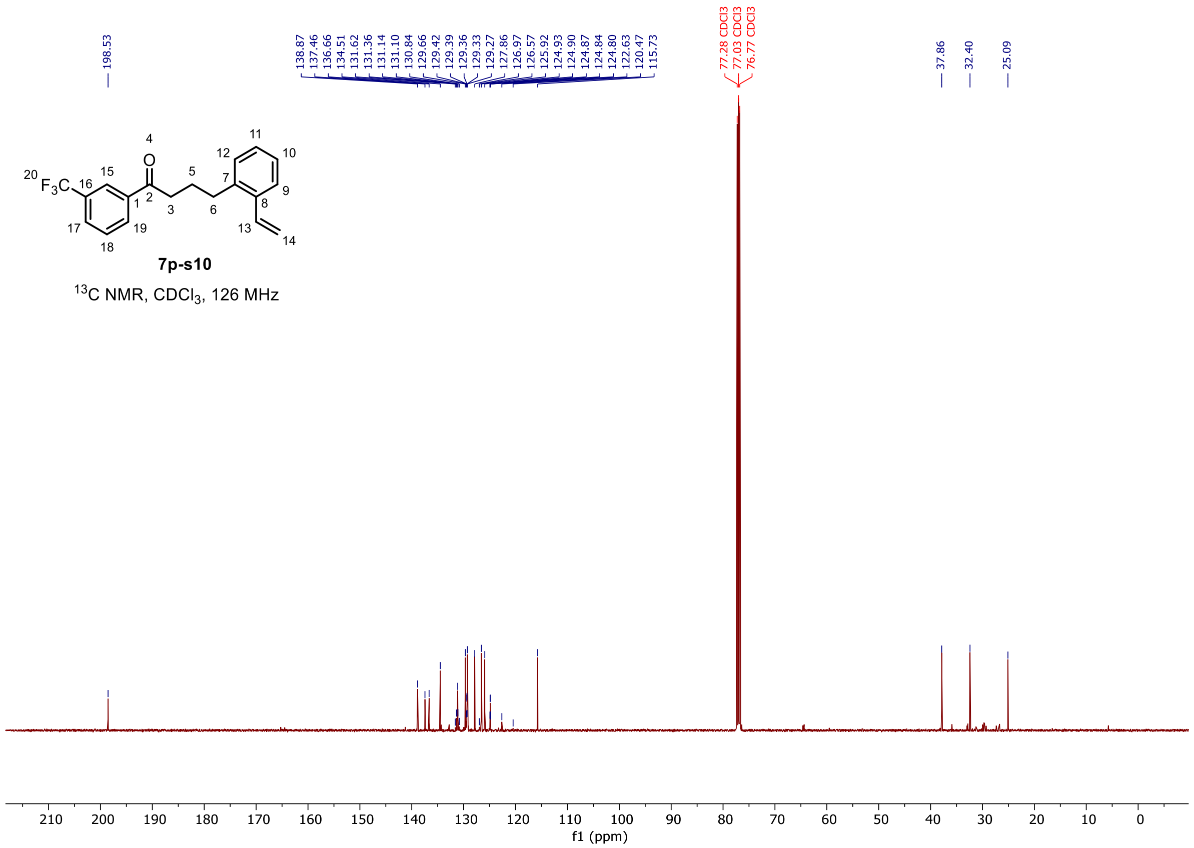
198.53
138.87 137.46 136.66 134.51 131.62 131.36 131.14 131.10 130.84 129.66 129.42 129.39 129.36 129.33 129.27 127.86 126.97 126.57 125.92 124.93 124.90 124.87 124.84 124.80 122.63 120.47 115.73
77.28 CDCl3 77.03 CDCl3 76.77 CDCl3
37.86 32.40 25.09
7p-s10
13C NMR, CDCl3, 126 MHz
f1 (ppm)

2-Hydroxy-1-(3-(trifluoromethyl)phenyl)-4-(2-vinylphenyl)butan-1-one 7p

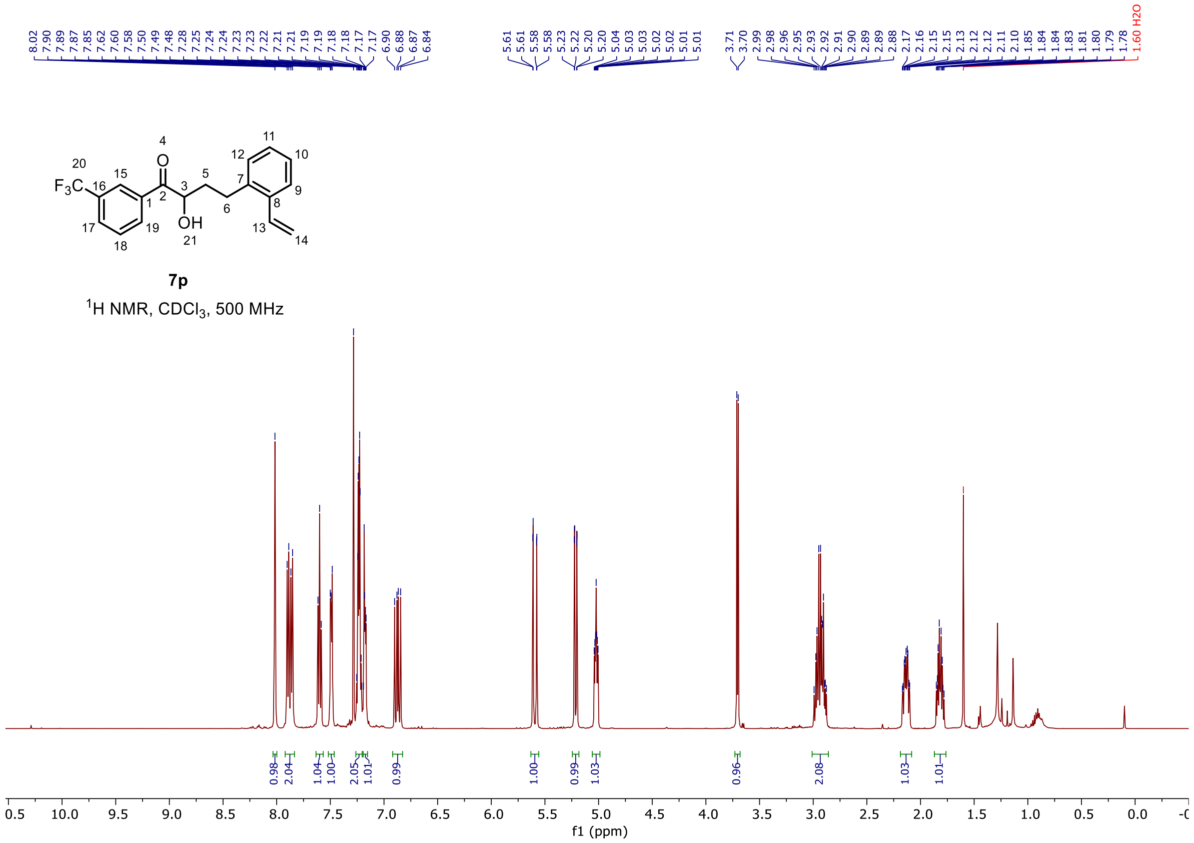

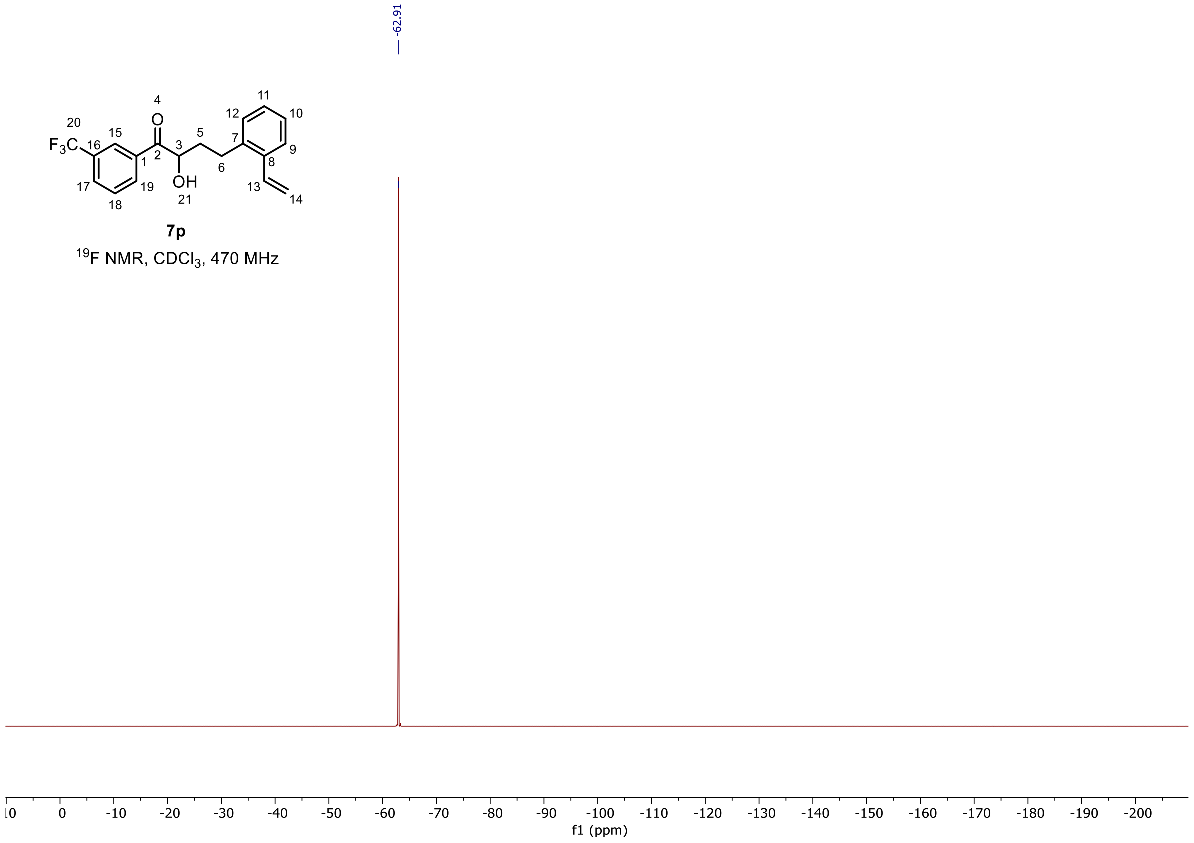

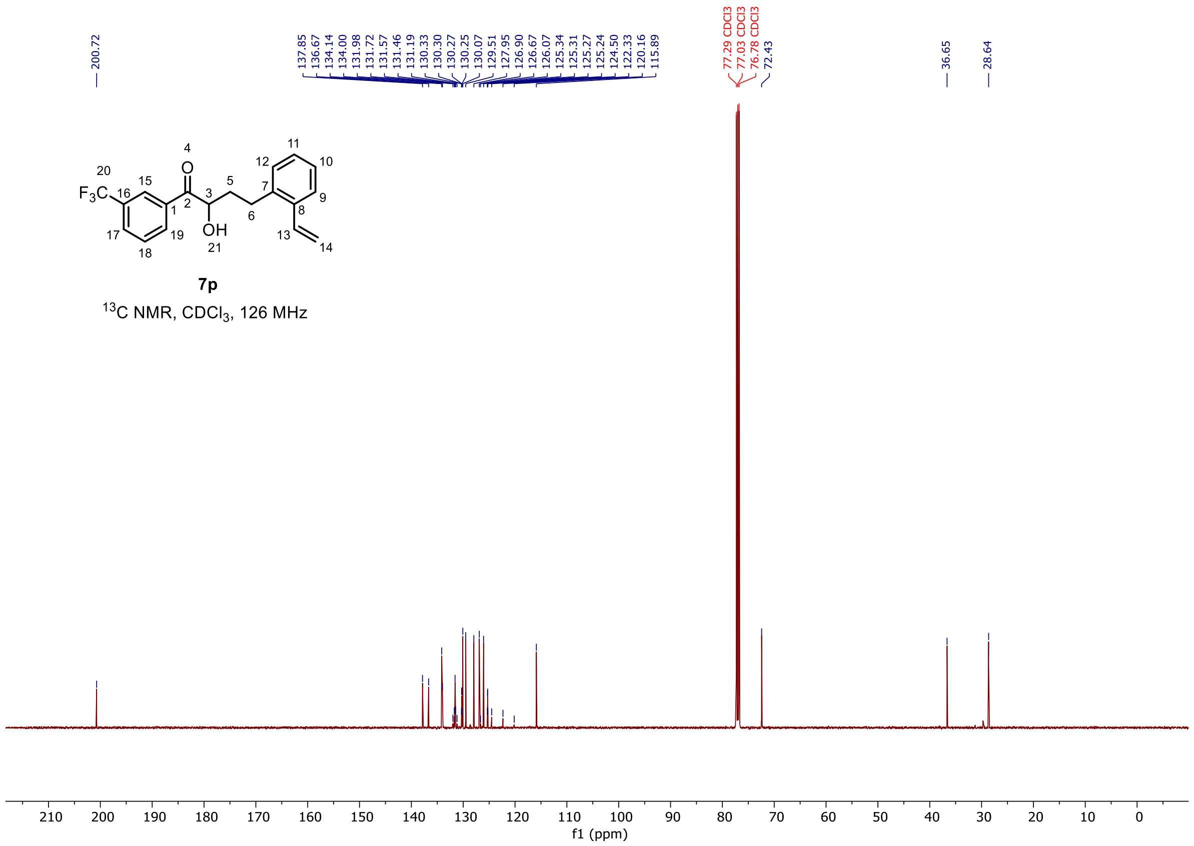
200.72
137.85
136.67
134.14
134.00
131.98
131.72
131.57
131.46
131.19
130.33
130.30
130.27
130.25
130.07
129.51
127.95
126.90
126.67
126.07
125.34
125.31
125.27
125.24
124.50
122.33
120.16
115.89
77.29 CDCl3
77.03 CDCl3
76.78 CDCl3
72.43
36.65
28.64
F3C
OH
7p
13C NMR, CDCl3, 126 MHz
f1 (ppm)

1-(3-Methoxyphenyl)-4-(2-vinylphenyl)butan-1-one 7q-s10

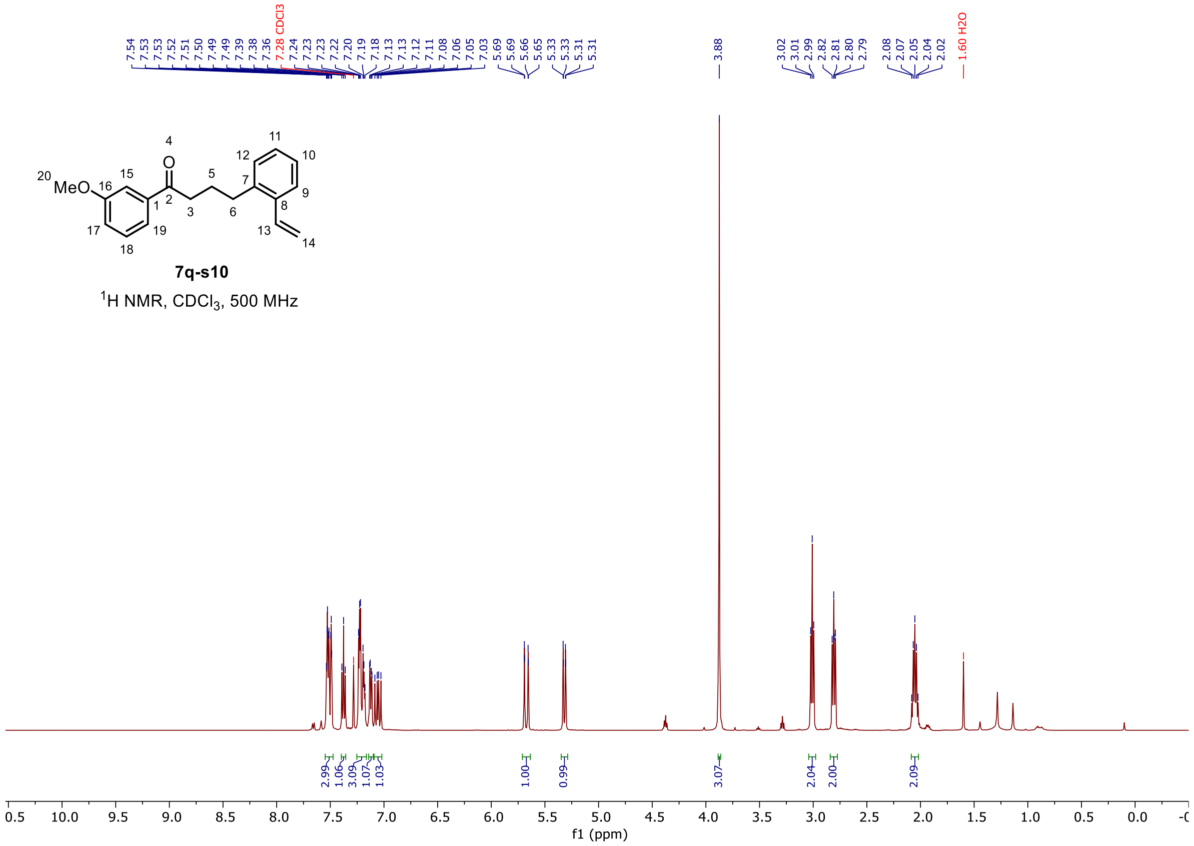

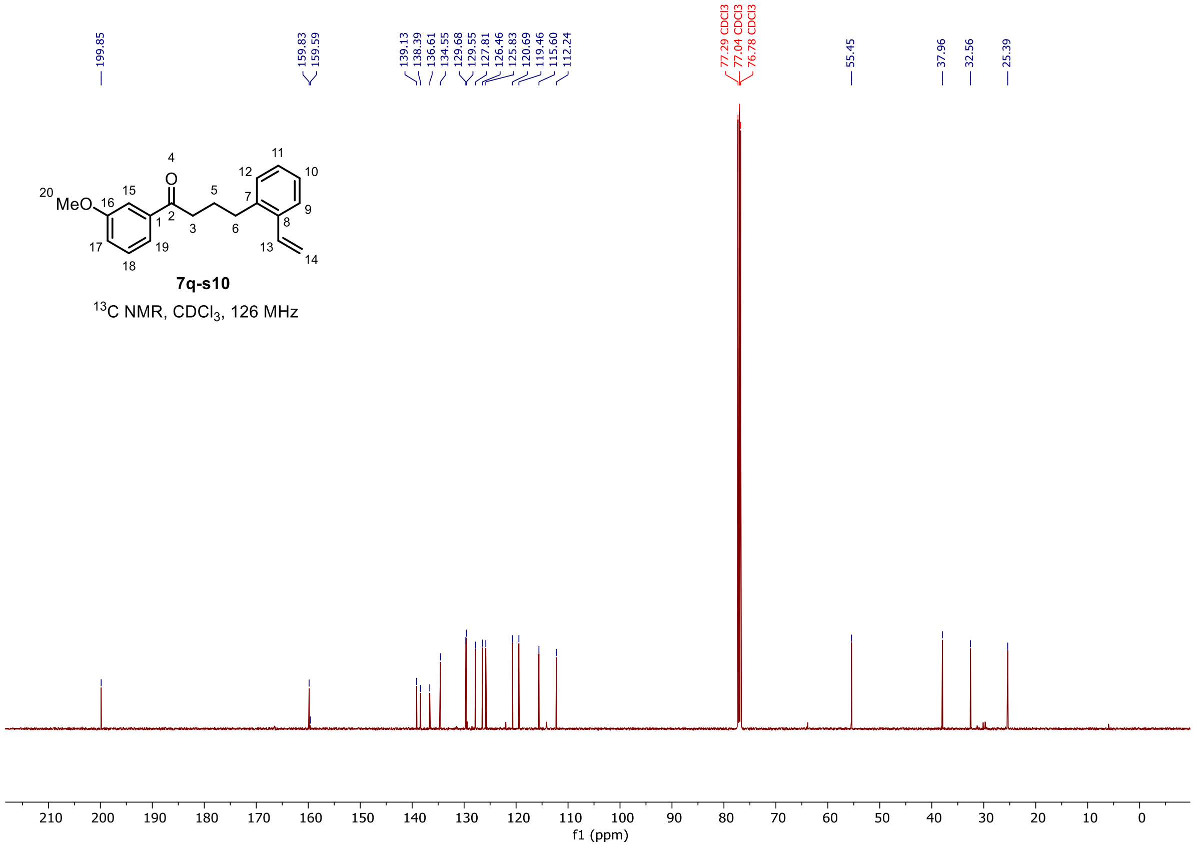

2-Hydroxy-1-(3-methoxyphenyl)-4-(2-vinylphenyl)butan-1-one 7q

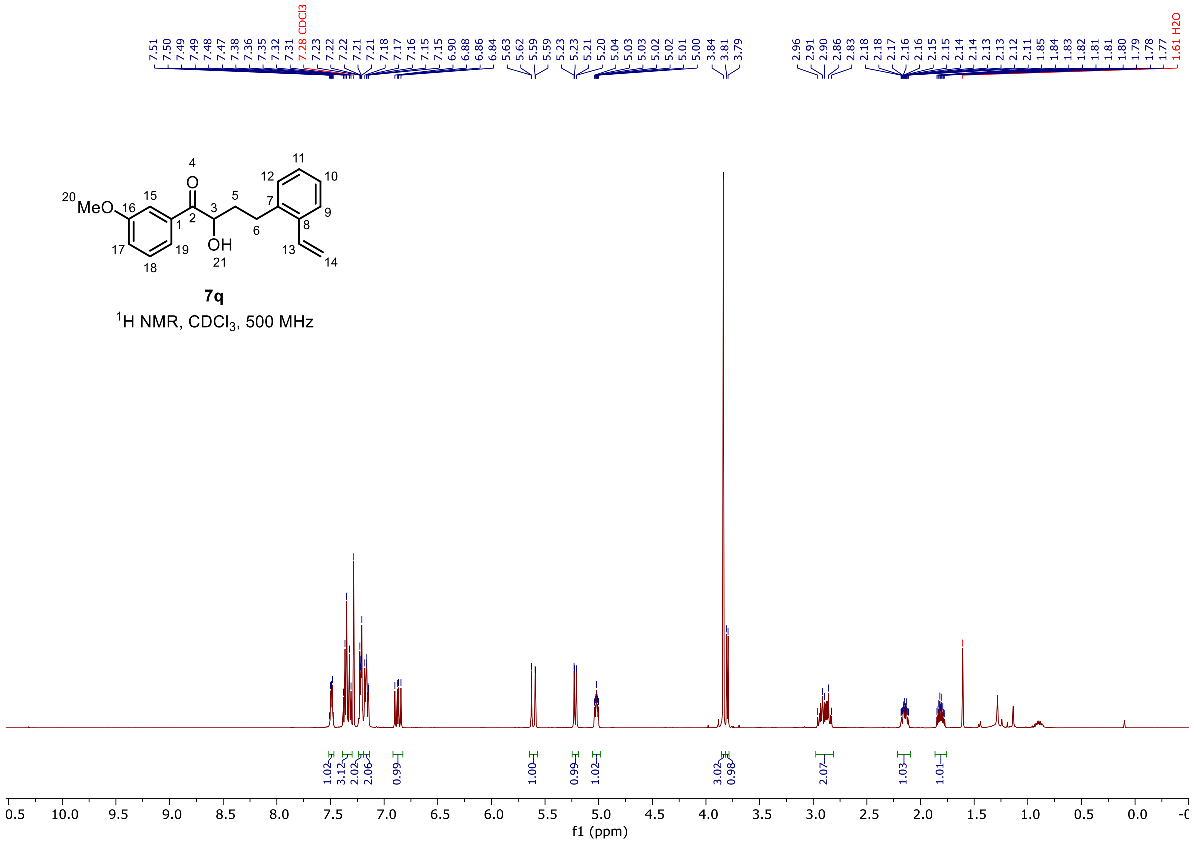

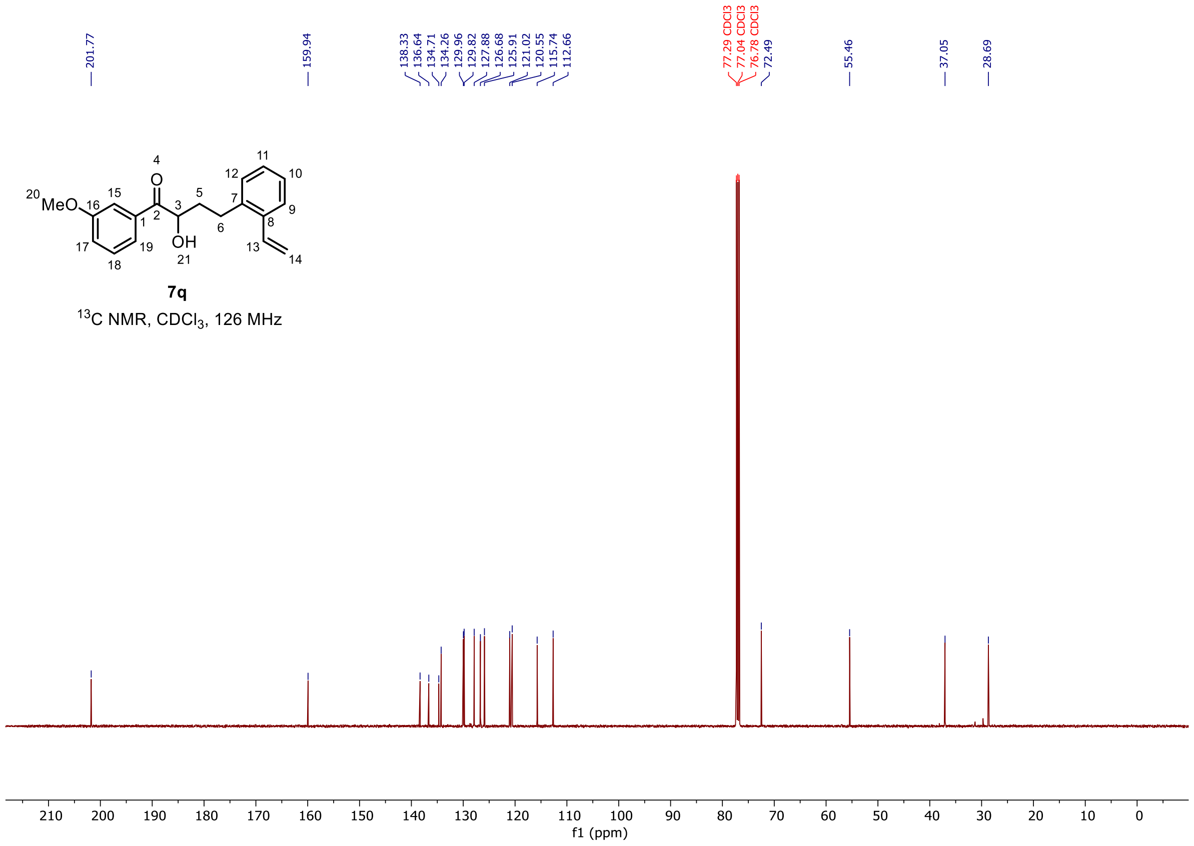

1-(2-Fluorophenyl)-4-(2-vinylphenyl)butan-1-one 7r-s10

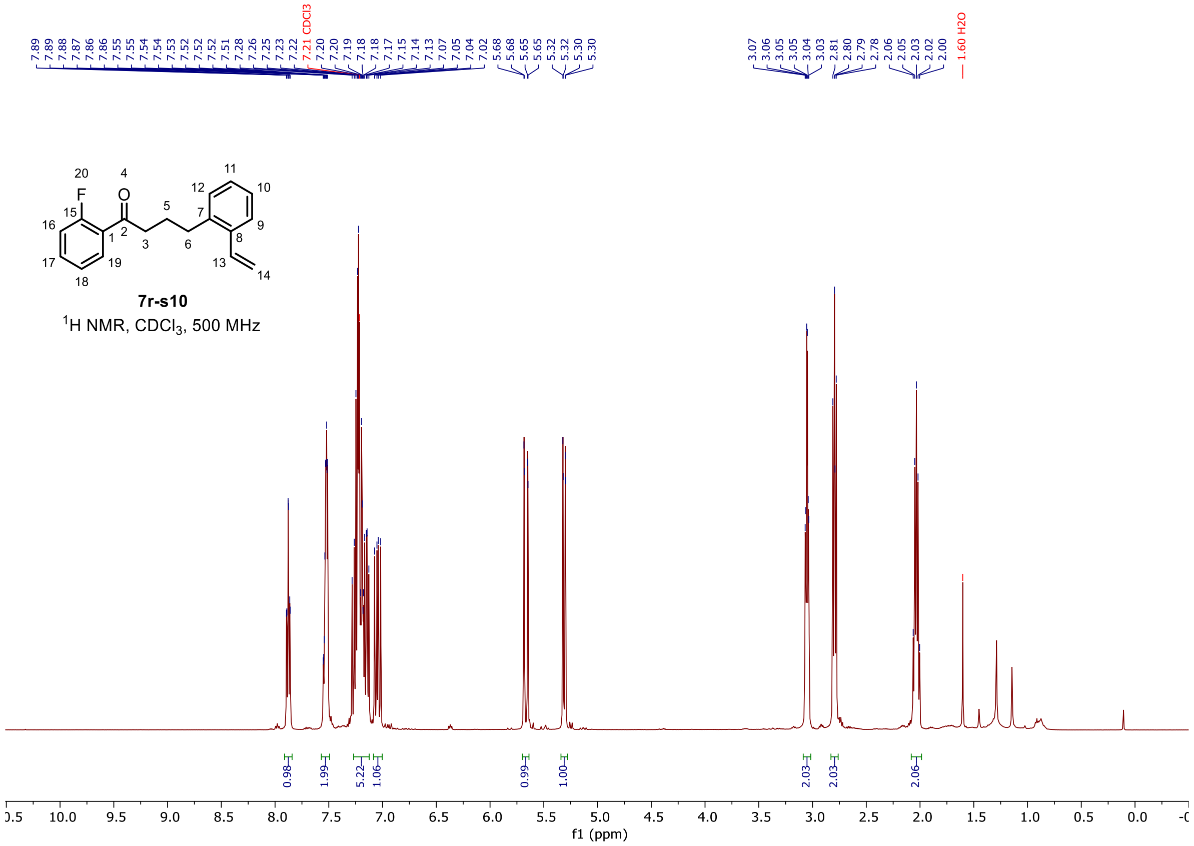

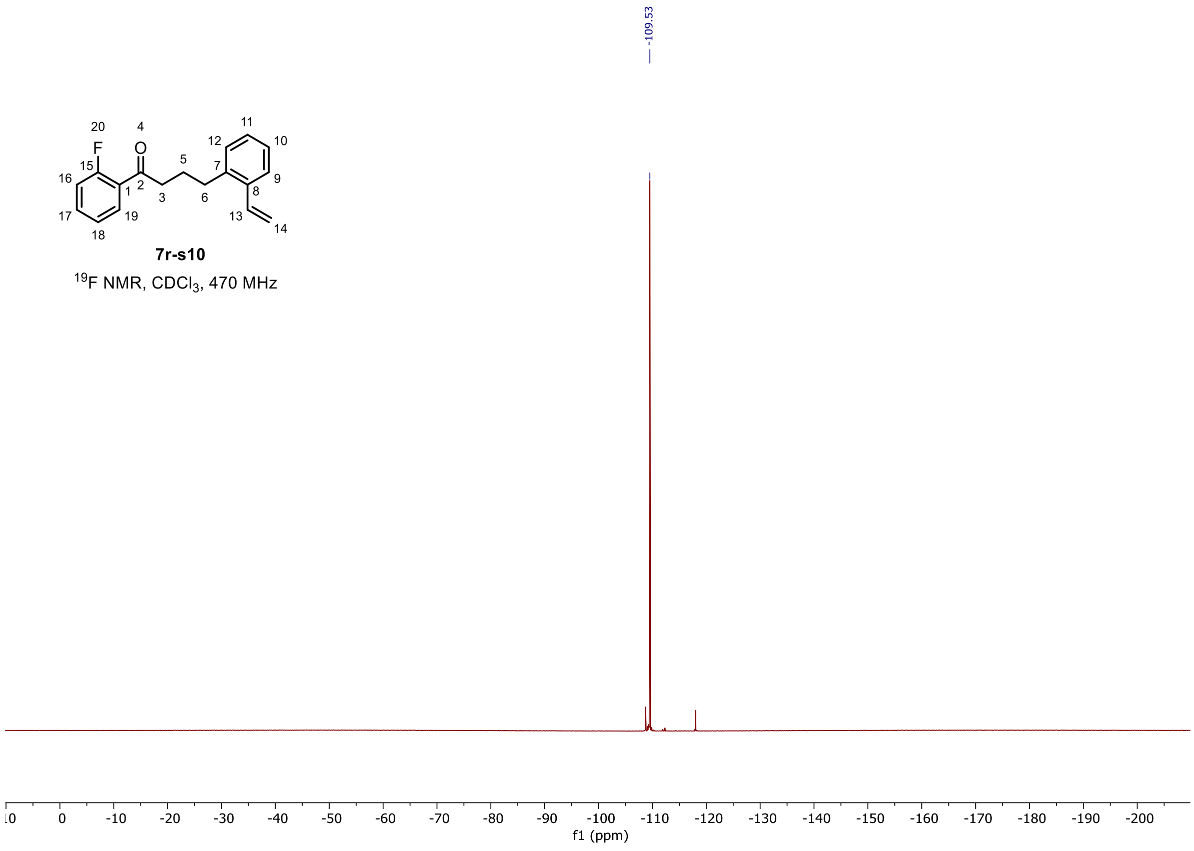

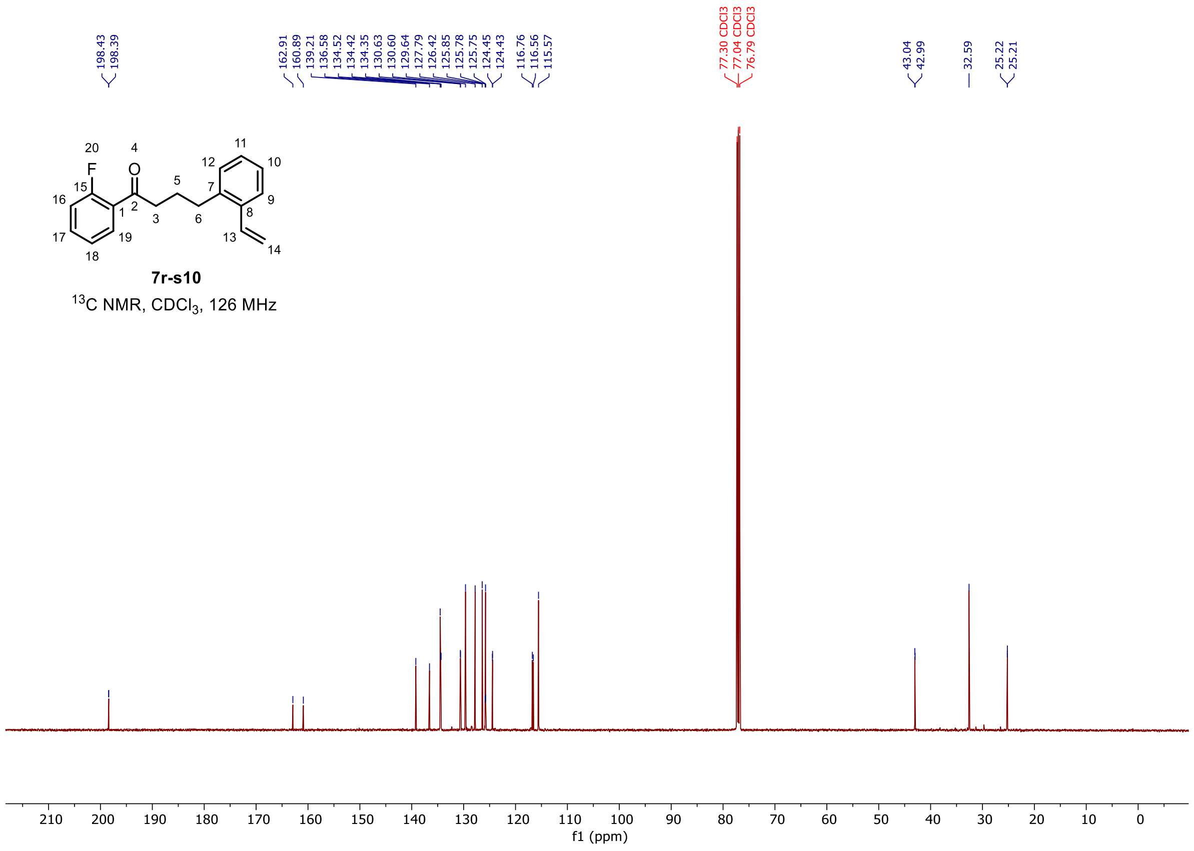
198.43
198.39
162.91
160.89
139.21
136.58
134.52
134.42
134.35
130.63
130.60
129.64
127.79
126.42
125.85
125.78
125.75
124.45
124.43
116.76
116.56
115.57
77.30 CDCl3
77.04 CDCl3
76.79 CDCl3
43.04
42.99
32.59
25.22
25.21
7r-s10
13C NMR, CDCl3, 126 MHz
f1 (ppm)

1-(2-Fluorophenyl)-2-hydroxy-4-(2-vinylphenyl)butan-1-one 7r

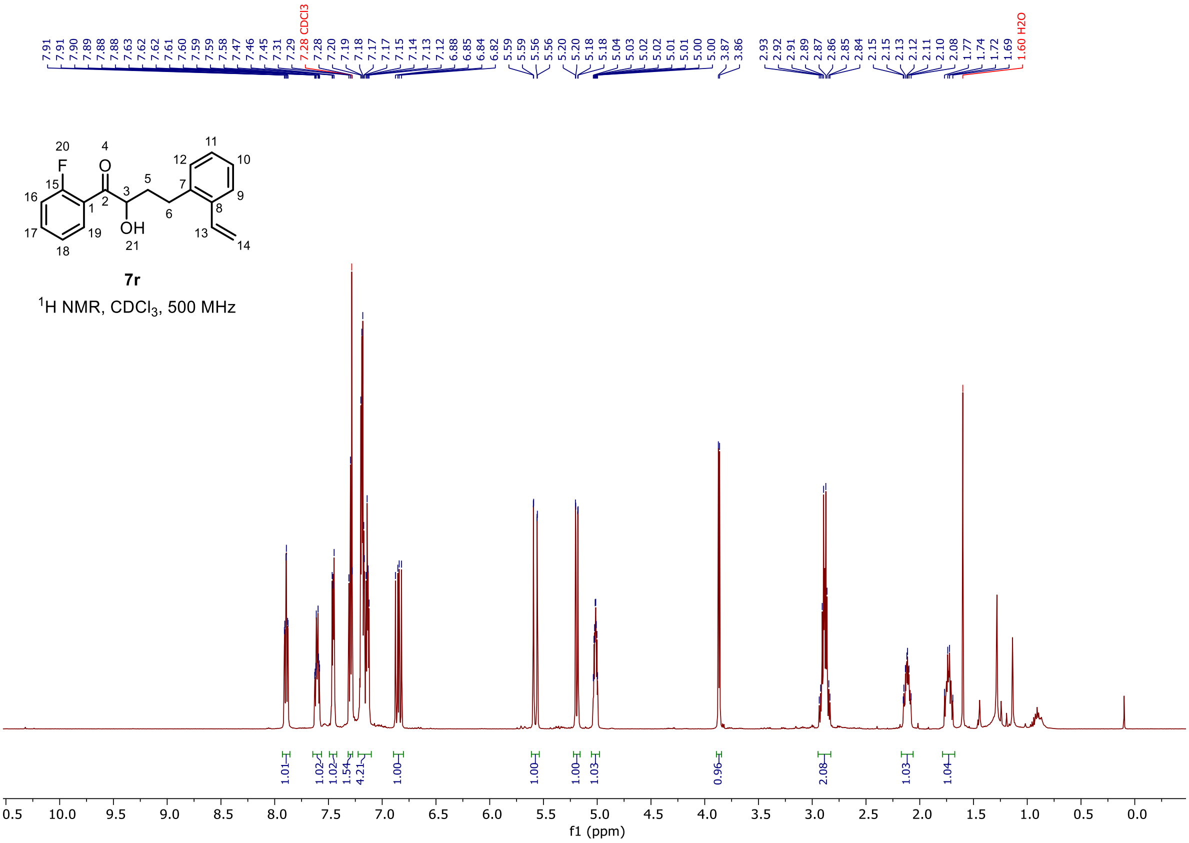

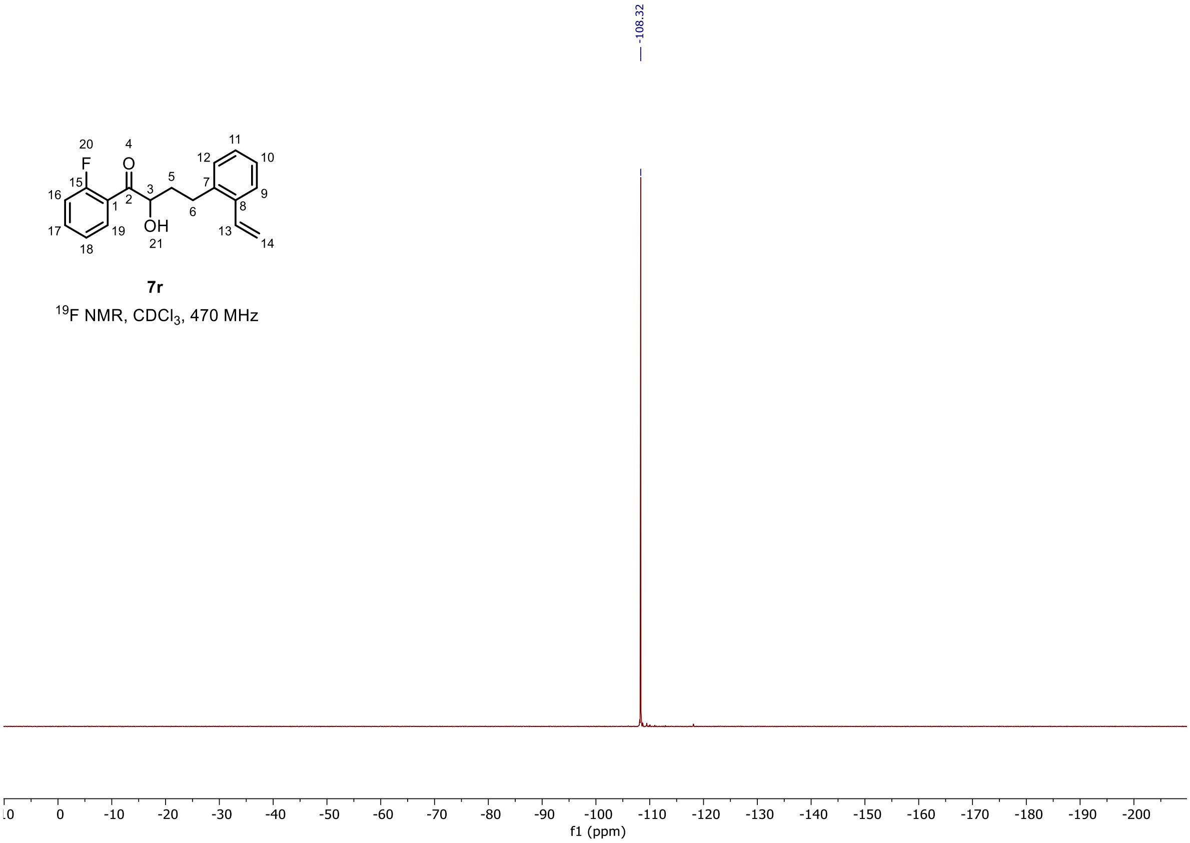

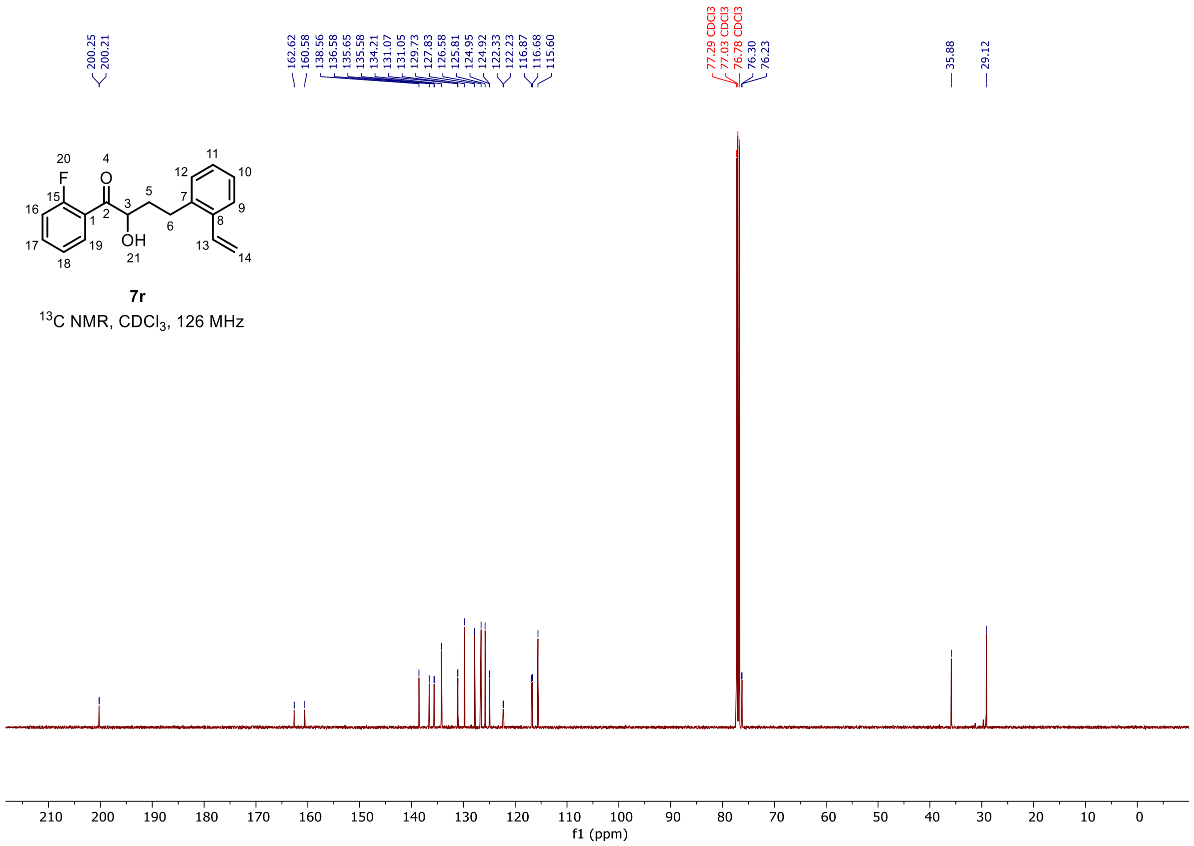
200.25
200.21
162.62
160.58
138.56
136.58
135.65
135.58
134.21
131.07
131.05
129.73
127.83
126.58
125.81
124.95
124.92
122.33
122.23
116.87
116.68
115.60
77.29 CDCl3
77.03 CDCl3
76.78 CDCl3
76.30
76.23
35.88
29.12
F
O
OH
7r
13C NMR, CDCl3, 126 MHz
f1 (ppm)

1-(Naphthalen-2-yl)-4-(2-vinylphenyl)butan-1-one 7s-s10

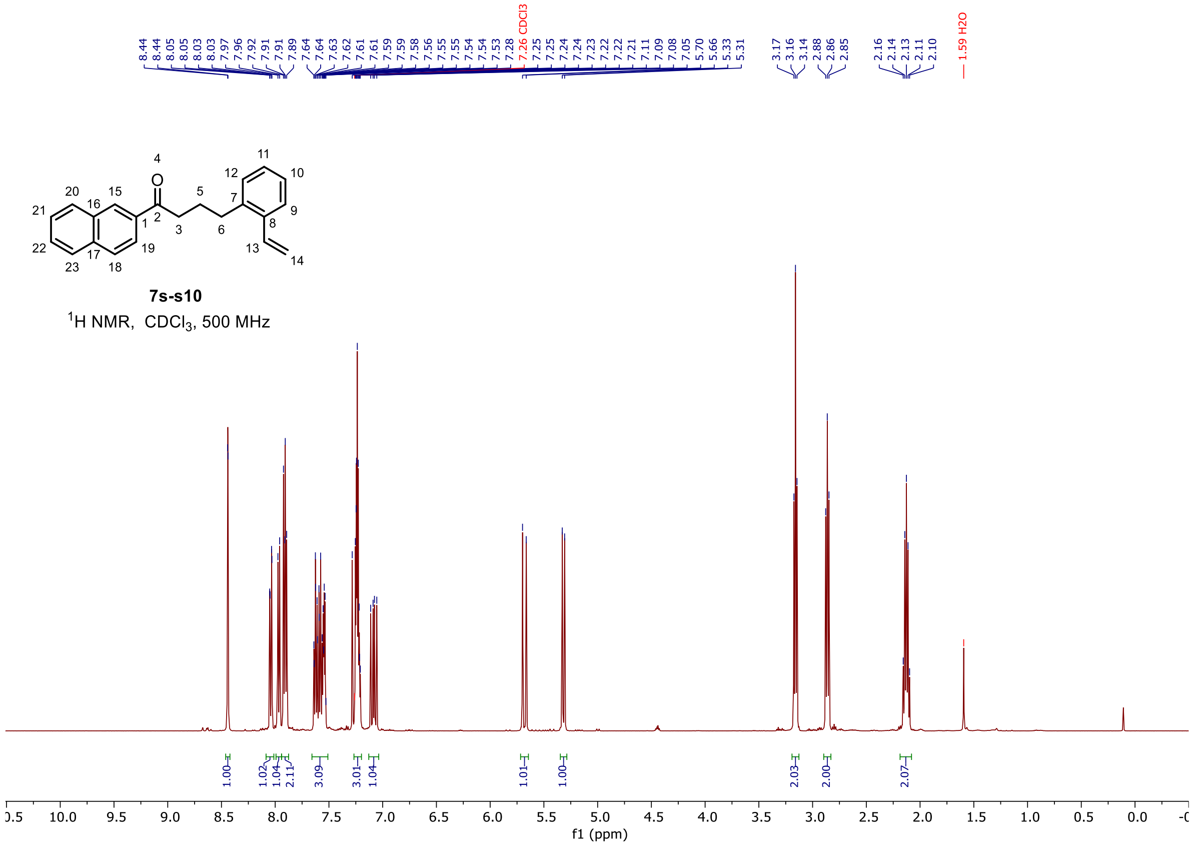

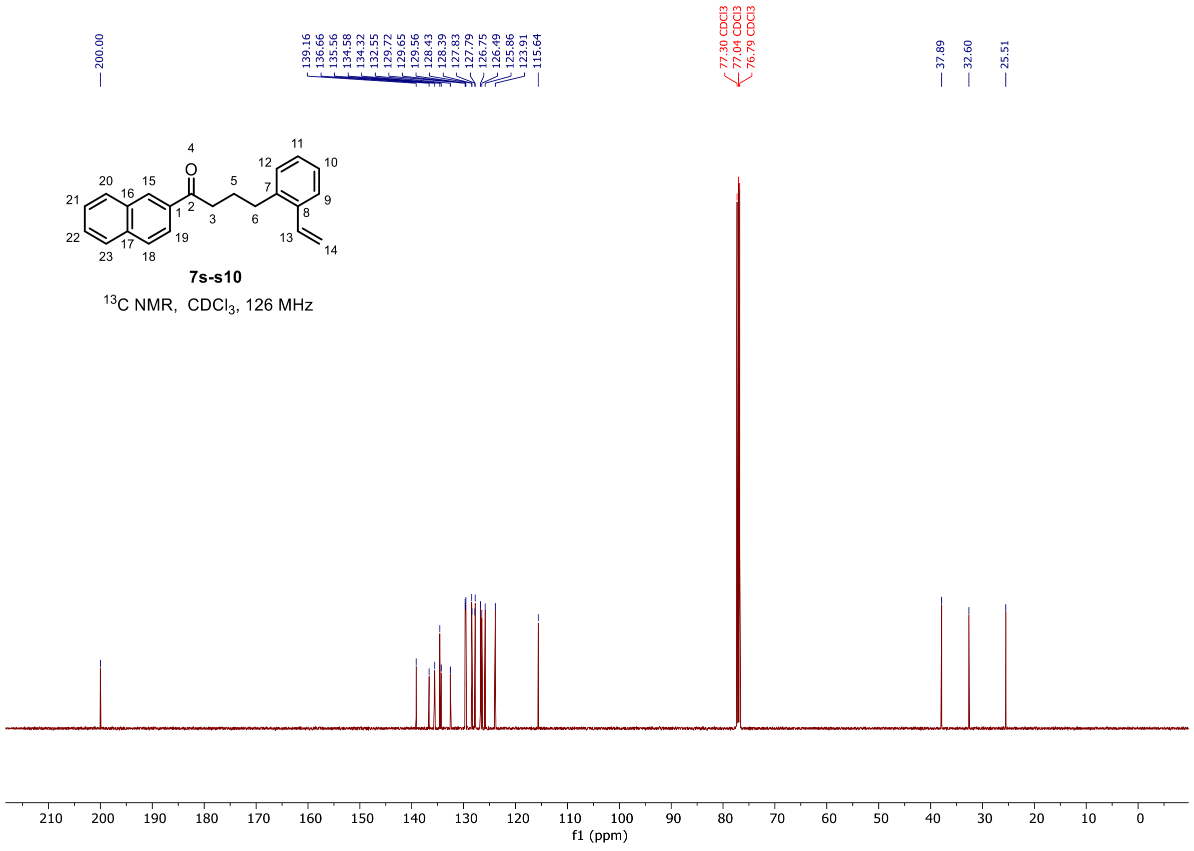

2-Hydroxy-1-(naphthalen-2-yl)-4-(2-vinylphenyl)butan-1-one 7s

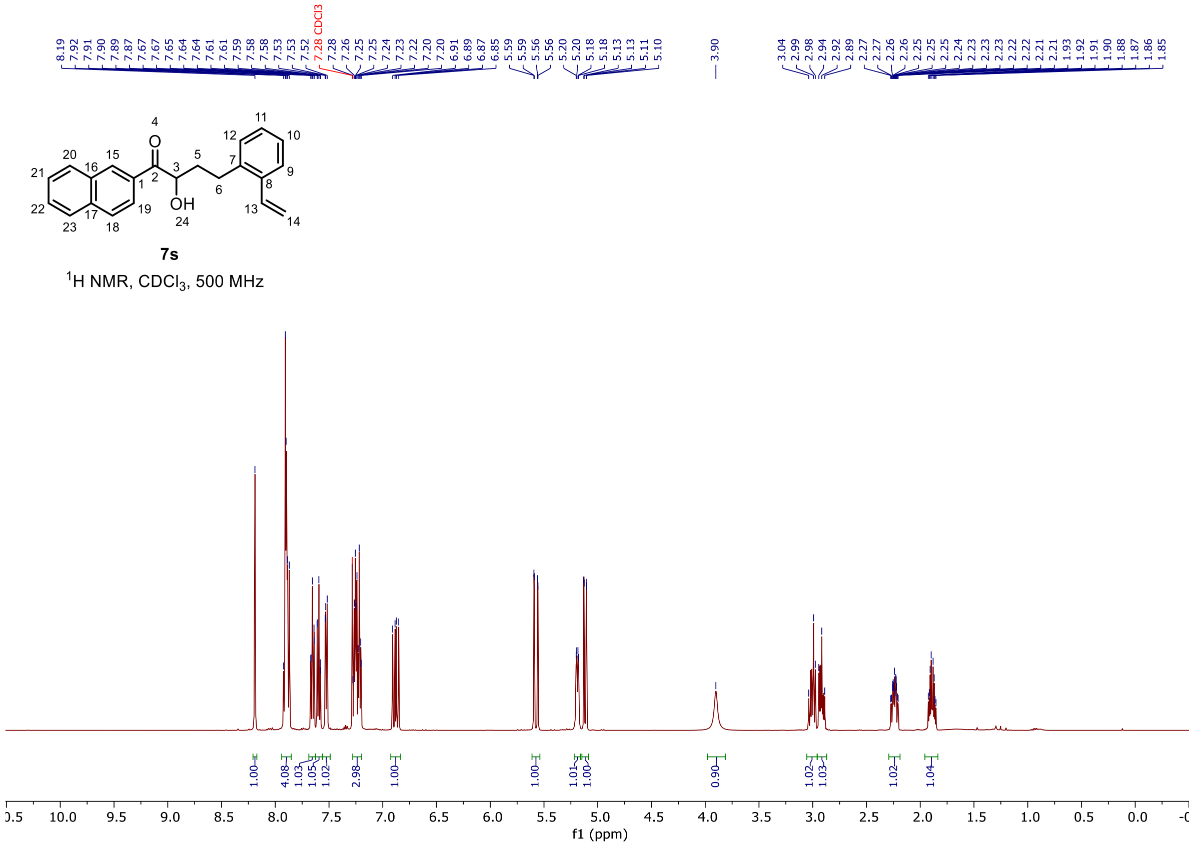

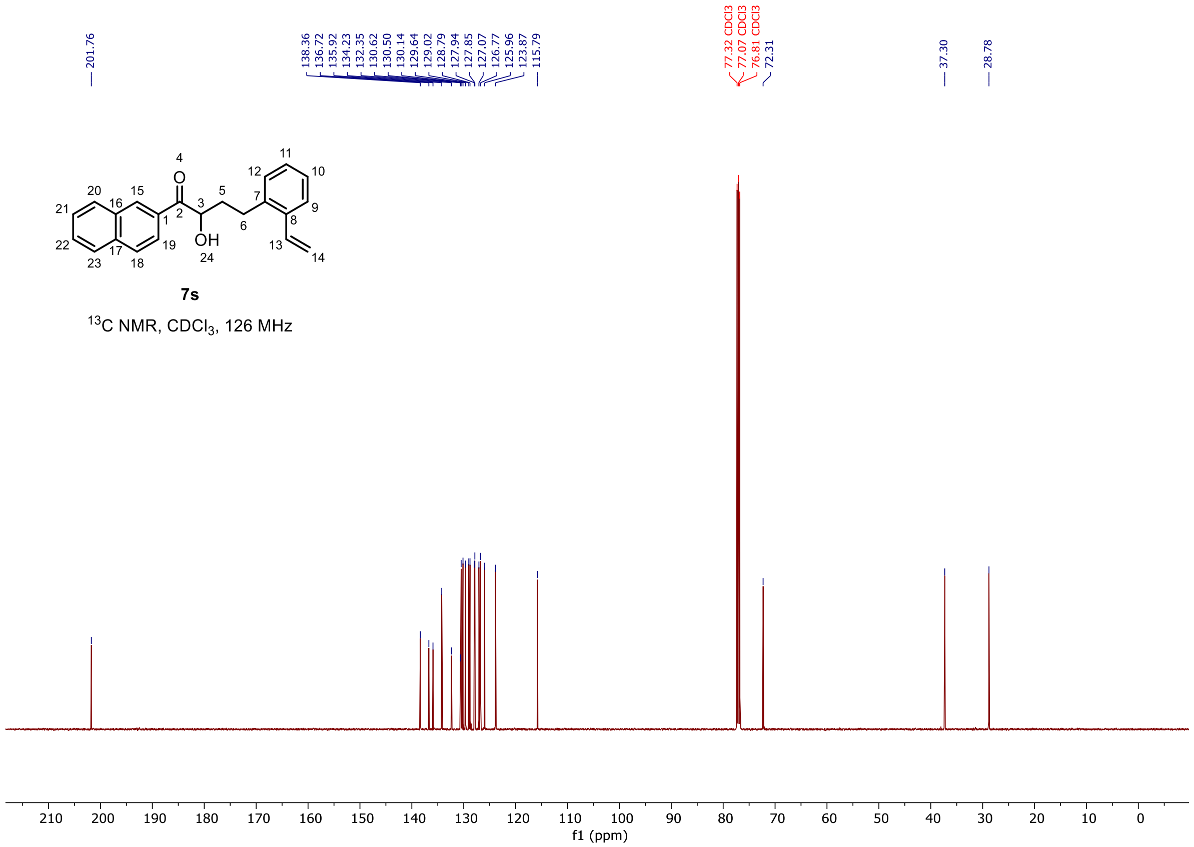

1-(Furan-2-yl)-4-(2-vinylphenyl)butan-1-one 7t-s10

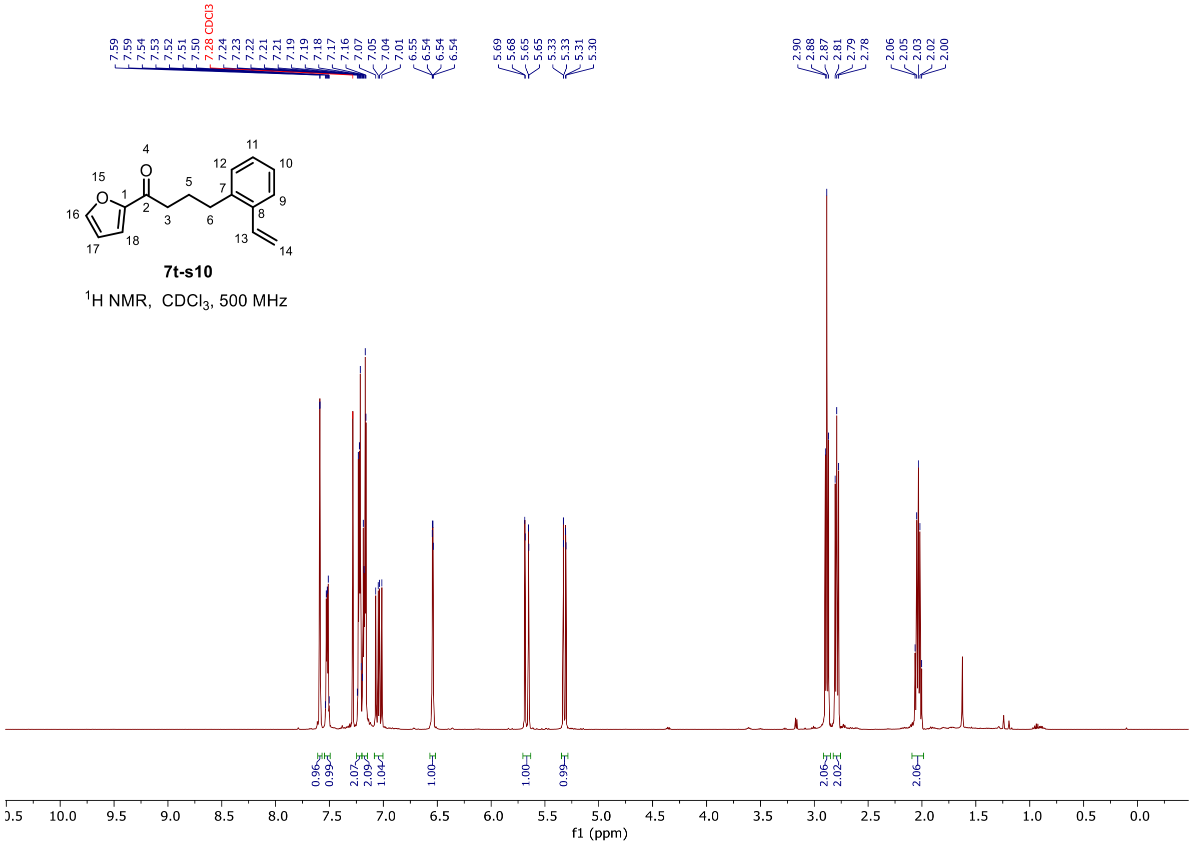

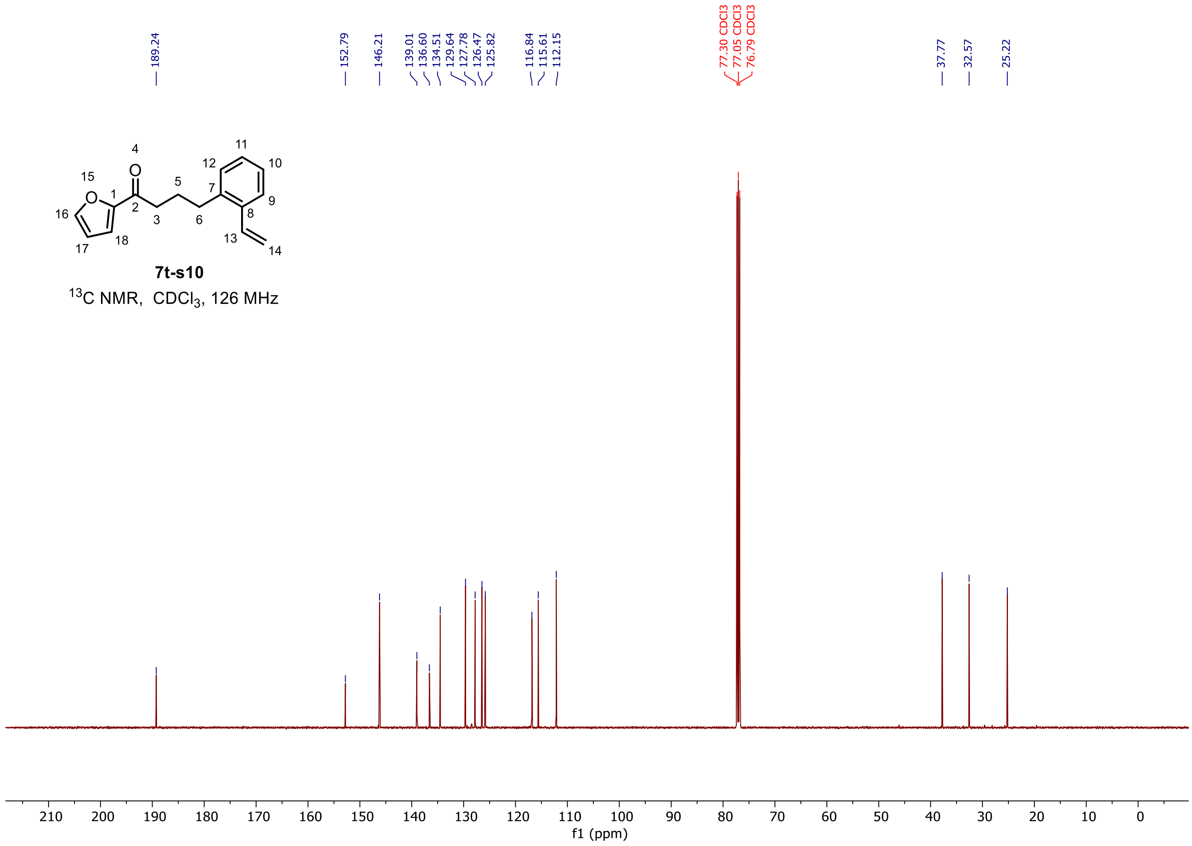

1-(Furan-2-yl)-2-hydroxy-4-(2-vinylphenyl)butan-1-one 7t

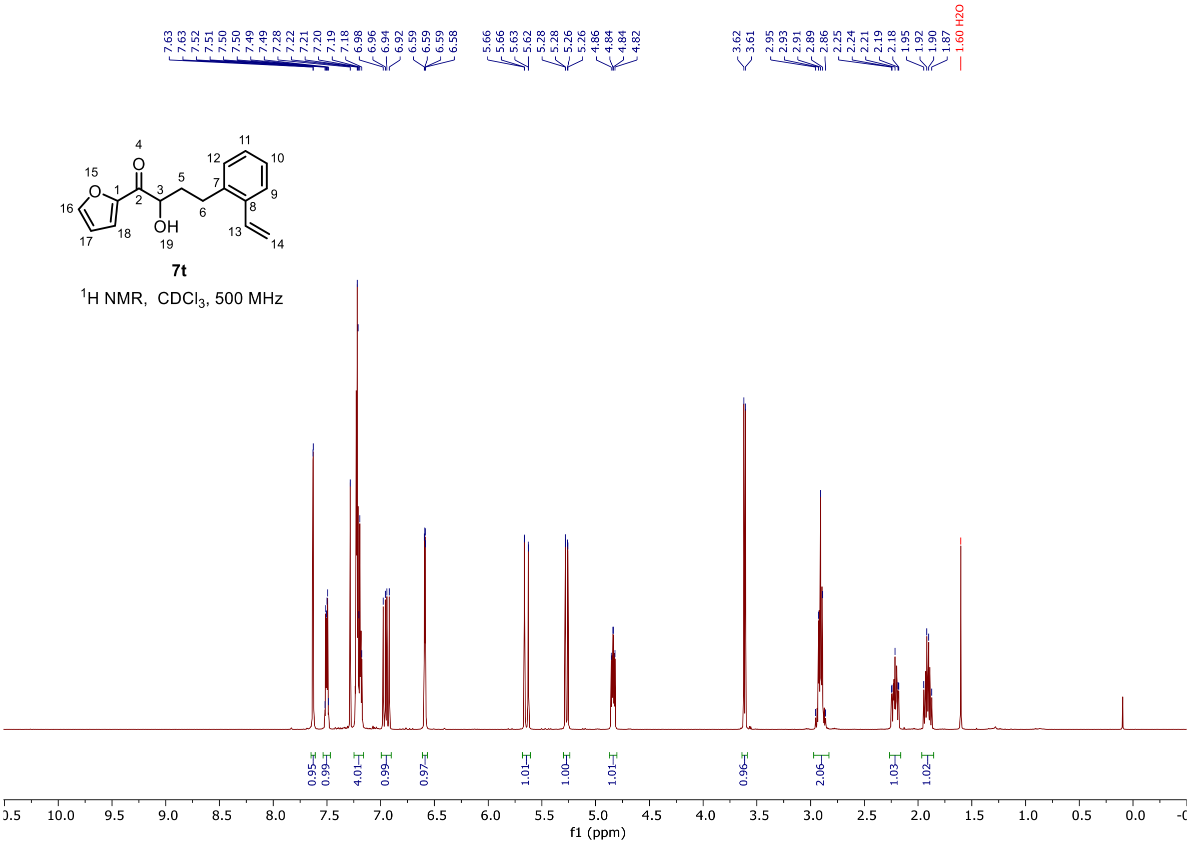

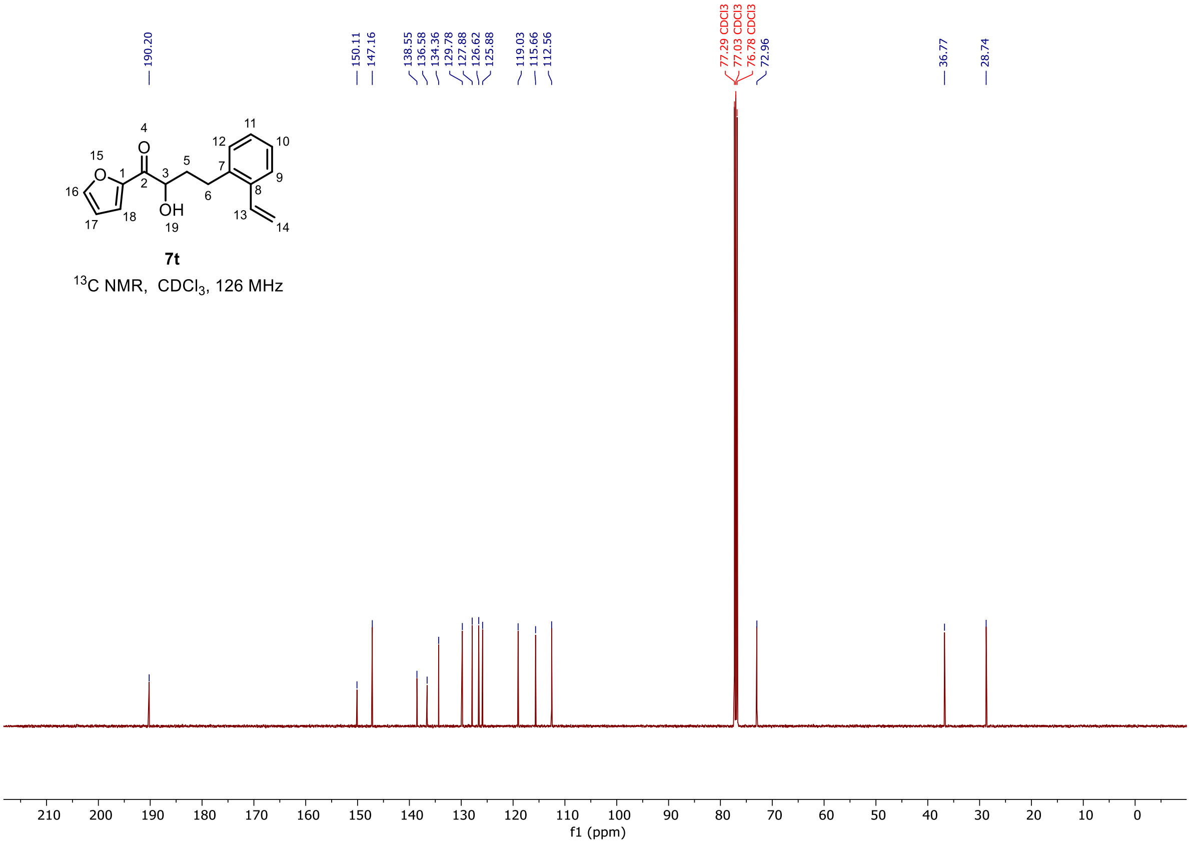

3-Hydroxy-5-(2-vinylphenyl)pentan-2-one 7u

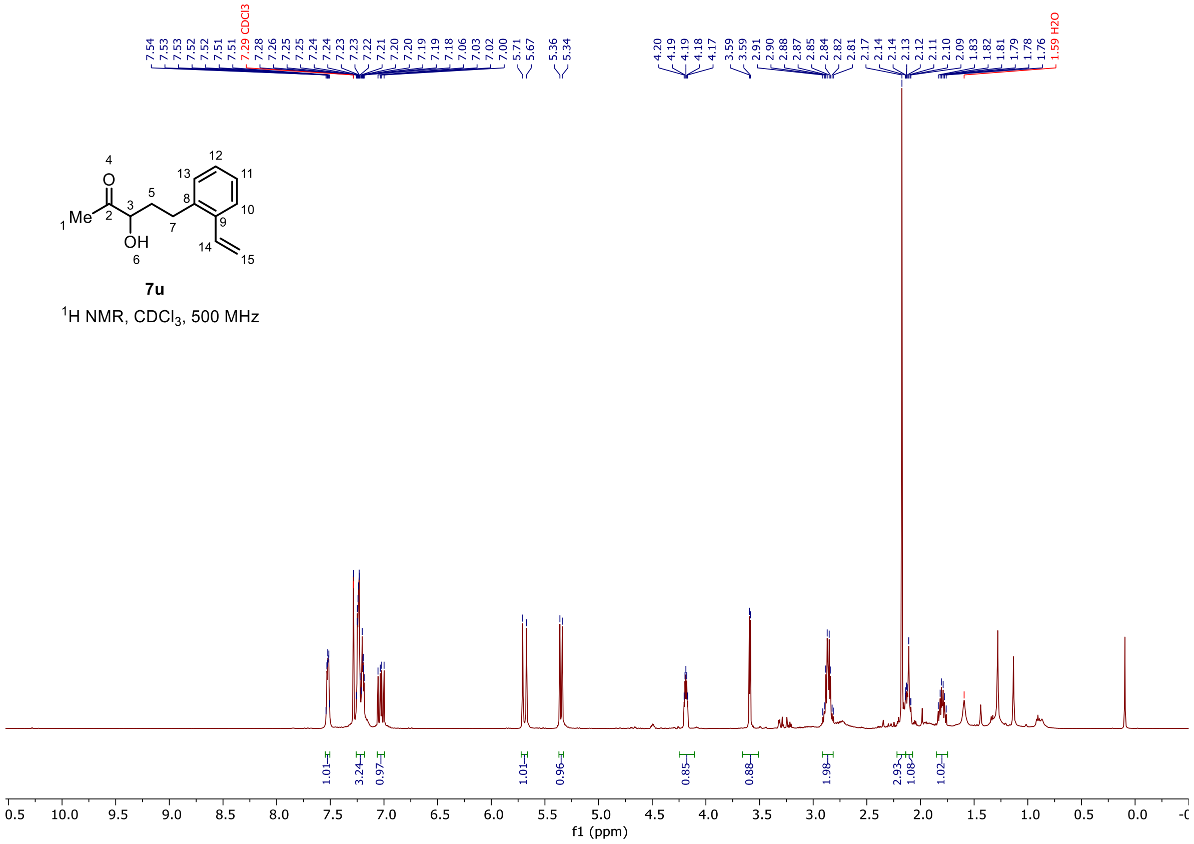

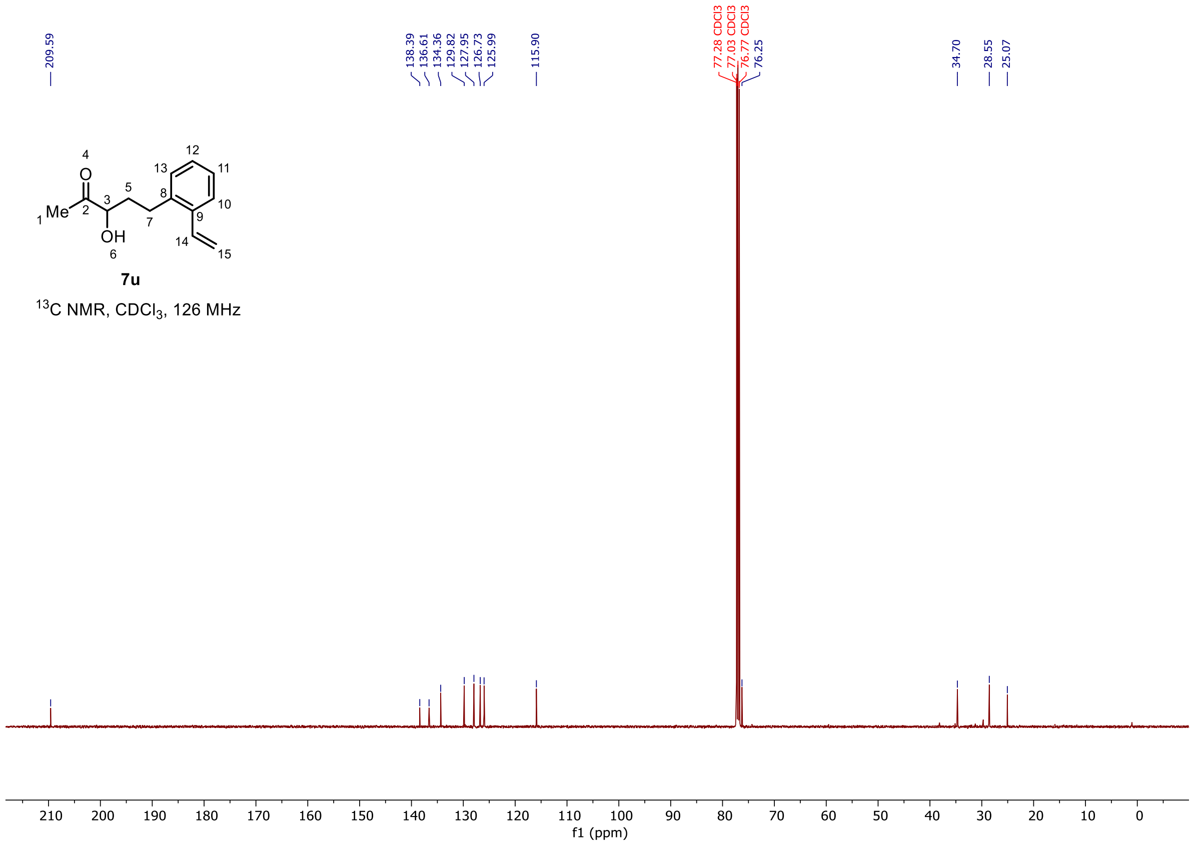

2-Hydroxy-1-phenyl-4-(2-(prop-1-en-2-yl)phenyl)butan-1-one 7v

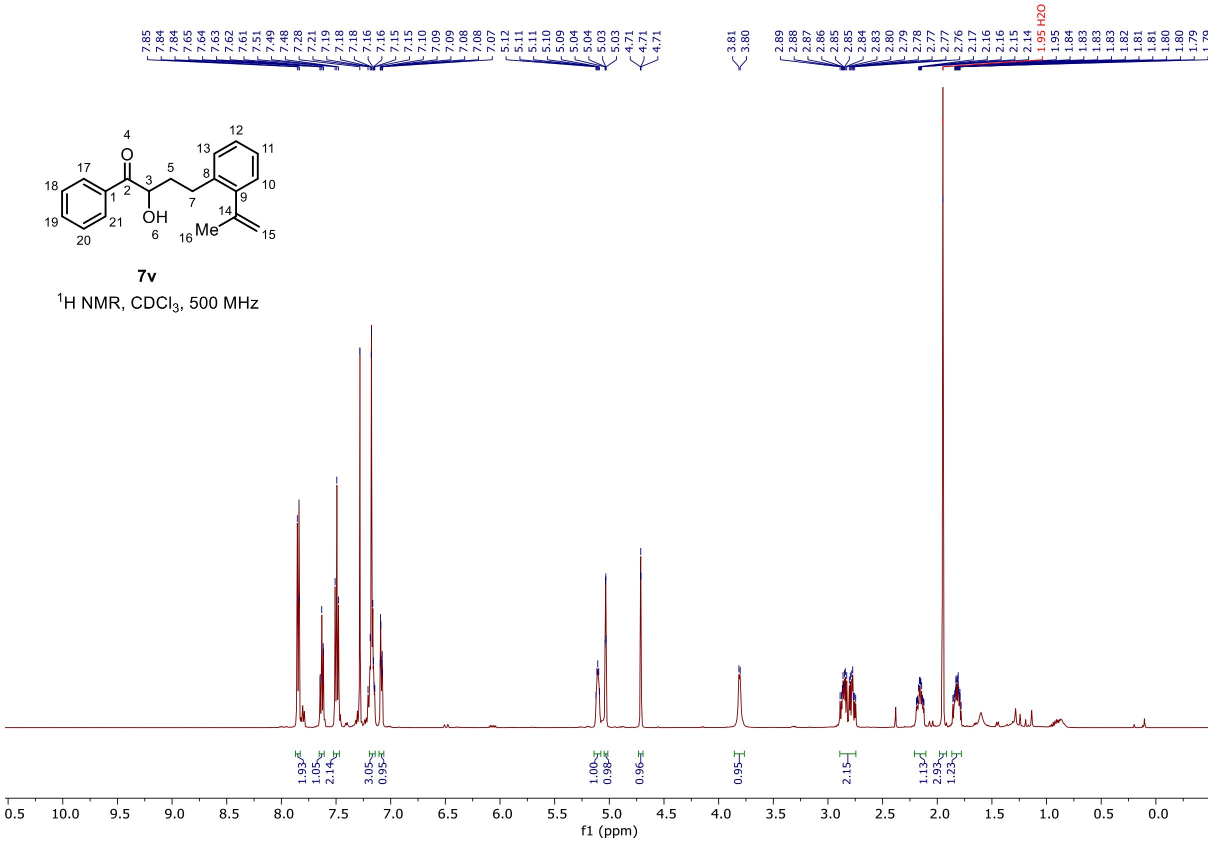

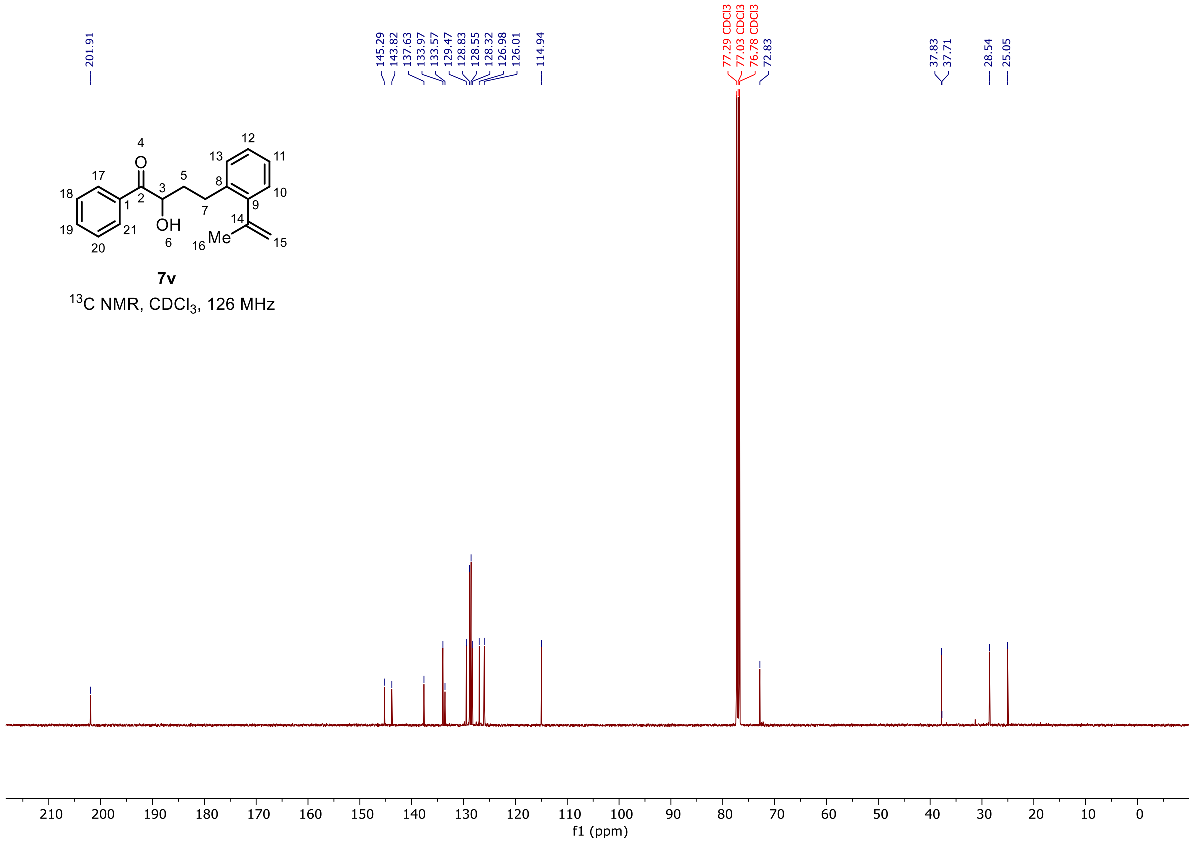

1-Phenyl-3-(2-vinylphenyl)propan-1-one 15-s10

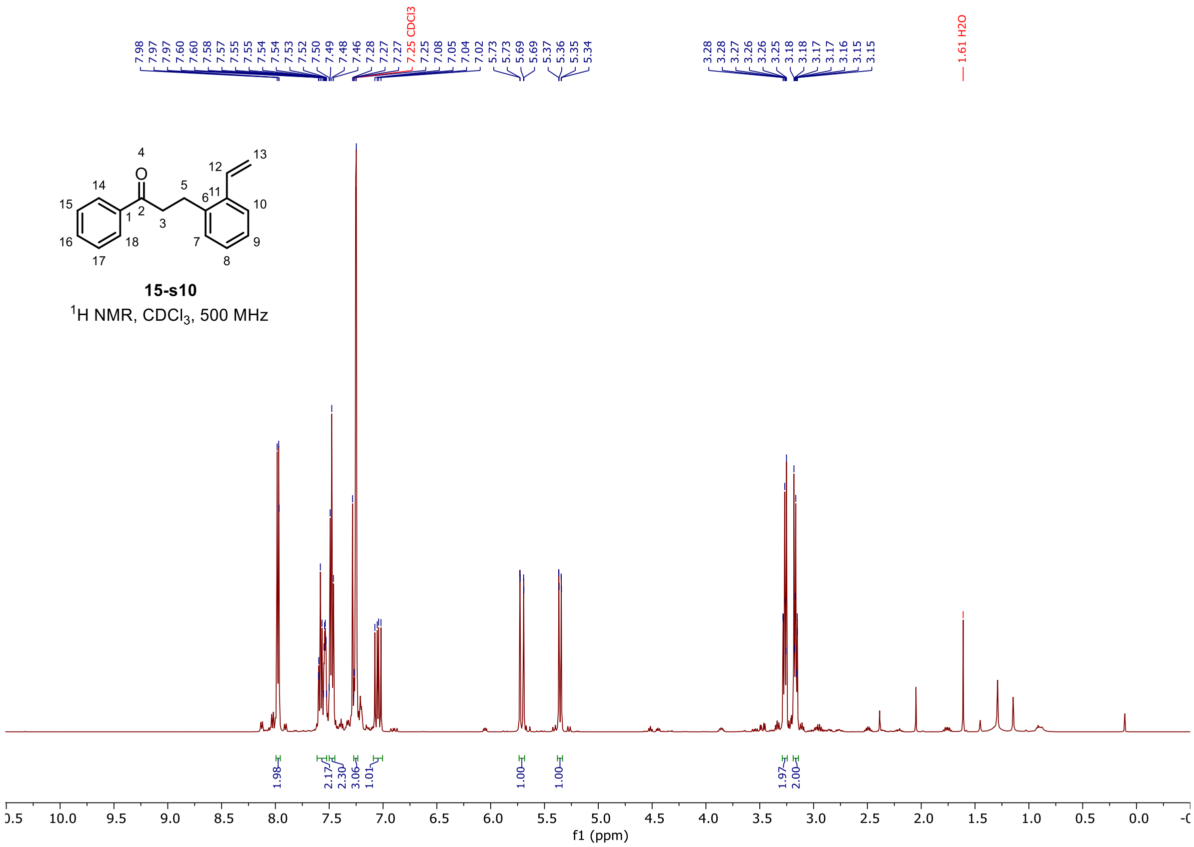

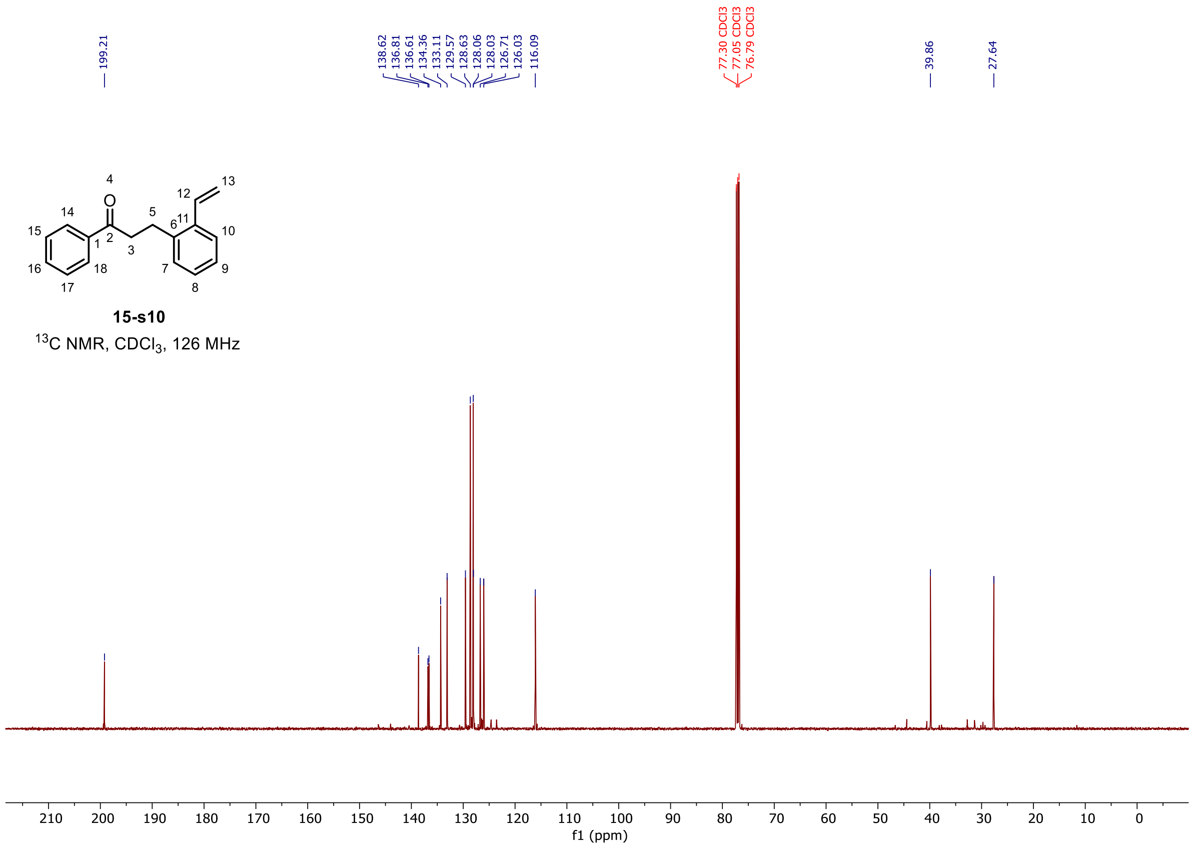

2-Hydroxy-1-phenyl-3-(2-vinylphenyl)propan-1-one 15

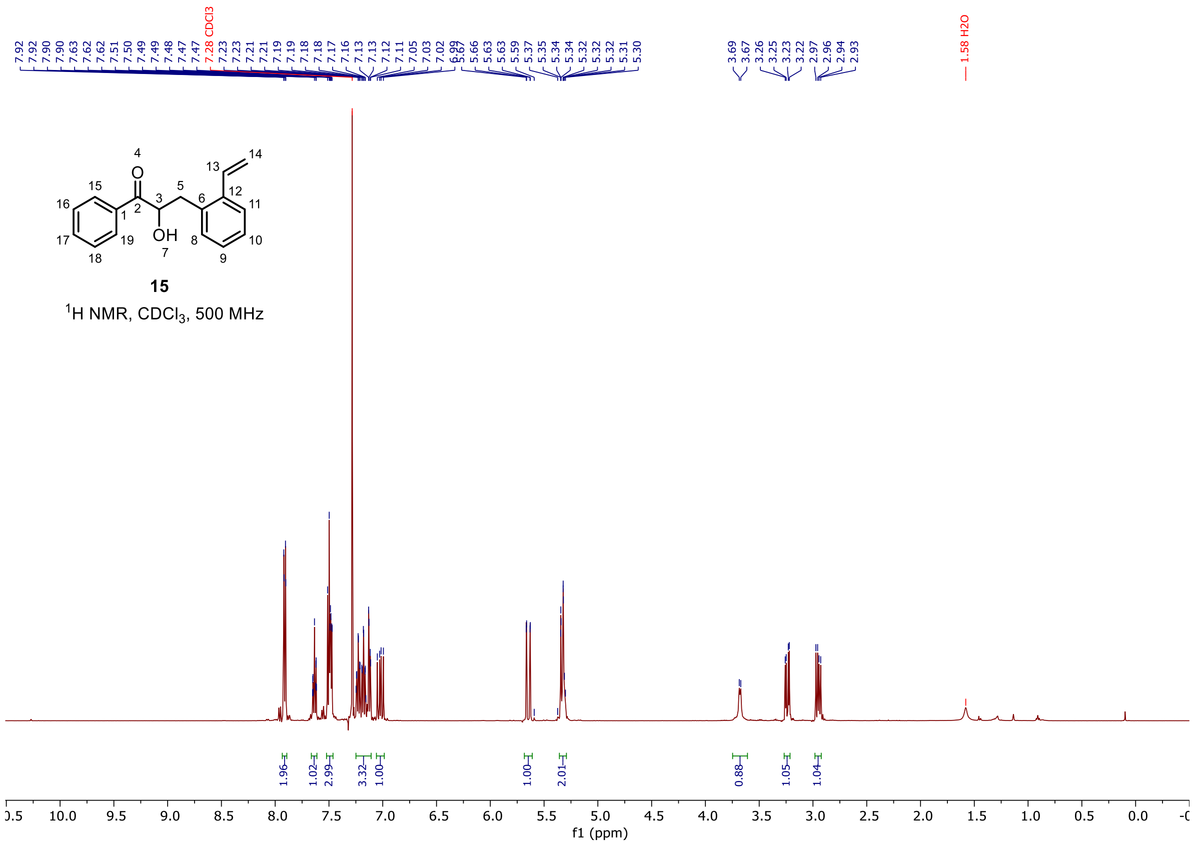

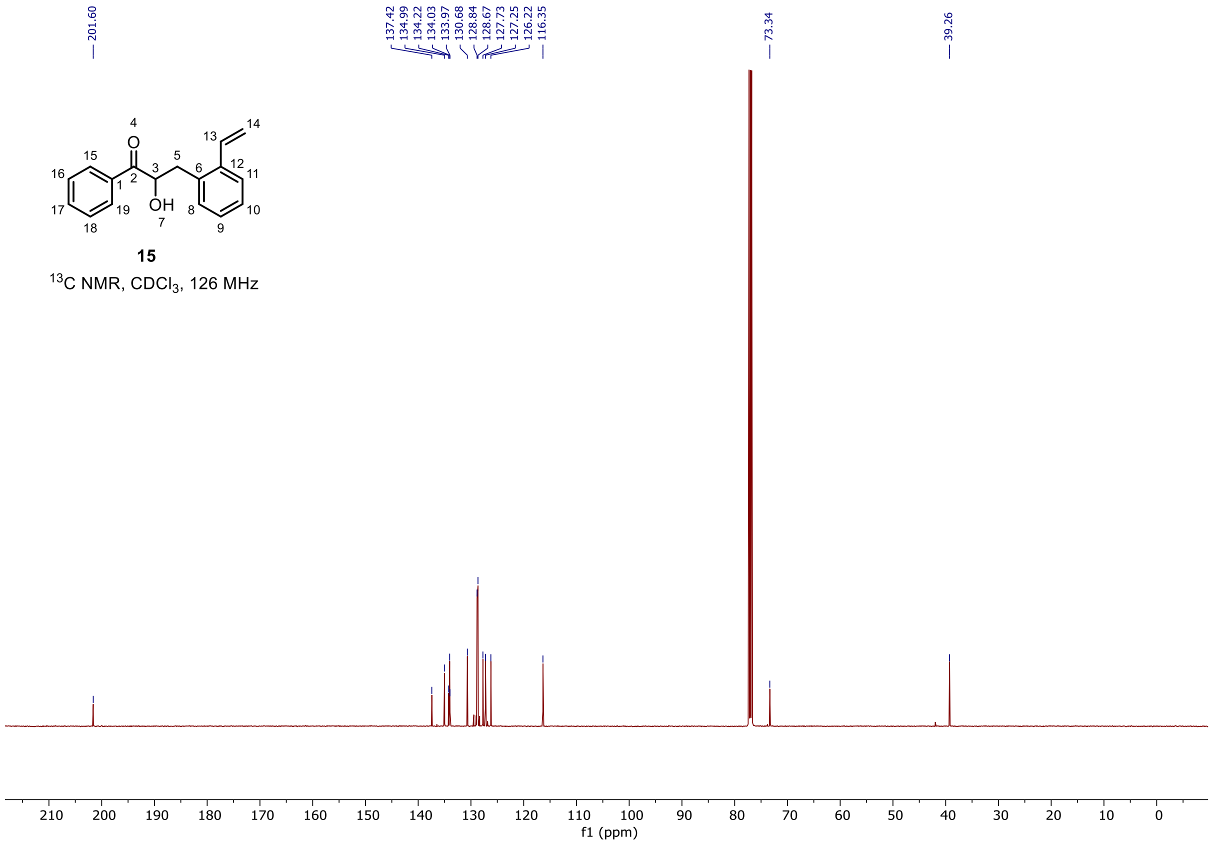

3-(2-Allylphenyl)-2-hydroxy-1-phenylpropan-1-one 11

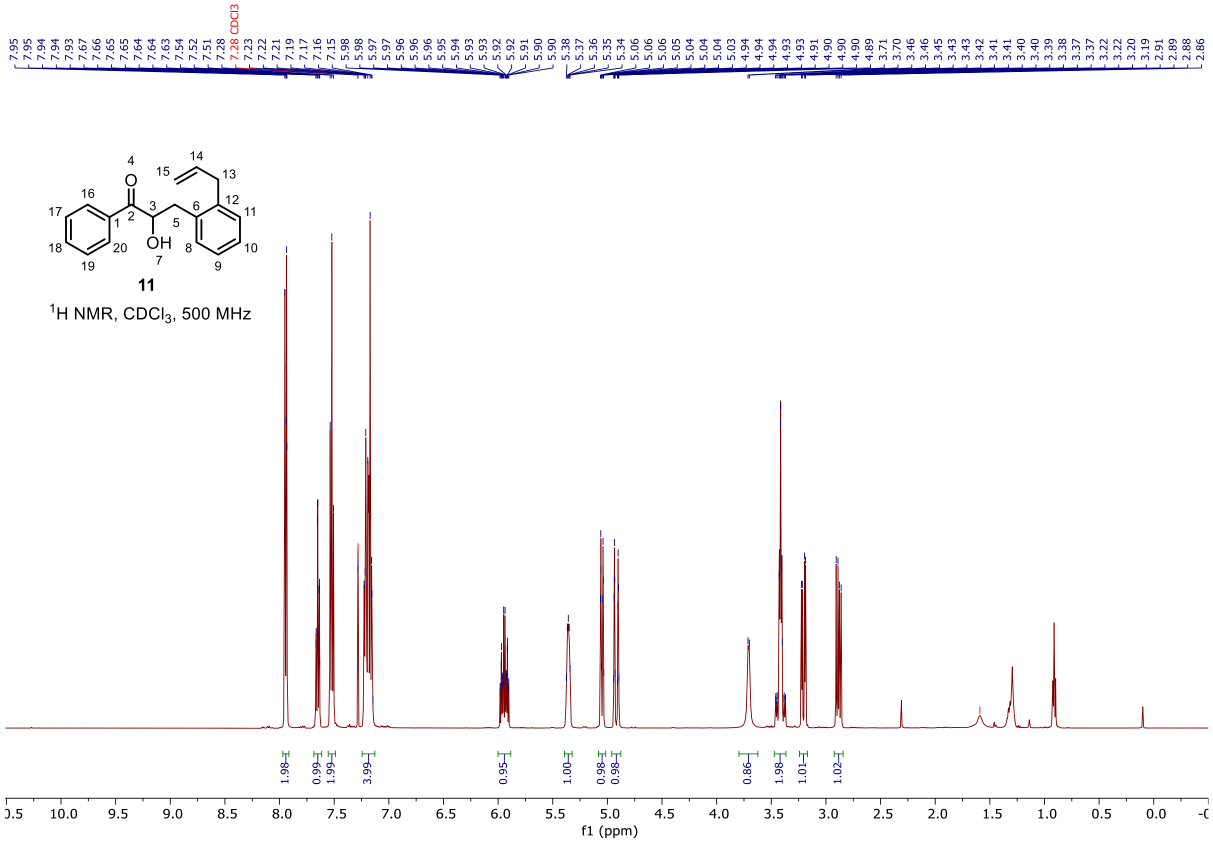

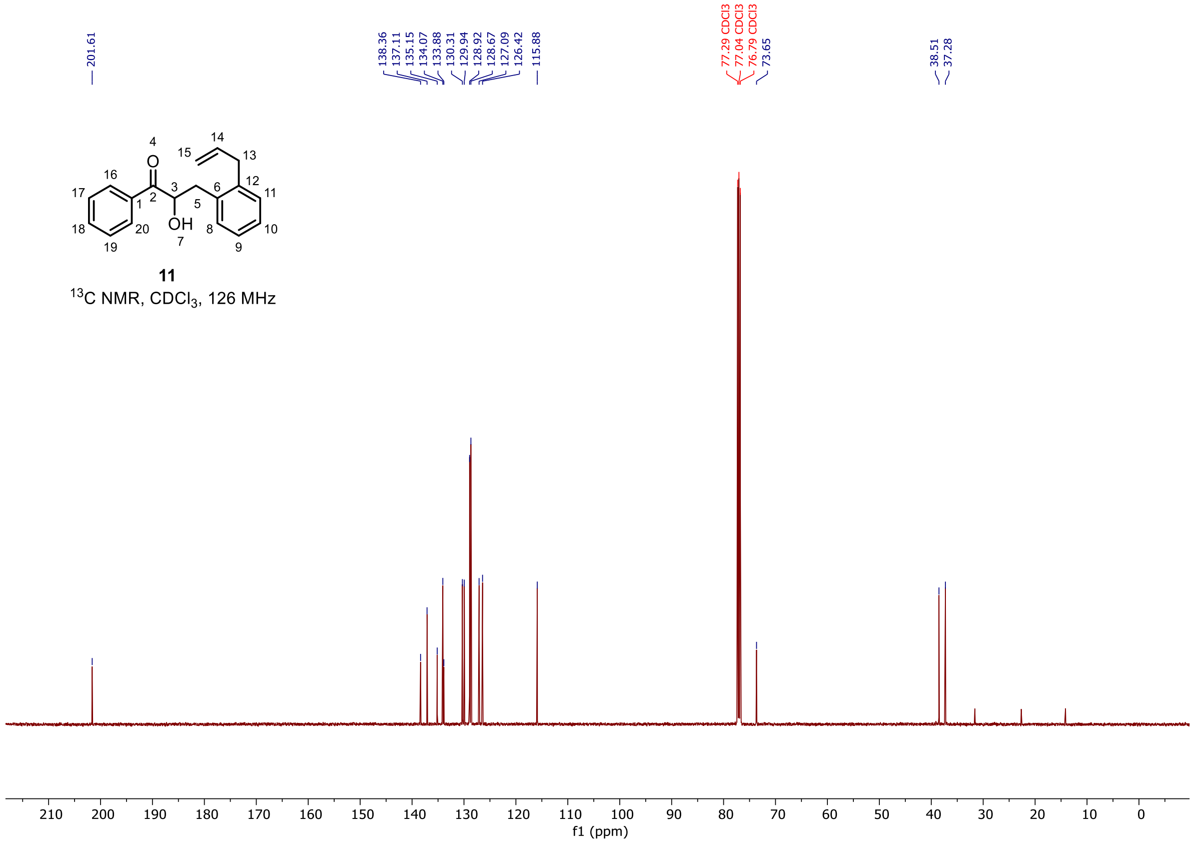

2-Hydroxy-1-(2-vinylphenyl)ethan-1-one s20

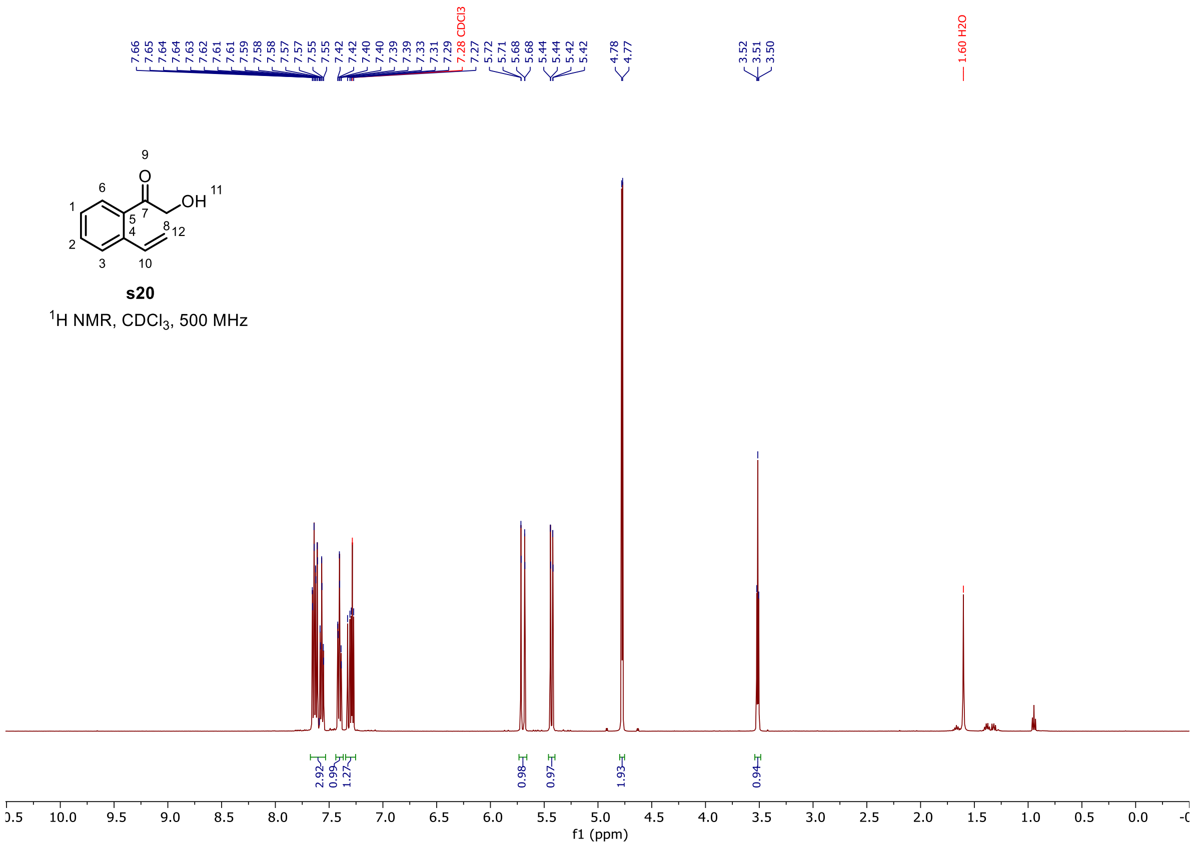

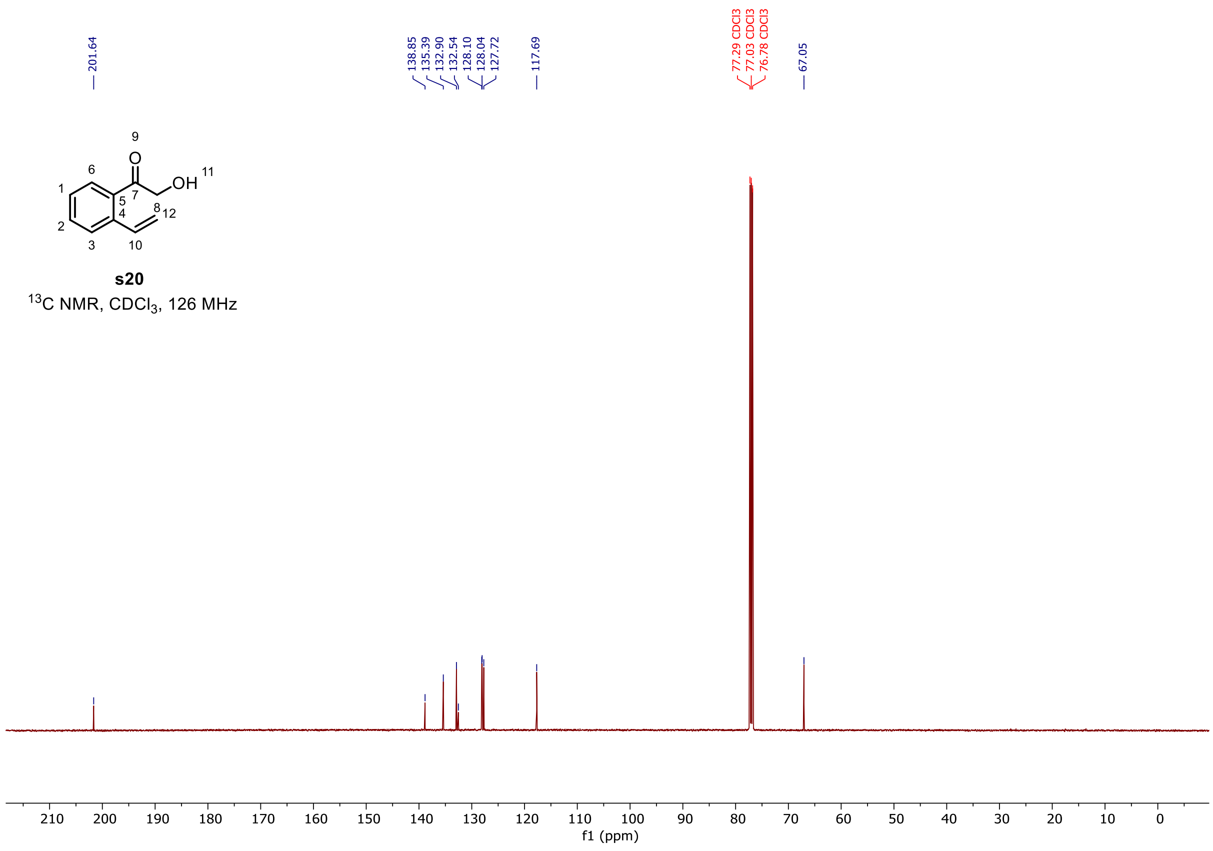

1-(2-Allylphenyl)-2-hydroxyethan-1-one 13

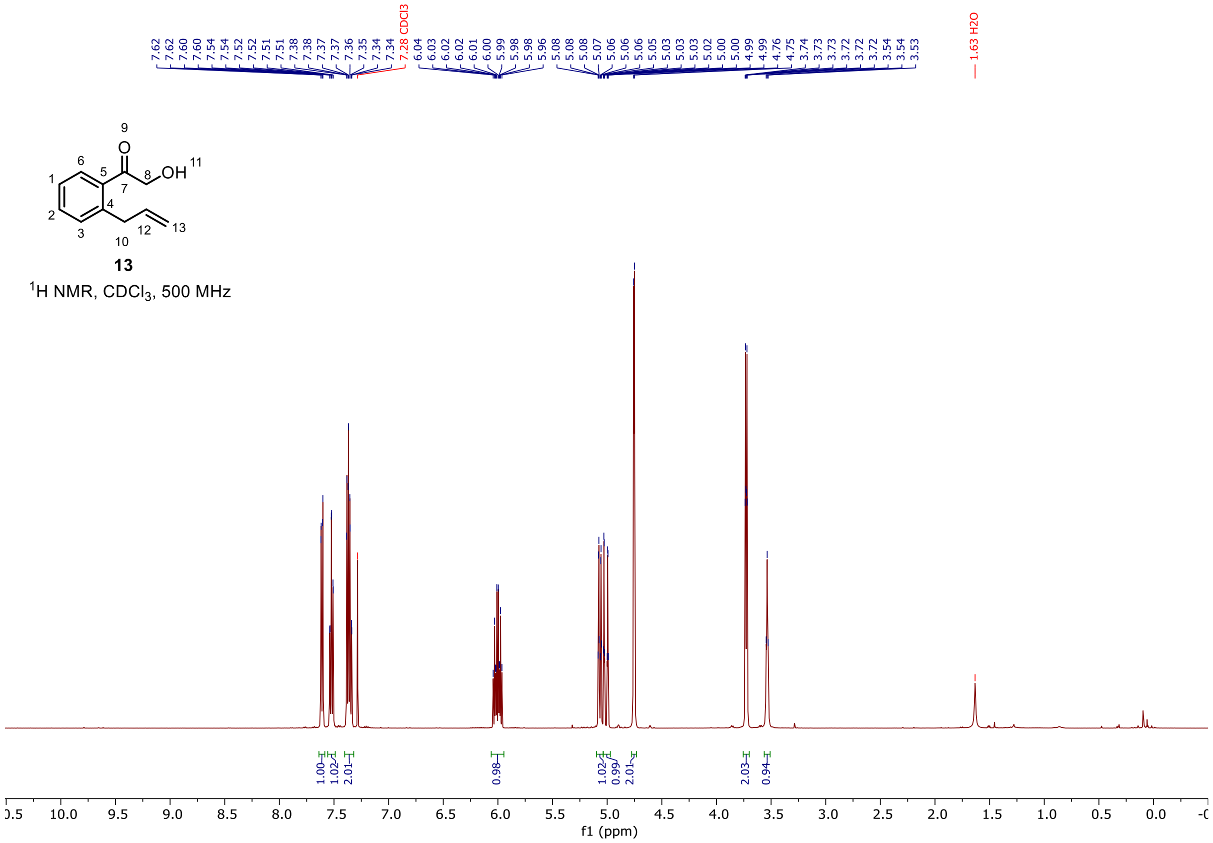

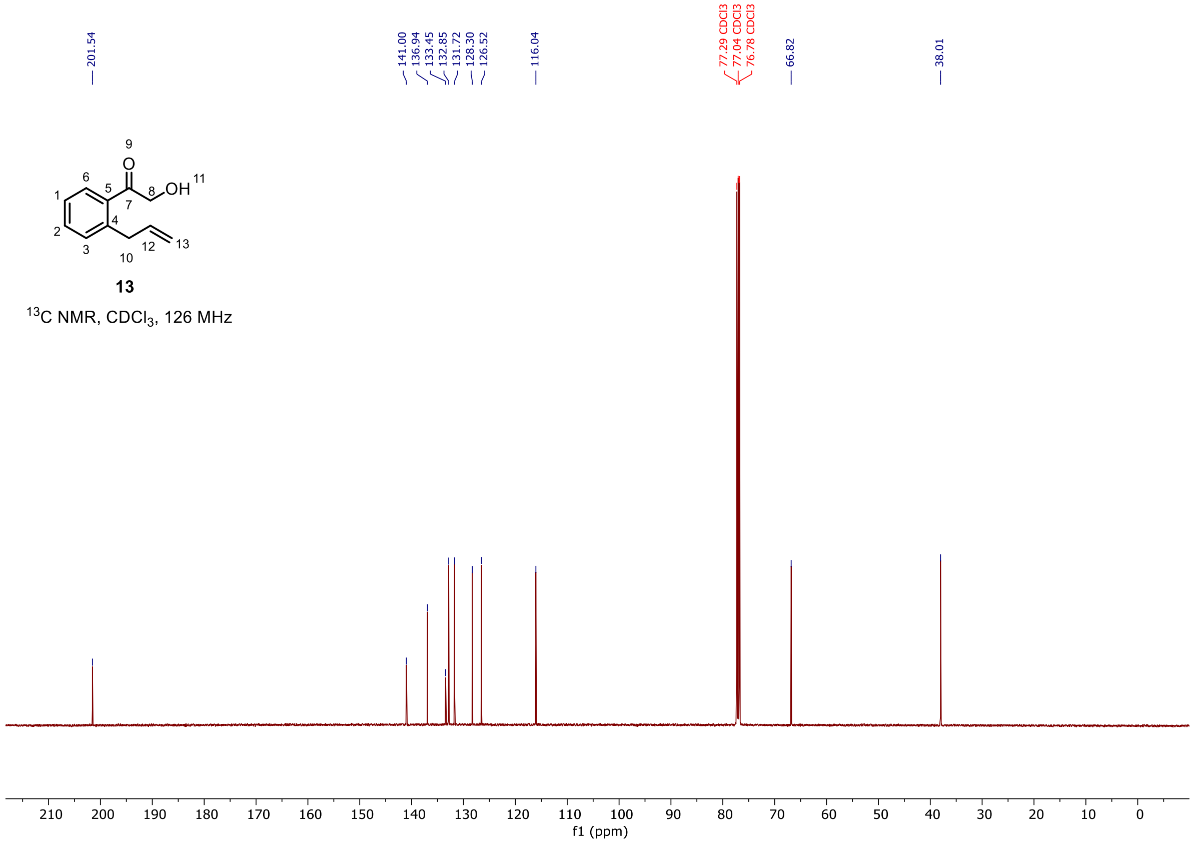

3-(Allyloxy)-2-hydroxy-1-phenylpropan-1-one 5k

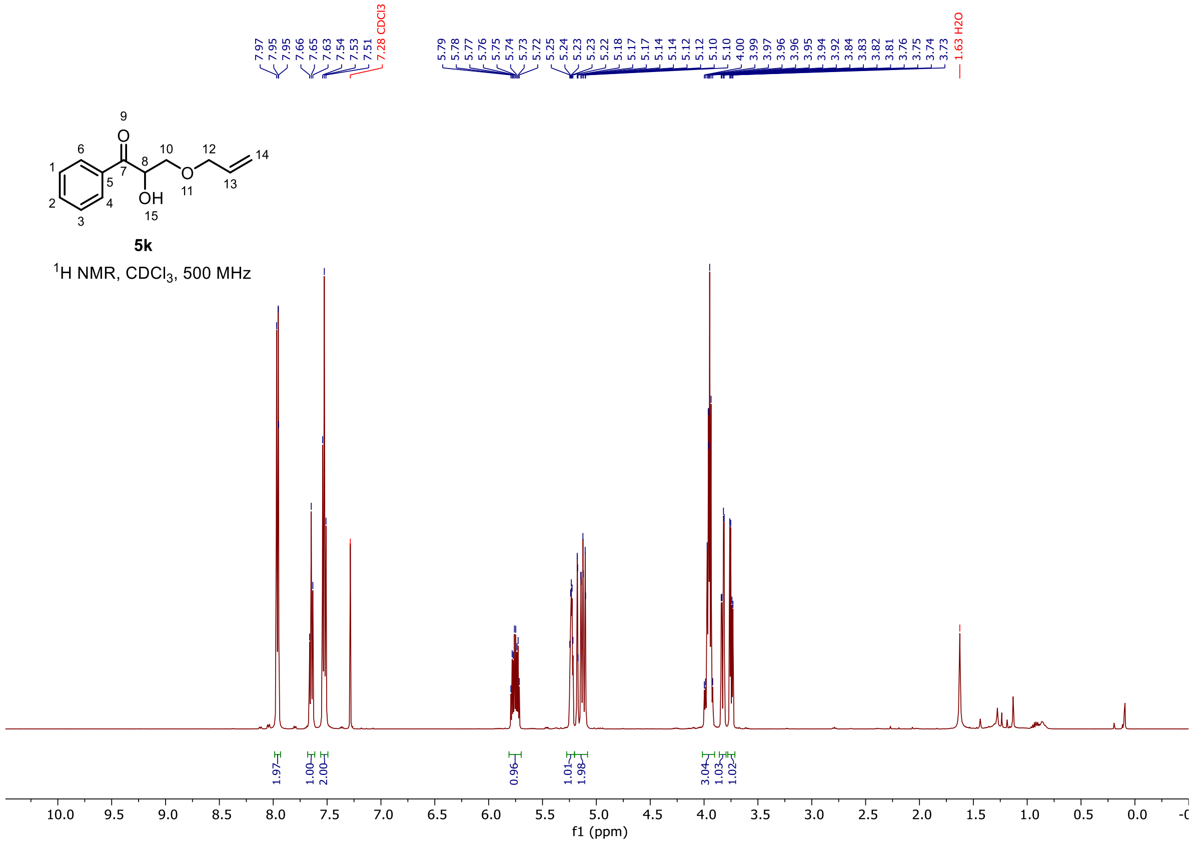

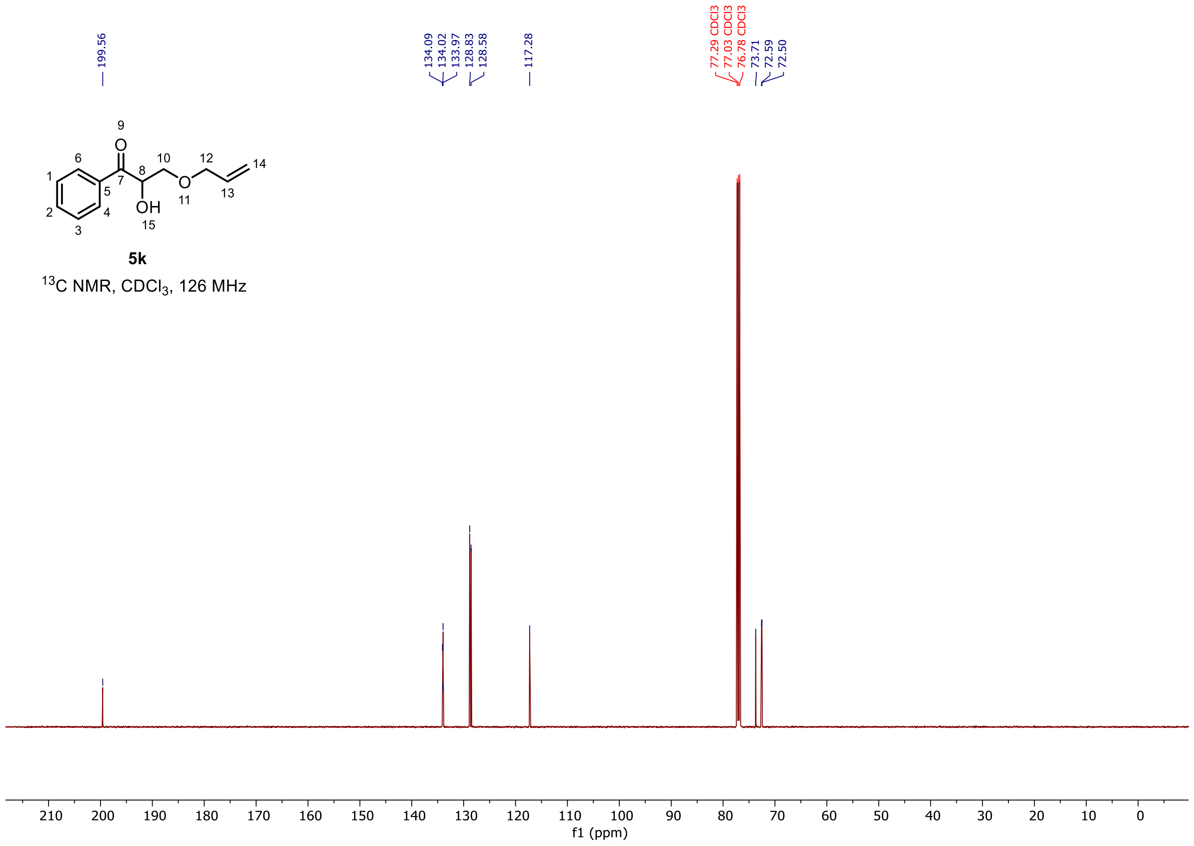

3-(But-3-en-1-yloxy)-1-phenylpropan-1-one 7k-s24

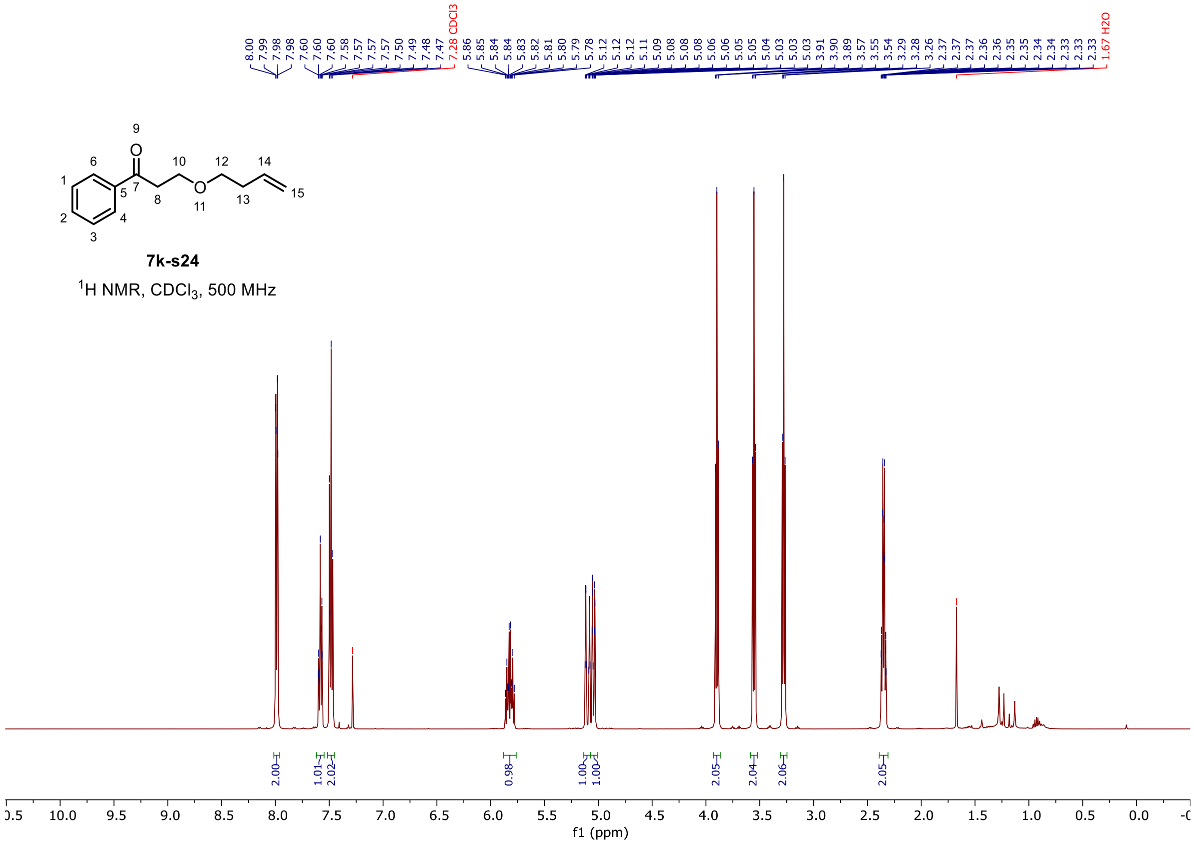

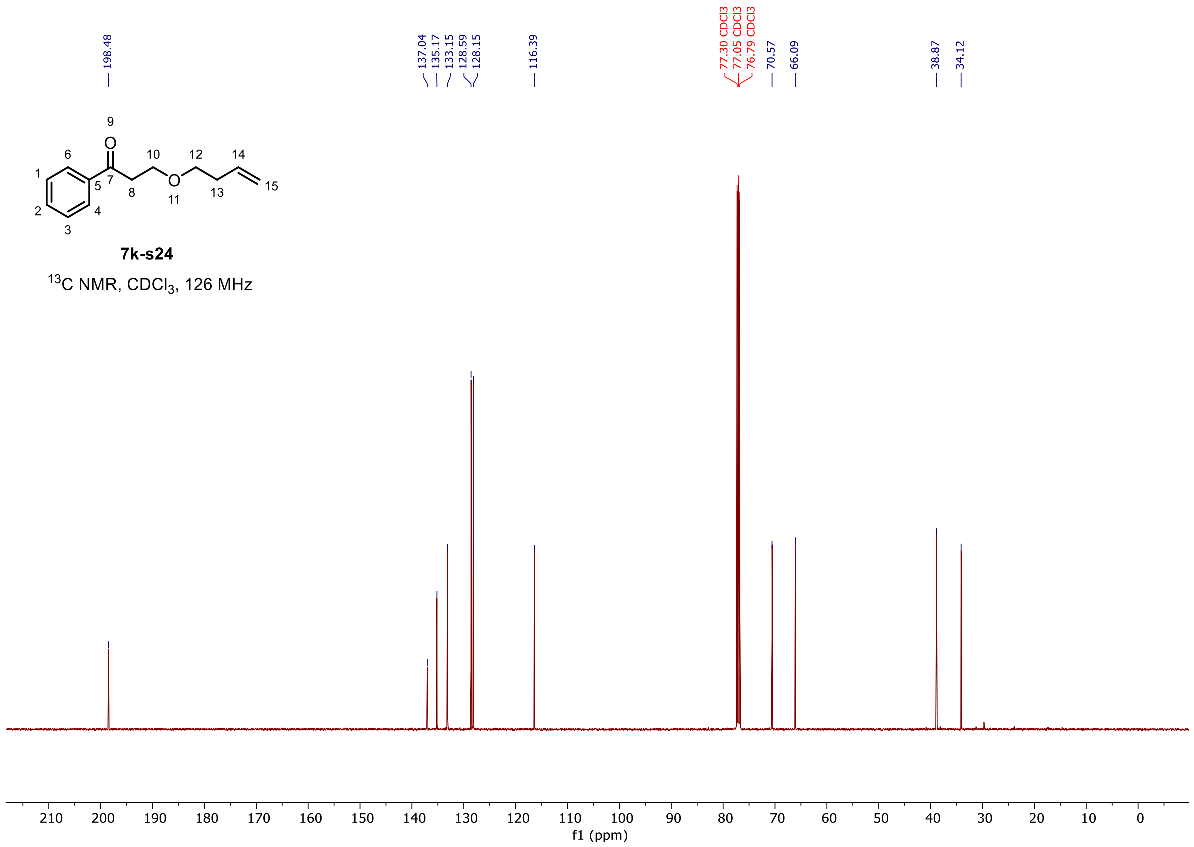

3-(But-3-en-1-yloxy)-2-hydroxy-1-phenylpropan-1-one 7k

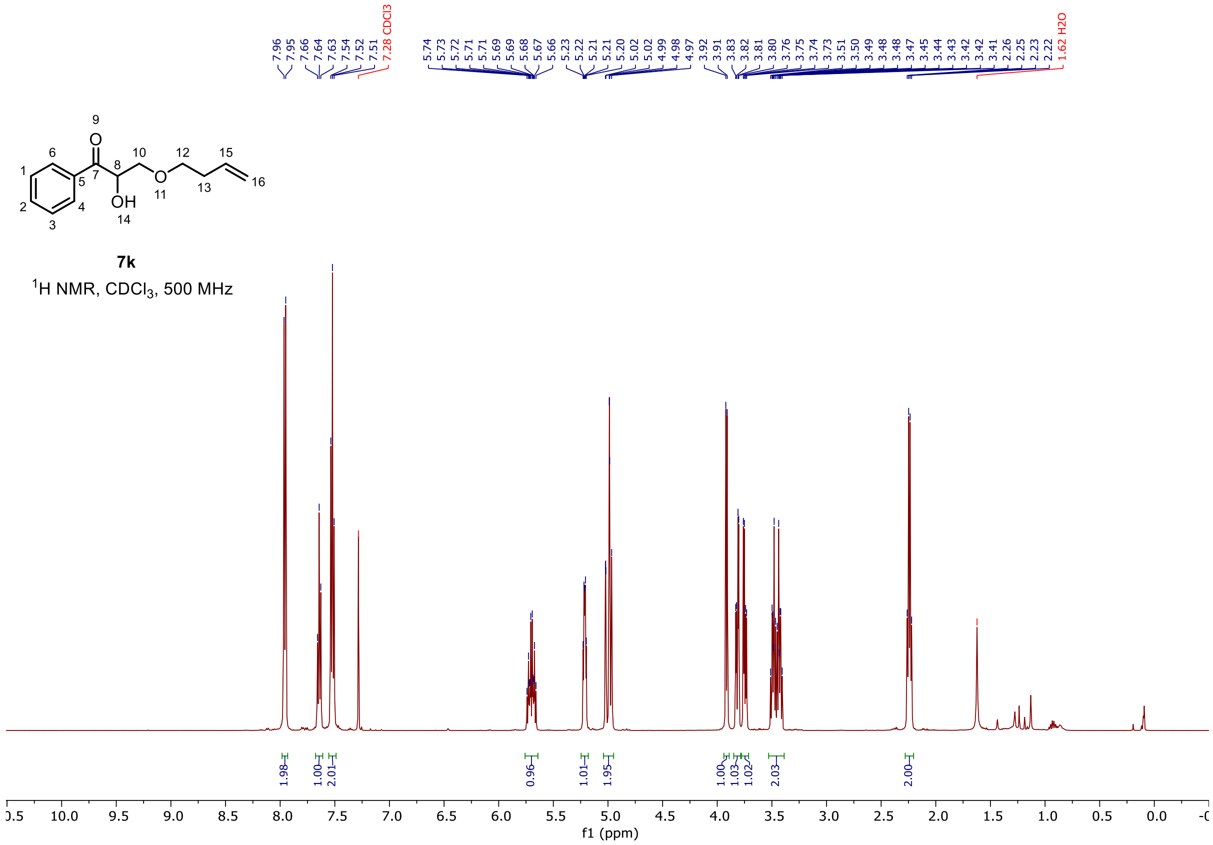

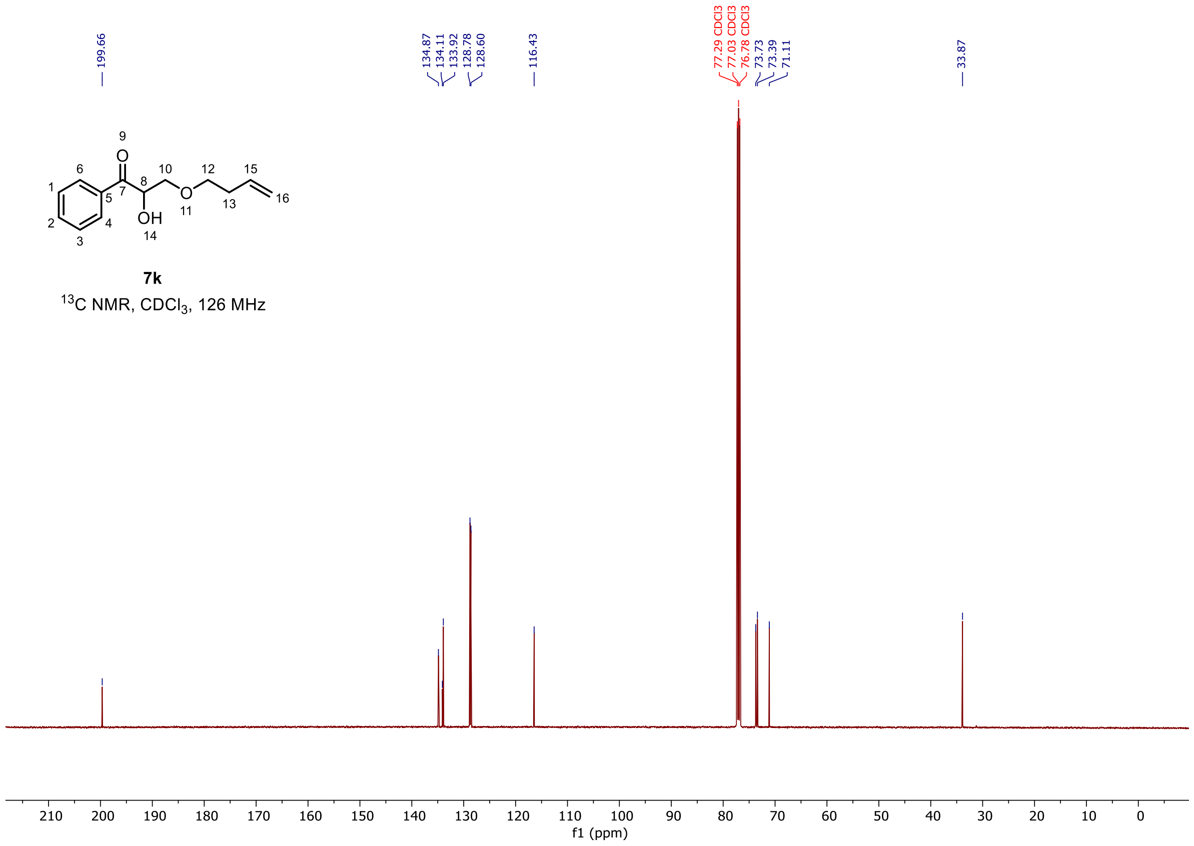

1-Hydroxy-1-phenylhept-6-en-2-one *iso*-5

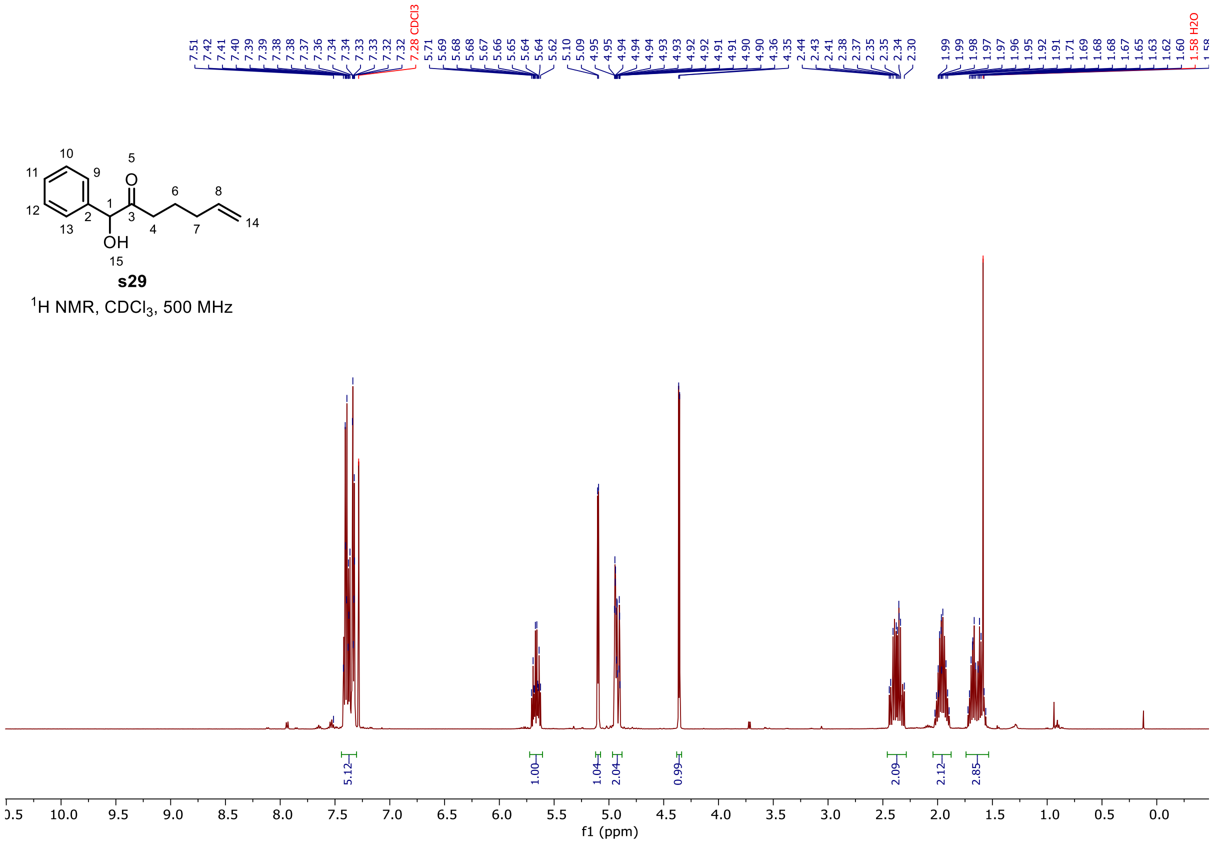

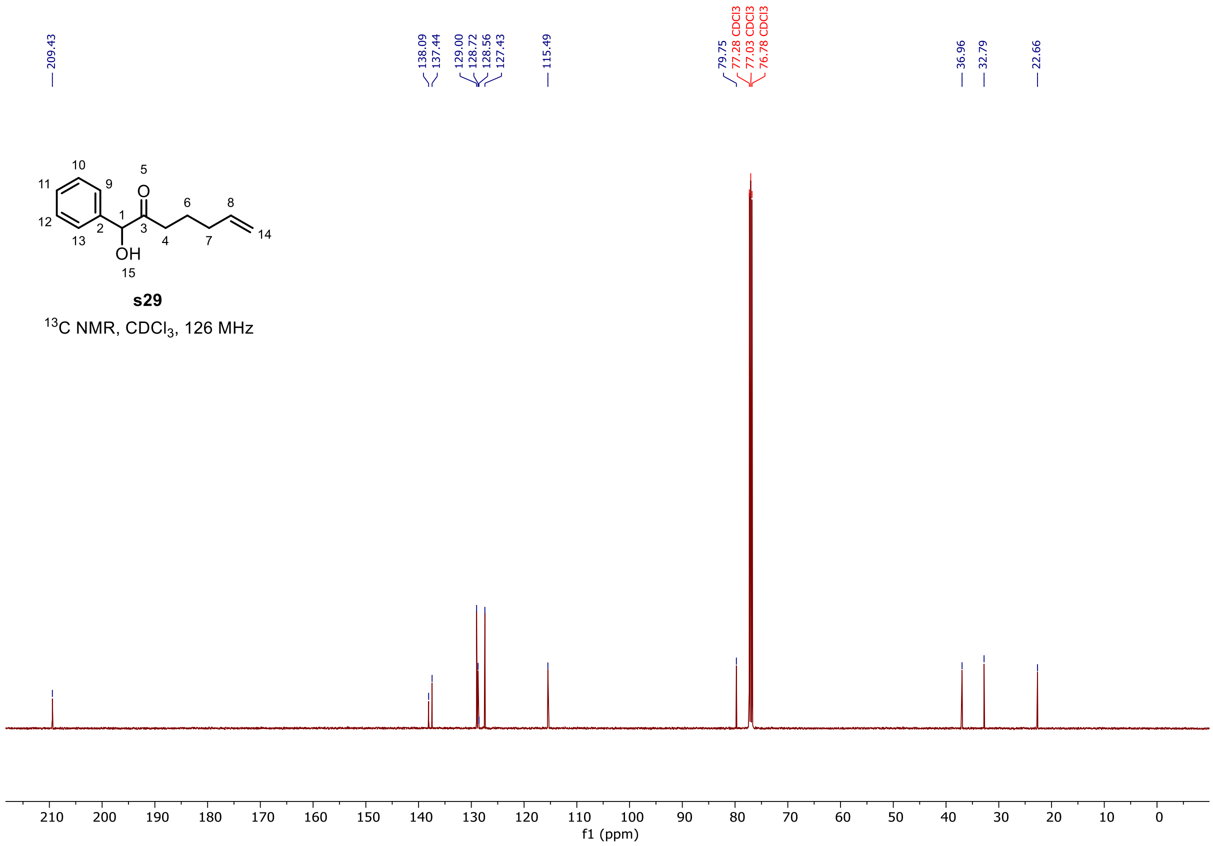

(*E*)-1-Hydroxy-1-phenyloct-6-en-2-one s30

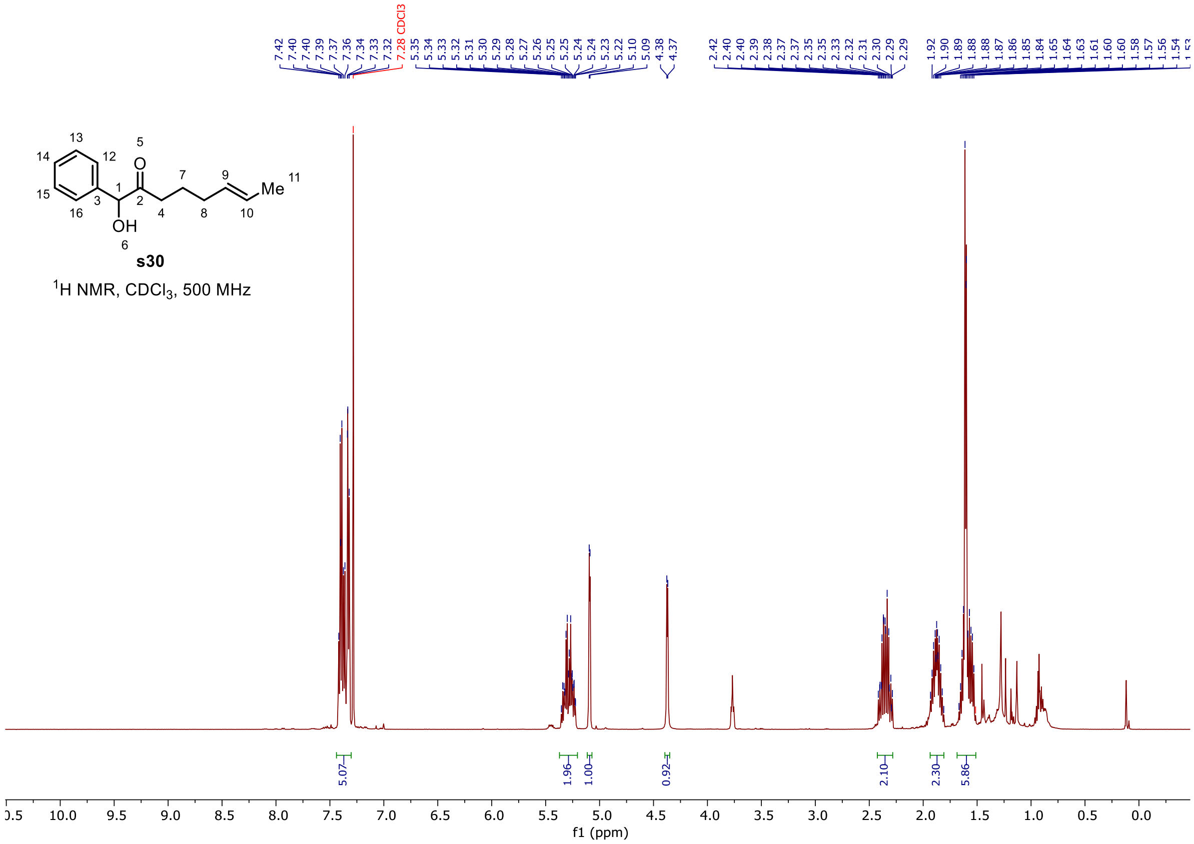

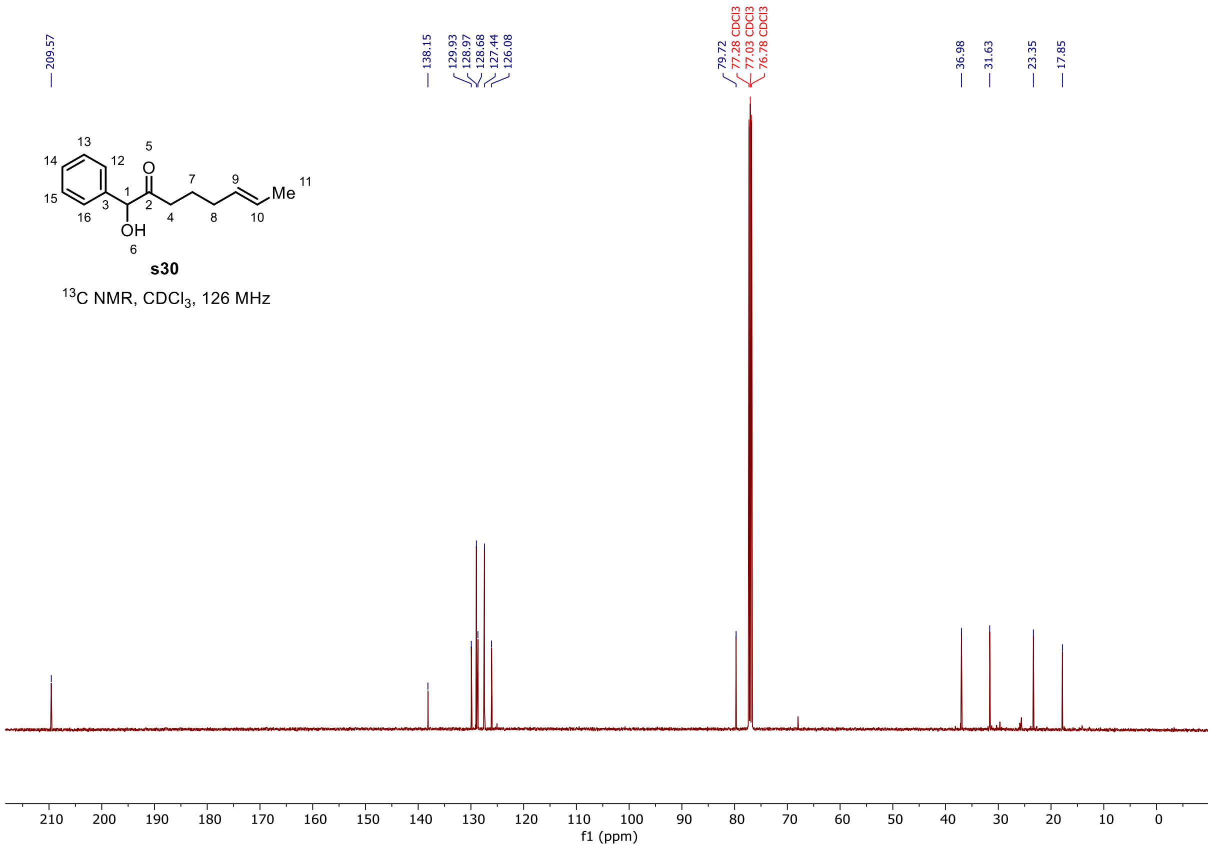

1-oxo-1-Phenyloct-7-en-2-yl acetate Ac-7a

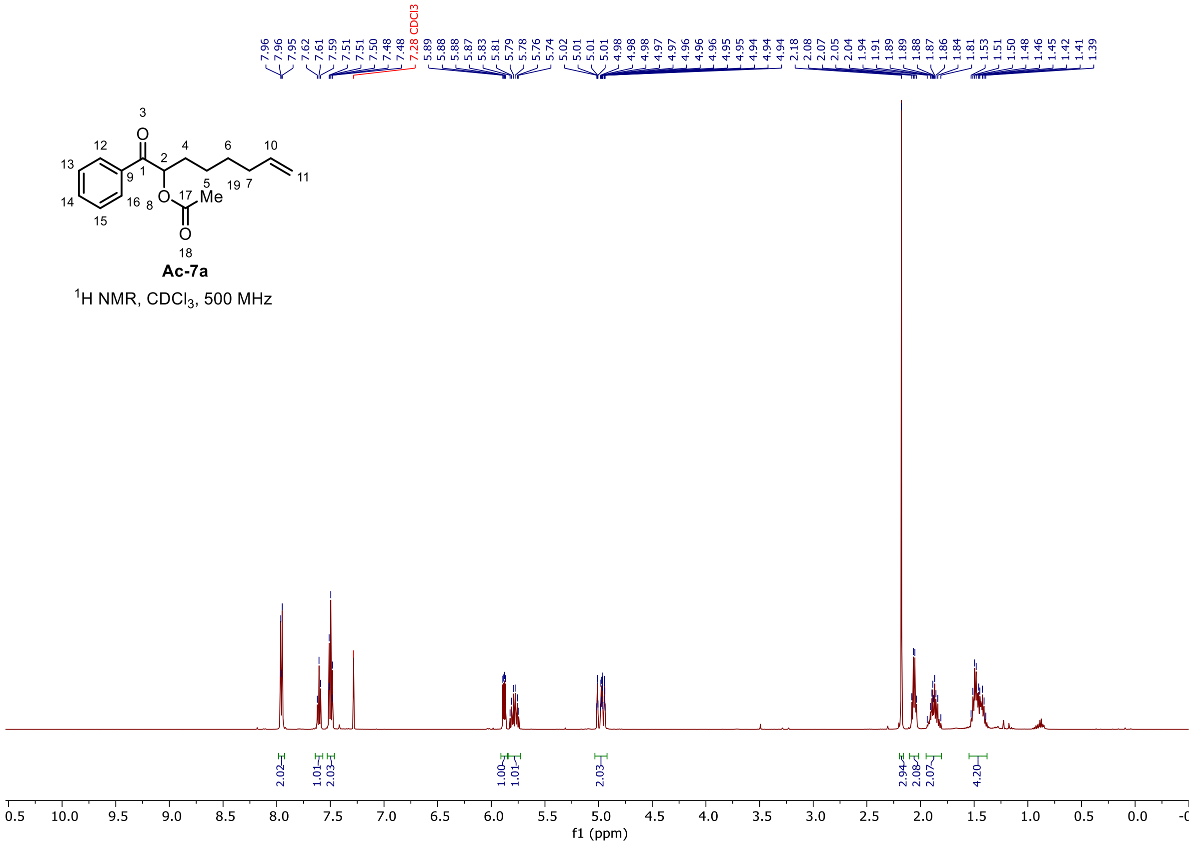

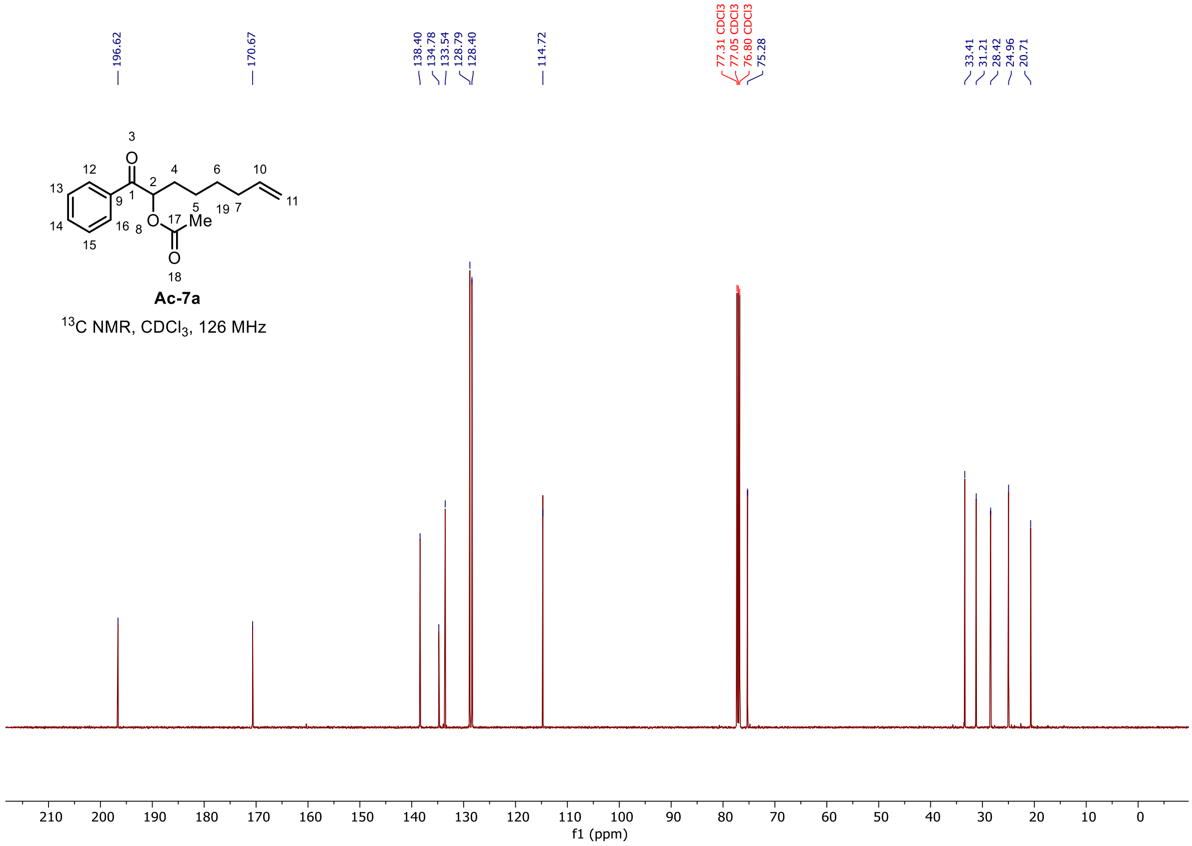

Products of Catalysis

(*trans*)-2-Hydroxy-6-methyl-2-phenylcyclohexan-1-one *iso*-6a

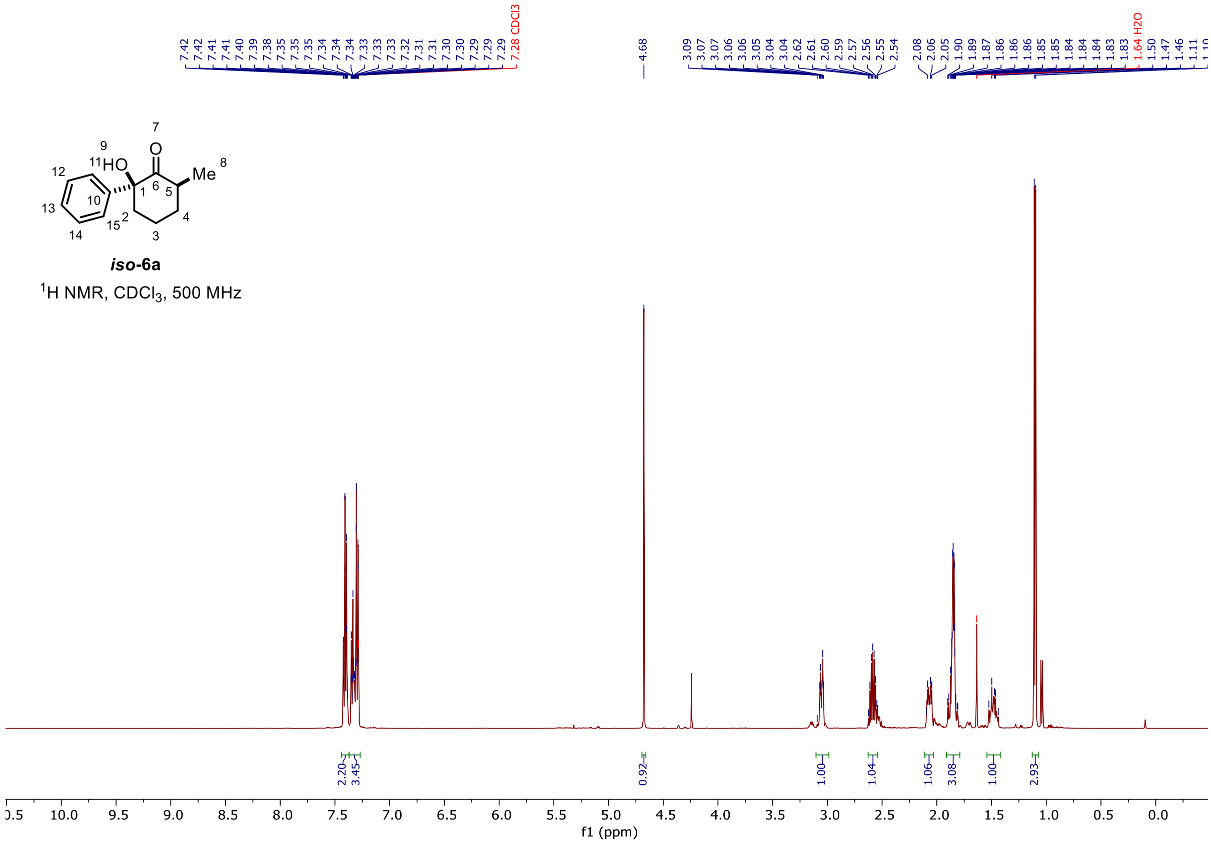

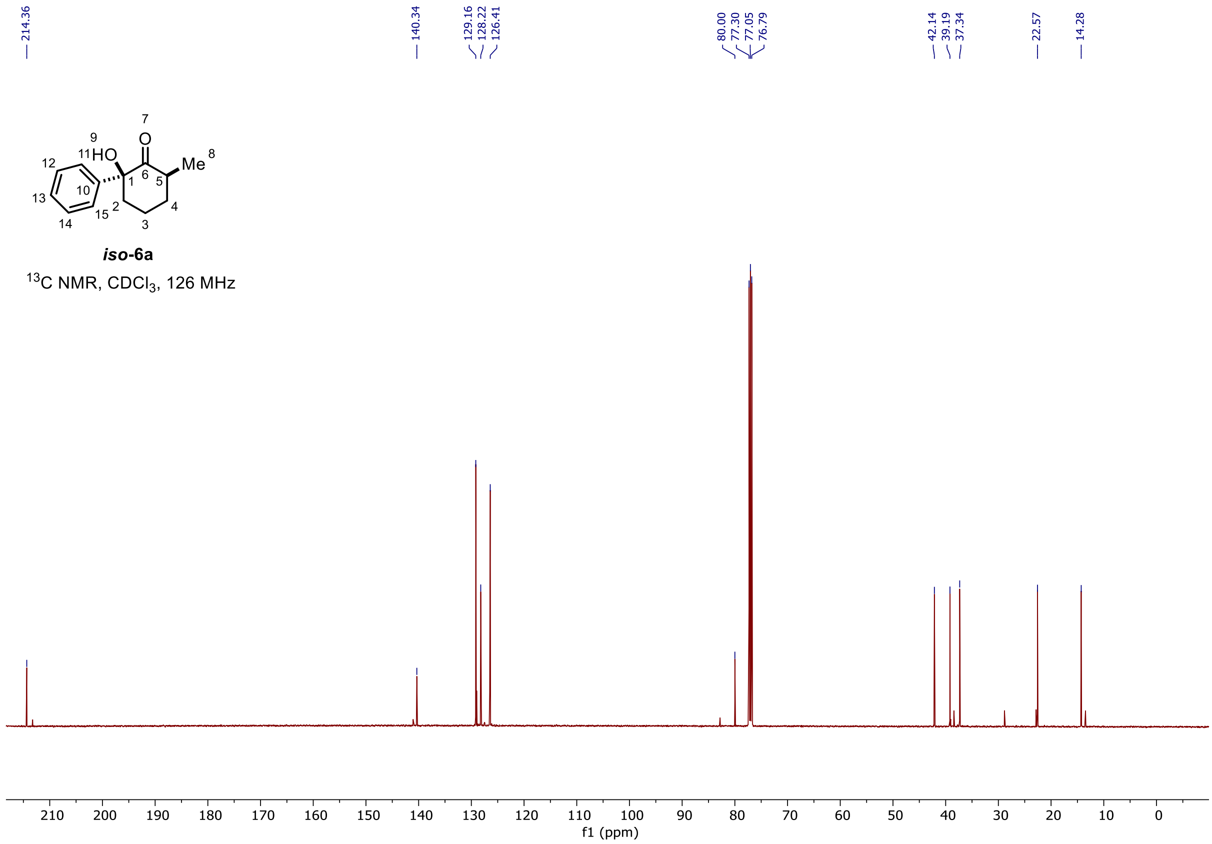

(*trans*)-2-Hydroxy-6-methyl-2-(p-tolyl)cyclohexan-1-one *iso*-6b

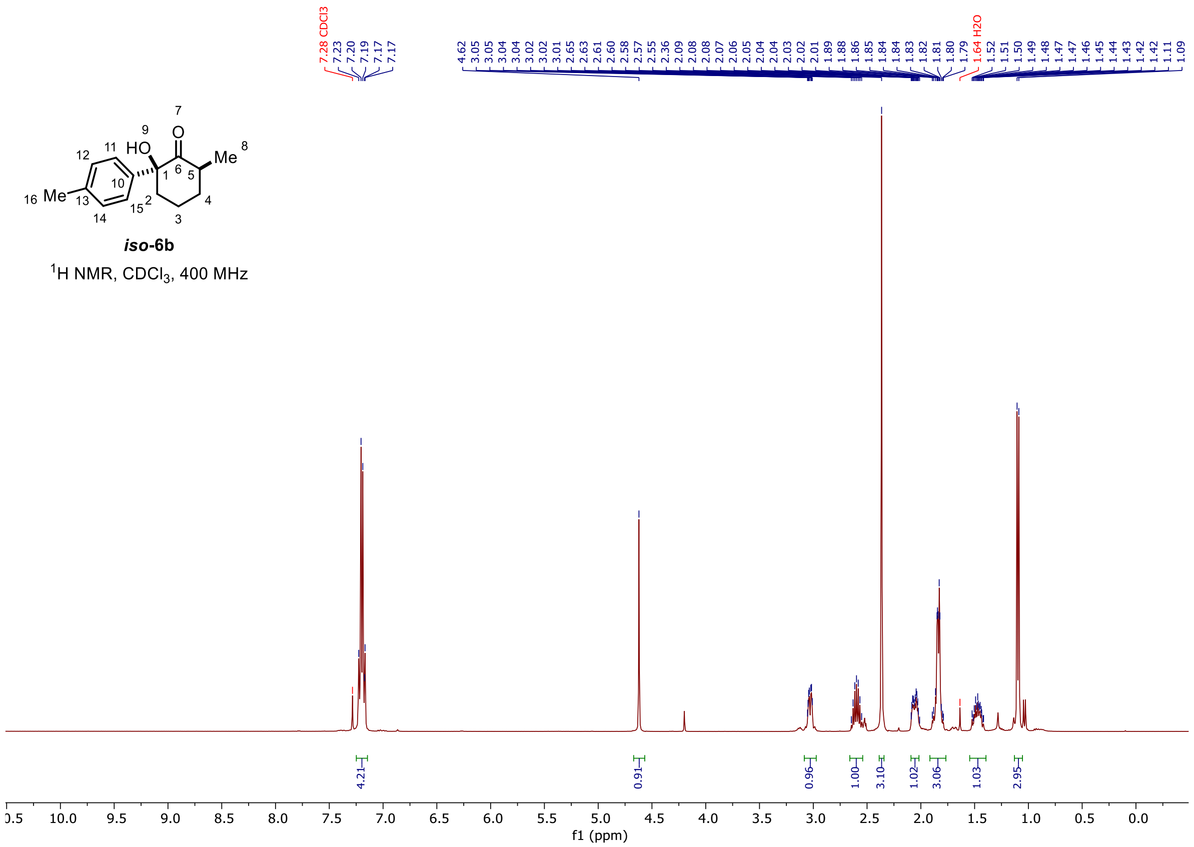

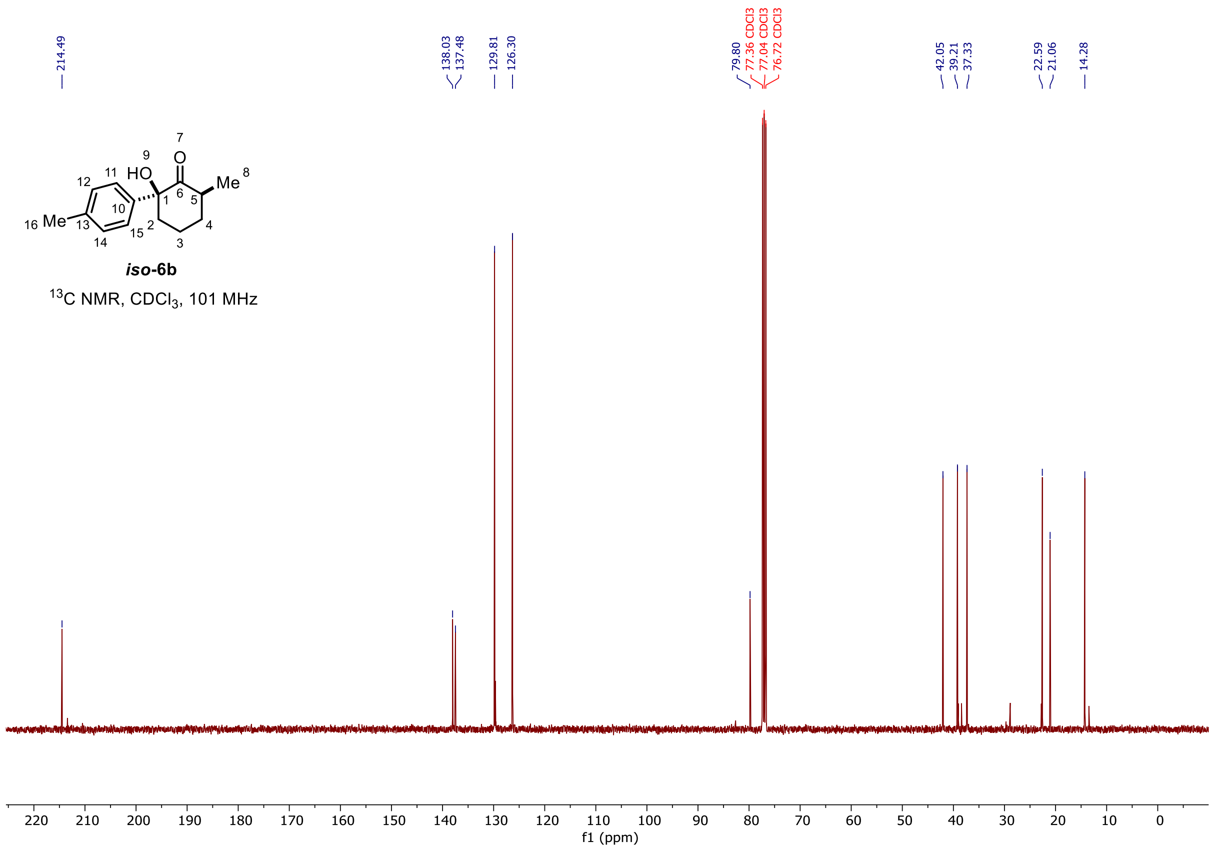

(*trans*)-2-(4-Chlorophenyl)-2-hydroxy-6-methylcyclohexan-1-one *iso*-6c and (*trans)*-2-(4-Chlorophenyl)-2-hydroxy-3-methylcyclohexan-1-one 6c

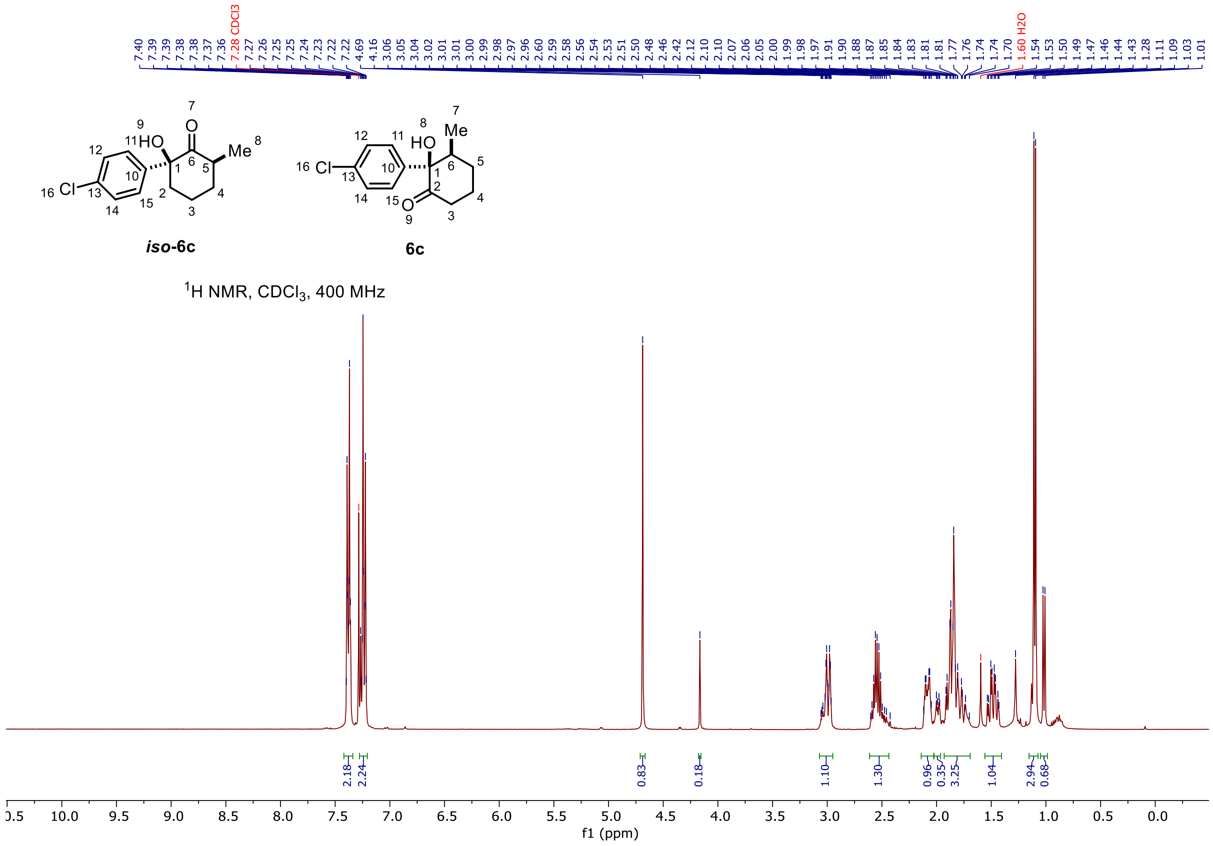

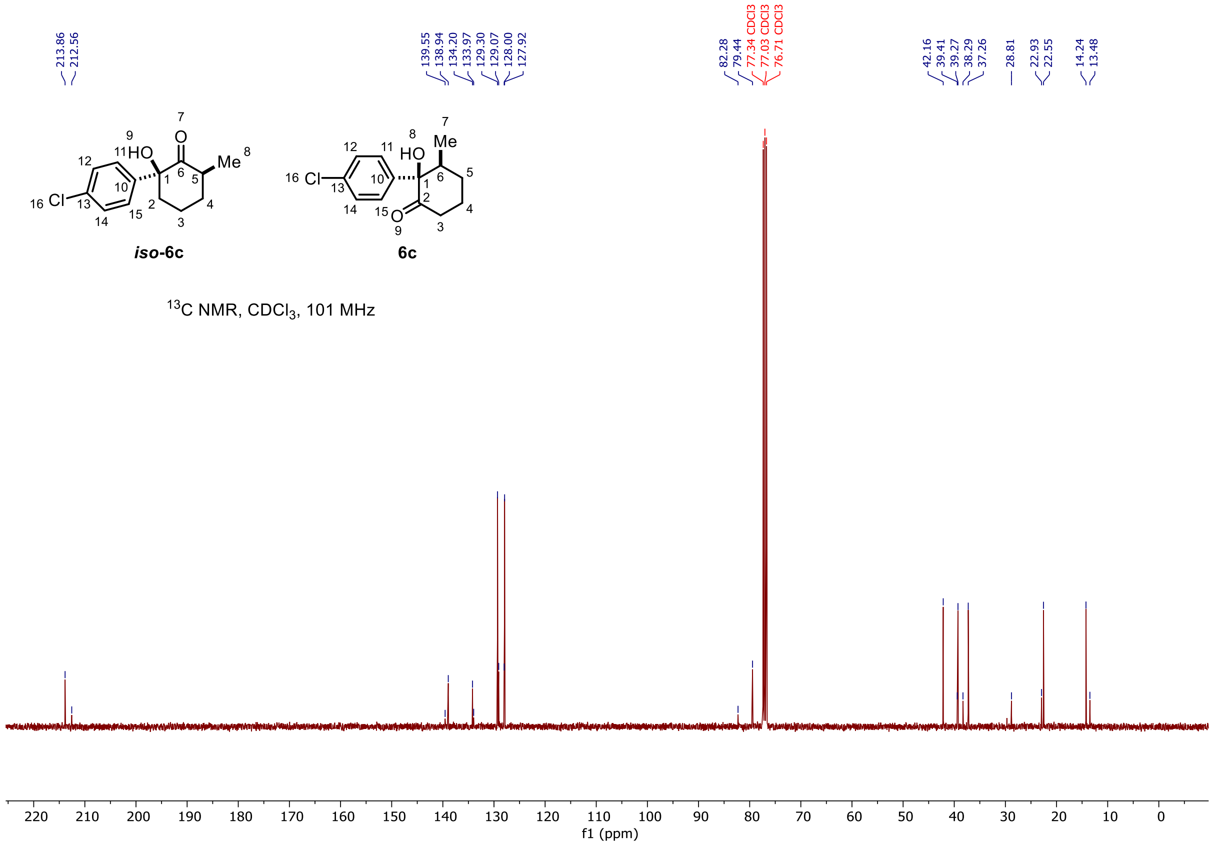

***(trans)*-2-(2-Fluorophenyl)-2-hydroxy-6-methylcyclohexan-1-one *iso*-6d**

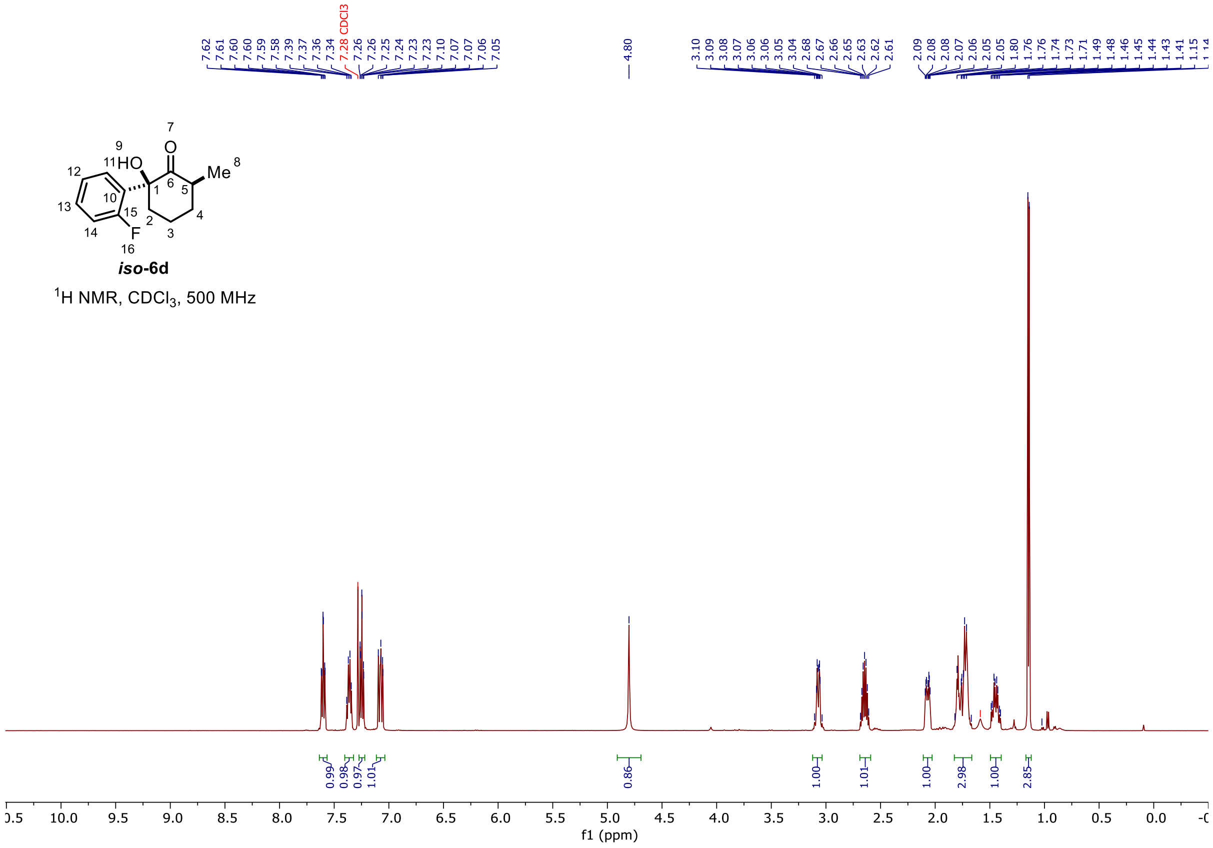

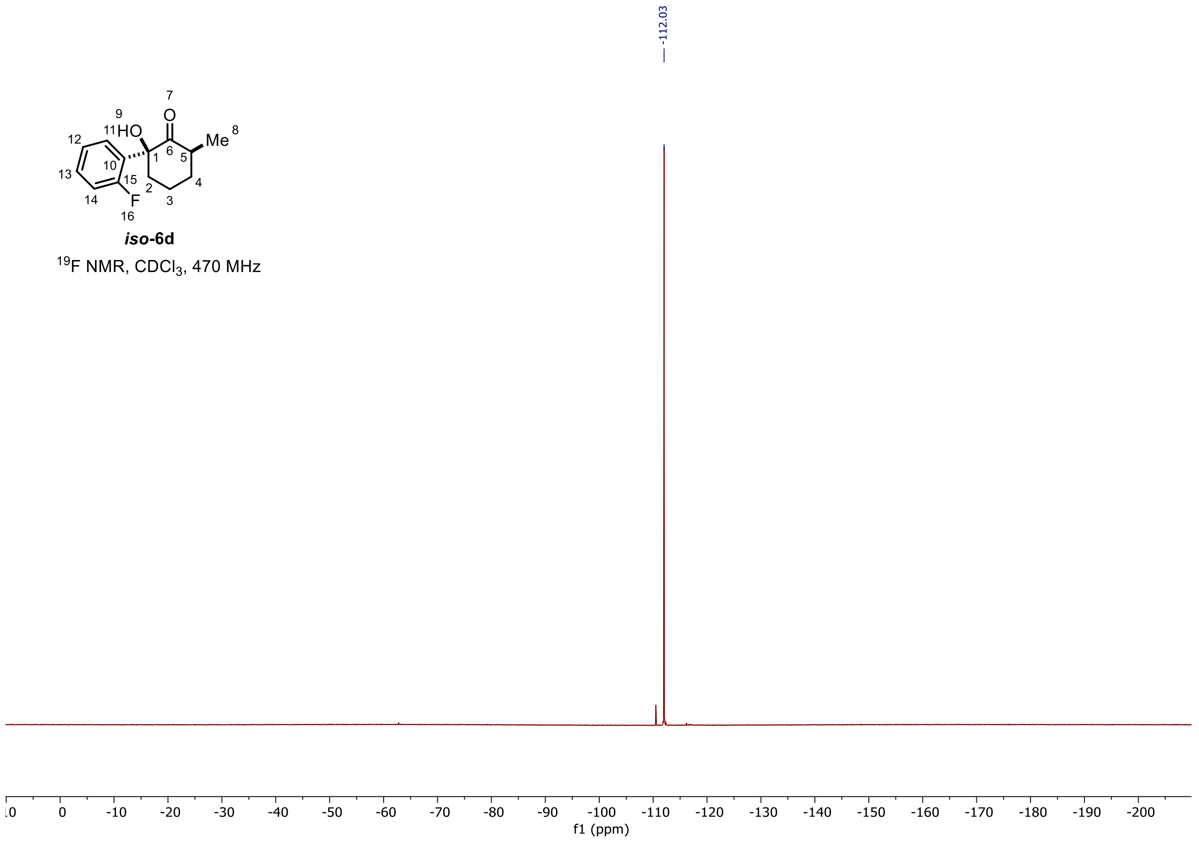

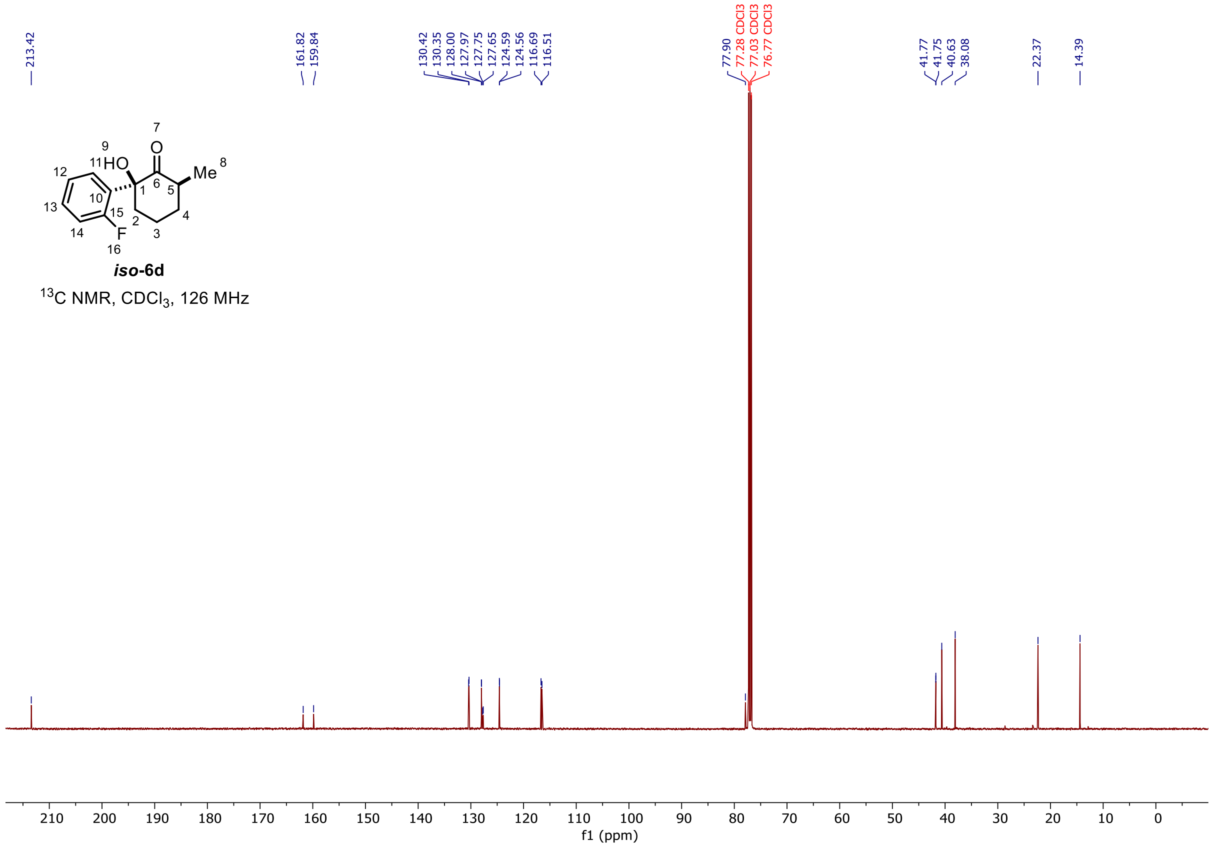

213.42
161.82
159.84
130.42
130.35
128.00
127.97
127.75
127.65
124.59
124.56
116.69
116.51
77.90
77.28 CDCl3
77.03 CDCl3
76.77 CDCl3
41.77
41.75
40.63
38.08
22.37
14.39
HO
O
Me
F
iso-6d
13C NMR, CDCl3, 126 MHz
f1 (ppm)

(trans)-2-(2-Fluorophenyl)-2-hydroxy-3-methylcyclohexan-1-one 6d

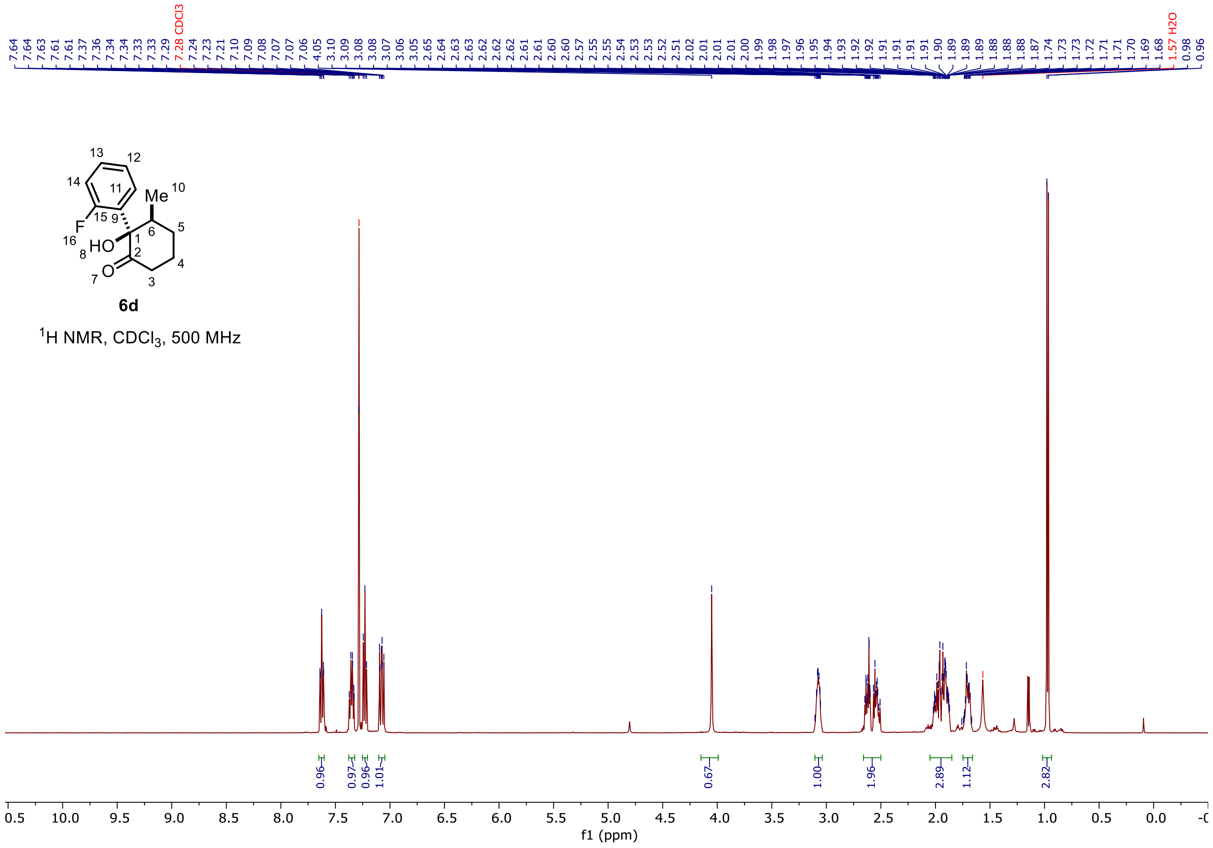

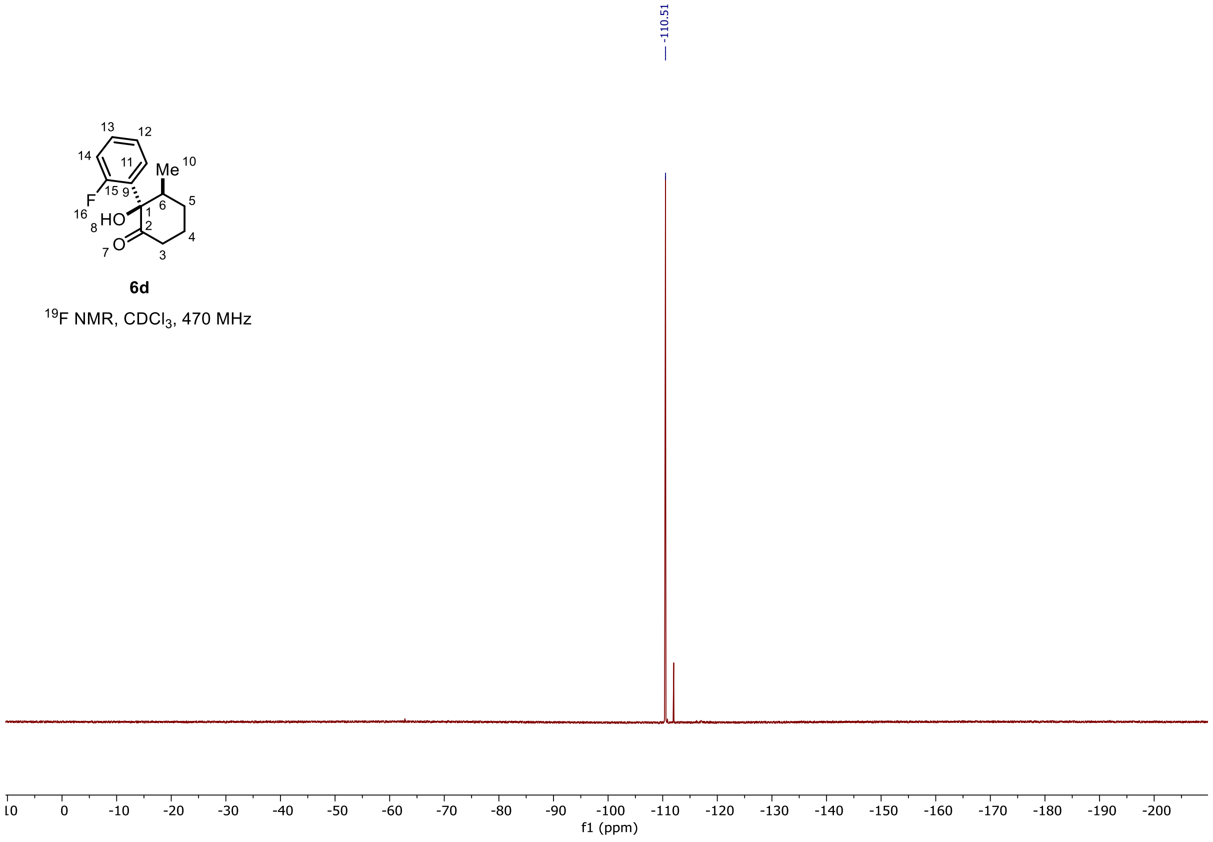

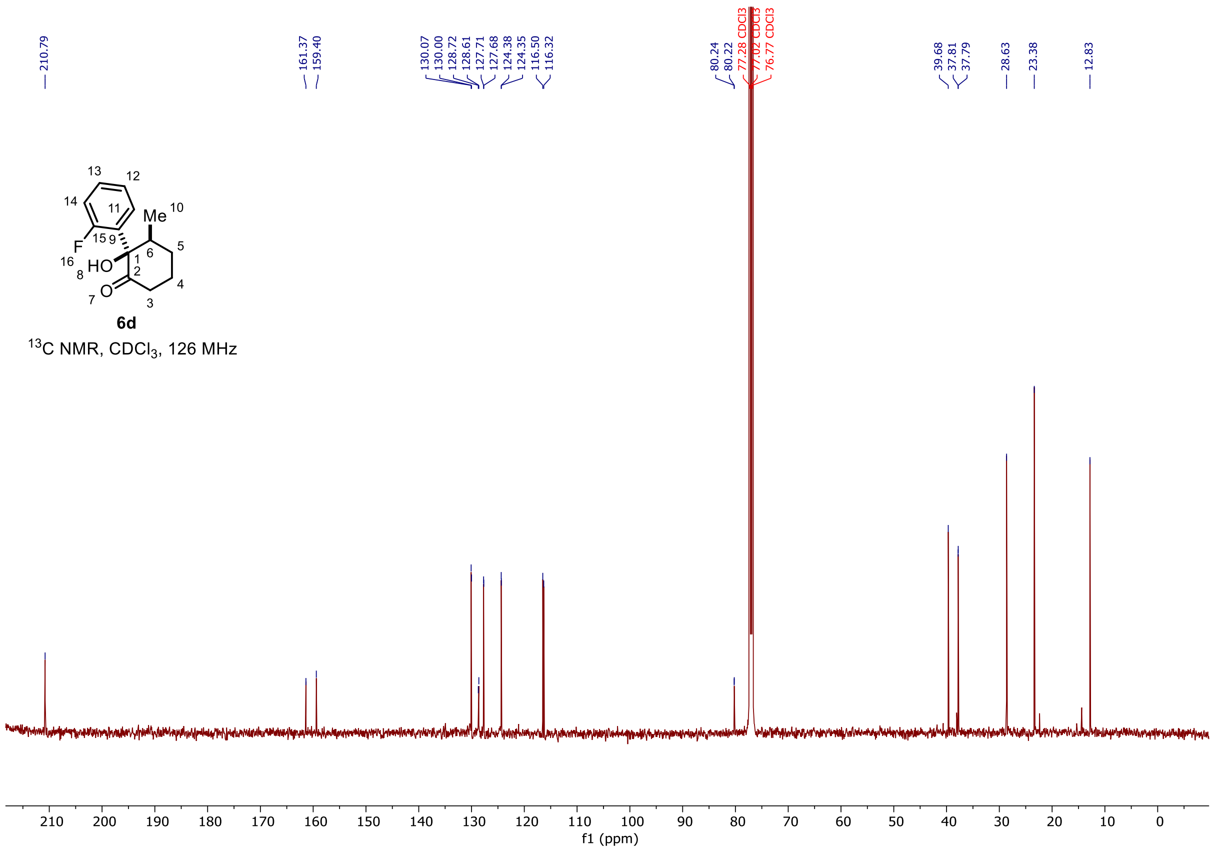

210.79
161.37
159.40
130.07
130.00
128.72
128.61
127.71
127.68
124.38
124.35
116.50
116.32
80.24
80.22
77.28 CDCl3
77.02 CDCl3
76.77 CDCl3
39.68
37.81
37.79
28.63
23.38
12.83
6d
13C NMR, CDCl3, 126 MHz
f1 (ppm)

(trans)-2-Hydroxy-2-(3-methoxyphenyl)-6-methylcyclohexan-1-one iso-6e

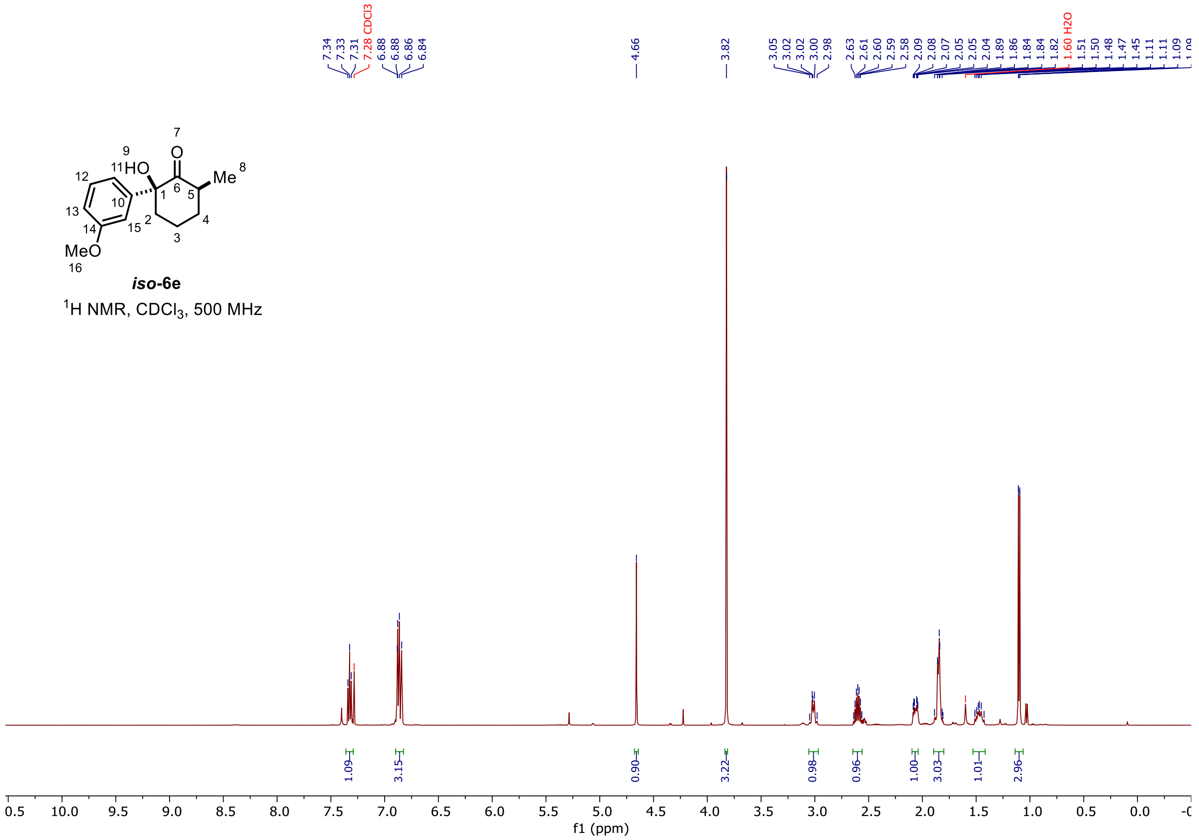

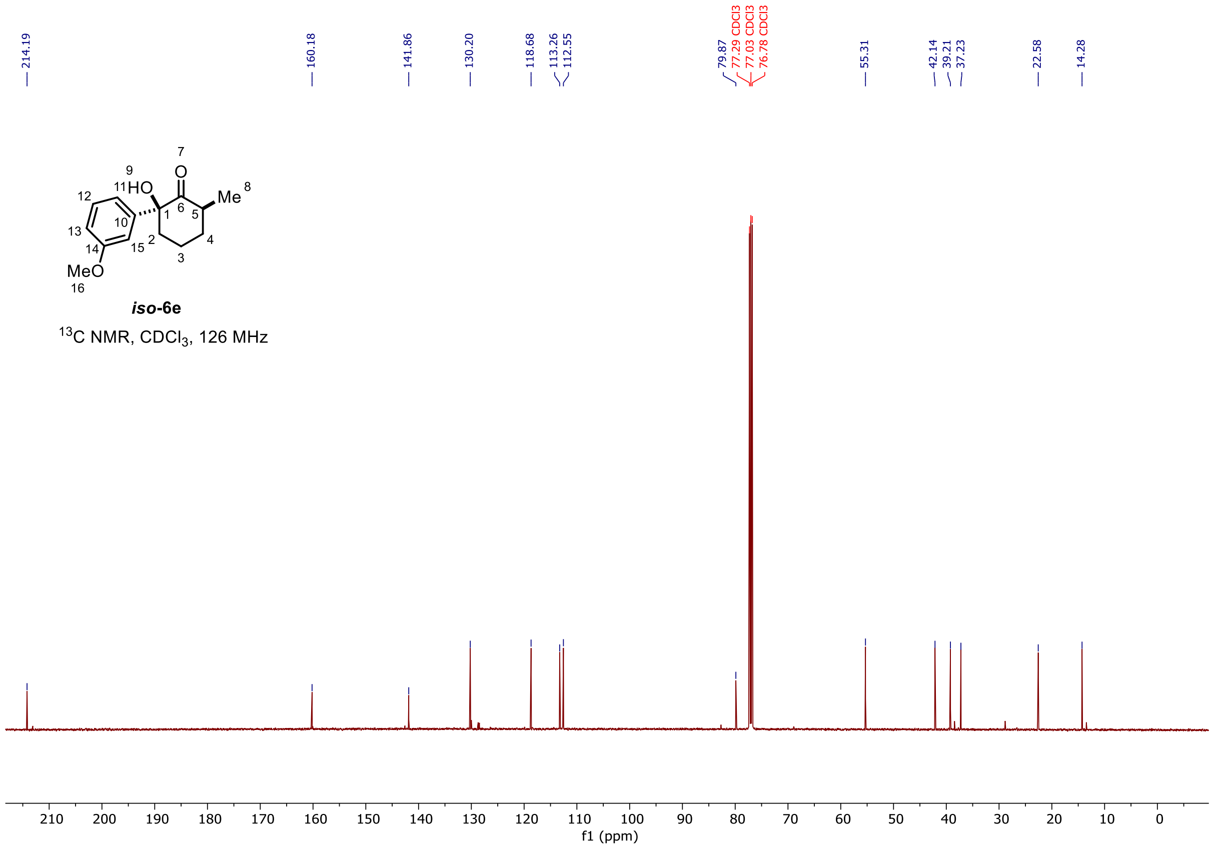

(trans)-2-Hydroxy-3-methyl-2-(3-(trifluoromethyl)phenyl)cyclohexan-1-one *iso*-6f

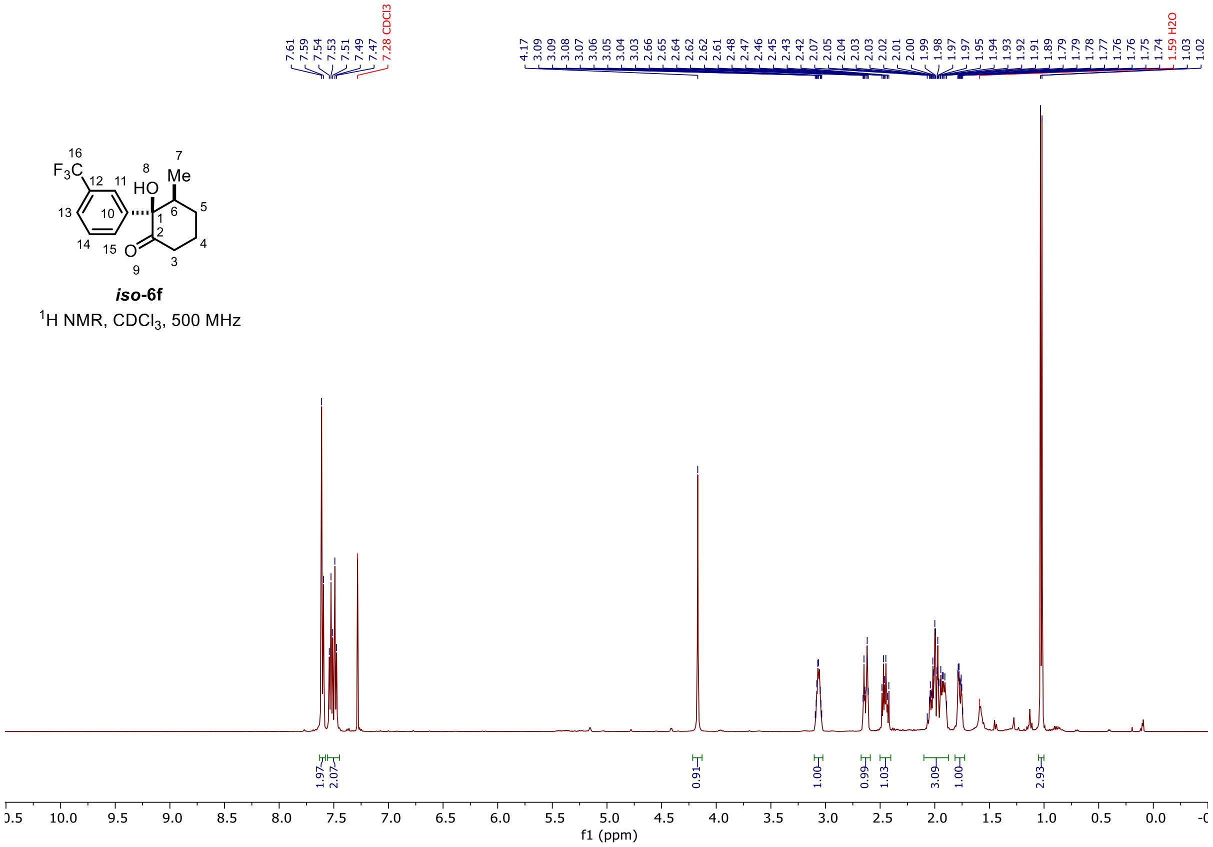

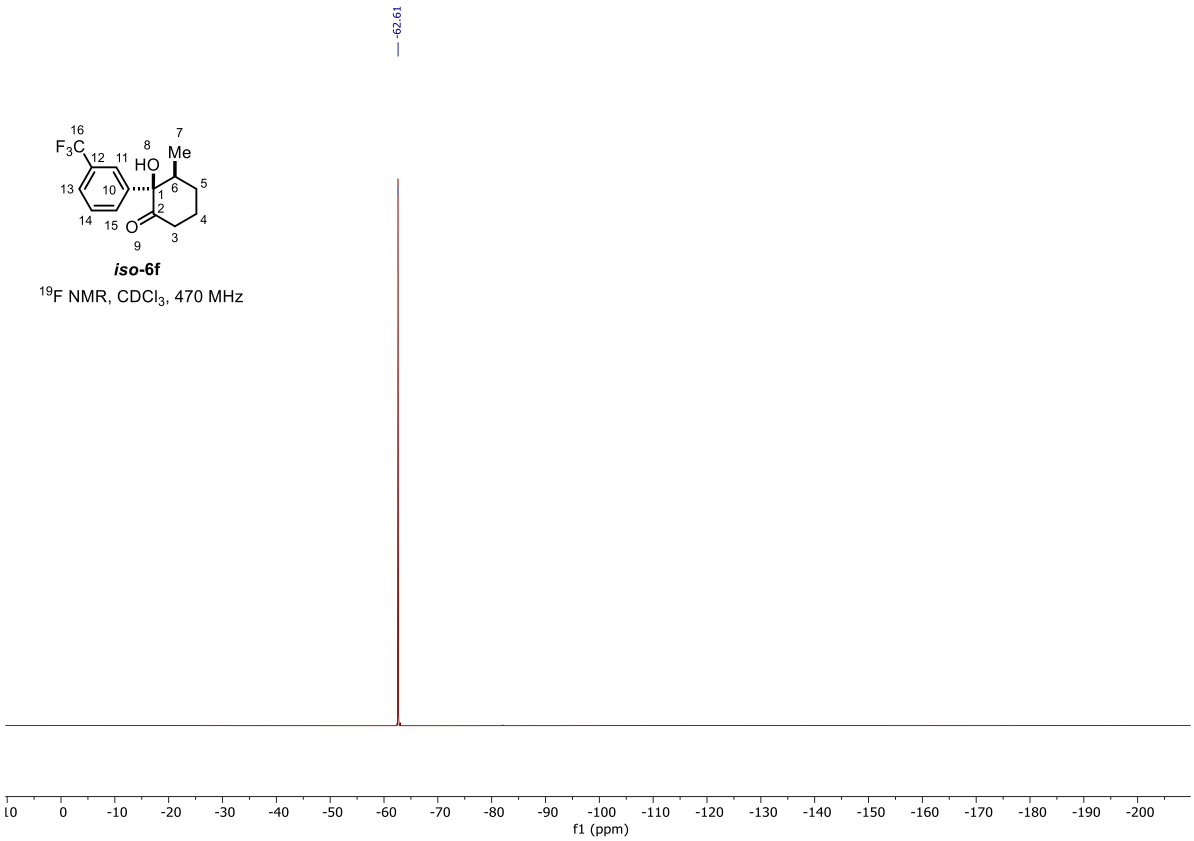

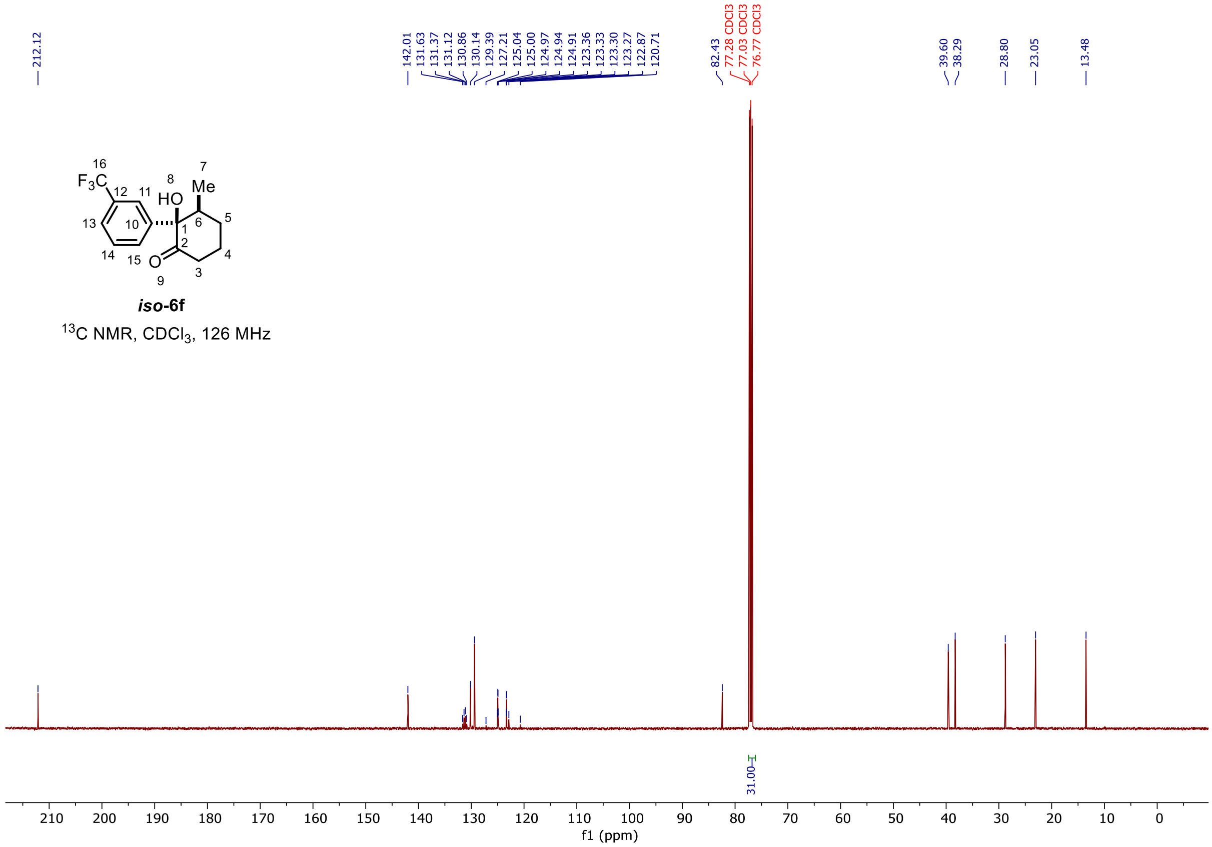

212.12
142.01
131.63
131.37
131.12
130.86
130.14
129.39
127.21
125.04
125.00
124.97
124.94
124.91
123.36
123.33
123.30
123.27
122.87
120.71
82.43
77.28 CDCl3
77.03 CDCl3
76.77 CDCl3
39.60
38.29
28.80
23.05
13.48
16
F_3C
12
11
8
HO
7
Me
13
10
1
6
5
2
14
15
O
4
3
9
iso-6f
^{13}C NMR, $CDCl_3$, 126 MHz
31.00
210
200
190
180
170
160
150
140
130
120
110
100
90
80
70
60
50
40
30
20
10
0
f1 (ppm)

(trans)-2-Hydroxy-6-methyl-2-(naphthalen-2-yl)cyclohexan-1-one iso-6g

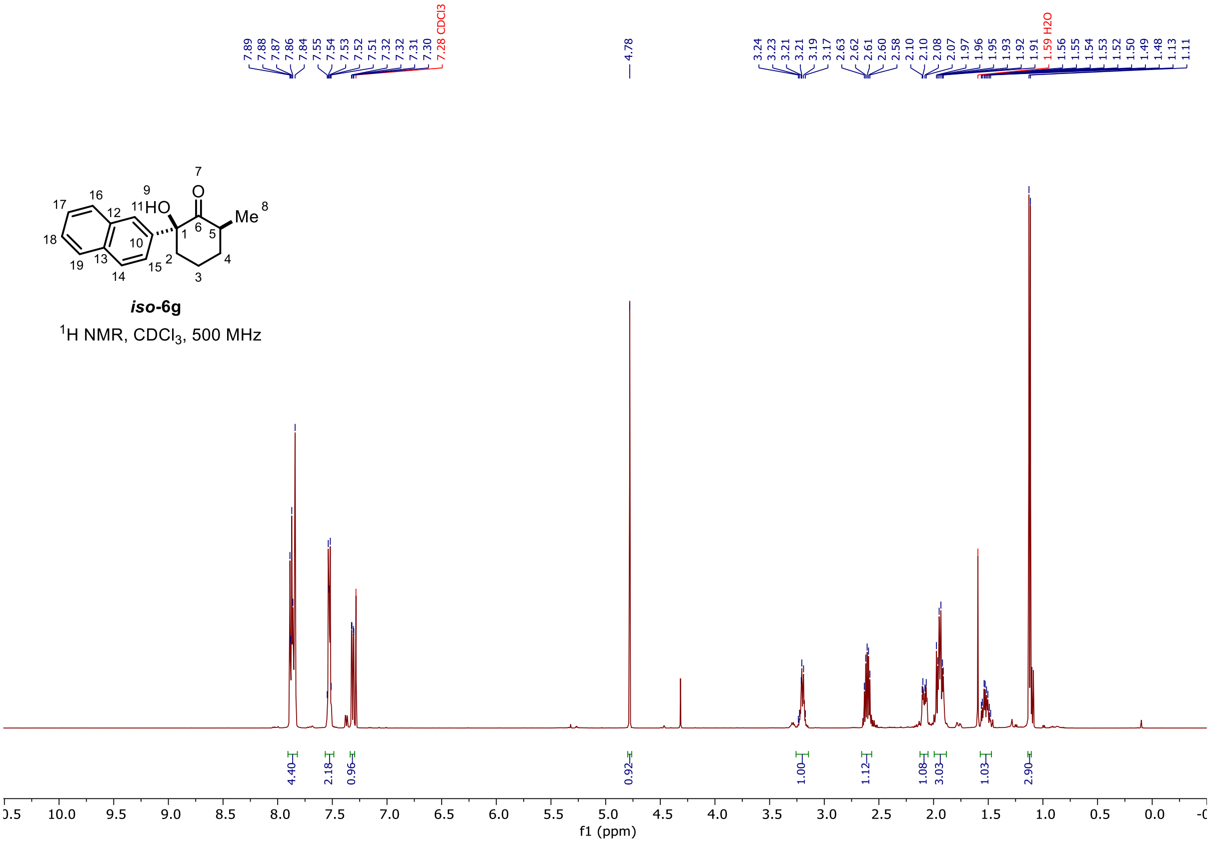

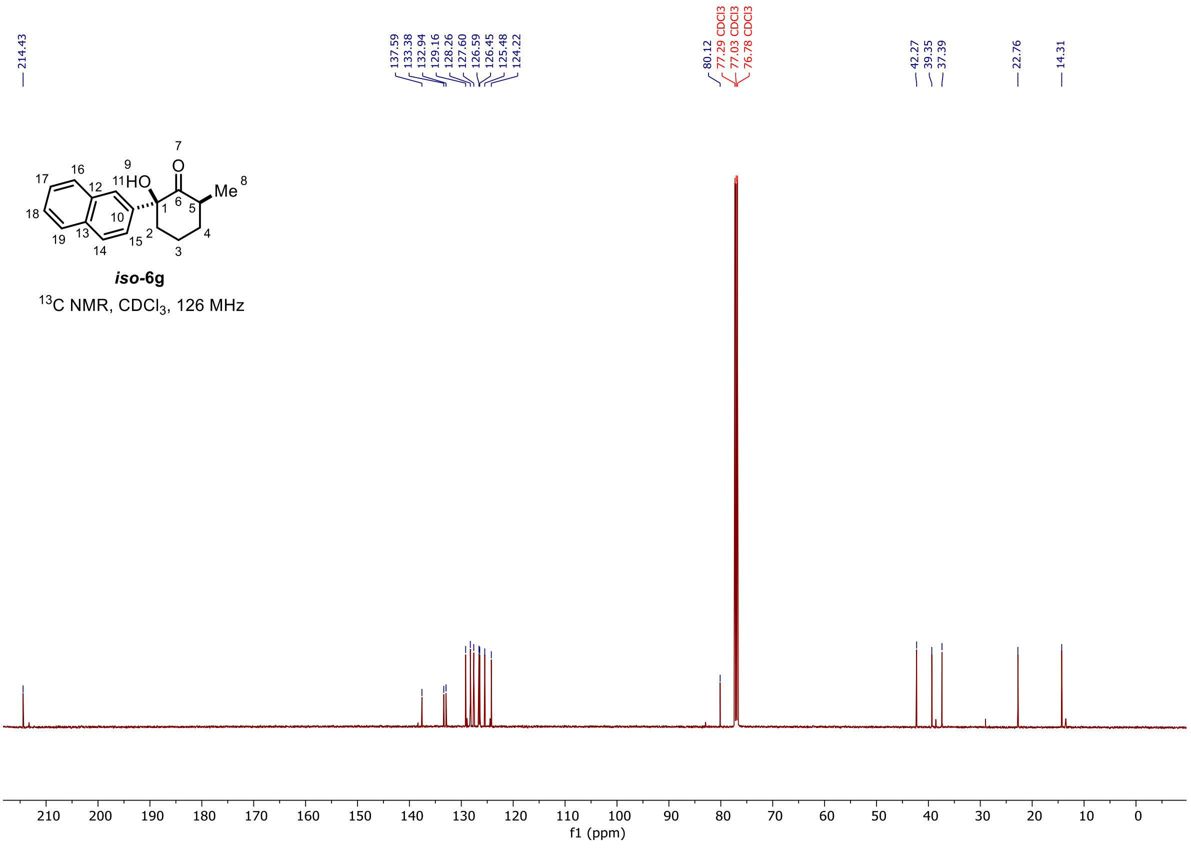

(*trans*)-2-(Furan-2-yl)-2-hydroxy-3-methylcyclohexan-1-one 6h and (*trans*)-2-(Furan-2-yl)-2-hydroxy-6-methylcyclohexan-1-one *iso*-6h

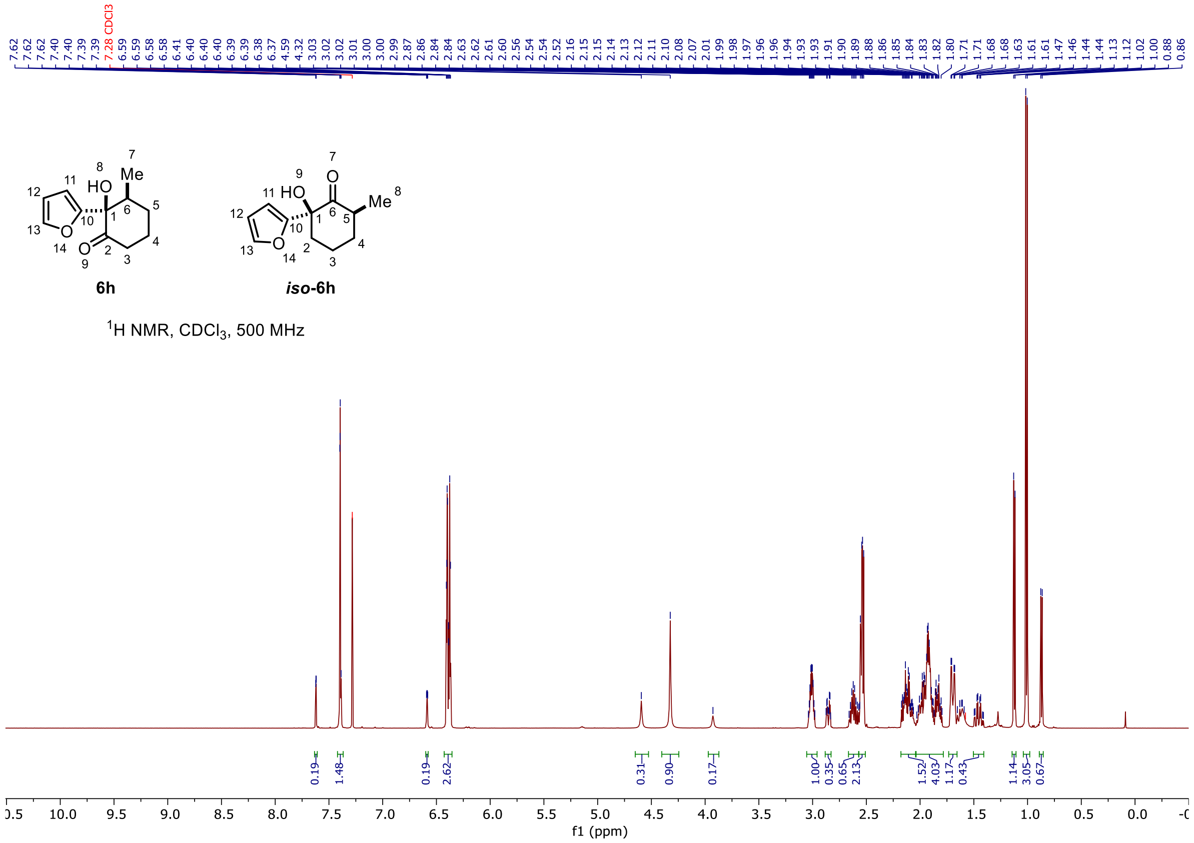

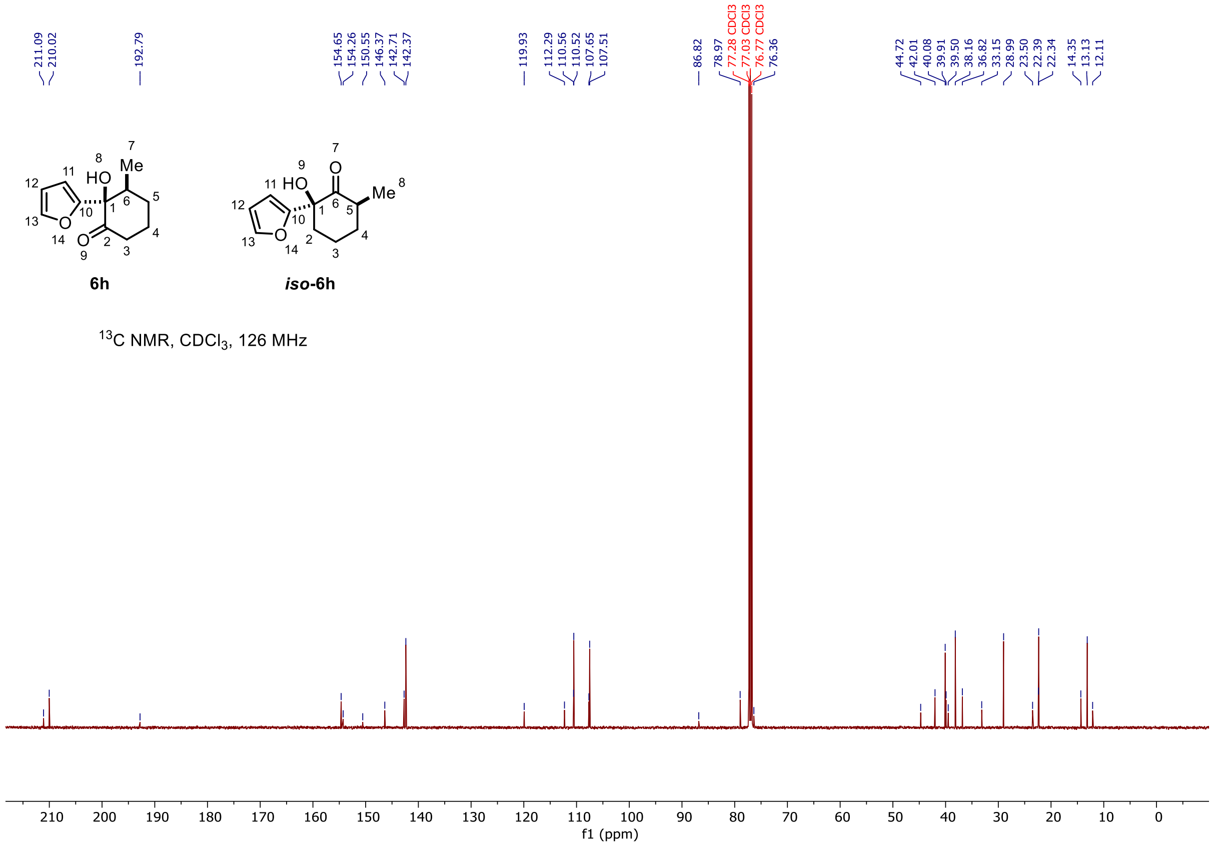

(trans)-2-Hydroxy-3-methyl-2-(1-methyl-1*H*-indol-2-yl)cyclohexan-1-one 6i

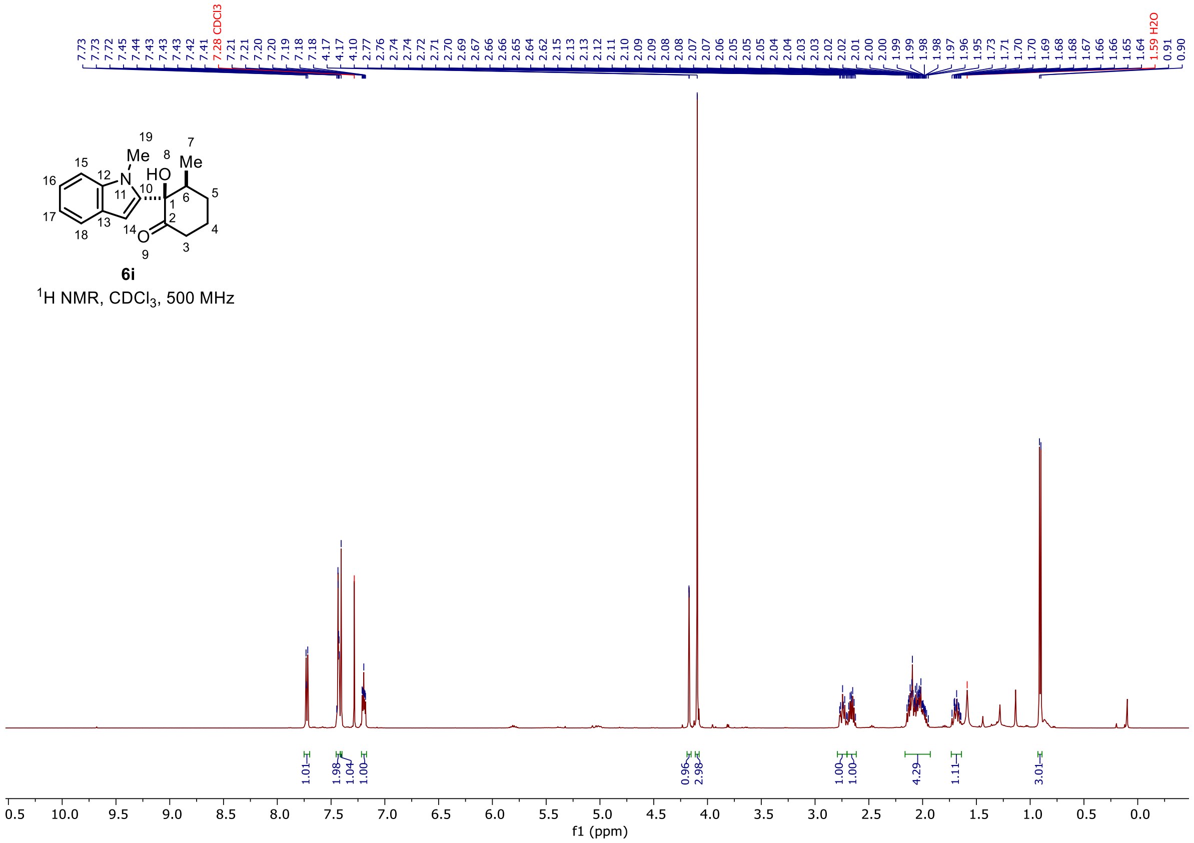

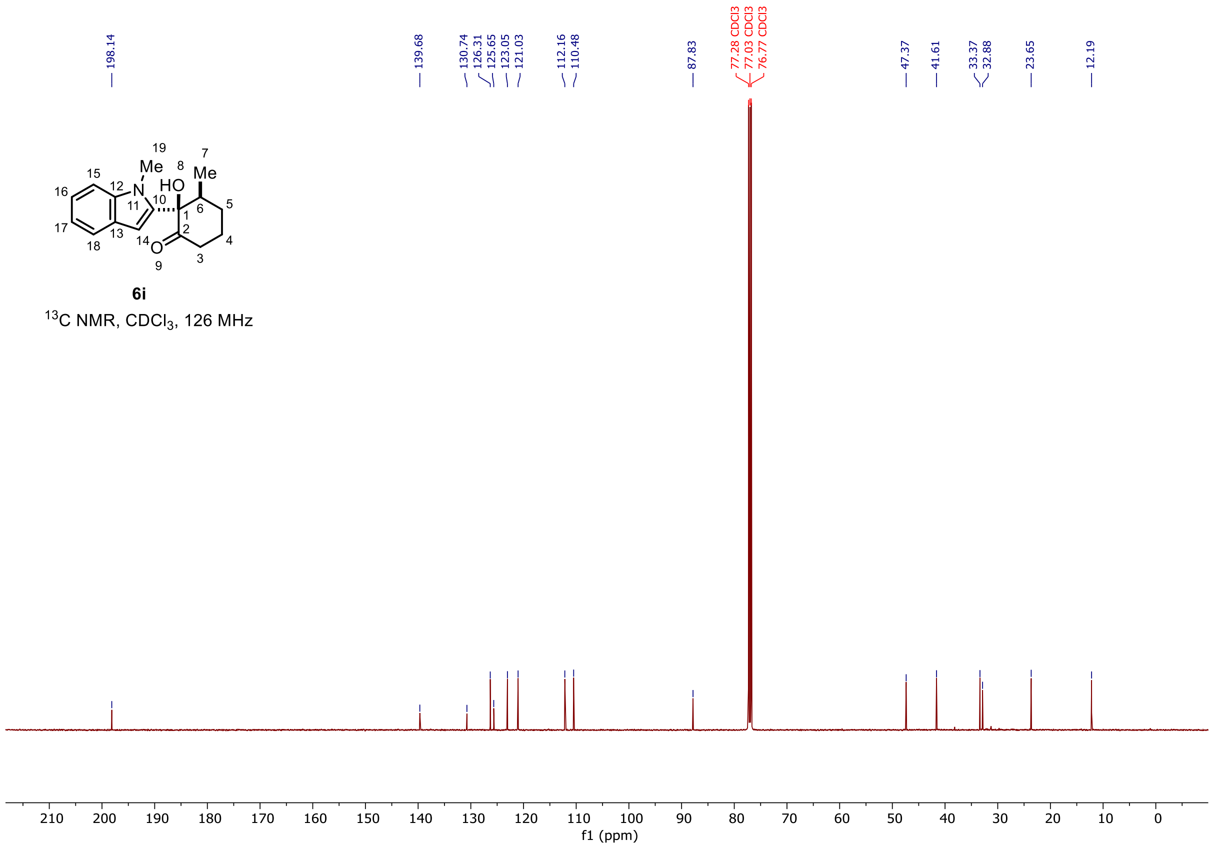

***(trans)*-2-(3-(Cyclopropylmethoxy)-4-(difluoromethoxy)phenyl)-2-hydroxy-6-methylcyclohexan-1-one *iso*-6j and (*trans*)-2-(3-(Cyclopropylmethoxy)-4-(difluoromethoxy)phenyl)-2-hydroxy-3-methylcyclohexan-1-one 6j**

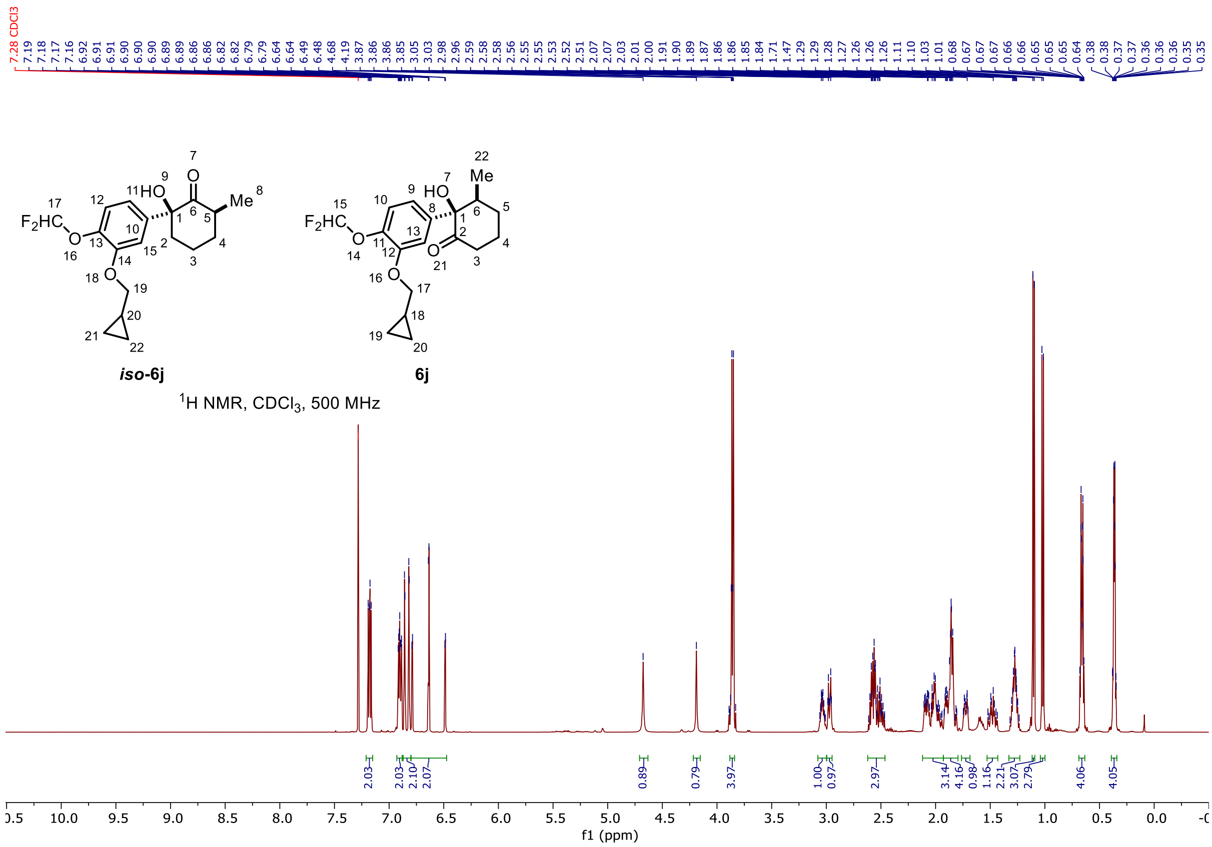

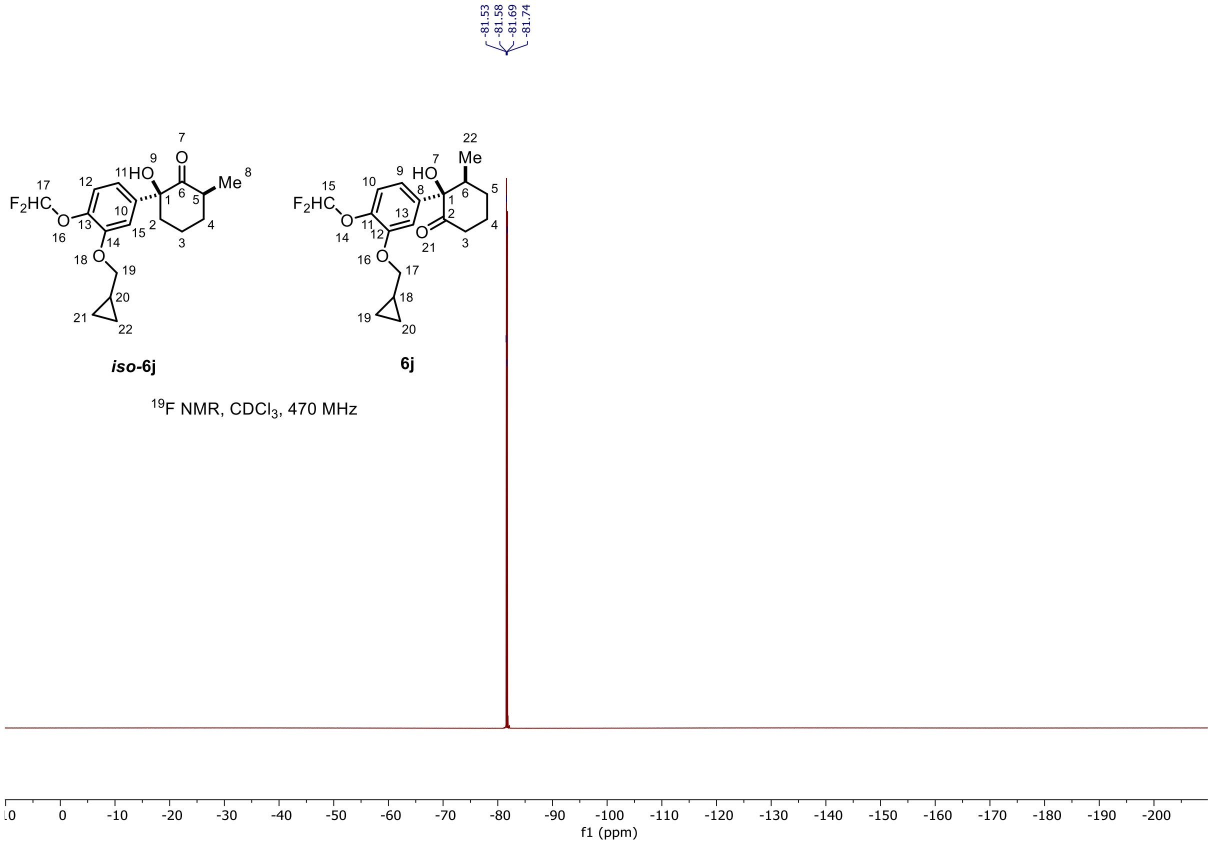

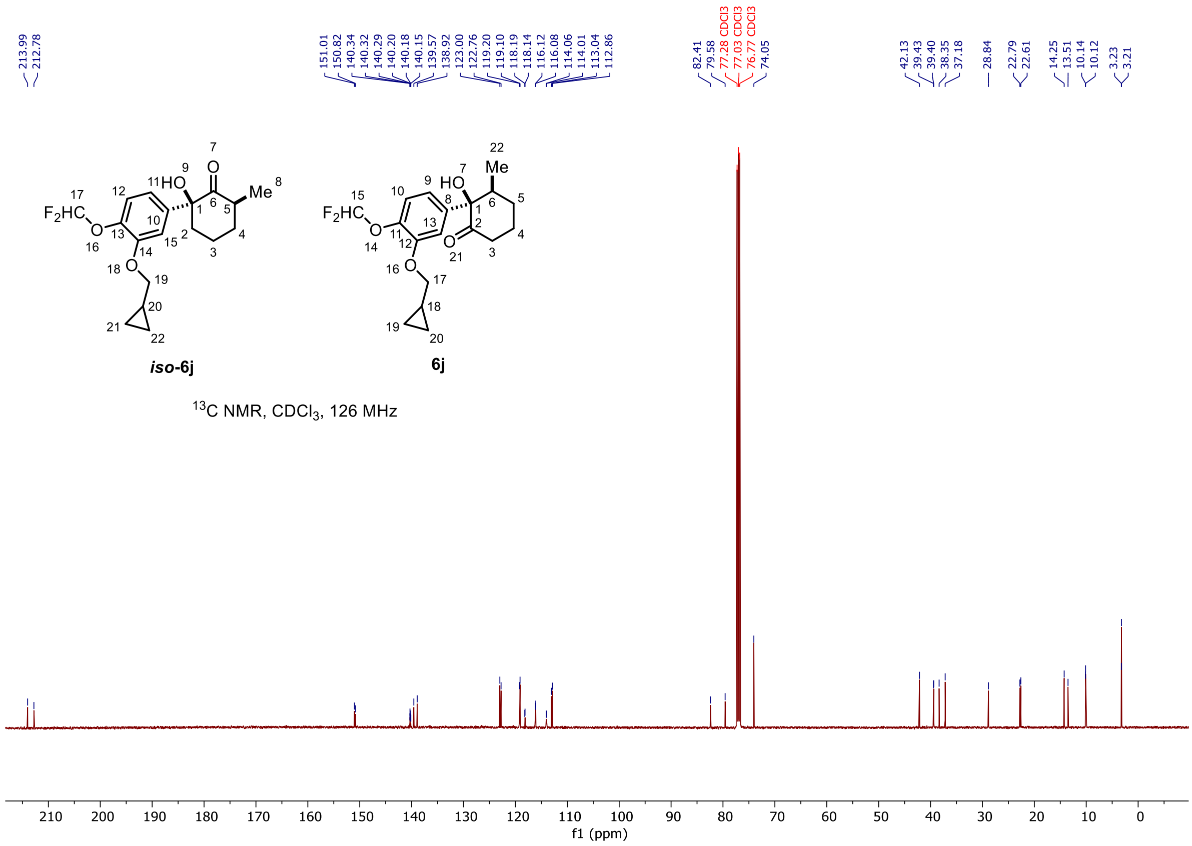

213.99
212.78
151.01
150.82
140.34
140.32
140.29
140.20
140.18
140.15
139.57
138.92
123.00
122.76
119.20
119.10
118.19
118.14
116.12
116.08
114.06
114.01
113.04
112.86
82.41
79.58
77.28 CDCl3
77.03 CDCl3
76.77 CDCl3
74.05
42.13
39.43
39.40
38.35
37.18
28.84
22.79
22.61
14.25
13.51
10.14
10.12
3.23
3.21
iso-6j
6j
^{13}C NMR, $CDCl_3$, 126 MHz
f1 (ppm)

(*trans*)-3-Hydroxy-5-methyl-3-phenyltetrahydro-4*H*-pyran-4-one *iso*-6k and (*trans*)-3-Hydroxy-4-methyltetrahydrofuran-3-yl)(phenyl)methanone 6k'

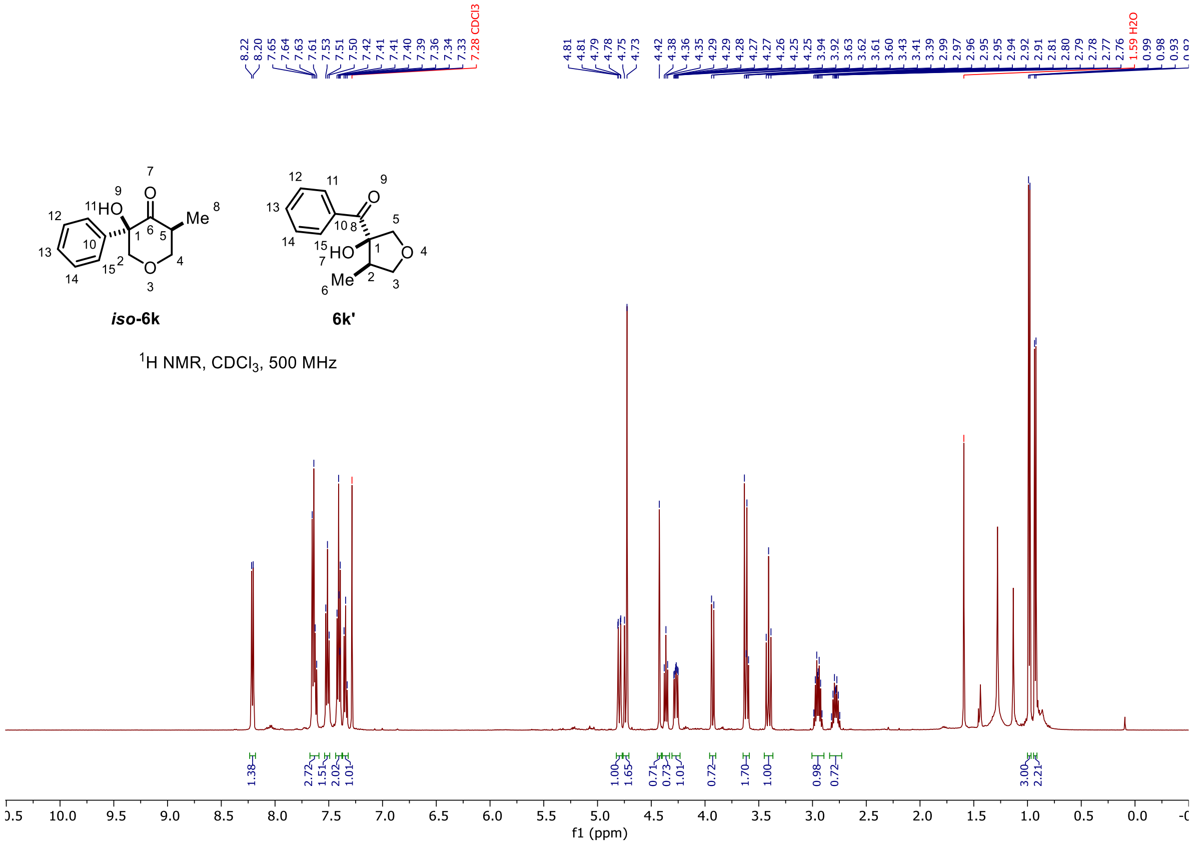

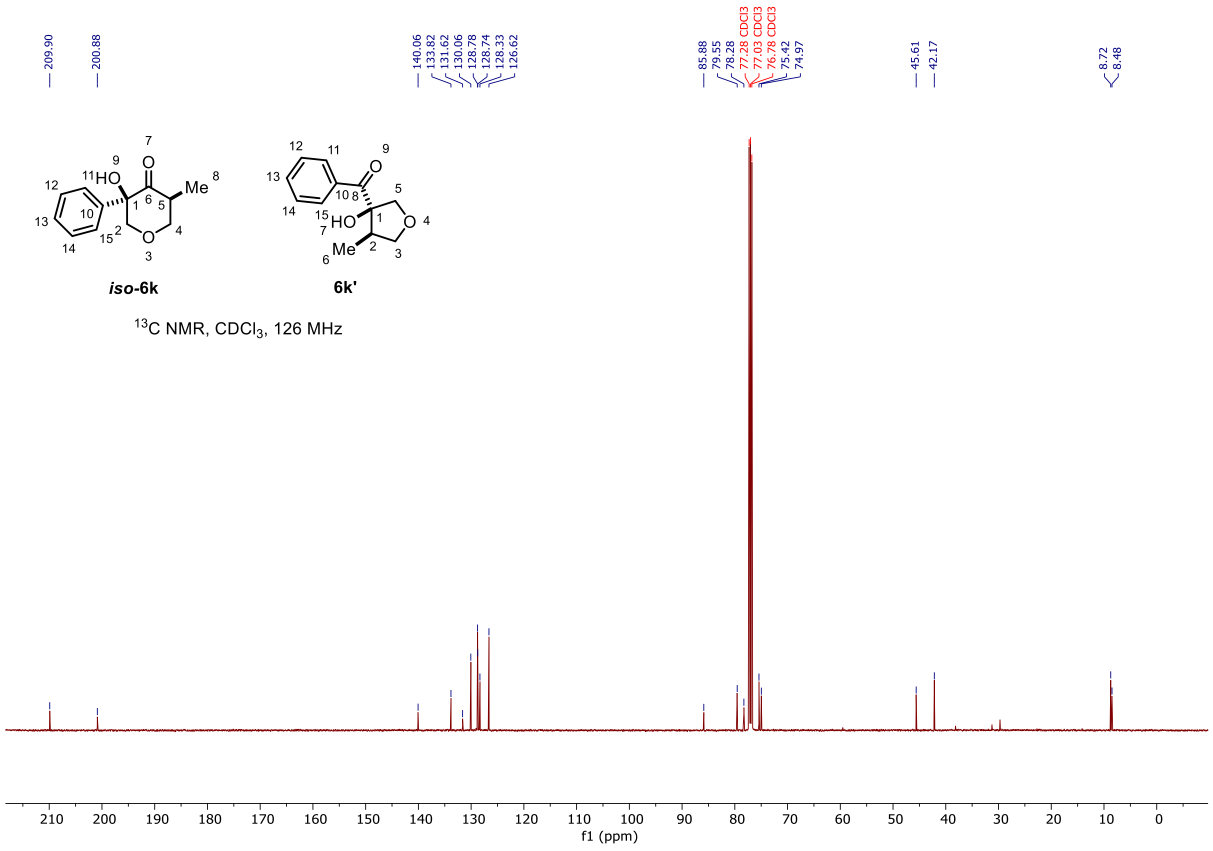

(*trans)*-(1-Hydroxy-2-methylcyclohexyl)(phenyl)methanone 8a

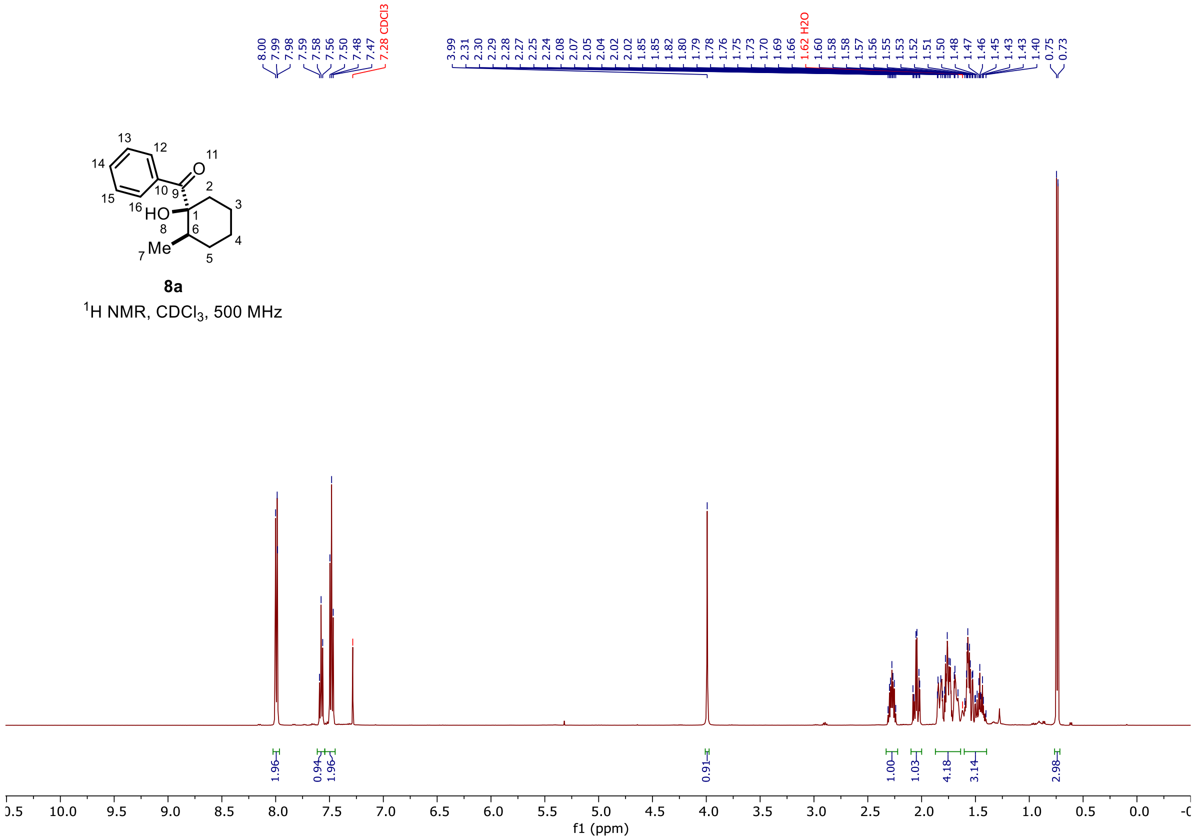

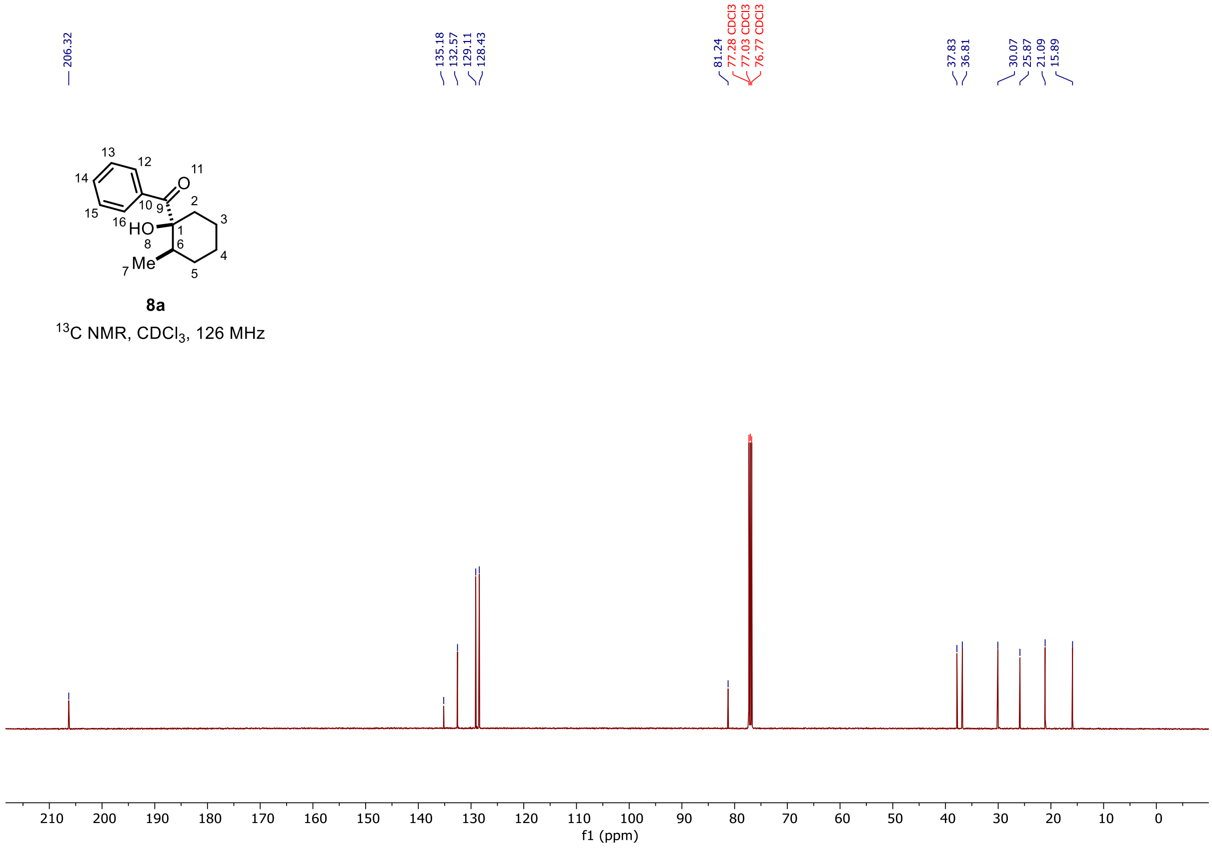

(*trans)*-(1-Hydroxy-2-methylcyclohexyl)(p-tolyl)methanone 8b

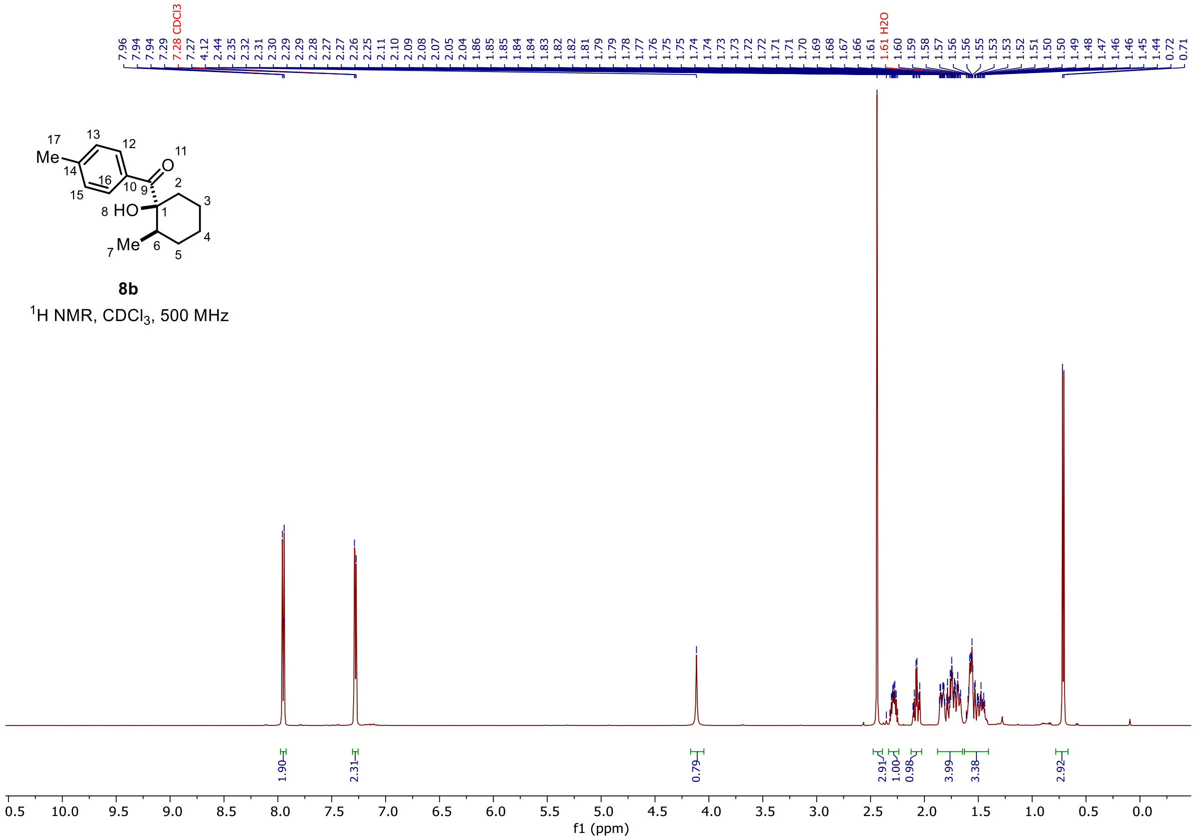

8b

^{1}H NMR, $CDCl_3$, 500 MHz

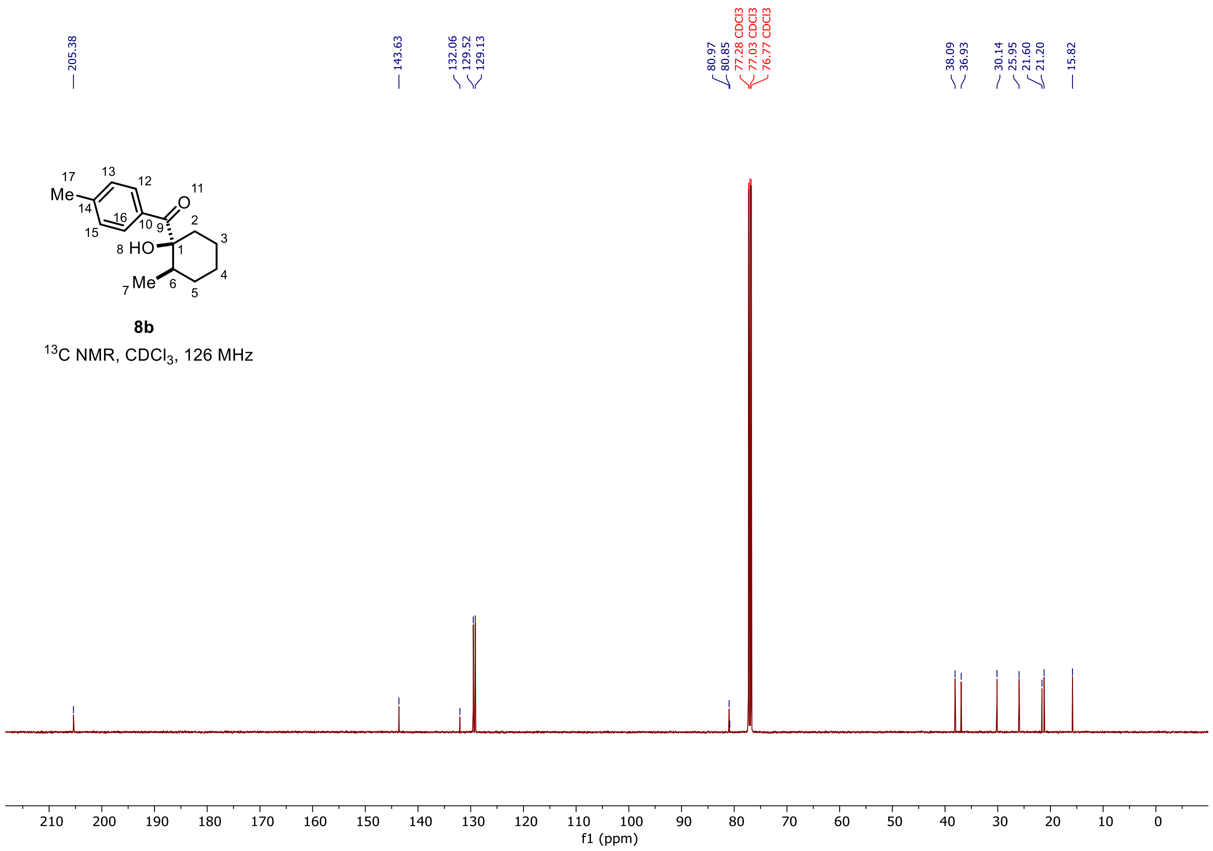

8b

^{13}C NMR, $CDCl_3$, 126 MHz

(4-Chlorophenyl)-(*trans*)-(1-hydroxy-2-methylcyclohexyl)methanone 8c

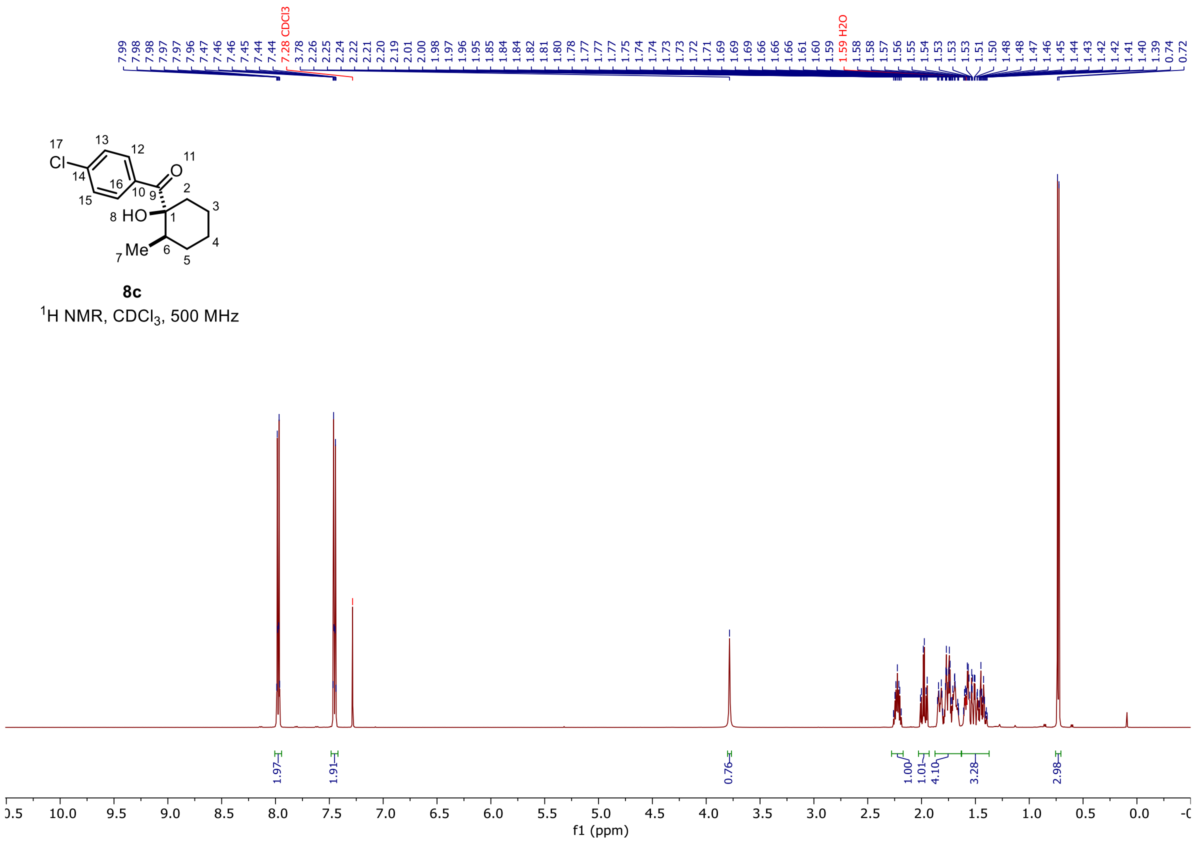

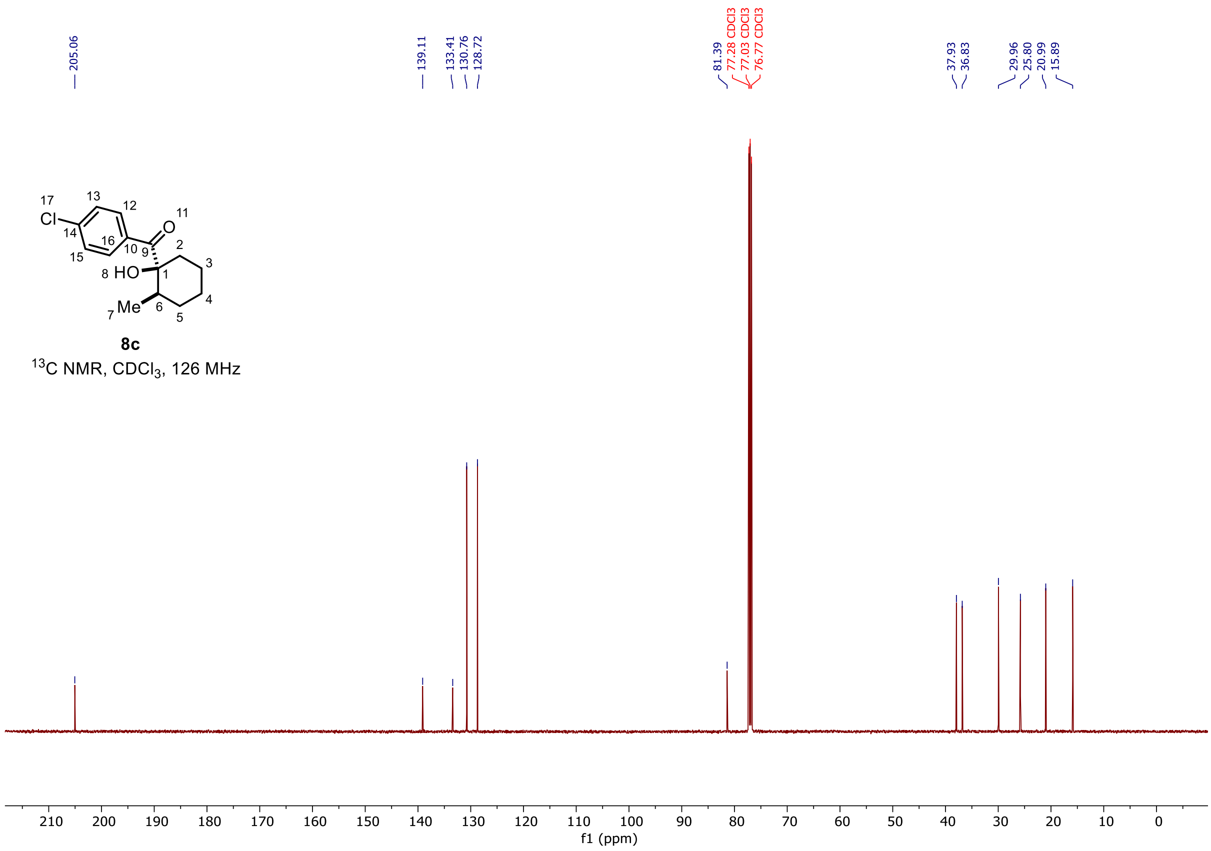

(2-Fluorophenyl)-(*trans)*-(1-hydroxy-2-methylcyclohexyl)methanone 8d

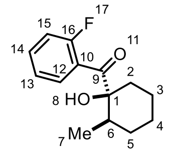

8d

^{1}H NMR, $CDCl_3$, 500 MHz

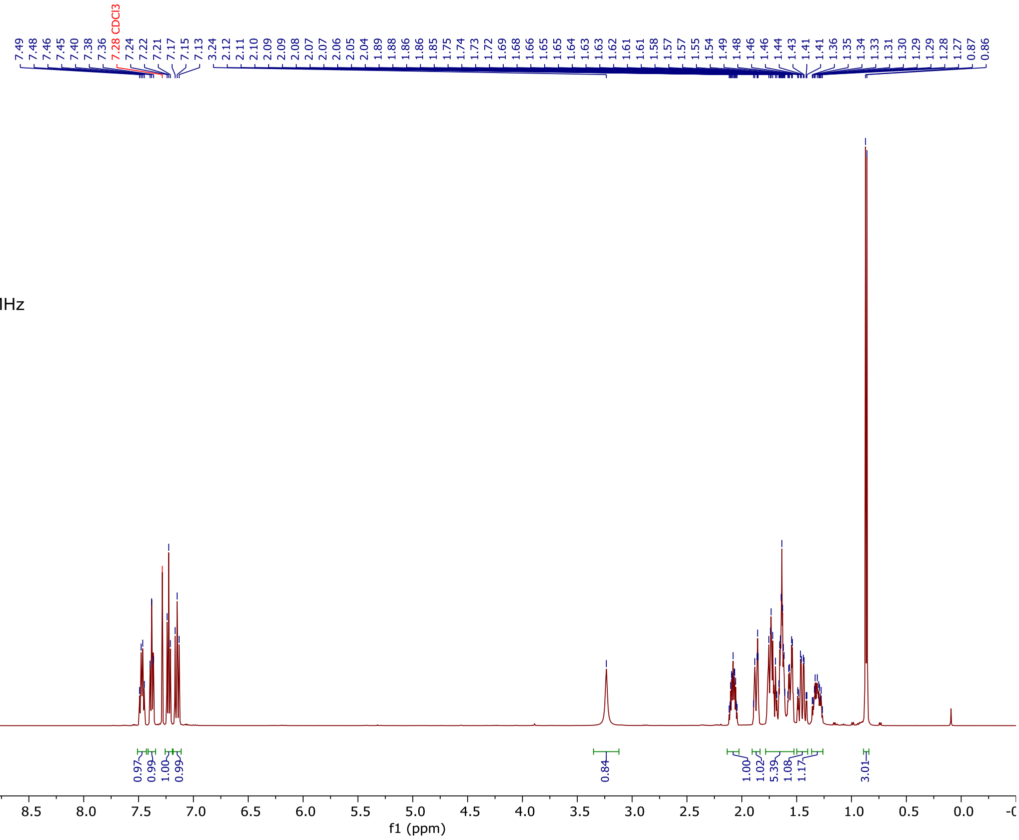

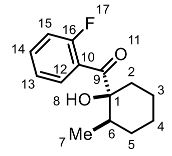

8d

^{19}F NMR, $CDCl_3$, 470 MHz

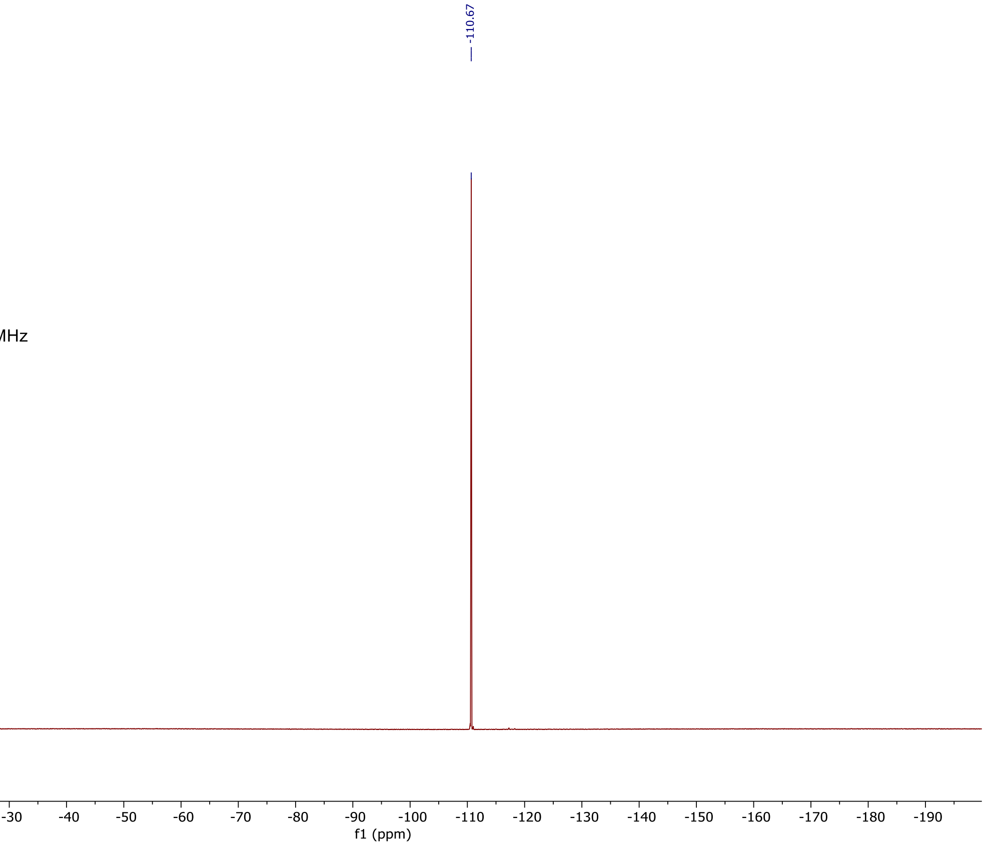

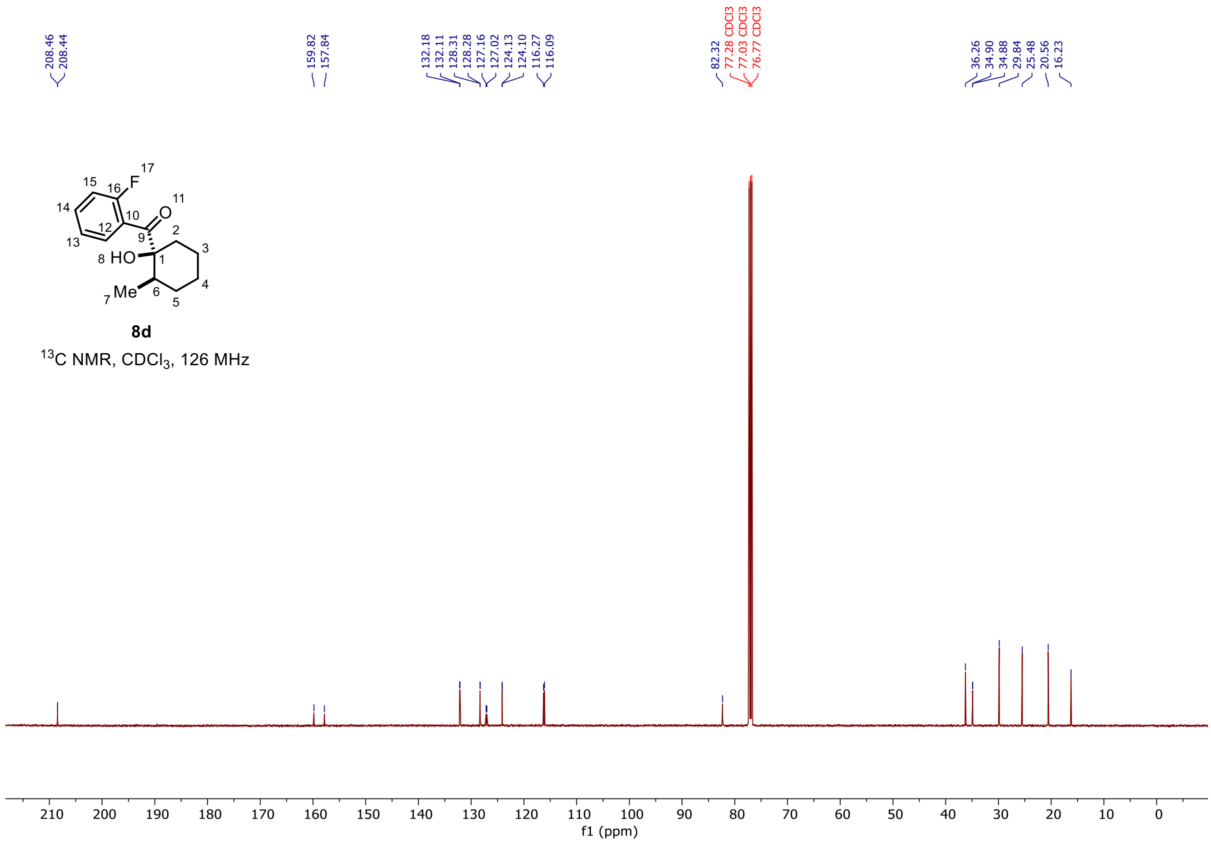
208.46
208.44
159.82
157.84
132.18
132.11
128.31
128.28
127.16
127.02
124.13
124.10
116.27
116.09
82.32
77.28 CDCl3
77.03 CDCl3
76.77 CDCl3
36.26
34.90
34.88
29.84
25.48
20.56
16.23
8d
^{13}C NMR, CDCl$_3$, 126 MHz
f1 (ppm)

(*trans*)-(1-Hydroxy-2-methylcyclohexyl)(3-methoxyphenyl)methanone 8e

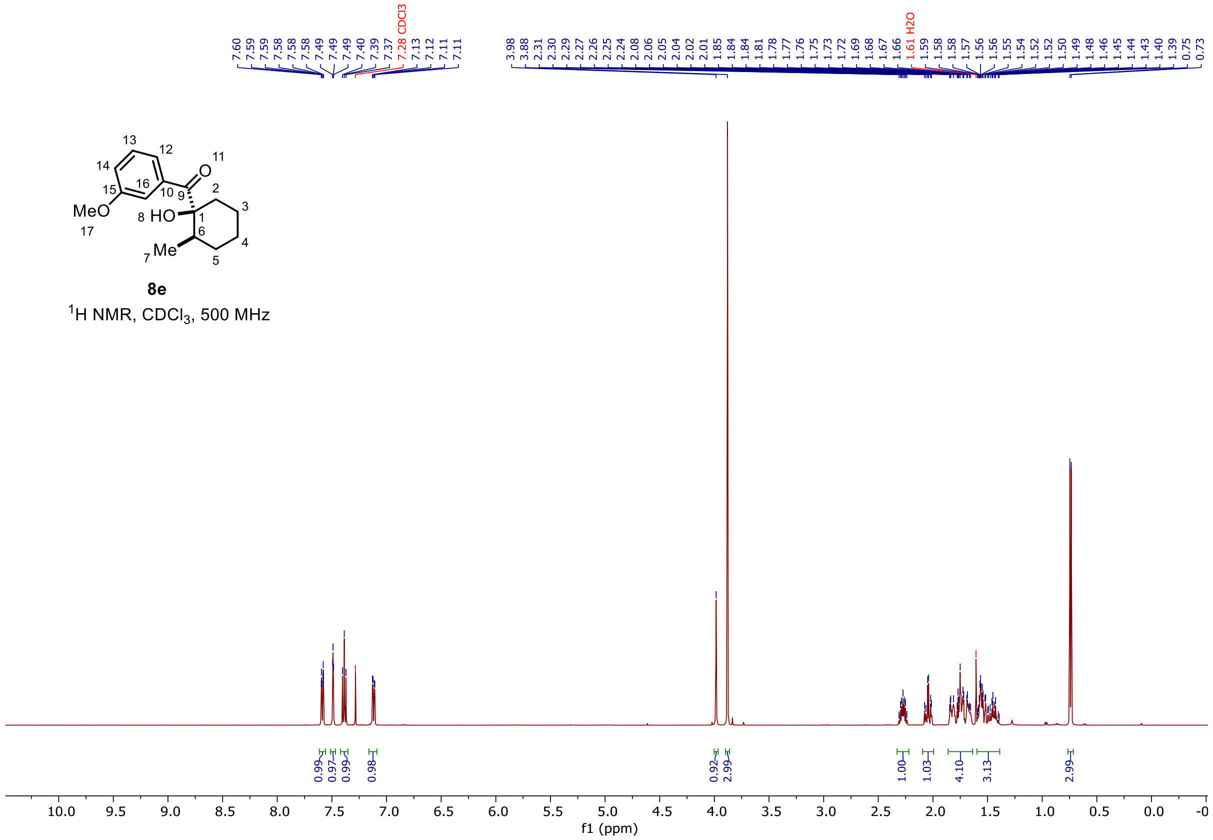

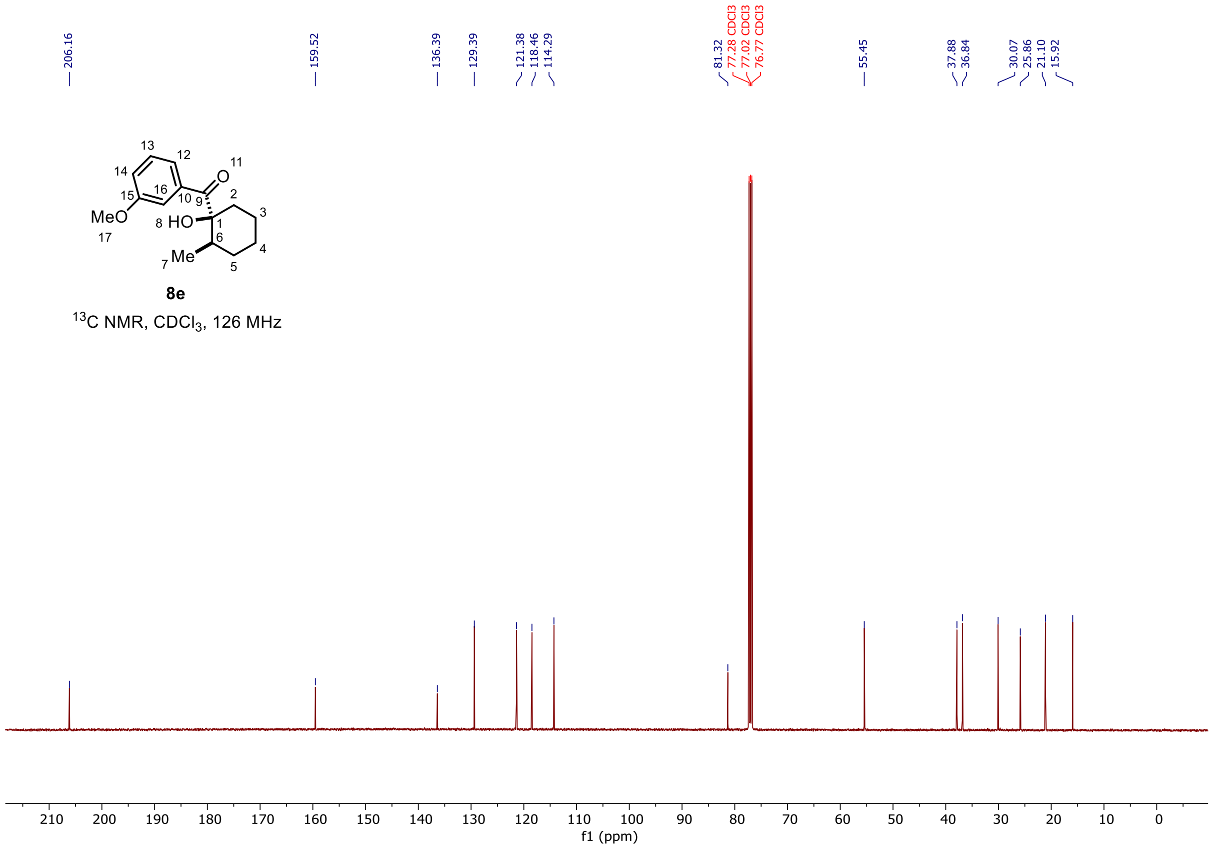

(*trans*)-(1-Hydroxy-2-methylcyclohexyl)(3-(trifluoromethyl)phenyl)methanone 8f

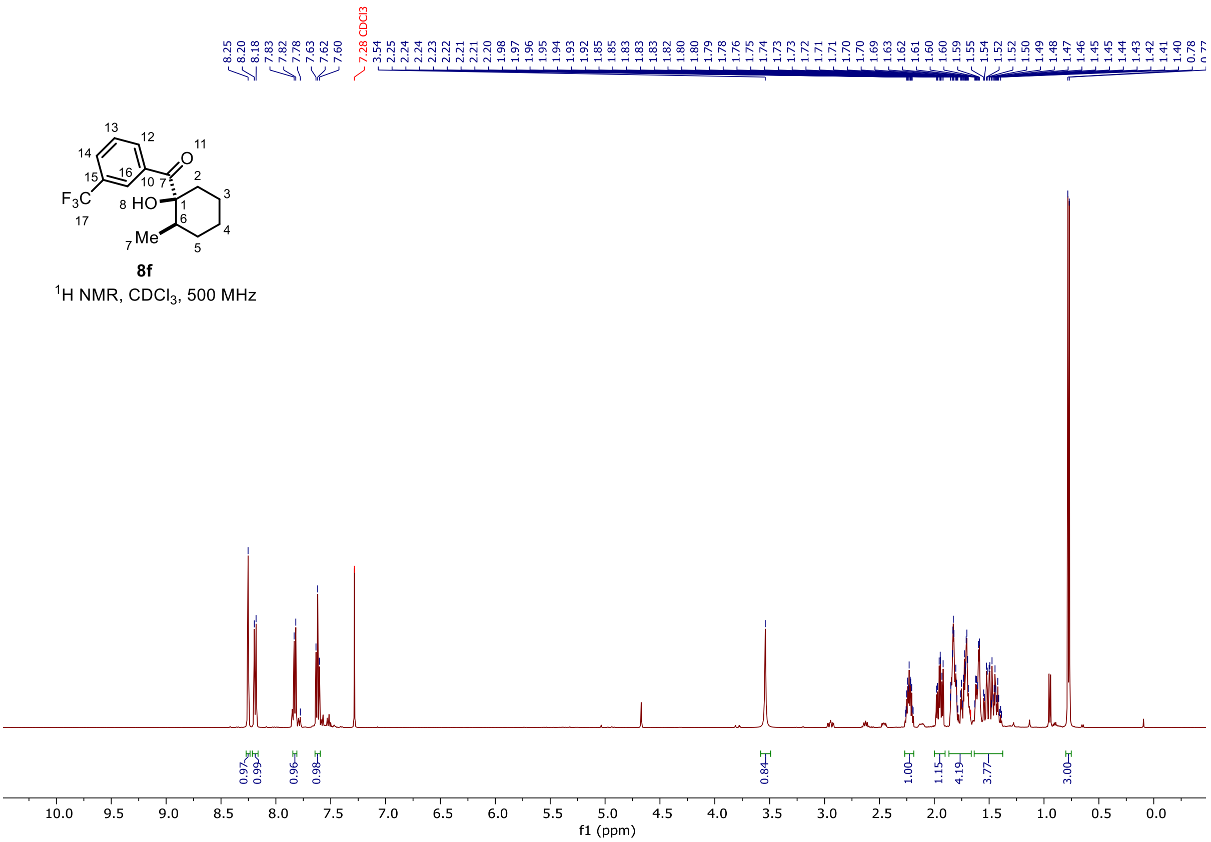

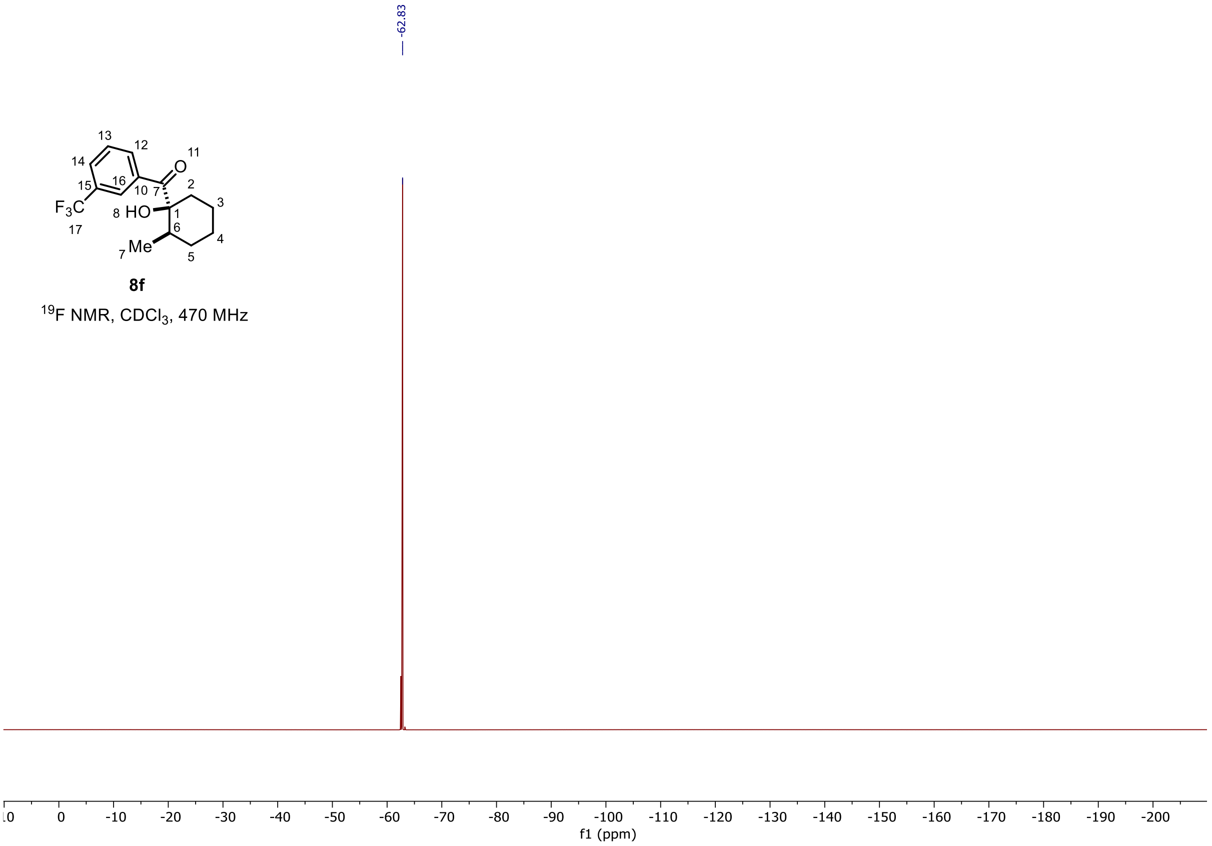

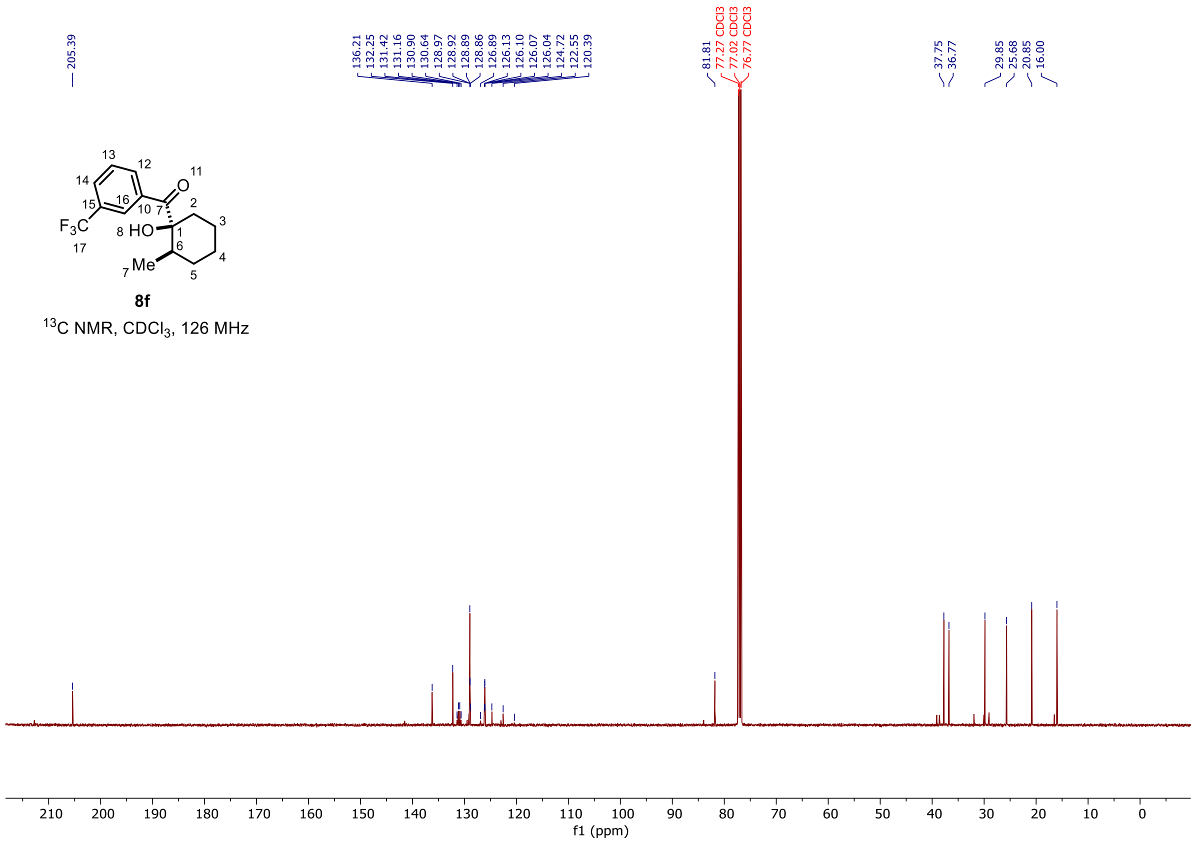
205.39
136.21
132.25
131.42
131.16
130.90
130.64
128.97
128.92
128.89
128.86
126.89
126.13
126.10
126.07
126.04
124.72
122.55
120.39
81.81
77.27 CDCl3
77.02 CDCl3
76.77 CDCl3
37.75
36.77
29.85
25.68
20.85
16.00
8f
13C NMR, CDCl3, 126 MHz
f1 (ppm)

(*trans*)-(1-Hydroxy-2-methylcyclohexyl)(naphthalen-2-yl)methanone 8g

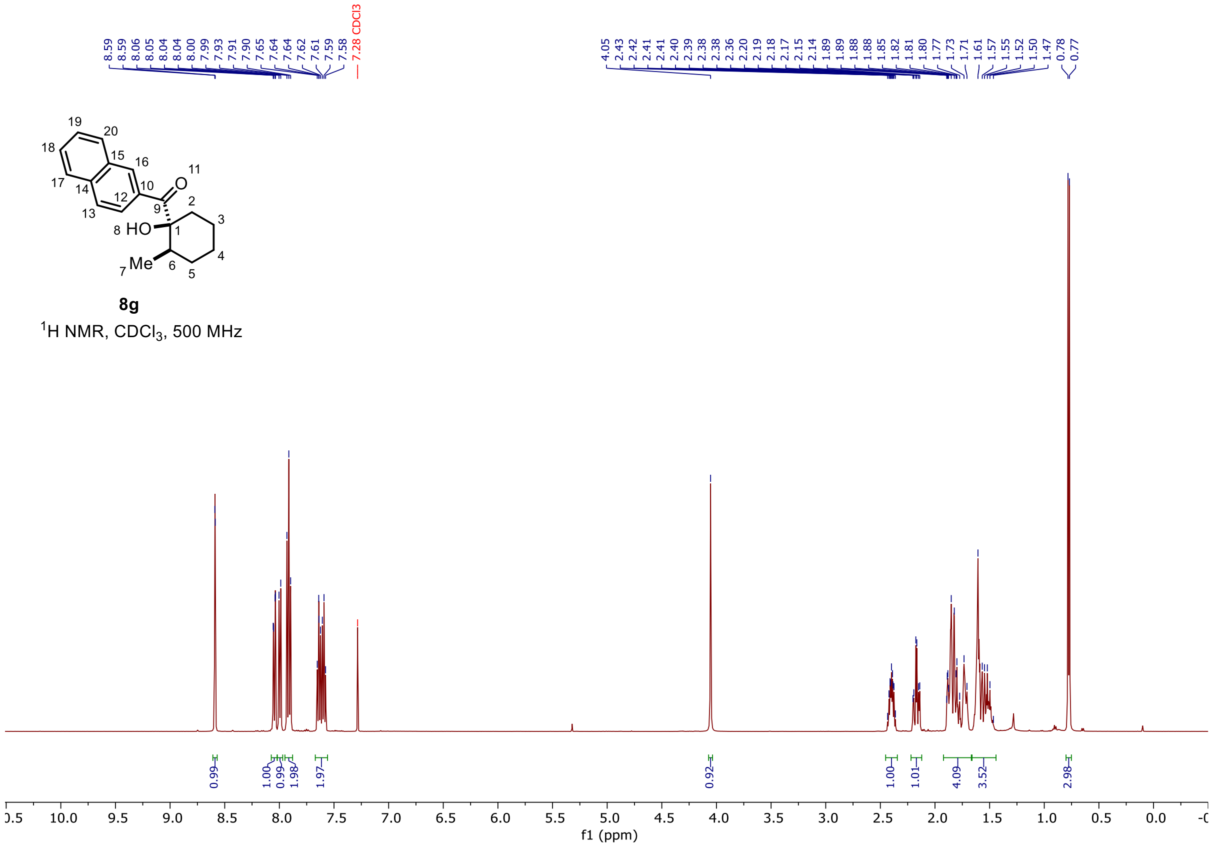

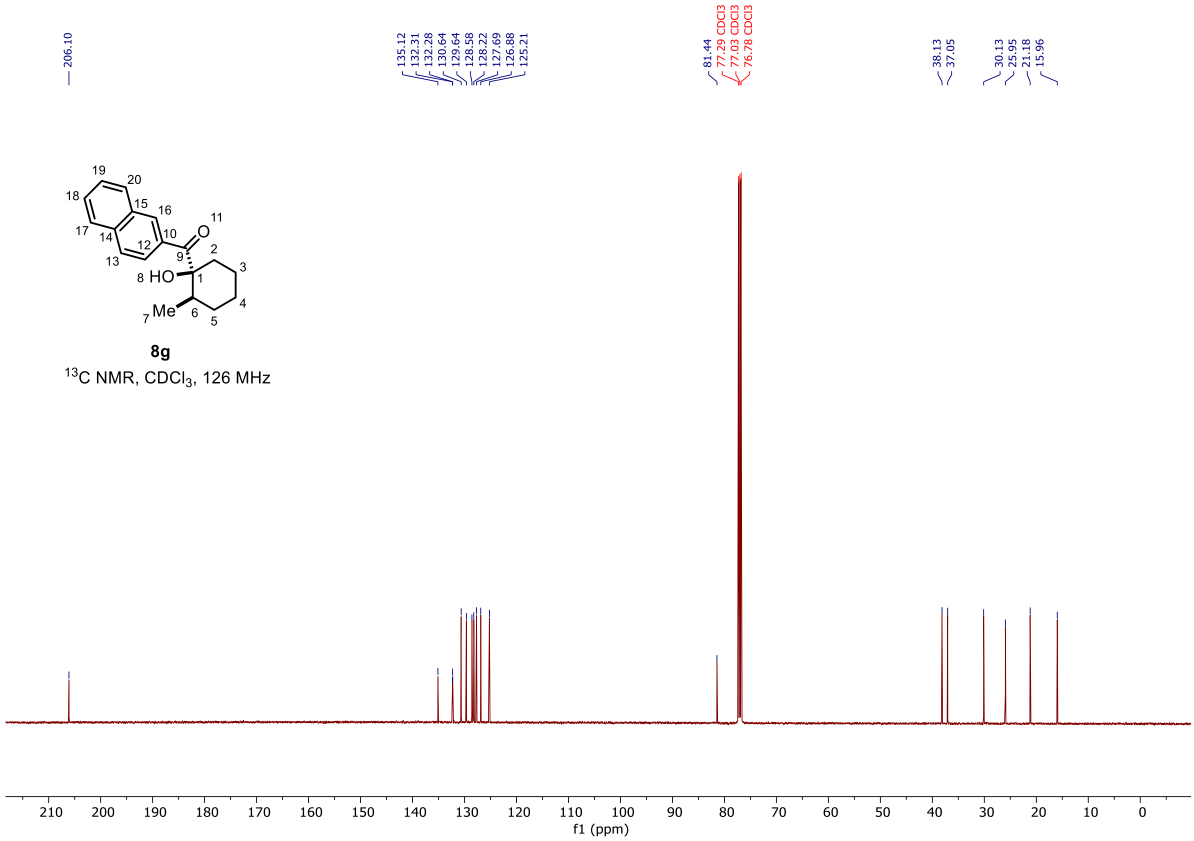

(Furan-2-yl)-(*trans)*-(1-hydroxy-2-methylcyclohexyl)methanone 8h

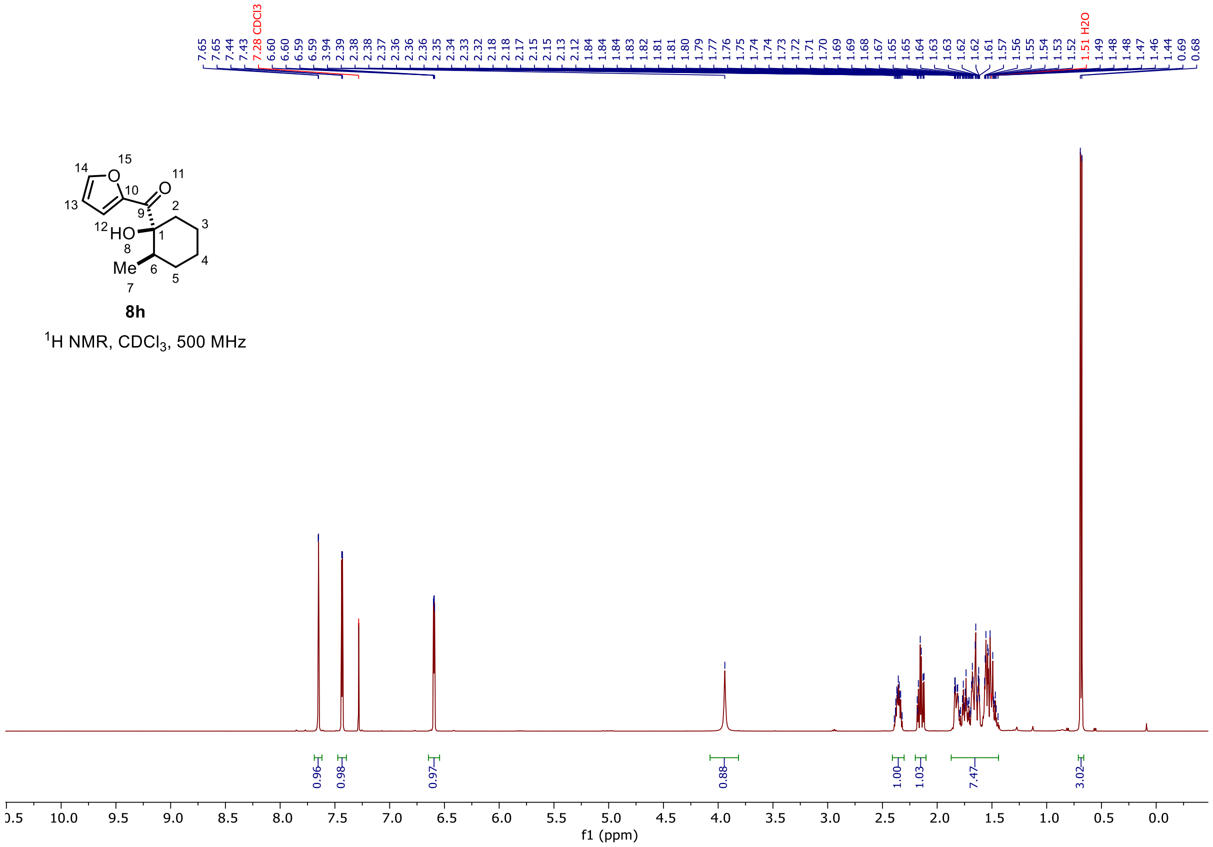

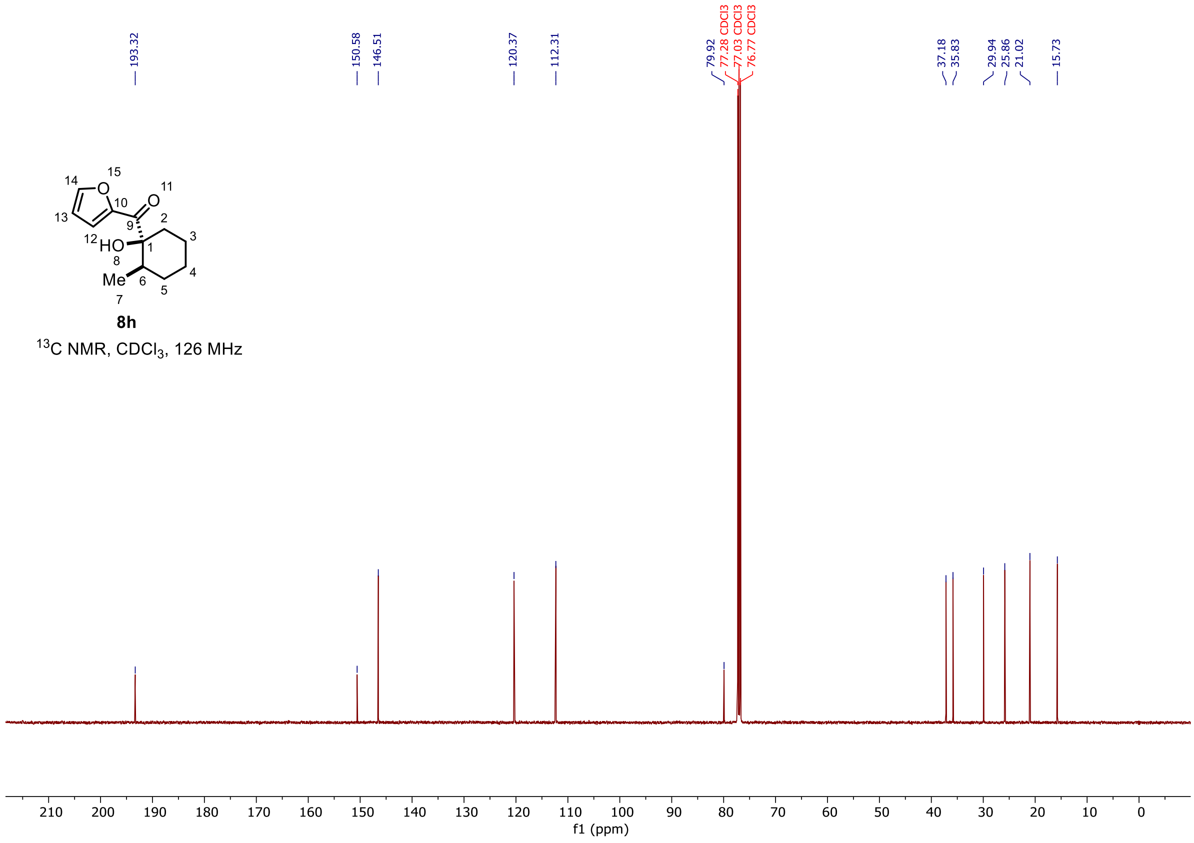

(*trans)*-(1-Hydroxy-2-methylcyclohexyl)(1-methyl-1*H*-indol-2-yl)methanone 8i

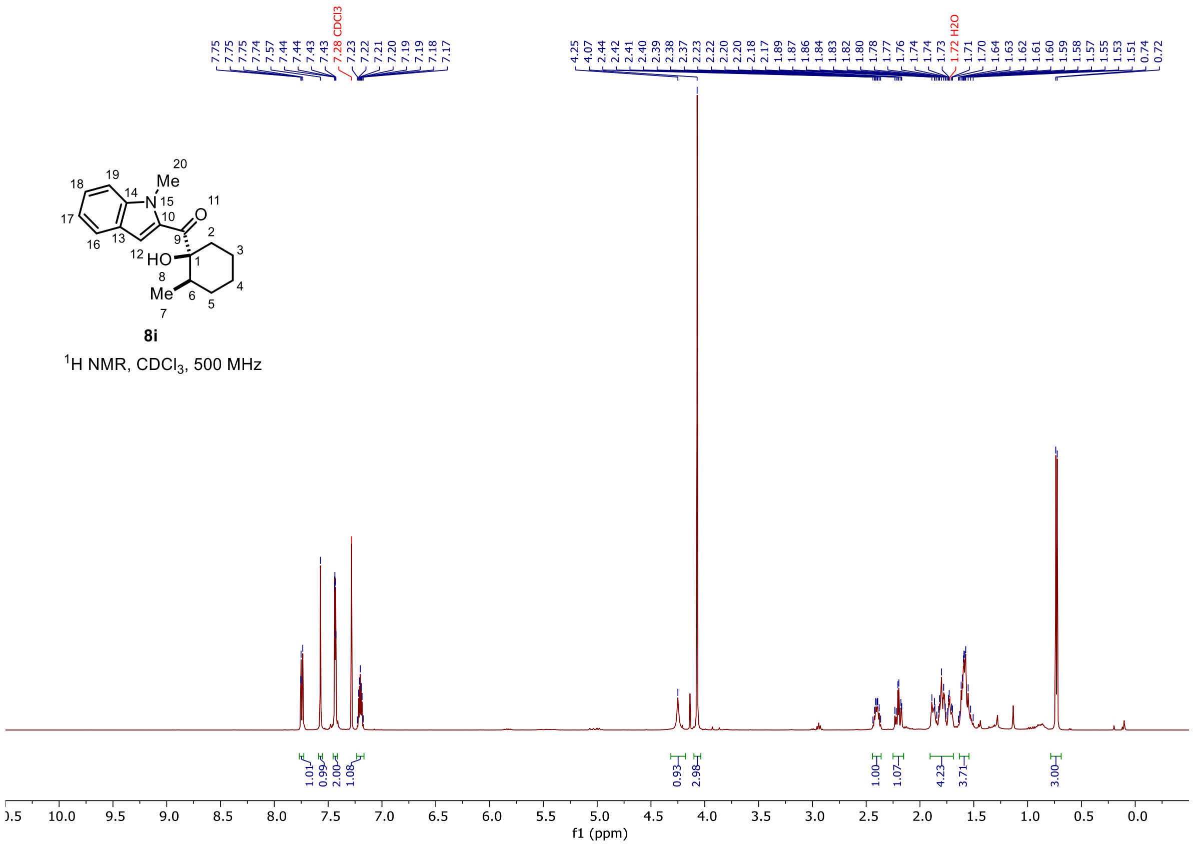

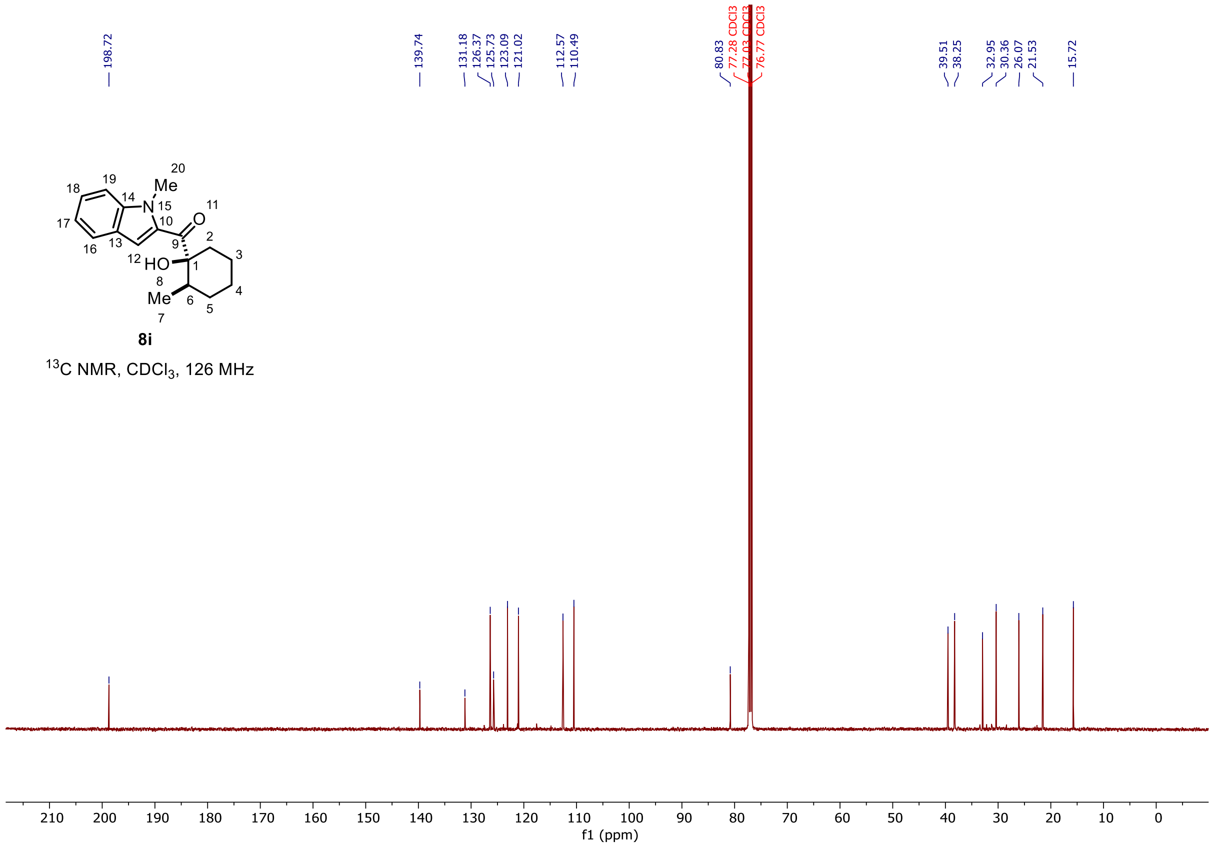

(3-(Cyclopropylmethoxy)-4-(difluoromethoxy)phenyl)-(*trans)*-(1-hydroxy-2-methylcyclohexyl)methanone 8j

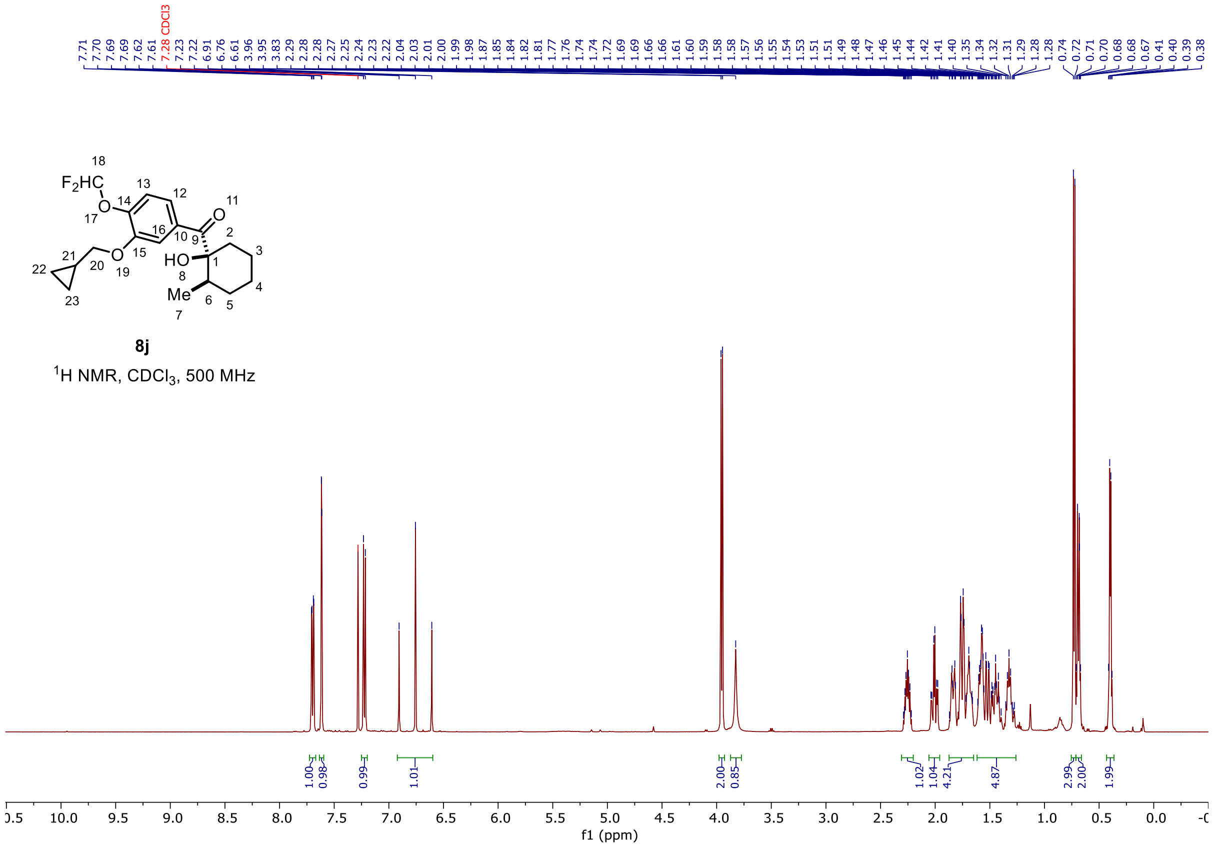

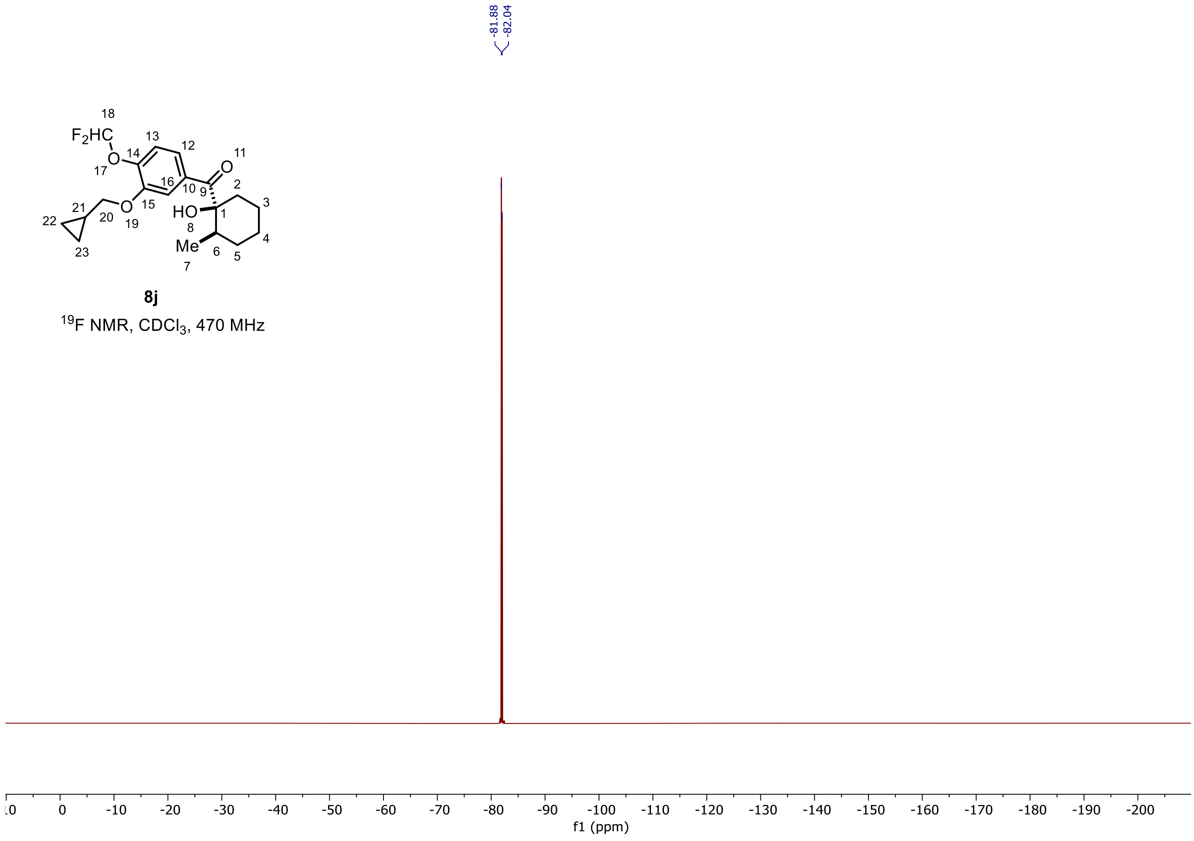

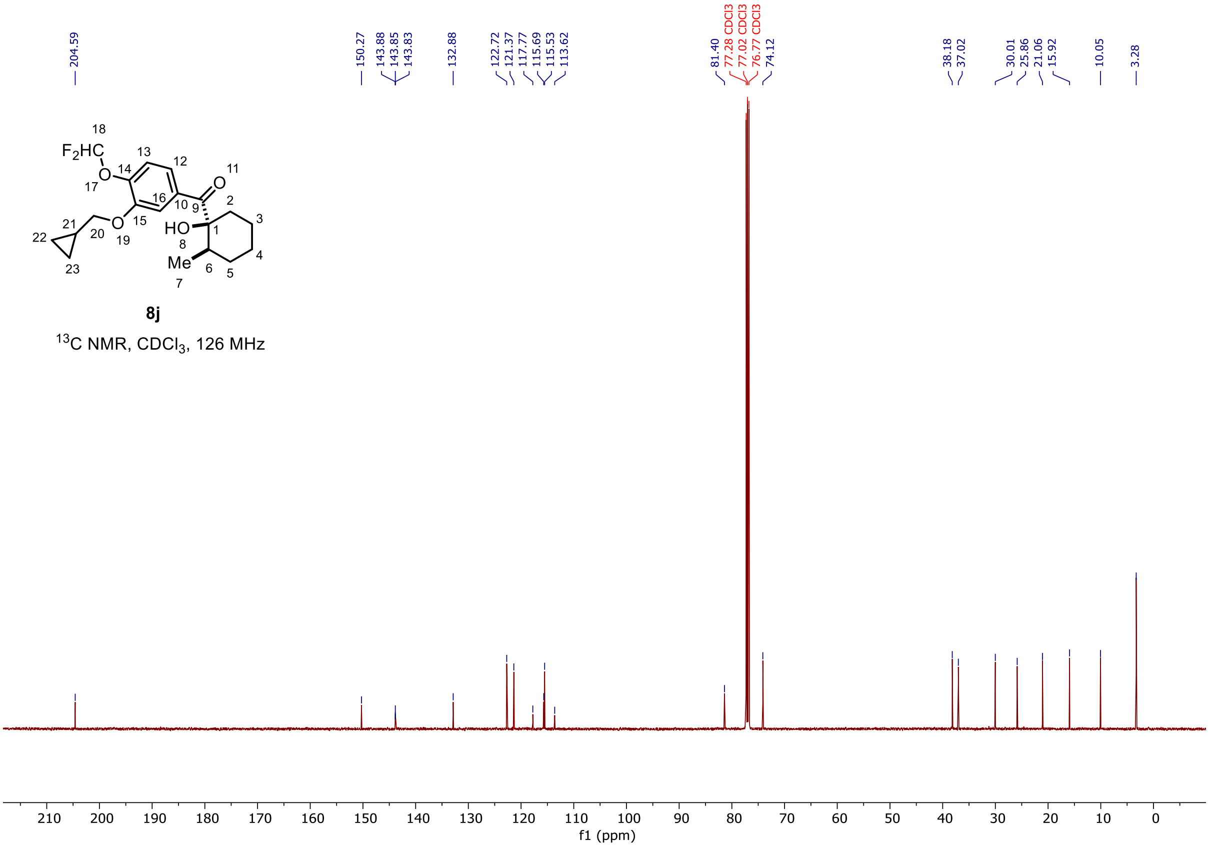

204.59
150.27
143.88
143.85
143.83
132.88
122.72
121.37
117.77
115.69
115.53
113.62
81.40
77.28 CDCl3
77.02 CDCl3
76.77 CDCl3
74.12
38.18
37.02
30.01
25.86
21.06
15.92
10.05
3.28
8j
^{13}C NMR, CDCl$_3$, 126 MHz
f1 (ppm)

(*trans*)-(3-Hydroxy-4-methyltetrahydro-2*H*-pyran-3-yl)(phenyl)methanone 8k

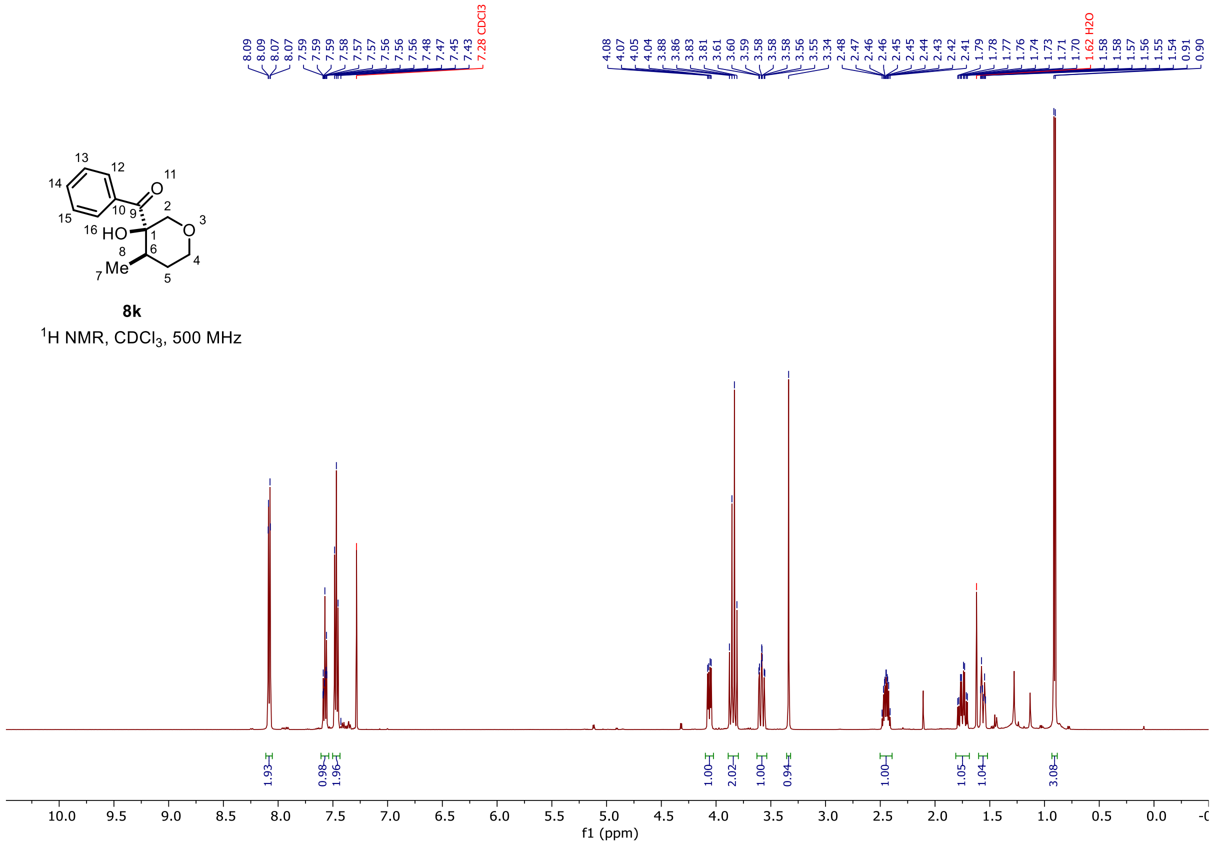

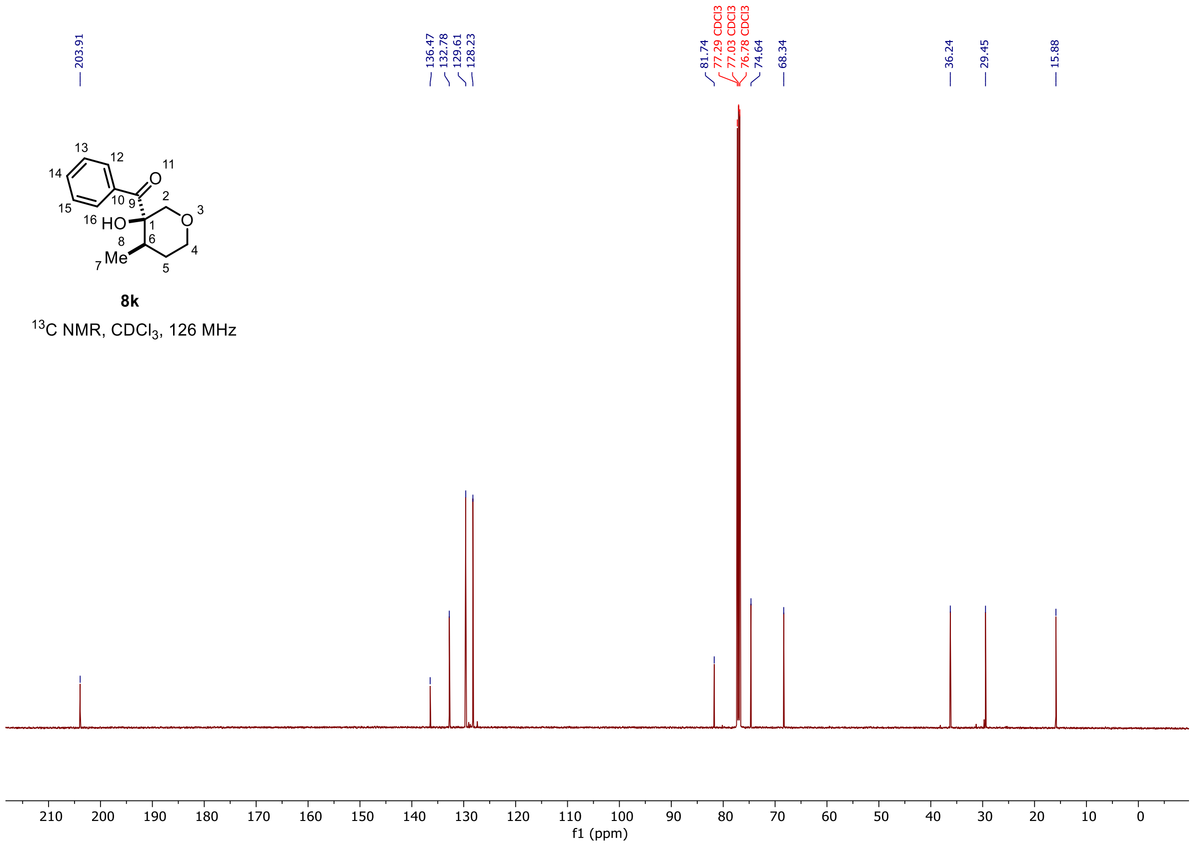

(1*R*,2*S*)-(2-Hydroxy-1-methyl-1,2,3,4-tetrahydronaphthalen-2-yl)(phenyl)methanone 8m

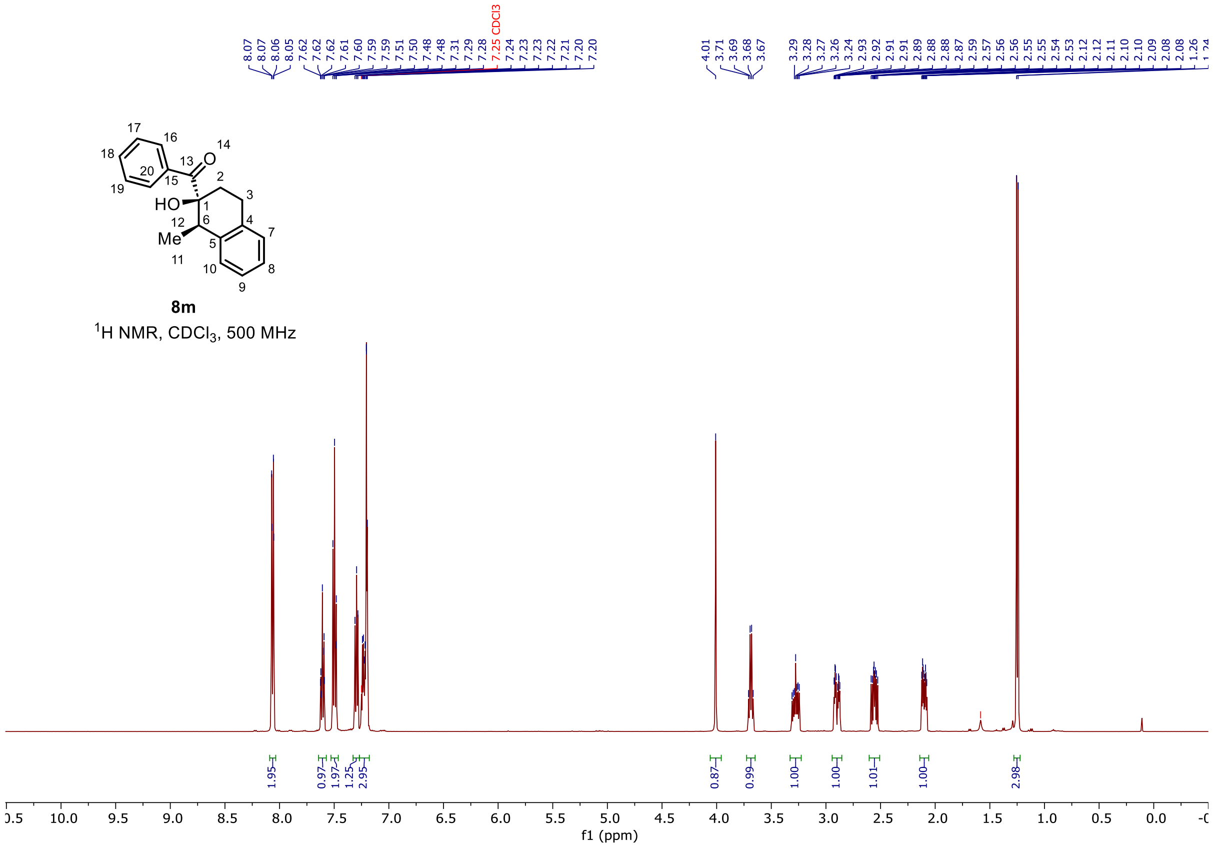

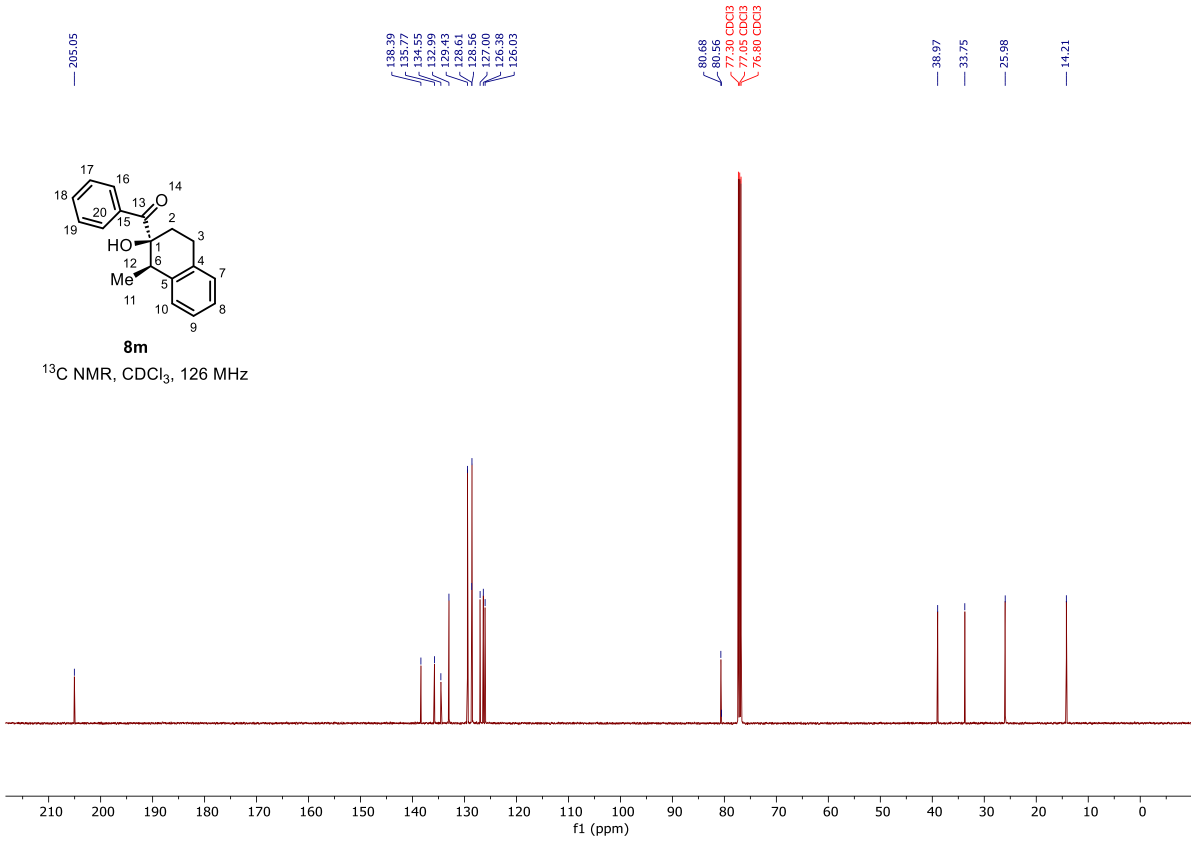

(1*R*,2*S*)-(2-Hydroxy-1-methyl-1,2,3,4-tetrahydronaphthalen-2-yl)(*p*-tolyl)methanone 8n

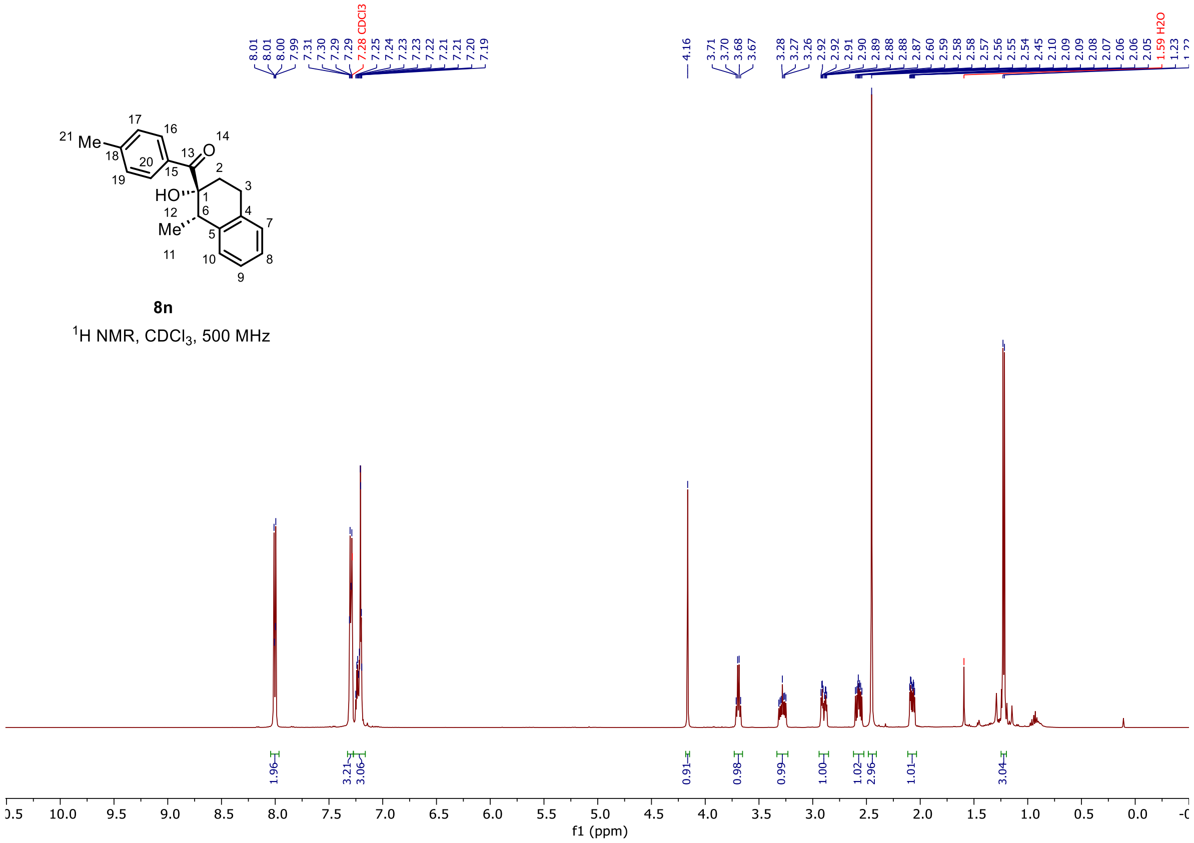

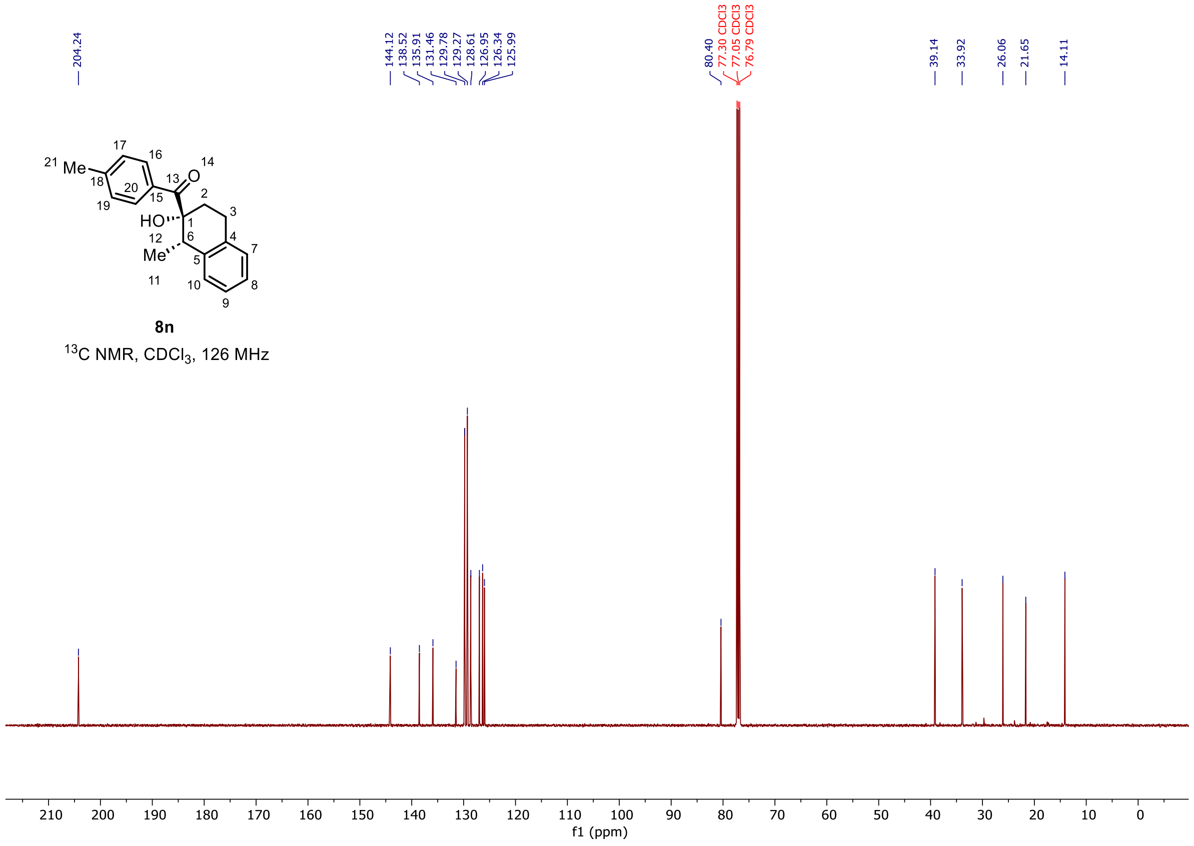

(4-Bromophenyl)-(1*R*,2*S*)-(2-hydroxy-1-methyl-1,2,3,4-tetrahydronaphthalen-2-yl)methanone 8o

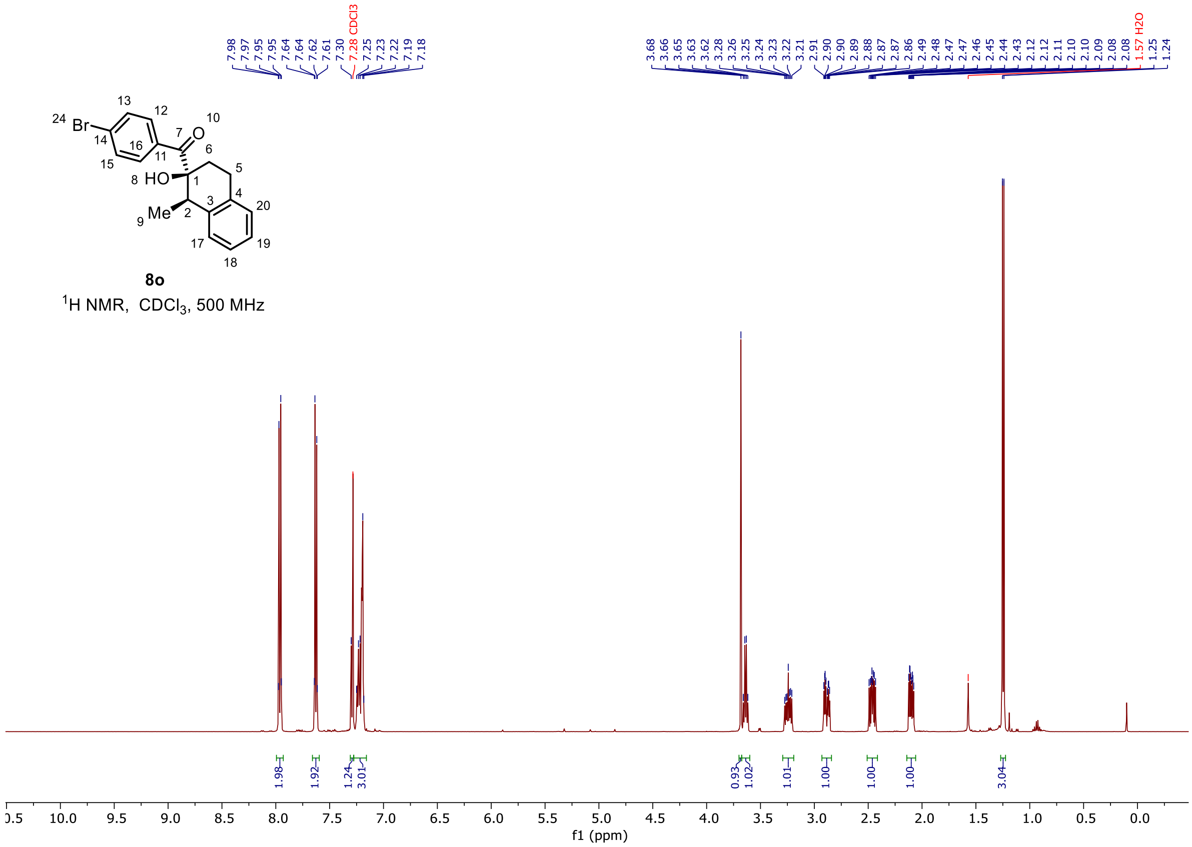

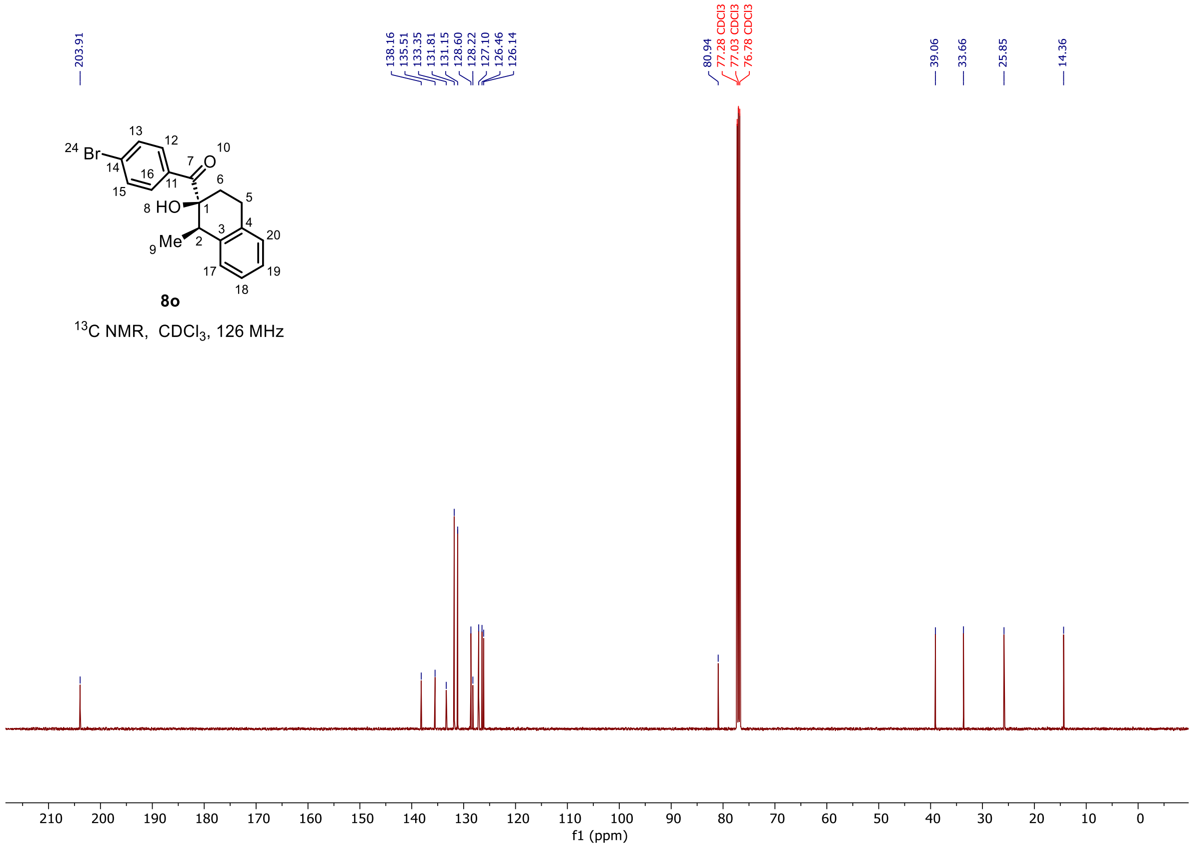

(1*R*,2*S*)-(2-Hydroxy-1-methyl-1,2,3,4-tetrahydronaphthalen-2-yl)(3-(trifluoromethyl)phenyl)methanone 8p

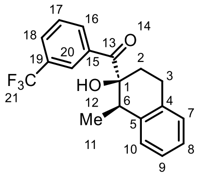

8p

^{1}H NMR, $CDCl_3$, 500 MHz

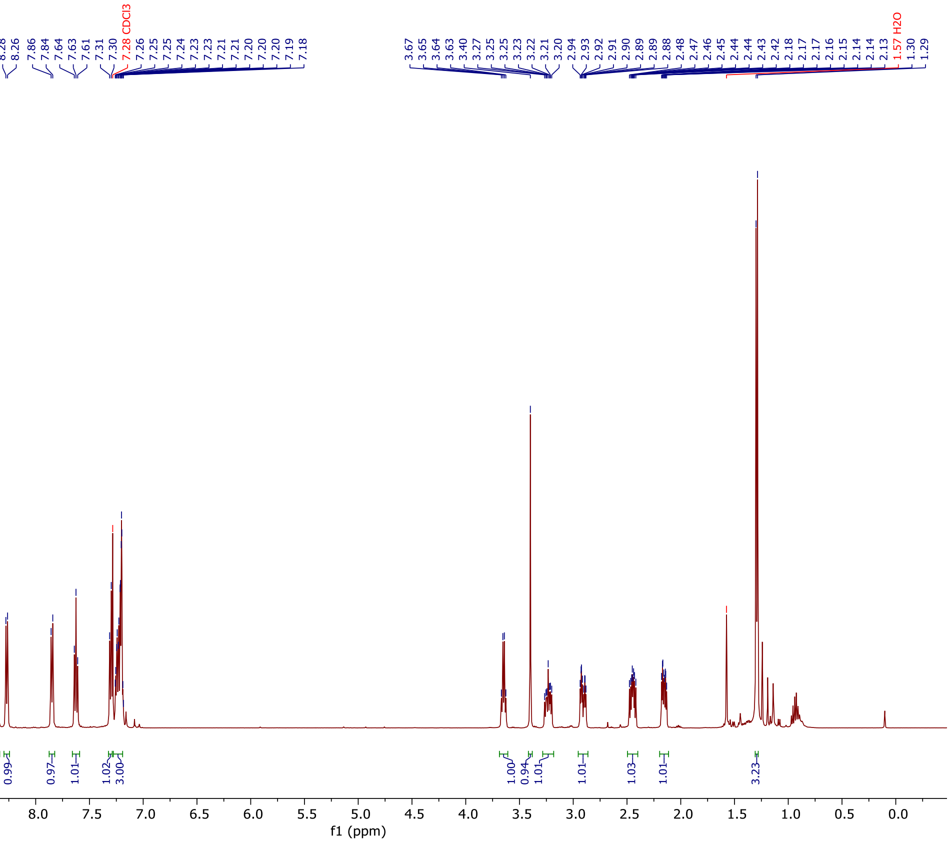

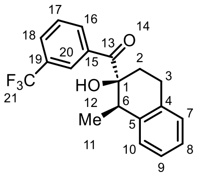

8p

^{19}F NMR, $CDCl_3$, 470 MHz

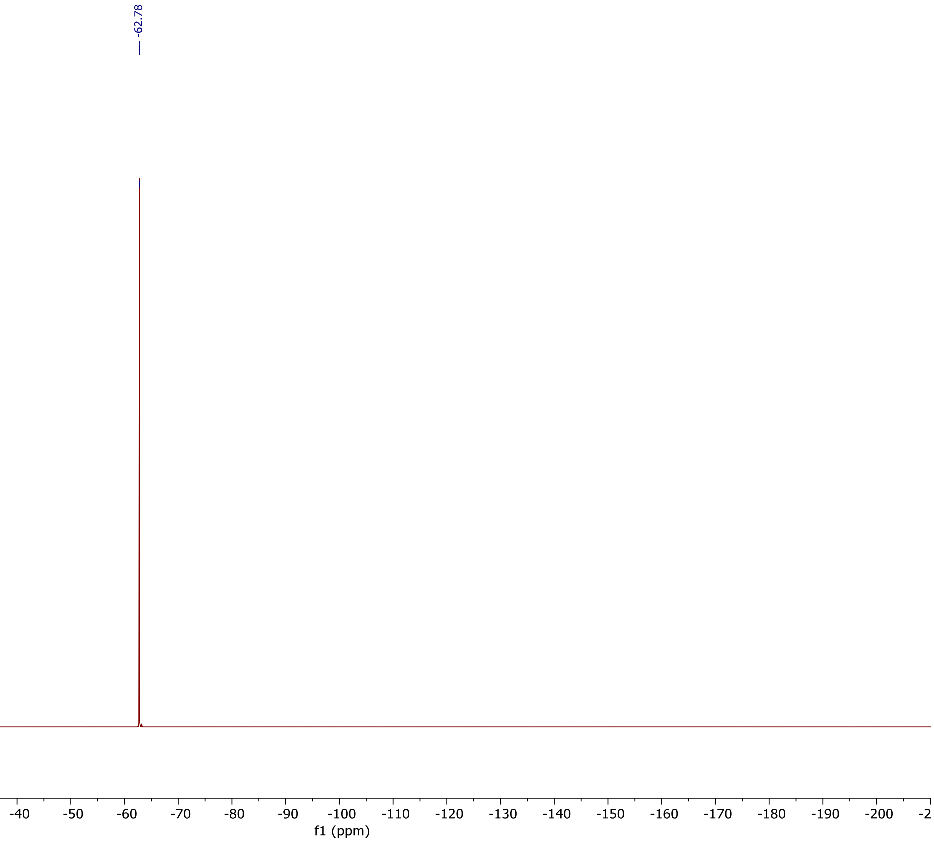

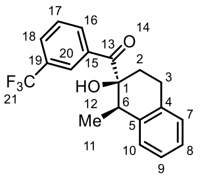

8p

^{13}C NMR, $CDCl_3$, 126 MHz

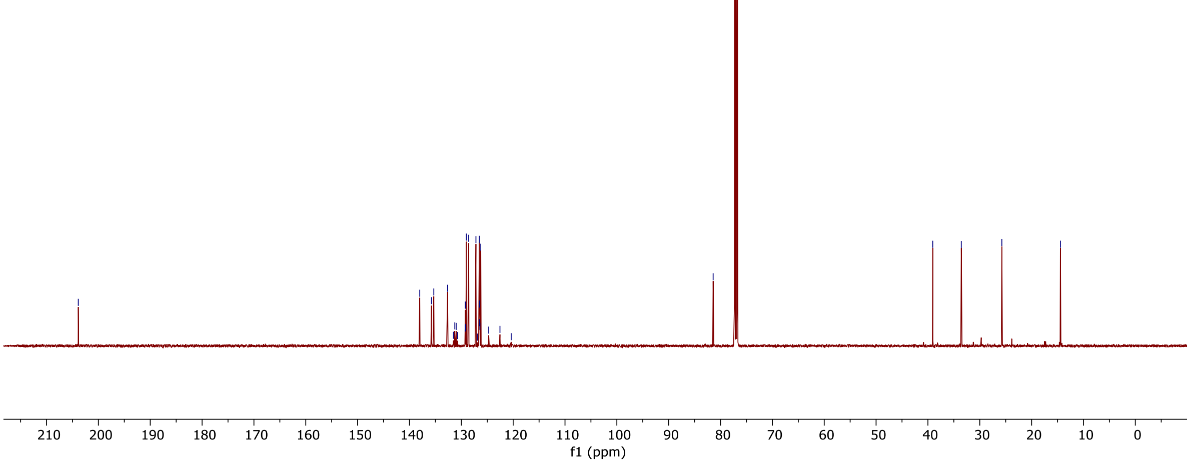

(1S,2*R*)-1-Methyl-2-(3-(trifluoromethyl)benzoyl)-1,2,3,4-tetrahydronaphthalen-2-yl 3,5-dinitrobenzoate 8p-Bz(NO$_2$)$_2$

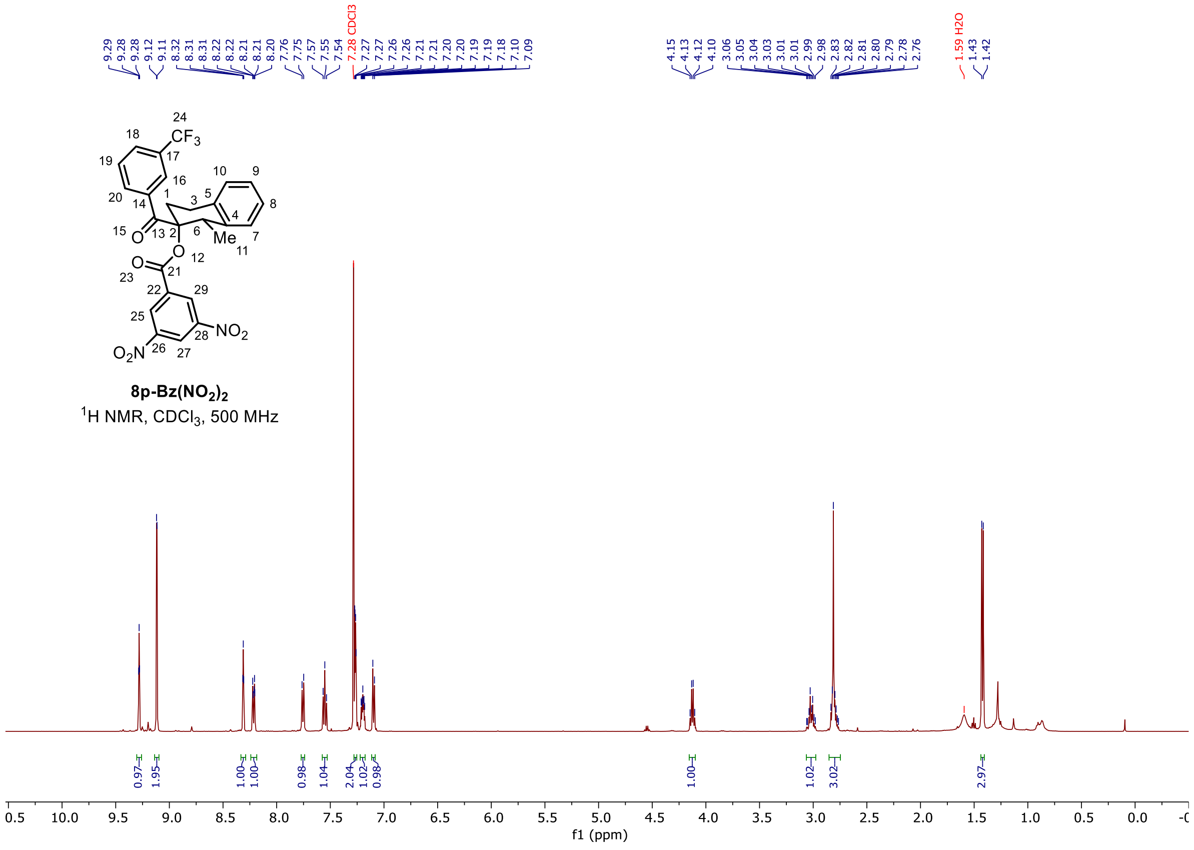

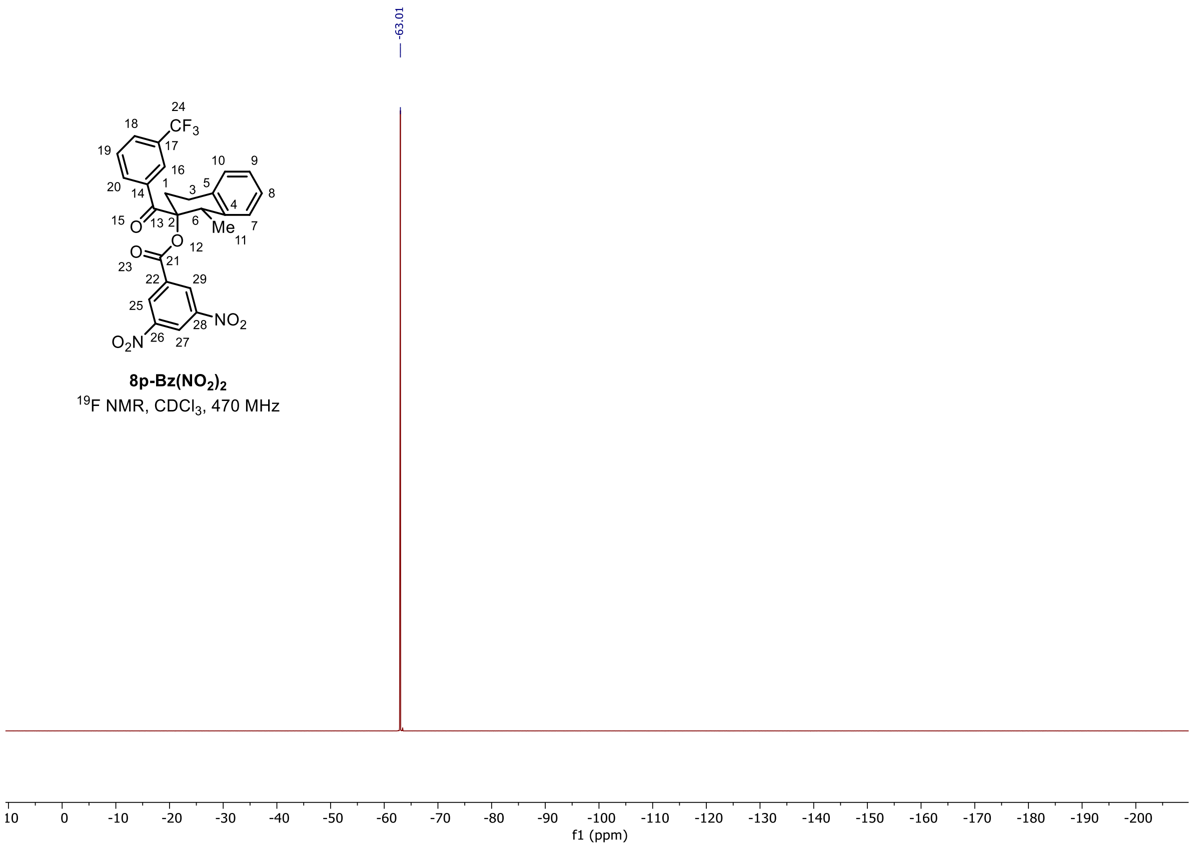

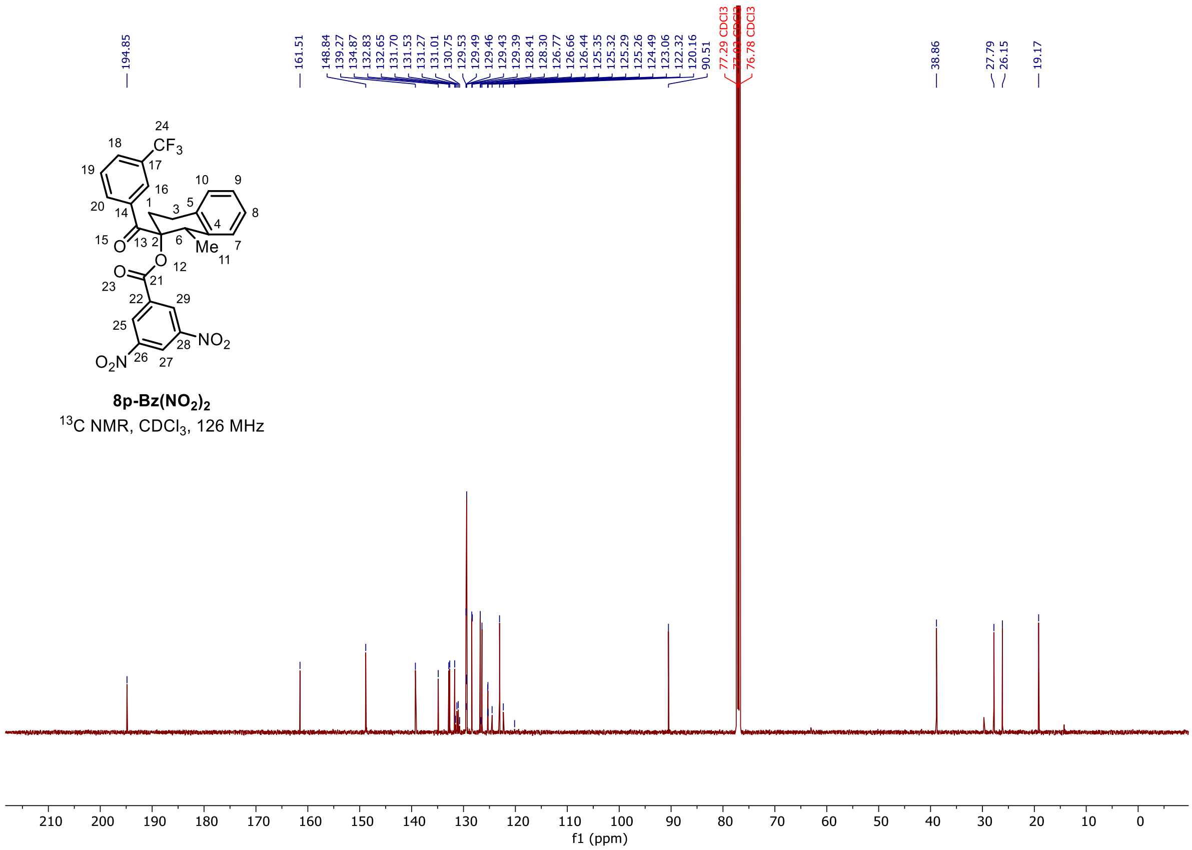
8p-Bz(NO2)2
13C NMR, CDCl3, 126 MHz
194.85
161.51
148.84
139.27
134.87
132.83
132.65
131.70
131.53
131.27
131.01
130.75
129.53
129.49
129.46
129.43
129.39
128.41
128.30
126.77
126.66
126.44
125.35
125.32
125.29
125.26
124.49
123.06
122.32
120.16
90.51
77.29 CDCl3
76.78 CDCl3
38.86
27.79
26.15
19.17
f1 (ppm)

(1*R*,2*S*)-(2-Hydroxy-1-methyl-1,2,3,4-tetrahydronaphthalen-2-yl)(3-methoxyphenyl)methanone 8q

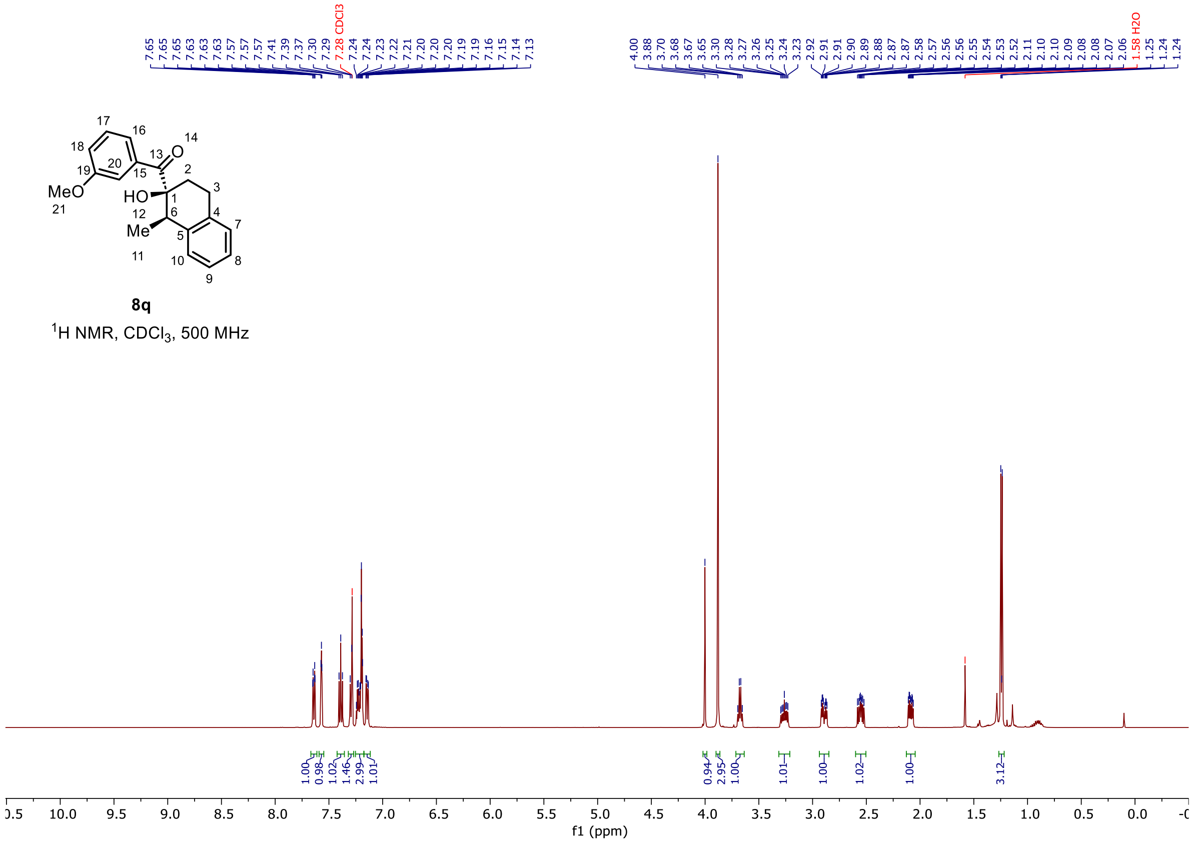

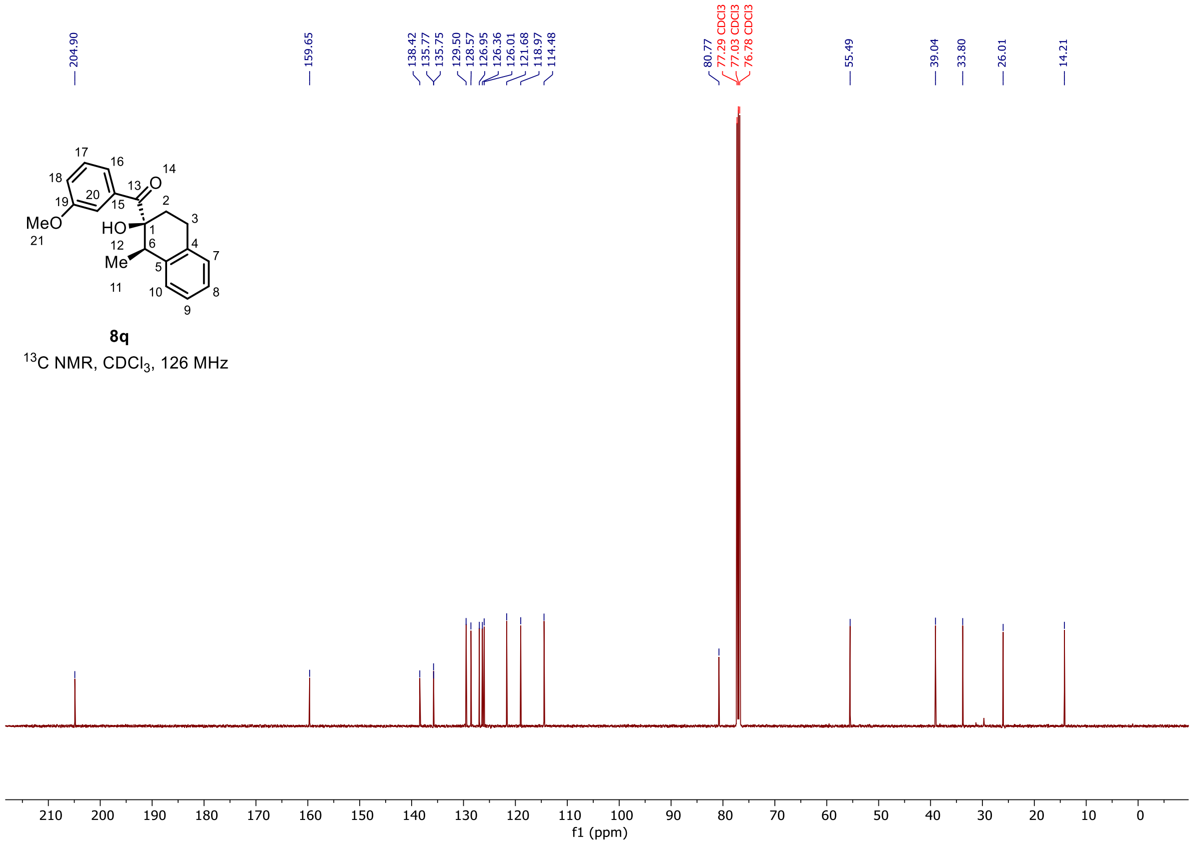

(2-Fluorophenyl)-(1*R*,2*S*)-(2-hydroxy-1-methyl-1,2,3,4-tetrahydronaphthalen-2-yl)methanone 8r

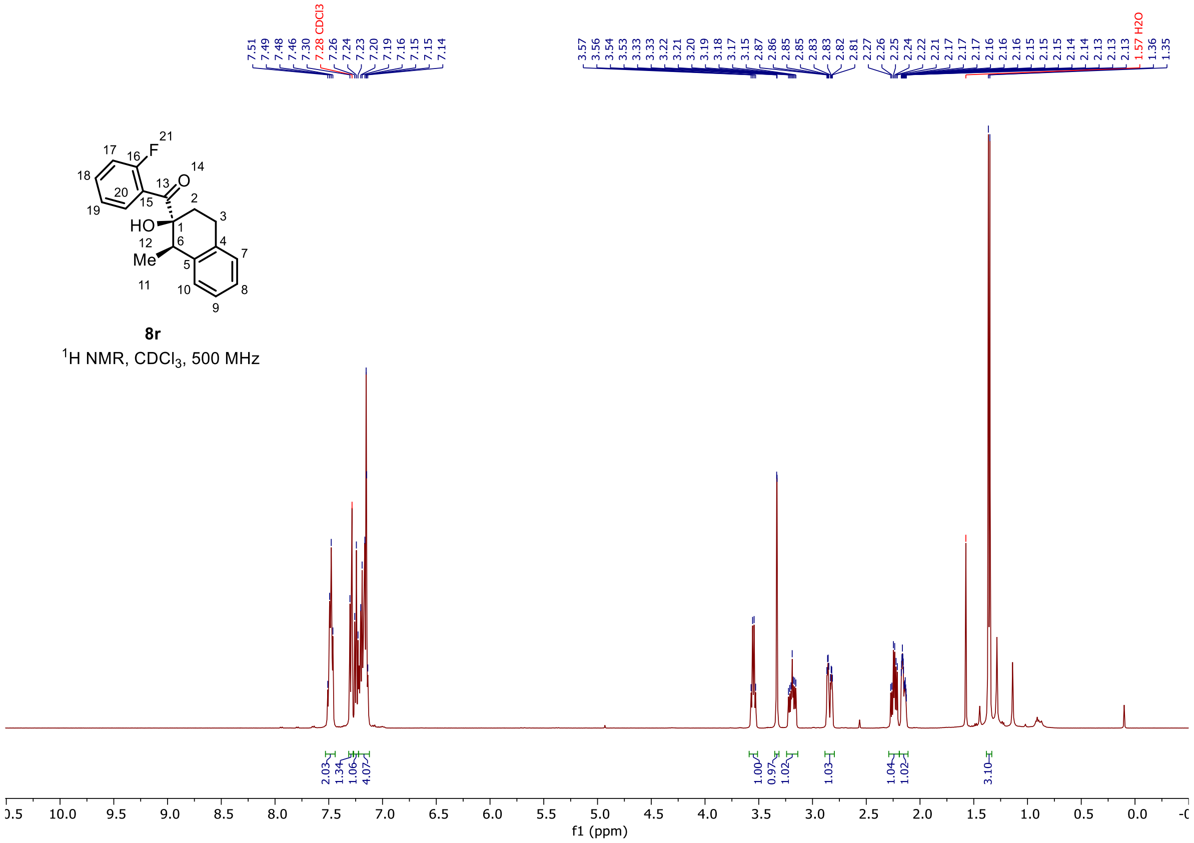

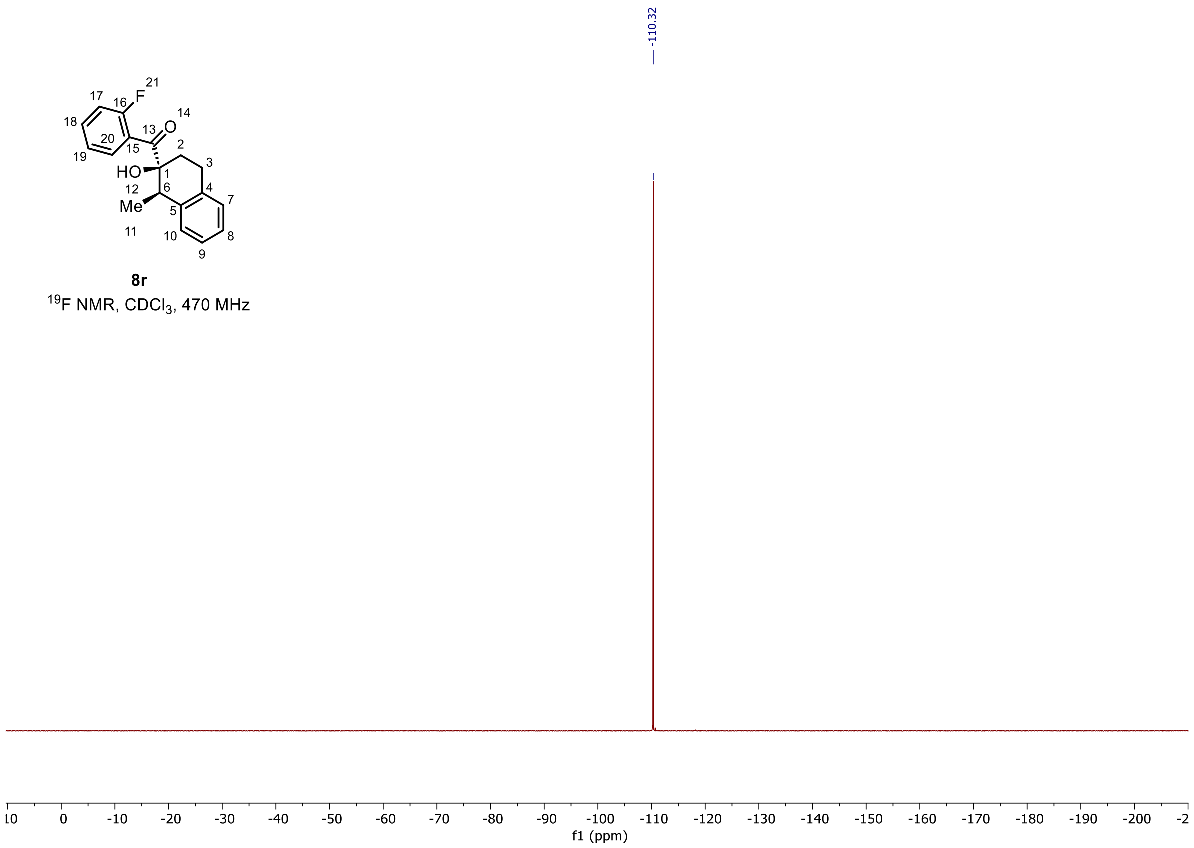

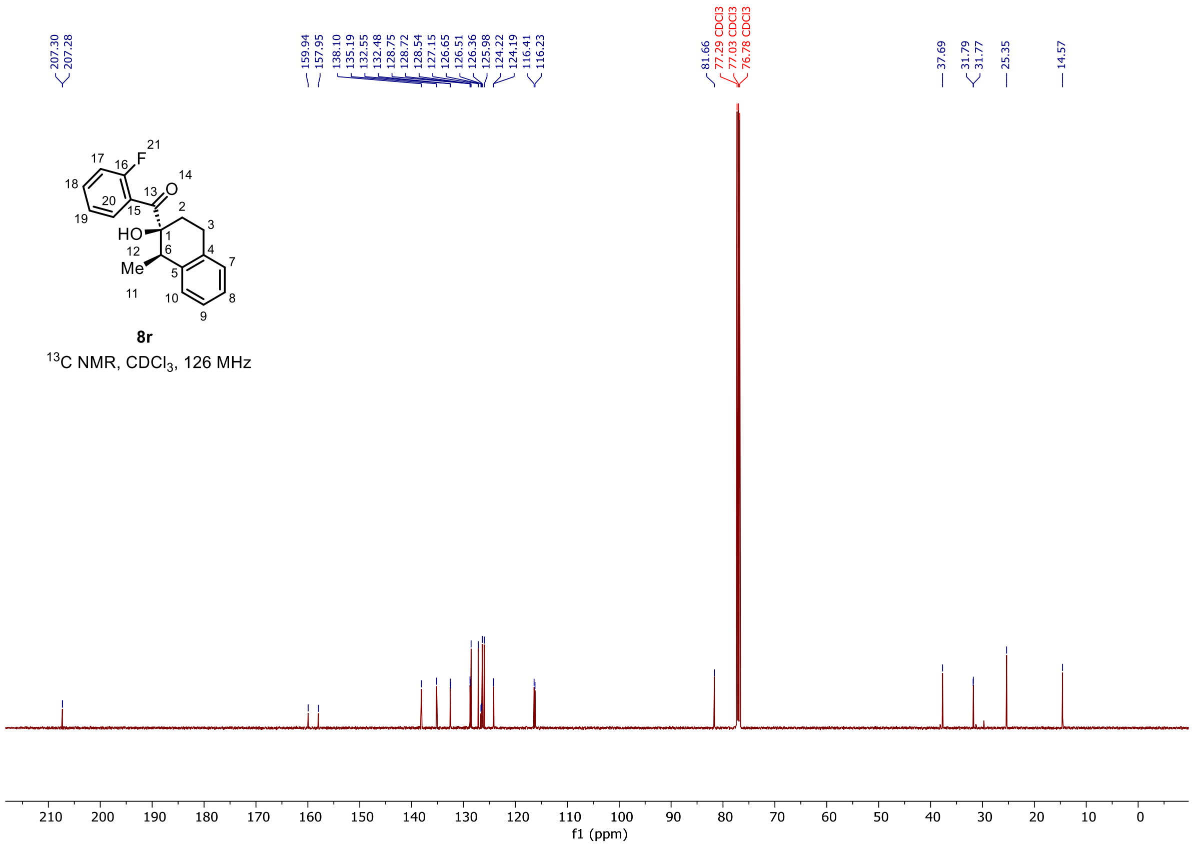
207.30
207.28
159.94
157.95
138.10
135.19
132.55
132.48
128.75
128.72
128.54
127.15
126.65
126.51
126.36
125.98
124.22
124.19
116.41
116.23
81.66
77.29 CDCl3
77.03 CDCl3
76.78 CDCl3
37.69
31.79
31.77
25.35
14.57
8r
^{13}C NMR, CDCl$_3$, 126 MHz
f1 (ppm)

(1*R*,2*S*)-(2-Hydroxy-1-methyl-1,2,3,4-tetrahydronaphthalen-2-yl)(naphthalen-2-yl)methanone 8s

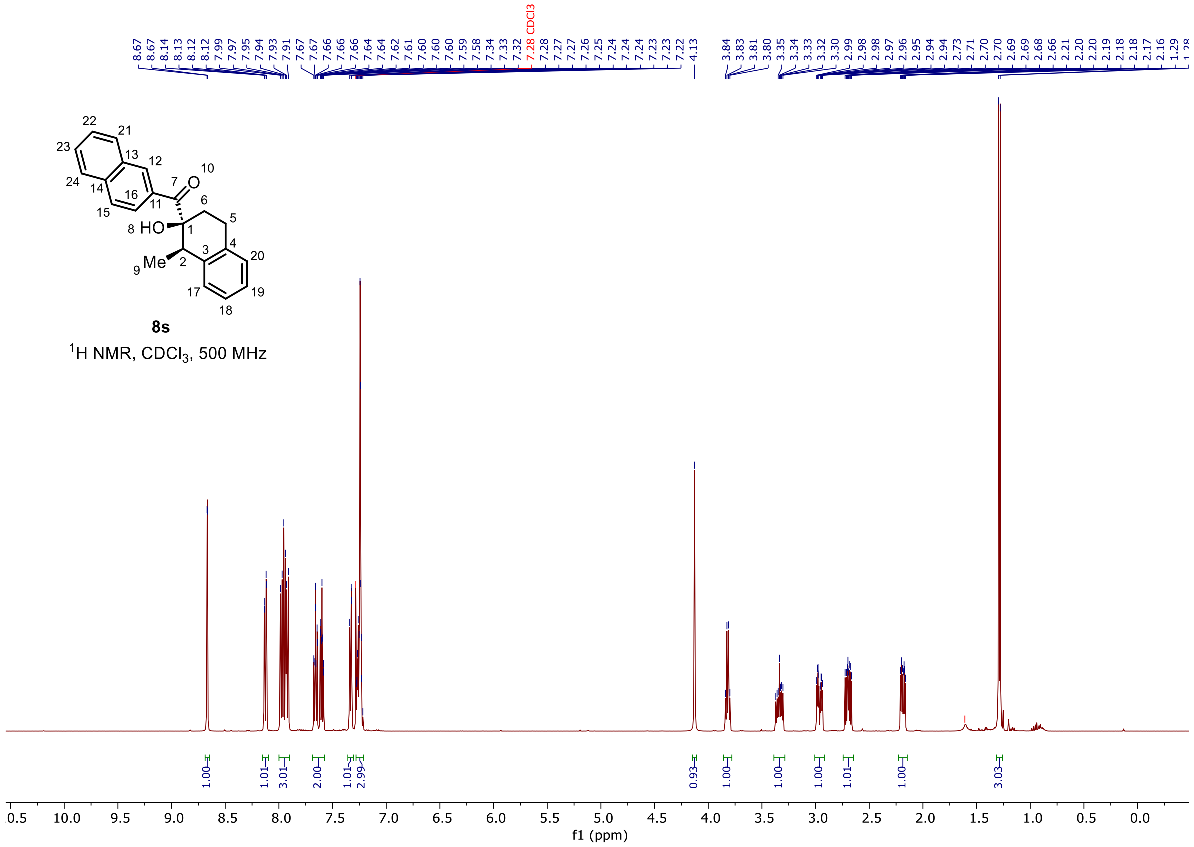

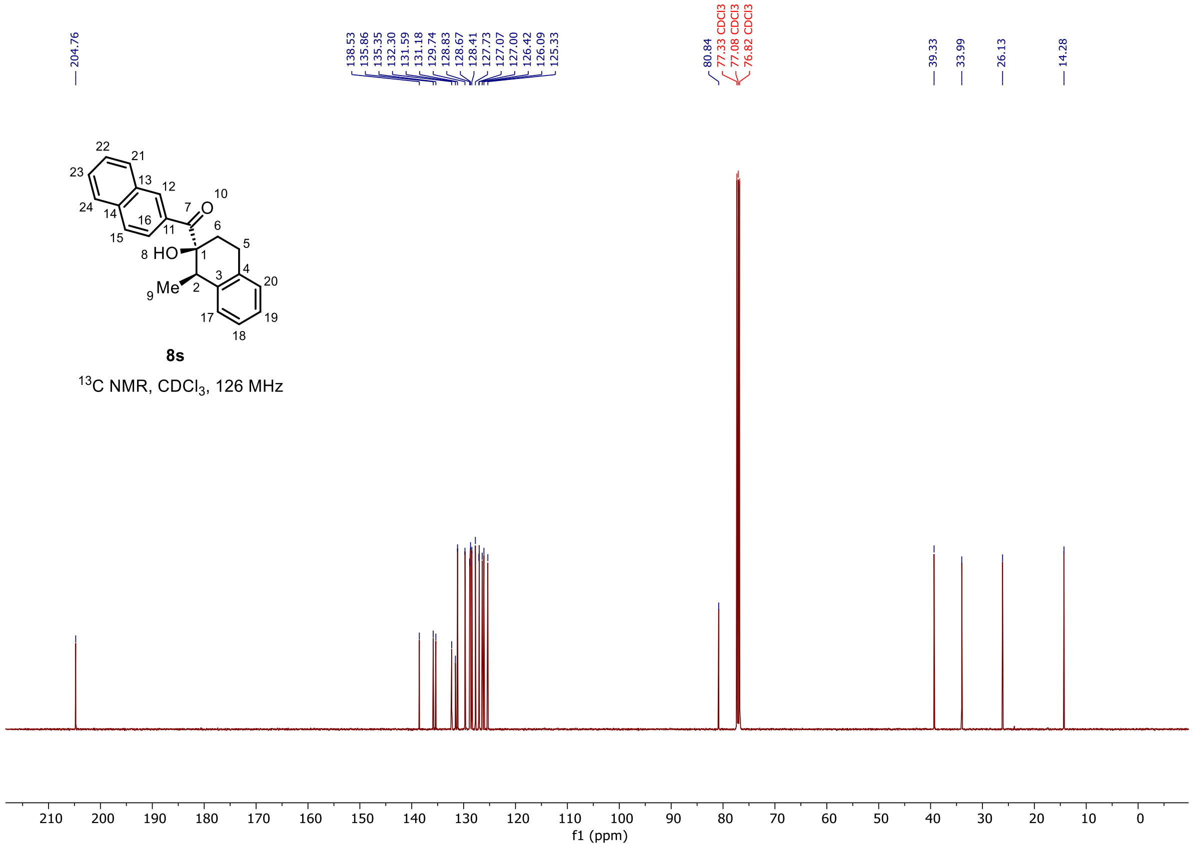

(Furan-2-yl)-(1*R*,2*S*)-(2-Hydroxy-1-methyl-1,2,3,4-tetrahydronaphthalen-2-yl)methanone 8t

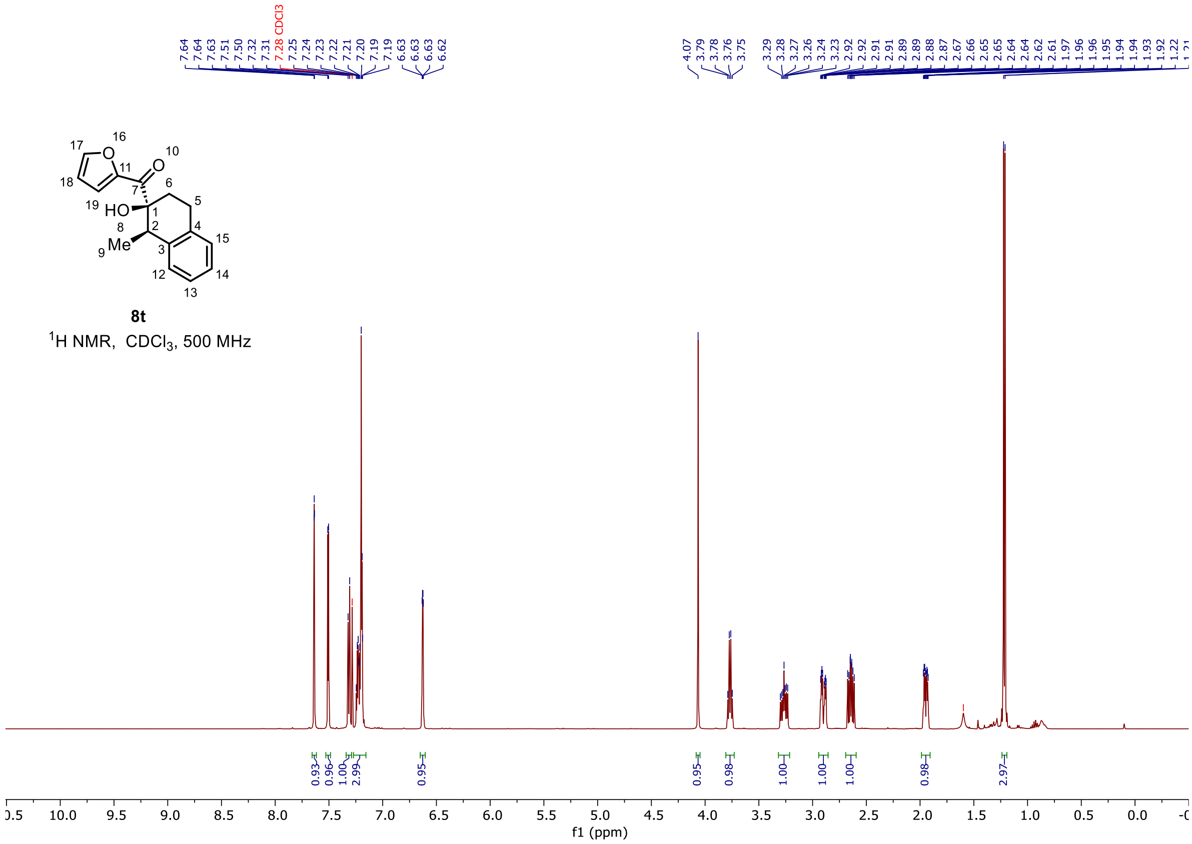

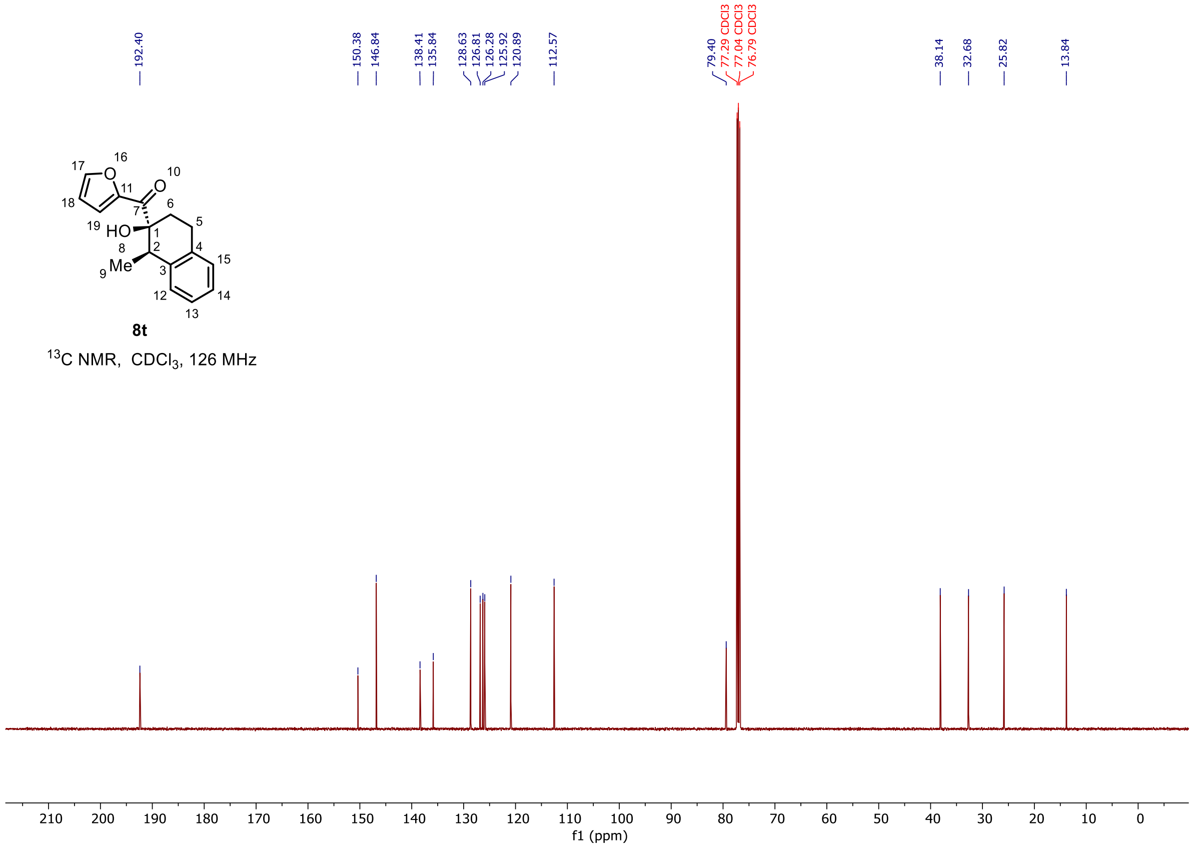

(*trans)*-(2-Hydroxy-1-methyl-1,2,3,4-tetrahydronaphthalen-2-yl)(3-(trifluoromethyl)phenyl)methanone 12

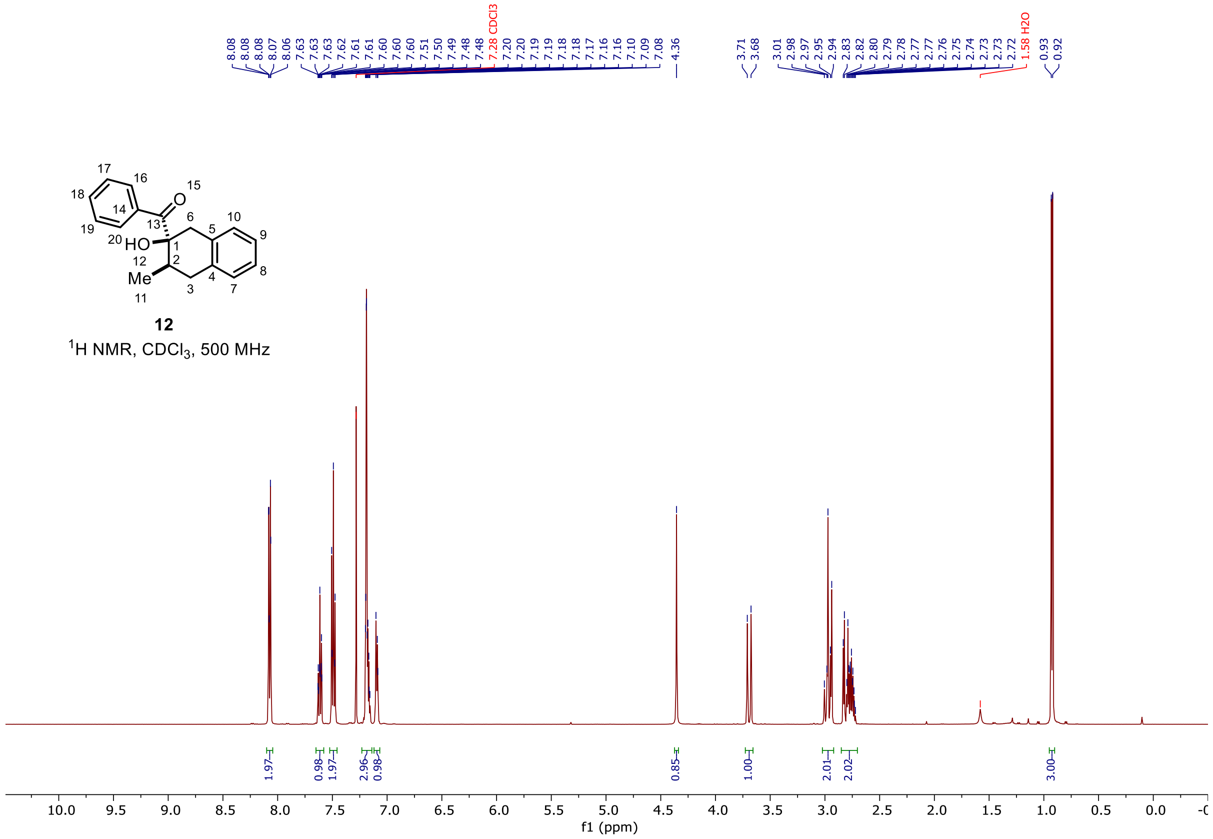

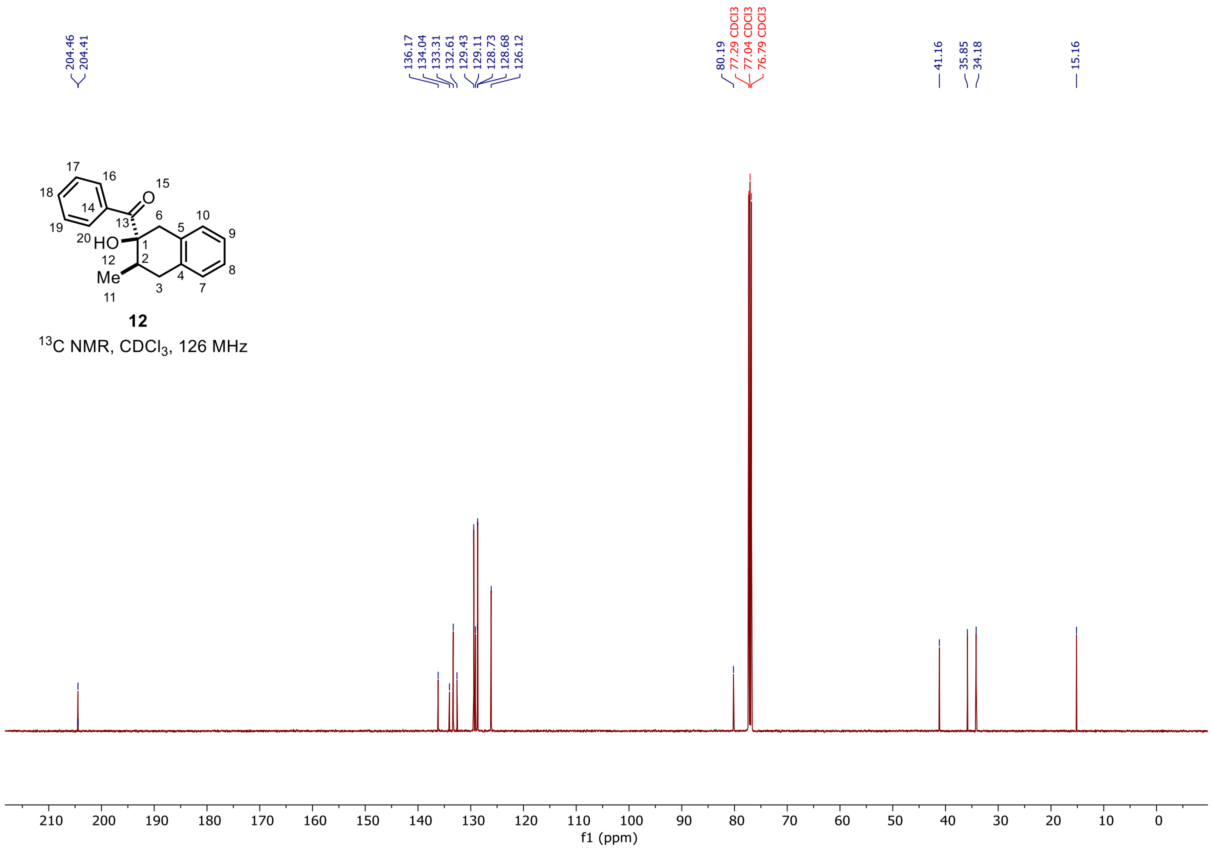

(trans)-2-Hydroxy-3-methyl-3,4-dihydronaphthalen-1(2H)-one 14

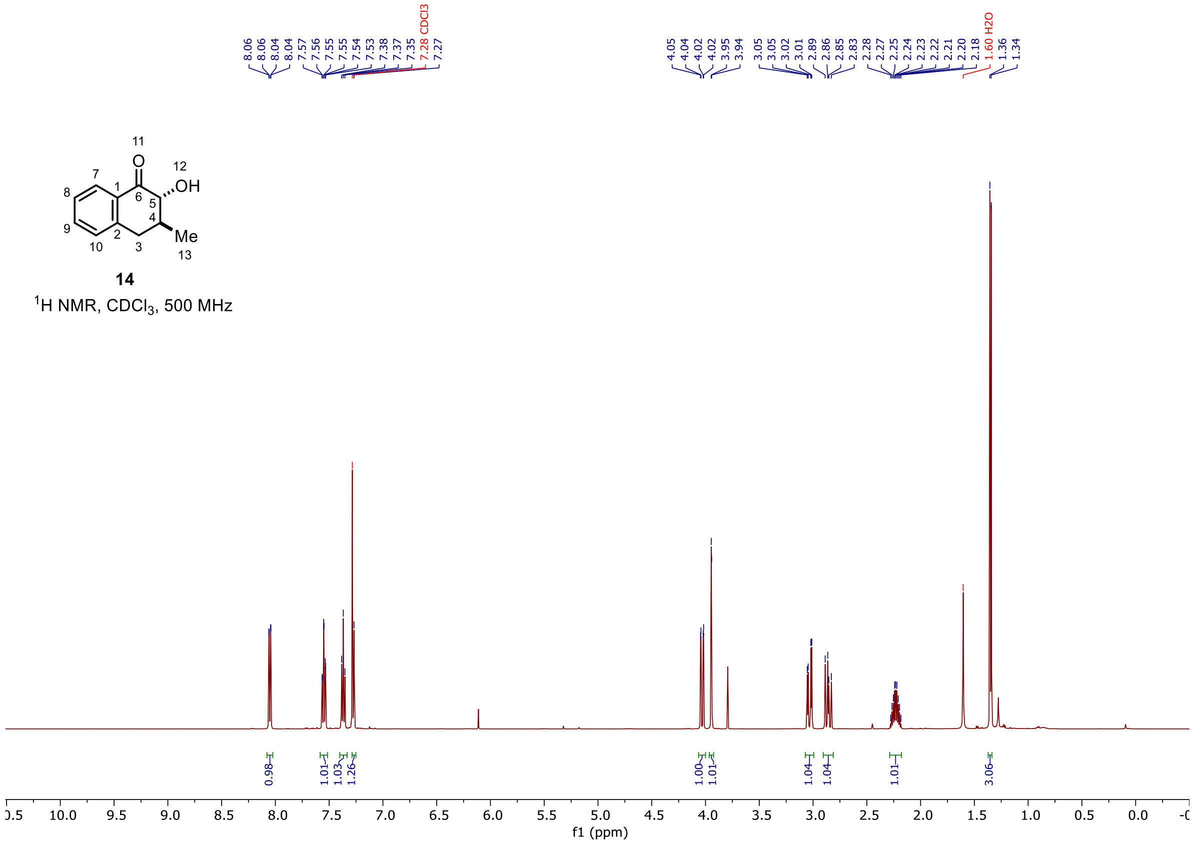

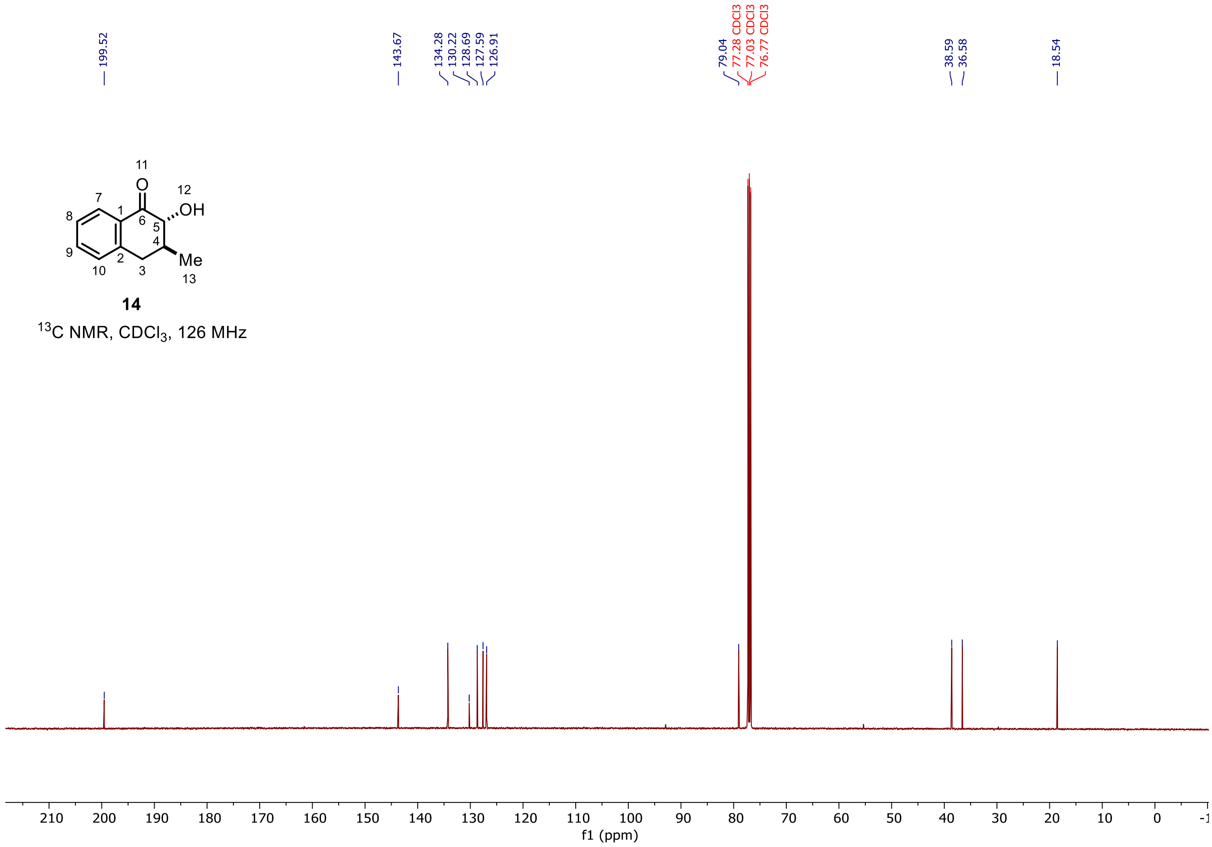

4-Methyl-3-phenylnaphthalen-2-ol 17

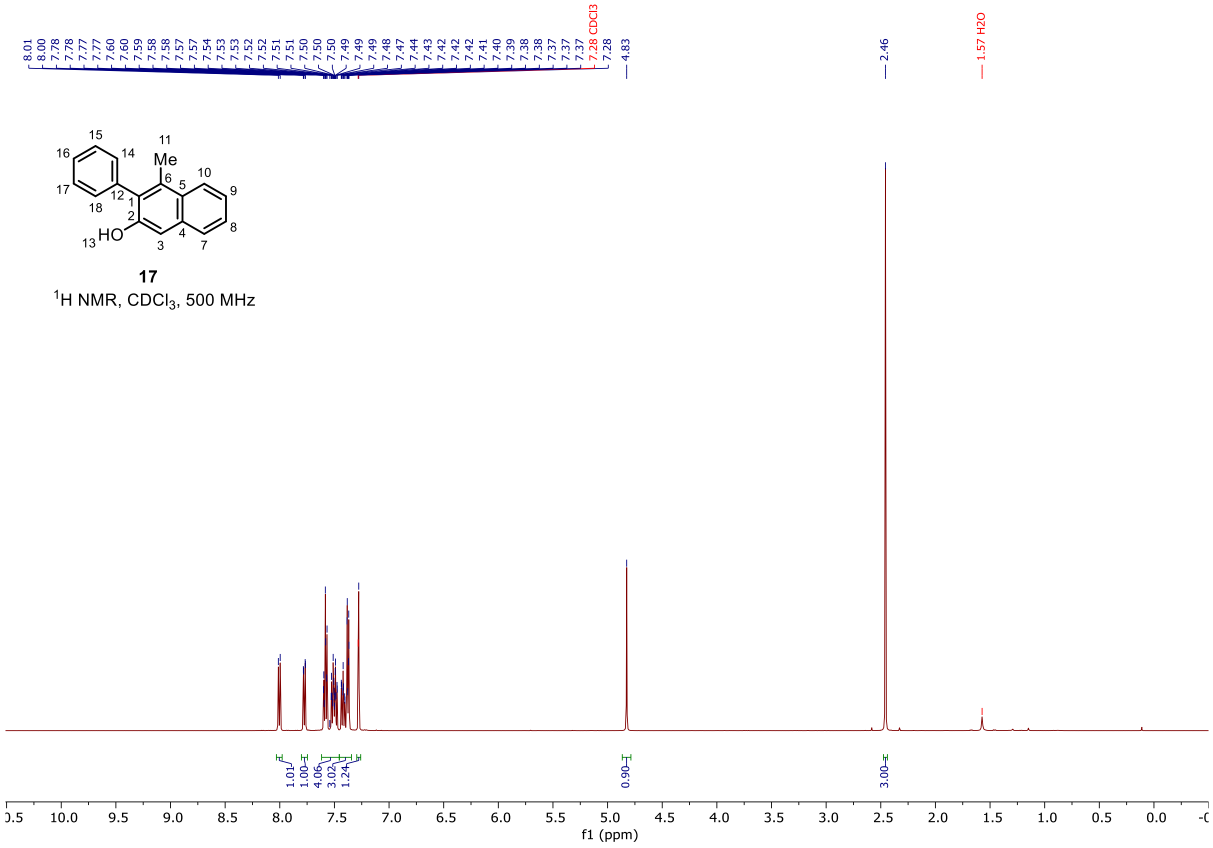

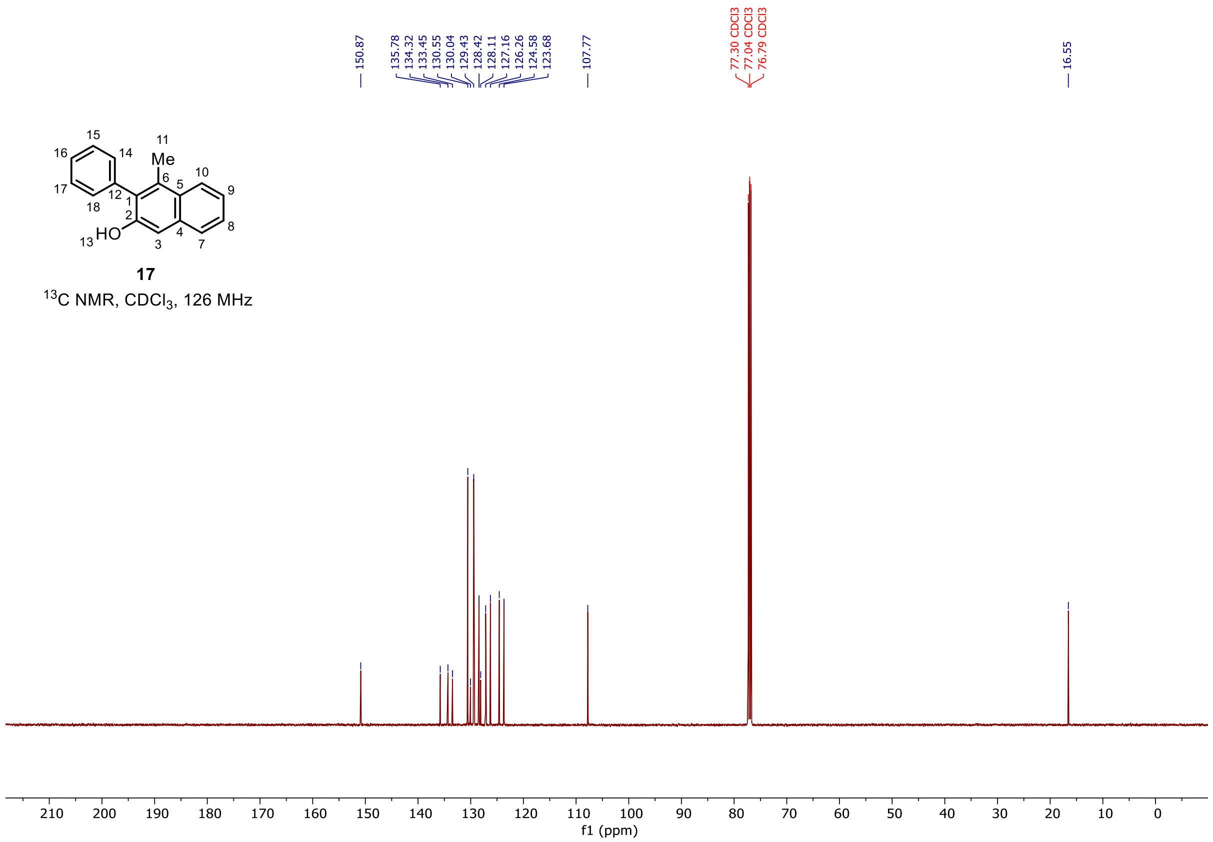

1-Methyl-3-phenylnaphthalen-2-ol *iso*-17

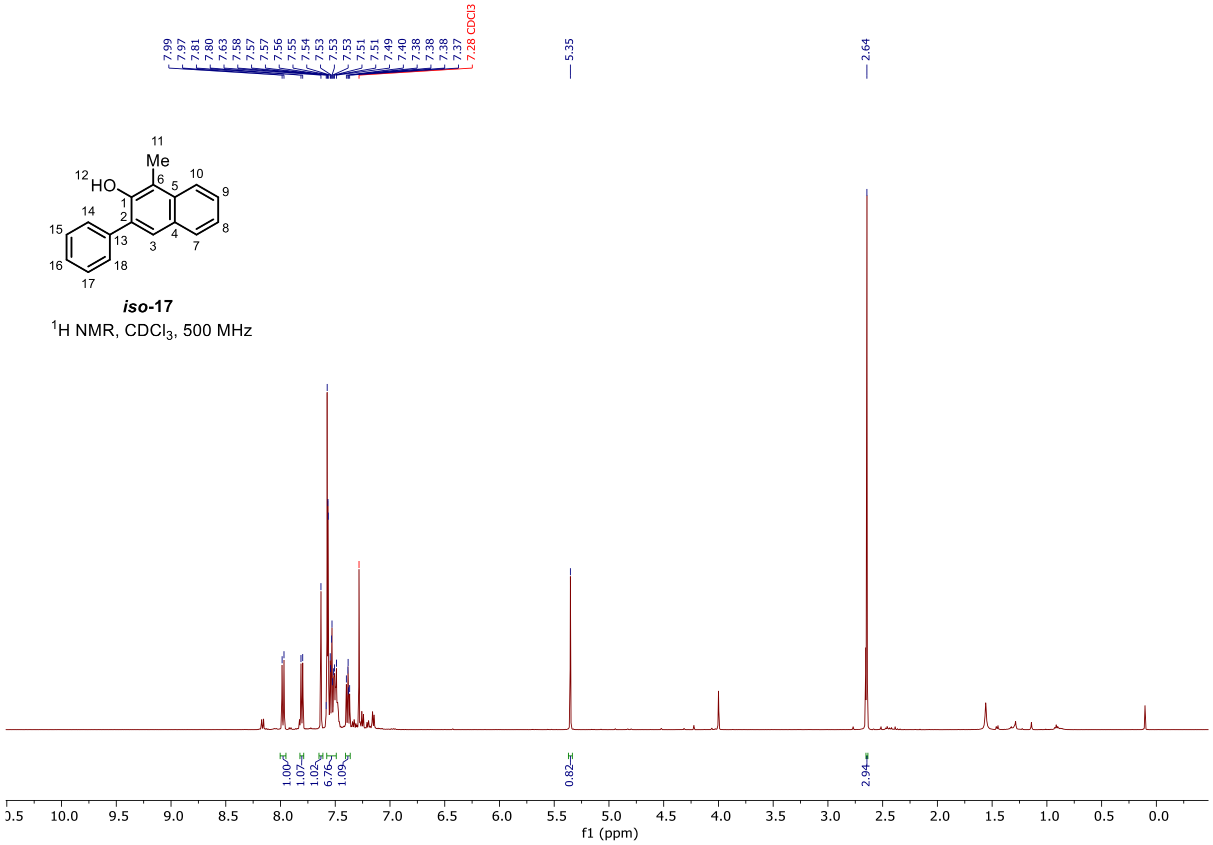

Derivatizations

(trans)-3-Methyl-1-phenylcyclohexane-1,2-diol s31

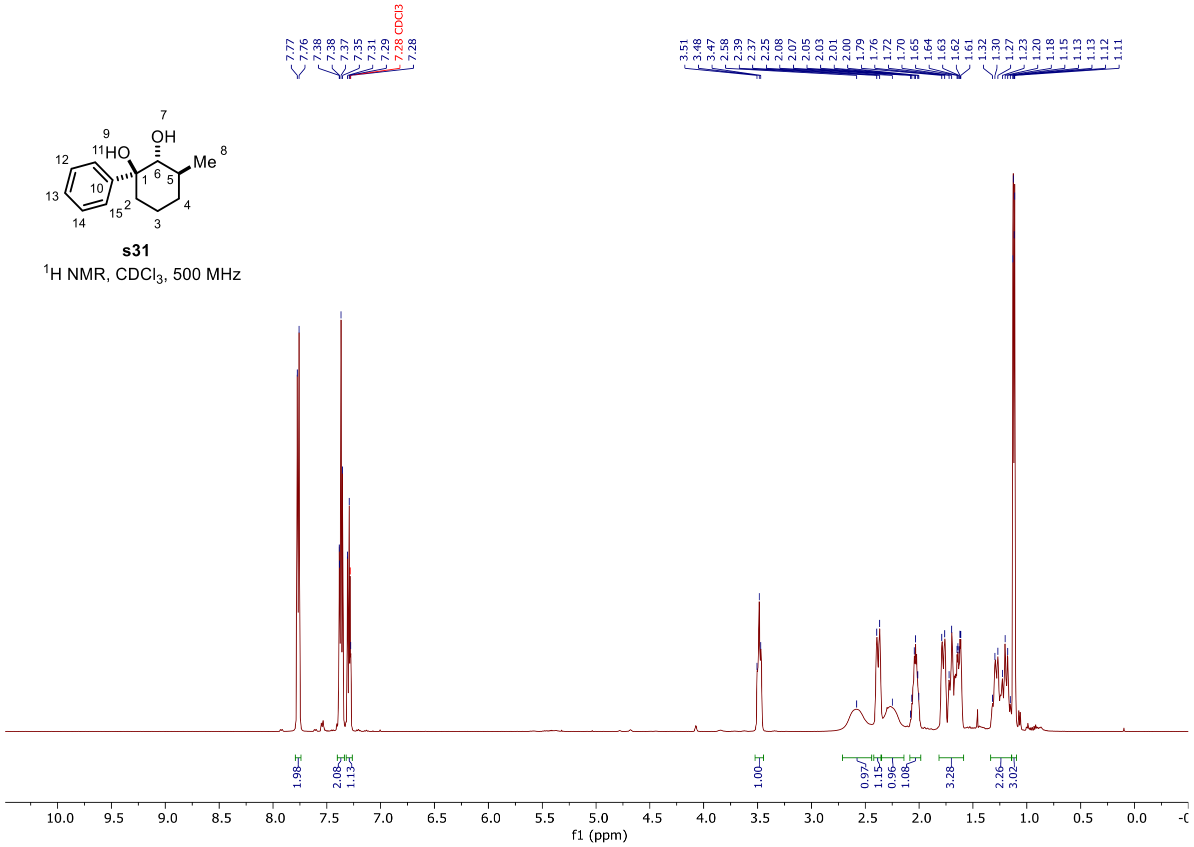

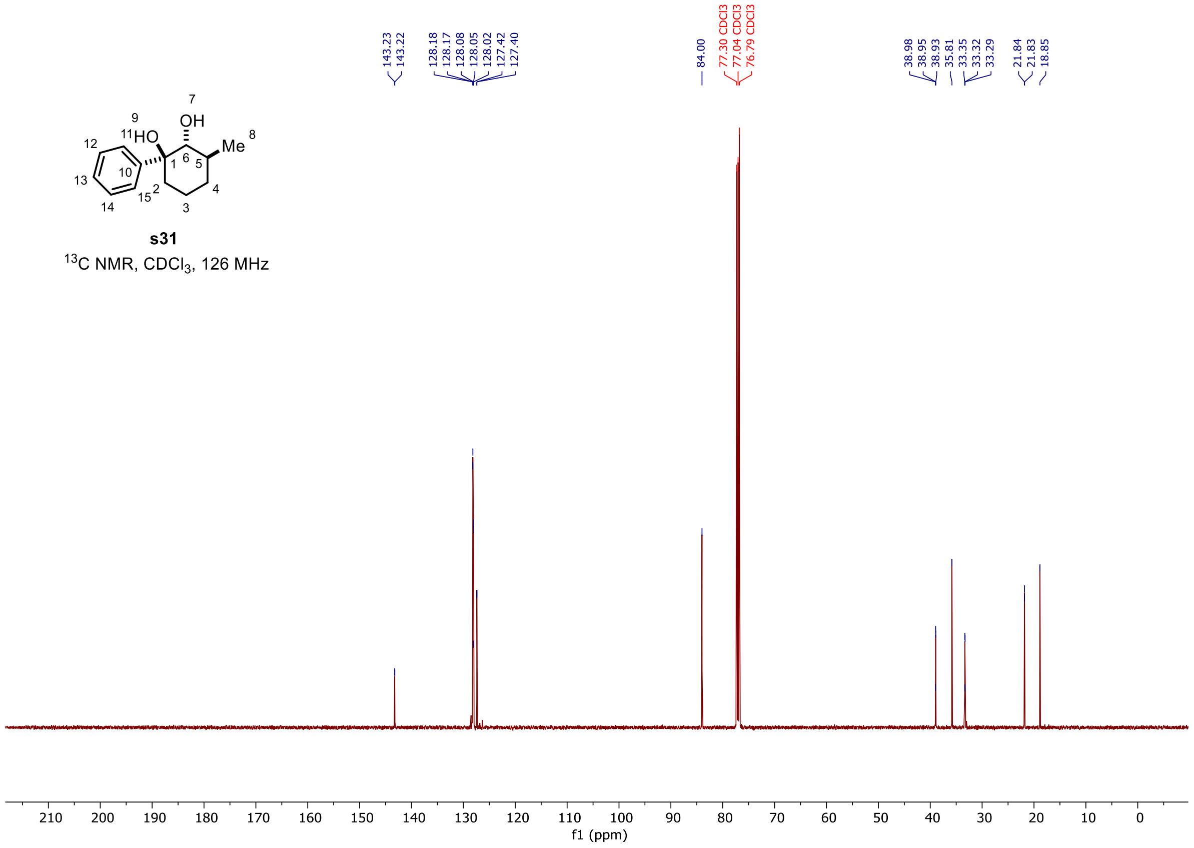

(*1R,2S*)-2-((*R*)-Hydroxy(phenyl)methyl)-1-methyl-1,2,3,4-tetrahydronaphthalen-2-ol s32-major

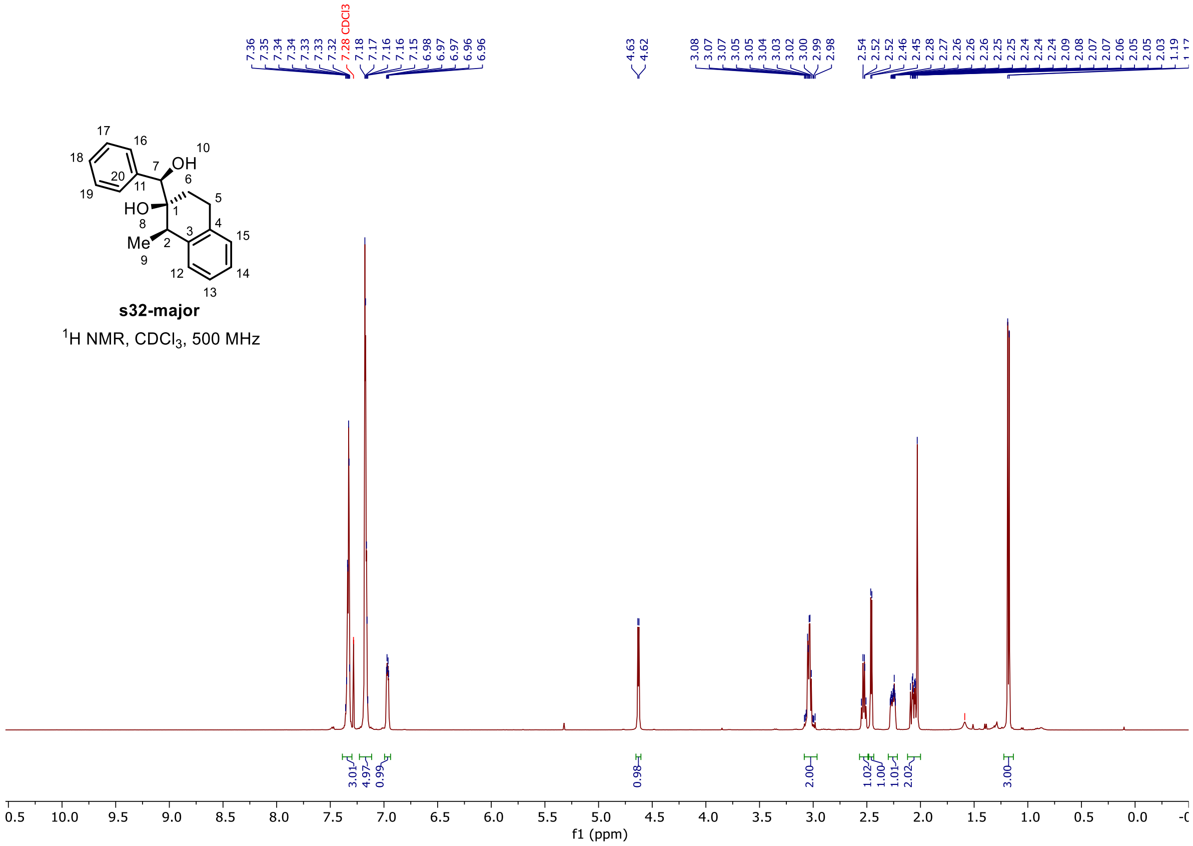

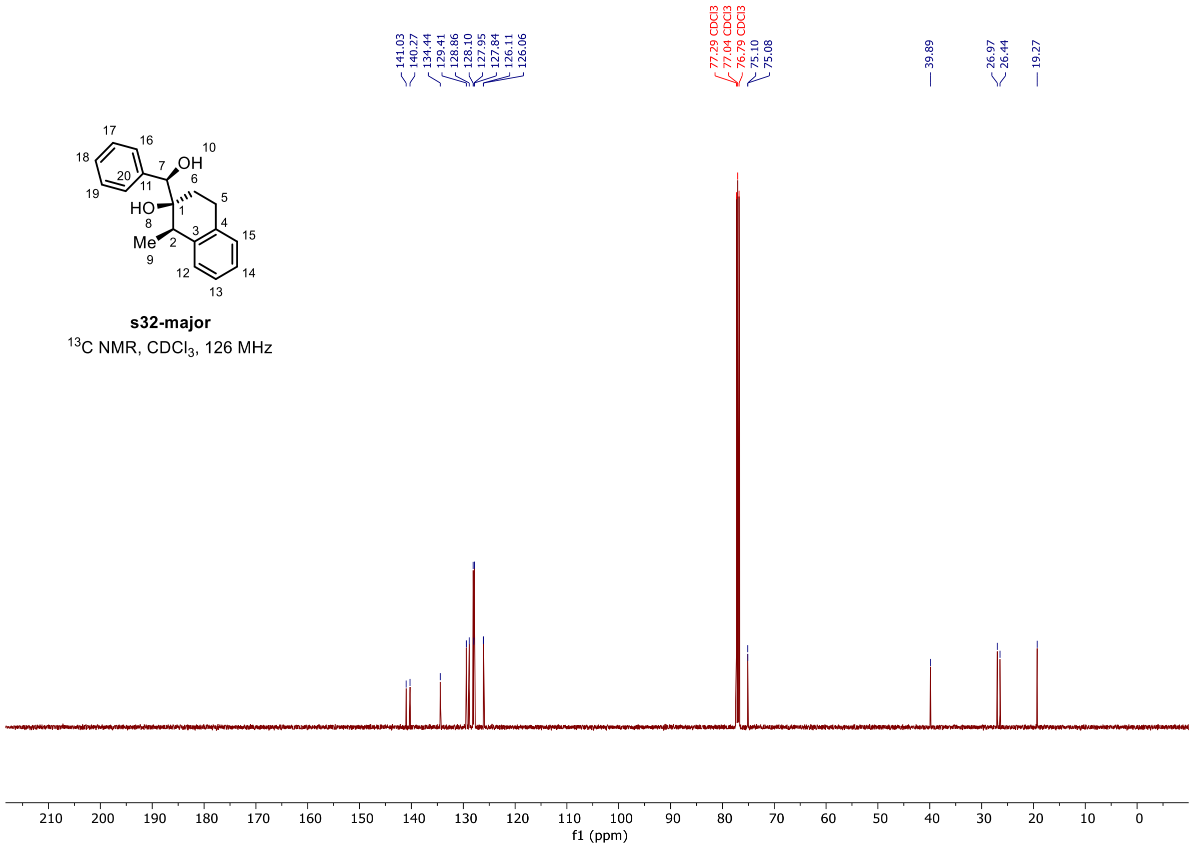

(1*R*,2*S*)-2-((*S*)-Hydroxy(phenyl)methyl)-1-methyl-1,2,3,4-tetrahydronaphthalen-2-ol s32-minor

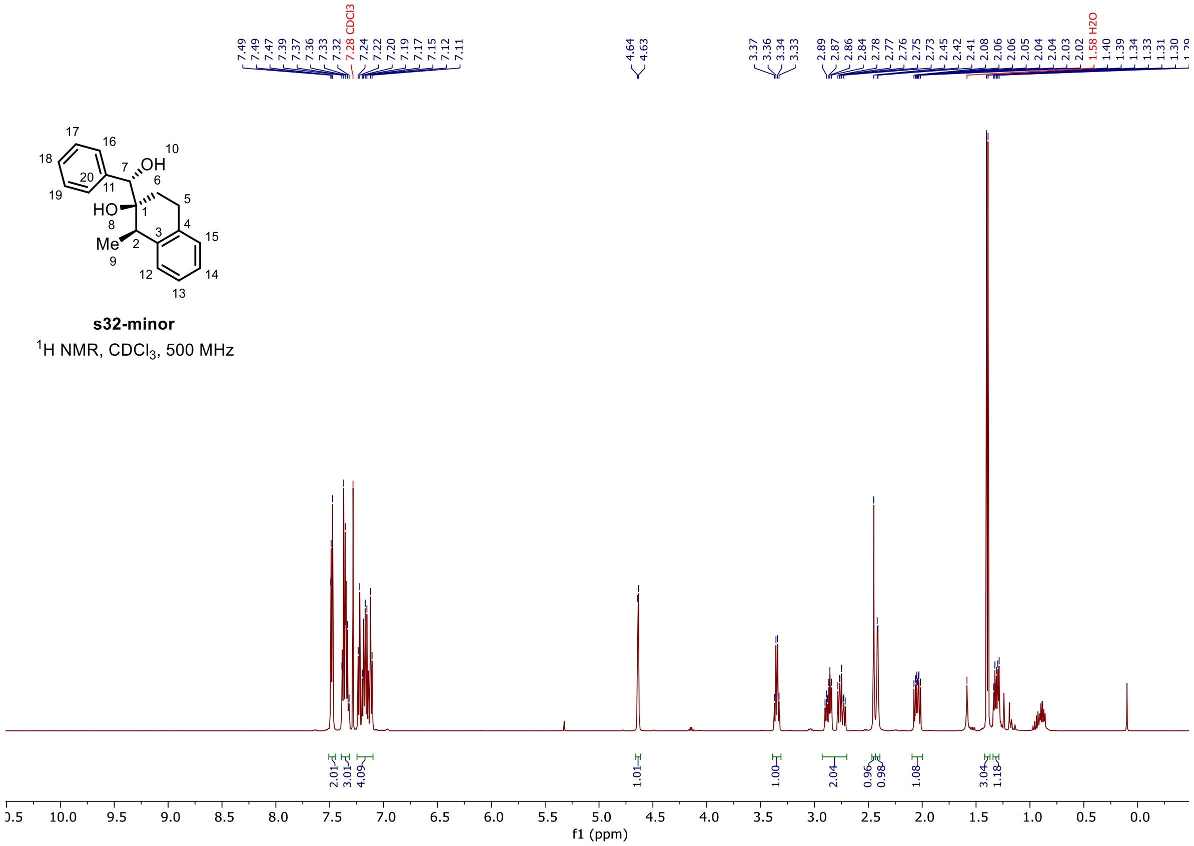

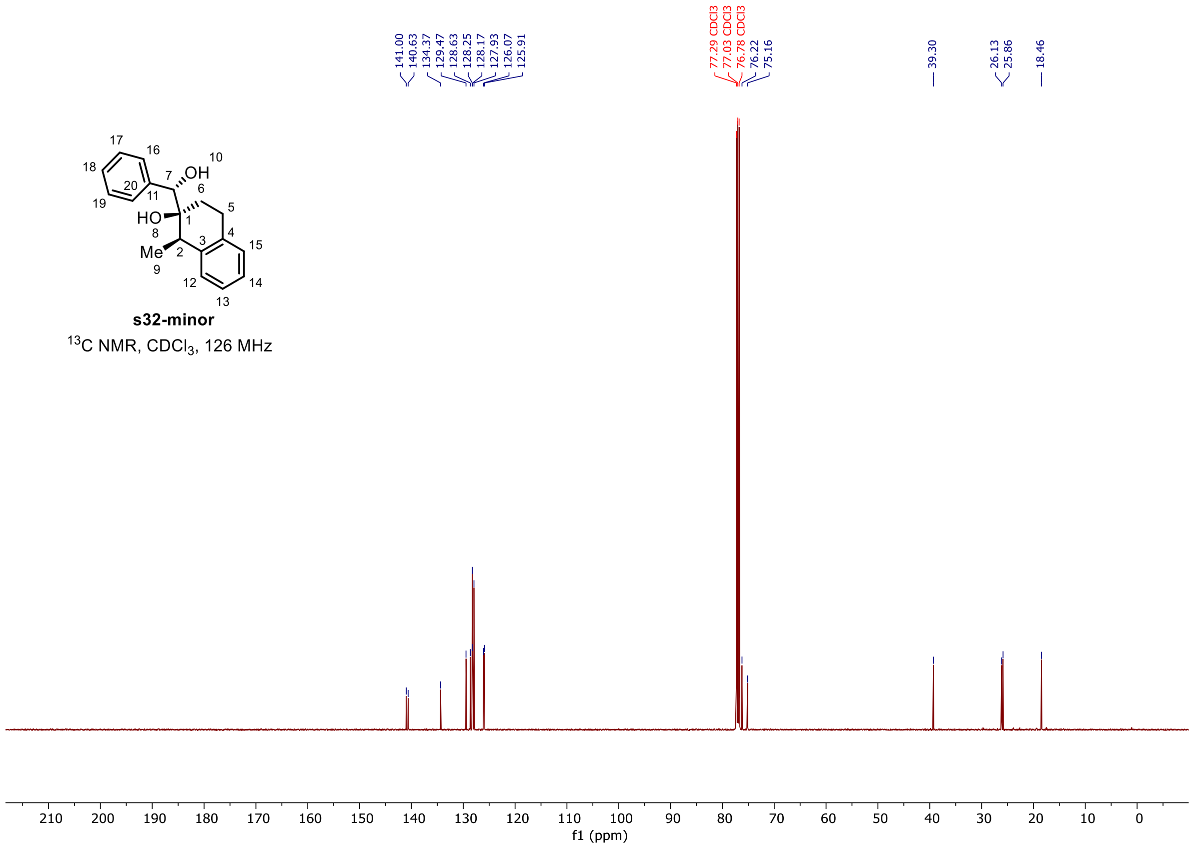

(*E*)-(*trans*)-2-Hydroxy-1-methyl-1,2,3,4-tetrahydronaphthalen-2-yl)(phenyl)methanone oxime s33

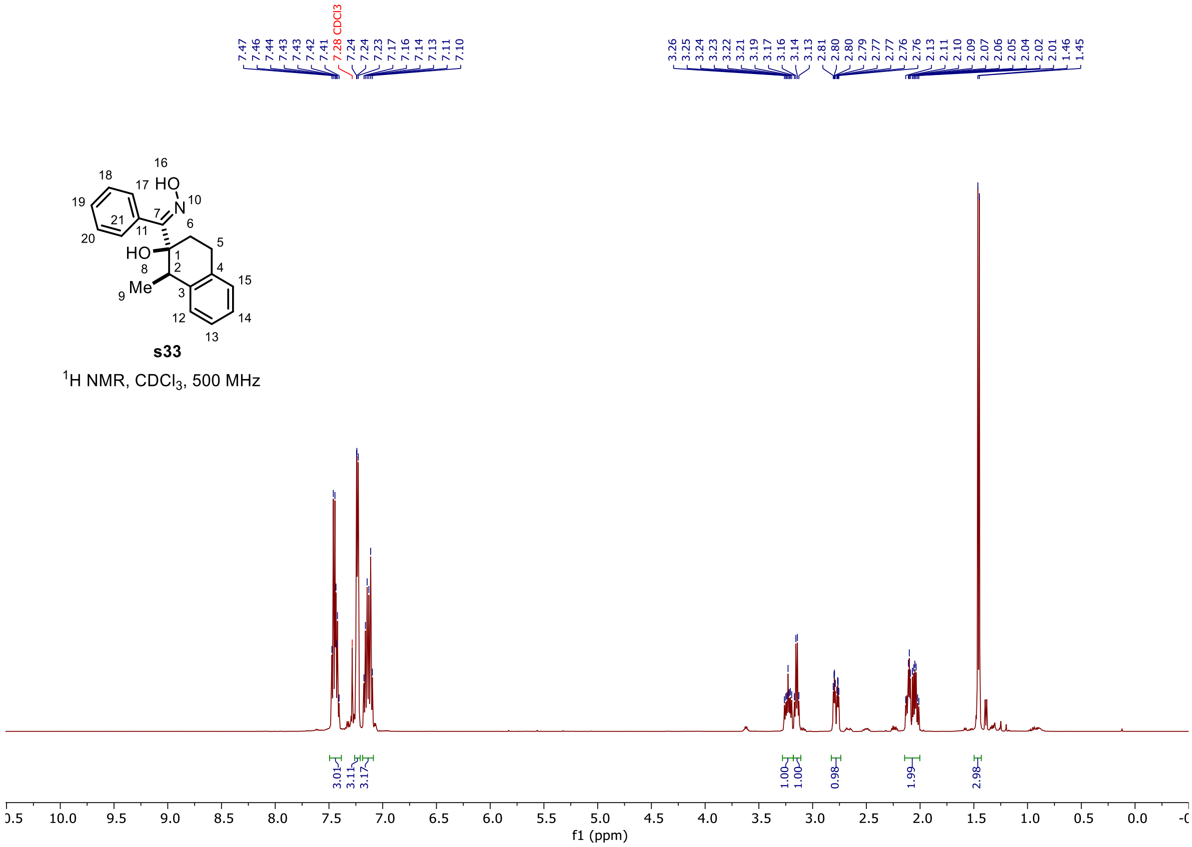

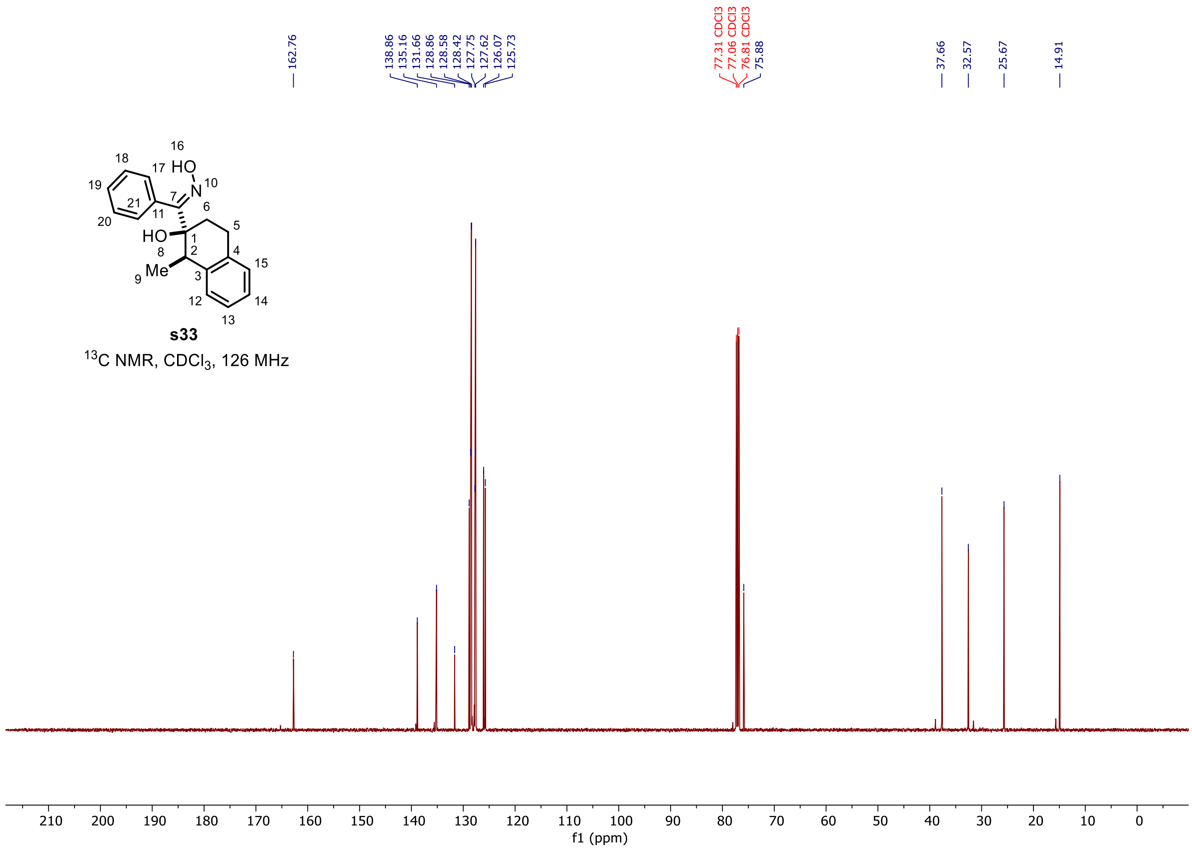

((1*R*,2*S*)-2-(benzyloxy)-1-methyl-1,2,3,4-tetrahydronaphthalen-2-yl)(phenyl)methanone Bn-8m

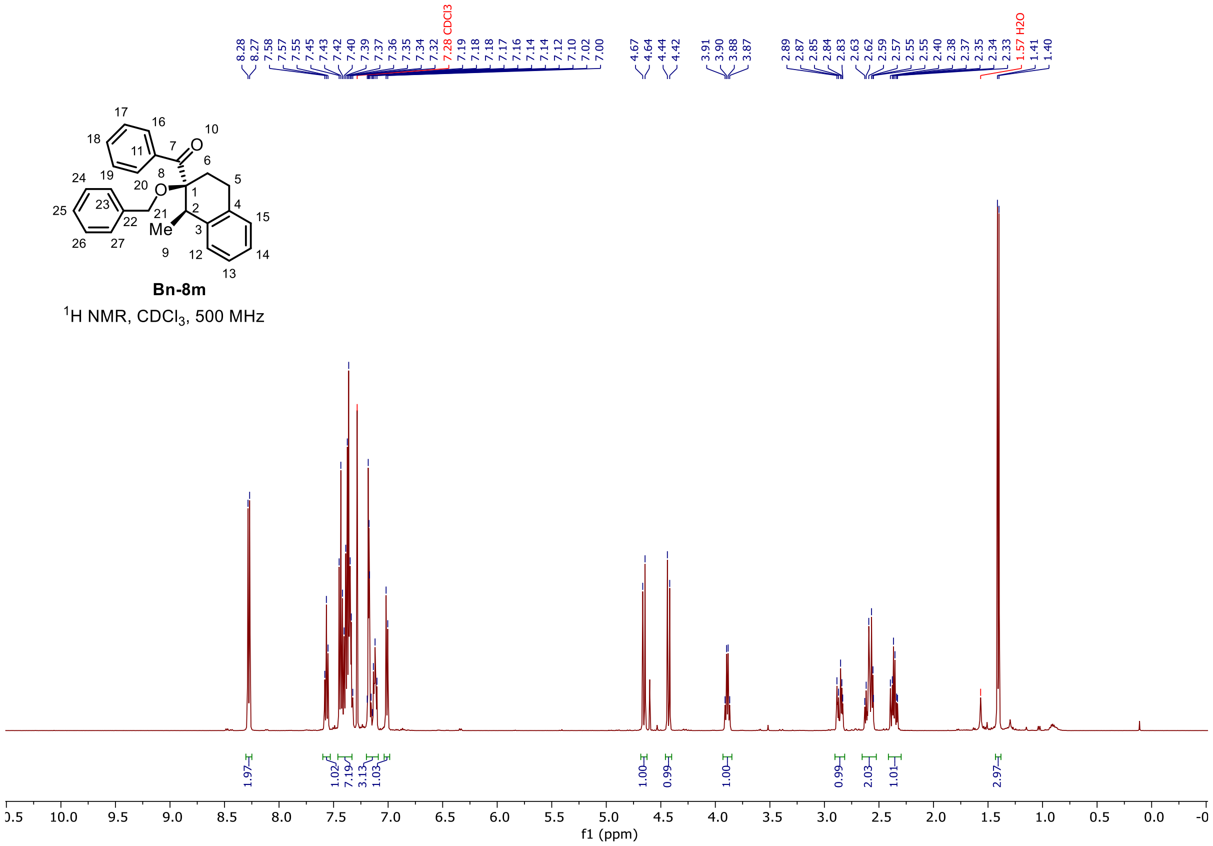

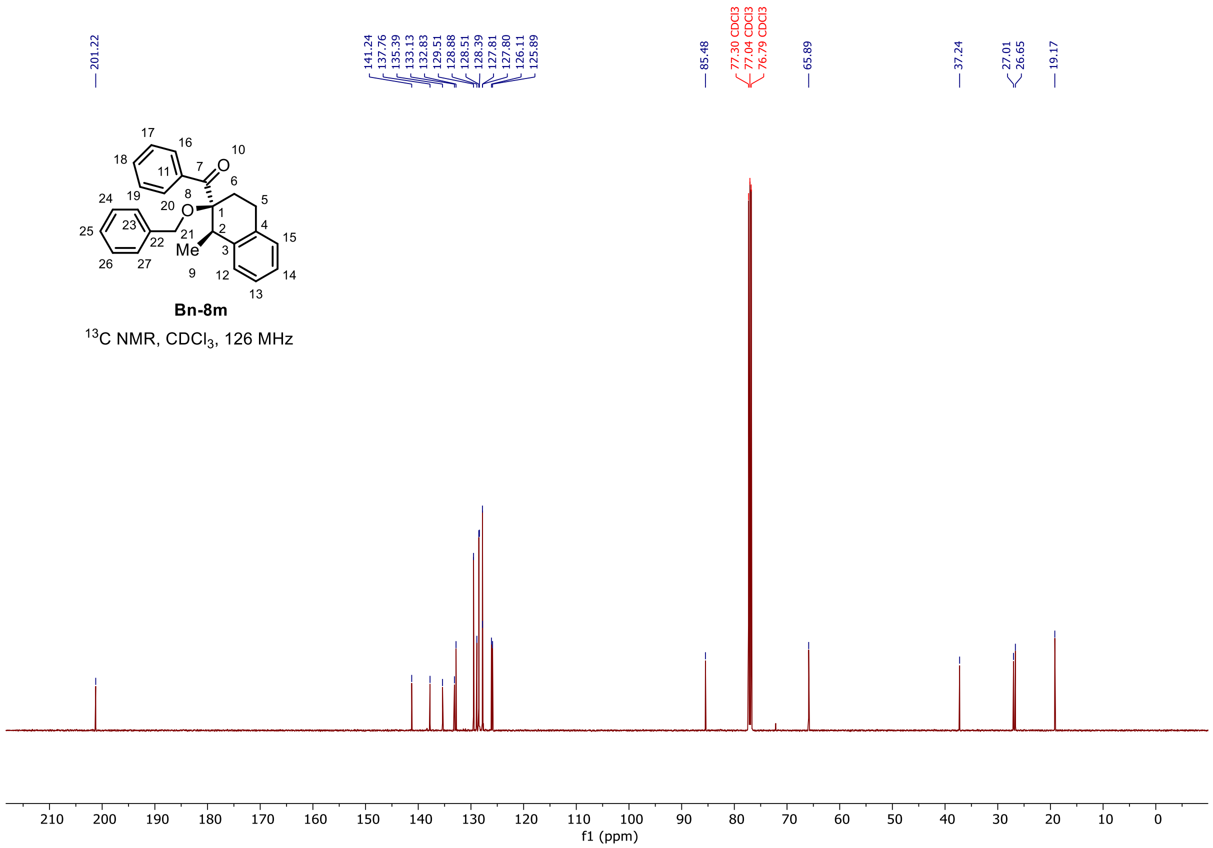

(1*R*,2*S*)-2-(Benzyloxy)-1-methyl-2-(1-phenylvinyl)-1,2,3,4-tetrahydronaphthalene s34

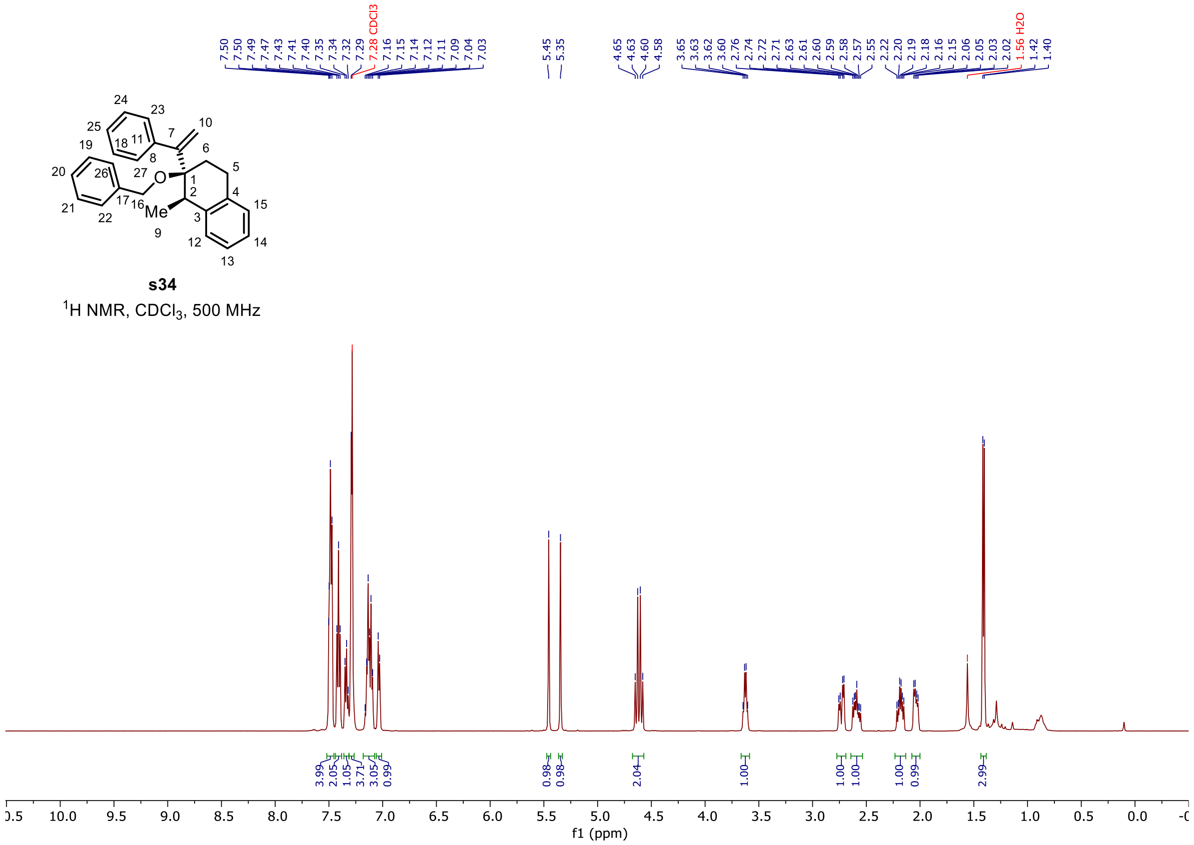

s34

^{1}H NMR, $CDCl_3$, 500 MHz

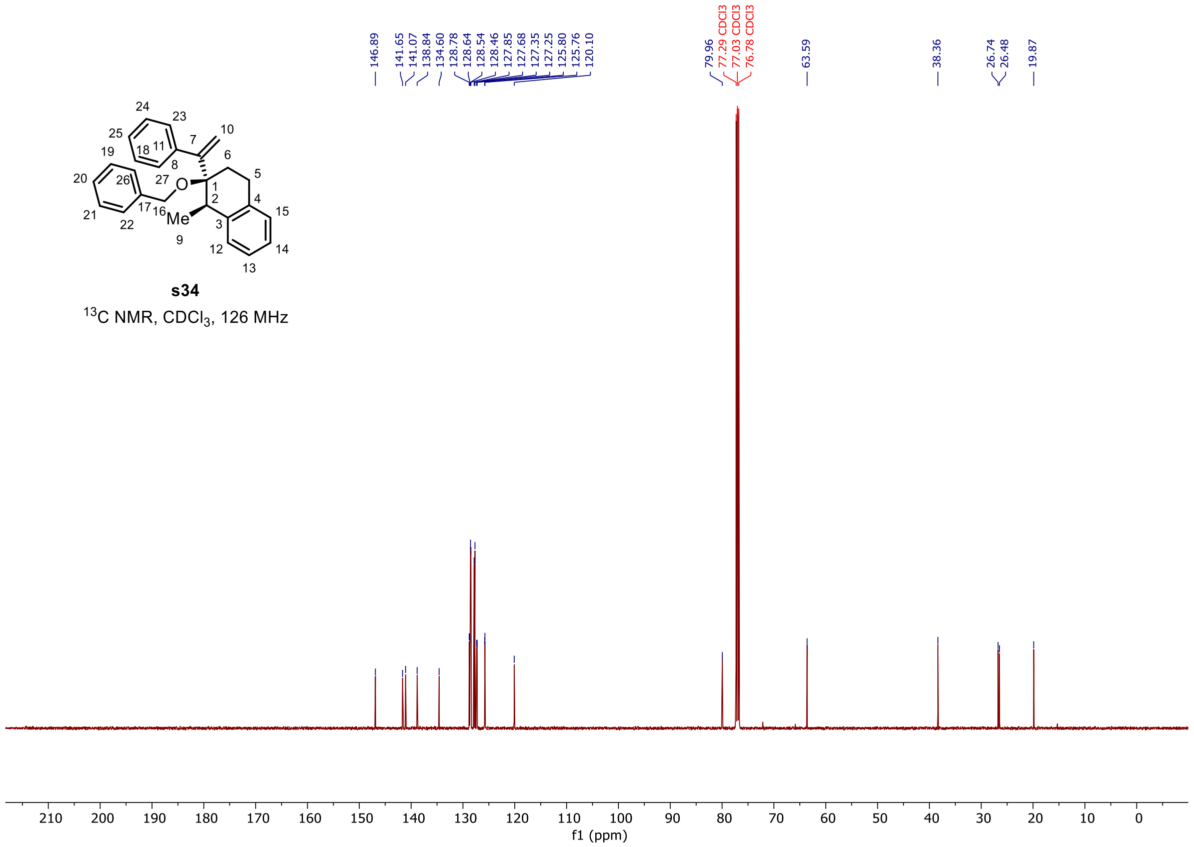

s34

^{13}C NMR, $CDCl_3$, 126 MHz

Mechanistic studies

2-Hydroxy-2-methyl-1-phenylhept-6-en-1-one s35

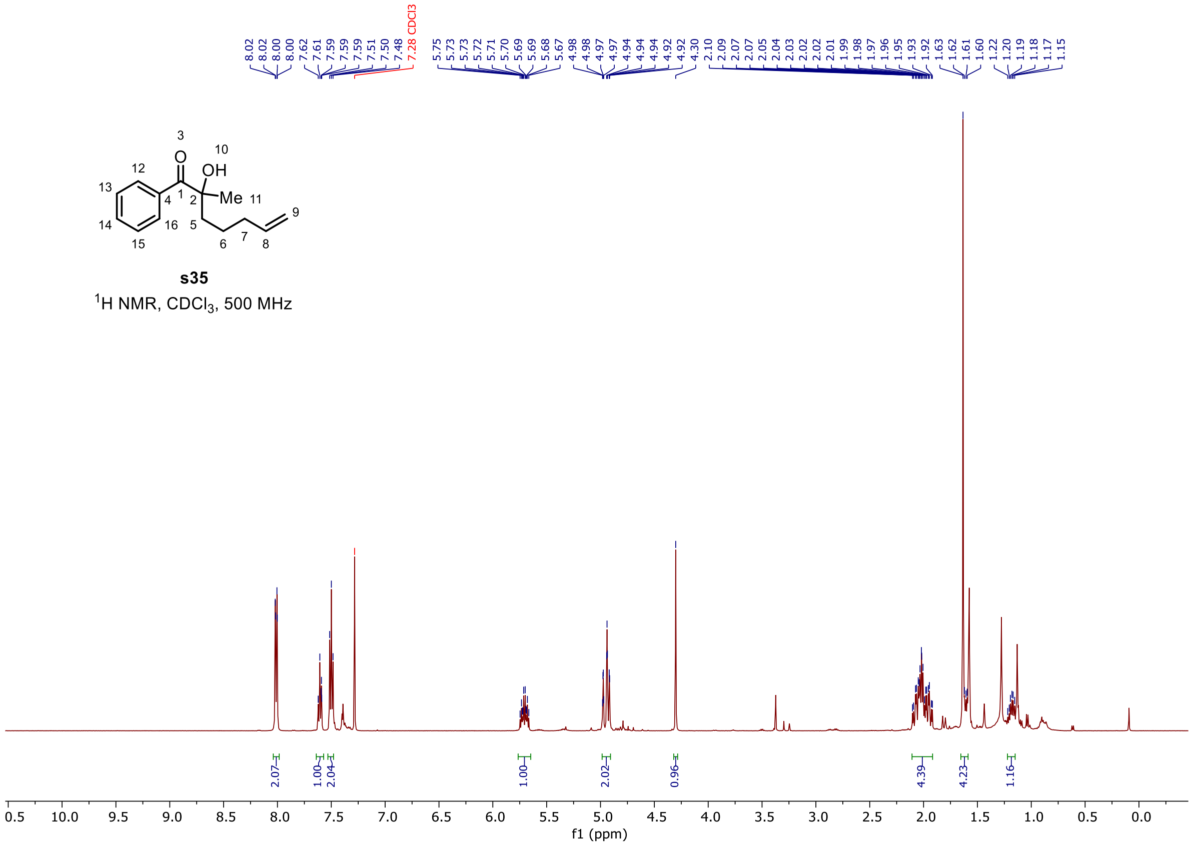

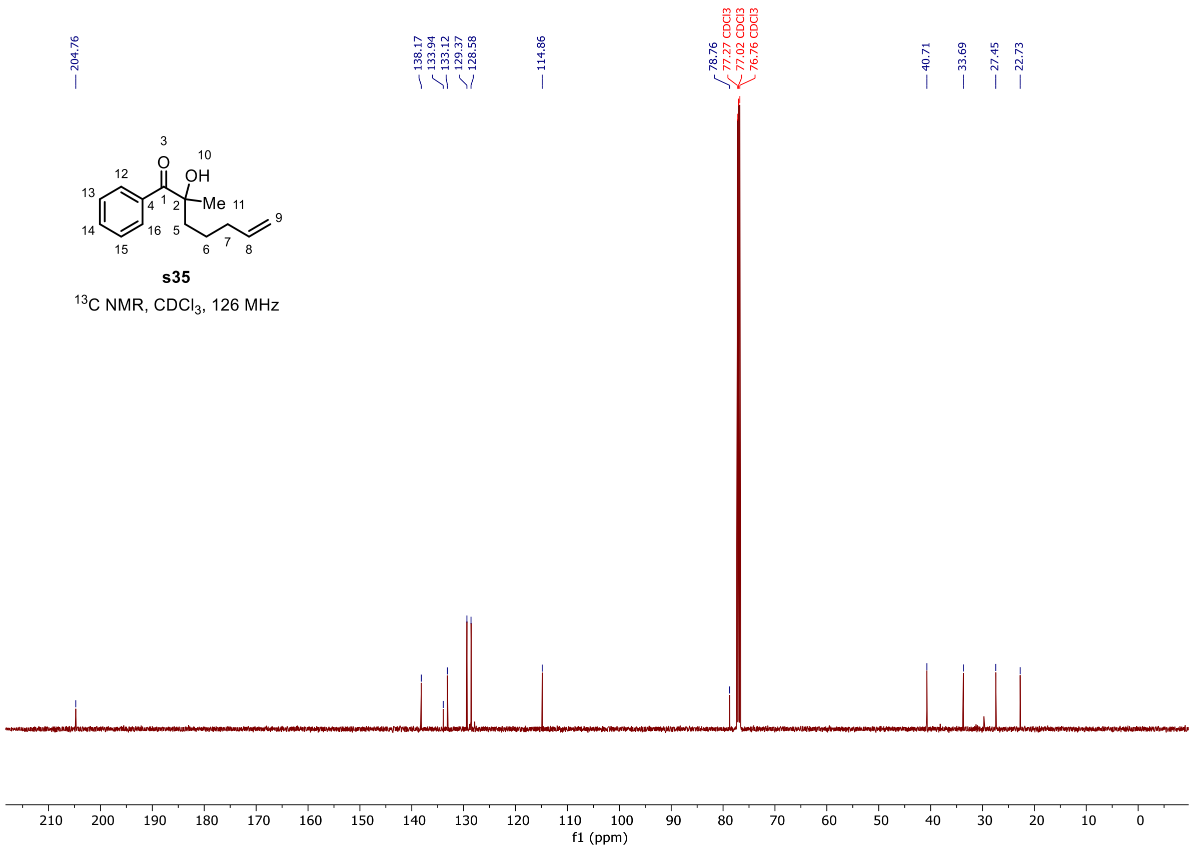

2-Hydroxy-2-phenyloct-7-en-3-one s36

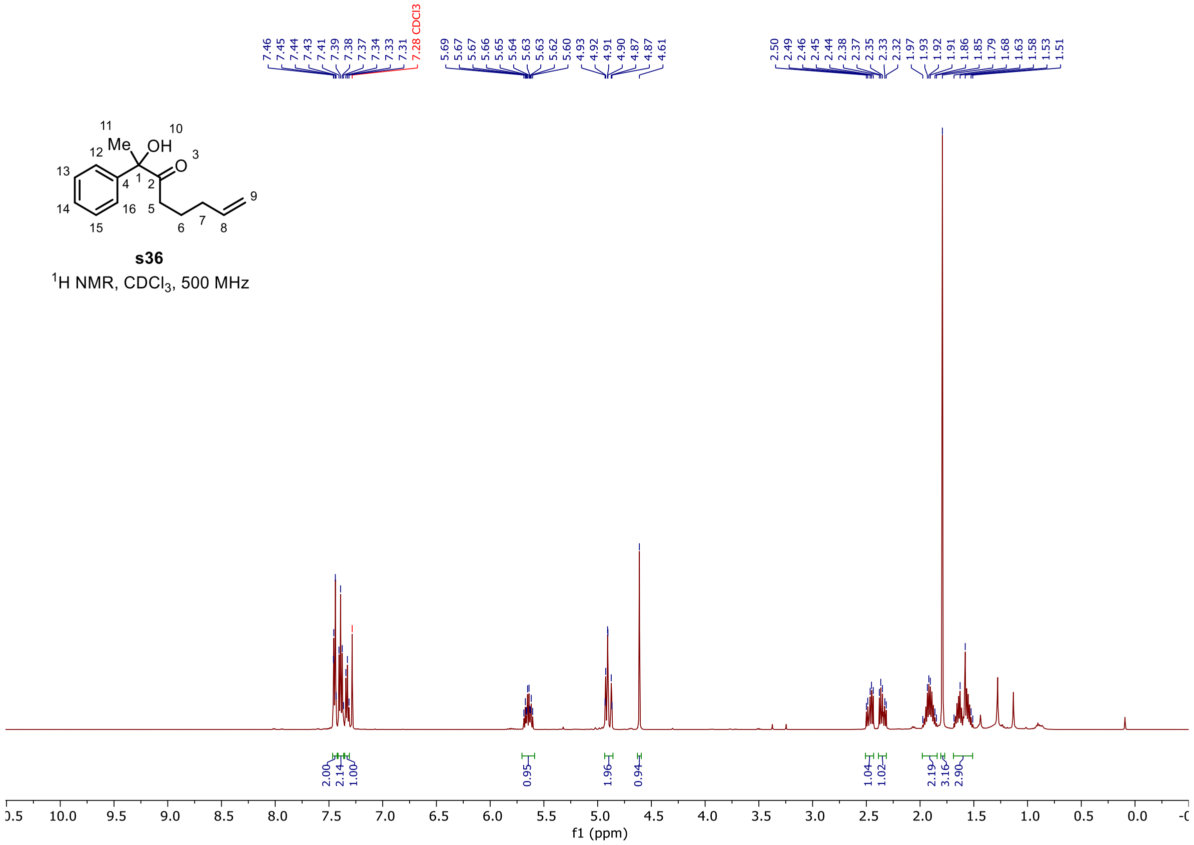

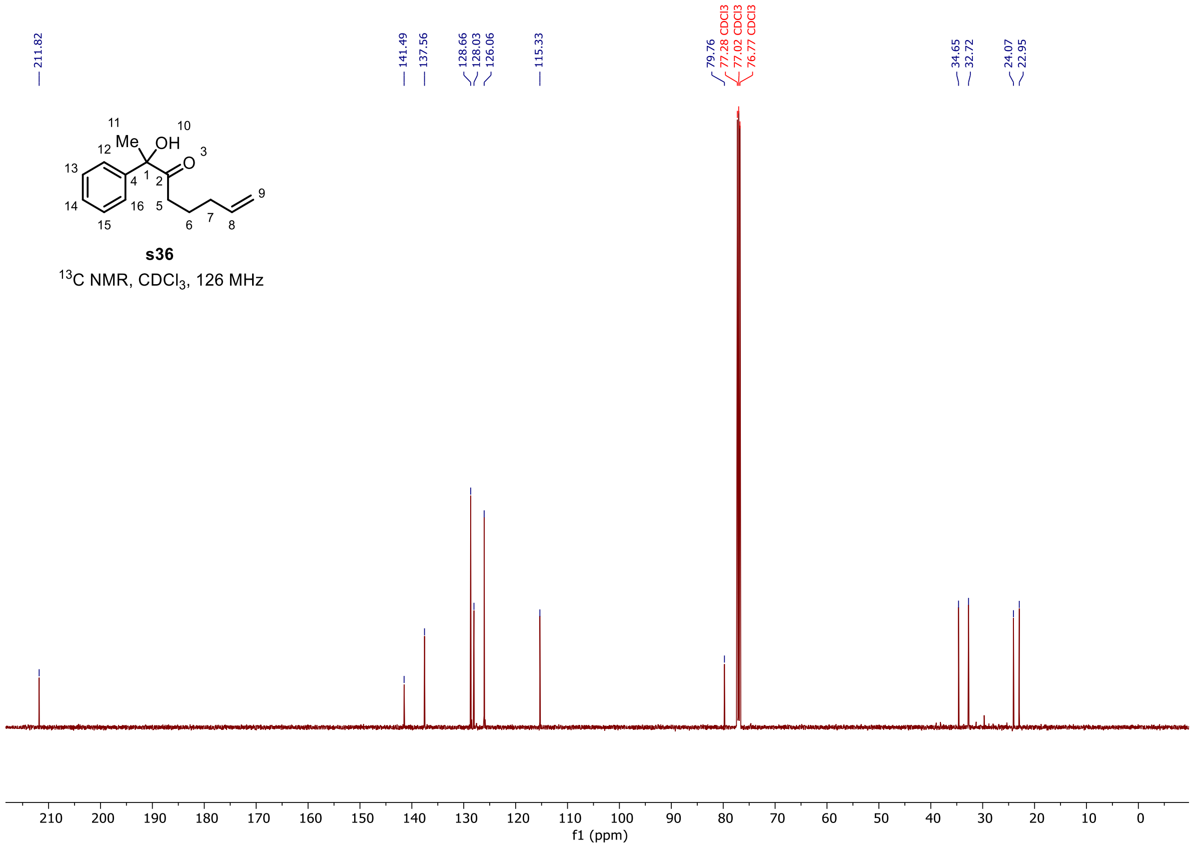

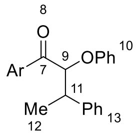
8
O
Ar
7
9
OPh
10
11
Me
12
Ph
13

OPh-3a

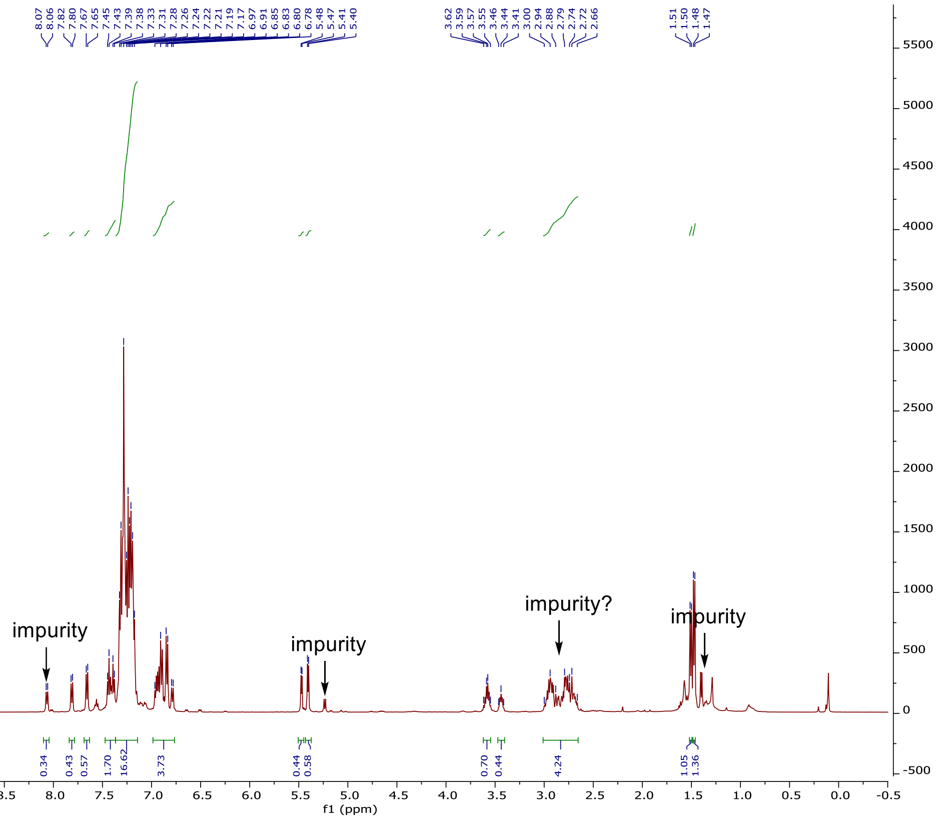
8.07
8.06
7.82
7.80
7.67
7.65
7.45
7.43
7.39
7.38
7.33
7.31
7.28
7.26
7.24
7.22
7.21
7.19
7.17
6.97
6.91
6.85
6.83
6.80
6.78
5.48
5.47
5.41
5.40
3.62
3.59
3.57
3.55
3.46
3.44
3.41
3.00
2.94
2.88
2.79
2.74
2.72
2.66
1.51
1.50
1.48
1.47
impurity
impurity
impurity?
impurity
0.34
0.43
0.57
1.70
16.62
3.73
0.44
0.58
0.70
0.44
4.24
1.05
1.36
f1 (ppm)

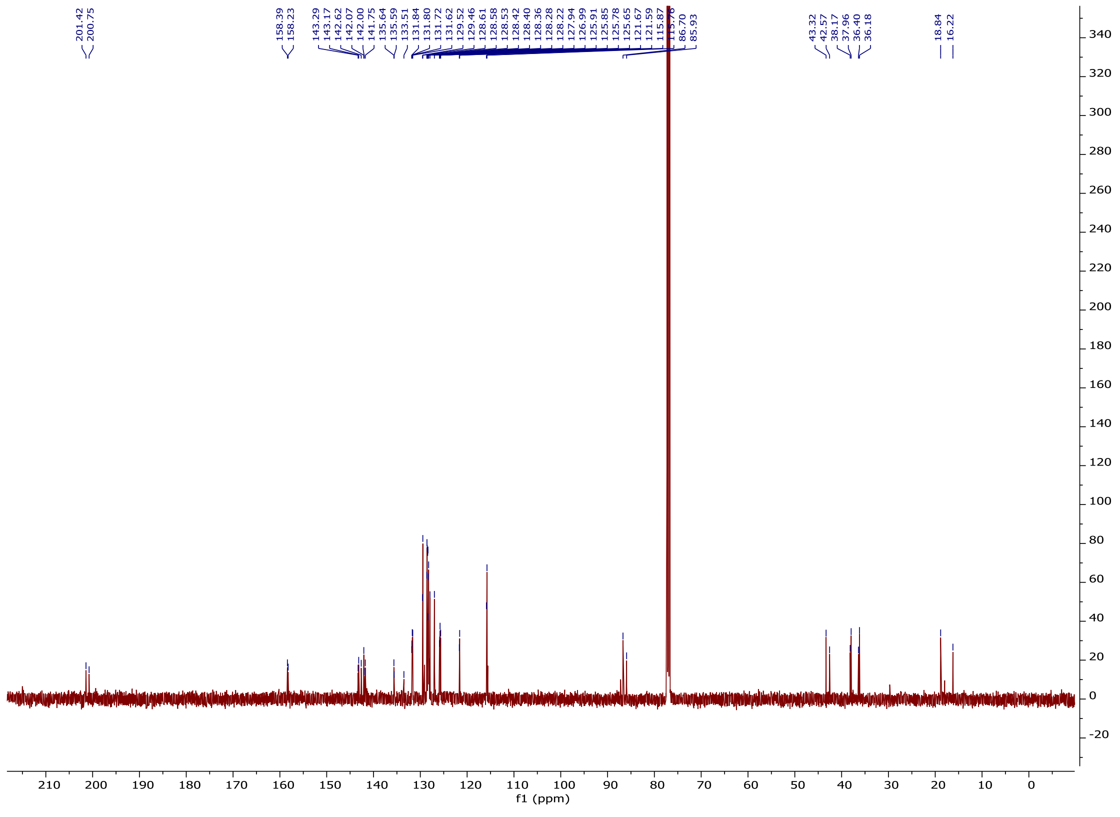
201.42
200.75
158.39
158.23
143.29
143.17
142.62
142.07
142.00
141.75
135.64
135.59
133.51
131.84
131.80
131.72
131.62
129.52
129.46
128.61
128.58
128.53
128.42
128.40
128.36
128.28
128.22
127.94
126.99
126.91
125.91
125.85
125.78
125.65
121.67
121.59
115.87
86.70
85.93
43.32
42.57
38.17
37.96
36.40
36.18
18.84
16.22
f1 (ppm)

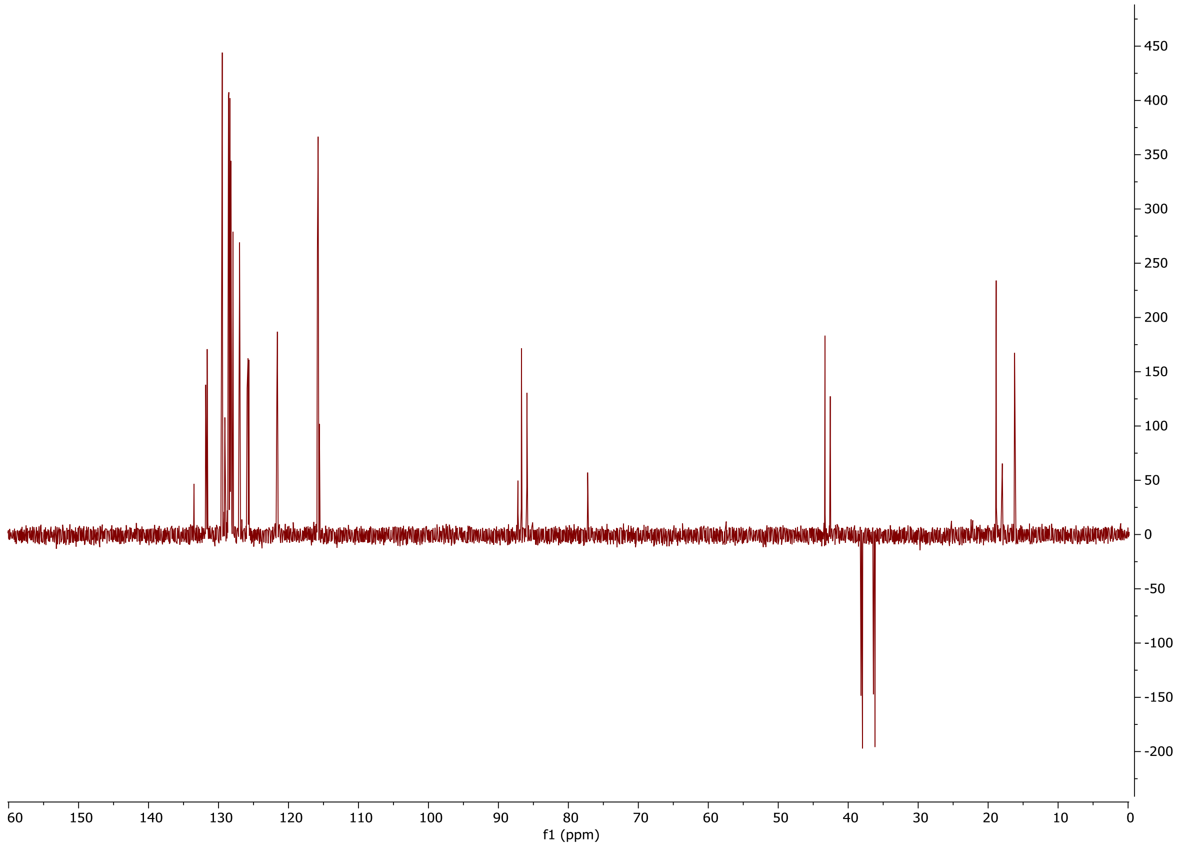

450
400
350
300
250
200
150
100
50
0
-50
-100
-150
-200
60
150
140
130
120
110
100
90
80
70
60
50
40
30
20
10
0
f1 (ppm)

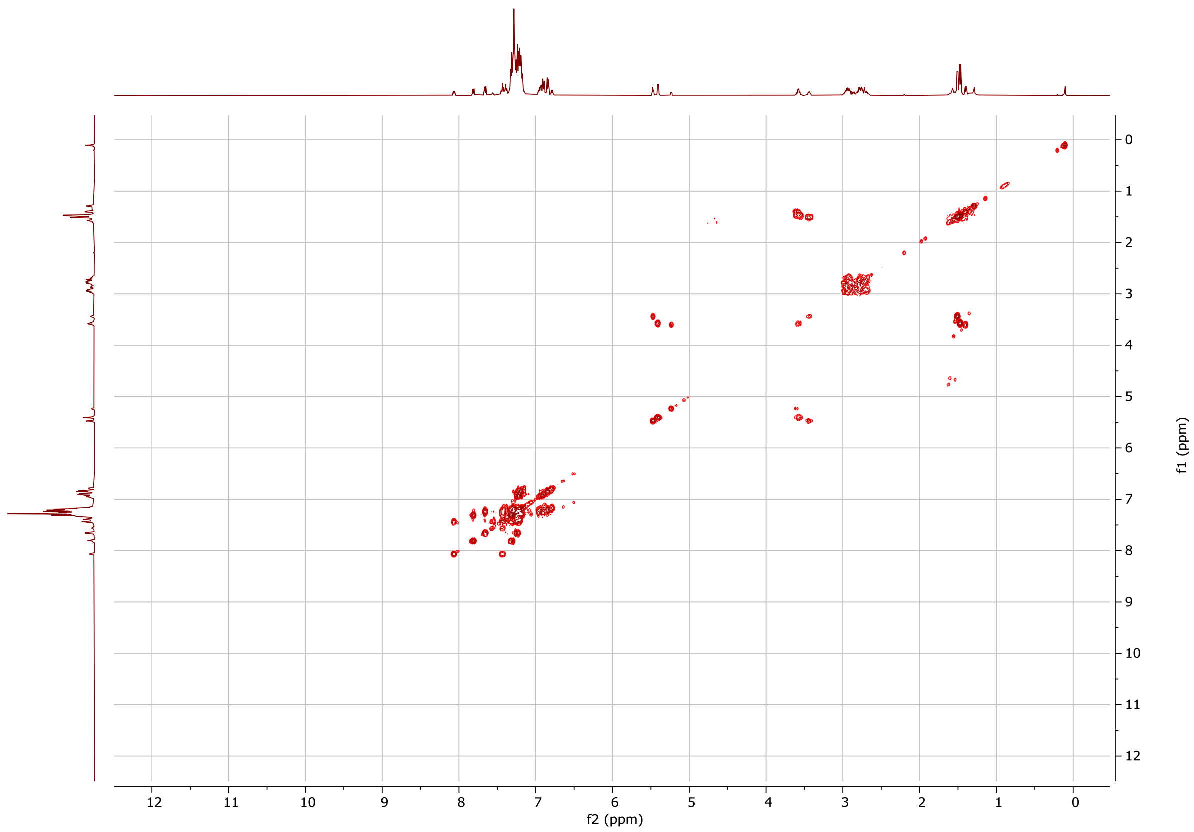

0
1
2
3
4
5
6
7
8
9
10
11
12
f1 (ppm)
12
11
10
9
8
7
6
5
4
3
2
1
0
f2 (ppm)

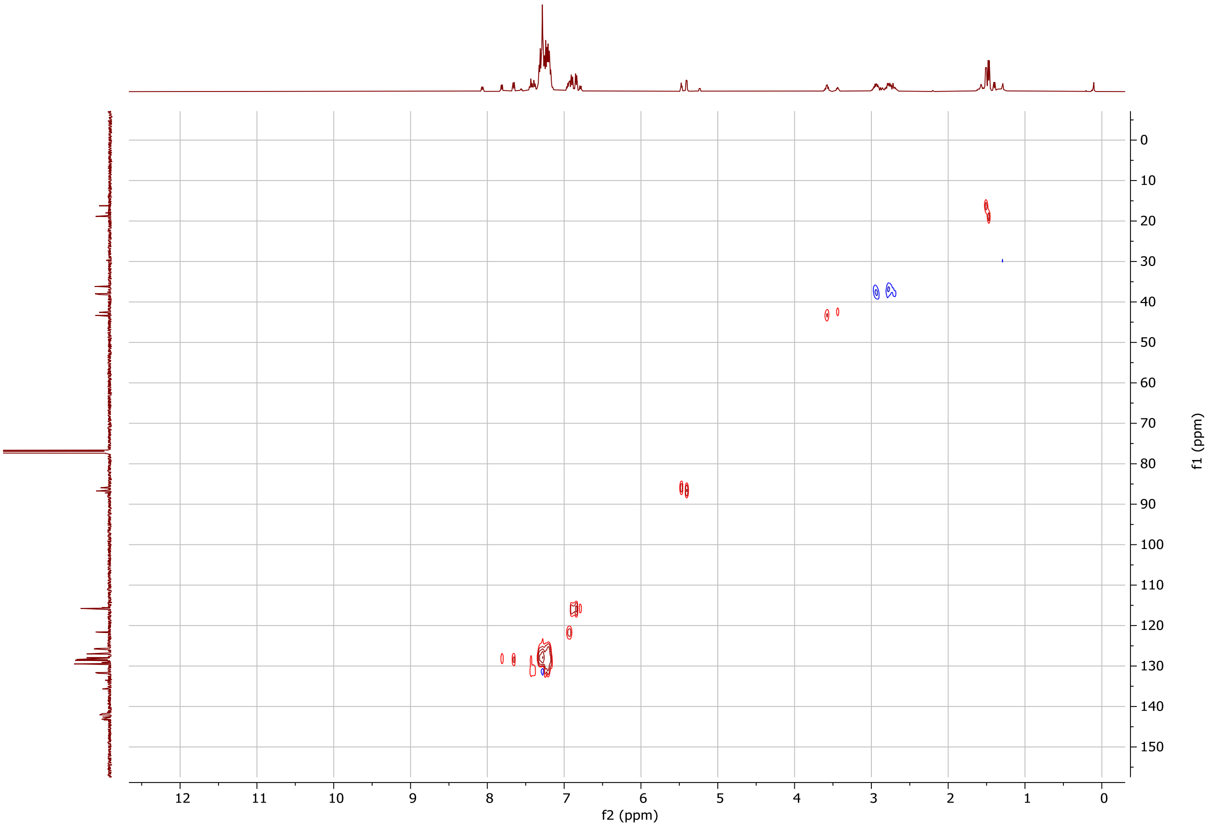
f2 (ppm)
f1 (ppm)

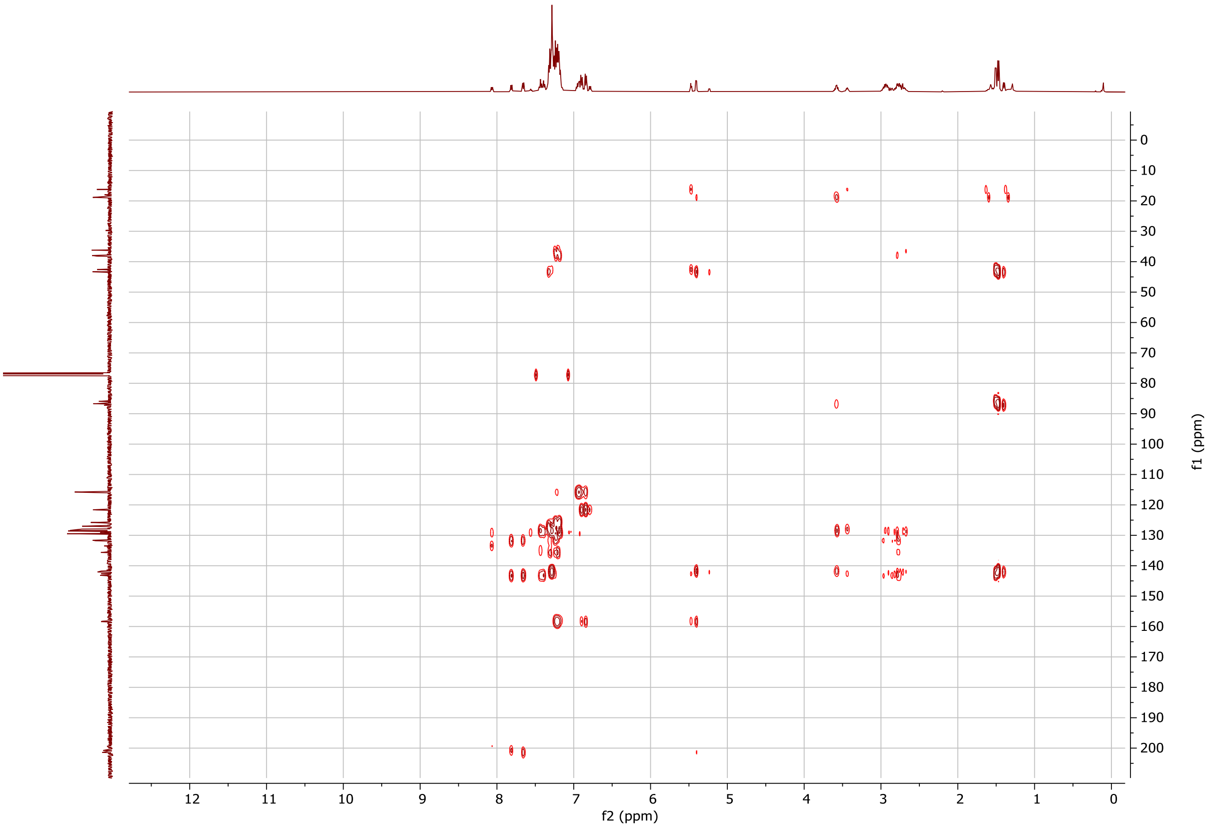
f2 (ppm)
f1 (ppm)

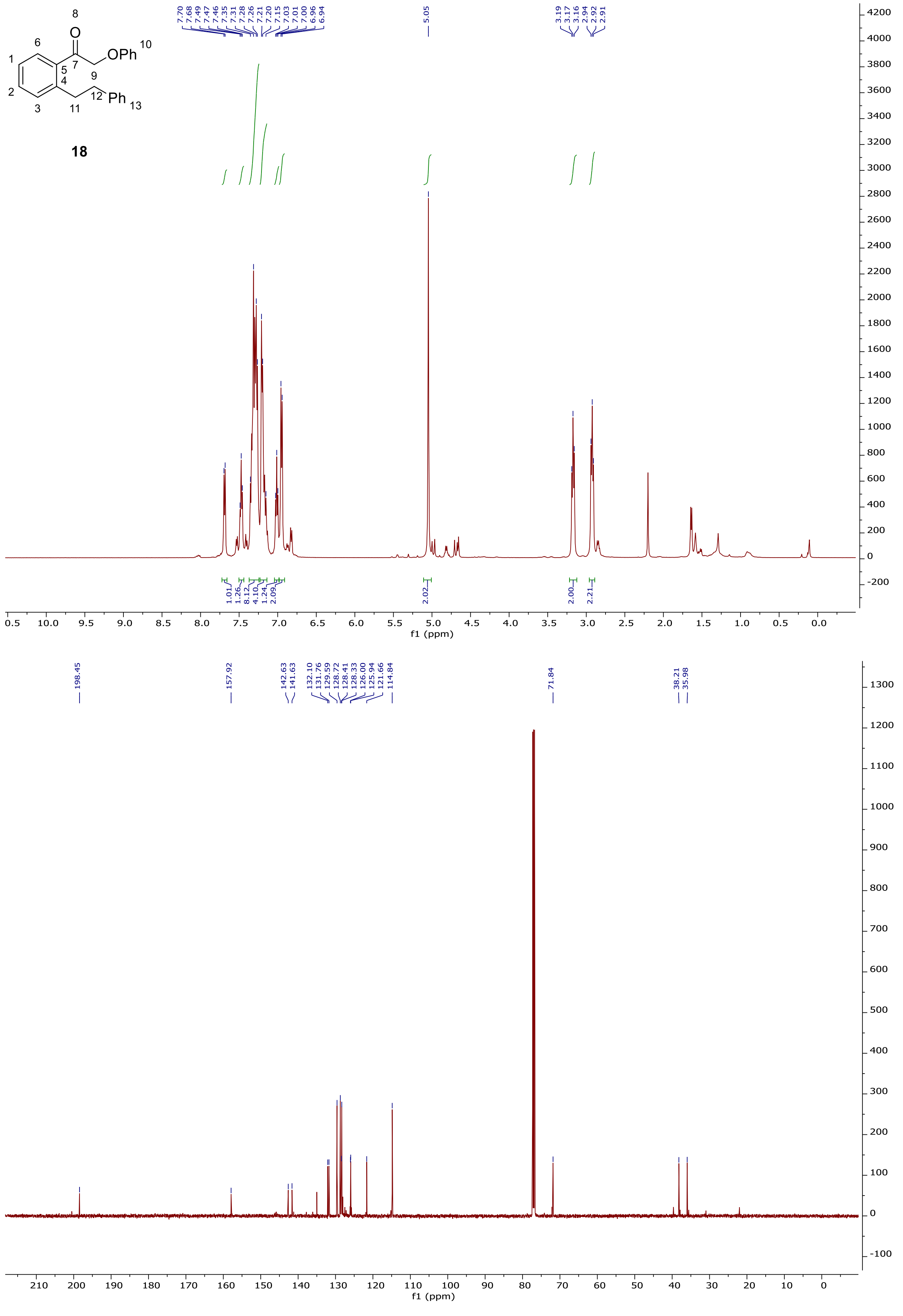
18
7.70 7.68 7.49 7.47 7.46 7.35 7.31 7.28 7.26 7.21 7.20 7.15 7.03 7.01 7.00 6.96 6.94
5.05
3.19 3.17 3.16 2.94 2.92 2.91
1.01 1.26 8.12 4.10 1.24 2.09
2.02
2.00 2.21
f1 (ppm)
198.45
157.92
142.63 141.63
132.10 131.76 129.59 128.72 128.41 128.33 126.00 125.94 121.66 114.84
71.84
38.21 35.98
f1 (ppm)

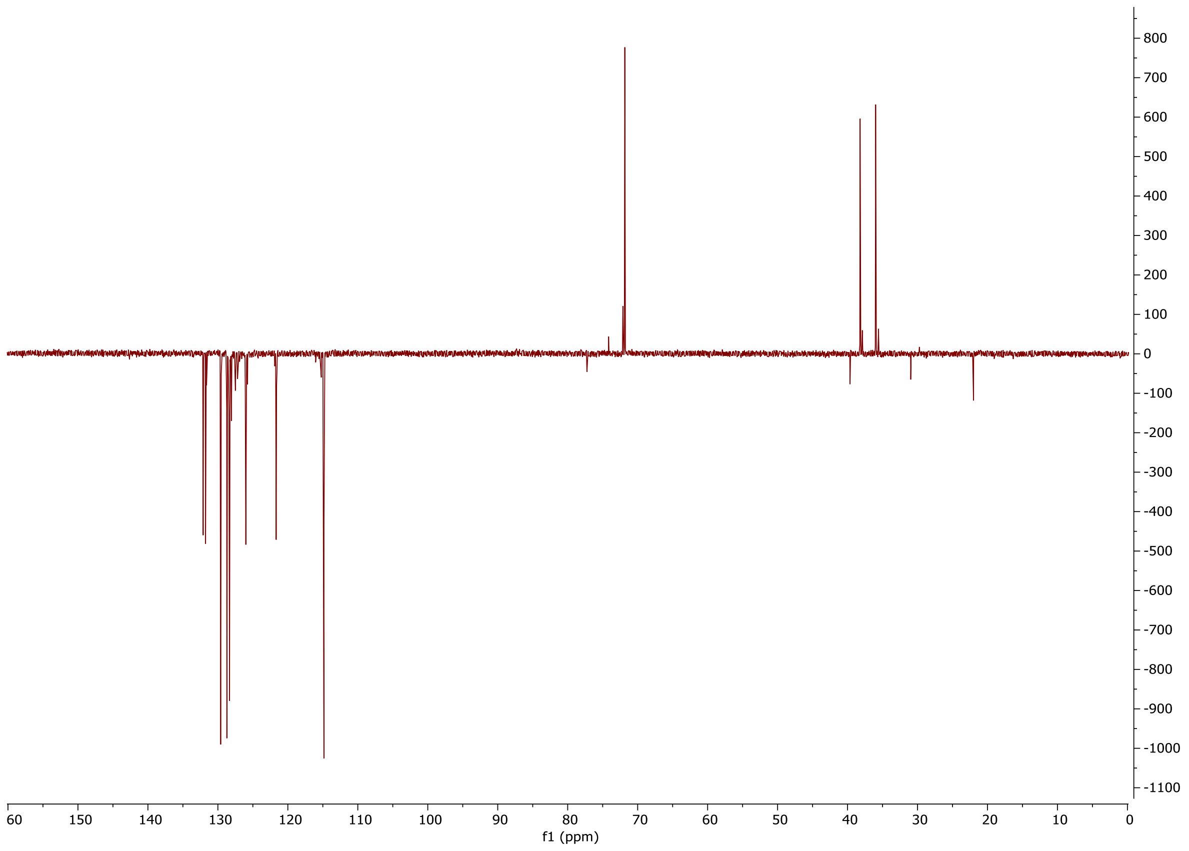
f1 (ppm)

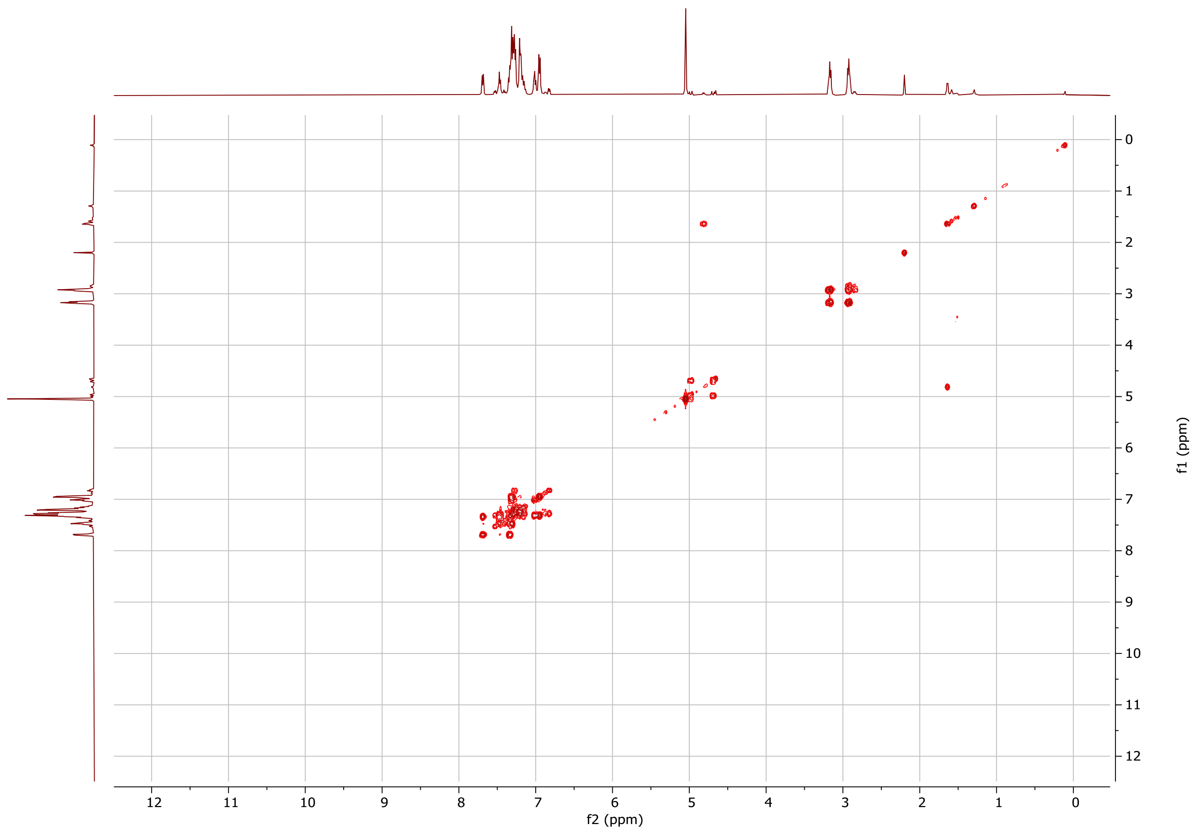
f2 (ppm)
f1 (ppm)

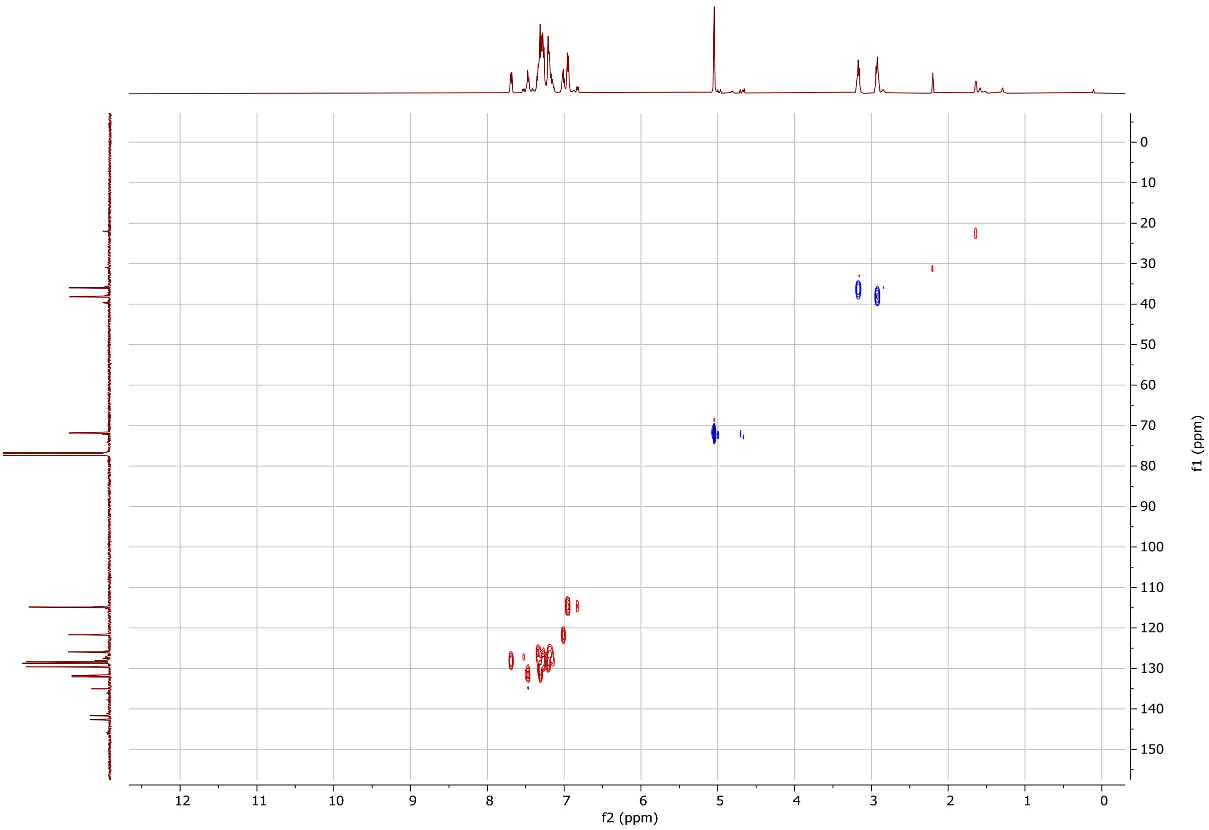
f2 (ppm)
f1 (ppm)

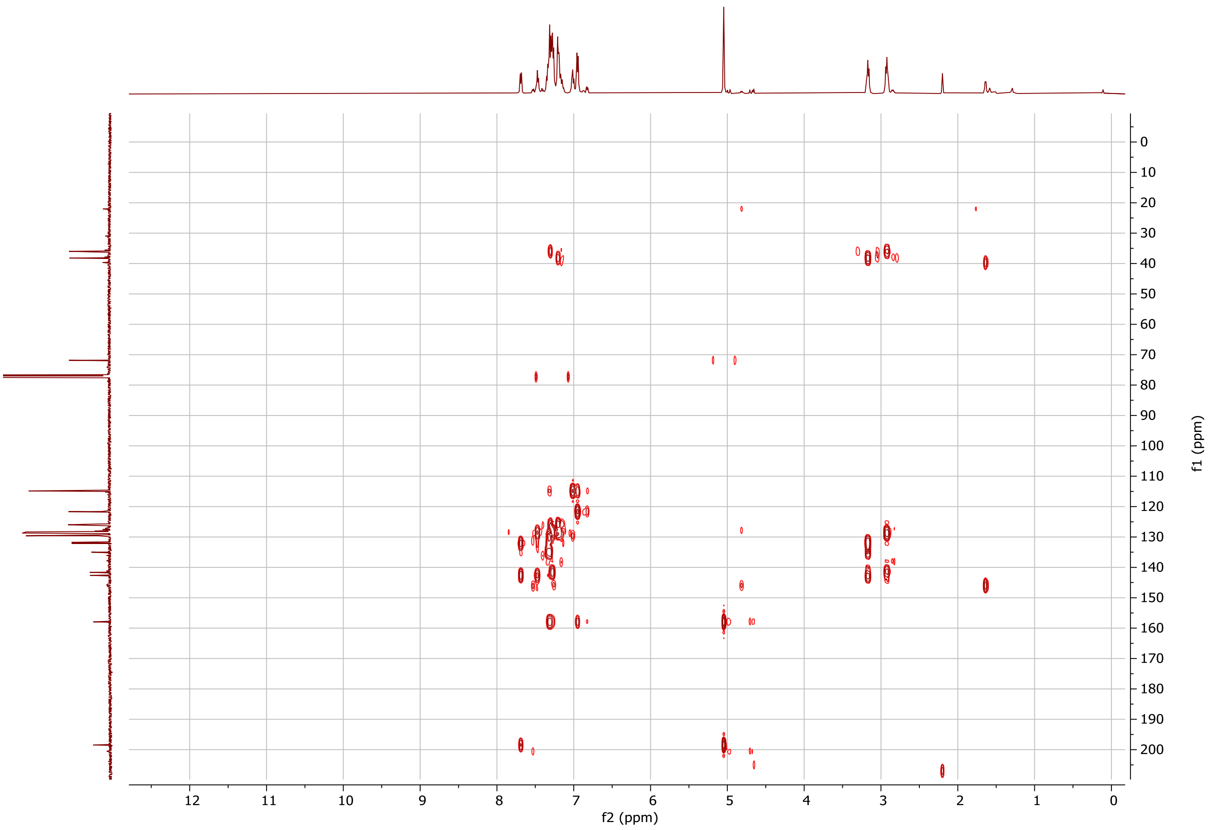
f2 (ppm)
f1 (ppm)
